# Stabilized Carbon Radical‐Mediated Assembly of Arylthianthrenium Salts, Alkenes and Amino Acid/Peptide Derivatives

**DOI:** 10.1002/advs.202411579

**Published:** 2024-11-21

**Authors:** Bo Dong, Weiguan Qi, Yifeng Chen, Yufei Zhang, Shiyu Gu, Jianlin Zhao, Qingfa Zhou, Jian Shen, Lan‐Gui Xie

**Affiliations:** ^1^ National and Local Joint Engineering Research Center of Biomedical Functional Materials, Jiangsu Key Laboratory of New Power Batteries, School of Chemistry and Materials Science Nanjing Normal University Nanjing 210023 P. R. China; ^2^ State Key Laboratory of Natural Medicines Department of Organic Chemistry China Pharmaceutical University Nanjing 210009 P. R. China; ^3^ Jiangsu Engineering Research Center of Interfacial Chemistry Nanjing University Nanjing 210023 P. R. China

**Keywords:** amino acid, captodative effect, late‐stage functionalization, radical coupling, thianthrenium salt

## Abstract

Efficiently assembling amino acids and peptides with bioactive molecules facilitates the modular and streamlined synthesis of a diverse library of peptide‐related compounds. Particularly notable is their application in pharmaceutical development, leveraging site‐selective late‐stage functionalization. Here, a visible light‐induced three‐component reaction involving arylthianthrenium salts, amino acid/peptide derivatives, and alkenes are introduced. This approach utilizes captodatively‐stabilized carbon radicals to enable radical‐radical C─C coupling, effectively constructing complex bioactive molecules. This method offers a promising alternative route for modular synthesis of peptide‐derived bio‐relevant compounds

## Graphical abstract

Photoredox‐catalyzed three‐component arylative alkylation of amino acid and peptide derivatives using aryl thianthrenium salts and alkenes is achieved. Key to its success is the radical‐radical coupling enabled by captodatively‐stabilized carbon radical of glycine derivatives. This protocol provides a straightforward pathway for integrating pharmacologically relevant fragments and amino acids/peptides through formal dual C─H functionalization, highlighting its potential for peptide‐drug development.

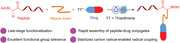

## 1 Introduction

Peptide‐derived drugs offer distinct advantages over small or large‐molecule medicines, including reduced immunogenicity, lower production cost, diminished potency, side effect, and enhanced selectivity and specificity.^[^ ^1^ ^]^ Consequently, they are increasingly garnering attentions. Since the introduction of therapeutic insulin in the early 20th century, the conjugation of drug molecules with amino acids and peptides has held a pivotal role in advancing novel drug development and has become integral to medical practice.^[^ ^2^ ^]^ Presently, over a hundred peptide‐drug molecules have received global approval and are extensively utilized in treating conditions such as diabetes, tumors, chronic pain, and multiple sclerosis.^[^ ^3^ ^]^ Common technologies employed for conjugating peptides with bioactive molecules include ADCs (Antibody‐Drug Conjugates),^[^ ^4^ ^]^ PDCs (Peptide‐Drug Conjugates),^[^ ^5^ ^]^ and PROTACs (Proteolysis Targeting Chimeras)^[^ ^6^ ^]^. Recent years have witnessed substantial efforts to advance strategies for modifying amino acids and peptides.^[^ ^7^ ^]^ These endeavors have yielded significant breakthroughs, particularly in the field of site‐selective C─H alkylation/arylation, underscoring its importance in synthesizing bio‐relevant molecules.

Site‐selective functionalization of aryl C−H bonds holds significant promise for complex molecule synthesis and late‐stage functionalization.^[^ ^8^ ^]^ Over the past decade, the use of aryl sulfonium salts (ArTT^+^/DBT^+^/PXT^+^, TT═thianthrene, DBT═dibenzo[*b,d*]‐thiophene, PXT═phenoxathiin) derived from arenes has emerged as a robust method for regioselectively activating aryl C−H bonds. This approach has demonstrated exceptional potentials in constructing both C(*sp^2^*)−C^[^ ^9^ ^]^ and C(*sp^2^)*−X^[^ ^10^ ^]^ bonds. By leveraging aryl sulfonium salts as precursors to aryl radicals, sequential reactions with C═C bonds and radical acceptors enable late‐stage functionalization and three‐component reactions, facilitating rapid library construction of complex molecules due to the versatility of these transformations.^[^ ^11^ ^]^

**Figure 1:**
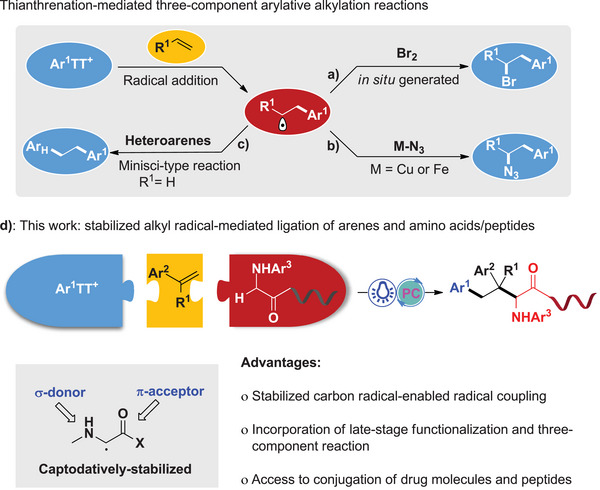
Radical three‐component reactions of aryl sulfonium salts.

Various types of radical acceptors have been developed for this purpose (**Figure** **1**a). Ritter's group has reported the use of in situ generated bromine (Figure 1a)^[^ ^12^ ^]^ and azido‐copper(II) complexes (Figure 1b),^[^ ^13^ ^]^ opening new pathways for bromoalkylation and azidoalkylation of arylthianthrenium salts, respectively. Koh/Wu et al. demonstrated that azido‐iron(III) intermediates intercept carbon radicals generated from the interaction of aryl sulfonium salts and alkenes (Figure 1b).^[^ ^14^ ^]^ Recently, Wu's group achieved modular synthesis of 1,2‐arylheteroaryl ethanes by employing heteroarenes in Minisci‐type reactions with carbon radical intermediates (Figure 1c).^[^ ^15^ ^]^ Additionally, we have shown that a reductive radical‐polar crossover process could couple arylthianthrenium salts with styrenes and CO_2_.^[^ ^11c^ ^]^ Despite these advances, reports on coupling alkyl radicals, which are crucial intermediates for C−C bond formation, with radical intermediates formed from aryl sulfonium salts and alkenes remain scarce.

Given the paramount importance of C(*sp^3^*)‐H alkylation of amino acid/peptide derivatives and late‐stage functionalization in modern drug development, we sought to combine these approaches. In our prior investigations, we identified that the carbon radicals derived from glycine derivatives exhibited exceptional stabilities by the captodative effect,^[^ ^16^ ^]^ facilitating radical‐radical type C−C coupling with transient carbon radicals. Based on this finding, we achieved the modular synthesis of sulfur‐containing amino acid and peptide derivatives through a three‐component radical coupling involving disulfanes, styrenes, and *N*‐aryl glycinates.^[^ ^17^ ^]^ Here, we present our findings focusing on stabilized alkyl radical‐enabled conjugation of amino acid/peptide derivatives with drug molecules, utilizing alkenes as linkers in a formal dual C─H (*sp^3^* and *sp^2^*) functionalization strategy (Figure 1d). This protocol entails the efficient assembly of *N*‐aryl glycine derivatives, alkenes, and arylthianthrenium salts under photoredox conditions.

## 2 Results and Discussion

The reaction development commenced with an evaluation using *N*‐phenylglycine ethyl ester **1a** and aryl thianthrenium salt **2a** as model substrates, with styrene **3a** serving as the linker precursor under blue light emitting diode (LED) irradiation (**Table** **1**). Employing 4CzIPN as the photocatalyst and acetonitrile as the initial solvent afforded glycine derivative **4aa** in 59% yield (entry 1). Optimization with various solvents revealed dimethyl sulfoxide (DMSO) to be optimal (entries 1–5), yielding **4aa** in 69%. The addition of base additives significantly influenced the reaction, with pyridine proving superior and enhancing the yield of **4aa** to 77% (entries 6–8). Alternative photocatalysts such as Eosin Y and complexes based on ruthenium and iridium showed diminished efficacy (entries 9–11). Control experiments underscored the indispensable roles of both light and the photocatalyst in achieving the desired product (entries 12 and 13).

**Table 1:** Optimization of reaction conditions.^a)^

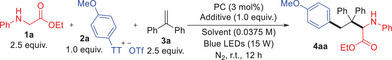				
Entry	PC	Solvent	Additive	Yield [%]^b)^
1	4CzIPN	MeCN	No	59%
2	4CzIPN	DCM	No	43%
3	4CzIPN	DMF	No	31%
4	4CzIPN	THF	No	19%
5	4CzIPN	DMSO	No	69%
6	4CzIPN	DMSO	K_2_CO_3_	50%
7	4CzIPN	DMSO	Cs_2_CO_3_	30%
8	4CzIPN	DMSO	Py	77%
9	Eosin Y	DMSO	Py	Trace
10	Ru(ppy)_3_PF_6_	DMSO	Py	53%
11	Ir[dF(CF_3_)ppy]_2_(dtbpy)PF_6_	DMSO	Py	61%
12	‐	DMSO	Py	Trace
13^c)^	4CzIPN	DMSO	Py	N.D.

^a)^ Standard conditions: **1a** 0.375 mmol (2.5 equiv.), **2a** 0.15 mmol (1.0 equiv.), **3a** 0.375 mmol (2.5 equiv.), PC 0.0045 mmol (3 mol%), Additive 0.15 mmol (1.0 equiv.), solvent 4.0 mL under blue LEDs (15 W, 462 nm), under N_2_ atmosphere, r.t. (23–25 °C), 12 h;

^b)^ Isolated yields are given;

^c)^ without light. TT═thianthrene PC═photocatalyst; DCM═dichloromethane; THF═tetrahydrofuran; DMF═*N,N*‐dimethylformide; DMSO═dimethyl sulfoxide; MeCN═acetonitrile; Py═pyridine; N.D.═Not detected. Structures of photocatalysts are given in Table S9 (Supporting Information).

**Scheme 1:**
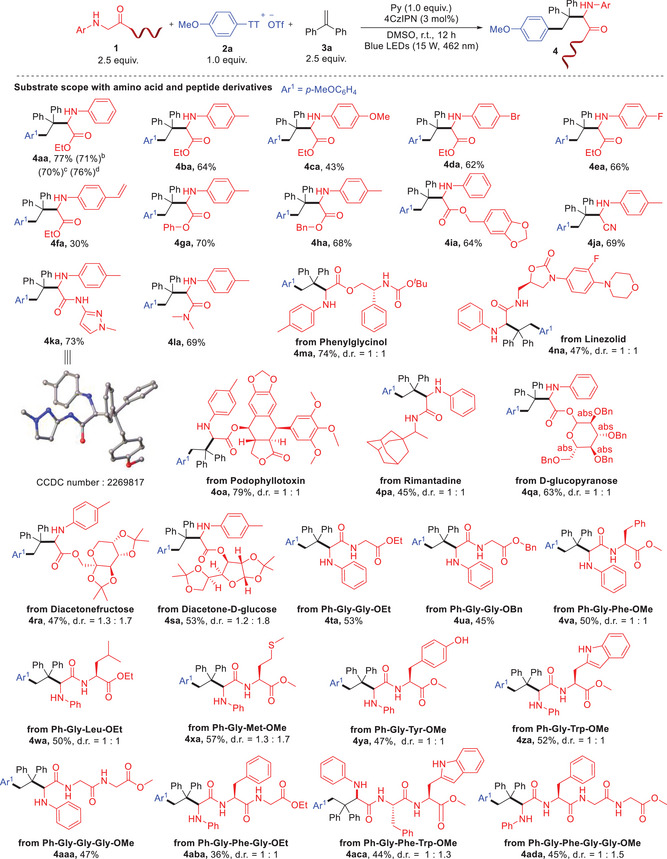
Substrate scope with amino acid derivatives. a) Standard conditions: **1** 0.375 mmol (2.5 equiv.), **2a** 0.15 mmol (1.0 equiv.), **3a** 0.375 mmol (2.5 equiv.), 4CzIPN 0.0045 mmol (3 mol%), Py 0.15 mmol (1.0 equiv.), MeCN 4.0 mL under blue LED irradiation, under N_2_ atmosphere, r.t. (23–25 °C), 12 h. b) Replace ^−^OTf with ^−^BF_4_. c) Replacing TT with dibenzo(b,d)thiophene. d) Replacing TT with phenoxathiin. Isolated yields are given. d.r. were determined by ^1^H NMR.

**Scheme 2:**
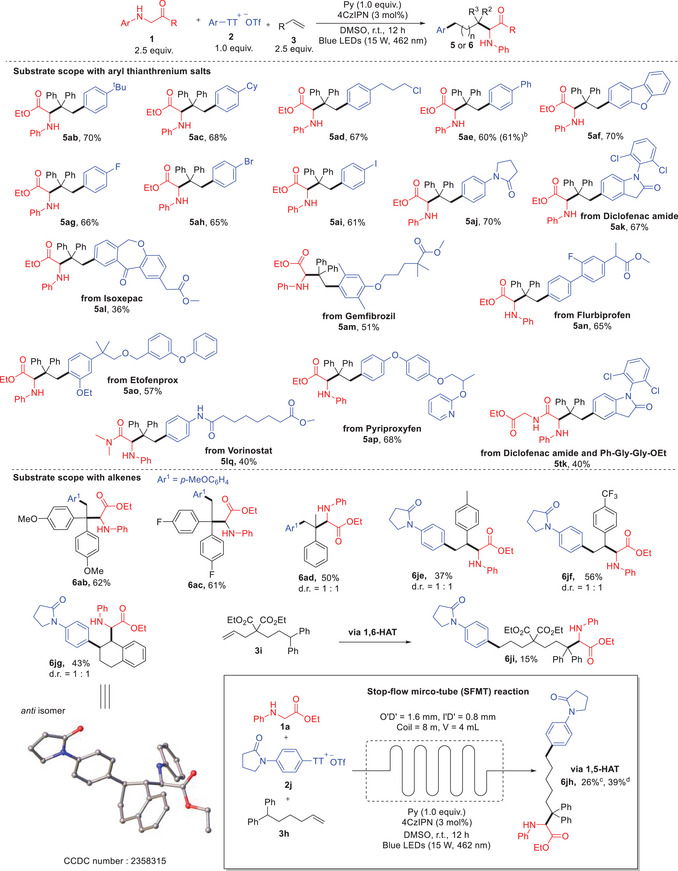
Substrates scope with aryl thianthrenium salts and alkenes. a) Standard conditions: **1** 0.375 mmol (2.5 equiv.), **2** 0.15 mmol (1.0 equiv.), **3** 0.375 mmol (2.5 equiv.), 4CzIPN 0.0045 mmol (3 mol%), Py 0.15 mmol (1.0 equiv.), MeCN 4.0 mL under blue LED irradiation, under N_2_ atmosphere, r.t. (23–25 °C), 12 h. b) Replace ^−^OTf with ^−^BF_4_. c) On bench reaction. d) Stop‐flow mirco‐tube (SFMT) reaction. Isolated yields are given. d.r. were determined by ^1^H NMR. HAT = Hydrogen atom transfer.

Under the optimized reaction conditions, we explored the scope of amino acid derivatives (**Scheme** **1**). Glycine esters bearing substituents with varied electronic effects (Me, OMe, Br, F, and Vinyl) on the aromatic rings provided moderate to good yields of coupling products (**4ba**‐**4fa**). Phenol and benzyl alcohol‐derived glycinates were also compatible, yielding products **4ga**‐**4ia**. α‐Amino nitrile and amide derivatives of glycine exhibited good compatibility, yielding products **4ja**‐**4la**, with the structure of **4ka** confirmed by X‐ray diffraction. Glycine derivatives derived from β‐amino alcohol (**4ma**), as well as drugs with antiviral (**4na**), anticancer properties (**4oa**), and antibacterial (**4pa**) were successfully synthesized. Glycoside‐derived glycinates proceeded smoothly under these conditions (**4qa**‐**4sa**). Furthermore, a series of dipeptides (**4ta**‐**4za**), tripeptides (**4aaa**‐**4aca**), and a tetrapeptide (**4ada**) derived from *N*‐aryl glycine were subjected to the standard reaction conditions (**Scheme** **2**), yielding the site‐selective coupling products in moderate yields. Notably, peptides containing sensitive functional groups such as thiomethyl (**4xa**), phenol (**4ya**), and indole (**4za**) proved to be excellent substrates for this transformation. Replacing triflate with fluoroborate as the counterion in **2a** delivered **4aa** in 71% yield. Analogues of **2a**, dibenzothiophenium salt, and phenoxathiinium salt derived from anisole were demonstrated with similar reactivities under these conditions. While using *p*‐iodoanisole and *p*‐bromoanisole as precursors of aryl radicals were unsuccessful to produce the corresponding product.

We expanded our investigation into the substrate scope of the three‐component radical coupling reaction, focusing on aryl thianthrenium salts (Scheme 2, top). The reaction exhibited tolerance toward diverse functional groups on arenes, including alkyl (**5ab** and **5ac**), chloroalkyl (**5ad**), aryl (**5ae**‐**5ai**), fluoride (**5ag**), bromide (**5ah**), iodide (**5ai**), and electron‐rich aniline derivatives (**5aj**). Notably, substrates synthesized via site‐selective C─H thianthrenation of drug molecules such as Diclofenac amide, Isoxepac, Gemfibrozil, Flurbiprofen, Etofenprox, Pyriproxyfen, and Vorinostat successfully yielded corresponding amino acid derivatives (**5ak**‐**5ap** and **5lq**). Particularly promising was the smooth transformation of Diclofenac amide thianthrenium salt (**2k**) with dipeptides, resulting in peptide‐drug conjugates **5tk**.

We also investigated the tolerance of various alkenes in the reaction (Scheme 2, bottom). Both electron‐rich (**6ab**) and electron‐deficient (**6ac**) 1,1‐diarylethylenes smoothly provided the coupling products. α‐Alkyl styrene was demonstrated good substrate for the transformation (**6ad**). Both electron‐deficient (**6jf**) and electron‐rich (**6je**) styrenes were compatible under these conditions. Additionally, cyclic styrene was able to facilitate the three‐component coupling reaction with reasonable yield (**6jg**).

Unfortunately, alkyl alkenes proved incompatible with this transformation. However, when remote arenes were present within the carbon chains, conjugations of aryl thianthrenium salts with amino acid derivatives were successfully achieved via either 1,5‐ or 1,6‐hydrogen atom transfer (**6jh** and **6ji**). Notably, conducting the synthesis of **6jh** using a stop‐flow micro‐tube (SFMT) reactor^[^ ^18^ ^]^ significantly enhanced efficiency. These findings underscore the flexibility of the linker and further highlight the potential of this protocol in synthesizing peptide‐drug conjugates.

**Scheme 3:**
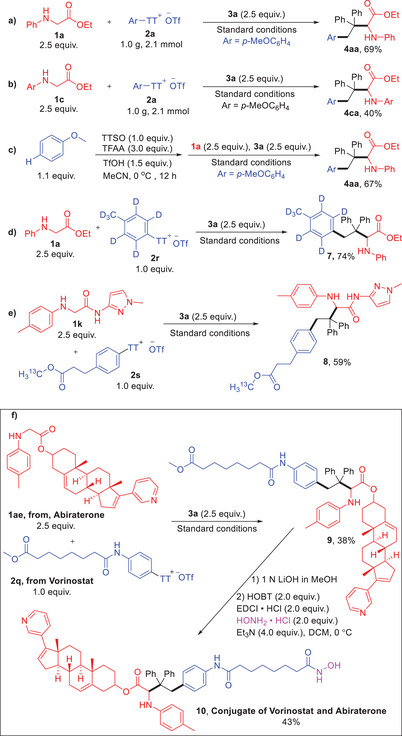
Synthetic applications. a,b) Gram‐scale reactions; c) One‐pot synthesis; d,e) Synthesis of deuterated and ^13^C labeling alkyl amino acid derivatives; f) Synthesis of conjugate (**10**) of Vorinostat and Abiraterone. EDCI · HCl = 1–ethyl–3–(3–dimethylaminopropyl)carbodiimide; HOBT = 1–hydroxybenzotriazole; Et_3_N = triethylamine; DCM = dichloromethane; THF = tetrahydrofuran.

To illustrate the practical application of this formal dual C─H functionalization, gram‐scale reactions were conducted using 2.1 mmol of **2a**, **1a,** and **1c** without modification of conditions, yielding the products in 69% and 40% isolated yields, respectively (**Scheme** **3**a,b). Integrating thianthrenation and photoredox‐catalyzed radical coupling into a one‐pot reaction provided product **4aa** in 67% isolated yield (Scheme 3c). It is well recognized that D‐ or ^13^C‐labeled^[^ ^19^ ^]^ bioactive compounds play a crucial role in material design and pharmaceutical science, especially in drug metabolism studies. Employing deuterated toluene (d^8^)‐derived aryl thianthrenium salt (**2r**) under these conditions yielded deuterated arylalkyl glycinate derivatives **7** in 74% isolated yield (Scheme 3d). ^13^C‐labeled aryl thianthrenium salt (**2s**) delivered the corresponding ^13^C‐labeled amino acid derivative **8** in 59% yield (Scheme 3e).

We utilized this formal dual C─H functionalization procedure to synthesize a conjugate (**10**, Scheme 3f) of Vorinostat,^[^ ^20^ ^]^ a histone deacetylase (HDAC) inhibitor for treating cutaneous T cell lymphoma (CTCL), and Abiraterone,^[^ ^21^ ^]^ prescribed alongside prednisone for treating metastatic prostate cancer. And preliminary investigation into the cytotoxicity of **10** against DU145 cancer cells was conducted. The compound exhibited moderate cytotoxicity with an IC50 value of 52.0 µM. These initial findings underscore the potential applications of this transformation in conjugating drug molecules with amino acid and peptide derivatives.

**Scheme 4:**
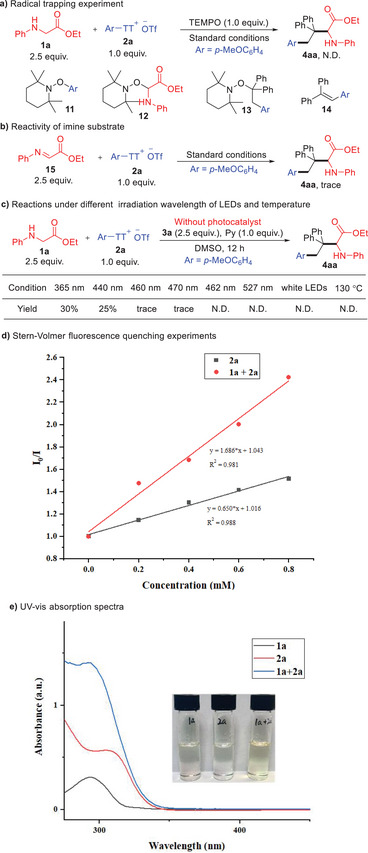
Control experiments and spectroscopic characterization.

**Scheme 5:**
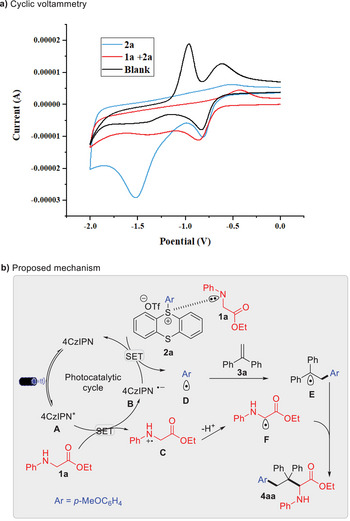
Electrochemical characterization and proposed mechanism.

To gain deeper mechanistic insights, a series of experiments were conducted. Addition of 2,2,6,6–tetramethyl–1–peperidyloxy (TEMPO, 1.0 equiv.) completely inhibited the transformation, and TEMPO‐adducts **11**, **12**, **13**, and by‐product **14** were identified by high‐resolution mass spectrometry (HRMS) (**Scheme** **4**a). Substituting **1a** with the imine analogues of *N*‐phenyl glycinate (**15**) resulted in no isolable product of **4aa** (Scheme 4b), indicating the importance of the glycine structure for product formation. It was found that under 365 nm irradiation the reaction was able to produce **4aa** in 30% yield without the presence of photocatalyst. Further experiments shown that the conversion could be observed under irradiation with 460 nm or shorter wavelength, while longer wavelength irradiation or heating failed to initiate the reaction (Scheme 4c). These results supported the formation of an EDA complex of **1a** and **2a**. Stern–Volmer fluorescence quenching experiments demonstrated significant fluorescence quenching of the excited state of 4CzIPN by mixing **1a** and **2a** (Scheme 4d). UV−Vis absorption spectra of individual components and their mixed solution in DMSO showed increased absorption upon mixing **1a** and **2a**, suggesting the formation of an electron donor‐acceptor (EDA) complex (Scheme 4e).^[^ ^22^ ^]^ Cyclic Voltammetry experiments revealed the reductive potential of thianthrenium salt **2a** (E_red_ = −1.5 V vs SCE in DMSO), indicating its unlikely reduction by the reductive photocatalyst (E [4CzIPN/4CzIPN^·^¯] = −1.24 V vs SCE in MeCN).^[^ ^31^ ^]^ However, mixing **1a** and **2a** shifted this potential to ‐1.18 V (vs SCE)^[^ ^23^ ^]^ (**Scheme** **5**a), aligning with conditions for photoredox catalytical reduction, consistent with the formation of an EDA complex observed in Stern‐Volmer plot and UV−Vis absorption experiments. Light on‐off experiments confirmed that continuous irradiation was crucial for product formation (Figure S23, Supporting Information).

Based on these findings, a plausible mechanism (Scheme 5b) is proposed. Upon light irradiation, the excited photocatalyst **A** interacts with **1a** to generate reductive photocatalyst specie 4CzIPN^·^¯ (**B**) and α‐amino radical **F** after deprotonation of intermediate **C**. An EDA complex forms between **1a** and **2a**, facilitating reduction of **2a** by intermediate **B** to form aryl radical **D**. Intermediate **D** adds to C = C bond of **3a**, generating benzyl radical **E**. Coupling between captodatively‐stabilized carbon radical **F** and active radical **E** ^[^ ^24^ ^]^ results in the conjugate of amino acid derivative and aryl thianthrenium salt.

## 3 Conclusion

In summary, we have developed a photoredox‐catalyzed three‐component arylative alkylation of amino acid and peptide derivatives using aryl thianthrenium salts and alkenes. Key to its success is the radical‐radical coupling enabled by captodatively‐stabilized carbon radicals of glycine derivatives. The method exhibits excellent tolerance toward diverse functional groups and demonstrates broad substrate scope with aryl thianthrenium salts, styrenes, and glycine derivatives. Additionally, alkenes participate in the conjugation with aryl thianthrenium and glycinate via either the 1,5‐ or 1,6‐hydrogen atom transfer process. This protocol provides a straightforward pathway for integrating pharmacologically relevant fragments and amino acids/peptides through formal dual C─H functionalization, highlighting its potential for peptide‐drug development.^[^ ^25^, ^26^, ^27^, ^28^, ^29^, ^30^, ^31^, ^32^, ^33^, ^34^, ^35^, ^36^, ^37^, ^38^, ^39^, ^40^ ^]^

## Conflict of Interest

The authors declare no conflict of interest.

## Supporting information



Supporting Information

## Data Availability

The data that support the findings of this study are available in the supplementary material of this article.
