# Supplementary material for: Stabilized Carbon Radical‐Mediated Assembly of Arylthianthrenium Salts, Alkenes and Amino Acid/Peptide Derivatives
Source: Adv Sci (Weinh). 2024 Nov 21;12(2):2411579. doi: 10.1002/advs.202411579 (PMC11727398; doi:10.1002/advs.202411579)
Supplement: Supplementary file 1 — Supporting Information [file ADVS-12-2411579-s001.pdf]

ADVANCED SCIENCE
Open Access

Supporting Information

for *Adv. Sci.*, DOI 10.1002/advs.202411579

Stabilized Carbon Radical-Mediated Assembly of Arylthianthrenium Salts, Alkenes and Amino Acid/Peptide Derivatives

Bo Dong, Weiguan Qi, Yifeng Chen, Yufei Zhang, Shiyu Gu, Jianlin Zhao, Qingfa Zhou, Jian Shen* and Lan-Gui Xie**

Supporting Information

Stabilized Carbon Radical-Mediated Assembly of Arylthianthrenium Salts, Alkenes and Amino Acid/Peptide Derivatives

Bo Dong,[1] Weiguan Qi,[1] Yifeng Chen,[1] Yufei Zhang,[3] Shiyu Gu,[1] Jianlin Zhao,[1] Qingfa Zhou,[3,*] Jian Shen[1,2,*] and Lan-Gui Xie[1,*]

[1] National and Local Joint Engineering Research Center of Biomedical Functional Materials, School of Chemistry and Materials Science, Nanjing Normal University, Nanjing 210023, China.

[2] Jiangsu Engineering Research Center of Interfacial Chemistry, Nanjing University, Nanjing 210023, China.

[3] State Key Laboratory of Natural Medicines, Department of Organic Chemistry, China Pharmaceutical University, Nanjing, 210009, P. R. China.

Email: zhouqingfa@cpu.edu.cn

jshen@njnu.edu.cn

xielg@njnu.edu.cn

Supporting Information

Contents

1 General Information.. 4

2 Experimental Procedures for Synthesis of Substrates 5

2.1 General procedure for the synthesis of *N*-aryl glycine derivatives..........5

2.1.1 Procedure A for the synthesis of *N*-aryl glycine derivatives 6

2.1.2 Procedure B for the synthesis of *N*-aryl glycine derivatives...............18

2.1.3 Procedure C for the synthesis of *N*-aryl glycine derivatives...............23

2.1.4 Preparation of ethyl 2-(phenylimino)acetate (15) [4]...........................26

2.2 General procedure for the synthesis of thianthrenium salt 27

2.2.1 Preparation of thianthrene *S*-oxide (TTSO) [5a]..................................28

2.2.2 Procedure D for the synthesis of aryl thianthrenium salt....................28

2.2.3 Procedure E for the synthesis of aryl thianthrenium salt 29

2.3 General procedure for the synthesis of alkenes 35

2.3.1 Procedure F for the synthesis of diphenyl olefins..............................35

3. Optimization of reaction conditions 37

Table S1. Screening the solvents. 37

Table S2. Screening the loading of aryl thianthrenium salt and *N*-phenylglycine ethyl ester. 38

Table S3. Screening the concentration of reaction solution. 38

Table S4. Screening loading of photocatalyst..39

Table S5. Screening the power of the blue light-emitting diode. 39

Table S6. Screening the reaction time. 40

Table S7. Screening the additive. 40

Table S8. Screening the wavelength of blue light-emitting diode...............41

Table S9. Screening the photocatalyst..42

Table S10. Electrochemical data for the employed photocatalysts. 43

4. General procedure for the stabilized alkyl radical-enabled conjugation of amino acid/peptide with aryl thianthrenium salt and characterization of products 44

4.1 Procedure G for the synthesis of conjugation of amino acid/peptide with aryl thianthrenium salt 44
4.2 Characterization of products 44
5 Studies on Synthetic Applications 86
5.1 Gram-scale synthesis 86
5.2 One-pot reaction 87
5.3 Preparation of D-labeled bioactive compounds (7) 88
5.4 Preparation of ^{13}C-labeled bioactive compounds (8) 89
5.5 Preparation of methyl 8-((4-(4-(((8*S*,9*R*,10*S*,13*R*,14*R*)-10,13-dimethyl-17-(pyridin-3-yl)-2,3,4,7,8,9,10,11,12,13,14,15-dodecahydro-1*H*-cyclopenta[*a*]phenanthren-3-yl)oxy)-4-oxo-2,2-diphenyl-3-(*p*-tolylamino)butyl)phenyl)amino)-8-oxooctanoate (9) 90
5.6 Preparation of (8*R*,9*S*,10*R*,13*S*,14*S*)-10,13-dimethyl-17-(pyridin-3-yl)-2,3,4,7,8,9,10,11,12,13,14,15-dodecahydro-1*H*-cyclopenta[a]phenanthren-3-yl-4-(4-(8-(hydroxyamino)-8-oxooctanamido)phenyl)-3,3-diphenyl-2-(*p*-tolylamino)butanoate (10) 92
5.7 Biological evaluation of 10 93
5.8 Set-up of SFMT reaction of 6jh 94
6 Mechanistic Studies 95
6.1 Trapping experiments 95
6.2 Controlled experiments 101
6.3 Stern-Volmer fluorescence quenching experiments. 104
6.4 UV-Vis absorption spectra 108
6.5 Light on-off experiments 109
6.6 Cyclic Voltammetry measurements 110
7 X-ray Crystallographic Data 111
7.1 Single-crystal X-ray diffraction measurement for 4ka 111
7.2 Single-crystal X-ray diffraction measurement for 6jg 114
8 References 116

9 NMR Spectra 119

1 General Information

All reactions involving air or moisture sensitive reagents were carried out in flame-dried glassware under argon atmosphere using standard Schlenk techniques. Solvents were either freshly distilled or obtained in extra-dry grade from commercial sources, and store over molecular sieve (3 Å). Dichloromethane (DCM) was refluxed over CaH_2 and used as freshly distilled. *N,N*-Dimethylformamide (DMF), *N,N*-Dimethylacetamide (DMA), Acetonitrile (MeCN), Tetrahydrofuran (THF), Dimethyl sulfoxide (DMSO) and 1,2-Dichloroethane (DCE) (extra dry over molecular sieves) was purchased commercially and used directly. Otherwise noted, commercially available chemicals were purchased from Energy Chemical and Titan Scientific Lab. The material of vessels for the reactions is borosilicate glass. Blue LED lamp (462 nm) was purchased from Zhongshan Langniu Lighting Technology Co., Ltd. (E27, 15 W). PhotoSyn-10 parallel photoreactor was purchased from Shanghai Quanhuan Technology Co., Ltd. Column chromatography was performed with silica gel (300−400 mesh). Merck silica gel 60 F254 plates were used for thin layer chromatography (TLC) with UV light (254/366 nm), Basic potassium permanganate and Phosphomolybdic acid as stains. The NMR spectra were recorded on a Bruker Avance 400 spectrometer at 400 MHz (^{1}H NMR), 101 MHz (^{13}C NMR) and 376 MHz (^{19}F NMR) in $CDCl_3$ or DMSO-d_6 with tetramethylsilane as the internal standard. Chemical shifts (δ) were reported in parts per million (ppm). Splitting patterns were designated as s, singlet; d, doublet; t, triplet; q, quartet; dd, doublet of doublets; m, multiplet. HR-ESI-MS was recorded on a Bruker MTQ III q-TOF instrument.

2 Experimental Procedures for Synthesis of Substrates

2.1 General procedure for the synthesis of *N*-aryl glycine derivatives

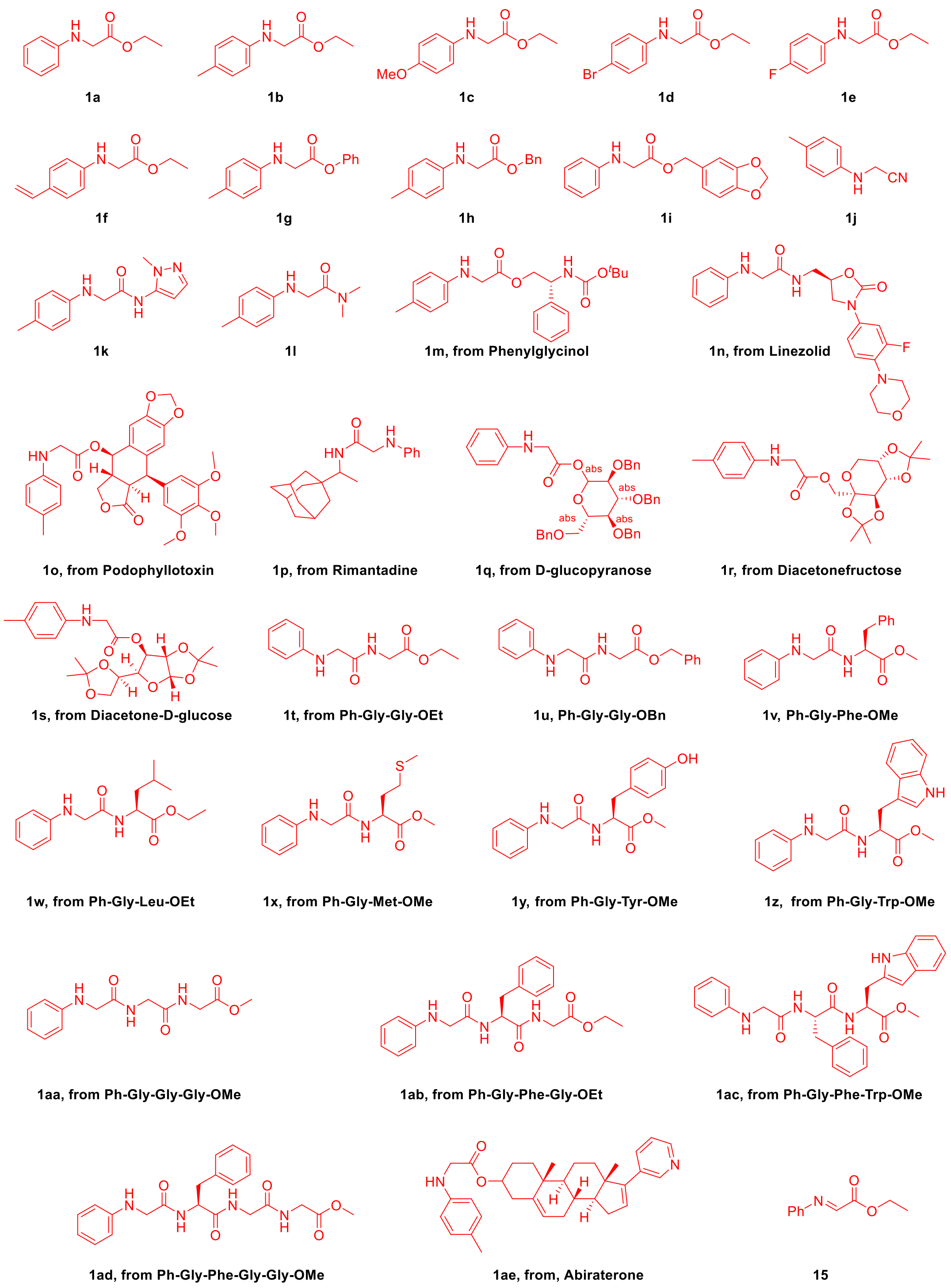

Figure S1. *N*-aryl glycine derivatives.

Substrates **1a** was purchased from commercial sources.

Substrates **1b, 1c, 1d, 1e, 1f, 1g, 1h, 1i, 1j, 1k, 1l, 1m, 1o, 1q, 1r, 1s,** and **1ae** were synthesized according to the general procedure A.

Substrates **1n, 1p, 1t, 1u, 1v, 1w, 1x, 1y, 1z,** and **1aa** were synthesized according to the general procedure B.

Substrates **1ab, 1ac** and **1ad** were synthesized according to the general procedure C.

Substrates **1b** [1], **1c** [1], **1d** [1], **1e** [1], **1f** [2], **1g** [1], **1h** [1], **1j** [1], **1l** [1] and **1y** [1] were prepared according to the literature procedure. Spectral data matched that reported in the literatures.

Substrates **1t** [3], **1z** [3] and **1aa** [3] were prepared according to the literature procedure. Spectral data matched that reported in the literatures.

Substrate **15** [4] was prepared according to the literature procedure. Spectral data matched that reported in the literatures.

Substrates **1i, 1k, 1m, 1n, 1o, 1p, 1q, 1r, 1s, 1u, 1v, 1w, 1x, 1aa, 1ac,1ad** and **1ae** were synthesized in the following procedures.

2.1.1 Procedure A for the synthesis of *N*-aryl glycine derivatives

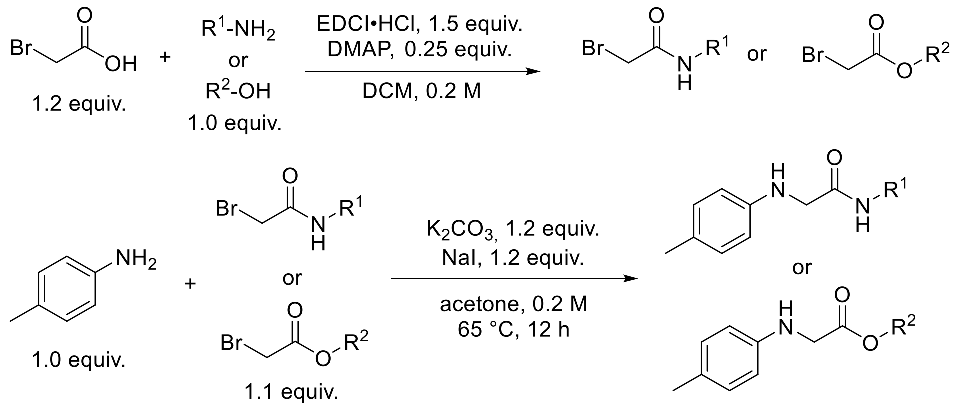

In a nitrogen atmosphere, bromoacetic acid (6.0 mmol, 1.2 equiv.), 4-dimethylaminopyridine (DMAP) (1.25 mmol, 0.25 equiv.), *N*-(3-dimethylaminopropyl)-*N'*-ethylcarbodiimide hydrochloride (EDCI·HCl) (7.5 mmol, 1.5 equiv.), amine or alcohol (5.0 mmol, 1.0 equiv.) and DCM (25 mL, 0.2 M.) were added to 100 mL schlenk tube. The reaction mixture is then cooled to 0 °C and stirred

at this temperature for 1 h, then stirred at room temperature (25 °C) for 12 h. After neutralized with saturated $NaHCO_3$ aqueous solution, and extraction with ethyl acetate (20 mL X 3), the combined organic layers were washed with brine, dried over anhydrous Na_2SO_4, filtrated and concentrated under reduced pressure. After purification by column chromatography on silica gel (ethyl acetate and petro ether as the elution), the title compound was obtained.

In a nitrogen atmosphere, amine (5.0 mmol, 1.0 equiv.), extra-dry acetone (25 mL, 0.2 M.), sodium iodide (6.0 mmol, 1.2 equiv.) and potassium carbonate (6.0mmol, 1.2 equiv.) were added to 100 mL schlenk tube. The reaction mixture stirred at temperature, followed by a slow drip of the corresponding bromoacetate-ester (5.5 mmol, 1.1 equiv.). The reaction mixture was stirred at 65 °C for 12 h, and extracted with ethyl acetate (20 mL X 3). The combined organic layer was dried on anhydrous Na_2SO_4 and condensed under reduced pressure. The residue was purified by flash chromatography (ethyl acetate and petro ether as the elution) on silica gel to afford *N*-aryl glycine derivatives.

Preparation of benzo[*d*][1,3]dioxol-5-ylmethyl phenylglycinate (1i)

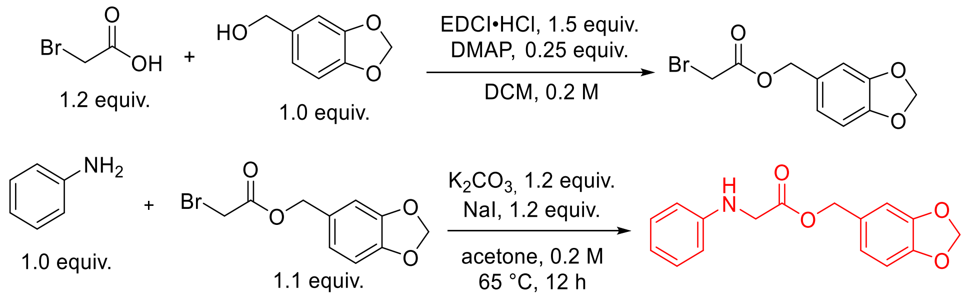

In a nitrogen atmosphere, bromoacetic acid (6.0 mmol, 1.2 equiv.), 4-dimethylaminopyridine (DMAP) (1.25 mmol, 0.25 equiv.), *N*-(3-dimethylaminopropyl)-*N'*-ethylcarbodiimide hydrochloride (EDCI·HCl) (7.5 mmol, 1.5 equiv.), piperonyl alcohol (5.0 mmol, 1.0 equiv.) and DCM (25 mL, 0.2 M.) were added to 100 mL schlenk tube. The reaction mixture is then cooled to 0 °C and stirred at this temperature for 1 h, then stirred at room temperature (25 °C) for 12 h. After neutralized with saturated $NaHCO_3$ aqueous solution, and extraction with ethyl acetate (20 mL X 3), the combined organic layers were washed with brine, dried over

anhydrous Na_2SO_4, filtrated and concentrated under reduced pressure. After purification by column chromatography on silica gel (ethyl acetate and petro ether as the elution), benzo[*d*][1,3]dioxol-5-ylmethyl 2-bromoacetate was obtained.

In a nitrogen atmosphere, aniline (5.0 mmol, 1.0 equiv.), extra-dry acetone (25 mL, 0.2 M.), sodium iodide (6.0 mmol, 1.2 equiv.) and potassium carbonate (6.0mmol, 1.2 equiv.) were added to 100 mL schlenk tube. The reaction mixture stirred at temperature, followed by a slow drip of benzo[*d*][1,3]dioxol-5-ylmethyl 2-bromoacetate (5.5 mmol, 1.1 equiv.) in DCM. The reaction mixture was stirred at 65 °C for 12 h, and extracted with ethyl acetate (20 mL X 3). The combined organic layer was dried on anhydrous Na_2SO_4 and condensed under reduced pressure. The residue was purified by flash chromatography (ethyl acetate and petro ether as the elution) on silica gel to afford *N*-aryl glycine derivatives **1i** as a colorless solid (570 mg, 40% yield). (petro ether / ethyl acetate = 3:1, Rf = 0.3, m.p. 74 °C).

^{1}H NMR (400 MHz, Chloroform-*d*) δ 7.22–7.18 (m, 2H), 6.85–6.74 (m, 4H), 6.62– 6.60 (m, 2H), 5.97 (s, 2H), 5.11 (s, 2H), 4.33– 4.26 (m, 1H), 3.94 (s, 2H) ppm.

^{13}C NMR (101 MHz, $CDCl_3$) δ 171.0, 147.8, 146.9, 129.3, 129.0, 122.5, 118.3, 113.0, 109.1, 108.3, 101.2, 67.0, 45.9 ppm.

HRMS (ESI) m/z: $[M+H]^+$ Calcd for $C_{16}H_{16}NO_4^+$ 286.1074; Found 286.1072.

Preparation of *N*-(1-methyl-1*H*-pyrazol-5-yl)-2-(*p*-tolylamino) acetamide (1k)

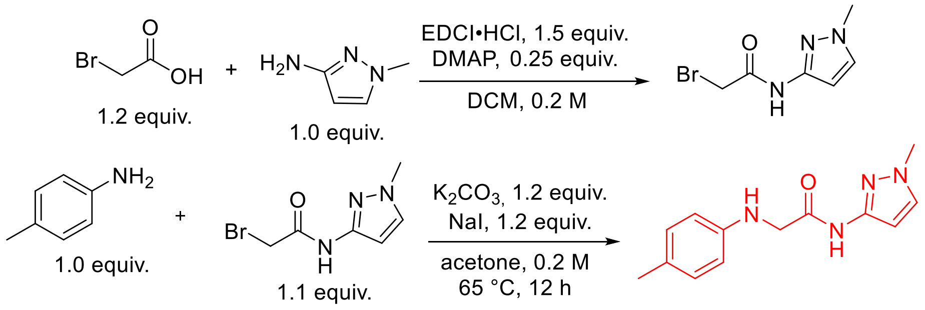

In a nitrogen atmosphere, bromoacetic acid (6.0 mmol, 1.2 equiv.), 4-dimethylaminopyridine (DMAP) (1.25 mmol, 0.25 equiv.), *N*-(3-dimethylaminopropyl)-*N'*-ethylcarbodiimide hydrochloride (EDCI·HCl) (7.5 mmol, 1.5 equiv.), 1-methyl-1*H*-pyrazol-5-ylamine (5.0 mmol, 1.0 equiv.) and DCM (25 mL,

0.2 M.) were added to 100 mL schlenk tube. The reaction mixture is then cooled to 0 °C and stirred at this temperature for 1 h, then stirred at room temperature (25 °C) for 12 h. After neutralized with saturated $NaHCO_3$ aqueous solution, and extraction with ethyl acetate (20 mL X 3), the combined organic layers were washed with brine, dried over anhydrous Na_2SO_4, filtrated and concentrated under reduced pressure. After purification by column chromatography on silica gel (ethyl acetate and petro ether as the elution), 2-bromo-*N*-(1-methyl-1*H*-pyrazol-5-yl) acetamide was obtained.

In a nitrogen atmosphere, *p*-toluidine (5.0 mmol, 1.0 equiv.), extra-dry acetone (25 mL, 0.2 M.), sodium iodide (6.0 mmol, 1.2 equiv.) and potassium carbonate (6.0mmol, 1.2 equiv.) were added to 100 mL schlenk tube. The reaction mixture stirred at temperature, followed by a slow drip of 2-bromo-*N*-(1-methyl-1*H*-pyrazol-5-yl) acetamide (5.5 mmol, 1.1 equiv.) in DCM. The reaction mixture was stirred at 65 °C for 12 h, and extracted with ethyl acetate (20 mL X 3). The combined organic layer was dried on anhydrous Na_2SO_4 and condensed under reduced pressure. The residue was purified by flash chromatography (dichloromethane and methanol as the elution) on silica gel to afford *N*-aryl glycine derivative **1k** as a yellowish solid (915 mg, 75% yield). (DCM / MeOH = 20:1, Rf = 0.3, m.p. 152 °C).

^{1}H NMR (400 MHz, $CDCl_3$) δ 9.05 (s, 1H), 7.23 (d, *J* = 2.3 Hz, 1H), 7.00 (d, *J* = 8.0 Hz, 2H), 6.72 (d, *J* = 2.3 Hz, 1H), 6.55 (d, *J* = 8.1 Hz, 2H), 4.27 (t, *J* = 5.6 Hz, 1H), 3.86 (d, *J* = 5.4 Hz, 2H), 3.75 (s, 3H), 2.24 (s, 3H) ppm.

^{13}C NMR (101 MHz, $CDCl_3$) δ 168.7, 146.3, 144.6, 130.8, 129.9, 128.7, 113.4, 97.2, 49.5, 38.7, 20.3 ppm.

HRMS (ESI) m/z: $[M+H]^+$ Calcd for $C_{13}H_{17}N_4O^+$ 245.1397; Found 245.1388.

Preparation of (*R*)-2-((tert-butoxycarbonyl)amino)-2-phenylethyl *p*-tolylglycinate (1m)

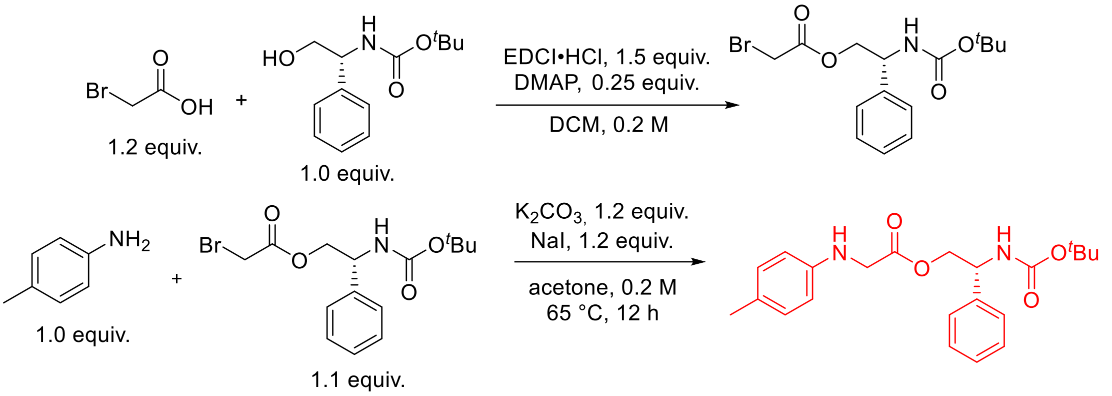

In a nitrogen atmosphere, bromoacetic acid (6.0 mmol, 1.2 equiv.), 4-dimethylaminopyridine (DMAP) (1.25 mmol, 0.25 equiv.), *N*-(3-Dimethylaminopropyl)-*N'*-ethylcarbodiimide hydrochloride (EDCI·HCl) (7.5 mmol, 1.5 equiv.), BOC-L-phenylglycinol (5.0 mmol, 1.0 equiv.) and DCM (25 mL, 0.2 M.) were added to 100 mL schlenk tube. The reaction mixture is then cooled to 0 °C and stirred at this temperature for 1 h, then stirred at room temperature (25 °C) for 12 h. After neutralized with saturated $NaHCO_3$ aqueous solution, and extraction with ethyl acetate (20 mL X 3), the combined organic layers were washed with brine, dried over anhydrous Na_2SO_4, filtrated and concentrated under reduced pressure. After purification by column chromatography on silica gel (ethyl acetate and petro ether as the elution), (*R*)-2-((*tert*-butoxycarbonyl) amino)-2-phenylethyl 2-bromoacetate was obtained.

In a nitrogen atmosphere, *p*-toluidine (5.0 mmol, 1.0 equiv.), extra-dry acetone (25 mL, 0.2 M.), sodium iodide (6.0 mmol, 1.2 equiv.) and potassium carbonate (6.0mmol, 1.2 equiv.) were added to 100 mL schlenk tube. The reaction mixture stirred at temperature, followed by a slow drip of (*R*)-2-((*tert*-butoxycarbonyl)amino)-2-phenylethyl 2-bromoacetate (5.5 mmol, 1.1 equiv.) in DCM. The reaction mixture was stirred at 65 °C for 12 h, and extracted with ethyl acetate (20 mL X 3). The combined organic layer was dried on anhydrous Na_2SO_4 and condensed under reduced pressure. The residue was purified by flash chromatography (ethyl acetate and petro ether as the elution) on silica gel to afford *N*-aryl glycine derivative **1m** as a yellowish solid (1.57 g, 82% yield). (petro ether / ethyl acetate = 5:1, Rf = 0.3, m.p. 109 °C).

^{1}H NMR (400 MHz, $CDCl_3$) δ 7.35–7.25 (m, 5H), 7.00 (d, *J* = 7.7 Hz, 2H), 6.49 (d, *J* = 7.6 Hz, 2H), 5.25 (s, 1H), 5.03 (s, 1H), 4.38 (s, 2H), 4.15 (s, 1H), 3.86 (s, 2H), 2.25 (s, 3H), 1.45 (s, 9H) ppm.

^{13}C NMR (101 MHz, $CDCl_3$) δ 171.2, 155.1, 144.5, 138.4, 129.7, 128.6, 127.7, 127.3, 126.4, 113.0, 79.7, 67.0, 53.3, 45.9, 28.2, 20.3 ppm.

HRMS (ESI) m/z: $[M+H]^+$ Calcd for $C_{22}H_{29}N_2O_4^+$ 385.2122; Found 385.2116.

Preparation of (5*R*,5a*R*,8a*R*,9*R*)-8-oxo-9-(3,4,5-trimethoxyphenyl)-5,5a,6,8,8a,9-hexahydrofuro[3',4':6,7]naphtho[2,3-*d*][1,3]dioxol-5-yl *p*-tolylglycinate (1o)

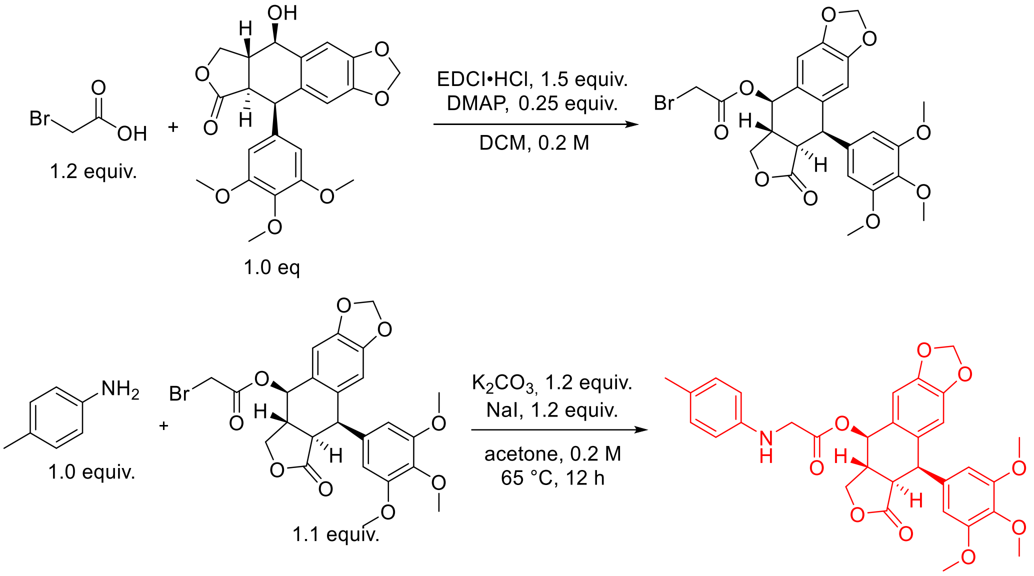

In a nitrogen atmosphere, bromoacetic acid (6.0 mmol, 1.2 equiv.), 4-dimethylaminopyridine (DMAP) (1.25 mmol, 0.25 equiv.), *N*-(3-dimethylaminopropyl)-*N'*-ethylcarbodiimide hydrochloride (EDCI·HCl) (7.5 mmol, 1.5 equiv.), Podophyllotoxin (5.0 mmol, 1.0 equiv.) and DCM (25 mL, 0.2 M.) were added to 100 mL schlenk tube. The reaction mixture is then cooled to 0 °C and stirred at this temperature for 1 h, then stirred at room temperature (25 °C) for 12 h. After neutralized with saturated $NaHCO_3$ aqueous solution, and extraction with Ethyl acetate (20 mL X 3), the combined organic layers were washed with brine, dried over anhydrous Na_2SO_4, filtrated and concentrated under reduced pressure. After purification by column chromatography on silica gel (ethyl acetate and petro ether as the elution), (8*R*,9*S*,10*R*,13*S*,14*S*)-10,13-dimethyl-17-(pyridin-3-yl)-

2,3,4,7,8,9,10,11,12,13,14,15-dodecahydro-1*H*-cyclopenta[*a*]phenanthren-3-yl 2-bromoacetate was obtained.

In a nitrogen atmosphere, *p*-toluidine (5.0 mmol, 1.0 equiv.), extra-dry acetone (25 mL, 0.2 M.), sodium iodide (6.0 mmol, 1.2 equiv.) and potassium carbonate (6.0mmol, 1.2 equiv.) were added to 100 mL schlenk tube. The reaction mixture stirred at temperature, followed by a slow drip of (8*R*,9*S*,10*R*,13*S*,14*S*)-10,13-dimethyl-17-(pyridin-3-yl)-2,3,4,7,8,9,10,11,12,13,14,15-dodecahydro-1*H*-cyclopenta[*a*]phenanthren-3-yl 2-bromoacetate (5.5 mmol, 1.1 equiv.) in DCM. The reaction mixture was stirred at 65 °C for 12 h, and extracted with ethyl acetate (20 mL X 3). The combined organic layer was dried on anhydrous Na_2SO_4 and condensed under reduced pressure. The residue was purified by flash chromatography (ethyl acetate and petro ether as the elution) on silica gel to afford *N*-aryl glycine derivative **1o** as a yellow solid (1.77 g, 63% yield). (petro ether / ethyl acetate = 3:1, Rf = 0.2, m.p. 113 °C).

^{1}H NMR (400 MHz, $CDCl_3$) δ 7.00 (d, *J* = 8.1 Hz, 2H), 6.66 (s, 1H), 6.55–6.52 (m, 3H), 6.37 (s, 2H), 5.98–5.96 (m, 2H), 5.92 (d, *J* = 8.8 Hz, 1H), 4.57 (d, *J* = 4.3 Hz, 1H), 4.28–4.24 (m, 1H), 4.17–4.08 (m, 2H), 4.04–3.96 (m, 2H), 3.81 (s, 3H), 3.75 (s, 6H), 2.90–2.75 (m, 2H), 2.23 (s, 3H) ppm.

^{13}C NMR (101 MHz, $CDCl_3$) δ 173.4, 172.0, 152.6, 148.1, 147.5, 144.3, 137.1, 134.7, 132.3, 129.9, 128.0, 127.7, 113.1, 109.7, 108.1, 106.8, 101.6, 74.4, 71.0, 60.7, 56.1, 46.3, 45.3, 43.6, 38.4, 20.3 ppm.

HRMS (ESI) m/z: $[M+H]^+$ Calcd for $C_{31}H_{32}NO_9^+$ 562.2072; Found 562.2070.

Preparation of (3*S*,4*R*,5*S*,6*S*)-3,4,5-tris(benzyloxy)-6-((benzyloxy)methyl)tetrahydro-2*H*-pyran-2-yl phenylglycinate (1q)

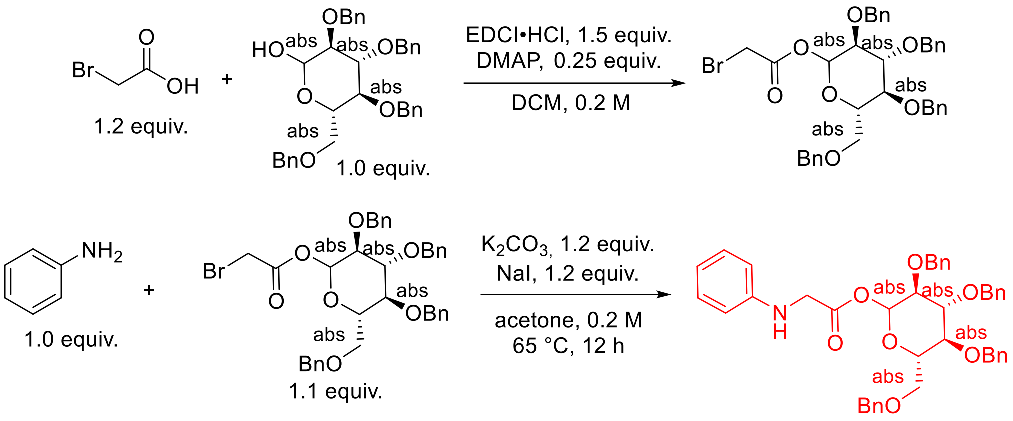

In a nitrogen atmosphere, bromoacetic acid (6.0 mmol, 1.2 equiv.), 4-dimethylaminopyridine (DMAP) (1.25 mmol, 0.25 equiv.), *N*-(3-dimethylaminopropyl)-*N*’-ethylcarbodiimide hydrochloride (EDCI·HCl) (7.5 mmol, 1.5 equiv.), 2,3,4,6-tetra-*O*-benzyl-D-glucopyranose (5.0 mmol, 1.0 equiv.) and DCM (25 mL, 0.2 M.) were added to 100 mL schlenk tube. The reaction mixture is then cooled to 0 °C and stirred at this temperature for 1 h, then stirred at room temperature (25 °C) for 12 h. After neutralized with saturated $NaHCO_3$ aqueous solution, and extraction with ethyl acetate (20 mL X 3), the combined organic layers were washed with brine, dried over anhydrous Na_2SO_4, filtrated and concentrated under reduced pressure. After purification by column chromatography on silica gel (ethyl acetate and petro ether as the elution), (3*S*,4*R*,5*S*,6*S*)-3,4,5-tris(benzyloxy)-6-((benzyloxy)methyl)tetrahydro-2*H*-pyran-2-yl 2-bromoacetate was obtained and easily purified by recrystallization from 2-propanol.

In a nitrogen atmosphere, aniline (5.0 mmol, 1.0 equiv.), extra-dry acetone (25 mL, 0.2 M.), sodium iodide (6.0 mmol, 1.2 equiv.) and potassium carbonate (6.0 mmol, 1.2 equiv.) were added to 100 mL schlenk tube. The reaction mixture stirred at temperature, followed by a slow drip of (3*S*,4*R*,5*S*,6*S*)-3,4,5-tris(benzyloxy)-6-((benzyloxy)methyl)tetrahydro-2*H*-pyran-2-yl 2-bromoacetate (5.5 mmol, 1.1 equiv.) in DCM. The reaction mixture was stirred at 65 °C for 12 h, and extracted with ethyl acetate (20 mL X 3). The combined organic layer was dried on anhydrous Na_2SO_4 and condensed under reduced pressure. The residue was purified by flash chromatography

(ethyl acetate and petro ether as the elution) on silica gel to afford *N*-aryl glycine derivative **1q** as a white solid (2.25 g, 67% yield) (petro ether / ethyl acetate = 10:1, Rf = 0.3, m.p. 86 °C) and easily purified by recrystallization from 2-propanol.

^{1}H NMR (400 MHz, $CDCl_3$) δ 7.37–7.31 (m, 18H), 7.20–7.16 (m, 4H), 6.77 (t, *J* = 7.4 Hz, 1H), 6.64 (d, *J* = 8.0 Hz, 2H), 6.45–6.43 (m, 1H), 4.96–4.93 (m, 1H), 4.87–4.81 (m, 2H), 4.73–4.64 (m, 2H), 4.63–4.59 (m, 1H), 4.53–4.47 (m, 2H), 4.08–3.96 (m, 2H), 3.93–3.89 (m, 1H), 3.79–3.70 (m, 4H), 3.62–3.59 (m, 1H) ppm.

^{13}C NMR (101 MHz, $CDCl_3$) δ 169.9, 146.8, 138.5, 138.0, 137.7, 137.4, 129.3, 128.5, 128.4, 128.1, 128.0, 127.9, 127.9, 127.8, 127.8, 127.7, 127.7, 118.4, 113.0, 91.0, 81.8, 78.7, 76.7, 75.7, 75.1, 73.5, 73.3, 73.0, 67.9, 45.8 ppm.

HRMS (ESI) m/z: $[M+H]^+$ Calcd for $C_{42}H_{44}NO_7^+$ 674.3112; Found 674.3108.

Preparation of ((3a*R*,5a*S*,8a*S*,8b*R*)-2,2,7,7-tetramethyltetrahydro-3a*H*-bis([1,3]dioxolo)[4,5-b:4',5'-d]pyran-3a-yl) methyl *p*-tolylglycinate (1r)

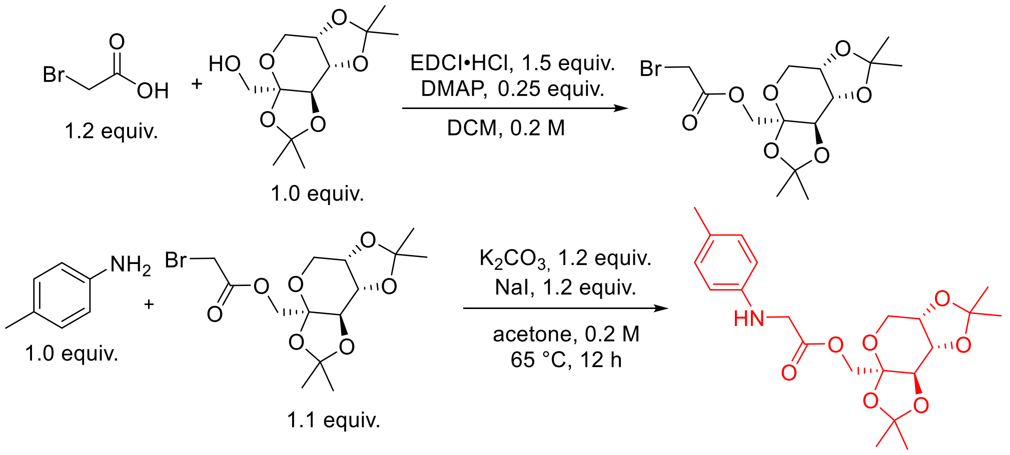

In a nitrogen atmosphere, bromoacetic acid (6.0 mmol, 1.2 equiv.), 4-dimethylaminopyridine (DMAP) (1.25 mmol, 0.25 equiv.), *N*-(3-dimethylaminopropyl)-*N*'-ethylcarbodiimide hydrochloride (EDCI·HCl) (7.5 mmol, 1.5 equiv.), diacetonefructose (5.0 mmol, 1.0 equiv.) and DCM (25 mL, 0.2 M.) were added to 100 mL schlenk tube. The reaction mixture is then cooled to 0 °C and stirred at this temperature for 1 h, then stirred at room temperature (25 °C) for 12 h. After neutralized with saturated $NaHCO_3$ aqueous solution, and extraction with ethyl acetate (20 mL X 3), the combined organic layers were washed with brine, dried over

anhydrous Na_2SO_4, filtrated and concentrated under reduced pressure. After purification by column chromatography on silica gel (ethyl acetate and petro ether as the elution), ((3a*R*,5a*S*,8a*S*,8b*R*)-2,2,7,7-tetramethyltetrahydro-3a*H*-bis([1,3]dioxolo)[4,5-b:4',5'-d]pyran-3a-yl)methyl 2-bromoacetate was obtained.

In a nitrogen atmosphere, *p*-toluidine (5.0 mmol, 1.0 equiv.), extra-dry acetone (25 mL, 0.2 M.), sodium iodide (6.0 mmol, 1.2 equiv.) and potassium carbonate (6.0 mmol, 1.2 equiv.) were added to 100 mL schlenk tube. The reaction mixture stirred at temperature, followed by a slow drip of ((3a*R*,5a*S*,8a*S*,8b*R*)-2,2,7,7-tetramethyltetrahydro-3a*H*-bis ([1,3] dioxolo) [4,5-b:4',5'-d] pyran-3a-yl) methyl 2-bromoacetate (5.5 mmol, 1.1 equiv.) in DCM. The reaction mixture was stirred at 65 °C for 12 h, and extracted with ethyl acetate (20 mL X 3). The combined organic layer was dried on anhydrous Na_2SO_4 and condensed under reduced pressure. The residue was purified by flash chromatography (ethyl acetate and petro ether as the elution) on silica gel to afford *N*-aryl glycine derivative **1r** as a yellow oil (1.28 g, 63% yield) (petro ether / ethyl acetate = 5:1, Rf = 0.3).

^1H NMR (400 MHz, $CDCl_3$) δ 6.99 (d, *J* = 8.1 Hz, 2H), 6.52 (d, *J* = 8.0 Hz, 2H), 4.62–4.59 (m, 1H), 4.53 (d, *J* = 11.6 Hz, 1H), 4.29 (d, *J* = 2.6 Hz, 1H), 4.24–4.21 (m, 2H), 4.15 (d, *J* = 11.6 Hz, 1H), 3.93–3.88 (m, 3H), 3.79–3.75 (m, 1H), 2.24 (s, 3H), 1.55 (s, 3H), 1.49 (s, 3H), 1.40 (s, 3H), 1.35 (s, 3H) ppm.

^{13}C NMR (101 MHz, $CDCl_3$) δ 170.5, 144.5, 129.5, 127.0, 112.9, 108.8, 108.5, 101.1, 70.4, 70.4, 69.8, 65.7, 61.0, 45.8, 26.2, 25.6, 24.9, 23.8, 20.1 ppm.

HRMS (ESI) m/z: $[M+H]^+$ Calcd for $C_{21}H_{30}NO_7^+$ 408.2017; Found 408.2014.

Preparation of (3a*S*,5*S*,6*R*,6a*S*)-5-((*R*)-2,2-dimethyl-1,3-dioxolan-4-yl)-2,2-dimethyltetrahydrofuro[2,3-d][1,3]dioxol-6-yl *p*-tolylglycinate (1s)

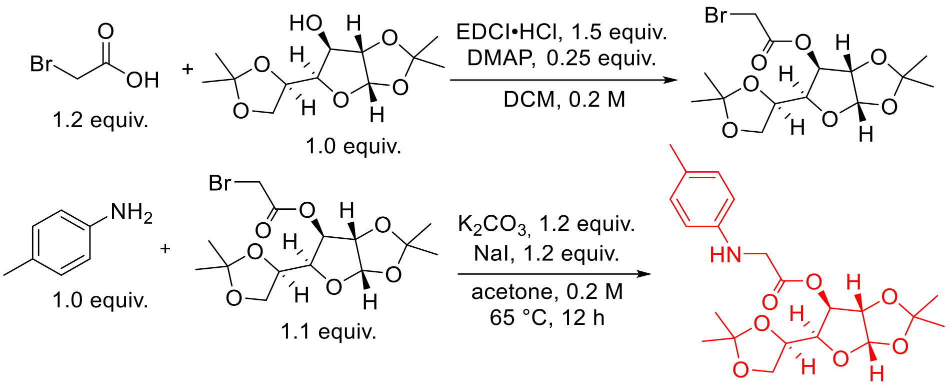

In a nitrogen atmosphere, bromoacetic acid (6.0 mmol, 1.2 equiv.), 4-dimethylaminopyridine (DMAP) (1.25 mmol, 0.25 equiv.), *N*-(3-dimethylaminopropyl)-*N*'-ethylcarbodiimide hydrochloride (EDCI·HCl) (7.5 mmol, 1.5 equiv.), diacetone-D-glucose (5.0 mmol, 1.0 equiv.) and DCM (25 mL, 0.2 M.) were added to 100 mL schlenk tube. The reaction mixture is then cooled to 0 °C and stirred at this temperature for 1 h, then stirred at room temperature (25 °C) for 12 h. After neutralized with saturated $NaHCO_3$ aqueous solution, and extraction with ethyl acetate (20 mL X 3), the combined organic layers were washed with brine, dried over anhydrous Na_2SO_4, filtrated and concentrated under reduced pressure. After purification by column chromatography on silica gel (ethyl acetate and petro ether as the elution), (3a*S*,5*S*,6*R*,6a*S*)-5-((*R*)-2,2-dimethyl-1,3-dioxolan-4-yl)-2,2-dimethyltetrahydrofuro[2,3-d][1,3]dioxol-6-yl 2-bromoacetate was obtained.

In a nitrogen atmosphere, *p*-toluidine (5.0 mmol, 1.0 equiv.), extra-dry acetone (25 mL, 0.2 M.), sodium iodide (6.0 mmol, 1.2 equiv.) and potassium carbonate (6.0 mmol, 1.2 equiv.) were added to 100 mL schlenk tube. The reaction mixture stirred at temperature, followed by a slow drip of (3a*S*,5*S*,6*R*,6a*S*)-5-((*R*)-2,2-dimethyl-1,3-dioxolan-4-yl)-2,2-dimethyltetrahydrofuro[2,3-d][1,3]dioxol-6-yl 2-bromoacetate (5.5 mmol, 1.1 equiv.) in DCM. The reaction mixture was stirred at 65 °C for 12 h, and extracted with ethyl acetate (20 mL X 3). The combined organic layer was dried on anhydrous Na_2SO_4 and condensed under reduced pressure. The residue was purified by flash chromatography (ethyl acetate and petro ether as the elution) on silica gel to afford

N-aryl glycine derivatives **1s** as a yellow oil (1.22 g, 60% yield) (petro ether / ethyl acetate = 10:1, Rf = 0.2).

^{1}H NMR (400 MHz, $CDCl_3$) δ 6.99 (d, *J* = 8.0 Hz, 2H), 6.52 (d, *J* = 8.0 Hz, 2H), 5.79 (d, *J* = 3.7 Hz, 1H), 5.35 (d, *J* = 2.8 Hz, 1H), 4.42 (d, *J* = 3.7 Hz, 1H), 4.22–4.14 (m, 3H), 4.08–3.99 (m, 2H), 3.92 (s, 2H), 2.23 (s, 3H), 1.51 (s, 3H), 1.40 (s, 3H), 1.30 (s, 3H), 1.28 (s, 3H) ppm.

^{13}C NMR (101 MHz, $CDCl_3$) δ 163.0, 137.5, 122.6, 120.4, 106.0, 105.1, 102.2, 97.8, 76.0, 72.6, 69.4, 65.1, 60.0, 39.0, 19.6, 19.5, 18.9, 18.0, 13.1 ppm.

HRMS (ESI) m/z: $[M+H]^+$ Calcd for $C_{21}H_{30}NO_7^+$ 408.2017; Found 408.2012.

Preparation of (8*R*,9*S*,10*R*,13*S*,14*S*)-10,13-dimethyl-17-(pyridin-3-yl)-2,3,4,7,8,9,10,11,12,13,14,15-dodecahydro-1*H*-cyclopenta[*a*]phenanthren-3-yl *p*-tolylglycinate (1ae)

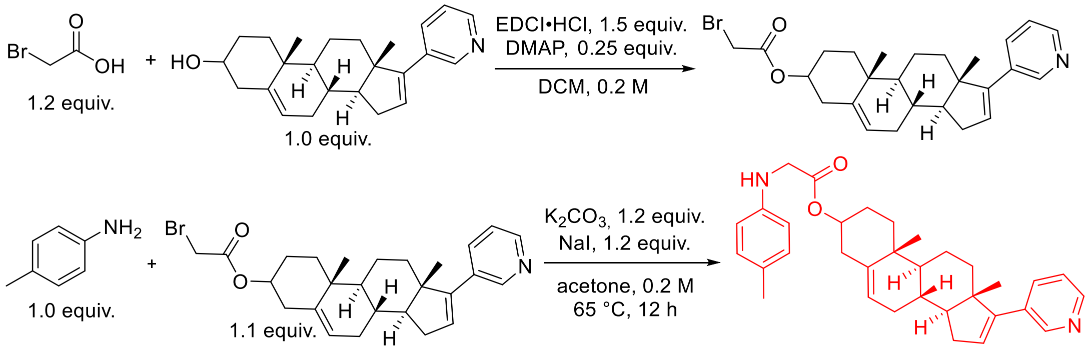

In a nitrogen atmosphere, bromoacetic acid (6.0 mmol, 1.2 equiv.), 4-dimethylaminopyridine (DMAP) (1.25 mmol, 0.25 equiv.), *N*-(3-dimethylaminopropyl)-*N*'-ethylcarbodiimide hydrochloride (EDCI·HCl) (7.5 mmol, 1.5 equiv.), abiraterone (5.0 mmol, 1.0 equiv.) and DCM (25 mL, 0.2 M.) were added to 100 mL schlenk tube. The reaction mixture is then cooled to 0 °C and stirred at this temperature for 1 h, then stirred at room temperature (25 °C) for 12 h. After neutralized with saturated $NaHCO_3$ aqueous solution, and extraction with ethyl acetate (20 mL X 3), the combined organic layers were washed with brine, dried over anhydrous Na_2SO_4, filtrated and concentrated under reduced pressure. After purification by column chromatography on silica gel (ethyl acetate and petro ether as the elution),

(5*R*,5a*R*,8a*R*,9*R*)-8-oxo-9-(3,4,5-trimethoxyphenyl)-5,5a,6,8,8a,9-hexahydrofuro[3',4':6,7]naphtho[2,3-d][1,3]dioxol-5-yl 2-bromoacetate was obtained.

In a nitrogen atmosphere, *p*-toluidine (5.0 mmol, 1.0 equiv.), extra-dry acetone (25 mL, 0.2 M.), sodium iodide (6.0 mmol, 1.2 equiv.) and potassium carbonate (6.0mmol, 1.2 equiv.) were added to 100 mL schlenk tube. The reaction mixture stirred at temperature, followed by a slow drip of (5*R*,5a*R*,8a*R*,9*R*)-8-oxo-9-(3,4,5-trimethoxyphenyl)-5,5a,6,8,8a,9-hexahydrofuro[3',4':6,7]naphtho[2,3-d][1,3]dioxol-5-yl 2-bromoacetate (5.5 mmol, 1.1 equiv.) in DCM. The reaction mixture was stirred at 65 °C for 12 h, and extracted with ethyl acetate (20 mL X 3). The combined organic layer was dried on anhydrous Na_2SO_4 and condensed under reduced pressure. The residue was purified by flash chromatography (ethyl acetate and petro ether as the elution) on silica gel to afford *N*-aryl glycine derivative **1ae** as a white solid (992 mg, 40% yield) (petro ether / ethyl acetate = 2:1, Rf = 0.2, m.p. 159 °C.).

^{1}H NMR (400 MHz, $CDCl_3$) δ 8.63–8.62 (m, 1H), 8.47–8.46 (m, 1H), 7.66–7.63 (m, 1H), 7.22–7.20 (m, 1H), 7.00 (d, *J* = 8.1 Hz, 2H), 6.55–6.53 (m, 2H), 6.00–5.99 (m, 1H), 5.43–5.42 (m, 1H), 4.76–4.68 (m, 1H), 4.17 (s, 1H), 3.86 (s, 2H), 2.39–2.37 (m, 2H), 2.31–2.24 (m, 4H), 2.10–2.04 (m, 3H), 1.92–1.86 (m, 2H), 1.79–1.57 (m, 6H), 1.52–1.45 (m, 1H), 1.21–1.09 (m, 5H), 1.05 (s, 3H) ppm.

^{13}C NMR (101 MHz, $CDCl_3$) δ 170.6, 151.6, 147.9, 147.8, 144.8, 139.7, 133.6, 132.9, 129.7, 129.2, 127.3, 123.0, 122.5, 113.1, 74.9, 57.4, 50.2, 47.3, 46.4, 38.0, 36.8, 36.7, 35.1, 31.7, 31.5, 30.3, 27.7, 20.8, 20.4, 19.2, 16.5 ppm.

HRMS (ESI) m/z: $[M+H]^+$ Calcd for $C_{33}H_{41}N_2O_2^+$ 497.3163; Found 497.3158.

2.1.2 Procedure B for the synthesis of *N*-aryl glycine derivatives

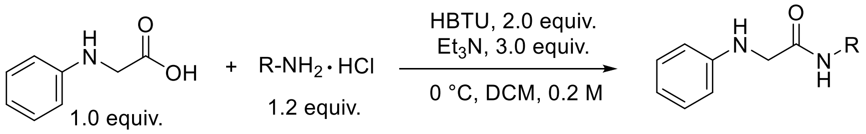

In a nitrogen atmosphere, *N*-phenylglycine (5.0 mmol, 1.0 equiv.), extra-dry DCM (25 mL, 0.2 M.), the amine ester hydrochloride (6.0 mmol, 1.2 equiv.) and Et_3N (15

mmol, 3.0 equiv.) were added to 100 mL schlenk tube. The reaction mixture stirred at 0 °C for 30 min, followed by added *O*-benzotriazole-*N*,*N*,*N'*,*N'*-tetramethyl-uronium-hexafluorophosphate (HBTU) (10 mmol, 2.0 equiv.). The reaction mixture was stirred at 0 °C for 12 h, and extracted with ethyl acetate (20 mL X 3). The combined organic layer was dried on anhydrous Na_2SO_4 and condensed under reduced pressure. The residue was purified by flash chromatography (ethyl acetate and petro ether as the elution) on silica gel to afford *N*-aryl glycine derivatives.

Preparation of (*S*)-*N*-((3-(3-fluoro-4-morpholinophenyl)-2-oxooxazolidin-5-yl)methyl)-2-(phenylamino)acetamide (1n)

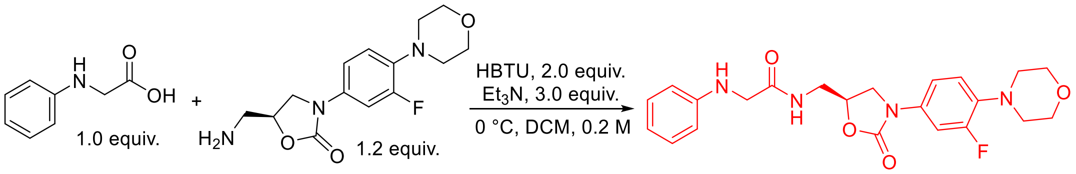

In a nitrogen atmosphere, *N*-phenylglycine (5.0 mmol, 1.0 equiv.), extra-dry DCM (25 mL, 0.2 M.), (*S*)-*N*-[[3-[3-fluoro-4-(4-morpholinyl)phenyl]-2-oxo-5-oxazolidinyl]methyl]amine (6.0 mmol, 1.2 equiv.) and Et_3N (15 mmol, 3.0 equiv.) were added to 100 mL schlenk tube. The reaction mixture stirred at 0 °C for 30 min, followed by added *O*-benzotriazole-*N*,*N*,*N'*,*N'*-tetramethyl-uronium-hexafluorophosphate (HBTU) (10 mmol, 2.0 equiv.). The reaction mixture was stirred at 0 °C for 12 h, and extracted with ethyl acetate (20 mL X 3). The combined organic layer was dried on anhydrous Na_2SO_4 and condensed under reduced pressure. The residue was purified by flash chromatography (ethyl acetate and petro ether as the elution) on silica gel to afford *N*-aryl glycine derivative **1n** as a white solid (1.86 g, 87% yield) (petro ether / ethyl acetate = 2:1, Rf = 0.5, m.p. 143 °C).

^{1}H NMR (400 MHz, $CDCl_3$) δ 7.42–7.38 (m, 1H), 7.25–7.22 (m, 1H), 7.13 (t, *J* = 7.7 Hz, 2H), 7.06–7.03 (m, 1H), 6.91 (t, *J* = 9.1 Hz, 1H), 6.77 (t, *J* = 7.3 Hz, 1H), 6.56 (d, *J* = 8.0 Hz, 2H), 4.77–4.71 (m, 1H), 4.25 (t, *J* = 5.7 Hz, 1H), 3.98 (t, *J* = 9.0 Hz, 1H), 3.88–3.86 (m, 4H), 3.82–3.80 (m, 2H), 3.73–3.66 (m, 3H), 3.07–3.04 (m, 4H) ppm.

^{13}C NMR (101 MHz, $CDCl_3$) δ 171.9, 155.2 (d, $J_{C\text{-}F}$ = 246.4 Hz), 154.1, 146.9, 136.3 (d, $J_{C\text{-}F}$ = 9.0 Hz), 132.7 (d, $J_{C\text{-}F}$ = 10.5 Hz), 129.2, 118.7, 118.6 (d, $J_{C\text{-}F}$ = 4.1 Hz), 113.7 (d, $J_{C\text{-}F}$ = 3.3 Hz), 112.8, 107.3 (d, $J_{C\text{-}F}$ = 26.4 Hz), 77.3, 66.7, 50.8, 50.8, 48.2, 47.4, 41.4 ppm.

^{19}F NMR (376 MHz, $CDCl_3$) δ -120.1.

HRMS (ESI) m/z: $[M+H]^+$ Calcd for $C_{22}H_{26}FN_4O_4^+$ 429.1933; Found 429.1925.

Preparation of *N*-(1-(adamantan-1-yl)ethyl)-2-(phenylamino)acetamide (1p)

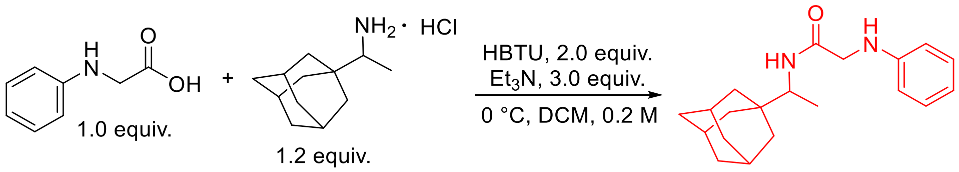

In a nitrogen atmosphere, *N*-phenylglycine (5.0 mmol, 1.0 equiv.), extra-dry DCM (25 mL, 0.2 M.), rimantadine hydrochloride (6.0 mmol, 1.2 equiv.) and Et_3N (15 mmol, 3.0 equiv.) were added to 100 mL schlenk tube. The reaction mixture stirred at 0 °C for 30 min, followed by added *O*-benzotriazole-*N*,*N*,*N'*,*N'*-tetramethyl-uronium-hexafluorophosphate (HBTU) (10 mmol, 2.0 equiv.). The reaction mixture was stirred at 0 °C for 12 h, and extracted with ethyl acetate (20 mL X 3). The combined organic layer was dried on anhydrous Na_2SO_4 and condensed under reduced pressure. The residue was purified by flash chromatography (ethyl acetate and petro ether as the elution) on silica gel to afford *N*-aryl glycine derivative **1p** as a white solid (1.36 g, 89% yield) (petro ether / ethyl acetate = 2:1, Rf = 0.3, m.p. 140 °C).

^{1}H NMR (400 MHz, $CDCl_3$) δ 7.23–7.19 (m, 2H), 6.83–6.79 (m, 1H), 6.67–6.59 (m, 3H), 4.37–4.24 (m, 1H), 3.81–3.79 (m, 2H), 3.76–3.68 (m, 1H), 1.92–1.89 (m, 3H), 1.66–1.62 (m, 3H), 1.54–1.43 (m, 6H), 1.38–1.33 (m, 3H), 0.97 (d, *J* = 6.9 Hz, 3H) ppm.

^{13}C NMR (101 MHz, $CDCl_3$) δ 169.5, 147.0, 129.4, 119.2, 113.4, 52.7, 49.0, 38.2, 36.9, 35.8, 28.2, 14.6 ppm.

HRMS (ESI) m/z: $[M+H]^+$ Calcd for $C_{20}H_{29}N_2O^+$ 313.2274; Found 313.2267.

Preparation of benzyl phenylglycylglycinate (1u)

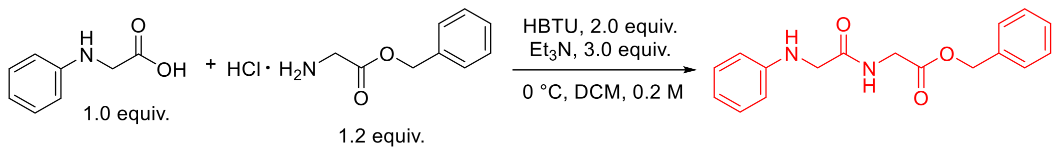

In a nitrogen atmosphere, *N*-phenylglycine (5.0 mmol, 1.0 equiv.), extra-dry DCM (25 mL, 0.2 M.), benzyl glycinate hydrochloride (6.0 mmol, 1.2 equiv.) and Et_3N (15 mmol, 3.0 equiv.) were added to 100 mL schlenk tube. The reaction mixture stirred at 0 °C for 30 min, followed by added *O*-benzotriazole-*N*,*N*,*N'*,*N'*-tetramethyl-uronium-hexafluorophosphate (HBTU) (10 mmol, 2.0 equiv.). The reaction mixture was stirred at 0 °C for 12 h, and extracted with ethyl acetate (20 mL X 3). The combined organic layer was dried on anhydrous Na_2SO_4 and condensed under reduced pressure. The residue was purified by flash chromatography (ethyl acetate and petro ether as the elution) on silica gel to afford *N*-aryl glycine derivative **1u** as a white solid (1.30 g, 87% yield) (petro ether / ethyl acetate = 2:1, Rf = 0.5, m.p. 99 °C).

H NMR (400 MHz, $CDCl_3$) δ 7.37–7.31 (m, 5H), 7.21–7.18 (m, 3H), 6.81 (t, *J* = 7.3 Hz, 1H), 6.63 (d, *J* = 8.0 Hz, 2H), 5.15 (s, 2H), 4.09 (d, *J* = 5.7 Hz, 2H), 3.84 (s, 2H) ppm.

^{13}C NMR (101 MHz, $CDCl_3$) δ 171.1, 169.5, 147.0, 135.1, 129.4, 128.6, 128.5, 128.3, 119.2, 113.3, 67.2, 48.7, 41.0 ppm.

HRMS (ESI) m/z: $[M+H]^+$ Calcd for $C_{17}H_{19}N_2O_3^+$ 299.1390; Found 299.1388.

Preparation of ethyl phenylglycyl-D-leucinate (1w)

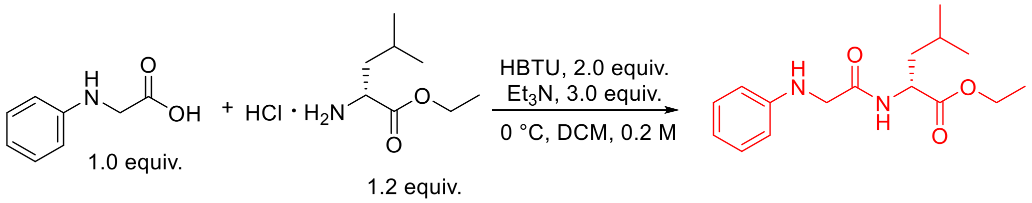

In a nitrogen atmosphere, *N*-phenylglycine (5.0 mmol, 1.0 equiv.), extra-dry DCM (25 mL, 0.2 M.), ethyl L-leucinate hydrochloride (6.0 mmol, 1.2 equiv.) and Et_3N (15 mmol, 3.0 equiv.) were added to 100 mL schlenk tube. The reaction mixture stirred at 0 °C for 30 min, followed by added *O*-benzotriazole-*N*,*N*,*N'*,*N'*-tetramethyl-uronium-hexafluorophosphate (HBTU) (10 mmol, 2.0 equiv.). The reaction mixture was stirred at 0 °C for 12 h, and extracted with ethyl acetate (20 mL X 3). The combined organic

layer was dried on anhydrous Na_2SO_4 and condensed under reduced pressure. The residue was purified by flash chromatography (ethyl acetate and petro ether as the elution) on silica gel to afford *N*-aryl glycine derivative **1w** as a colorless oil (1.26 g, 86% yield) (petro ether / ethyl acetate = 2:1, Rf = 0.5).

^{1}H NMR (400 MHz, $CDCl_3$) δ 7.19 (t, *J* = 7.7 Hz, 2H), 7.06 (d, *J* = 8.7 Hz, 1H), 6.78 (t, *J* = 7.3 Hz, 1H), 6.61 (d, *J* = 8.0 Hz, 2H), 4.67–4.61 (m, 1H), 4.42 (s, 1H), 4.13 (q, *J* = 7.1 Hz, 2H), 3.84–3.75 (m, 2H), 1.63–1.44 (m, 3H), 1.22 (t, *J* = 7.2 Hz, 3H), 0.89 (d, *J* = 6.0 Hz, 3H), 0.85 (d, *J* = 6.1 Hz, 3H) ppm.

^{13}C NMR (101 MHz, $CDCl_3$) δ 172.6, 170.5, 147.1, 129.2, 118.9, 113.2, 61.2, 50.4, 48.7, 41.2, 24.7, 22.7, 21.6, 14.0 ppm.

HRMS (ESI) m/z: $[M+H]^+$ Calcd for $C_{16}H_{25}N_2O_3^+$ 293.1860; Found 293.1855.

Preparation of methyl phenylglycyl-L-methioninate (1x)

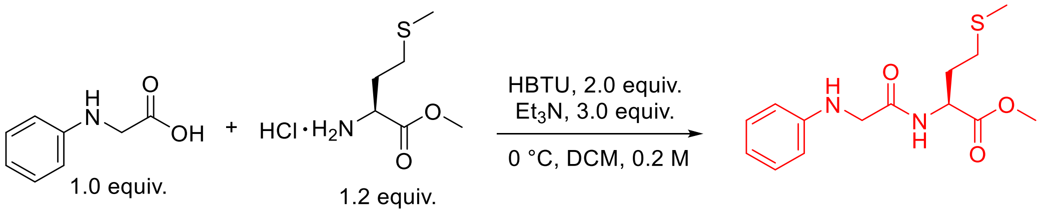

In a nitrogen atmosphere, *N*-phenylglycine (5.0 mmol, 1.0 equiv.), extra-dry DCM (25 mL, 0.2 M.), L-methionine methyl ester hydrochloride (6.0 mmol, 1.2 equiv.) and Et_3N (15 mmol, 3.0 equiv.) were added to 100 mL schlenk tube. The reaction mixture stirred at 0 °C for 30 min, followed by added *O*-benzotriazole-*N,N,N',N'*-tetramethyl-uronium-hexafluorophosphate (HBTU) (10 mmol, 2.0 equiv.). The reaction mixture was stirred at 0 °C for 12 h, and extracted with ethyl acetate (20 mL X 3). The combined organic layer was dried on anhydrous Na_2SO_4 and condensed under reduced pressure. The residue was purified by flash chromatography (ethyl acetate and petro ether as the elution) on silica gel to afford *N*-aryl glycine derivative **1w** as a yellow oil (1.24 g, 84% yield) (petro ether / ethyl acetate = 2:1, Rf = 0.5).

^{1}H NMR (400 MHz, $CDCl_3$) δ 7.32 (d, *J* = 8.5 Hz, 1H), 7.22–7.18 (m, 2H), 6.80 (t, *J* = 7.4 Hz, 1H), 6.63–6.61 (m, 2H), 4.78–4.73 (m, 1H), 4.38 (s, 1H), 3.86–3.77 (m, 2H), 3.70 (s, 3H), 2.38 (t, *J* = 7.4 Hz, 2H), 2.17–2.08 (m, 1H), 1.97–1.90 (m, 4H) ppm.

^{13}C NMR (101 MHz, $CDCl_3$) δ 172.0, 170.6, 146.9, 129.3, 119.1, 113.2, 52.4, 51.0, 48.6, 31.3, 29.8, 15.3 ppm.

HRMS (ESI) m/z: $[M+H]^+$ Calcd for $C_{14}H_{21}N_2O_3S^+$ 297.1267; Found 297.1264.

Preparation of methyl phenylglycylglycylglycinate (1aa)

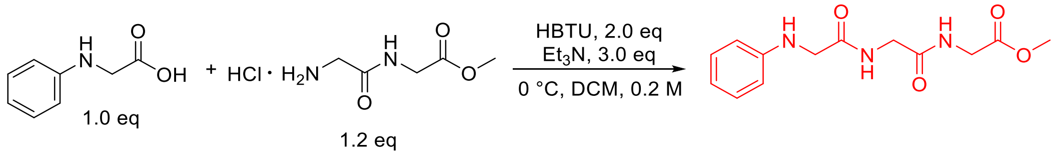

In a nitrogen atmosphere, *N*-phenylglycine (5.0 mmol, 1.0 equiv.), extra-dry DCM (25 mL, 0.2 M.), 2-(2-aminoacetylamino)acetic acid methyl·hydrochloride (6.0 mmol, 1.2 equiv.) and Et_3N (15 mmol, 3.0 equiv.) were added to 100 mL schlenk tube. The reaction mixture stirred at 0 °C for 30 min, followed by added *O*-benzotriazole-*N*,*N*,*N'*,*N'*-tetramethyl-uronium-hexafluorophosphate (HBTU) (10 mmol, 2.0 equiv.). The reaction mixture was stirred at 0 °C for 12 h, and extracted with ethyl acetate (20 mL X 3). The combined organic layer was dried on anhydrous Na_2SO_4 and condensed under reduced pressure. The residue was purified by flash chromatography (ethyl acetate and petro ether as the elution) on silica gel to afford *N*-aryl glycine derivative **1aa** as a white solid (1.23 g, 88% yield) (petro ether / ethyl acetate = 1:1, Rf = 0.2, m.p. 120 °C).

^{1}H NMR (400 MHz, $CDCl_3$) δ 7.43–7.39 (m, 1H), 7.22–7.19 (m, 2H), 6.82–6.78 (m, 1H), 6.74–6.61 (m, 1H), 6.62 (d, J = 7.9 Hz, 3H), 4.06–3.98 (m, 4H), 3.85 (s, 2H), 3.73 (s, 3H) ppm.

^{13}C NMR (101 MHz, $CDCl_3$) δ 171.7, 170.1, 169.0, 146.9, 129.5, 119.2, 113.2, 52.4, 48.5, 42.8, 41.1 ppm.

HRMS (ESI) m/z: $[M+H]^+$ Calcd for $C_{13}H_{18}N_3O_4^+$ 280.1292; Found 280.1285.

2.1.3 Procedure C for the synthesis of *N*-aryl glycine derivatives

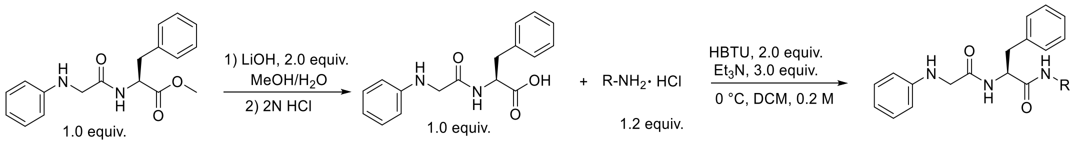

The compound **1ao** (5.00 mmol, 1.0 equiv.) was added to a flame-dried round-bottom flask in an ice-water bath, followed by the addition of MeOH (20 mL, 0.25 M). Finally, slowly add LiOH (10.0 mmol, 2.0 equiv. dissolved in 5 mL water) to the mixture, and the reaction was allowed to stirred at 0 °C until complete conversion is detected by TLC, remove methanol under reduced pressure and adjust the pH to 2 with 2 N HCl, diluted with water (30 mL) and extracted with ethyl acetate (20 mL X 3). The combined organic extracts were washed with brine (2 X 30 mL), dried over Na_2SO_4 and concentrated under reduced pressure to afford phenylglycyl-L-phenylalanine.

In a nitrogen atmosphere, phenylglycyl-L-phenylalanine (5.0 mmol, 1.0 equiv.), extra-dry DCM (25 mL, 0.2 M.), the amine ester hydrochloride (6.0 mmol, 1.2 equiv.) and Et_3N (15 mmol, 3.0 equiv.) were added to 100 mL schlenk tube. The reaction mixture stirred at 0 °C for 30 min, followed by added *O*-benzotriazole-*N*,*N*,*N'*,*N'*-tetramethyl-uronium-hexafluorophosphate (HBTU) (10 mmol, 2.0 equiv.). The reaction mixture was stirred at 0 °C for 12 h, and extracted with ethyl acetate (20 mL X 3). The combined organic layer was dried on anhydrous Na_2SO_4 and condensed under reduced pressure. The residue was purified by flash chromatography (ethyl acetate and methanol as the elution) on silica gel to afford *N*-aryl glycine derivatives.

Preparation of methyl phenylglycyl-L-phenylalanyl-L-tryptophanate (1ac)

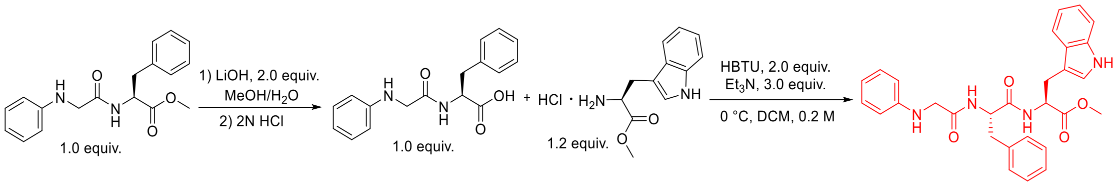

The compound **1v** (5.00 mmol, 1.0 equiv.) was added to a flame-dried round-bottom flask in an ice-water bath, followed by the addition of MeOH (20 mL, 0.25 M). Finally, slowly add LiOH (10.0 mmol, 2.0 equiv. dissolved in 5 mL water) to the mixture, and the reaction was allowed to stirred at 0 °C until complete conversion is detected by TLC, remove methanol under reduced pressure and adjust the pH to 2 with 2 N HCl, diluted with water (30 mL) and extracted with ethyl acetate (20 mL X 3). The

combined organic extracts were washed with brine (2 X 30 mL), dried over Na_2SO_4 and concentrated under reduced pressure to afford phenylglycyl-L-phenylalanine.

In a nitrogen atmosphere, phenylglycyl-L-phenylalanine (5.0 mmol, 1.0 equiv.), extra-dry DCM (25 mL, 0.2 M.), methyl L-tryptophanate hydrochloride (6.0 mmol, 1.2 equiv.) and Et_3N (15 mmol, 3.0 equiv.) were added to 100 mL schlenk tube. The reaction mixture stirred at 0 °C for 30 min, followed by added *O*-benzotriazole-*N*,*N*,*N'*,*N'*-tetramethyl-uronium-hexafluorophosphate (HBTU) (10 mmol, 2.0 equiv.). The reaction mixture was stirred at 0 °C for 12 h, and extracted with ethyl acetate (20 mL X 3). The combined organic layer was dried on anhydrous Na_2SO_4 and condensed under reduced pressure. The residue was purified by flash chromatography on silica gel to afford *N*-aryl glycine derivative **1ac** as a white solid (2.04 g, 82% yield) (ethyl acetate / methanol = 50:1, Rf = 0.4, m.p. 110 °C).

^{1}H NMR (400 MHz, $CDCl_3$) δ 8.63 (s, 1H), 7.35–7.30 (m, 2H), 7.20–7.04 (m, 9H), 6.99–6.96 (m, 2H), 6.82–6.78 (m, 2H), 6.41–6.39 (m, 2H), 5.01–4.95 (m, 1H), 4.92–4.84 (m, 1H), 3.68 (s, 3H), 3.40–3.36 (m, 1H), 3.29–3.20 (m, 2H), 3.19–3.14 (, 1H), 3.03–2.98 (m, 1H), 2.90–2.85 (m, 1H) ppm.

^{13}C NMR (101 MHz, $CDCl_3$) δ 171.8, 171.0, 170.9, 146.8, 136.0, 135.9, 129.4, 129.3, 128.5, 127.4, 126.8, 123.3, 121.9, 119.5, 118.9, 118.4, 113.0, 111.3, 109.4, 53.2, 52.9, 52.4, 48.2, 37.9, 27.3 ppm.

HRMS (ESI) m/z: $[M+H]^+$ Calcd for $C_{29}H_{31}N_4O_4^+$ 499.2340; Found 499.2337.

Preparation of methyl phenylglycyl-L-phenylalanylglycylglycinate (1ad)

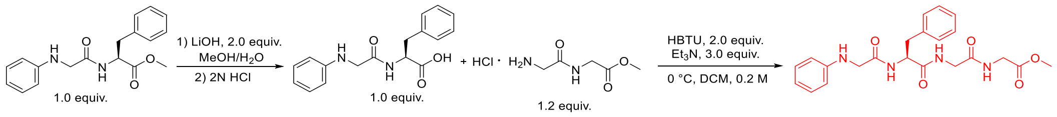

The compound **1v** (5.00 mmol, 1.0 equiv.) was added to a flame-dried round-bottom flask in an ice-water bath, followed by the addition of MeOH (20 mL, 0.25 M). Finally, slowly add LiOH (10.0 mmol, 2.0 equiv. dissolved in 5 mL water) to the mixture, and the reaction was allowed to stirred at 0 °C until complete conversion is detected by TLC, remove methanol under reduced pressure and adjust the pH to 2 with 2 N HCl, diluted with water (30 mL) and extracted with ethyl acetate (20 mL X 3). The

combined organic extracts were washed with brine (2 X 30 mL), dried over Na_2SO_4 and concentrated under reduced pressure to afford phenylglycyl-L-phenylalanine.

In a nitrogen atmosphere, phenylglycyl-L-phenylalanine (5.0 mmol, 1.0 equiv.), extra-dry DCM (25 mL, 0.2 M.), 2-(2-aminoacetylamino)acetic acid methyl·hydrochloride (6.0 mmol, 1.2 equiv.) and Et_3N (15 mmol, 3.0 equiv.) were added to 100 mL schlenk tube. The reaction mixture stirred at 0 °C for 30 min, followed by added *O*-benzotriazole-*N,N,N',N'*-tetramethyl-uronium-hexafluorophosphate (HBTU) (10 mmol, 2.0 equiv.). The reaction mixture was stirred at 0 °C for 12 h, and extracted with ethyl acetate (20 mL X 3). The combined organic layer was dried on anhydrous Na_2SO_4 and condensed under reduced pressure. The residue was purified by flash chromatography on silica gel to afford *N*-aryl glycine derivative **1ad** as a white solid (1.64 g, 77% yield) (ethyl acetate / methanol = 50:1, Rf = 0.2, m.p. 134 °C).

^{1}H NMR (400 MHz, $CDCl_3$) δ 7.60–7.57 (m, 1H), 7.47 (d, *J* = 7.7 Hz, 1H), 7.27–7.25 (m, 1H), 7.16–7.12 (m, 5H), 7.03–7.01 (m, 2H), 6.78–6.74 (m, 1H), 6.49–6.47 (m, 2H), 4.80–4.75 (m, 1H), 4.56 (s, 1H), 3.97–3.92 (m, 3H), 3.86–3.81 (m, 1H), 3.77–3.73 (m, 1H), 3.67–3.63 (m, 4H), 3.10–3.05 (m, 1H), 3.00–2.94 (m, 1H) ppm.

^{13}C NMR (101 MHz, $CDCl_3$) δ 171.8, 171.8, 170.4, 169.5, 147.1, 136.1, 129.3, 129.1, 129.1, 128.5, 126.7, 118.7, 113.0, 54.3, 52.3, 48.2, 42.8, 41.0, 37.7 ppm.

HRMS (ESI) m/z: $[M+H]^+$ Calcd for $C_{22}H_{27}N_4O_5^+$ 427.1976 Found 427.1975.

2.1.4 Preparation of ethyl 2-(phenylimino)acetate (15) [4]

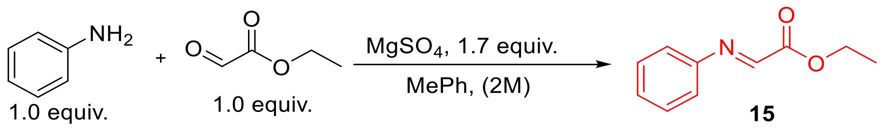

In a nitrogen atmosphere, ethyl glyoxylate (50 wt% in toluene, 5.0 mmol, 1.00 equiv.) in dry toluene (2.5 mL) were added anhydrous $MgSO_4$ (8.5 mmol, 1.7 equiv.) and aniline (5.0 mmol, 1.0 equiv.). It was stirred at rt. for 1.5 h. Then the solids were filtered off and the solvent removed under reduced pressure. The product **15** was

obtained as a yellow oil (882 mg, 98%) and used in the next step without further purification.

2.2 General procedure for the synthesis of thianthrenium salt

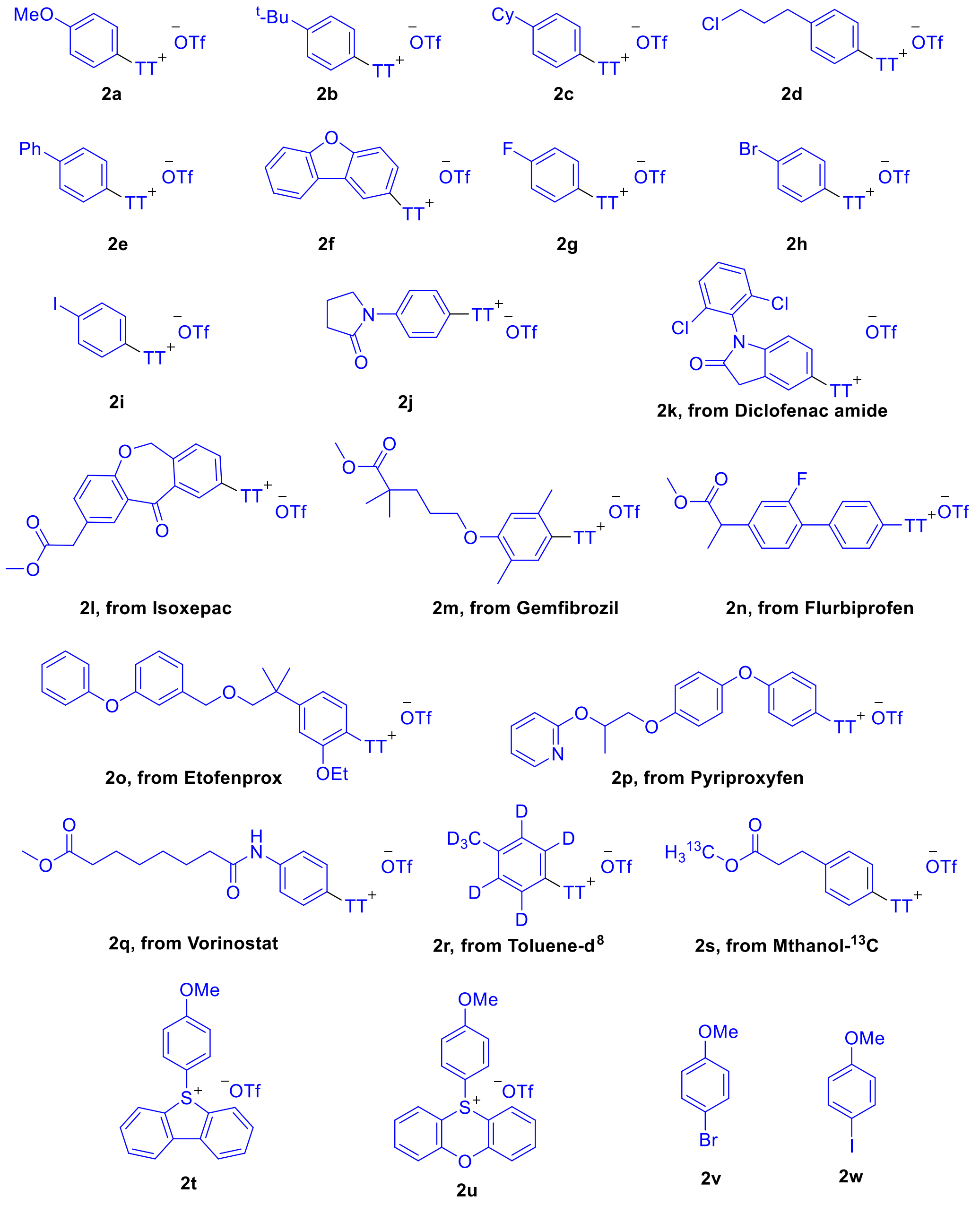

Figure S2. Aryl sulfonium salt and aryl halides.

The known aryl thianthrenium salt were synthesized according to the literatures. Spectral data matched that reported in the literatures.

The substrates **2a, 2b, 2c, 2d, 2e, 2f, 2g, 2h, 2i, 2j, 2l, 2m**, **2o, 2p,** and **2t** were synthesized according to the method reported in literature [5].

Substrates **1v** and **1w** were purchased from commercial sources.

Substrates **2k, 2o, 2p, 2q** and **2u** were synthesized in the following procedures.

2.2.1 Preparation of thianthrene *S*-oxide (TTSO) [5a]

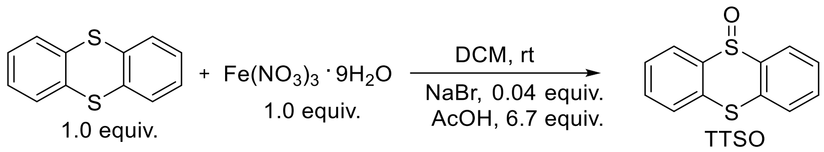

Thianthrene (50 mmol, 1.0 equiv), $Fe(NO_3)_3 \cdot 9H_2O$ (50 mmol, 1.0 equiv.) and NaBr (2 mmol, 0.04 equiv) were added to a 250 mL dry flask, followed by 150 mL of normal dichloromethane (DCM). Then, acetic acid (20 mmol, 0.4 equiv.) was injected into the reaction solution with a syringe under room temperature and air condition and detected by TLC until the end of the reaction. The dichloromethane was removed under reduced pressure. After the extraction with ethyl acetate (20 mL X 3), the combined organic layers were washed with brine, dried over anhydrous Na_2SO_4, filtrated and concentrated under reduced pressure. After purification by column chromatography on silica gel (ethyl acetate), the title compound was obtained as a white solid (11.5 g, 99% yield).

2.2.2 Procedure D for the synthesis of aryl thianthrenium salt

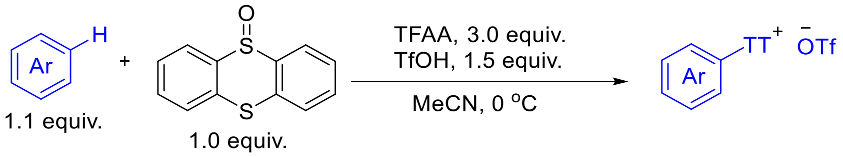

In a nitrogen atmosphere, thianthrene *S*-oxide (TTSO) (5.0 mmol, 1.0 equiv.), normal MeCN (25 mL, 0.2 M.) and arene (5.5 mmol, 1.1 equiv.) were added to 100 mL schlenk tube. The reaction mixture is then cooled to 0 °C and stirred at this temperature, followed by a slow drip of TFAA (15 mmol, 3.0 equiv.) and TfOH (7.5 mmol, 1.5 equiv.). The reaction mixture was stirred at 0 °C for 1 h, then stirred at room

temperature (25 °C) for 12 h, neutralized with saturated $NaHCO_3$ aqueous solution, and extracted with ethyl acetate (20 mL X 3). The combined organic layer was dried on anhydrous Na_2SO_4 and condensed under reduced pressure. The residue was purified by flash chromatography (DCM/MeOH = 20/1) on silica gel to afford aryl thianthrenium salt.

2.2.3 Procedure E for the synthesis of aryl thianthrenium salt

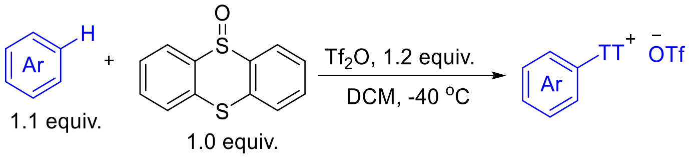

In a nitrogen atmosphere, thianthrene *S*-oxide (TTSO) (5.0 mmol, 1.0 equiv.), extra-dry DCM (25 mL, 0.2 M) and arene (5.5 mmol, 1.1 equiv.) were added to 100 mL schlenk tube. The reaction mixture is then cooled to -40 °C and stirred at this temperature, followed by a slow drip of Tf_2O (6 mmol, 1.2 equiv.). The reaction mixture was stirred at -40 °C for 30 min, then stirred at room temperature (25 °C) for 12 h, neutralized with saturated $NaHCO_3$ aqueous solution, and extracted with ethyl acetate (20 mL X 3). The combined organic layer was dried on anhydrous Na_2SO_4 and condensed under reduced pressure. The residue was purified by flash chromatography (DCM/MeOH = 20:1) on silica gel to afford aryl thianthrenium salt.

Preparation of 5-(1-(2,6-dichlorophenyl)-2-oxoindolin-5-yl)-5*H*-thianthren-5-ium trifluoromethanesulfonate (2k)

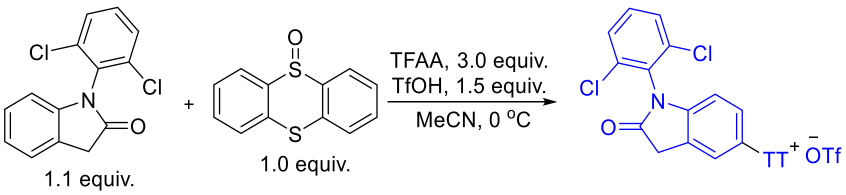

In a nitrogen atmosphere, thianthrene *S*-oxide (TTSO) (5.0 mmol, 1.0 equiv.), MeCN (25 mL, 0.2 M.) and 1-(2,6-dichlorophenyl)indolin-2-one (5.5 mmol, 1.1 equiv.) were added to 100 mL schlenk tube. The reaction mixture is then cooled to 0 °C and stirred at this temperature, followed by a slow drip of TFAA (15 mmol, 3.0 equiv.) and

TfOH (7.5 mmol, 1.5 equiv.). The reaction mixture was stirred at 0 °C for 1 h, then stirred at room temperature (25 °C) for 12 h, neutralized with saturated $NaHCO_3$ aqueous solution, and extracted with Ethyl acetate (20 mL X 3). The combined organic layer was dried on anhydrous Na_2SO_4 and condensed under reduced pressure. The residue was purified by flash chromatography on silica gel to afford **2k** as a brown solid (2.53 g, 79% yield) (DCM/MeOH = 20:1, Rf = 0.3, m.p. 146 °C).

^{1}H NMR (400 MHz, $CDCl_3$) δ 8.60–8.57 (m, 2H), 7.86–7.72 (m, 6H), 7.49–7.46 (m, 3H), 7.41–7.37 (m, 1H), 7.21–7.18 (m, 1H), 6.41 (d, *J* = 8.5 Hz, 1H), 3.76 (s, 2H) ppm.

^{13}C NMR (101 MHz, $CDCl_3$) δ 172.2, 147.8, 136.2, 135.2, 135.1, 134.8, 131.5, 130.4, 130.2, 129.5, 129.1, 129.0, 127.3, 125.3, 119.0, 117.2, 110.6, 35.3 ppm.

^{19}F NMR (376 MHz, $CDCl_3$) δ -78.2 ppm.

HRMS (ESI) m/z: [M-OTf]$^+$ Calcd for $C_{26}H_{17}Cl_2NOS_2^+$ 492.0045; Found 492.0040.

Preparation of 5-(4-(8-methoxy-8-oxooctanamido) phenyl)-5*H*-thianthren-5-ium trifluoromethanesulfonate (2q)

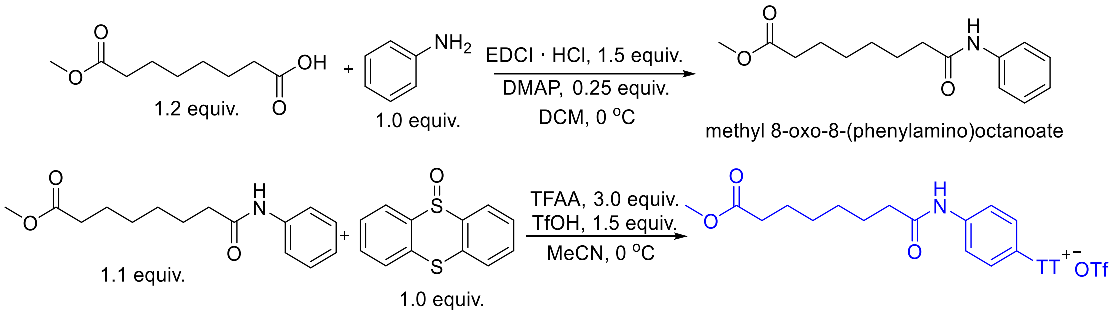

In a nitrogen atmosphere, monomethyl octanedioate (6.0 mmol, 1.2 equiv.), 4-dimethylaminopyridine (DMAP) (1.25 mmol, 0.25 equiv.), *N*-(3-dimethylaminopropyl)-*N*'-ethylcarbodiimide hydrochloride (EDCI·HCl) (7.5 mmol, 1.5 equiv.), aniline (5.0 mmol, 1.0 equiv.) and DCM (25 mL, 0.2 M.) were added to 100 mL schlenk tube. The reaction mixture is then cooled to 0 °C and stirred at this temperature for 1 h, then stirred at room temperature (25 °C) for 12 h. After the extraction with ethyl acetate (20 mL X 3), the combined organic layers were washed

with brine, dried over anhydrous Na_2SO_4, filtrated and concentrated under reduced pressure. After purification by column chromatography on silica gel (ethyl acetate/petroleum ether = 1:5), the title compound methyl 8-oxo-8-(phenylamino) octanoate was obtained as a white solid.

In a nitrogen atmosphere, thianthrene *S*-oxide (TTSO) (5.0 mmol, 1.0 equiv.), MeCN (25 mL, 0.2 M.) and 8-oxo-8-(phenylamino) octanoate (5.5 mmol, 1.1 equiv.) were added to 100 mL schlenk tube. The reaction mixture is then cooled to 0 °C and stirred at this temperature, followed by a slow drip of TFAA (15 mmol, 3.0 equiv.) and TfOH (7.5 mmol, 1.5 equiv.). The reaction mixture was stirred at 0 °C for 1 h, then stirred at room temperature (25 °C) for 12 h, neutralized with saturated $NaHCO_3$ aqueous solution, and extracted with ethyl acetate (20 mL X 3). The combined organic layer was dried on anhydrous Na_2SO_4 and condensed under reduced pressure. The residue was purified by flash chromatography on silica gel to afford **2q** as a yellow oil (1.51 g, 48% yield) (DCM/MeOH = 20:1, Rf = 0.3).

^{1}H NMR (400 MHz, $CDCl_3$) δ 9.51 (s, 1H), 8.32 (d, *J* = 7.7 Hz, 2H), 7.86–7.69 (m, 8H), 6.97 (d, *J* = 8.5 Hz, 2H), 3.63 (s, 3H), 2.38 (t, *J* = 7.5 Hz, 2H), 2.25 (t, *J* = 7.5 Hz, 2H), 1.62–1.52 (m, 4H), 1.33–1.24 (m, 4H) ppm.

^{13}C NMR (101 MHz, $CDCl_3$) δ 174.3, 173.5, 144.4, 136.2, 134.7, 134.2, 130.4, 130.1, 129.1, 121.2, 119.3, 113.9, 51.4, 37.0, 33.9, 28.7, 28.6, 25.1, 24.7 ppm.

^{19}F NMR (376 MHz, $CDCl_3$) δ -78.2 ppm.

HRMS (ESI) m/z: [M-OTf]$^+$ Calcd for $C_{27}H_{29}NO_3S_2^+$ 478.1505; Found 478.1501.

Preparation of 5-(4-(methyl-d^3)phenyl-2,3,5,6-d^4)-5*H*-thianthren-5-ium trifluoromethanesulfonate (2r)

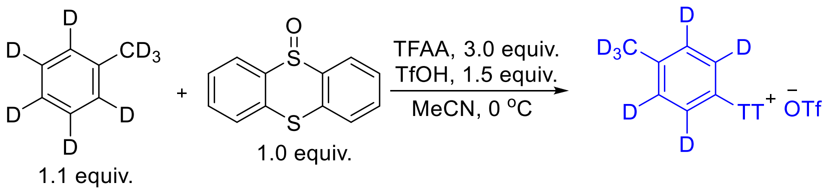

In a nitrogen atmosphere, thianthrene *S*-oxide (TTSO) (5.0 mmol, 1.0 equiv.), MeCN (25 mL, 0.2 M.) and toluene-d^8 (5.5 mmol, 1.1 equiv.) were added to 100 mL

schlenk tube. The reaction mixture is then cooled to 0 °C and stirred at this temperature, followed by a slow drip of TFAA (15 mmol, 3.0 equiv.) and TfOH (7.5 mmol, 1.5 equiv.). The reaction mixture was stirred at 0 °C for 1 h, then stirred at room temperature (25 °C) for 12 h, neutralized with saturated $NaHCO_3$ aqueous solution, and extracted with ethyl acetate (20 mL X 3). The combined organic layer was dried on anhydrous Na_2SO_4 and condensed under reduced pressure. The residue was purified by flash chromatography on silica gel to afford **2o** as a yellowish solid (1.97 g, 85% yield) [2] [9] (DCM/MeOH = 20:1, Rf = 0.3, m.p. 119 °C).

^{1}H NMR (400 MHz, $CDCl_3$) δ 8.57–8.53 (m, 2H), 7.84–7.78 (m, 4H), 7.76–7.72 (m, 2H) ppm.

^{13}C NMR (101 MHz, $CDCl_3$) δ 144.1, 136.4, 135.2, 135.1, 134.8, 134.8, 131.0 (t, *J* = 24.4 Hz), 130.2, 130.2, 130.2, 127.5 (t, *J* = 25.7 Hz), 122.4, 120.0, 119.2, 118.9, 118.8, 20.57–20.18 (m) ppm.

^{19}F NMR (376 MHz, $CDCl_3$) δ -78.1, -78.1 ppm.

HRMS (ESI) m/z: $[M-OTf]^+$ Calcd for $C_{19}H_8D_7S_2^+$ 314.1049; Found 314.1041.

Preparation of 5-(4-(3-(methoxy-^{13}C)-3-oxopropyl) phenyl)-5*H*-thianthren-5-ium trifluoromethanesulfonate (2s)

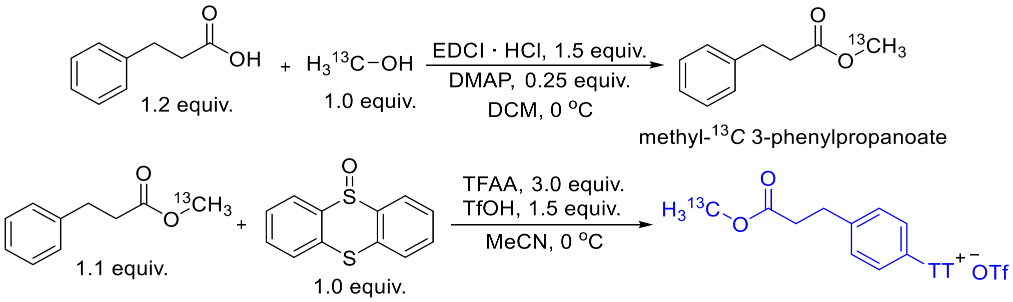

In a nitrogen atmosphere, 3-phenylpropionic acid (6.0 mmol, 1.2 equiv.), 4-dimethylaminopyridine (DMAP) (1.25 mmol, 0.25 equiv.), *N*-(3-dimethylaminopropyl)-*N*'-ethylcarbodiimide hydrochloride (EDCI·HCl) (7.5 mmol, 1.5 equiv.), and extra-dry DCM (25 mL, 0.2 M.) were added to 100 mL schlenk tube. The reaction mixture is then cooled to 0 °C and stirred at this temperature, followed by a slow drip of methanol-^{13}C (5.0 mmol, 1.0 equiv.). The reaction mixture was stirred at

0 °C for 1 h, then stirred at room temperature (25 °C) for 12 h. After the extraction with ethyl acetate (20 mL X 3), the combined organic layers were washed with brine, dried over anhydrous Na_2SO_4, filtrated and concentrated under reduced pressure. After purification by column chromatography on silica gel, the title compound methyl-^{13}C 3-phenylpropanoate was obtained as a colorless oil (0.83 g, 95% yield) (petroleum ether / ethyl acetate = 50:1, Rf = 0.3).

In a nitrogen atmosphere, thianthrene *S*-oxide (TTSO) (5.0 mmol, 1.0 equiv.), MeCN (25 mL, 0.2 M.) and methyl-^{13}C 3-phenylpropanoate (5.5 mmol, 1.1 equiv.) were added to 100 mL schlenk tube. The reaction mixture is then cooled to 0 °C and stirred at this temperature, followed by a slow drip of TFAA (15 mmol, 3.0 equiv.) and TfOH (7.5 mmol, 1.5 equiv.). The reaction mixture was stirred at 0 °C for 1 h, then stirred at room temperature (25 °C) for 12 h, neutralized with saturated $NaHCO_3$ aqueous solution, and extracted with ethyl acetate (20 mL X 3). The combined organic layer was dried on anhydrous Na_2SO_4 and condensed under reduced pressure. The residue was purified by flash chromatography on silica gel to afford **2p** as a colorless oil (2.46 g, 94% yield) (DCM/MeOH = 20:1, Rf = 0.3).

^{1}H NMR (400 MHz, $CDCl_3$) δ 8.44–8.41 (m, 2H), 7.80–7.75 (m, 4H), 7.69–7.65 (m, 2H), 7.22–7.19 (m, 2H), 7.04–7.00 (m, 2H), 3.51 (d, *J* = 147.1 Hz, 3H), 2.83 (t, *J* = 7.5 Hz, 2H), 2.49 (t, *J* = 7.5 Hz, 2H) ppm.

^{13}C NMR (101 MHz, $CDCl_3$) δ 172.2(d, *J* = 2.7 Hz), 146.5, 136.2, 134.9, 134.7, 130.5, 130.2, 129.9, 127.8, 121.0, 120.6 (q, *J* = 322.2 Hz) 118.1, 51.5, 34.2, 29.9 ppm.

^{19}F NMR (376 MHz, $CDCl_3$) δ -78.2 ppm.

HRMS (ESI) m/z: $[M-OTf]^+$ Calcd for $C_{21}{}^{13}CH_{19}O_2S_2{}^+$ 380.0855; Found 380.0855.

99% ^{13}C (calculated from HRMS).

Peak area ration = 2305483/(2305483+21063) × 100% = 99%.

Preparation of 10-(4-methoxyphenyl)-10*H*-phenoxathiin-10-ium trifluoromethanesulfonate (2u) [5i]

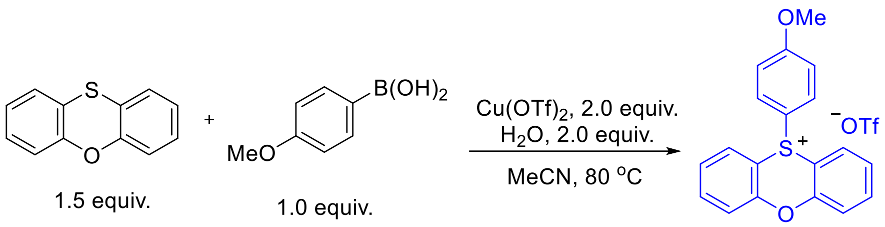

In a nitrogen atmosphere, 4-methoxyphenylboronic acid (3.0 mmol, 1.0 equiv.), $Cu(OTf)_2$ (6.0 mmol, 2.0 equiv.), phenoxathiin (4.5 mmol, 1.5 equiv.), water (6.0 mmol, 2.0 equiv.) and MeCN (15 mL, 0.2 M) were added to 100 mL schlenk tube. The reaction mixture is stirred at 80 °C for 6 h, and $NH_3{\cdot}H_2O$ (30 wt% in H_2O, 10 mL) was added after cooling room temperature. After the extraction with DCM (20 mL X 3), the combined organic layers were washed with brine, dried over anhydrous Na_2SO_4. The mixture was evaporated to dryness and subsequently treated with the minimum amount of DCM at -78 °C or Et_2O (50 mL), resulting in the formation of a precipitate. The product was then dried to yield **2u** (m.p. 128 °C).

^{1}H NMR (400 MHz, $CDCl_3$) δ 8.24–8.11 (m, 2H), 7.81–7.74 (m, 4H), 7.60–7.57 (m, 2H), 7.49–7.45 (m, 2H), 7.00–6.98 (m, 2H), 3.80 (s, 3H).

^{19}F NMR (376 MHz, $CDCl_3$) δ -78.1.

^{13}C NMR (101 MHz, $CDCl_3$) δ 164.6, 151.3, 136.3, 131.9, 131.8, 127.5, 120.9, 120.2, 117.0, 106.7, 56.0.

HRMS (ESI) m/z: $[M\text{-}OTf]^+$ Calcd for $C_{19}H_{15}O_2S^+$ 307.0787; Found 307.0784.

2.3 General procedure for the synthesis of alkenes

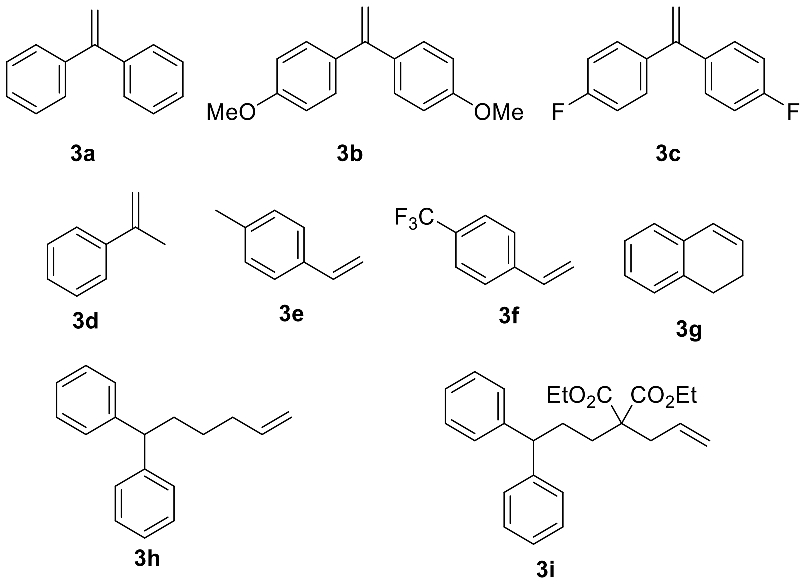

Figure S3. Alkenes.

Substrates **3a, 3d, 3e, 3f, 3g** and **3h** were purchased from commercial sources.

The substrates **3b, 3c, 3h** and **3i** were synthesized according to the method reported in literature [6].

The substrates **3h** and **3i** were synthesized according to the method reported in literature [7].

2.3.1 Procedure F for the synthesis of diphenyl olefins

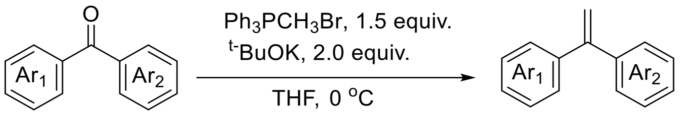

Ph_3PCH_3Br (7.5 mmol, 1.5 equiv.) was added to a flame-dried round-bottom flask, evacuated, backfilled with N_2 three times, and suspended in extra-dry THF (20 mL, 0.25 M). To this vigorously stirring heterogeneous solution was added *n*-butyllithium (10 mmol, 2.0 equiv.), and the reaction was allowed to stirred at 0 °C for 30 min until a bright yellow heterogeneous mixture was achieved. The appropriate ketone (5 mmol, 1.0 equiv.) was added slowly. Upon completing addition, the cooling bath was removed, and the reaction was allowed to stir for 12 h at ambient temperature, quenched with

saturated NH_4Cl (30 mL), diluted with water (30 mL) and extracted with ethyl acetate (20 mL X 3). The combined organic extracts were washed with brine (20 mL X 3), dried over Na_2SO_4 and concentrated under reduced pressure. The residue was purified by flash chromatography (petro ether / ethyl acetate = 100:1) on silica gel to afford alkenes.

Preparation of hex-5-ene-1,1-diyldibenzene (3h) [7]

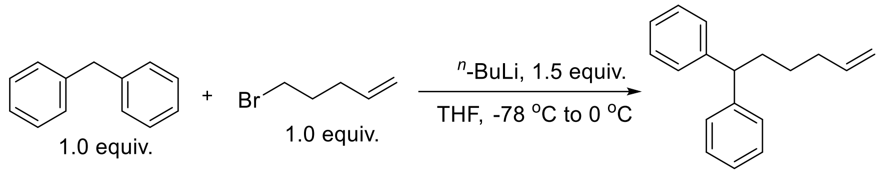

In a nitrogen atmosphere, diphenylmethane (5.0 mmol, 1.0 equiv.), ^{n}BuLi (1.5 equiv, 2.5 M in THF), and extra-dry THF (25 mL, 0.2 M.) were added to 100 mL schlenk tube at -78 °C and stirred at 0 °C for 1 h. The 5-bromo-1-pentene (5.0 mmol, 1.0 equiv.) was added dropwise at 0 °C and stirred at room temperature over 24 h. After the extraction with ethyl acetate (20 mL X 3), the combined organic layers were washed with brine, dried over anhydrous Na_2SO_4, filtrated and concentrated under reduced pressure. After purification by column chromatography on silica gel, the title compound was obtained **3h** as a colorless oil (956 mg, 81% yield) (petro ether / ethyl acetate = 100:1, Rf = 0.4).

Preparation of diethyl 2-allyl-2-(3,3-diphenylpropyl) malonate (3i) [7]

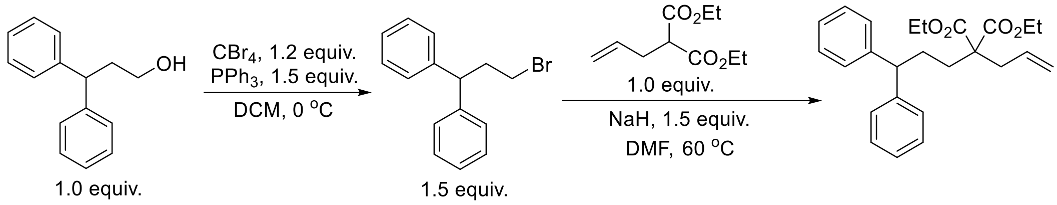

In a nitrogen atmosphere, 3,3-diphenylpropan-1-ol (5.0 mmol, 1.0 equiv.), CBr_4 (6.0 mmol, 1.2 equiv.), PPh_3 (7.5 mmol, 1.5 equiv.), and extra-dry DCM (25 mL, 0.2 M.) were added to 100 mL schlenk tube at 0 °C and stirred at this temperature for 1 h. The mixture was stirred at room temperature for 6 h. After the extraction with ethyl

acetate (20 mL X 3), the combined organic layers were washed with brine, dried over anhydrous Na_2SO_4, filtrated and concentrated under reduced pressure. After purification by column chromatography on silica gel (petro ether / ethyl acetate = 100:1), the title compound (3-bromopropane-1,1-diyl) dibenzene was obtained as a colorless oil (1.1 g, 80% yield).

In a nitrogen atmosphere, diethyl 2-allylmalonate (5.0 mmol, 1.0 equiv.) was added dropwise to a suspension of NaH (7.5 mmol, 1.5 equiv.) in anhydrous extra-dry DMF (25 mL, 0.2 M.) at 0 °C, and the reaction mixture was stirred for 30 min. Then, (3-bromopropane-1,1-diyl) dibenzene (7.5 mmol, 1.5 equiv.) was added and the reaction mixture was heated to 60 °C, and TLC monitored the complete conversion of diethyl allylmalonate, After the extraction with ethyl acetate (20 mL X 3), the combined organic layers were washed with brine, dried over anhydrous Na_2SO_4, filtrated and concentrated under reduced pressure. After purification by column chromatography on silica gel, the title compound was obtained **3i** as a colorless oil (1.67 g, 85% yield) (petro ether / ethyl acetate = 20:1, Rf = 0.4).

3. Optimization of reaction conditions

Table S1. Screening the solvents.

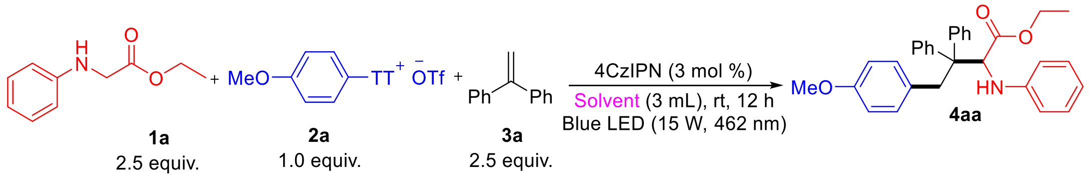

Entry[a]	Solvent	Yield [%]
1	DMSO	69
2	DCM	43
3	THF	19
4	MeCN	59

5	DMF	31
6	Toluene	15

[a] 0.15 mmol scale. Yields refer to isolated yields. Reaction under irradiation with blue LED lamp (462 nm, E27, 15 W).

Table S2. Screening the loading of aryl thianthrenium salt and *N*-phenylglycine ethyl ester.

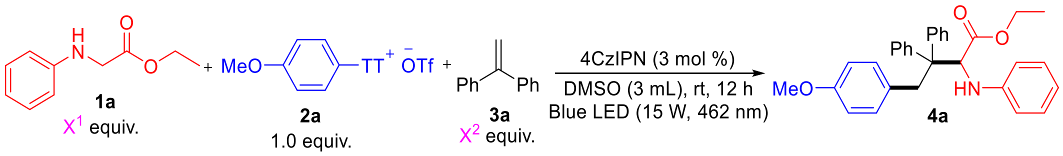

Entry[a]	X^1 (equiv.)	X^2 (equiv.)	Yield [%]
1	1.3	1.5	35
2	1.5	1.5	37
3	2.0	1.5	43
4	2.5	1.5	50
5	2.0	2.0	56
6	2.0	2.5	62
7	3.0	3.0	56
8	2.5	2.5	69

[a] 0.15 mmol scale. Yields refer to isolated yields. Reaction under irradiation with blue LED lamp (462 nm, E27, 15 W).

Table S3. Screening the concentration of reaction solution.

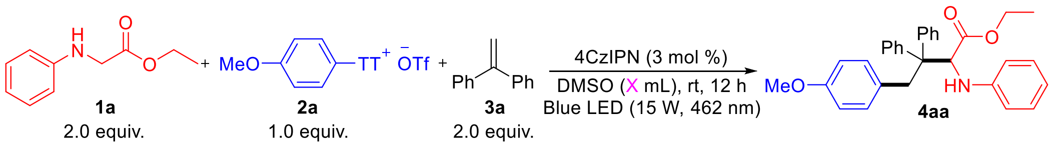

Entry[a]	X (mL)	Yield [%]
1	2	35
2	3	56

3	4	58
4	5	53

[a] 0.15 mmol scale. Yields refer to isolated yields. Reaction under irradiation with blue LED lamp (462 nm, E27, 15 W).

Table S4. Screening loading of photocatalyst.

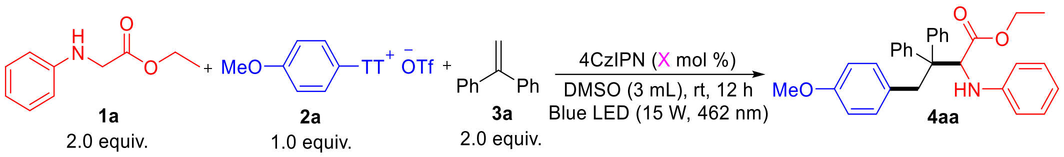

Entry[a]	X (mol %)	Yield [%]
1	2	55
2	3	56
3	5	44
4	10	32

[a] 0.15 mmol scale. Yields refer to isolated yields. Reaction under irradiation with blue LED lamp (462 nm, E27, 15 W).

Table S5. Screening the power of the blue light-emitting diode.

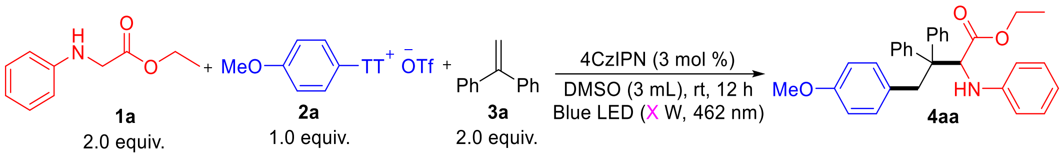

Entry[a]	X	Yield [%]
1	5	30
2	15	56
3	36	49

[a] 0.15 mmol scale. Yields refer to isolated yields. Reaction under irradiation with blue LED lamp (462 nm, E27, 15 W).

Table S6. Screening the reaction time.

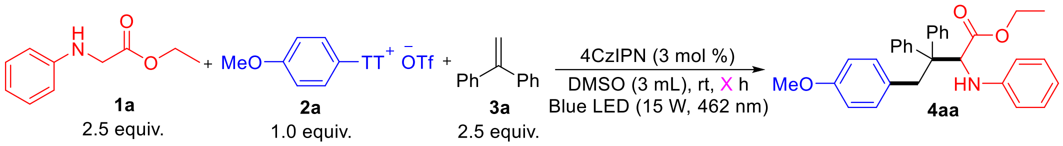

Entry[a]	X	Yield [%]
1	10	53
2	12	69
3	14	65
4	17	56
5	20	42

[a] 0.15 mmol scale. Yields refer to isolated yields. Reaction under irradiation with blue LED lamp (462 nm, E27, 15 W).

Table S7. Screening the additive.

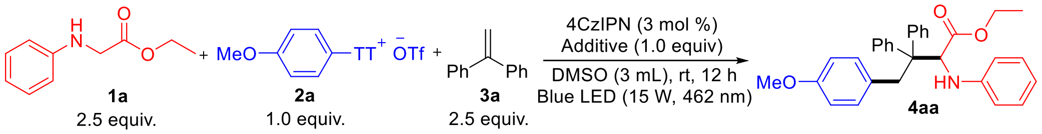

Entry[a]	Additive	Yield [%]
1	No	69
2	Py	77
3	K_2CO_3	50
4	Cs_2CO_3	53
5	Et_3N	65

[a] 0.15 mmol scale. Yields refer to isolated yields. Reaction under irradiation with blue LED lamp (462 nm, E27, 15 W).

Table S8. Screening the wavelength of blue light-emitting diode.

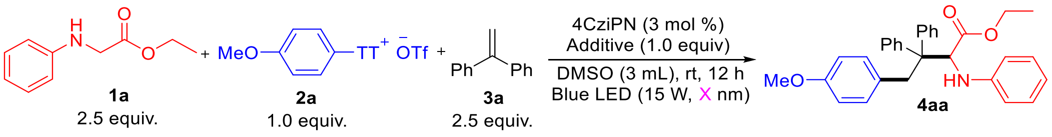

Entry[a]	Wavelength (nm)	Yield [%]
1[a]	395-400	30
2[a]	430-435	41
3[a]	440-445	39
4[a]	450-455	27
5[a]	460-465	39
6[a]	470-475	36
7[b]	462	77

[a] 0.15 mmol scale. Yields refer to isolated yields. Reaction under irradiation with PhotoSyn-10 parallel photoreactor purchased from Shanghai Quanhuan Technology Co., Ltd. [b] 0.15 mmol scale. Yields refer to isolated yields. Reaction under irradiation with blue LED lamp (462 nm, E27, 15 W) was purchased from Zhongshan Langniu Lighting Technology Co., Ltd.

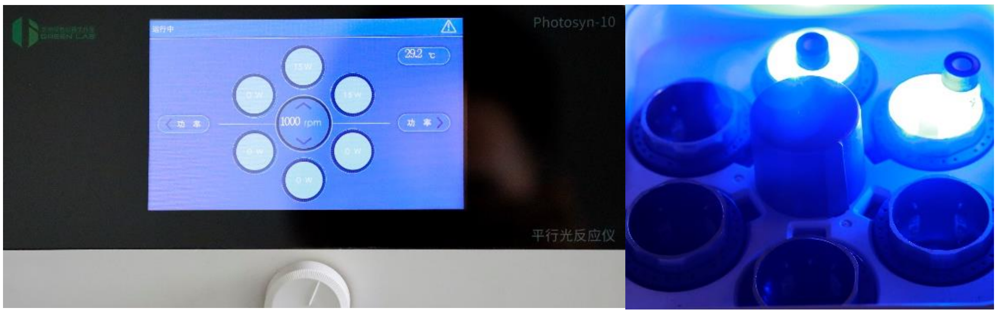

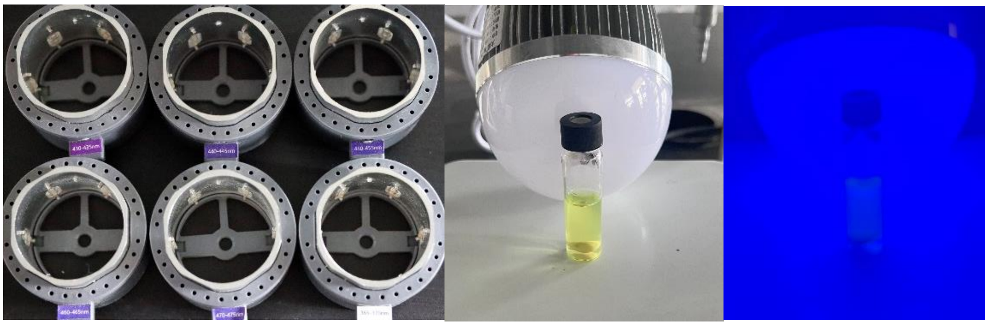

Figure S4. The photoreactors used for this work.

Table S9. Screening the photocatalyst.

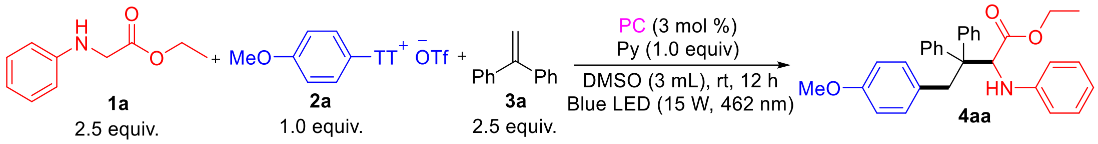

Entry[a]	Photocatalyst	Yield [%]
1	4CzIPN	69
2	Eosin B	trace
3	Eosin Y	trace
4	$Ru(bpy)_3[PF_6]_2$	53
5	$Ru(dmb)_3(PF_6)_2$	N.D.
6	$Ir[dF(CF_3)ppy]_2(dtbpy)[PF_6]$	61
7	$[Ir(dtbbpy)(ppy)_2][PF_6]$	13
8	Fluorescein	N.D.
9	Rose Bengal (Acid Red 94)	N.D.
10	$Mes\text{-}Acr^{+\cdot}[BF_4]$	61

[a] 0.15 mmol scale. Yields refer to isolated yields. Reaction under irradiation with blue LED lamp (462 nm, E27, 15 W).

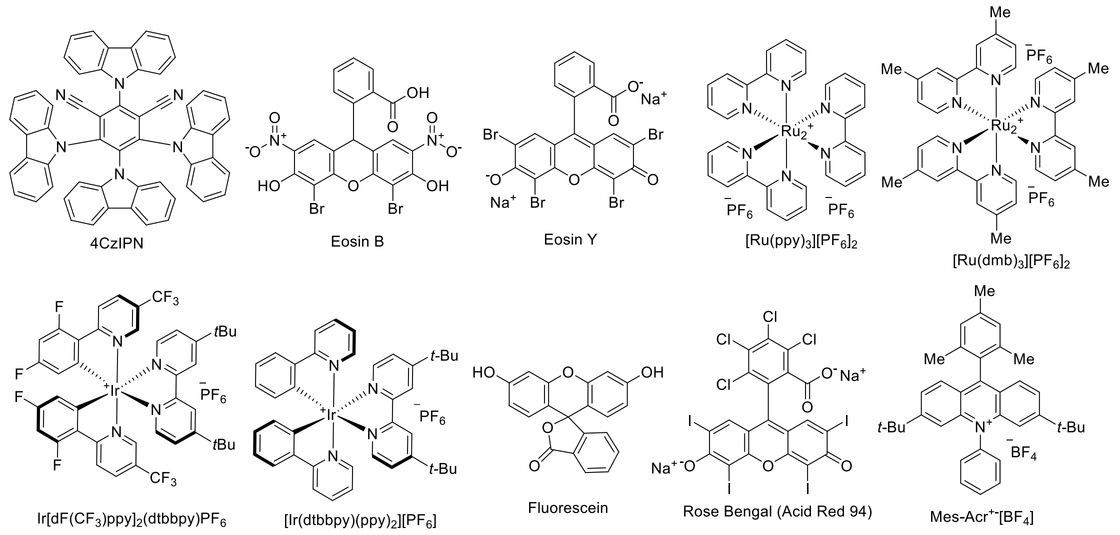

Figure S5. Photocatalysts.

Table S10. Electrochemical data for the employed photocatalysts.

Photocatalyst	$E_{1/2}$(PC*/PC$^{\bullet-}$) V vs. SCE	$E_{1/2}$(PC$^{\bullet+}$/PC*) V vs. SCE	$E_{1/2}$(PC$^{\bullet+}$/PC) V vs. SCE	$E_{1/2}$(PC/PC$^{\bullet-}$) V vs. SCE	E_T kcal/mol	Reference
4CzIPN	+1.43	-1.18	1.49	-1.24	60.0	[8], [9]
Esion B	+0.78	-1.37	-	-1.27	-	[10]
2H-Esion Y	+1.18	-1.60	+0.72	-1.14		[11], [12]
2Na-Esion Y	+0.76	-1.15	+0.83	-1.08	43.6	[11], [17]
[Ru(bpy)$_3$](PF$_6$)$_2$	+0.77	-0.81	+1.29	-1.33	46.5	[13], [14]
Ir[dF(CF$_3$)ppy]$_2$(dtbbpy)PF$_6$	+1.21	-0.89	+1.69	-1.37	60.1	[13], [14]
[Ir(ppy)$_2$(dtbbpy)](PF$_6$)	+0.66	-0.96	+1.21	-1.51	49.2	[13], [14]
Fluorescein	+1.25	-1.55	+0.87	-1.17	-	[10]
2Na-Rose bengal (Acid Red 94)	+0.81	-0.96	+0.84	-0.99	40.9	[10], [15], [18]
Mes-Acr^{+-}[BF$_4$]	+2.06	-	-	-0.57	-	[16]

4. General procedure for the stabilized alkyl radical-enabled conjugation of amino acid/peptide with aryl thianthrenium salt and characterization of products

4.1 Procedure G for the synthesis of conjugation of amino acid/peptide with aryl thianthrenium salt

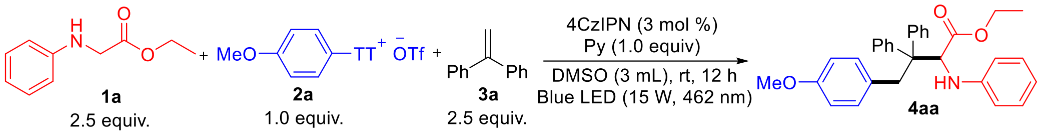

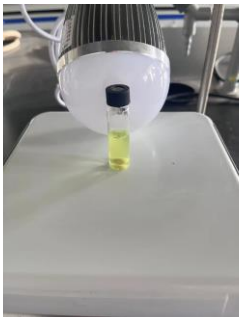

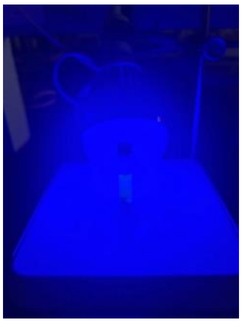

To an oven-dried transparent sample bottle (8 mL) equipped with a magnetic stir bar was added **1a** (0.375 mmol, 2.5 equiv.), **2a** (0.15 mmol, 1.0 equiv.), and 4CzIPN (0.0045 mmol, 3 mol%). The tube was sealed and connected to a vacuum line where it was evacuated and back-filled with N_2 three times. Then dry DMSO (4 mL), Py (0.15 mmol, 1.0 equiv.), and the **3a** (0.375 mmol, 2.5 equiv.) were added. During the reaction stirring process, the tube was constantly irradiated with a blue LED lamp (462 nm, E27, 15 W) keeping the reaction region located in the center of the LED lamp (2 - 3 cm away, with a cooling fan to keep the reaction temperature at 23 - 25 °C) for 12 hours before quenching with H_2O. After the extraction with ethyl acetate (20 mL X 3), the combined organic layer was washed with brine, dried over Na_2SO_4, filtrated, and concentrated under reduced pressure. The crude product was then purified by column chromatography on silica gel using petroleum ether and ethyl acetate as the eluent.

4.2 Characterization of products

ethyl 4-(4-methoxyphenyl)-3,3-diphenyl-2-(phenylamino)butanoate (4aa)

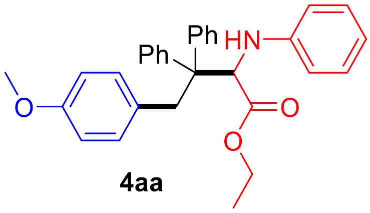

4aa

According to procedure G for the synthesis of conjugation of amino acid/peptide with aryl thianthrenium salt to afford **4aa** as a white solid (54 mg, 77% yield) (petro ether / ethyl acetate = 20:1, Rf = 0.4, m.p. 101 °C).

Replace $^{-}$OTf with $^{-}BF_4$: To a 100 mL flame-dried round-bottom flask, **2a** (5.0 mmol, 1.0 equiv.), and MeCN (25 mL, 0.2 M.), followed by a slow drip of saturated $NaBF_4$ aqueous solution. The reaction mixture was stirred for 8 h, and extracted with ethyl acetate (20 mL X 3). The combined organic layer was dried on anhydrous Na_2SO_4 and condensed under reduced pressure. The residue could be employed in procedure G for the synthesis of conjugation of amino acid/peptide with aryl thianthrenium salt to afford **4aa** as a white solid (50 mg, 71% yield).

^{1}H NMR (400 MHz, $CDCl_3$) δ 7.35–7.30 (m, 5H), 7.24–7.22 (m, 3H), 7.15 (t, *J* = 8.0 Hz, 2H), 7.10–7.08 (m, 2H), 6.74 (t, *J* = 7.2 Hz, 1H), 6.66– 6.60 (m, 4H), 6.56 (d, *J* = 8.0 Hz, 2H), 4.86 (d, *J* = 9.2 Hz, 1H), 4.11–4.07 (m, 1H), 3.91–3.85 (m, 1H), 3.83–3.78 (m, 1H), 3.76 (s, 3H), 3.66–3.56 (m, 2H), 0.87 (t, *J* = 7.1 Hz, 3H) ppm.

^{13}C NMR (101 MHz, $CDCl_3$) δ 172.2, 158.1, 146.6, 143.7, 143.3, 132.1, 130.0, 129.5, 129.2, 129.0, 127.6, 127.3, 126.8, 126.6, 118.6, 113.9, 112.8, 60.7, 59.5, 55.5, 55.1, 43.7, 13.6 ppm.

HRMS (ESI) m/z: $[M+H]^+$ Calcd for $C_{31}H_{32}NO_3^+$ 466.2377; Found 466.2376.

ethyl 4-(4-methoxyphenyl)-3,3-diphenyl-2-(phenylamino)butanoate (4ba)

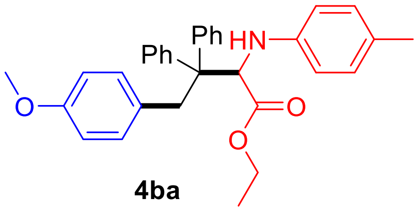

4ba

According to procedure G for the synthesis of conjugation of amino acid/peptide with aryl thianthrenium salt to afford **4ba** as a yellowish solid (46 mg, 64% yield) (petro ether / ethyl acetate = 20:1, Rf = 0.5, m.p. 97 °C).

^{1}H NMR (400 MHz, $CDCl_3$) δ 7.35–7.30 (m, 5H), 7.25–7.20 (m, 3H), 7.11–7.09 (m, 2H), 6.98–6.96 (m, 2H), 6.66–6.60 (m, 4H), 6.51–6.47 (m, 2H), 4.84 (s, 1H), 4.03–3.94 (m, 1H), 3.90–3.78 (m, 2H), 3.76 (s, 3H), 3.67–3.56 (m, 2H), 2.24 (s, 3H), 0.89 (t, *J* = 7.1 Hz, 3H) ppm.

^{13}C NMR (101 MHz, $CDCl_3$) δ 172.4, 158.0, 144.2, 143.73 143.4, 132.1, 130.0, 129.7, 129.6, 129.0, 127.7, 127.5, 127.3, 126.8, 126.5, 114.0, 112.8, 60.6, 59.7, 55.5, 55.1, 43.7, 20.4, 13.7 ppm.

HRMS (ESI) m/z: $[M+H]^+$ Calcd for $C_{32}H_{34}NO_3^+$ 480.2533; Found 480.2529.

ethyl 4-(4-methoxyphenyl)-2-((4-methoxyphenyl)amino)-3,3-diphenylbutanoate (4ca)

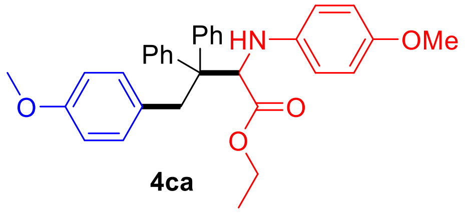

4ca

According to procedure G for the synthesis of conjugation of amino acid/peptide with aryl thianthrenium salt to afford **4ca** as a yellow solid (32 mg, 43% yield) (petro ether / ethyl acetate = 10:1, Rf = 0.4, m.p. 97 °C).

^{1}H NMR (400 MHz, $CDCl_3$) δ 7.34–7.29 (m, 5H), 7.24–7.21 (m, 3H), 7.15–7.08 (m, 2H), 6.76–6.73 (m, 2H), 6.64–6.60 (m, 4H), 6.55–6.52 (m, 2H), 4.77 (s, 1H), 3.92–3.74 (m, 9H), 3.67–3.51 (m, 2H), 0.88 (t, *J* = 7.2 Hz, 3H) ppm.

^{13}C NMR (101 MHz, $CDCl_3$) δ 172.4, 158.0, 152.7, 143.7, 143.4, 140.6, 132.1, 130.0, 129.6, 129.0, 127.5, 127.3, 126.8, 126.5, 115.3, 114.6, 112.7, 60.6, 60.4, 55.6, 55.5, 55.1, 43.8, 13.7 ppm.

HRMS (ESI) m/z: $[M+H]^+$ Calcd for $C_{32}H_{34}NO_4^+$ 496.2482; Found 496.2474.

ethyl 2-((4-bromophenyl)amino)-4-(4-methoxyphenyl)-3,3-diphenylbutanoate (4da)

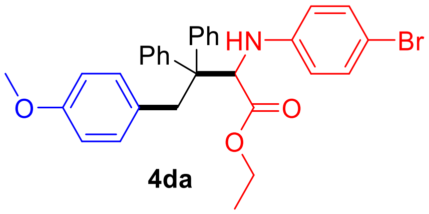

4da

According to procedure G for the synthesis of conjugation of amino acid/peptide with aryl thianthrenium salt to afford **4da** as a yellow solid (50 mg, 62% yield) (petro ether / ethyl acetate = 20:1, Rf = 0.4, m.p. 92 °C).

^{1}H NMR (400 MHz, $CDCl_3$) δ 7.30 (s, 5H), 7.24–7.22 (m, 5H), 7.07–7.04 (m, 2H), 6.62–6.56 (m, 4H), 6.44–6.40 (m, 2H), 4.77 (d, J = 11.2 Hz, 1H), 4.11 (d, J = 11.3 Hz, 1H), 3.89–3.77 (m, 2H), 3.75 (s, 3H), 3.64–3.54 (m, 2H), 0.88 (t, J = 7.1 Hz, 3H) ppm.

^{13}C NMR (101 MHz, $CDCl_3$) δ 171.9, 158.1, 145.6, 143.4, 143.1, 132.0, 132.0, 129.9, 129.5, 128.8, 127.6, 127.4, 126.9, 126.7, 115.4, 112.9, 110.3, 60.8, 59.5, 55.5, 55.1, 43.6, 13.7 ppm.

HRMS (ESI) m/z: $[M+H]^+$ Calcd for $C_{31}H_{31}BrNO_3^+$ 544.1482; Found 544.1487.

ethyl 2-((4-fluorophenyl)amino)-4-(4-methoxyphenyl)-3,3-diphenylbutanoate (4ea)

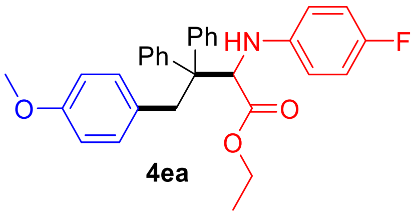

4ea

According to procedure G for the synthesis of conjugation of amino acid/peptide with aryl thianthrenium salt to afford **4ea** as a colorless solid (48 mg, 66% yield) (petro ether / ethyl acetate = 20:1, Rf = 0.3, m.p. 98 °C).

^{1}H NMR (400 MHz, $CDCl_3$) δ 7.34–7.30 (m, 5H), 7.26–7.21 (m, 3H), 7.11–7.09 (m, 2H), 6.90–6.84 (m, 2H), 6.63 (s, 4H), 6.52–6.48 (m, 2H), 4.78 (d, J = 11.4 Hz, 1H), 4.00 (d, J = 11.6 Hz, 1H), 3.94–3.76 (m, 5H), 3.69–3.56 (m, 2H), 0.89 (t, J = 7.1 Hz, 3H) ppm.

^{13}C NMR (101 MHz, $CDCl_3$) δ 172.1, 158.1, 156.4 (d, $J_{C\text{-}F}$ = 237.1 Hz), 143.6, 143.2, 142.8 (d, $J_{C\text{-}F}$ = 2.0 Hz), 132.1, 129.9, 129.5, 128.9, 127.6, 127.3, 126.9, 126.6, 115.7 (d, $J_{C\text{-}F}$ = 22.4 Hz), 114.9 (d, $J_{C\text{-}F}$ = 7.5 Hz), 112.8, 60.7, 60.2, 55.5, 55.1, 43.7, 13.7 ppm.

^{19}F NMR (376 MHz, $CDCl_3$) δ -126.5 ppm.

HRMS (ESI) m/z: $[M+H]^+$ Calcd for $C_{31}H_{31}FNO_3^+$ 484.2282; Found 484.2286.

ethyl 4-(4-methoxyphenyl)-3,3-diphenyl-2-((4-vinylphenyl)amino)butanoate (4fa)

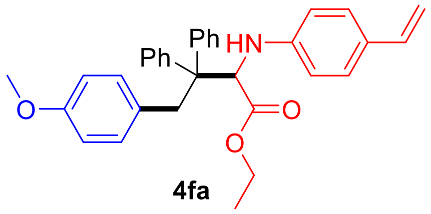

4fa

According to procedure G for the synthesis of conjugation of amino acid/peptide with aryl thianthrenium salt to afford **4fa** as a yellow solid (22 mg, 30% yield) (petro ether / ethyl acetate = 10:1, Rf = 0.5, m.p. 143 °C).

^{1}H NMR (400 MHz, $CDCl_3$) δ 7.33–7.29 (m, 5H), 7.23–7.19 (m, 5H), 7.08–7.05 (m, 2H), 6.63–6.56 (m, 5H), 6.51–6.48 (m, 2H), 5.55–5.51 (m, 1H), 5.04–5.02 (m, 1H), 4.86–4.83 (m, 1H), 4.17–4.14 (m, 1H), 3.90–3.77 (m, 2H), 3.75 (s, 3H), 3.64–3.54 (m, 2H), 0.87 (t, J = 7.1 Hz, 3H) ppm.

^{13}C NMR (101 MHz, $CDCl_3$) δ 172.1, 158.1, 146.3, 143.6, 143.2, 136.4, 132.9, 129.9, 129.5, 128.9, 128.3, 127.6, 127.4, 127.3, 126.9, 126.6, 113.8, 112.8, 110.0, 60.8, 59.4, 55.5, 55.1, 43.7, 13.7 ppm.

HRMS (ESI) m/z: $[M+H]^+$ Calcd for $C_{33}H_{34}NO_3^+$ 492.2533; Found 492.2542.

phenyl 4-(4-methoxyphenyl)-3,3-diphenyl-2-(*p*-tolylamino)butanoate (4ga)

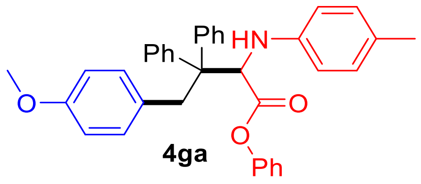

4ga

According to procedure G for the synthesis of conjugation of amino acid/peptide with aryl thianthrenium salt to afford **4ga** as a yellow solid (55 mg, 70% yield) (petro ether / ethyl acetate = 30:1, Rf = 0.4, m.p. 135 °C).

^{1}H NMR (400 MHz, $CDCl_3$) δ 7.49–7.47 (m, 2H), 7.42–7.36 (m, 3H), 7.31–7.29 (m, 3H), 7.26–7.21 (m, 4H), 7.15–7.11 (m, 1H), 7.06–7.04 (m, 2H), 6.72–6.61 (m, 6H), 6.55–6.52 (m, 2H), 5.10 (d, J = 11.3 Hz, 1H), 4.21 (d, J = 11.4 Hz, 1H), 3.78 (s, 3H), 3.76–3.65 (m, 2H), 2.29 (s, 3H) ppm.

^{13}C NMR (101 MHz, $CDCl_3$) δ 171.5, 158.2, 150.3, 144.0, 143.7, 143.2, 132.1, 130.1, 129.8, 129.7, 129.1, 128.9, 128.1, 127.8, 127.5, 127.0, 126.8, 125.7, 121.2, 114.1, 112.9, 60.3, 55.4, 55.1, 43.7, 20.4 ppm.

HRMS (ESI) m/z: $[M+H]^+$ Calcd for $C_{33}H_{34}NO_3^+$ 528.2533; Found 528.2536.

benzyl 4-(4-methoxyphenyl)-3,3-diphenyl-2-(*p*-tolylamino)butanoate (4ha)

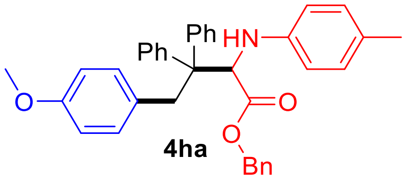

4ha

According to procedure G for the synthesis of conjugation of amino acid/peptide with aryl thianthrenium salt to afford **4ha** as a white solid (55 mg, 68% yield) (petro ether / ethyl acetate = 50:1, Rf = 0.4, m.p. 77 °C).

^{1}H NMR (400 MHz, $CDCl_3$) δ 7.30–7.27 (m, 5H), 7.25–7.19 (m, 6H), 7.12–7.10 (m, 2H), 6.97 (d, J = 8.0 Hz, 2H), 6.91–6.89 (m, 2H), 6.62 (s, 4H), 6.51–6.45 (m, 2H), 4.96–4.91 (m, 2H), 4.73 (d, J = 12.3 Hz, 1H), 4.01–3.99 (m, 1H), 3.77 (s, 3H), 3.74–3.670(m, 1H), 3.52–3.49 (m, 1H), 2.27 (s, 3H) ppm.

^{13}C NMR (101 MHz, $CDCl_3$) δ 172.3, 158.1, 144.2, 143.5, 143.2, 135.3, 132.1, 130.0, 129.8, 129.7, 129.0, 128.2, 127.9, 127.9, 127.5, 127.3, 126.8, 126.6, 114.2, 112.8, 66.5, 59.8, 55.7, 55.1, 43.9, 20.4 ppm.

HRMS (ESI) m/z: $[M+H]^+$ Calcd for $C_{37}H_{36}NO_3^+$ 542.2690; Found 542.2697.

benzo[*d*][1,3]dioxol-5-ylmethyl 4-(4-methoxyphenyl)-3,3-diphenyl-2-(phenylamino)butanoate (4ia)

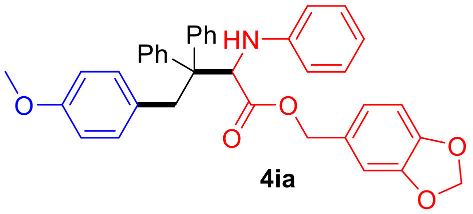

4ia

According to procedure G for the synthesis of conjugation of amino acid/peptide with aryl thianthrenium salt to afford **4ia** as a white solid (55 mg, 64% yield) (petro ether / ethyl acetate = 10:1, Rf = 0.3, m.p. 132 °C).

^{1}H NMR (400 MHz, $CDCl_3$) δ 7.30–7.25 (m, 5H), 7.24–7.19 (m, 3H), 7.17–7.07 (m, 4H), 6.75 (t, *J* = 7.3 Hz, 1H), 6.64–6.60 (m, 5H), 6.54 (d, *J* = 8.1 Hz, 2H), 6.43 (d, *J* = 7.9 Hz, 1H), 6.33 (s, 1H), 5.96–5.89 (m, 2H), 4.92 (d, *J* = 11.4 Hz, 1H), 4.81 (d, *J* = 12.0 Hz, 1H), 4.61 (d, *J* = 12.0 Hz, 1H), 4.09 (d, *J* = 11.5 Hz, 1H), 3.75 (s, 3H), 3.69–3.64 (m, 1H), 3.57–3.50 (m, 1H) ppm.

^{13}C NMR (101 MHz, $CDCl_3$) δ 172.1, 158.1, 147.4, 147.3, 146.5, 143.5, 143.1, 132.1, 129.9, 129.6, 129.2, 128.9, 128.9, 127.5, 127.4, 126.8, 126.7, 122.2, 118.7, 114.0, 112.8, 109.0, 107.8, 101.0, 66.5, 59.5, 55.6, 55.1, 43.7 ppm.

HRMS (ESI) m/z: $[M+H]^+$ Calcd for $C_{37}H_{34}NO_5^+$ 572.241; Found 572.2430.

4-(4-methoxyphenyl)-3,3-diphenyl-2-(*p*-tolylamino)butanenitrile (4ja)

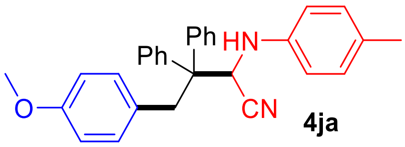

4ja

According to procedure G for the synthesis of conjugation of amino acid/peptide with aryl thianthrenium salt to afford **4ja** as a white solid (45 mg, 69% yield) (Petro ether / ethyl acetate = 20:1, Rf = 0.2, m.p. 144 °C).

^{1}H NMR (400 MHz, DMSO-d_6) δ 7.49–7.29 (m, 8H), 7.23–7.09 (m, 2H), 7.03–7.01 (m, 2H), 6.83–6.81 (m, 2H), 6.56–6.54 (m, 2H), 6.29–6.27 (m, 2H), 4.93 (d, *J* =

10.8 Hz, 1H), 4.48 (d, J = 10.8 Hz, 1H), 3.99–3.83 (m, 1H), 3.63 (s, 3H), 3.25–3.08 (m, 1H), 2.21 (s, 3H) ppm.

^{13}C NMR (101 MHz, DMSO-d_6) δ 157.9, 142.7, 142.5, 141.7, 131.3, 129.6, 129.1, 128.1, 127.8, 127.8,127.5, 127.4, 127.1, 119.3, 114.6, 113.0, 54.9, 54.3, 49.7, 42.2, 20.1 ppm.

HRMS (ESI) m/z: [M+H]$^+$ Calcd for $C_{30}H_{29}N_2O^+$ 433.2274; Found 433.2283.

4-(4-methoxyphenyl)-*N*-(1-methyl-1*H*-pyrazol-5-yl)-3,3-diphenyl-2-(*p*-tolylamino)butanamide (4ka)

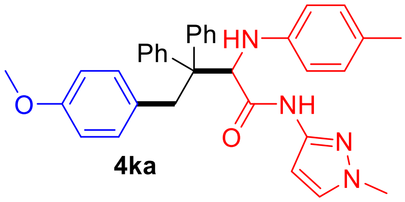

4ka

According to procedure G for the synthesis of conjugation of amino acid/peptide with aryl thianthrenium salt to afford **4ka** as a white solid (58 mg, 73% yield) (petro ether / ethyl acetate = 3:1, Rf = 0.2, m.p. 165 °C).

^{1}H NMR (400 MHz, CDCl$_3$) δ 8.44 (s, 1H), 7.49–7.47 (m, 2H), 7.32–7.29 (m, 2H), 7.25–7.19 (m, 2H), 7.16–7.13 (m, 3H), 6.97–6.95 (m, 2H), 6.86–6.84 (m, 2H), 6.76–6.74 (m, 2H), 6.62 (d, J = 2.3 Hz, 1H), 6.57–6.55 (m, 2H), 6.48–6.46 (m, 2H), 4.55 (d, J = 13.0 Hz, 1H), 4.41 (d, J = 1.6 Hz, 1H), 4.16 (s, 1H), 3.70 (s, 3H), 3.65 (s, 3H), 2.98 (d, J = 13.0 Hz, 1H), 2.21 (s, 3H) ppm.

^{13}C NMR (101 MHz, CDCl$_3$) δ 170.1, 157.8, 146.1, 144.2, 143.0, 141.9, 132.5, 130.3, 129.9, 129.8, 129.1, 128.9, 128.8, 127.9, 127.7, 127.2, 126.6, 114.1, 112.5, 96.7, 62.5, 56.9, 54.9, 43.9, 38.5, 20.3 ppm.

HRMS (ESI) m/z: [M+H]$^+$ Calcd for $C_{34}H_{35}N_4O_2^+$ 531.2755; Found 531.2748.

4-(4-methoxyphenyl)-*N*,*N*-dimethyl-3,3-diphenyl-2-(*p*-tolylamino)butanamide (4la)

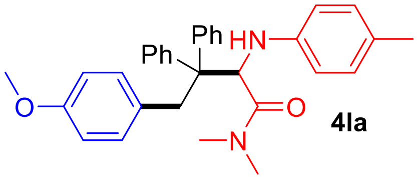

4la

According to procedure G for the synthesis of conjugation of amino acid/peptide with aryl thianthrenium salt to afford **4la** as a white solid (49 mg, 69% yield) (petro ether / ethyl acetate = 5:1, Rf = 0.3, m.p. 153 °C).

^{1}H NMR (400 MHz, $CDCl_3$) δ 7.49–7.47 (m, 2H), 7.33–7.28 (m, 3H), 7.24–7.19 (m, 5H), 6.97–6.91 (m, 4H), 6.65–6.63 (m, 2H), 6.32–6.30 (m, 2H), 5.07 (s, 1H), 4.36–4.17 (m, 2H), 3.75–3.72 (m, 4H), 2.63 (s, 3H), 2.21 (s, 3H), 2.20 (s, 3H) ppm.

^{13}C NMR (101 MHz, $CDCl_3$) δ 171.8, 158.0, 145.7, 143.9, 132.0, 130.8, 130.1, 129.7, 129.7, 129.6, 127.7, 127.6, 127.3, 126.6, 126.5, 113.3, 113.0, 60.1, 55.1, 54.6, 41.0, 36.6, 35.8, 20.4 ppm.

HRMS (ESI) m/z: $[M+H]^+$ Calcd for $C_{32}H_{35}N_2O_2^+$ 479.2693; Found 479.2692.

(*R*)-2-((*tert*-butoxycarbonyl)amino)-2-phenylethyl 4-(4-methoxyphenyl)-3,3-diphenyl-2-(*p*-tolylamino)butanoate (4ma)

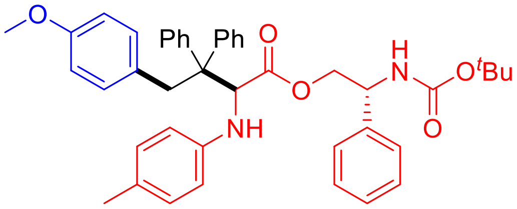

4ma, from Phenylglycinol

According to procedure G for the synthesis of conjugation of amino acid/peptide with aryl thianthrenium salt to afford **4ma** with two isomers as a reddish-brown oil (74 mg, 74% yield) (DCM, Rf = 0.5, d.r. = 1:1, d.r. is determined by ^{1}H NMR). The data is written as observed.

^{1}H NMR (400 MHz, $CDCl_3$) δ 7.39–7.27 (m, 5H), 7.26–7.15 (m, 6H), 7.12–7.02 (m, 2H), 6.94–6.84 (m, 4H), 6.61–6.53 (m, 4H), 6.32–6.24 (m, 2H), 4.82 (s, 1H), 4.66–4.32 (m, 2H), 4.14–3.83 (m, 3H), 3.75 (s, 3H), 3.71–3.60 (m, 1H), 3.50–3.42 (m, 1H), 2.23–2.22 (m, 3H), 1.45–1.38 (m, 9H) ppm.

^{13}C NMR (101 MHz, CDCl$_3$) δ 172.2, 172.1, 158.1, 158.1, 154.9, 154.8, 144.0, 143.8, 143.7, 143.5, 143.3, 143.0, 138.8, 132.1, 132.0, 129.9, 129.9, 129.8, 129.8, 129.6, 129.5, 128.8, 128.7, 128.4, 128.3, 128.0, 127.8, 127.7 127.6, 127.5, 127.5, 127.4, 127.3, 127.1, 126.9, 126.8, 126.8, 126.4, 126.2, 114.1, 113.8, 112.8, 112.8, 79.7, 79.5, 67.3, 66.2, 60.2, 60.0, 55.5, 55.1, 28.3, 20.4, 20.4 ppm.

HRMS (ESI) m/z: [M+H]$^+$ Calcd for $C_{43}H_{47}N_2O_5^+$ 671.3479; Found 671.3485.

_N_-(((_R_)-3-(3-fluoro-4-morpholinophenyl)-2-oxooxazolidin-5-yl)methyl)-4-(4-methoxyphenyl)-3,3-diphenyl-2-(phenylamino)butanamide (4na)

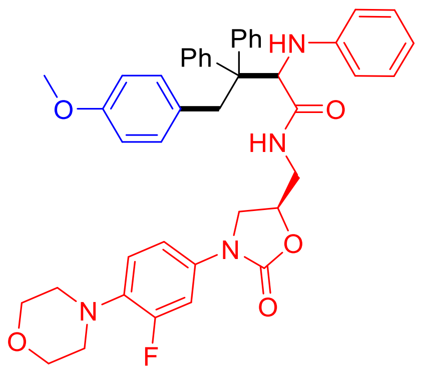

4na, from Linezolid

According to procedure G for the synthesis of conjugation of amino acid/peptide with aryl thianthrenium salt to afford **4na** with two isomers as a yellowish solid (50 mg, 47% yield) (DCM / MeOH = 100:1, Rf = 0.3, m.p. 159 °C, d.r. = 1:1, d.r. is determined by ^{1}H NMR and ^{19}F NMR). The data is written as observed.

^{1}H NMR (400 MHz, CDCl$_3$) δ 7.48–7.45 (m, 2H), 7.43–7.27 (m, 4H), 7.22–7.11 (m, 4H), 7.06–6.90 (m, 3H), 6.82–6.54 (m, 8H), 6.50–6.47 (m, 1H), 6.43–6.40 (m, 1H), 4.41–4.26 (m, 3H), 4.23–4.18 (m, 1H), 3.90–3.87(m, 4H), 3.79–3.71 (m, 1H), 3.70 (d, J = 1.2 Hz, 3H), 3.51–3.31 (m, 2H), 3.08–3.06 (m, 4H), 3.04–2.95 (m, 2H) ppm.

^{13}C NMR (101 MHz, CDCl$_3$) δ 173.1, 173.1, 158.0, 157.9, 156.7, 156.7, 154.3, 154.2, 153.9, 153.6, 146.4, 146.0, 143.5, 143.4, 141.8, 141.6, 132.6, 130.3, 129.4, 129.3, 129.0, 128.9, 128.8, 128.0, 128.0, 127.8, 127.5, 127.4, 126.8, 119.9, 119.7, 118.7, 1, 114.0, 113.7, 113.6, 113.5, 112.6, 111.9, 107.4, 107.2, 71.2, 71.2, 66.9, 62.3, 61.7, 56.4, 56.4, 55.0, 51.1, 51.1, 51.0, 47.9, 47.3, 43.9, 43.9, 41.7, 40.7 ppm.

^{19}F NMR (376 MHz, $CDCl_3$) δ -120.29, -120.54 ppm.

HRMS (ESI) m/z: $[M+H]^+$ Calcd for $C_{43}H_{44}FN_4O_5^+$ 715.2390; Found 715.3283.

(5*R*,5a*R*,8a*R*,9*R*)-8-oxo-9-(3,4,5-trimethoxyphenyl)-5,5a,6,8,8a,9-hexahydrofuro[3',4':6,7]naphtho[2,3-d][1,3]dioxol-5-yl 4-(4-methoxyphenyl)-3,3-diphenyl-2-(*p*-tolylamino)butanoate (4oa)

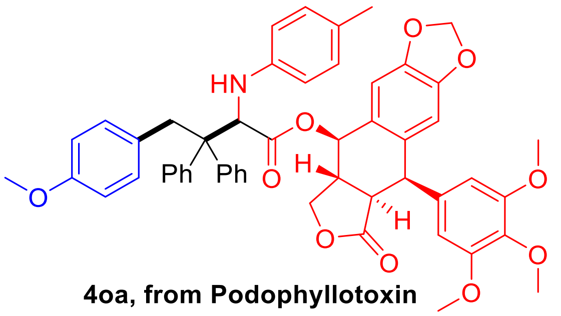

4oa, from Podophyllotoxin

According to procedure G for the synthesis of conjugation of amino acid/peptide with aryl thianthrenium salt to afford **4oa** with two isomers as a yellow solid (100 mg, 79% yield) (petro ether / ethyl acetate = 1:1, Rf = 0.5, Rf = 0.3, m.p. 165 °C, d.r. = 1:1, d.r. is determined by ^{1}H NMR). The data is written as observed.

^{1}H NMR (400 MHz, $CDCl_3$) δ 7.33–7.18 (m, 10H), 7.00 (d, J = 8.1 Hz, 1H), 6.86 (d, J = 8.1 Hz, 1H), 6.64–6.52 (m, 5H), 6.46–6.44 (m, 2H), 6.28 (d, J = 6.2 Hz, 2H), 6.02–5.83 (m, 3H), 5.63–5.56 (m, 1H), 4.97–4.93 (m, 1H), 4.51–4.44 (m, 1H), 4.02–3.84 (m, 7H), 3.75 (s, 6H), 3.66 (s, 3H), 3.54–3.45 (m, 1H), 2.74–2.66 (m, 1H), 2.42–2.29 (m, 1H), 2.23 (s, 1.5H), 2.15 (s, 1.5H) ppm.

^{13}C NMR (101 MHz, $CDCl_3$) δ 173.6, 173.5, 173.3, 173.1, 158.3, 158.2, 152.5, 152.5, 147.9, 147.9, 147.3, 147.2, 143.6, 143.5, 142.8, 142.7, 137.1, 137.0, 134.9, 134.8, 132.1, 132.0, 130.0, 130.0, 129.8, 129.4, 129.0, 128.8, 128.7, 128.6, 127.7, 127.6, 127.5, 127.4, 127.4, 127.2, 127.1, 127.0, 114.4, 114.1, 112.9, 112.9, 109.4, 109.2, 108.0, 107.8, 107.8, 107.5, 101.5, 101.4, 74.1, 71.4, 71.1, 60.7, 60.7, 60.1, 59.9, 56.0, 55.2, 55.0, 54.9, 45.6, 45.3, 44.0, 43.6, 43.4, 43.3, 38.5, 38.2, 20.3, 20.3 ppm.

HRMS (ESI) m/z: $[M+H]^+$ Calcd for $C_{52}H_{50}NO_{10}^+$ 848.3429; Found 848.3423.

N-(1-(adamantan-1-yl)ethyl)-4-(4-methoxyphenyl)-3,3-diphenyl-2-(phenylamino)butanamide (4pa)

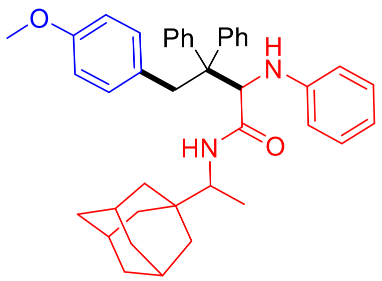

4pa, from Rimantadine

According to procedure G for the synthesis of conjugation of amino acid/peptide with aryl thianthrenium salt to afford **4pa** with two isomers as a white solid (40 mg, 45% yield) (petro ether / ethyl acetate = 10:1, Rf = 0.4, Rf = 0.3, m.p. 206 °C, d.r. = 1:1, d.r. is determined by ^{1}H NMR). The data is written as observed.

^{1}H NMR (400 MHz, $CDCl_3$) δ 7.52–7.48 (m, 2H), 7.40–7.28 (m, 3H), 7.19–7.07 (m, 5H), 6.83–6.76 (m, 3H), 6.68–6.20 (m, 7H), 4.67–4.57 (m, 1H), 4.44–4.38 (m, 1H), 4.30–4.24 (m, 1H), 3.71 (s, 1.5H), 3.70 (s, 1.5H), 3.45–3.34 (m, 1H), 2.95–2.91 (m, 1H), 1.80–1.74 (m, 3H), 1.60–1.43 (m, 5H), 1.34–1.21 (m, 3H), 1.08–1.05 (m, 1H), 1.01–0.93 (m, 3H), 0.75 (d, J = 6.8 Hz, 1.7H), 0.35 (d, J = 6.9 Hz, 1.3H) ppm.

^{13}C NMR (101 MHz, $CDCl_3$) δ 171.0, 170.5, 157.8, 146.9, 146.2, 144.2, 143.9, 143.1, 142.6, 132.7, 130.3, 129.4, 129.4, 129.3, 129.2, 129.2, 128.2, 128.1, 127.6, 127.5, 127.0, 127.0, 126.5, 126.4, 119.5, 119.5, 114.2, 114.2, 112.5, 62.5, 60.9, 56.2, 56.1, 55.0, 55.0, 53.6, 52.4, 44.4, 44.2, 38.0, 37.9, 36.8, 36.7, 35.9, 34.6, 28.1, 28.0, 13.8, 13.3 ppm.

HRMS (ESI) m/z: $[M+H]^+$ Calcd for $C_{41}H_{47}N_2O_2^+$ 599.3632; Found 599.3628.

(3*S*,4*R*,5*S*,6*S*)-3,4,5-tris(benzyloxy)-6-((benzyloxy)methyl)tetrahydro-2*H*-pyran-2-yl 4-(4-methoxyphenyl)-3,3-diphenyl-2-(phenylamino)butanoate (4qa)

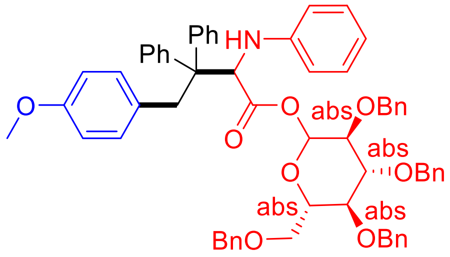

4qa, **from D-glucopyranose**

According to procedure G for the synthesis of conjugation of amino acid/peptide with aryl thianthrenium salt to afford **4qa** with two isomers as a yellow solid (91 mg, 63% yield) (petro ether / ethyl acetate = 5:1, Rf = 0.5, Rf = 0.3, m.p. 70 °C, d.r. = 1:1, d.r. is determined by ^{1}H NMR). The data is written as observed.

^{1}H NMR (400 MHz, $CDCl_3$) δ 7.43–7.04 (m, 33H), 6.80–6.59 (m, 6H), 6.34–6.25 (m, 1H), 5.08–4.36 (m, 9H), 4.25–3.96 (m, 2H), 3.79 (m, 3H), 3.67–2.62 (m, 7H) ppm.

^{13}C NMR (101 MHz, $CDCl_3$) δ 171.2, 170.6, 15f8.2, 158.0, 146.3, 146.1, 143.1, 142.7, 142.7, 142.4, 138.7, 138.6, 138.6, 138.4, 137.8, 137.8, 137.5, 132.1, 132.1, 129.9, 129.6, 129.3, 129.2, 128.8, 128.3, 128.2, 128.2, 128.2, 128.2, 128.0, 127.9, 127.9, 127.8, 127.7, 127.6, 127.6, 127.5, 127.4, 127.4, 127.3, 127.1, 126.7, 126.5, 118.7, 118.6, 114.0, 112.9, 112.8, 91.0, 90.7, 81.3, 81.0, 78.8, 78.4, 76.6, 76.3, 75.4, 75.4, 74.8, 74.6, 73.4, 73.3, 73.0, 72.9, 72.7, 72.4, 67.6, 59.1, 58.4, 56.0, 55.0, 55.0, 44.2 ppm.

HRMS (ESI) m/z: $[M+H]^+$ Calcd for $C_{63}H_{62}NO_8^+$ 960.4470; Found 960.4469.

((3a*R*,5a*S*,8a*S*,8b*R*)-2,2,7,7-tetramethyltetrahydro-3a*H*-bis([1,3]dioxolo)[4,5-b:4',5'-d]pyran-3a-yl)methyl 4-(4-methoxyphenyl)-3,3-diphenyl-2-(*p*-tolylamino)butanoate (4ra)

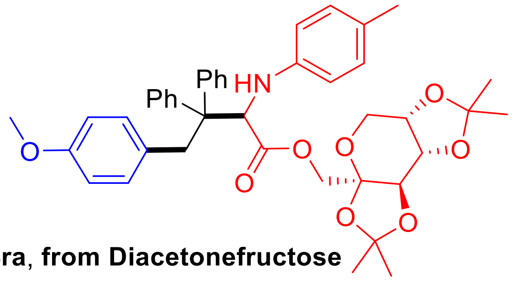

4ra, **from Diacetonefructose**

According to procedure G for the synthesis of conjugation of amino acid/peptide with aryl thianthrenium salt to afford **4ra** with two isomers as a white solid (49 mg, 47%

yield) (petro ether / ethyl acetate = 5:1, Rf = 0.4, m.p. 72 °C, d.r. = 1:1.3, d.r. is determined by ^{1}H NMR). The data is written as observed.

^{1}H NMR (400 MHz, $CDCl_3$) δ 7.37–7.20 (m, 8H), 7.14–7.12 (m, 1H),7.03–6.95 (m, 3H), 6.70–6.57 (m, 4H), 6.46–6.39 (m, 2H), 4.85 (d, J = 6.3 Hz, 1H), 4.48–4.22 (m, 1H), 4.17–4.01 (m, 2H), 3.93–3.77 (m, 3H), 3.75–3.74 (m, 3H), 3.66–3.25 (m, 4H), 2.22 (s, 3H), 1.46–1.30 (m, 9H), 0.97 (s, 3H) ppm.

^{13}C NMR (101 MHz, $CDCl_3$) δ 172.5, 172.4, 158.1, 158.0, 143.8, 143.6, 143.5, 143.4, 143.1, 132.2, 132.1, 130.2, 129.9, 129.7, 129.6, 129.5, 129.1, 128.9, 128.0, 127.7, 127.7, 127.6, 127.4, 126.8, 126.7, 126.5, 113.9, 113.2, 112.9, 112.7, 109.0, 108.8, 108.6, 108.3, 101.0, 100.8, 70.8, 70.7, 69.9, 69.7, 69.4, 66.1, 65.9, 61.2, 60.5, 60.0, 55.7, 55.5, 55.1, 55.0, 43.9, 43.3, 26.3, 26.3, 26.0, 25.9, 25.1, 24.8, 24.3, 24.1, 20.4, 20.3 ppm.

HRMS (ESI) m/z: $[M+H]^+$ Calcd for $C_{42}H_{48}NO_8^+$ 694.3374; Found 694.3365.

(3a*S*,5*S*,6*R*,6a*S*)-5-((R)-2,2-dimethyl-1,3-dioxolan-4-yl)-2,2-dimethyltetrahydrofuro[2,3-d][1,3]dioxol-6-yl 4-(4-methoxyphenyl)-3,3-diphenyl-2-(*p*-tolylamino)butanoate (4sa)

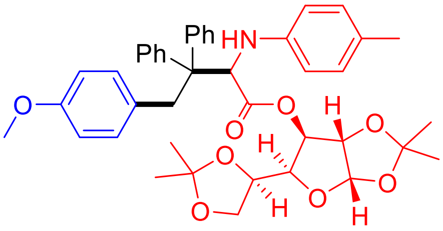

4sa, from Diacetone-D-glucose

According to procedure G for the synthesis of conjugation of amino acid/peptide with aryl thianthrenium salt to afford **4sa** with two isomers as a white solid (55 mg, 53% yield) (petro ether / ethyl acetate = 5:1, Rf = 0.4, m.p. 109 °C, d.r. = 1:1.5, d.r. is determined by ^{1}H NMR). The data is written as observed.

^{1}H NMR (400 MHz, $CDCl_3$) δ 7.36–7.16 (m, 9H), 7.05–6.97 (m, 3H), 6.67–6.57 (m, 4H), 6.51–6.45 (m, 2H), 5.30–4.88 (m, 3H), 4.04–3.89 (m, 2H), 3.82–3.26 (m, 9H), 2.22 (s, 3H), 1.43–1.41 (m, 3H), 1.35–1.33 (m, 3H), 1.30–1.22 (m, 3H), 1.14–1.06 (m, 3H) ppm.

^{13}C NMR (101 MHz, $CDCl_3$) δ 171.9, 171.1, 158.2, 158.2, 143.9, 143.9, 143.8, 143.6, 143.4, 142.9, 132.2, 132.0, 130.1, 129.8, 129.8, 129.7, 129.7, 129.6, 128.8, 128.6, 128.1, 127.8, 127.7, 127.3, 126.9, 126.8, 126.8, 126.6, 115.2, 114.0, 113.7, 113.0, 112.8, 111.9, 111.7, 109.1, 108.9, 104.8, 104.5, 82.6, 82.6, 78.6, 76.8, 76.3, 72.2, 71.3, 66.7, 65.8, 60.3, 59.2, 55.5, 55.1, 55.0, 44.1, 43.5, 26.8, 26.8, 26.5, 26.4, 26.0, 25.7, 25.3, 24.8, 20.3, 20.3 ppm.

HRMS (ESI) m/z: $[M+H]^+$ Calcd for $C_{42}H_{48}NO_8^+$ 694.3374; Found 694.3363.

ethyl (4-(4-methoxyphenyl)-3,3-diphenyl-2-(phenylamino)butanoyl)glycinate (4ta)

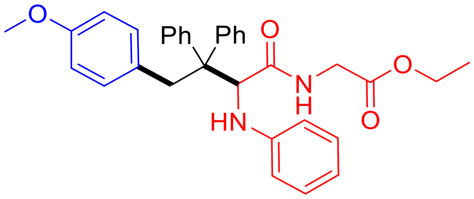

4ta, from Ph-Gly-Gly-OEt

According to procedure G for the synthesis of conjugation of amino acid/peptide with aryl thianthrenium salt to afford **4ta** as a yellow solid (41 mg, 53% yield) (DCM / MeOH = 20:1, Rf = 0.2, m.p. 142 °C).

^{1}H NMR (400 MHz, $CDCl_3$) δ 7.47–7.28 (m, 5H), 7.22–7.12 (m, 5H), 6.83–6.68 (m, 6H), 6.56–6.53 (m, 4H), 4.47 (d, J = 13.0 Hz, 1H), 4.38 (s, 1H), 4.17 (s, 1H), 4.08–4.03 (m, 2H), 3.92–3.86 (m, 1H), 3.70 (s, 3H), 3.38–3.33 (m, 1H), 2.94 (d, J = 12.8 Hz, 1H), 1.17 (t, J = 7.2 Hz, 3H) ppm.

^{13}C NMR (101 MHz, $CDCl_3$) δ 172.3, 168.8, 157.8, 146.7, 143.4, 141.8, 132.5, 130.4, 129.3, 129.0, 129.0, 127.8, 127.7, 127.2, 126.7, 119.5, 114.2, 112.5, 62.1, 61.1, 56.8, 55.0, 43.8, 41.2, 14.0 ppm.

HRMS (ESI) m/z: $[M+H]^+$ Calcd for $C_{33}H_{35}N_2O_4^+$ 523.2591; Found 523.2586.

benzyl (4-(4-methoxyphenyl)-3,3-diphenyl-2-(phenylamino)butanoyl)glycinate (4ua)

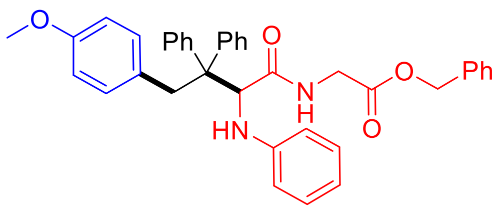

4ua, from Ph-Gly-Gly-OBn

According to procedure G for the synthesis of conjugation of amino acid/peptide with aryl thianthrenium salt to afford **4ua** as a yellowish solid (39 mg, 45% yield) (petro ether / ethyl acetate = 5:1, Rf = 0.5, m.p. 174 °C).

^{1}H NMR (400 MHz, $CDCl_3$) δ 7.46–7.44 (m, 2H), 7.35–7.31 (m, 5H), 7.27–7.23 (m, 3H), 7.21–7.13 (m, 5H), 6.82–6.69 (m, 6H), 6.56–6.54 (m, 4H), 5.10–5.01 (m, 2H), 4.47 (d, J = 12.8 Hz, 1H), 4.39 (s, 1H), 4.17 (s, 1H), 3.97–3.91 (m, 1H), 3.70 (s, 3H), 3.45–3.39 (m, 1H), 2.95 (d, J = 12.9 Hz, 1H) ppm.

^{13}C NMR (101 MHz, $CDCl_3$) δ 172.3, 168.6, 157.9, 146.7, 143.3, 141.8, 135.3, 132.6, 130.4, 129.3, 129.1, 129.0, 128.5, 128.4, 128.2, 127.8, 127.7, 127.2, 126.7, 119.6, 114.2, 112.6, 66.8, 62.1, 56.8, 55.0, 43.9, 41.3 ppm.

HRMS (ESI) m/z: $[M+H]^+$ Calcd for $C_{38}H_{37}N_2O_4^+$ 585.2748; Found 585.2742.

methyl (4-(4-methoxyphenyl)-3,3-diphenyl-2-(phenylamino)butanoyl)-L-phenylalaninate (4va)

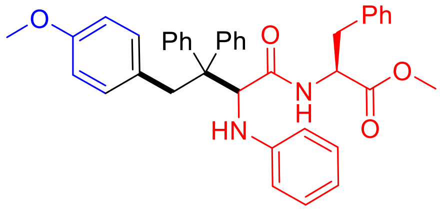

4va, from Ph-Gly-Phe-OMe

According to procedure G for the synthesis of conjugation of amino acid/peptide with aryl thianthrenium salt to afford **4va** with two isomers as a white solid (45 mg, 50% yield) (petro ether / ethyl acetate = 5:1, Rf = 0.5, m.p. 88 °C, d.r. = 1:1, d.r. is determined by ^{1}H NMR).

Isomer-1: ^{1}H NMR (400 MHz, $CDCl_3$) δ 7.42–7.40 (m, 2H), 7.32–7.28 (m, 2H), 7.24–7.17 (m, 4H), 7.14–7.10 (m, 2H), 7.07–7.04 (m, 1H), 6.98–6.95 (m, 2H), 6.87–6.83 (m, 1H), 6.79–6.69 (m, 7H), 6.57–6.52 (m, 4H), 4.66–4.61 (m, 1H), 4.46–4.43 (m, 2H), 4.17 (d, J = 2.7 Hz, 1H), 3.71 (s, 3H), 3.45 (s, 3H), 2.95 (d, J = 12.9 Hz, 1H), 2.92–2.87 (m, 1H), 2.82–2.77 (m, 1H) ppm.

^{13}C NMR (101 MHz, $CDCl_3$) δ 171.6, 170.3, 157.8, 146.5, 143.3, 142.0, 135.4, 132.5, 130.3, 129.4, 129.1, 129.1, 129.0, 128.1, 127.8, 127.7, 127.0, 126.6, 126.6, 119.4, 113.9, 112.6, 61.6, 56.5, 55.0, 53.2, 51.7, 44.0, 38.7 ppm.

HRMS (ESI) m/z: $[M+H]^+$ Calcd for $C_{39}H_{39}N_2O_4^+$ 599.2904; Found 599.2901.

Isomer-2: ^{1}H NMR (400 MHz, $CDCl_3$) δ 7.53–7.51 (m, 2H), 7.42–7.38 (m, 2H), 7.35–7.31 (m, 1H), 7.22–7.12 (m, 8H), 6.83–6.74 (m, 6H), 6.71–6.69 (m, 2H), 6.55–6.49 (m, 4H), 4.55 (d, J = 13.0 Hz, 1H), 4.52– 4.47 (m, 1H), 4.37 (d, J = 2.4 Hz, 1H), 4.14 (d, J = 2.4 Hz, 1H), 3.70 (s, 3H), 3.42 (s, 3H), 2.94 (d, J = 12.9 Hz, 1H), 2.64–2.60 (m, 1H), 2.35–2.29 (m, 1H) ppm.

^{13}C NMR (101 MHz, $CDCl_3$) δ 171.6, 171.2, 157.9, 146.6, 143.8, 142.1, 135.6, 132.6, 130.4, 129.3, 129.1, 129.0, 128.9, 128.4, 127.9, 127.7, 127.3, 126.9, 126.6, 119.6, 114.4, 112.6, 62.1, 56.7, 55.0, 52.9, 51.8, 44.1, 37.5 ppm.

HRMS (ESI) m/z: $[M+H]^+$ Calcd for $C_{39}H_{39}N_2O_4^+$ 599.2904; Found 599.2909.

ethyl (4-(4-methoxyphenyl)-3,3-diphenyl-2-(phenylamino)butanoyl)-L-leucinate (4wa)

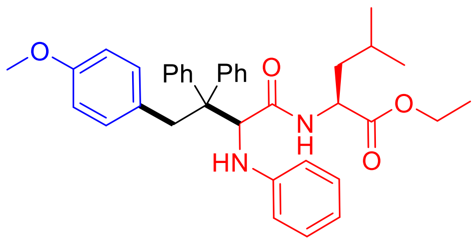

4wa, from Ph-Gly-Leu-OEt

According to procedure G for the synthesis of conjugation of amino acid/peptide with aryl thianthrenium salt to afford **4wa** with two isomers as a white solid (43 mg,

50% yield) (petro ether / ethyl acetate = 10:1, Rf = 0.4, m.p. 105 °C, d.r. = 1:1, d.r. is determined by ^{1}H NMR).

Isomer-1: ^{1}H NMR (400 MHz, $CDCl_3$) δ 7.45– 7.43 (m, 2H), 7.35–7.28 (m, 3H), 7.19–7.11 (m, 5H), 6.81–6.66 (m, 6H), 6.55–6.50 (m, 4H), 4.46–4.34 (m, 3H), 4.19–4.18 (m, 1H), 4.12–3.96 (m, 2H), 3.70 (s, 3H), 2.96 (d, *J* = 12.9 Hz, 1H), 1.34–1.26 (m, 3H), 1.23 (t, *J* = 7.1 Hz, 3H), 0.81 (d, *J* = 5.8 Hz, 3H), 0.77 (d, *J* = 6.0 Hz, 3H) ppm.

^{13}C NMR (101 MHz, $CDCl_3$) δ 171.3, 171.2, 157.8, 146.6, 143.3, 142.1, 132.5, 130.3, 129.2, 129.2, 129.1, 127.8, 127.7, 127.0, 126.6, 119.4, 113.9, 112.5, 61.9, 60.8, 56.6, 55.0, 50.5, 44.0, 42.1, 24.5, 22.4, 22.3, 14.1 ppm.

HRMS (ESI) m/z: $[M+H]^+$ Calcd for $C_{37}H_{43}N_2O_4^+$ 579.3217; Found 579.3226.

Isomer-2: ^{1}H NMR (400 MHz, $CDCl_3$) δ 7.49–7.47 (m, 2H), 7.39–7.31 (m, 3H), 7.20–7.16 (m, 3H), 7.13–7.10 (m, 2H), 6.84–6.80 (m, 1H), 6.77–6.74 (m, 2H), 6.70 (d, 1H), 6.65–6.64 (m, 2H), 6.58–6.52 (m, 4H), 4.54 (d, *J* = 12.9 Hz, 1H), 4.34 (s, 1H), 4.21–4.15 (m, 2H), 4.08–4.02 (m, 2H), 3.69 (s, 3H), 2.91 (d, *J* = 12.8 Hz, 1H), 1.20–1.11 (m, 4H), 1.07–0.97 (m, 1H), 0.94–0.87 (m, 1H), 0.78 (d, *J* = 6.5 Hz, 3H), 0.75 (d, *J* = 6.4 Hz, 3H) ppm.

^{13}C NMR (101 MHz, $CDCl_3$) δ 172.0, 171.8, 157.8, 147.0, 143.8, 142.2, 132.6, 130.4, 129.2, 129.2, 129.1, 128.0, 127.6, 127.1, 126.6, 119.7, 114.6, 112.5, 62.3, 60.9, 56.7, 55.0, 50.8, 44.1, 40.1, 24.2, 22.4, 22.3, 14.0 ppm.

HRMS (ESI) m/z: $[M+H]^+$ Calcd for $C_{37}H_{43}N_2O_4^+$ 579.3217; Found 579.3223.

methyl (4-(4-methoxyphenyl)-3,3-diphenyl-2-(phenylamino)butanoyl)-L-methioninate (4xa)

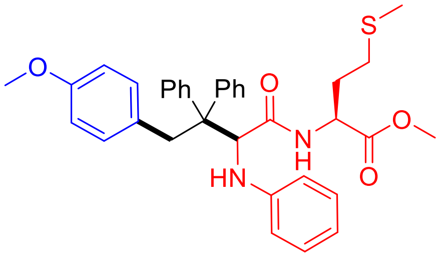

4xa, from Ph-Gly-Met-OMe

According to procedure G for the synthesis of conjugation of amino acid/peptide with aryl thianthrenium salt to afford **4xa** with two isomers as a yellowish solid (50 mg, 57% yield) (petro ether / ethyl acetate = 5:1, Rf = 0.4, m.p. 70 °C, d.r. = 1:1.3, d.r. is determined by ^{1}H NMR). The data is written as observed.

^{1}H NMR (400 MHz, $CDCl_3$) δ 7.50–7.29 (m, 5H), 7.21–7.10 (m, 5H), 6.96–6.65 (m, 6H), 6.57–6.51 (m, 4H), 4.54–4.37 (m, 3H), 4.23–4.21 (m, 1H), 3.70 (s, 3H), 3.60-3.55 (m, 3H), 2.96–2.90 (m, 1H), 2.16–2.09 (m, 1H), 1.99 (s, 1.7H), 1.96–1.89 (m, 1H), 1.89 (s, 1.3H), 1.77–1.65 (m, 1H), 1.63–1.43 (m, 1H) ppm.

^{13}C NMR (101 MHz, $CDCl_3$) δ 172.0, 171.6, 171.5, 170.8, 157.8, 146.7, 146.4, 143.8, 143.3, 142.1, 141.9, 132.6, 132.5, 130.3, 130.3, 129.3, 129.2, 129.0, 129.0, 129.0, 128.0, 127.8, 127.7, 127.7, 127.3, 127.0, 126.6, 119.7, 119.5, 114.5, 113.8, 112.5, 112.5, 62.1, 61.8, 56.6, 56.4, 55.0, 55.0, 52.1, 52.1, 51.2, 44.0, 44.0, 32.0, 30.8, 29.6, 29.2, 15.2, 15.2 ppm.

HRMS (ESI) m/z: $[M+H]^+$ Calcd for $C_{35}H_{39}N_2O_4S^+$ 583.2625; Found 583.2624.

methyl (4-(4-methoxyphenyl)-3,3-diphenyl-2-(phenylamino)butanoyl)-L-tyrosinate (4ya)

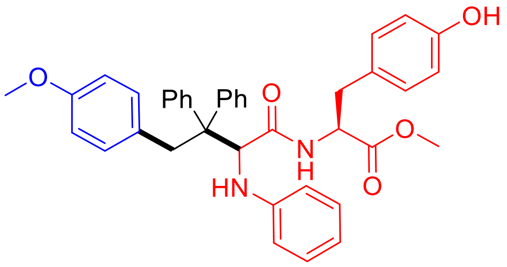

4ya, from Ph-Gly-Tyr-OMe

According to procedure G for the synthesis of conjugation of amino acid/peptide with aryl thianthrenium salt to afford **4ya** with two isomers as a white solid (43 mg, 47% yield) (petro ether / ethyl acetate = 2:1, Rf = 0.5, m.p. 110 °C, d.r. = 1:1, d.r. is determined by ^{1}H NMR). The data is written as observed.

^{1}H NMR (400 MHz, $CDCl_3$) δ 7.52–7.50 (m, 1H), 7.41–7.38 (m, 2H), 7.35–7.27 (m, 2H), 7.24–7.16 (m, 3H), 7.15–7.09 (m, 3H), 6.86–6.73 (m, 4H), 6.70–6.68 (m, 2H), 6.58 (s, 1H), 6.57–6.48 (m, 5H), 6.40–6.38 (m, 1H), 5.22–5.00 (m, 1H), 4.61–4.52 (m, 1H), 4.46–4.35 (m, 2H), 4.17–4.13 (m, 1H), 3.70–3.69 (m, 3H), 3.46–3.43 (m, 3H), 2.95–2.92 (m, 1H), 2.82–2.69 (m, 1H), 2.58–2.21 (m, 1H) ppm.

^{13}C NMR (101 MHz, $CDCl_3$) δ 171.8, 171.7, 171.4, 170.5, 157.8, 154.9, 154.6, 146.5, 146.4, 143.7, 143.2, 142.0, 132.5, 132.5, 130.3, 130.2, 130.0, 129.4, 129.3, 129.1, 129.1, 129.02, 129.0, 127.9, 127.8, 127.7, 127.7, 127.4, 127.1, 127.0, 127.0, 126.6, 126.6, 119.6, 119.4, 115.3, 115.1, 114.4, 113.9, 112.6, 112.6, 62.1, 61.7, 56.7, 56.5, 55.0, 55.0, 53.3, 53.1, 51.9, 51.8, 44.0, 37.9, 36.6 ppm.

HRMS (ESI) m/z: $[M+H]^+$ Calcd for $C_{39}H_{39}N_2O_5^+$ 615.2853; Found 615.2844.

methyl (4-(4-methoxyphenyl)-3,3-diphenyl-2-(phenylamino)butanoyl)-L-tryptophanate (4za)

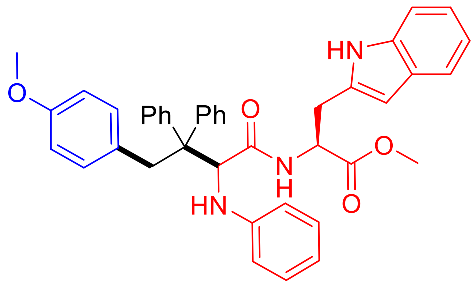

4za, from Ph-Gly-Trp-OMe

According to procedure G for the synthesis of conjugation of amino acid/peptide with aryl thianthrenium salt to afford **4za** with two isomers as a white solid (50 mg, 52% yield) (petro ether / ethyl acetate = 2:1, Rf = 0.5, m.p. 205 °C, d.r. = 1:1, d.r. is determined by ^{1}H NMR). The data is written as observed.

Isomer-1: ^{1}H NMR (400 MHz, $CDCl_3$) δ 7.62 (s, 1H), 7.42–7.41 (m, 2H), 7.35–7.27 (m, 3H), 7.25–7.23 (m, 1H), 7.20–7.14 (m, 5H), 7.12–7.06 (m, 2H), 7.01–6.97 (m, 1H), 6.84–6.80 (m, 3H), 6.76 (d, *J* = 7.7 Hz, 1H), 6.72 (d, *J* = 7.7 Hz, 2H), 6.57–6.55 (m, 2H), 6.53–6.51 (m, 2H), 6.17 (d, *J* = 2.4 Hz, 1H), 4.70–4.64 (m, 1H), 4.49–4.43 (m, 2H), 4.18 (d, *J* = 2.9 Hz, 1H), 3.71 (s, 3H), 3.37 (s, 3H), 3.15–3.10 (m, 1H), 2.99–2.94 (m, 2H) ppm.

^{13}C NMR (101 MHz, $CDCl_3$) δ 171.5, 170.8, 157.8, 146.5, 143.2, 142.1, 135.7, 132.5, 130.2, 129.4, 129.1, 129.1, 127.8, 127.6, 127.2, 127.0, 126.6, 123.1, 121.7, 119.2, 119.2, 118.4, 113.8, 112.6, 110.9, 109.2, 61.5, 56.4, 55.0, 52.5, 51.9, 44.0, 28.1 ppm.

HRMS (ESI) m/z: $[M+H]^+$ Calcd for $C_{41}H_{40}N_3O_4^+$ 638.3013; Found 638.3014.

Isomer-2: ^{1}H NMR (400 MHz, $CDCl_3$) δ 7.83 (s, 1H), 7.45–7.43 (m, 2H), 7.30–7.28 (m, 2H), 7.26–7.17 (m, 4H), 7.15–7.09 (m, 5H), 7.07–7.023 (m, 1H), 6.86 (d, *J* = 7.0 Hz, 1H), 6.77–6.69 (m, 5H), 6.54–6.51 (m, 3H), 6.44 (d, *J* = 7.9 Hz, 2H), 4.52 (d, *J* = 12.9 Hz, 1H), 4.45 (q, *J* = 6.8, 5.6 Hz, 1H), 4.36 (s, 1H), 4.02 (s, 1H), 3.69 (s, 3H), 3.48 (s, 3H), 2.93 (d, *J* = 12.8 Hz, 1H), 2.86–2.81 (m, 1H), 2.62–2.56 (m, 1H) ppm.

^{13}C NMR (101 MHz, $CDCl_3$) δ 171.8, 171.8, 157.8, 146.4, 143.6, 142.1, 135.9, 132.6, 130.4, 129.2, 129.1, 129.0, 127.7, 127.7, 127.2, 127.1, 126.6, 122.3, 122.1, 119.4, 118.5, 114.3, 112.5, 111.0, 109.7, 61.8, 56.8, 55.0, 52.6, 51.9, 44.1, 26.9 ppm.

HRMS (ESI) m/z: $[M+H]^+$ Calcd for $C_{41}H_{40}N_3O_4^+$ 638.3013; Found 638.3010.

methyl (4-(4-methoxyphenyl)-3,3-diphenyl-2-(phenylamino)butanoyl)glycylglycinate (4aaa)

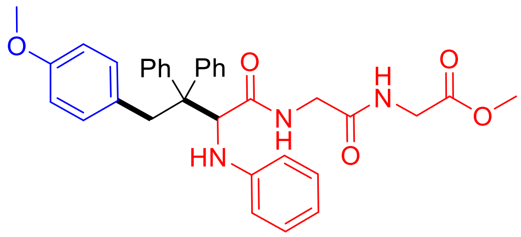

4aaa, from Ph-Gly-Gly-Gly-OMe

According to procedure G for the synthesis of conjugation of amino acid/peptide with aryl thianthrenium salt to afford **4aaa** as a white solid (40 mg, 47% yield) (petro ether / ethyl acetate = 3:1, Rf = 0.3, m.p. 184 °C).

^{1}H NMR (400 MHz, CDCl$_3$) δ 7.50–7.48 (m, 2H), 7.40–7.37 (m, 2H), 7.34–7.32 (m, 1H), 7.21–7.13 (m, 5H), 6.83–6.71 (m, 5H), 6.57–6.55 (m, 4H), 5.92 (t, *J* = 5.9 Hz, 1H), 4.49 (s, 1H), 4.40 (d, *J* = 12.9 Hz, 1H), 4.30 (s, 1H), 3.88–3.78 (m, 3H), 3.70 (s, 6H), 3.32–3.26 (m, 1H), 2.97 (d, *J* = 12.9 Hz, 1H) ppm.

^{13}C NMR (101 MHz, CDCl$_3$) δ 173.0, 169.6, 168.8, 157.9, 146.2, 143.5, 141.6, 132.5, 130.2, 129.5, 129.1, 128.7, 128.0, 127.8, 127.3, 126.8, 119.7, 113.7, 112.6, 61.7, 56.3, 55.0, 52.2, 43.8, 42.8, 40.7 ppm.

HRMS (ESI) m/z: [M+H]$^+$ Calcd for $C_{34}H_{36}N_3O_5^+$ 566.2649; Found 566.2645.

ethyl (4-(4-methoxyphenyl)-3,3-diphenyl-2-(phenylamino)butanoyl)-L-phenylalanylglycinate (4aba)

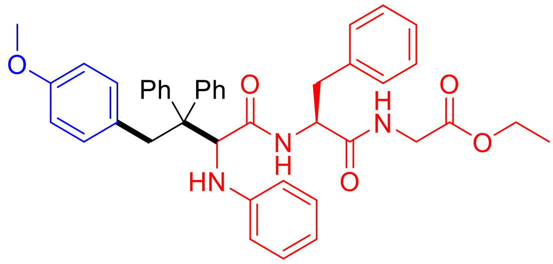

4aba, from Ph-Gly-Phe-Gly-OEt

According to procedure G for the synthesis of conjugation of amino acid/peptide with aryl thianthrenium salt to afford **4aba** with two isomers as a yellowish solid (36 mg, 36% yield) (petro ether / ethyl acetate = 3:1, Rf = 0.3, m.p. 98 °C, d.r. = 1:1, d.r. is determined by ^{1}H NMR). The data is written as observed.

^{1}H NMR (400 MHz, CDCl$_3$) δ 7.55–7.53 (m, 1H), 7.48–7.44 (m, 2H), 7.41–7.28 (m, 2H), 7.25–7.14 (m, 5H), 7.11–7.00 (m, 4H), 6.86–6.81 (m, 2H), 6.79–6.69 (m, 5H), 6.60–6.55 (m, 3H), 6.47–6.43 (m, 2H), 5.92–5.54 (m, 1H), 4.57–4.37 (m, 3H), 4.25–4.19 (m, 2H), 4.17–4.11 (m, 1H), 3.97—3.72 (m, 1H), 3.71–3.70 (m, 3H), 3.00–2.94

(m, 1.5H), 2.69–2.61 (m, 1H), 2.32–2.26 (m, 0.5H), 1.31 (t, *J* = 7.1 Hz, 1.5H), 1.24 (t, *J* = 7.1 Hz, 1.5H) ppm.

^{13}C NMR (101 MHz, $CDCl_3$) δ 172.5, 171.9, 170.3, 170.1, 169.2, 168.9, 157.9, 157.9, 146.6, 146.1, 143.8, 143.7, 142.1, 141.9, 135.9, 135.7, 132.6, 132.5, 130.2, 130.2, 129.5, 129.4, 129.4, 129.2, 129.2, 129.0, 128.9, 128.8, 128.4, 128.3, 128.1, 128.0, 127.7, 127.7, 127.5, 127.0, 126.8, 126.7, 126.7, 126.6, 119.7, 119.6, 113.9, 113.7, 112.6, 62.0, 61.4, 61.3, 61.2, 56.4, 56.2, 55.0, 53.9, 53.6, 44.1, 41.2, 41.0, 37.5, 36.5, 14.2, 14.1 ppm.

HRMS (ESI) m/z: $[M+H]^+$ Calcd for $C_{42}H_{44}N_3O_5^+$ 670.3275; Found 670.3273.

methyl (4-(4-methoxyphenyl)-3,3-diphenyl-2-(phenylamino)butanoyl)-L-phenylalanyl-L-tryptophanate (4aca)

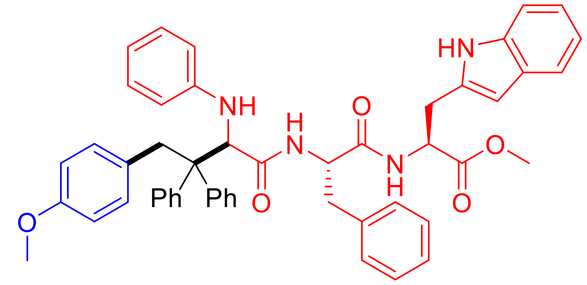

4aca, **from Ph-Gly-Phe-Trp-OMe**

According to procedure G for the synthesis of conjugation of amino acid/peptide with aryl thianthrenium salt to afford **4aca** with two isomers as a yellow solid (52 mg, 44% yield) (DCM / MeOH = 50:1, Rf = 0.3, m.p. 106 °C, d.r. = 1:1.3, d.r. is determined by ^{1}H NMR). The data is written as observed.

^{1}H NMR (400 MHz, $CDCl_3$) δ 8.25–8.21 (m, 0.56H), 7.72–7.68 (m, 0.44H), 7.65–7.54 (m, 1.5H), 7.47–7.34 (m, 2H), 7.28–7.26 (m, 1H), 7.22–7.14 (m, 4H), 7.13–7.02 (m, 9H), 7.00–6.94 (m, 2H), 6.88–6.87 (m, 1H), 6.81–6.77 (m, 3H), 6.71–6.69 (m, 1.5H), 6.64–6.57 (m, 3H), 6.47–6.39 (m, 2H), 6.18–6.06 (m, 1H), 4.71–4.42 (m, 2H), 4.34–3.99 (m, 3H), 3.73 (s, 1.7H), 3.71 (s, 1.3H), 3.65 (s, 1.7H), 3.57 (s, 1.3H), 3.34–2.98 (m, 2.5H), 2.76–2.61 (m, 2H), 2.08–2.02 (m, 0.5H) ppm.

^{13}C NMR (101 MHz, $CDCl_3$) δ 172.4, 171.8, 171.8, 171.4, 169.5, 169.5, 169.1, 157.8, 157.8, 146.4, 146.4, 143.6, 143.1, 141.9, 141.8, 136.2, 135.9, 135.8, 135.7, 132.5,

132.4, 130.3, 130.0, 129.5, 129.3, 129.1, 129.1, 128.9, 128.6, 128.3, 128.2, 127.7, 127.6, 127.5, 127.1, 127.1, 126.9, 126.7, 126.7, 126.6, 126.5, 123.2, 122.7, 122.3, 121.8, 119.8, 119.5, 119.3, 119.3, 118.3, 118.3, 113.7, 113.6, 112.5, 111.5, 111.1, 109.7, 109.3, 61.8, 61.5, 56.6, 56.0, 55.0, 54.2, 53.9, 53.1, 52.4, 52.2, 52.1, 44.0, 43.5, 37.6, 36.8, 27.7, 27.5 ppm.

HRMS (ESI) m/z: $[M+H]^+$ Calcd for $C_{50}H_{49}N_4O_5^+$ 785.3697; Found 785.3704.

methyl (4-(4-methoxyphenyl)-3,3-diphenyl-2-(phenylamino)butanoyl)-L-phenylalanylglycylglycinate (4ada)

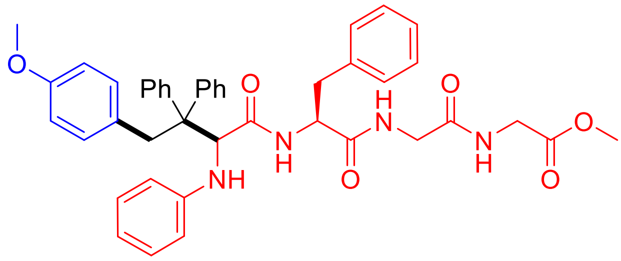

4ada, from Ph-Gly-Phe-Gly-Gly-OMe

According to procedure G for the synthesis of conjugation of amino acid/peptide with aryl thianthrenium salt to afford **4ada** with two isomers as a yellowish solid (48 mg, 45% yield) (petro ether / ethyl acetate = 1:2, Rf = 0.3, m.p. 112 °C, d.r. = 1:1.5, d.r. is determined by ^{1}H NMR). The data is written as observed.

^{1}H NMR (400 MHz, $CDCl_3$) δ 7.57–7.30 (m, 5H), 7.22–6.92 (m, 10H), 6.86–6.62 (m, 6H), 6.56–6.45 (m, 4H), 6.38 (d, J = 8.0 Hz, 1H), 5.69–5.31 (s, 1H), 4.55–4.39 (m, 1H), 4.31–3.88 (m, 6H), 3.74–3.63 (m, 6H), 3.59–2.64 (m, 3H), 2.24–1.16 (m, 1H) ppm.

^{13}C NMR (101 MHz, $CDCl_3$) δ 174.0, 173.2, 170.7, 170.6, 169.9, 169.9, 169.2, 169.2, 158.0, 157.9, 146,3, 145.4, 144.1, 143.6, 141.8, 141.5, 135.0, 134.9, 132.6, 132.3, 130.0, 129.5, 129.4, 129.2, 129.0, 128.8, 128.7, 128.5, 128.5, 128.5, 128.1, 128.0, 127.8, 127.7, 127.5, 127.5, 127.1, 127.0, 126.8, 126.8, 120.0, 120.0, 113.8, 113.7, 112.6, 112.5, 62.0, 60.8, 55.9, 55.8, 55.1, 54.9, 54.9, 54.5, 52.2, 52.1, 43.9, 43.8, 42.8, 42.4, 40.9, 40.9, 37.1, 36,0 ppm.

HRMS (ESI) m/z: $[M+H]^+$ Calcd for $C_{43}H_{45}N_4O_6^+$ 713.3334; Found 713.3338.

ethyl 4-(4-(*tert*-butyl)phenyl)-3,3-diphenyl-2-(phenylamino)butanoate (5ab)

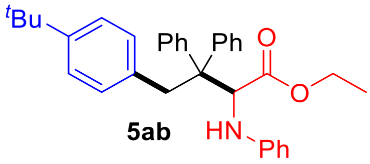

5ab

According to procedure G for the synthesis of conjugation of amino acid/peptide with aryl thianthrenium salt to afford **5ab** as a yellowish solid (52 mg, 70% yield) (petro ether / ethyl acetate = 50:1, Rf = 0.3, m.p. 138 °C).

^{1}H NMR (400 MHz, $CDCl_3$) δ 7.34–7.28 (m, 5H), 7.23–7.20 (m, 3H), 7.16–7.06 (m, 6H), 6.75–6.71 (m, 1H), 6.67–6.65 (m, 2H), 6.53–6.50 (m, 2H), 4.89 (d, *J* = 11.1 Hz, 1H), 4.09 (d, *J* = 11.1 Hz, 1H), 3.90–3.75 (m, 2H), 3.74–3.60 (m, 2H), 1.27 (s, 9H), 0.87 (t, *J* = 7.1 Hz, 3H) ppm.

^{13}C NMR (101 MHz, $CDCl_3$) δ 172.3, 149.2, 146.5, 143.8, 143.5, 134.0, 130.7, 129.8, 129.6, 129.2, 127.6, 127.3, 126.8, 126.6, 124.4, 118.4, 113.8, 60.6, 60.1, 55.3, 43.8, 34.3, 31.3, 13.7 ppm.

HRMS (ESI) m/z: $[M+H]^+$ Calcd for $C_{34}H_{38}NO_2^+$ 492.2897; Found 492.2892.

ethyl 4-(4-cyclohexylphenyl)-3,3-diphenyl-2-(phenylamino)butanoate (5ac)

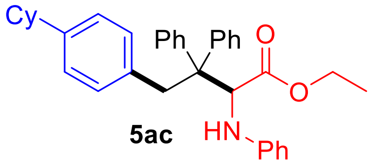

5ac

According to procedure G for the synthesis of conjugation of amino acid/peptide with aryl thianthrenium salt to afford **5ac** as a yellowish solid (52 mg, 68% yield) (petro ether / ethyl acetate = 50:1, Rf = 0.3, m.p. 110 °C).

^{1}H NMR (400 MHz, $CDCl_3$) δ 7.35–7.29 (m, 5H), 7.24–7.21 (m, 3H), 7.19–7.10 (m, 4H), 6.91 (d, *J* = 7.8 Hz, 2H), 6.74 (t, *J* = 7.4 Hz, 1H), 6.65 (d, *J* = 7.7 Hz, 2H), 6.53 (d, *J* = 8.0 Hz, 2H), 4.90 (d, *J* = 11.1 Hz, 1H), 4.10 (d, *J* = 11.2 Hz, 1H), 3.91–3.73

(m, 2H), 3.73–3.60 (m, 2H), 2.46–2.38 (m, 1H), 1.85–1.83 (m, 4H), 1.76–1.73 (m, 1H), 1.40–1.35 (m, 4H), 1.28–1.25 (m, 1H), 0.88 (t, *J* = 7.1 Hz, 3H) ppm.

^{13}C NMR (101 MHz, $CDCl_3$) δ 172.3, 146.5, 146.1, 143.8, 143.5, 134.4, 130.9, 129.9, 129.6, 129.2, 127.6, 127.3, 126.8, 126.6, 125.9, 118.5, 113.8, 60.6, 60.0, 55.4, 44.1, 44.0, 34.5, 34.4, 26.9, 26.2, 13.7 ppm.

HRMS (ESI) m/z: $[M+H]^+$ Calcd for $C_{36}H_{40}NO_2^+$ 518.3054; Found 518.3053.

ethyl 4-(4-(3-chloropropyl)phenyl)-3,3-diphenyl-2-(phenylamino)butanoate (5ad)

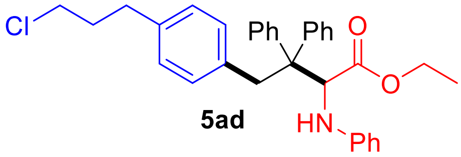

5ad

According to procedure G for the synthesis of conjugation of amino acid/peptide with aryl thianthrenium salt to afford **5ad** as a yellowish solid (51 mg, 67% yield) (petro ether / ethyl acetate = 20:1, Rf = 0.4, m.p. 105 °C).

^{1}H NMR (400 MHz, $CDCl_3$) δ 7.37–7.32 (m, 5H), 7.25–7.22 (m, 3H), 7.20–7.16 (m, 2H), 7.13–7.11 (m, 2H), 6.92 (d, *J* = 7.8 Hz, 2H), 6.78–6.75 (m, 1H), 6.70 (d, *J* = 7.7 Hz, 2H), 6.58 (d, *J* = 7.9 Hz, 2H), 4.92 (d, *J* = 11.4 Hz, 1H), 4.12 (d, *J* = 11.3 Hz, 1H), 3.95–3.78 (m, 2H), 3.72–3.65 (m, 2H), 3.51 (t, *J* = 6.5 Hz, 2H), 2.72 (t, *J* = 7.4 Hz, 2H), 2.06 (p, *J* = 6.9 Hz, 2H), 0.90 (t, *J* = 7.2 Hz, 3H) ppm.

^{13}C NMR (101 MHz, $CDCl_3$) δ 172.2, 146.5, 143.6, 143.2, 138.6, 134.9, 131.1, 129.9, 129.5, 129.2, 127.6, 127.6, 127.3, 126.8, 126.6, 118.6, 113.9, 60.6, 59.6, 55.4, 44.2, 44.1, 33.9, 32.2, 13.6 ppm.

HRMS (ESI) m/z: $[M+H]^+$ Calcd for $C_{33}H_{35}ClNO_2^+$ 512.2351; Found 512.2353.

ethyl 4-([1,1'-biphenyl]-4-yl)-3,3-diphenyl-2-(phenylamino)butanoate (5ae)

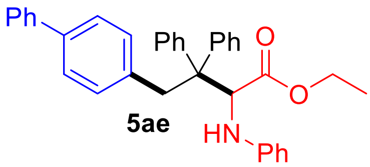

5ae

According to procedure G for the synthesis of conjugation of amino acid/peptide with aryl thianthrenium salt to afford **5ae** as a white solid (46 mg, 60% yield) (petro ether / ethyl acetate = 20:1, Rf = 0.4, m.p. 112 °C).

Replace $^{-}$OTf with $^{-}BF_4$: To a 100 mL flame-dried round-bottom flask, **2a** (5.0 mmol, 1.0 equiv.), and MeCN (25 mL, 0.2 M.), followed by a slow drip of saturated $NaBF_4$ aqueous solution. The reaction mixture was stirred for 8 h, and extracted with ethyl acetate (20 mL X 3). The combined organic layer was dried on anhydrous Na_2SO_4 and condensed under reduced pressure. The residue could be employed in procedure G for the synthesis of conjugation of amino acid/peptide with aryl thianthrenium salt to afford **5ae** as a white solid (47 mg, 61% yield).

^{1}H NMR (400 MHz, $CDCl_3$) δ 7.59–7.56 (m, 2H), 7.45–7.41 (m, 2H), 7.39–7.31 (m, 8H), 7.27– 7.23 (m, 3H), 7.19–7.13 (m, 4H), 6.81 (d, *J* = 7.9 Hz, 2H), 6.76 (t, *J* = 7.3 Hz, 1H), 6.59 (d, *J* = 8.0 Hz, 2H), 4.93 (d, *J* = 11.4 Hz, 1H), 4.12 (d, *J* = 11.4 Hz, 1H), 3.94–3.86 (m, 1H), 3.85–3.79 (m, 1H), 3.78–3.68 (m, 2H), 0.89 (t, *J* = 7.1 Hz, 3H) ppm.

^{13}C NMR (101 MHz, $CDCl_3$) δ 172.2, 146.5, 143.6, 143.2, 140.8, 139.0, 136.3, 131.5, 130.0, 129.6, 129.2, 128.7, 127.6, 127.4, 127.1, 126.9, 126.9, 126.7, 126.0, 118.7, 114.0, 60.7, 59.6, 55.6, 44.3, 13.7 ppm.

HRMS (ESI) m/z: $[M+H]^+$ Calcd for $C_{36}H_{34}NO_2^+$ 512.2584; Found 512.2577.

ethyl 4-(dibenzo[b,d]furan-3-yl)-3,3-diphenyl-2-(phenylamino)butanoate (5af)

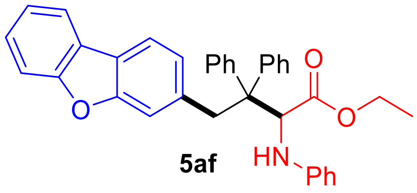

5af

According to procedure G for the synthesis of conjugation of amino acid/peptide with aryl thianthrenium salt to afford **5af** as a white solid (55 mg, 70% yield) (petro ether / ethyl acetate = 20:1, Rf = 0.4, m.p. 107 °C).

^{1}H NMR (400 MHz, $CDCl_3$) δ 7.64 (d, *J* = 7.6 Hz, 1H), 7.55–7.53 (m, 1H), 7.43 (t, *J* = 7.8 Hz, 1H), 7.36–7.25 (m, 10H), 7.23–7.14 (m, 5H), 6.79–6.72 (m, 2H), 6.60–6.56 (m, 2H), 4.90 (d, *J* = 11.6 Hz, 1H), 4.11 (d, *J* = 11.7 Hz, 1H), 4.02–3.93 (m, 1H), 3.91–3.87 (m, 1H), 3.85–3.78 (m, 1H), 3.69 – 3.61 (m, 1H), 0.88 (t, *J* = 7.0 Hz, 3H) ppm.

^{13}C NMR (101 MHz, $CDCl_3$) δ 172.2, 156.3, 155.1, 146.5, 143.4, 143.1, 131.3, 130.3, 130.1, 129.8, 129.3, 127.5, 127.5, 126.9, 126.9, 126.8, 124.3, 123.5, 123.1, 122.5, 120.5, 118.8, 114.1, 111.5, 110.3, 60.7, 59.0, 56.2, 44.6, 13.7 ppm.

HRMS (ESI) m/z: $[M+H]^+$ Calcd for $C_{36}H_{32}NO_3^+$ 526.2377; Found 526.2371.

ethyl 4-(4-fluorophenyl)-3,3-diphenyl-2-(phenylamino)butanoate (5ag)

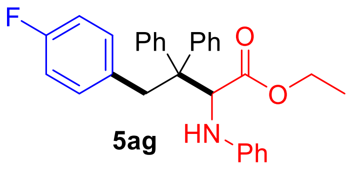

5ag

According to procedure G for the synthesis of conjugation of amino acid/peptide with aryl thianthrenium salt to afford **5ag** as a white solid (54 mg, 66% yield) (petro ether / ethyl acetate = 50:1, Rf = 0.3, m.p. 122 °C).

^{1}H NMR (400 MHz, $CDCl_3$) δ 7.35–7.31 (m, 5H), 7.25–7.21 (m, 2H), 7.19–7.15 (m, 2H), 7.09–7.07 (m, 2H), 6.78–6.74 (m, 3H), 6.71–6.67 (m, 2H), 6.57 (d, *J* = 7.9 Hz, 2H), 4.84 (d, *J* = 11.5 Hz, 1H), 4.07 (d, *J* = 11.5 Hz, 1H), 3.93–3.75 (m, 2H), 3.68–3.59 (m, 2H), 0.87 (t, *J* = 7.1 Hz, 3H) ppm.

^{13}C NMR (101 MHz, $CDCl_3$) δ 172.1, 161.6 (d, $J_{C\text{-}F}$ = 245.6 Hz), 146.5, 143.4, 142.9, 132.7 (d, $J_{C\text{-}F}$ = 3.0 Hz), 132.5 (d, $J_{C\text{-}F}$ = 8.1 Hz), 129.9, 129.5, 129.3, 127.7, 127.4, 127.0, 126.7, 118.8, 114.2 (d, $J_{C\text{-}F}$ = 21.2 Hz), 114.0, 60.6, 59.3, 55.5, 43.8, 13.6 ppm.

^{19}F NMR (376 MHz, $CDCl_3$) δ -116.74 ppm.

HRMS (ESI) m/z: $[M+H]^+$ Calcd for $C_{30}H_{29}FNO_2^+$ 454.2177; Found 454.2175.

ethyl 4-(4-bromophenyl)-3,3-diphenyl-2-(phenylamino)butanoate (5ah)

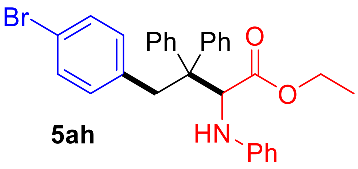

5ah

According to procedure G for the synthesis of conjugation of amino acid/peptide with aryl thianthrenium salt to afford **5ah** as a white solid (50 mg, 65% yield) (petro ether / ethyl acetate = 20:1, Rf = 0.4, m.p. 121 °C).

^{1}H NMR (400 MHz, $CDCl_3$) δ 7.35–7.31 (m, 5H), 7.25–7.22 (m, 3H), 7.21–7.16 (m, 4H), 7.10–7.07 (m, 2H), 6.77 (t, *J* = 7.3 Hz, 1H), 6.61–6.56 (m, 4H), 4.82 (d, *J* = 11.6 Hz, 1H), 4.05 (d, *J* = 11.6 Hz, 1H), 3.93–3.85 (m, 1H), 3.83–3.75 (m, 1H), 3.68–3.60 (m, 2H), 0.87 (t, *J* = 7.1 Hz, 3H) ppm.

^{13}C NMR (101 MHz, $CDCl_3$) δ 172.1, 146.4, 143.2, 142.7, 136.0, 132.8, 130.5, 129.9, 129.4, 129.3, 127.7, 127.5, 127.1, 126.8, 120.5, 118.8, 114.0, 60.8, 59.2, 55.5, 44.1, 13.6 ppm.

HRMS (ESI) m/z: $[M+H]^+$ Calcd for $C_{30}H_{29}BrNO_2^+$ 514.1376; Found 514.1379.

ethyl 4-(4-iodophenyl)-3,3-diphenyl-2-(phenylamino)butanoate (5ai)

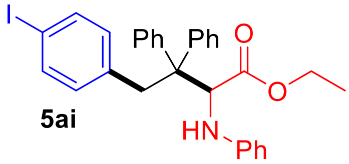

5ai

According to procedure G for the synthesis of conjugation of amino acid/peptide with aryl thianthrenium salt to afford **5ai** as a white solid (51 mg, 61% yield) (petro ether / ethyl acetate = 50:1, Rf = 0.3, m.p. 124 °C).

^{1}H NMR (400 MHz, $CDCl_3$) δ 7.39–7.37 (m, 2H), 7.34–7.20 (m, 5H), 7.24–7.21 (m, 3H), 7.19–7.15 (m, 2H), 7.09–7.07 (m, 2H), 6.78–6.74 (m, 1H), 6.56 (d, *J* = 7.4 Hz, 2H), 6.46 (d, *J* = 8.3 Hz, 2H), 4.81 (d, *J* = 11.6 Hz, 1H), 4.04 (d, *J* = 11.6 Hz, 1H), 3.92–3.74 (m, 2H), 3.59 (s, 2H), 0.86 (t, *J* = 7.2 Hz, 3H) ppm.

^{13}C NMR (101 MHz, $CDCl_3$) δ 172.1, 146.4, 143.2, 142.7, 136.7, 136.5, 133.2, 129.9, 129.5, 129.3, 127.7, 127.5, 127.1, 126.8, 118.8, 114.0, 92.2, 60.8, 59.2, 55.5, 44.3, 13.6 ppm.

HRMS (ESI) m/z: $[M+H]^+$ Calcd for $C_{30}H_{29}INO_2^+$ 562.1237; Found 562.1233.

Ethyl 4-(4-(2-oxopyrrolidin-1-yl)phenyl)-3,3-diphenyl-2-(phenylamino)butanoate (5aj)

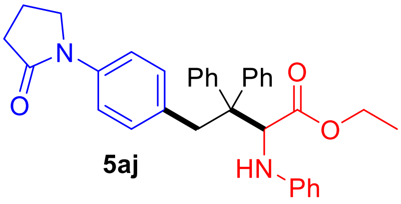

5aj

According to procedure G for the synthesis of conjugation of amino acid/peptide with aryl thianthrenium salt to afford **5aj** as a yellowish oil (54 mg, 70% yield) (petro ether / ethyl acetate = 3:1, Rf = 0.3).

^{1}H NMR (400 MHz, $CDCl_3$) δ 7.36–7.30 (m, 7H), 7.24–7.20 (m, 3H), 7.17–7.02 (m, 4H), 6.76–6.70 (m, 3H), 6.58–6.56 (m, 2H), 4.87 (d, *J* = 11.4 Hz, 1H), 4.07 (d, *J* = 11.4 Hz, 1H), 3.91–3.78 (m, 4H), 3.69–3.59 (m, 2H), 2.59 (t, *J* = 8.1 Hz, 2H), 2.12 (d, *J* = 7.6 Hz, 2H), 0.88 (t, *J* = 7.1 Hz, 3H) ppm.

^{13}C NMR (101 MHz, $CDCl_3$) δ 174.0, 172.1, 146.5, 143.4, 143.1, 137.8, 133.0, 131.4, 129.9, 129.5, 129.2, 127.6, 127.3, 126.9, 126.6, 118.6, 118.5, 114.0, 60.7, 59.4, 55.5, 48.6, 44.1, 32.8, 17.9, 13.6 ppm.

HRMS (ESI) m/z: $[M+H]^+$ Calcd for $C_{34}H_{35}N_2O_3^+$ 519.2642; Found 519.2644.

ethyl 4-(1-(2,6-dichlorophenyl)-2-oxoindolin-5-yl)-3,3-diphenyl-2-(phenylamino)butanoate (5ak)

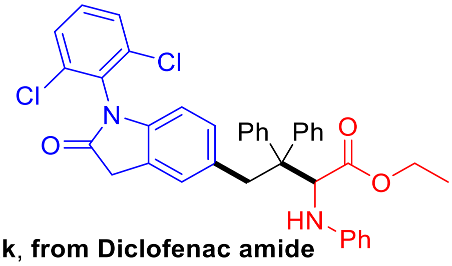

5ak, from Diclofenac amide

According to procedure G for the synthesis of conjugation of amino acid/peptide with aryl thianthrenium salt to afford **5ak** as a yellow solid (64 mg, 67% yield) (petro ether / ethyl acetate = 5:1, Rf = 0.3, m.p. 96 °C).

^{1}H NMR (400 MHz, $CDCl_3$) δ 7.51–7.48 (m, 2H), 7.38–7.29 (m, 6H), 7.24–7.22 (m, 3H), 7.17–7.10 (m, 4H), 6.76–6.72 (m, 1H), 6.61–6.58 (m, 2H), 6.55–6.53 (m, 2H), 6.12 (d, *J* = 7.9 Hz, 1H), 4.86 (d, *J* = 11.3 Hz, 1H), 4.09 (d, *J* = 11.3 Hz, 1H), 3.90–3.82 (m, 1H), 3.81–3.73 (m, 1H), 3.72–3.59 (m, 2H), 3.55 (s, 2H), 0.85 (t, *J* = 7.1 Hz, 3H) ppm.

^{13}C NMR (101 MHz, $CDCl_3$) δ 173.7, 172.2, 146.5, 143.4, 143.1, 141.7, 135.4, 135.4, 131.9, 130.6, 130.5, 130.3, 129.9, 129.6, 129.2, 129.0, 127.6, 127.5, 127.4, 126.9, 126.7, 123.3, 118.7, 113.8, 108.1, 60.7, 59.6, 55.7, 44.2, 35.6, 13.6 ppm.

HRMS (ESI) m/z: $[M+H]^+$ Calcd for $C_{38}H_{33}Cl_2N_2O_3^+$ 635.1863; Found 635.1858.

ethyl 4-(2-(2-methoxy-2-oxoethyl)-11-oxo-6,11-dihydrodibenzo[b,e]oxepin-9-yl)-3,3-diphenyl-2-(phenylamino)butanoate (5al)

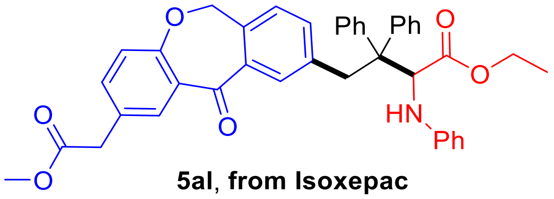

5al, from Isoxepac

According to procedure G for the synthesis of conjugation of amino acid/peptide with aryl thianthrenium salt to afford **5al** as a yellowish solid (35 mg, 36% yield) (petro ether / ethyl acetate = 5:1, Rf = 0.3, m.p. 127 °C).

^{1}H NMR (400 MHz, $CDCl_3$) δ 7.93 (d, *J* = 2.5 Hz, 1H), 7.76–7.73 (m, 1H), 7.50–7.39 (m, 3H), 7.28–7.26 (m, 2H), 7.21–7.17 (m, 2H), 7.15–7.10 (m, 8H), 7.07–7.01 (m, 1H), 6.75–6.71 (m, 1H), 6.63–6.60 (m, 2H), 5.11 (d, *J* = 11.6 Hz, 1H), 4.45–4.37 (m, 2H), 4.03 (d, *J* = 11.6 Hz, 1H), 3.94 (d, *J* = 12.9 Hz, 1H), 3.89–3.81 (m, 1H), 3.80–3.72 (m, 1H), 3.71 (s, 3H), 3.65 (d, *J* = 12.9 Hz, 1H), 3.53–3.51 (m, 2H), 0.84 (t, *J* = 7.1 Hz, 3H) ppm.

^{13}C NMR (101 MHz, $CDCl_3$) δ 191.4, 172.1, 171.9, 159.2, 146.6, 142.7, 142.0, 140.4, 139.0, 135.3, 132.2, 131.1, 129.9, 129.6, 129.4, 129.2, 129.0, 128.9, 127.4, 127.0, 126.9, 126.8, 126.6, 126.4, 124.5, 118.7, 114.1, 72.8, 60.7, 59.4, 55.7, 52.0, 40.0, 36.9, 13.6 ppm.

HRMS (ESI) m/z: $[M+H]^+$ Calcd for $C_{41}H_{38}NO_6^+$ 640.2694; Found 640.2688.

methyl 5-(4-(4-ethoxy-4-oxo-2,2-diphenyl-3-(phenylamino)butyl)-2,5-dimethylphenoxy)-2,2-dimethylpentanoate (5am)

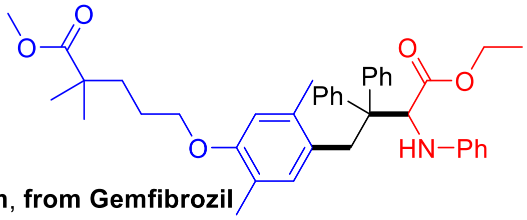

5am, from Gemfibrozil

According to procedure G for the synthesis of conjugation of amino acid/peptide with aryl thianthrenium salt to afford **5am** as a white solid (48 mg, 51% yield) (petro ether / ethyl acetate = 20:1, Rf = 0.3, m.p. 101 °C).

^{1}H NMR (400 MHz, $CDCl_3$) δ 7.32–7.27 (m, 5H), 7.25–7.14 (m, 7H), 6.90 (s, 1H), 6.75 (t, J = 7.4 Hz, 1H), 6.60–6.58 (m, 2H), 6.35 (s, 1H), 5.06 (d, J = 11.3 Hz, 1H), 4.03 (d, J = 11.3 Hz, 1H), 3.96 (d, J = 13.2 Hz, 1H), 3.90–3.86 (m, 2H), 3.85–3.74 (m, 2H), 3.68 (s, 3H), 3.21 (d, J = 13.1 Hz, 1H), 2.03 (s, 3H), 1.74–1.72 (m, 4H), 1.28 (s, 3H), 1.23 (s, 6H), 0.85 (t, J = 7.1 Hz, 3H) ppm.

^{13}C NMR (101 MHz, $CDCl_3$) δ 178.3, 172.3, 155.4, 146.6, 142.6, 142.3, 137.7, 133.8, 130.3, 130.2, 129.2, 127.3, 127.0, 127.0, 126.8, 126.6, 123.1, 118.6, 67.8, 60.5, 59.6, 56.0, 51.7, 42.1, 39.5, 37.1, 25.2, 18.5, 15.5, 13.6 ppm.

HRMS (ESI) m/z: $[M+H]^+$ Calcd for $C_{40}H_{48}NO_5^+$ 622.3527; Found 622.3522.

ethyl 4-(2'-fluoro-4'-(1-methoxy-1-oxopropan-2-yl)-[1,1'-biphenyl]-4-yl)-3,3-diphenyl-2-(phenylamino)butanoate (5an)

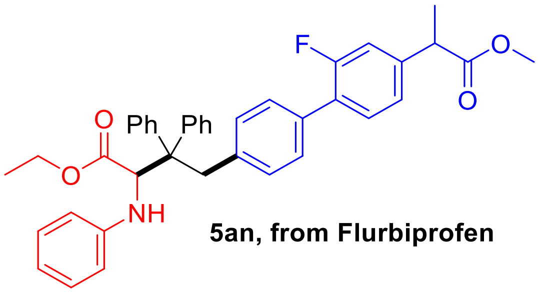

5an, from Flurbiprofen

According to procedure G for the synthesis of conjugation of amino acid/peptide with aryl thianthrenium salt to afford **5an** as a white solid (60 mg, 65% yield) (petro ether / ethyl acetate = 20:1, Rf = 0.3, m.p. 98 °C, d.r. was not able to be determined by NMR spectra).

^{1}H NMR (400 MHz, $CDCl_3$) δ 7.29–7.32 (m, 6H), 7.27–7.23 (m, 5H), 7.18–7.09 (m, 6H), 6.81–6.73 (m, 3H), 6.58 (d, J = 8.0 Hz, 2H), 4.93 (s, 1H), 3.94–3.74 (m, 5H), 3.71 (s, 3H), 1.54 (d, J = 7.2 Hz, 3H), 0.89 (t, J = 6.8 Hz, 3H) ppm.

^{13}C NMR (101 MHz, $CDCl_3$) δ 174.4, 172.2, 159.7 (d, $J_{C\text{-}F}$ = 249.5 Hz), 146.5, 143.5, 143.1, 141.5 (d, $J_{C\text{-}F}$ = 7.6 Hz), 136.7, 133.4, 131.2, 130.7 (d, $J_{C\text{-}F}$ = 3.9 Hz), 130.64, 129.9, 129.5, 129.2, 127.9 (d, $J_{C\text{-}F}$ = 3.2 Hz), 127.6, 127.4, 126.9, 126.7, 123.4 (d, $J_{C\text{-}F}$ = 3.4 Hz), 118.6, 115.2 (d, $J_{C\text{-}F}$ = 23.8 Hz), 114.0, 60.7, 59.6, 55.5, 52.2, 44.9, 44.3, 18.4, 13.6 ppm.

^{19}F NMR (376 MHz, $CDCl_3$) δ -117.3 ppm.

HRMS (ESI) m/z: [M+H$^+$ Calcd for $C_{44}H_{39}FNO_4^+$ 616.2858; Found 616.2861.

ethyl 4-(2-ethoxy-4-(2-methyl-1-((3-phenoxybenzyl)oxy)propan-2-yl)phenyl)-3,3-diphenyl-2-(phenylamino)butanoate (5ao)

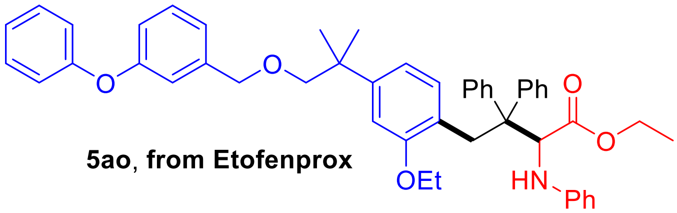

5ao, from Etofenprox

According to procedure G for the synthesis of conjugation of amino acid/peptide with aryl thianthrenium salt to afford **5ao** as a white solid (63 mg, 57% yield) (petro ether / ethyl acetate = 10:1, Rf = 0.4).

^{1}H NMR (400 MHz, $CDCl_3$) δ 7.37–7.32 (m, 4H), 7.30–7.25 (m, 4H), 7.23–7.21 (m, 1H), 7.15–7.00 (m, 12H), 6.97–6.95 (m, 1H), 6.93–6.90 (m, 1H), 6.71 (t, *J* = 7.3 Hz, 1H), 6.60 (d, *J* = 7.9 Hz, 2H), 6.46 (d, *J* = 8.6 Hz, 1H), 5.14 (d, *J* = 11.0 Hz, 1H), 4.42 (s, 2H), 4.31–4.28 (m, 1H), 3.97–3.93 (m, 1H), 3.88–3.80 (m, 2H), 3.66 (d, *J* = 13.3 Hz, 1H), 3.57–3.41 (m, 2H), 3.31 (s, 2H), 1.23 (s, 3H), 1.22 (s, 3H), 1.16 (t, *J* = 7.0 Hz, 3H), 0.90 (t, *J* = 7.1 Hz, 3H) ppm.

^{13}C NMR (101 MHz, $CDCl_3$) δ 172.3, 157.3, 157.2, 155.4, 146.8, 143.8, 142.9, 141.2, 138.2, 130.1, 129.8, 129.7, 129.7, 129.5, 129.1, 127.3, 126.7, 126.2, 125.3, 125.0, 123.2, 121.9, 118.9, 118.3, 117.6, 117.5, 113.9, 109.9, 80.3, 72.6, 62.8, 60.8, 60.5, 55.3, 38.4, 36.3, 26.0, 25.9, 14.6, 13.7 ppm.

HRMS (ESI) m/z: $[M+H]^+$ Calcd for $C_{49}H_{52}NO_5^+$ 734.3840; Found 734.3834.

ethyl -3,3-diphenyl-2-(phenylamino)-4-(4-(4-(2-(pyridin-2-yloxy)propoxy)phenoxy)phenyl)butanoate (5ap)

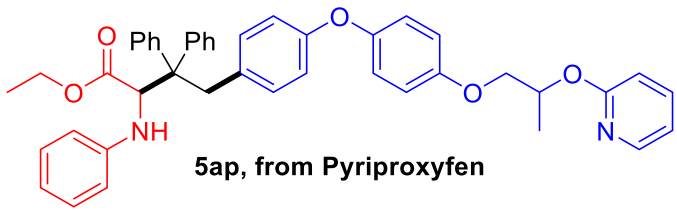

5ap, from Pyriproxyfen

According to procedure G for the synthesis of conjugation of amino acid/peptide with aryl thianthrenium salt to afford **5ap** as a colourless oil (69 mg, 68% yield) (petro ether / ethyl acetate = 10:1, Rf = 0.3, d.r. was not able to be determined by NMR spectra).

^{1}H NMR (400 MHz, $CDCl_3$) δ 8.17–8.15 (m, 1H), 7.58–7.55 (m, 1H), 7.35–7.50 (m, 5H), 7.23–7.22 (m, 3H), 7.18–7.14 (m, 2H), 7.10–7.08 (m, 2H), 6.92 (s, 4H), 6.88–6.85 (m, 1H), 6.76–6.72 (m, 2H), 6.66 (s, 4H), 6.57–6.55 (m, 2H), 5.63–5.56 (m, 1H), 4.88 (d, *J* = 11.2 Hz, 1H), 4.21–4.17 (m, 1H), 4.10–4.06 (m, 2H), 3.92–3.75 (m, 2H), 3.66–3.62 (m, 2H), 1.49 (d, *J* = 6.4 Hz, 3H), 0.87 (t, *J* = 7.2 Hz, 3H) ppm.

13C NMR (101 MHz, $CDCl_3$) δ 172.2, 163.1, 156.8, 155.0, 150.5, 146.7, 146.5, 143.5, 143.1, 138.7, 132.2, 131.2, 129.9, 129.5, 129.2, 127.6, 127.4, 126.9, 126.6, 120.4, 118.6, 116.8, 116.7, 115.7, 113.9, 111.7, 71.0, 69.2, 60.7, 59.5, 55.5, 43.8, 17.0, 13.6 ppm.

HRMS (ESI) m/z: $[M+Na]^+$ Calcd for $C_{44}H_{42}N_2NaO_5^+$ 701.2986; Found 701.2987.

methyl 8-((4-(4-(dimethylamino)-4-oxo-2,2-diphenyl-3-(*p*-tolylamino)butyl)phenyl)amino)-8-oxooctanoate (5lq)

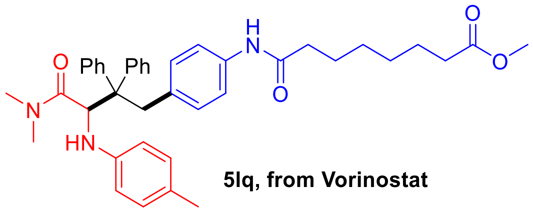

5lq, from Vorinostat

According to procedure G for the synthesis of conjugation of amino acid/peptide with aryl thianthrenium salt to afford **5lq** as a yellow solid (38 mg, 40% yield) (DCM / MeOH = 50:1, Rf = 0.2, m.p. 90 °C).

^{1}H NMR (400 MHz, $CDCl_3$) δ 7.46–7.43 (m, 2H), 7.30–7.26 (m, 3H), 7.23–7.19 (m, 8H), 6.95–6.89 (m, 4H), 6.436–6.32 (m, 2H), 5.04 (d, *J* = 9.3 Hz, 1H), 4.32 (d, *J* = 9.4 Hz, 1H), 4.12–4.09 (m, 1H), 3.76 (d, *J* = 13.6 Hz, 1H), 3.65 (s, 3H), 2.61 (s, 3H), 2.32–2.26 (m, 4H), 2.19–2.17 (m, 6H), 1.70–1.60 (m, 4H), 1.36–1.33 (m, 4H) ppm.

^{13}C NMR (101 MHz, $CDCl_3$) δ 174.3, 171.9, 171.0, 145.4, 143.9, 136.3, 134.5, 131.5, 130.0, 129.7, 129.6, 127.8 127.6, 127.4, 126.7, 126.6, 118.7, 113.5, 60.4, 41.4, 37.5, 36.6, 35.9, 33.9, 28.7, 28.7, 25.3, 24.6, 20.4 ppm.

HRMS (ESI) m/z: $[M+H]^+$ Calcd for $C_{40}H_{48}N_3O_4^+$ 634.3639; Found 634.3647.

ethyl (4-(1-(2-chlorophenyl)-2-oxoindolin-5-yl)-3-phenyl-2-(phenylamino)butanoyl)glycinate (5tk)

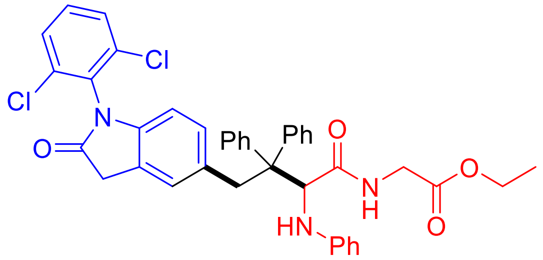

5tk, **from Diclofenac amide and Ph-Gly-Gly-OEt**

According to procedure G for the synthesis of conjugation of amino acid/peptide with aryl thianthrenium salt to afford **5tk** as a yellowish solid (41 mg, 40% yield) (petro ether / ethyl acetate = 5:1, Rf = 0.1, m.p. 130 °C).

^{1}H NMR (400 MHz, $CDCl_3$) δ 7.47–7.44 (m, 4H), 7.39–7.28 (m, 4H), 7.21–7.11 (m, 5H), 7.01 (s, 1H), 6.85–6.81 (m, 1H), 6.78–6.76 (m, 2H), 6.62 (t, *J* = 5.4 Hz, 1H), 6.59–6.56 (m, 2H), 6.51–6.48 (m, 1H), 6.00 (d, *J* = 8.1 Hz, 1H), 4.49 (d, *J* = 13.0 Hz, 1H), 4.40 (d, *J* = 2.1 Hz, 1H), 4.19–4.18 (m, 1H), 4.08–4.03 (m, 2H), 3.90 (dd, *J* = 18.1, 5.9 Hz, 1H), 3.63–3.49 (m, 2H), 3.36 (dd, *J* = 18.0, 4.7 Hz, 1H), 2.98 (d, *J* = 12.9 Hz, 1H), 1.16 (t, *J* = 7.1 Hz, 3H) ppm.

^{13}C NMR (101 MHz, $CDCl_3$) δ 173.8, 172.2, 168.7, 146.5, 143.1, 141.6, 141.4, 135.5, 135.4, 131.9, 130.8, 130.5, 130.5, 130.3, 129.3, 129.0, 128.9, 128.9, 127.9, 127.8, 127.8, 127.3, 126.8, 123.1, 119.6, 114.2, 107.9, 62.0, 61.2, 56.8, 44.4, 41.2, 35.7, 14.0 ppm.

HRMS (ESI) m/z: $[M+H]^+$ Calcd for $C_{40}H_{36}Cl_2N_3O_4^+$ 692.2077; Found 692.2068.

ethyl 3,3,4-tris(4-methoxyphenyl)-2-(phenylamino)butanoate (6ab)

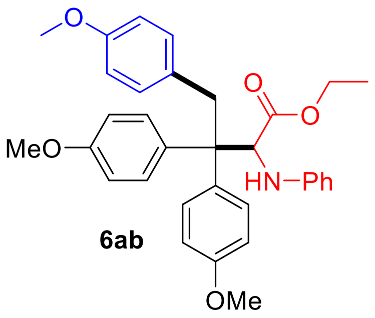

6ab

According to procedure G for the synthesis of conjugation of amino acid/peptide with aryl thianthrenium salt to afford **6ab** as a yellowish solid (49 mg, 62% yield) (petro ether / ethyl acetate = 5:1, Rf = 0.4, m.p. 105 °C).

^{1}H NMR (400 MHz, $CDCl_3$) δ 7.22–7.19 (m, 2H), 7.16–7.12 (m, 2H), 7.03–7.01 (m, 2H), 6.84–6.82 (m, 2H), 6.78–6.75 (m, 2H), 6.73–6.70 (m, 1H), 6.65–6.61 (m, 4H), 6.55–6.53 (m, 2H), 4.76 (s, 1H), 4.05–4.01 (m, 1H), 3.93–3.87 (m, 2H), 3.83 (s, 3H), 3.80 (s, 3H), 3.76 (s, 3H), 3.64–3.61 (m, 1H), 3.46–3.39 (m, 1H), 0.90 (t, J = 7.2 Hz, 3H) ppm.

^{13}C NMR (101 MHz, $CDCl_3$) δ 172.4, 158.4, 158.1, 158.0, 146.6, 135.7, 135.3, 132.2, 131.0, 130.6, 129.2, 118.5, 113.9, 112.8, 112.7, 112.6, 60.6, 59.7, 55.2, 55.2, 55.1, 54.3, 44.0, 13.8 ppm.

HRMS (ESI) m/z: $[M+H]^+$ Calcd for $C_{33}H_{36}NO_5^+$ 526.2588; Found 526.2589.

ethyl 3,3-bis(4-fluorophenyl)-4-(4-methoxyphenyl)-2-(phenylamino)butanoate (6ac)

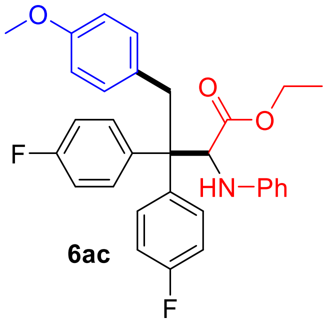

6ac

According to procedure G for the synthesis of conjugation of amino acid/peptide with aryl thianthrenium salt to afford **6ac** as a white solid (46 mg, 61% yield) (petro ether / ethyl acetate = 10:1, Rf = 0.3, m.p. 136 °C).

^{1}H NMR (400 MHz, $CDCl_3$) δ 7.28–7.25 (m, 2H), 7.18–7.14 (m, 2H), 7.07–6.99 (m, 4H), 6.96–6.91 (m, 2H), 6.78–6.75 (m, 1H), 6.66–6.61 (m, 4H), 6.57–6.55 (m, 2H), 4.81 (s, 1H), 4.01–3.94 (m, 1H), 3.93–3.88 (m, 1H), 3.84–3.79 (m, 1H), 3.76 (s, 3H), 3.64–3.57 (m, 1H), 3.49–3.46 (m, 1H), 0.91 (t, 1.8 Hz, 3H) ppm.

^{13}C NMR (101 MHz, CDCl$_3$) δ 172.0, 161.7 (d, $J_{C\text{-}F}$ = 248.0 Hz), 161.6 (d, $J_{C\text{-}F}$ = 247.5 Hz), 158.3, 146.3, 139.2 (d, $J_{C\text{-}F}$ = 3.5 Hz), 138.8 (d, $J_{C\text{-}F}$ = 3.2 Hz), 132.0, 131.5 (d, $J_{C\text{-}F}$ = 7.9 Hz), 131.1 (d, $J_{C\text{-}F}$ = 7.8 Hz), 129.3, 128.4, 119.0, 114.4 (d, $J_{C\text{-}F}$ = 21.1 Hz), 114.2 (d, $J_{C\text{-}F}$ = 21.1Hz), 114.1, 113.0, 60.8, 59.5, 55.1, 54.7, 44.1, 13.7 ppm.

^{19}F NMR (376 MHz, CDCl$_3$) δ -115.6, -115.81 ppm.

HRMS (ESI) m/z: $[M+H]^+$ Calcd for $C_{31}H_{30}F_2NO_3^+$ 502.2188; Found 502.2194.

ethyl 4-(4-methoxyphenyl)-3-methyl-3-phenyl-2-(phenylamino)butanoate (6ad)

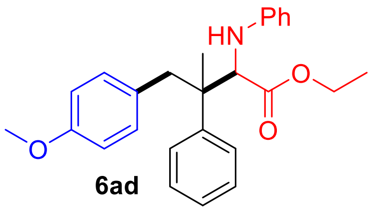

6ad

According to procedure G for the synthesis of conjugation of amino acid/peptide with aryl thianthrenium salt to afford **6ad** with two isomers as a yellowish solid (30 mg, 50% yield) (petro ether / ethyl acetate = 20:1, Rf = 0.3, d.r. = 1:1, d.r. is determined by ^{1}H NMR). The data is written as observed.

^{1}H NMR (400 MHz, CDCl$_3$) δ 7.42–7.30 (m, 4H), 7.28–7.24 (m, 1H), 7.22–7.18 (m, 1H), 7.14–7.10 (m, 1H), 6.82–6.68 (m, 5H), 6.62–6.60 (m, 1H), 6.57–6.51 (m, 1H), 4.42–4.26 (m, 1H), 4.12–4.05 (m, 1H), 3.75–3.71 (m, 3H), 3.32–3.10 (m, 2H), 1.64–1.51 (m, 2H), 1.43–1.35 (m, 3H), 1.13–0.79 (m, 3H) ppm.

^{13}C NMR (101 MHz, CDCl$_3$) δ 172.7, 172.6, 158.1, 158.0, 147.6, 147.0, 142.9, 142.6, 131.8, 131.4, 129.7, 129.4, 129.3, 129.2, 128.1, 127.8, 127.8, 127.3, 126.8, 126.6, 118.7, 118.6, 114.3, 114.0, 113.1, 112.9, 65.3, 64.3, 60.7, 60.4, 55.1, 55.1, 46.4, 45.3, 44.4, 44.2, 20.6, 18.4, 14.1, 13.5 ppm.

HRMS (ESI) m/z: $[M+H]^+$ Calcd for $C_{26}H_{30}NO_3^+$ 404.2220; Found 404.2215.

ethyl 4-(4-(2-oxopyrrolidin-1-yl)phenyl)-2-(phenylamino)-3-(p-tolyl)butanoate (6je)

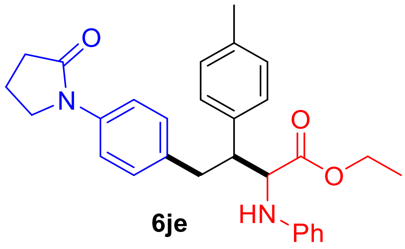

6je

According to procedure G for the synthesis of conjugation of amino acid/peptide with aryl thianthrenium salt to afford **6je** with two isomers as a white solid (25 mg, 37% yield) (petro ether / ethyl acetate = 3:1, Rf = 0.3, m.p. 72 °C, d.r. = 1:1, d.r. is determined by ^{1}H NMR). The data is written as observed.

^{1}H NMR (400 MHz, $CDCl_3$) δ 7.51–7.47 (m, 2H), 7.15–7.00 (m, 8H), 6.74–6.71 (m, 1H), 6.57–6.55 (m, 1H), 6.50–6.48 (m, 1H), 4.26–4.23 (m, 1H), 4.09 (q, *J* = 6.8 Hz, 1H), 3.97 (q, *J* = 7.2 Hz, 1H), 3.85–3.80 (m, 2H), 3.56–3.35 (m, 1H), 3.28–3.21 (m, 1H), 3.08–3.00 (m, 1H), 2.62–2.57 (m, 2H), 2.33–2.28 (m, 3H), 2.18–2.10 (m, 2H), 1.20–1.05 (m, 3H) ppm.

^{13}C NMR (101 MHz, $CDCl_3$) δ 174.1, 174.04 173.2, 172.3, 147.3, 146.5, 137.6, 137.56 136.9, 136.7, 136.3, 135.9, 135.8, 135.7, 129.6, 129.56 129.3, 129.2, 128.9, 128.4, 128.2, 119.8, 119.62 118.5, 118.3, 114.1, 113.8, 61.0, 60.9, 60.6, 59.6, 50.1, 49.2, 48.7, 48.7, 37.5, 37.4, 32.7, 21.0, 18.0, 17.9, 14.2, 13.9 ppm.

HRMS (ESI) m/z: $[M+H]^+$ Calcd for $C_{29}H_{33}N_2O_3^+$ 457.2486; Found 457.2481.

ethyl 4-(4-(2-oxopyrrolidin-1-yl)phenyl)-2-(phenylamino)-3-(4-(trifluoromethyl)phenyl)butanoate (6jf)

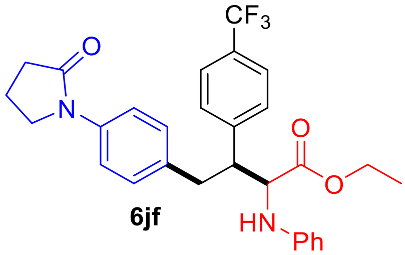

6jf

According to procedure G for the synthesis of conjugation of amino acid/peptide with aryl thianthrenium salt to afford **6jf** with two isomers as a yellowish solid (43 mg,

56% yield) (petro ether / ethyl acetate = 2:1, Rf = 0.4, m.p. 82 °C, d.r. = 1:1, d.r. is determined by ^{1}H NMR). The data is written as observed.

^{1}H NMR (400 MHz, $CDCl_3$) δ 7.55–7.53 (m, 1H), 7.52–7.46 (m, 2H), 7.43–7.38 (m, 1H), 7.32–7.28 (m, 1H), 7.17–7.07 (m, 4H), 7.03–6.84 (m, 1H), 6.78–6.72 (m, 1H), 6.60–6.45 (m, 2H), 4.34–4.26 (m, 1H), 4.18–3.60 (m, 3H), 3.84–3.77 (m, 2H), 3.44–3.24 (m, 1H), 3.14–3.02 (m, 1H), 2.99–2.49 (m, 1H), 2.62–2.56 (m, 2H), 2.18–2.10 (m, 2H), 1.2–0.94 (m, 3H) ppm.

^{13}C NMR (101 MHz, $CDCl_3$) δ 174.2, 174.1, 174.1, 172.7, 172.2, 172.0, 147.0, 146.4, 146.3, 144.1, 143.8, 143.4, 137.9, 137.8, 137.7, 135.2, 134.9, 134.8, 129.6, 129.5, 129.4, 129.3, 129.3, 129.0, 129.0, 128.8, 128.0, 125.4 (q, J_{C-F} = 3.7 Hz), 125.2 (q, J_{C-F} = 4.0 Hz), 119.8, 119.7, 119.6, 119.0, 118.9, 118.8, 114.3, 113.9, 113.89, 61.6, 61.3, 61.2, 61.1, 60.6, 60.0, 50.5, 49.7, 48.7, 48.7, 47.2, 45.8, 42.3, 37.7, 37.4, 37.1, 32.7, 32.7, 18.0, 17.9, 14.2, 13.9, 13.8 ppm.

^{19}F NMR (376 MHz, $CDCl_3$) δ -62.4, -62.4, -62.5, -62.5, -62.6, -62.6.

HRMS (ESI) m/z: $[M+H]^+$ Calcd for $C_{29}H_{30}F_3N_2O_3^+$ 511.2203; Found 511.2200.

ethyl 2-(2-(4-(2-oxopyrrolidin-1-yl)phenyl)-1,2,3,4-tetrahydronaphthalen-1-yl)-2-(phenylamino)acetate (6jg)

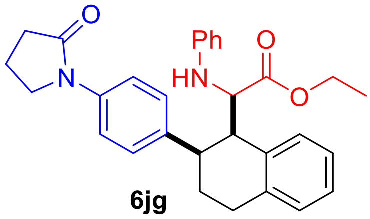

6jg

According to procedure G for the synthesis of conjugation of amino acid/peptide with aryl thianthrenium salt to afford **6jg** with two isomers as a white solid (30 mg, 43% yield) (petro ether / ethyl acetate = 3:1, Rf = 0.3, m.p. 91 °C, d.r. = 1:1, d.r. is determined by ^{1}H NMR). The data is written as observed.

Single isomer of **6jg** can be obtained by recrystallization using n-hexane and dichloromethane, which is suitable for X-ray diffraction.

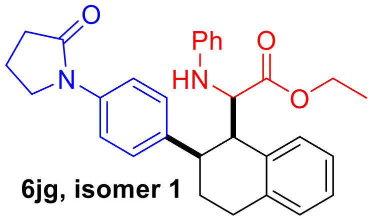

6jg, isomer 1

^{1}H NMR (400 MHz, $CDCl_3$) δ 7.56–7.54 (m, 2H), 7.37–7.35 (m, 1H), 7.28–7.26 (m, 2H), 7.19–7.13 (m, 2H), 7.10–7.08 (m, 1H), 7.04–7.00 (m, 2H), 6.66 (t, J = 7.3 Hz, 1H), 6.28–6.26 (m, 2H), 4.51 (s, 1H), 4.27 (d, J = 4.9 Hz, 1H), 3.95–3.82 (m, 3H), 3.79–3.71 (m, 1H), 3.58–3.55 (m, 1H), 3.50–3.44 (m, 1H), 2.88–2.80 (m, 1H), 2.71–2.60 (m, 3H), 2.21–2.14 (m, 3H), 1.85–1.75 (m, 1H), 0.97 (t, J = 7.1 Hz, 3H) ppm.

^{13}C NMR (101 MHz, $CDCl_3$) δ 174.0, 172.6, 146.5, 142.2, 138.9, 137.8, 135.8, 129.1, 128.8, 128.7, 128.4, 126.4, 126.0, 120.2, 118.2, 113.71, 62.0, 61.1, 48.8, 48.0, 42.5, 32.6, 31.1, 29.0, 18.0, 13.8 ppm.

HRMS (ESI) m/z: $[M+H]^+$ Calcd for $C_{30}H_{33}N_2O_3^+$ 469.2486; Found 469.2489.

ethyl 8-(4-(2-oxopyrrolidin-1-yl)phenyl)-3,3-diphenyl-2-(phenylamino)octanoate (6jh)

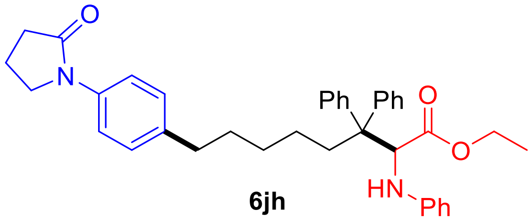

6jh

According to procedure G for the synthesis of conjugation of amino acid/peptide with aryl thianthrenium salt to afford **6jh** as a yellow oil (22 mg, 26% yield) (petro ether / ethyl acetate = 3:1, Rf = 0.4). And performing the synthesis of **6jh** with stop-flow mirco-tube (SFMT) reactor could significantly enhance the efficiency (34 mg, 39% yield).

^{1}H NMR (400 MHz, $CDCl_3$) δ 7.48–7.46 (m, 2H), 7.35–7.27 (m, 5H), 7.25–7.21 (m, 3H), 7.20–7.14 (m, 4H), 7.11–7.08 (m, 2H), 6.77–6.73 (m, 1H), 6.70–6.63 (m, 2H), 4.97 (s, 1H), 3.99–3.91 (m, 2H), 3.84 (t, J = 7.2 Hz, 2H), 2.60 (t, J = 8.0 Hz, 2H), 2.48

(t, J = 7.6 Hz, 2H), 2.39–2.32 (m, 1H), 2.18–2.10 (m, 3H), 1.55–1.47 (m, 2H), 1.46–1.38 (m, 1H), 1.27–1.16 (m, 2H), 1.00 (t, J = 6.8 Hz, 3H), 0.90–0.79 (m, 2H) ppm.

^{13}C NMR (101 MHz, $CDCl_3$) δ 174.0, 172.1, 146.5, 144.4, 143.3, 138.9, 137.0, 129.6, 129.3, 129.0, 128.7, 127.6, 127.5, 126.9, 126.2, 120.0, 118.6, 113.9, 60.6, 60.1, 53.7, 48.9, 39.2, 35.1, 32.7, 31.0, 29.5, 24.0, 18.0, 13.9 ppm.

HRMS (ESI) m/z: $[M+H]^+$ Calcd for $C_{38}H_{43}N_2O_3^+$ 575.3268; Found 575.3261.

ethyl 6,6-bis((ethylperoxy)-l2-methyl)-9-(4-(2-oxopyrrolidin-1-yl)phenyl)-3,3-diphenyl-2-(phenylamino)nonanoate (6ji)

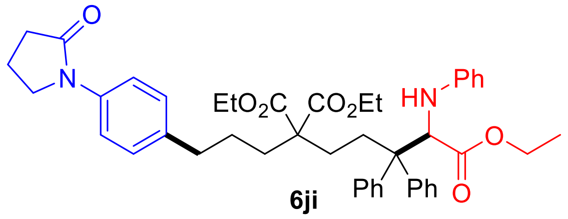

6ji

According to procedure G for the synthesis of conjugation of amino acid/peptide with aryl thianthrenium salt to afford **6ji** as a yellow oil (16 mg, 15% yield) (petro ether / ethyl acetate = 1:1, Rf = 0.2).

^{1}H NMR (400 MHz, $CDCl_3$) δ 7.50–7.47 (m, 2H), 7.31–7.28 (m, 2H), 7.24–7.20 (m, 2H), 7.19–7.08 (m, 8H), 6.76–6.72 (m, 1H), 6.66–6.64 (m, 2H), 4.93 (d, J = 11.6 Hz, 1H), 4.16–4.03 (m, 4H), 4.00–3.80 (m, 7H), 2.60 (t, J = 8.1 Hz, 2H), 2.48 (t, J = 7.4 Hz, 2H), 2.24–2.11 (m, 4H), 2.05–2.00 (m, 2H), 1.84–1.80 (m, 2H), 1.58–1.52 (m, 1H), 1.17 (t, J = 7.1 Hz, 3H), 1.10 (t, J = 7.1 Hz, 3H), 0.91 ((t, J = 6.8 Hz, 3H) ppm.

^{13}C NMR (101 MHz, $CDCl_3$) δ 174.0, 171.6, 171.5, 171.5, 146.5, 143.7, 142.9, 138.0, 137.2, 129.5, 129.3, 129.2, 128.7, 127.7, 127.6, 127.0, 126.5, 119.9, 118.7, 114.1, 61.1, 61.0, 60.7, 60.3, 57.1, 53.6, 48.8, 35.2, 33.1, 32.7, 31.6, 31.2, 26.3, 25.0, 22.6, 18.0, 14.0, 13.9, 13.7 ppm.

HRMS (ESI) m/z: $[M+H]^+$ Calcd for $C_{45}H_{53}N_2O_7^+$ 733.3847; Found 733.3849.

5 Studies on Synthetic Applications

5.1 Gram-scale synthesis

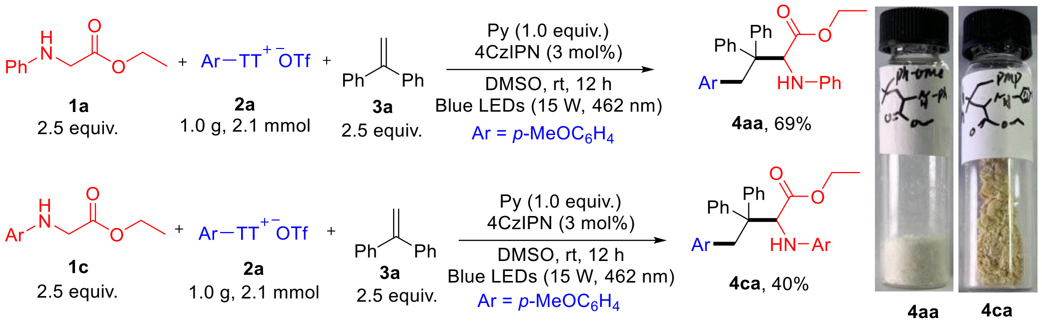

To a 100 mL flame-dried round-bottom flask, equipped with a magnetic stir bar was added **1a** (5.25 mmol, 2.5 equiv.) or **1c** (5.25 mmol, 2.5 equiv.), **2a** (2.1 mmol, 1.0 equiv.), and 4CzIPN (0.063 mmol, 3 mol%). The tube was sealed and connected to a vacuum line where it was evacuated and back-filled with N_2 three times. Then dry DMSO (56 mL), pyridine (2.1 mmol, 1.0 equiv.), and the **3a** (5.25 mmol, 2.5 equiv.) were added. During the reaction stirring process, the tube was constantly irradiated with a blue LED lamp (462 nm, E27, 15 W) keeping the reaction region located in the center of the LED lamp (2 - 3 cm away, with a cooling fan to keep the reaction temperature at 23 - 25 °C) for 48 hours before quenching with H_2O. After the extraction with ethyl acetate (20 mL X 3), the combined organic layer was washed with brine, dried over Na_2SO_4, filtrated, and concentrated under reduced pressure. The crude product was then purified by column chromatography on silica gel using petro ether and ethyl acetate as the eluent to afford **4aa** as a white solid (674 mg, 69% yield) (petro ether / ethyl acetate = 20:1, Rf = 0.4, m.p. 101 °C) or **4ca** as a yellow solid (416 mg, 40% yield) (petro ether / ethyl acetate = 10:1, Rf = 0.4, m.p. 97 °C).

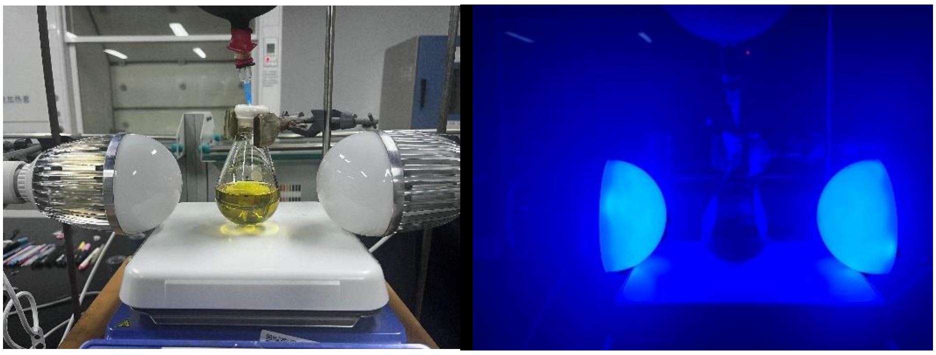

Figure S6. Gram-scale reaction.

5.2 One-pot reaction

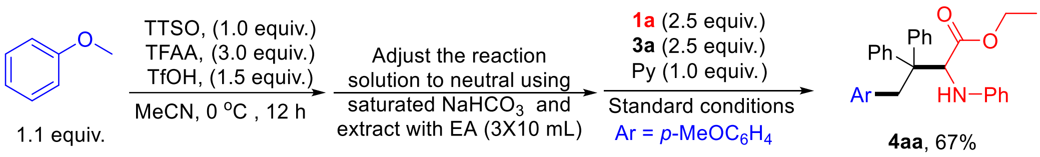

In a nitrogen atmosphere, thianthrene *S*-oxide (TTSO) (0.15 mmol, 1.0 equiv.), MeCN (1.0 mL, 0.15 M.) and anisole (0.165 mmol, 1.1 equiv.) were added to 25 mL schlenk tube. The reaction mixture is then cooled to 0 °C and stirred at this temperature, followed by a slow drip of TFAA (0.45 mmol, 3.0 equiv.) and TfOH (0.225 mmol, 1.5 equiv.). The reaction mixture was stirred at 0 °C for 1 h, then stirred at room temperature (25 °C) for 12 h, neutralized with saturated $NaHCO_3$ aqueous solution, and extracted with ethyl acetate (20 mL X 3). The combined organic layer was dried on anhydrous Na_2SO_4 and condensed under reduced pressure. The residue could be employed in subsequent transformations without purification.

To an oven-dried transparent sample bottle (8 mL) equipped with a magnetic stir bar was added **1a** (0.375 mmol, 2.5 equiv.), the residue (crude **2a,** 0.15 mmol, 1.0 equiv.), and 4CzIPN (0.0045 mmol, 3 mol%). The tube was sealed and connected to a vacuum line where it was evacuated and back-filled with N_2 three times. Then dry DMSO (4 mL), pyridine (0.15 mmol, 1.0 equiv.), and the **3a** (0.375 mmol, 2.5 equiv.) were added. During the reaction stirring process, the tube was constantly irradiated with a blue LED lamp (462 nm, E27, 15 W) keeping the reaction region located in the center of the LED lamp (2 - 3 cm away, with a cooling fan to keep the reaction temperature at

23 - 25 °C) for 12 hours before quenching with H_2O. After the extraction with ethyl acetate (20 mL X 3), the combined organic layer was washed with brine, dried over Na_2SO_4, filtrated, and concentrated under reduced pressure. The crude product was then purified by column chromatography on silica gel using petroleum ether and ethyl acetate as the eluent to afford **4aa** as a white solid (47 mg, 67% yield) (petro ether / ethyl acetate = 20:1, Rf = 0.4, m.p. 101 °C).

5.3 Preparation of D-labeled bioactive compounds (7)

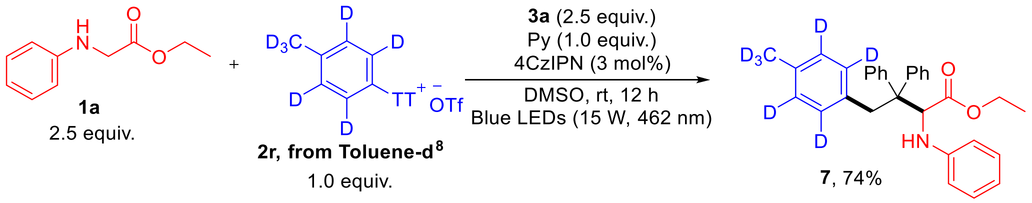

To an oven-dried transparent sample bottle (8 mL) equipped with a magnetic stir bar was added **1a** (0.375 mmol, 2.5 equiv.), **2r** (from toluene-d^8, 0.15 mmol, 1.0 equiv.), and 4CzIPN (0.0045 mmol, 3 mol%). The tube was sealed and connected to a vacuum line where it was evacuated and back-filled with N_2 three times. Then dry DMSO (4 mL), pyridine (0.15 mmol, 1.0 equiv.), and the **3a** (0.375 mmol, 2.5 equiv.) were added. During the reaction stirring process, the tube was constantly irradiated with a blue LED lamp (462 nm, E27, 15 W) keeping the reaction region located in the center of the LED lamp (2 - 3 cm away, with a cooling fan to keep the reaction temperature at 23 - 25 °C) for 12 hours before quenching with H_2O. After the extraction with ethyl acetate (20 mL X 3), the combined organic layer was washed with brine, dried over Na_2SO_4, filtrated, and concentrated under reduced pressure. The crude product was then purified by column chromatography on silica gel using petroleum ether and ethyl acetate as the eluent to afford **7** as a white solid (51 mg, 74% yield) (petro ether / ethyl acetate = 50:1, Rf = 0.2, m.p. 134 °C).

^1H NMR (400 MHz, $CDCl_3$) δ 7.37–7.31 (m, 5H), 7.25–7.22 (m, 3H), 7.17–7.11 (m, 4H), 6.77–6.73 (m, 1H), 6.58–6.56 (m, 2H), 4.90 (d, J = 11.4 Hz, 1H), 4.12 (d, J = 11.4 Hz, 1H), 3.93–3.77 (m, 2H), 3.71–3.62 (m, 2H), 0.89 (t, J = 7.1 Hz, 3H) ppm.

^{13}C NMR (101 MHz, $CDCl_3$) δ 172.2, 146.5, 143.7, 143.4, 135.5, 133.7, 130.6 (t, *J* = 24.3 Hz), 129.9, 129.6, 129.2, 127.8 (t, *J* = 22.7 Hz), 127.5, 127.3, 126.8, 126.6, 118.5, 113.9, 60.6, 59.6, 55.4, 44.0, 20.1 (m), 13.7 ppm.

HRMS (ESI) m/z: $[M+H]^+$ Calcd for $C_{31}H_{25}D_7NO_2^+$ 457.2867; Found 457.2861.

5.4 Preparation of ^{13}C-labeled bioactive compounds (8)

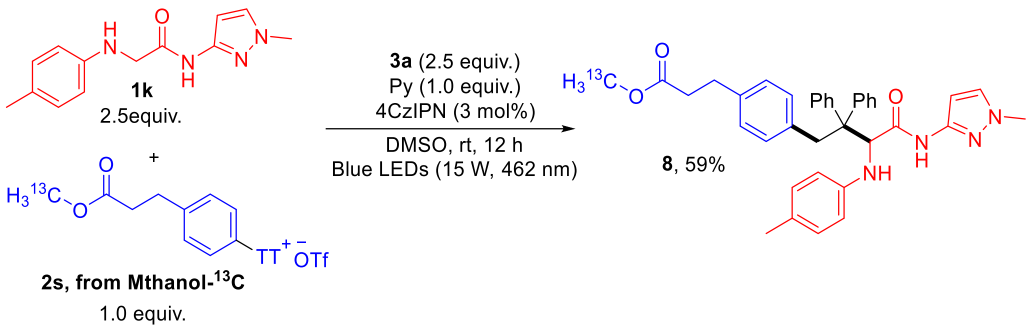

To an oven-dried transparent sample bottle (8 mL) equipped with a magnetic stir bar was added **1k** (0.375 mmol, 2.5 equiv.), **2s** (from mthanol-^{13}C, 0.15 mmol, 1.0 equiv.), and 4CzIPN (0.0045 mmol, 3 mol%). The tube was sealed and connected to a vacuum line where it was evacuated and back-filled with N_2 three times. Then dry DMSO (4 mL), pyridine (0.15 mmol, 1.0 equiv.), and the **3a** (0.375 mmol, 2.5 equiv.) were added. During the reaction stirring process, the tube was constantly irradiated with a blue LED lamp (462 nm, E27, 15 W) keeping the reaction region located in the center of the LED lamp (2 - 3 cm away, with a cooling fan to keep the reaction temperature at 23 - 25 °C) for 12 hours before quenching with H_2O. After the extraction with ethyl acetate (20 mL X 3), the combined organic layer was washed with brine, dried over Na_2SO_4, filtrated, and concentrated under reduced pressure. The crude product was then purified by column chromatography on silica gel using petroleum ether and ethyl acetate as the eluent to afford **8** as a white solid (52 mg, 59% yield) (petro ether / ethyl acetate = 20:1, Rf = 0.2, m.p. 178 °C).

^{1}H NMR (400 MHz, $CDCl_3$) δ 8.40 (s, 1H), 7.46–7.44 (m, 2H), 7.31–7.27 (m, 2H), 7.25–7.22 (m, 1H), 7.21–7.09 (m, 4H), 6.95 (d, *J* = 8.0 Hz, 2H), 6.84–6.80 (m, 4H), 6.72–6.71 (m, 2H), 6.60 (d, *J* = 2.4 Hz, 1H), 6.46–6.44 (m, 2H), 4.56 (d, *J* = 12.8 Hz,

1H), 4.40 (d, J = 0.8 Hz, 1H), 4.13 (s, 1H), 3.80 (s, 1.5H), 3.65 (s, 3H), 3.43 (s, 1.5H), 2.99 (d, J = 12.8 Hz, 1H), 2.80 (t, J = 8.0 Hz, 2H), 2.52 (t, J = 7.6 Hz, 2H), 2.20 (s, 3H) ppm.

^{13}C NMR (101 MHz, $CDCl_3$) δ 173.4 (d, J = 2.6 Hz), 173.4, 170.1, 146.1, 144.2, 142.9, 141.8, 138.0, 135.1, 131.7, 130.3, 130.3, 129.9, 128.9, 128.0, 127.7, 127.3, 127.0, 126.6, 114.1, 96.7, 62.6, 56.9, 51.5, 44.4, 38.6, 35.7, 30.5, 20.4 ppm.

HRMS (ESI) m/z: $[M+H]^+$ Calcd for $C_{36}{}^{13}CH_{38}N_4O_3{}^+$ 588.3050; Found 588.3048.

99% ^{13}C (calculated from HRMS).

Peak area ration = 840138/(5873+840138) × 100% = 99%.

5.5 Preparation of methyl 8-((4-(4-(((8*S*,9*R*,10*S*,13*R*,14*R*)-10,13-dimethyl-17-(pyridin-3-yl)-2,3,4,7,8,9,10,11,12,13,14,15-dodecahydro-1*H*-cyclopenta[*a*]phenanthren-3-yl)oxy)-4-oxo-2,2-diphenyl-3-(*p*-tolylamino)butyl)phenyl)amino)-8-oxooctanoate (9)

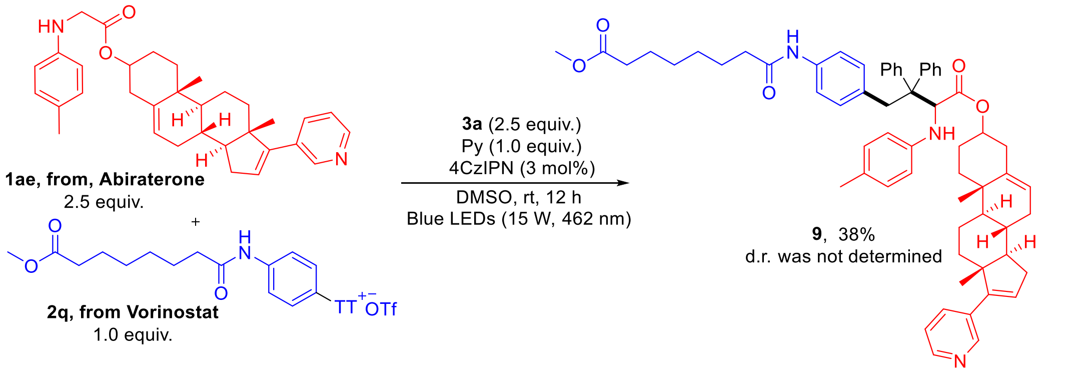

To an oven-dried transparent sample bottle (8 mL) equipped with a magnetic stir bar was added **1ae** (0.375 mmol, 2.5 equiv.), **2q** (from Vorinostat, 0.15 mmol, 1.0 equiv.), and 4CzIPN (0.0045 mmol, 3mol%). The tube was sealed and connected to a vacuum line where it was evacuated and back-filled with N_2 three times. Then dry DMSO (4 mL), pyrinde (0.15 mmol, 1.0 equiv.), and the **3a** (0.375 mmol, 2.5 equiv.) were added. During the reaction stirring process, the tube was constantly irradiated with a blue LED lamp (462 nm, E27, 15 W) keeping the reaction region located in the center of the LED lamp (2 - 3 cm away, with a cooling fan to keep the reaction temperature at

23 - 25 °C) for 12 hours before quenching with H_2O. After the extraction with ethyl acetate (20 mL X 3), the combined organic layer was washed with brine, dried over Na_2SO_4, filtrated, and concentrated under reduced pressure. The crude product was then purified by column chromatography on silica gel using DCM and MeOH as the eluent to afford **9** as a yellowish solid (53 mg, 38% yield) (DCM/MeOH = 30:1, Rf = 0.2, m.p. 117 °C).

The isomer of **9** can't be separated by column chromatography on silica gel, d.r. was not determined by ^1H NMR analysis.

^1H NMR (400 MHz, $CDCl_3$) δ 8.61 (s, 1H), 8.45 (s, 1H), 7.69–7.66 (m, 1H), 7.31–7.25 (m, 6H), 7.24–7.19 (m, 6H), 7.14–7.06 (m, 2H), 6.94 (d, *J* = 8.0 Hz, 2H), 6.66–6.64 (m, 2H), 6.49–6.47 (m, 2H), 6.00–5.97 (m, 1H), 5.30–5.25 (m, 1H), 4.81–4.78 (m, 1H), 4.39–4.31 (m, 1H), 3.95–3.92 (m, 1H), 3.73–3.49 (m, 5H), 2.33–2.25 (m, 5H), 2.22 (s, 3H), 2.08–1.95 (m, 4H), 1.91–1.84 (m, 1H), 1.75–1.52 (m, 10H), 1.48–1.25 (m, 8H), 1.21–1.10 (m, 1H), 1.01 (s, 3H), 0.93 (s, 3H) ppm.

^{13}C NMR (101 MHz, $CDCl_3$) δ 174.2, 171.6, 171.6, 171.0, 151.3, 147.1, 147.0, 144.2, 143.6, 143.2, 143.2, 139.9, 139.7, 136.3, 134.3, 132.9, 131.6, 130.1, 129.7, 129.6, 127.8, 127.5, 127.3, 126.8, 126.6, 123.3, 123.3, 123.2, 122.1, 122.1, 118.5, 114.2, 59.7, 59.6, 57.4, 55.4, 55.4, 51.5, 50.1, 47.3, 44.2, 44.2, 37.6, 37.5, 36.7, 36.7, 36.6, 35.1, 33.9, 31.7, 31.4, 31.4, 30.3, 28.8, 28.7, 27.2, 27.2, 25.3, 24.7, 20.7, 20.4, 19.1, 16.5 ppm.

HRMS (ESI) m/z: $[M+H]^+$ Calcd for $C_{62}H_{72}N_3O_5^+$ 938.5466; Found 938.5461.

5.6 Preparation of (8*R*,9*S*,10*R*,13*S*,14*S*)-10,13-dimethyl-17-(pyridin-3-yl)-2,3,4,7,8,9,10,11,12,13,14,15-dodecahydro-1*H*-cyclopenta[a]phenanthren-3-yl-4-(4-(8-(hydroxyamino)-8-oxooctanamido)phenyl)-3,3-diphenyl-2-(*p*-tolylamino)butanoate (10)

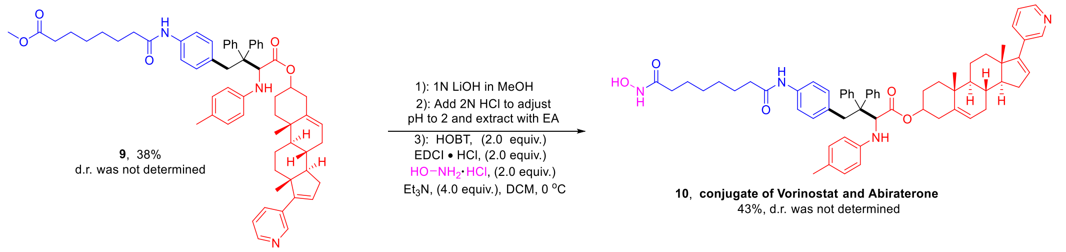

The compound **9** (0.3 mmol, 1.0 equiv.) was added to a 100 mL flame-dried round-bottom flask in an ice-water bath, followed by the addition of 1 N LiOH in MeOH (10 mL), and the reaction was allowed to stirred at 0 °C until complete conversion is detected by TLC, remove methanol under reduced pressure and adjust the pH to 2 with 2 N HCl, diluted with water (30 mL) and extracted with ethyl acetate (20 mL X 3). The combined organic extracts were washed with brine (20 mL X 3), dried over Na_2SO_4 and concentrated under reduced pressure. The residue could be employed in subsequent transformations without purification.

To a 100 mL round-bottom flask, the residue, EDCI·HCl (1-ethyl-3-(3-dimethylaminopropyl)carbodiimide hydrochloride, 0.6 mmol, 2.0 equiv.) and HOBt (1-hydroxybenzotriazole, 0.6 mmol, 2.0 equiv.) were dissolved in DCM (5 mL) under N_2 atmosphere, and the mixture was stirred at 25 °C for 30 min. Then the reaction was cooled in an ice-water bath. The hydroxylamine hydrochloride (0.6 mmol, 2.0 equiv.) was added into the flask, followed by the addition of Et_3N (1.2 mmol, 4.0 equiv.) under N_2. After 30 min, the reaction mixture was warmed up to 25 °C and stirred overnight. After quenching the reaction with water (30 mL), the resulting mixture was separated and extracted with ethyl acetate (20 mL X 3). The combined organic layers were dried over Na_2SO_4, concentrated under reduced pressure, and purified through a silica gel column using DCM and MeOH as the eluent to afford **10** as a yellowish solid (107 mg, 38% yield) (DCM/MeOH = 10:1, Rf = 0.2, m.p. 111 °C).

The isomer of **10** can't be separated by column chromatography on silica gel, d.r. was not determined by ^{1}H NMR analysis.

^{1}H NMR (400 MHz, $CDCl_3$) δ 8.62–8.48 (m, 1H), 7.72 (d, *J* = 7.9 Hz, 1H), 7.36 (s, 1H), 7.31–7.27 (m, 6H), 7.22–7.20 (m, 5H), 7.12–7.08 (m, 2H), 6.94 (d, *J* = 8.1 Hz, 2H), 6.64 (d, *J* = 8.1 Hz, 2H), 6.48 (d, *J* = 8.0 Hz, 2H), 6.01 (s, 1H), 5.29–5.23 (m, 1H), 4.79 (d, *J* = 8.6 Hz, 1H), 4.39–4.31 (m, 1H), 3.97–3.87 (m, 1H), 3.72–3.48 (m, 3H), 2.37–2.29 (m, 4H), 2.21 (s, 3H), 2.06–1.95 (m, 4H), 1.90–1.84 (m, 1H), 1.73–1.62 (m, 6H), 1.60–1.52 (m, 3H), 1.42–1.37 (m, 4H), 1.28–1.24 (m, 6H), 1.15–1.13 (m, 1H), 1.01 (s, 3H), 0.93 (s, 3H), 0.90–0.81 (m, 2H) ppm.

^{13}C NMR (101 MHz, $CDCl_3$) δ 177.9, 171.7, 171.3, 151.0, 146.2, 144.2, 143.3, 143.2, 139.8, 139.7, 136.3, 134.9, 132.8, 131.6, 130.1, 130.0, 129.7, 128.0, 127.8, 127.5, 127.3, 126.8, 126.6, 126.2, 122.1, 118.6, 114.2, 74.4, 59.5, 57.3, 55.3, 50.0, 47.2, 44.2, 37.5, 36.6, 35.0, 34.0, 31.8, 31.4, 30.2, 29.7, 28.7, 28.7, 27.2, 25.3, 24.6, 22.7, 20.7, 20.4, 19.1, 16.5, 14.1 ppm.

HRMS (ESI) m/z: $[M-H]^-$ Calcd for $C_{61}H_{69}N_4O_5^-$ 937.5267; Found 937.5266.

5.7 Biological evaluation of 10

Procedure for determination of DU145 cell viability by MTT assay: Human prostate cancer cells (DU145) were seeded in 96-well plates at the density of 2000 cells per well with 100 μL of complete culture medium. After adhesion for 24 hours, selected products were added to the medium with five concentrations ranging from 1.2 μM to 100 μM. The cells were then cultured for another 48 h. At the end of stimulation, 20 μL MTT (5 mg/mL) was added to the medium, and the cells were cultured for another 4 h. Then, the supernatant was removed, and 100 μL of DMSO was added to each well for another 15 min at 37 °C. The culture plates were then shaken for 2 min and the optical density (OD) values were read at a wave-length of 570 nm in a microplate reader (BioTek ELx800, USA). Note: N=3 for each experimental group, and measurements were taken from 3 distinct samples.

Table S11. IC50 values of different compounds for DU145.

Compound	DU145 IC50 (μM)
**Vorinostat**	0.932
**Abiraterone**	31.532
**10**	52.087

5.8 Set-up of SFMT reaction of 6jh

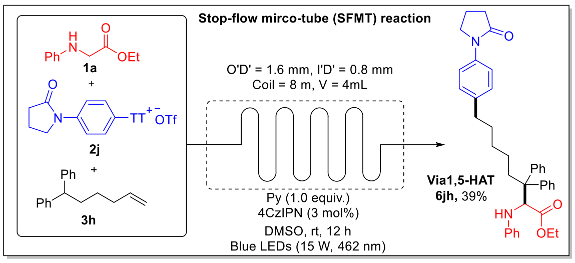

To an oven-dried transparent sample bottle (8 mL) equipped with a magnetic stir bar was added **1a** (0.375 mmol, 2.5 equiv.), **2j** (0.15 mmol, 1.0 equiv.), and 4CzIPN (0.0045 mmol, 3 mol%). The tube was sealed and connected to a vacuum line where it was evacuated and back-filled with N_2 three times. Then dry DMSO (4 mL), Py (0.15 mmol, 1.0 equiv.), and the **3h** (0.375 mmol, 2.5 equiv.) were added. After the raw materials are completely dissolved, use a syringe to inject the reaction solution into perfluoroalkoxyalkane (PFA) stop-flow tubing reactor (SFMT reactor, O'D' = 1.6 mm, I'D' = 0.8 mm, Coil = 8 m, V = 4mL). The tube was constantly irradiated with a blue LED lamp (462 nm, E27, 15 W) keeping the reaction region located in the center of the LED lamp (2 - 3 cm away, with a cooling fan to keep the reaction temperature at 23 - 25 °C) for 12 hours before quenching with H_2O. After the extraction with ethyl acetate (20 mL X 3), the combined organic layer was washed with brine, dried over Na_2SO_4, filtrated, and concentrated under reduced pressure. The crude product was then purified by column chromatography on silica gel using petroleum ether and ethyl acetate as the eluent to afford **6jh** as a yellow oil (34 mg, 39% yield) (petro ether / ethyl acetate = 3:1, Rf = 0.4).

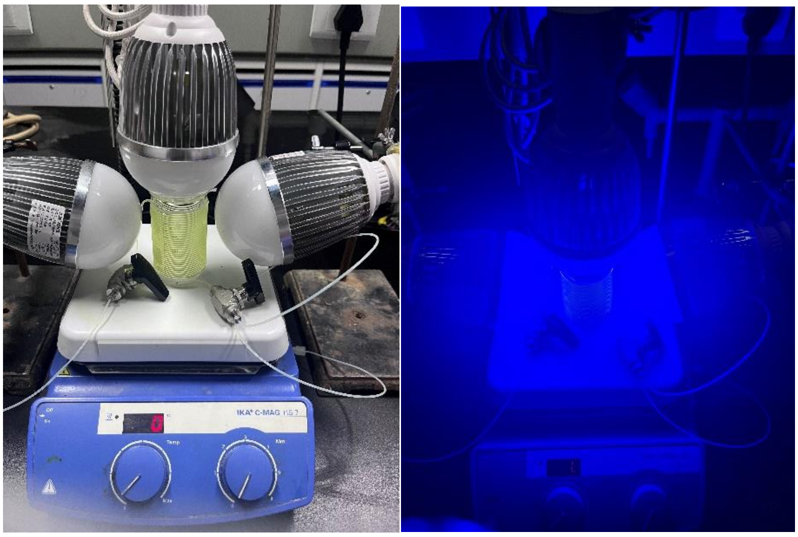

Figure S7. Set-up of SFMT reaction.

6 Mechanistic Studies

6.1 Trapping experiments

To an oven-dried transparent sample bottle (8 mL) equipped with a magnetic stir bar was added **1a** (0.375 mmol, 2.5 equiv.), **2a** (0.15 mmol, 1.0 equiv.), 4CzIPN (0.0045 mmol, 3 mol%), and TEMPO (0.15 mmol, 1.0 equiv.). The tube was sealed and connected to a vacuum line where it was evacuated and back-filled with N_2 three times. Then dry DMSO (4 mL), pyridine (0.15 mmol, 1.0 equiv.), and the **3a** (0.375 mmol, 2.5 equiv.) were added. During the reaction stirring process, the tube was constantly irradiated with a blue LED lamp (462 nm, E27, 15 W) keeping the reaction region located in the center of the LED lamp (2 - 3 cm away, with a cooling fan to keep the reaction temperature at 23 - 25 °C) for 12 h. Then, the reaction mixture was analyzed by HRMS, which shows the exact molecular ion indicating the formation of the proposed TEMPO adduct to the *p*-methoxyphenyl radical intermediate, the stabilized α-alkyl radical intermediate, and benzyl radical intermediate.

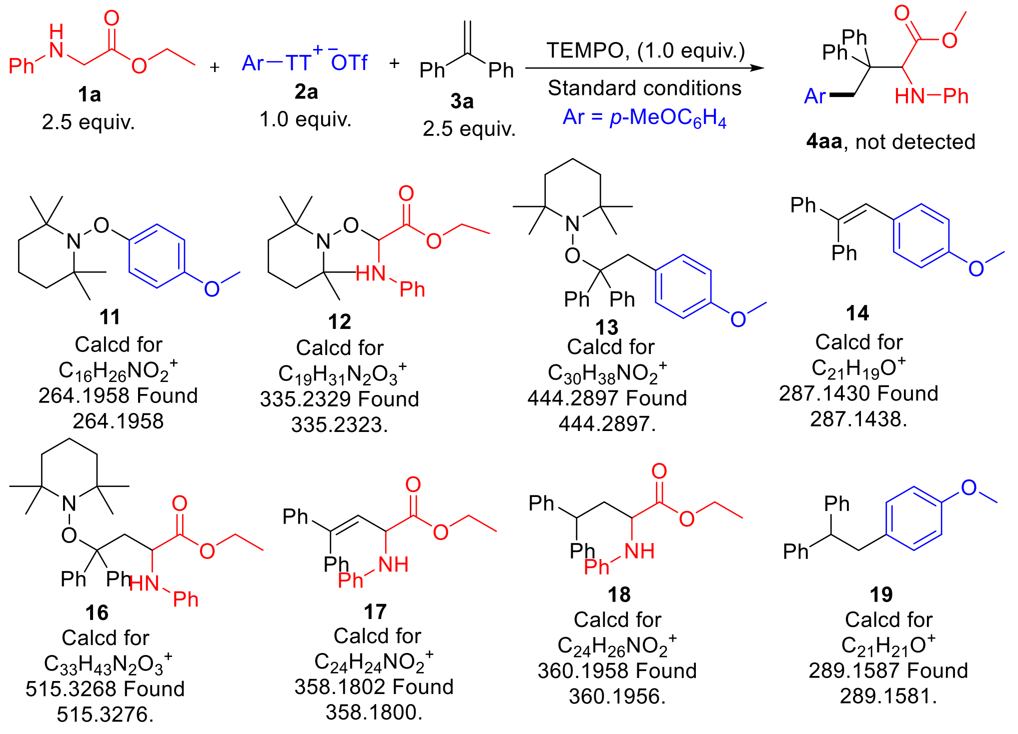

11 HRMS (ESI) m/z: $[M+H]^+$ Calcd for $C_{16}H_{26}NO_2^+$ 264.1958 Found 264.1958

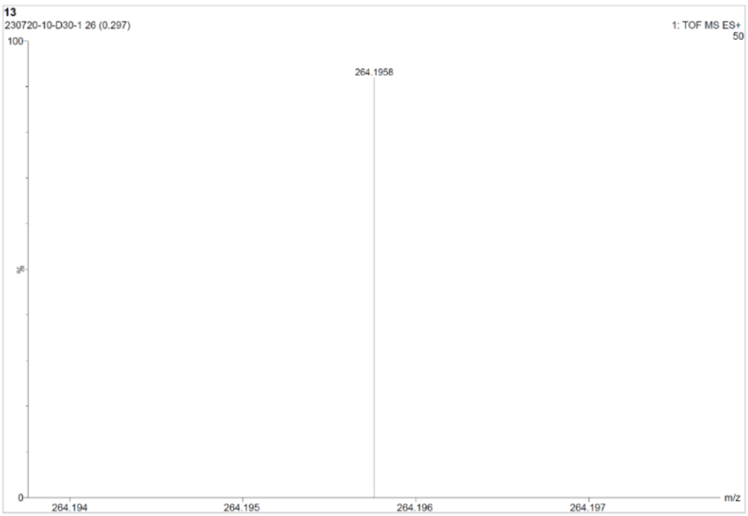

Figure S8. HRMS of **11.**

12 HRMS (ESI) m/z: $[M+H]^+$ Calcd for $C_{19}H_{31}N_2O_3^+$ 335.2329 Found 335.2323.

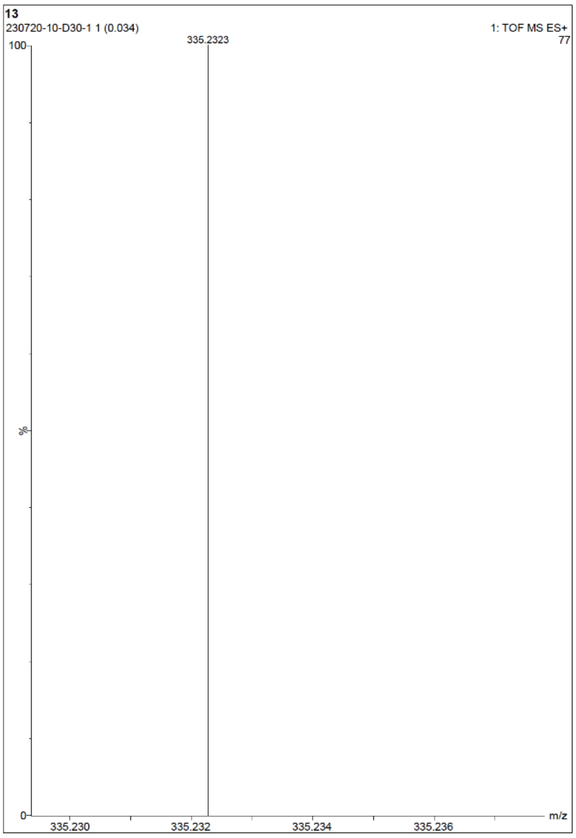

Figure S9. HRMS of **12.**

13 HRMS (ESI) m/z: $[M+H]^+$ Calcd for $C_{30}H_{38}NO_2^+$ 444.2897 Found 444.2897.

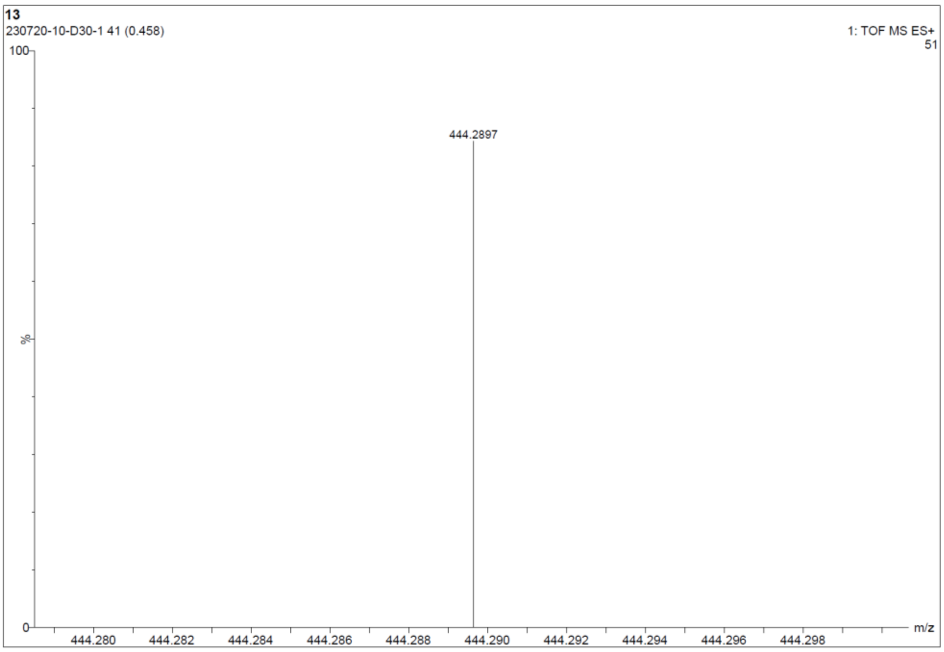

Figure S10. HRMS of **13.**

14 HRMS (ESI) m/z: $[M+H]^+$ Calcd for $C_{21}H_{19}O^+$ 287.1430 Found 287.1438.

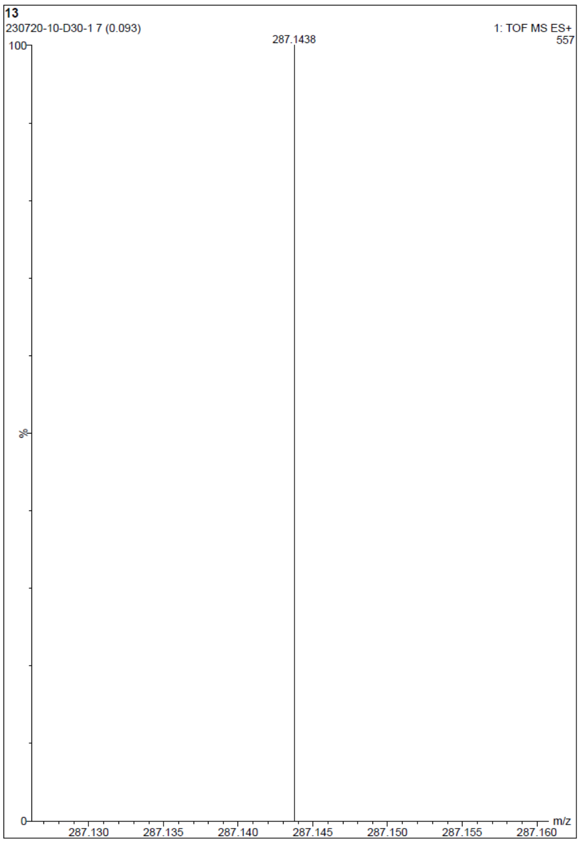

Figure S11. HRMS of **14.**

16 HRMS (ESI) m/z: [M+H]$^+$ Calcd for $C_{33}H_{43}N_2O_3^+$ 515.3268 Found 515.3276.

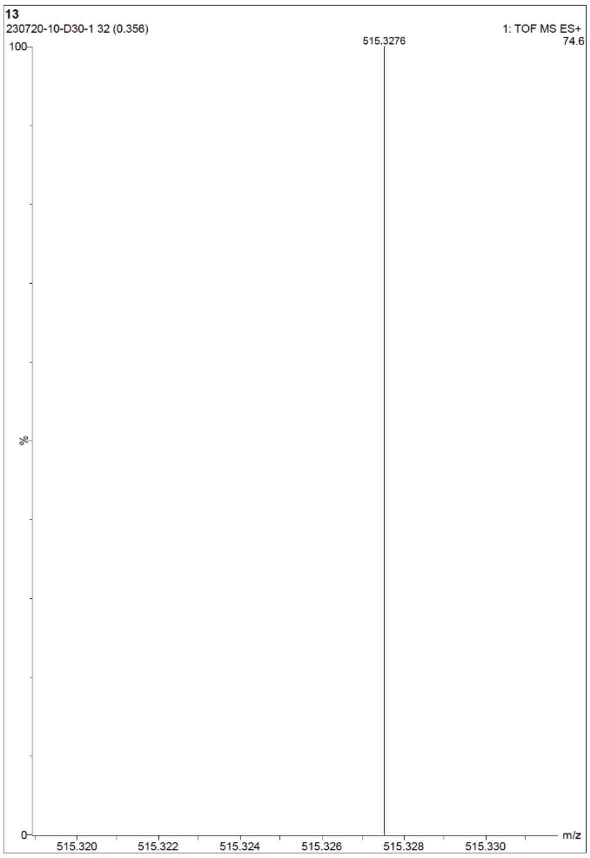

Figure S12. HRMS of **16.**

17 HRMS (ESI) m/z: [M+H]$^+$ Calcd for $C_{24}H_{24}NO_2^+$ 358.1802 Found 358.1800.

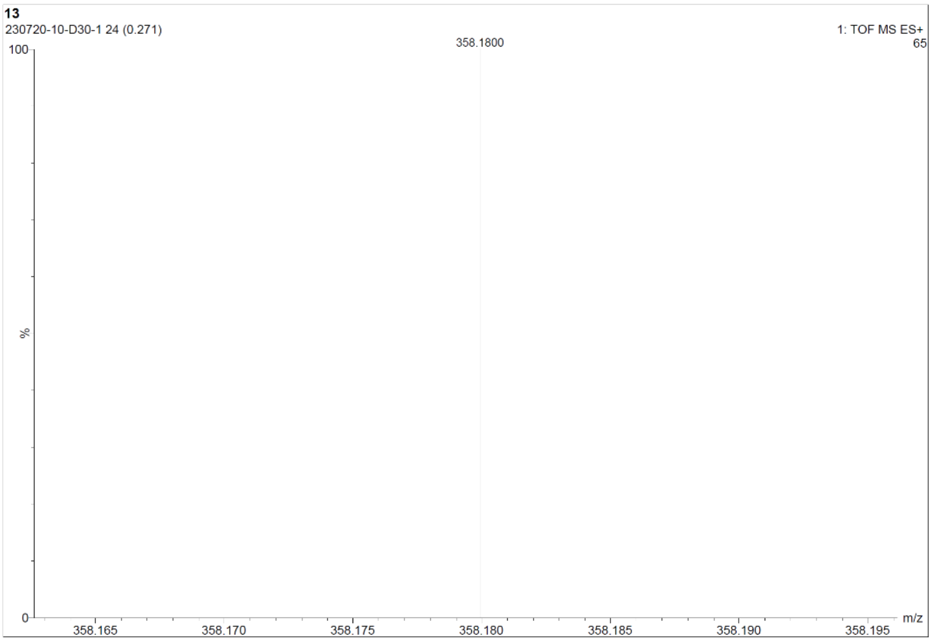

Figure S13. HRMS of **17.**

18 HRMS (ESI) m/z: $[M+H]^+$ Calcd for $C_{24}H_{26}NO_2^+$ 360.1958 Found 360.1956.

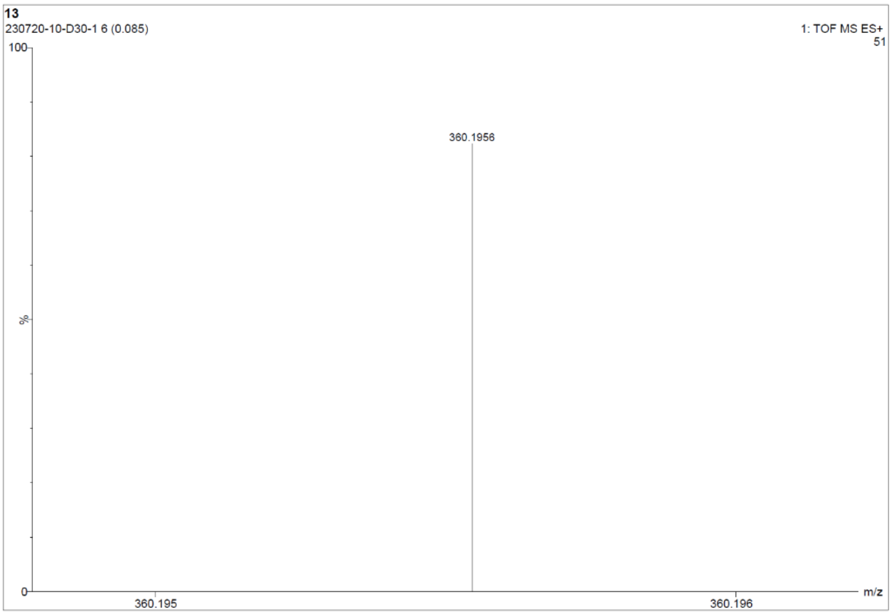

Figure S14. HRMS of **18.**

19 SHRMS (ESI) m/z: $[M+H]^+$ Calcd for $C_{21}H_{21}O^+$ 289.1587 Found 289.1581.

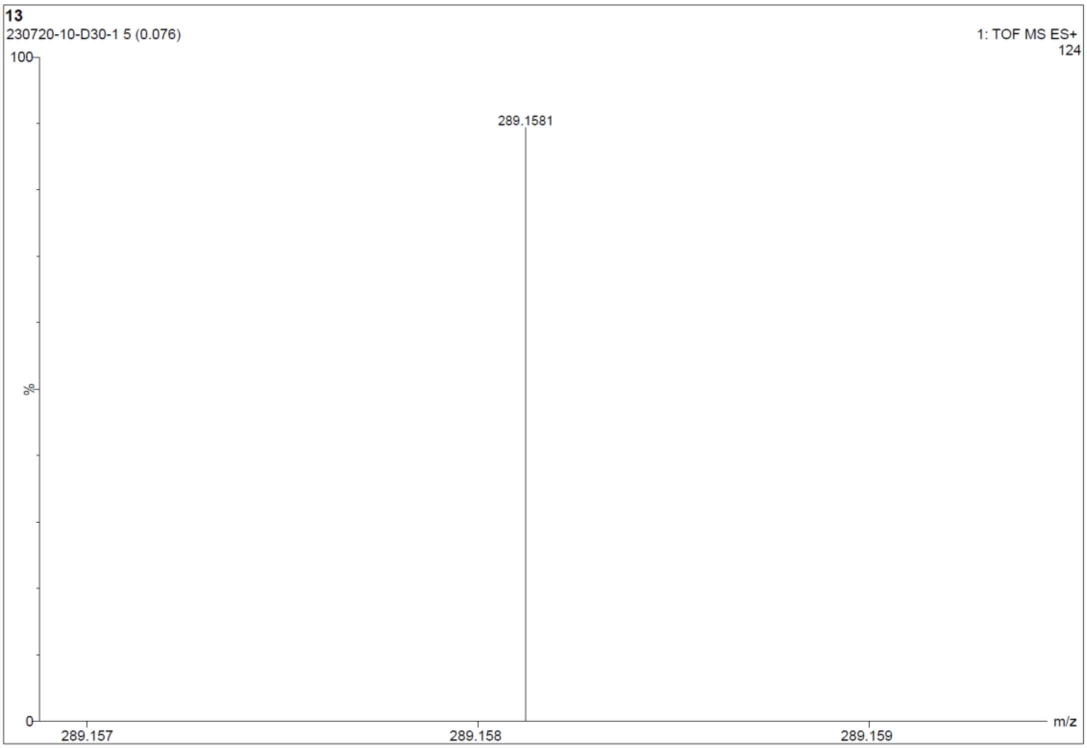

Figure S15. HRMS of **19.**

6.2 Controlled experiments

Reactivity of imine 15 under these conditions

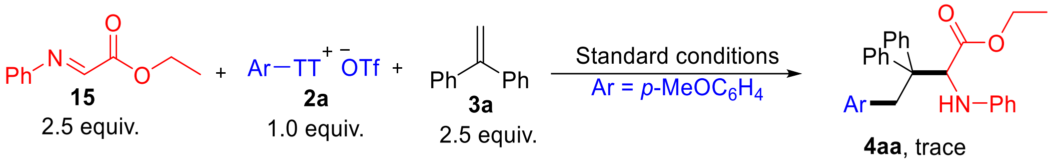

To an oven-dried transparent sample bottle (8 mL) equipped with a magnetic stir bar was added **15** (0.375 mmol, 2.5 equiv.), **2a** (0.15 mmol, 1.0 equiv.), and 4CzIPN (0.0045 mmol, 3 mol%). The tube was sealed and connected to a vacuum line where it was evacuated and back-filled with N_2 three times. Then dry DMSO (4 mL), pyridine (0.15 mmol, 1.0 equiv.), and the **3a** (0.375 mmol, 2.5 equiv.) were added. During the reaction stirring process, the tube was constantly irradiated with a blue LED lamp (462 nm, E27, 15 W) keeping the reaction region located in the center of the LED lamp (2 - 3 cm away, with a cooling fan to keep the reaction temperature at 23 - 25 °C) for 12 hours before quenching with H_2O. After the extraction with ethyl acetate (20 mL X 3), the combined organic layer was washed with brine, dried over Na_2SO_4, filtrated, and concentrated under reduced pressure. Trace of **4aa** was detected by thin-layer chromatography (TLC), indicating the importance of the glycine structure for product formation.

Reactivity in the absence of photocatalyst with different wavelengths of light-emitting diode and temperature

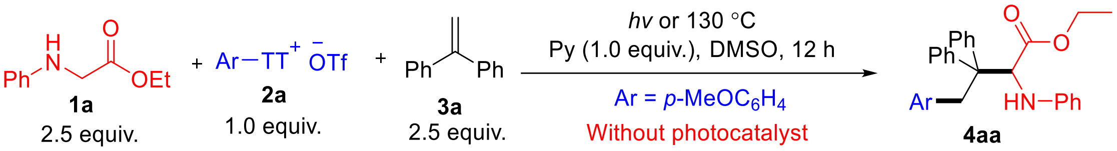

To an oven-dried transparent sample bottle (8 mL) equipped with a magnetic stir bar was added **1a** (0.375 mmol, 2.5 equiv.) and **2a** (0.15 mmol, 1.0 equiv.). The tube was sealed and connected to a vacuum line where it was evacuated and back-filled with N_2 three times. Then dry DMSO (4 mL), pyridine (0.15 mmol, 1.0 equiv.), and the **3a** (0.375 mmol, 2.5 equiv.) were added. During the reaction stirring process, the tube was

constantly irradiated with LED lamp keeping the reaction region located in the center of the LED lamp (2 - 3 cm away, with a cooling fan to keep the reaction temperature at 23 - 25 °C) for 12 hours before quenching with H_2O. After the extraction with ethyl acetate (20 mL X 3), the combined organic layer was washed with brine, dried over Na_2SO_4, filtrated, and concentrated under reduced pressure. The crude product was then purified by column chromatography on silica gel using petroleum ether and ethyl acetate as the eluent to afford **4aa**. It was found that under 365 nm irradiation the reaction was able to produce in 30% yield of **4aa** without the assistance of photocatalyst. Further experiments shown that the conversion could be observed under irradiation with 460 nm or shorter wavelength, while longer wavelength irradiation or heating failed to initiate the reaction (Table S12). These results supported the formation of an EDA complex of **1a** and **2a**.

Table S12. **Reactions irradiated under different wavelengths and temperature in the absence of photocatalyst**

Entry[a]	Wavelength (nm)	Power (W)	Yield [%]
1[a]	365	30	17
2[b]	365	15	30
3[a]	395-400	15	23
4[b]	430-435	15	24
5[b]	440-445	15	25
6[b]	450-455	15	17
7[b]	460-465	15	trace
8[b]	470-475	15	trace
9[c]	462	15	trace
10[d]	527	15	N.D.
11[e]	white LEDs	24	N.D.
12[f]	-	-	N.D.

Reactions were carried out on 0.15 mmol scale. Yields refer to isolated yields. [a] Reaction under irradiation with UV LED lamp (30 W, purchased from Rongyao Lighting Technology Co., Ltd). [b] Reaction under LED irradiation with PhotoSyn-10 parallel photoreactor purchased from Shanghai Quanhuan Technology Co., Ltd. [c] Reaction under irradiation with blue LED lamp (462 nm, E27, 15 W, purchased from Zhongshan Langniu Lighting Technology Co., Ltd). [d] Reaction under irradiation with green LED lamp (527 nm, E27, 15 W, purchased from Zhongshan Langniu Lighting Technology Co., Ltd). [e] Reaction bottle under irradiation with white LED lamp (E27, 24 W, 6500 K, purchased from Bull Group Co., Ltd). [f] The reaction was carried out at 130 °C (oil bath).

Reactivities of aryl sulfonium salts and aryl halides

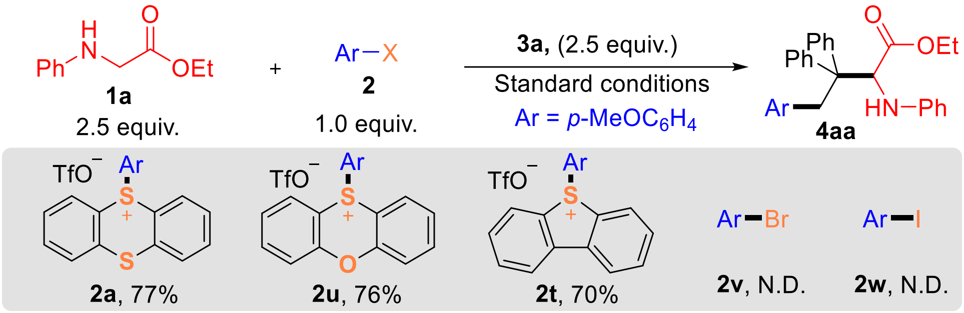

To an oven-dried transparent sample bottle (8 mL) equipped with a magnetic stir bar was added **1a** (0.375 mmol, 2.5 equiv.), **2** (0.15 mmol, 1.0 equiv.), and 4CzIPN (0.0045 mmol, 3 mol%). The tube was sealed and connected to a vacuum line where it was evacuated and back-filled with N_2 three times. Then dry DMSO (4 mL), pyridine (0.15 mmol, 1.0 equiv.), and the **3a** (0.375 mmol, 2.5 equiv.) were added. During the reaction stirring process, the tube was constantly irradiated with a blue LED lamp (462 nm, E27, 15 W) keeping the reaction region located in the center of the LED lamp (2 - 3 cm away, with a cooling fan to keep the reaction temperature at 23 - 25 °C) for 12 hours before quenching with H_2O. After the extraction with ethyl acetate (20 mL X 3), the combined organic layer was washed with brine, dried over Na_2SO_4, filtrated, and concentrated under reduced pressure. The crude product was then purified by column

chromatography on silica gel using petroleum ether and ethyl acetate as the eluent to afford **4aa**.

6.3 Stern-Volmer fluorescence quenching experiments

The UV-Vis absorption spectrum was performed on UV visible spectrophotometer (recorded in anhydrous DMSO in path quartz cuvettes using Lambda 365+ UV-vis spectrophotometer from PerkinElmer).

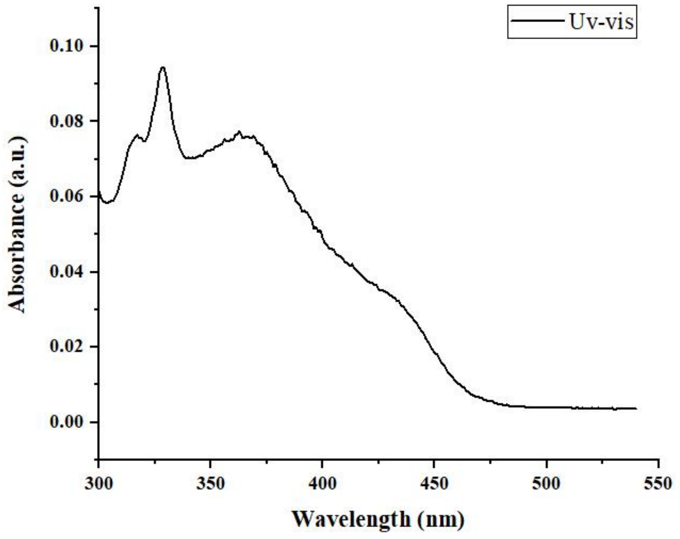

Figure S16. UV-Vis absorption spectrum of **4CzIPN**

Fluorescence spectra was collected on HORIBA Scientific spectrofluorimeter (FouoroMAX-4). Emission spectra of 4CzIPN with excitation at 330 nm in anhydrous DMSO at room temperature.

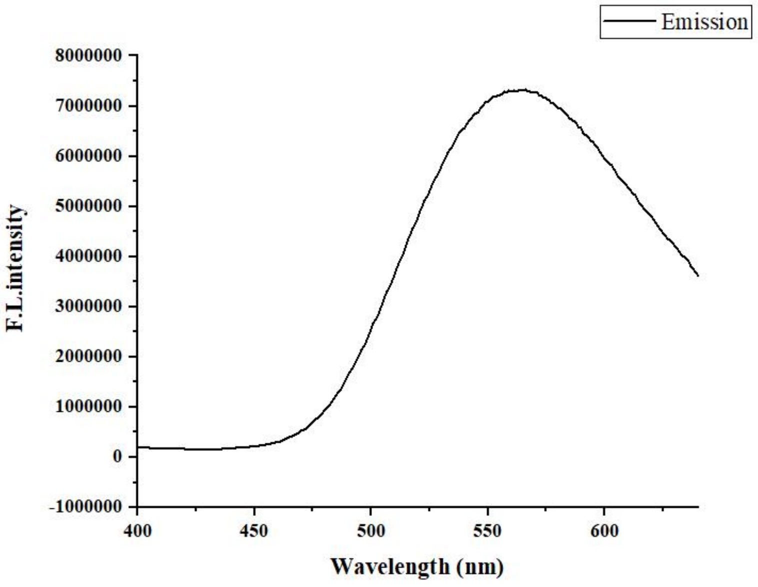

Figure S17. Emission spectra of **4CzIPN**

Stern-Volmer quenching experiments were carried out using a 0.01 mM solution of photocatalyst 4CzIPN and variable concentrations (0.2, 0.4, 0.6, 0.8 mM) of **2a** in anhydrous DMSO. Samples consisting of noted concentration of quencher were prepared at room temperature. The intensity of the emission peak at 560 nm (λ_{ex} = 330 nm).

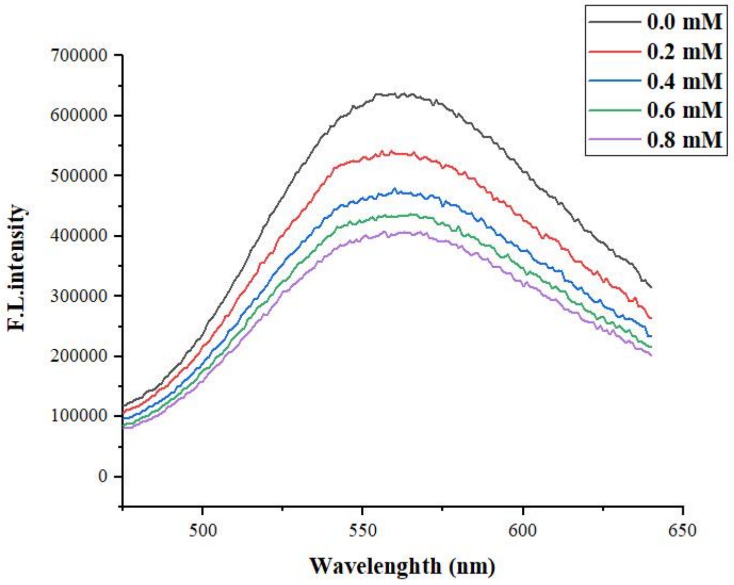

Figure S18. Fluorescence spectra of 4CzIPN with different concentration of **2a**.

Stern-Volmer quenching experiments were carried out using a 0.01 mM solution of photocatalyst 4CzIPN and variable concentrations (0.2, 0.4, 0.6, 0.8 mM) of **[1a + 2a (1a:** 2.5 mM, **2a:** 1.0 mM**)]** in anhydrous DMSO. Samples consisting of noted concentration of quencher were prepared at room temperature. The intensity of the emission peak at 560 nm (λ_{ex} = 330 nm).

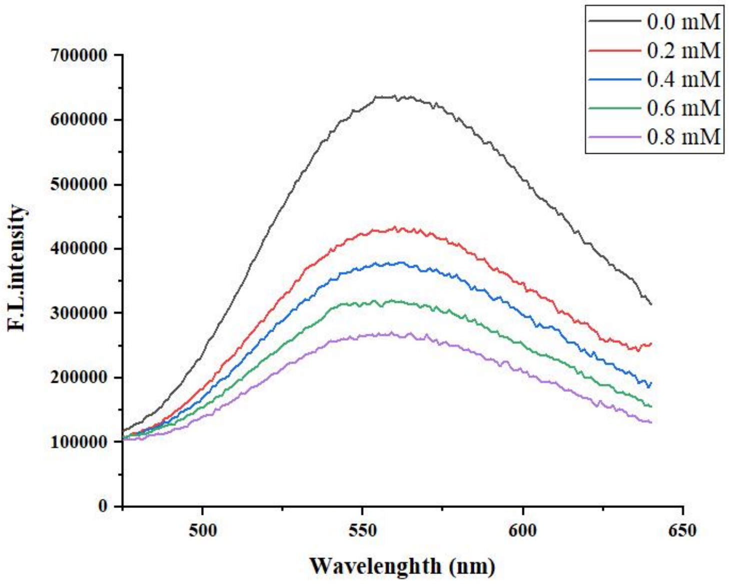

Figure S19. Fluorescence spectra of 4CzIPN with different concentration of [**1a + 2a (1a : 2a = 2.5 : 1)]**

Stern-Volmer quenching experiments were carried out using a 0.01 mM solution of photocatalyst 4CzIPN and 0.8 mM of **2a** and **[1a** + **2a (1a:** 2.0 mM, **2a:** 0.8 mM**)]** in anhydrous DMSO. Samples consisting of noted concentration of quencher were prepared at room temperature. The intensity of the emission peak at 560 nm (λ_{ex} = 330 nm).

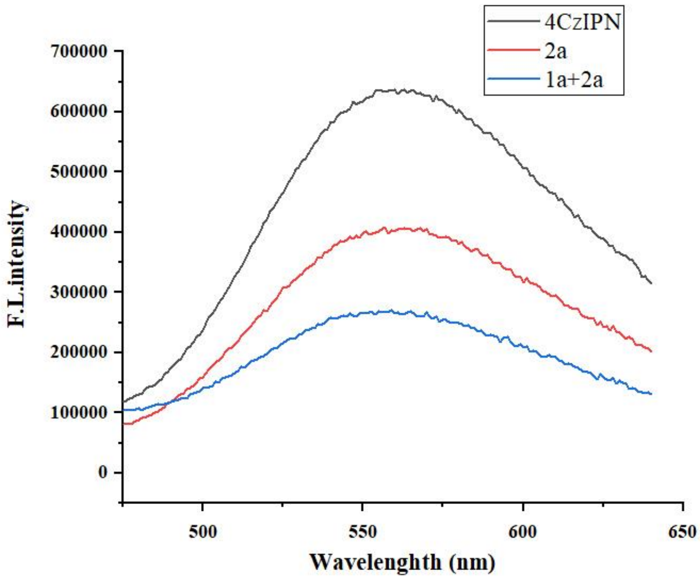

Figure S20. Fluorescence spectra of 4CzIPN with **2a** and [**1a + 2a (1a : 2a = 2.5 : 1)].**

Comparison of different quenchers in Stern-Volmer plot

Fluorescence spectra were collected on HORIBA Scientific spectrofluorimeter (FouoroMAX-4). The intensity of the emission peak at 560 nm (λ_{ex} = 330 nm) expressed as the ratio I_0/I, where I_0 is the emission intensity of 4CzIPN at 560 nm in the absence of a quencher and I is the observed intensity, as a function of the quencher concentration was measured.

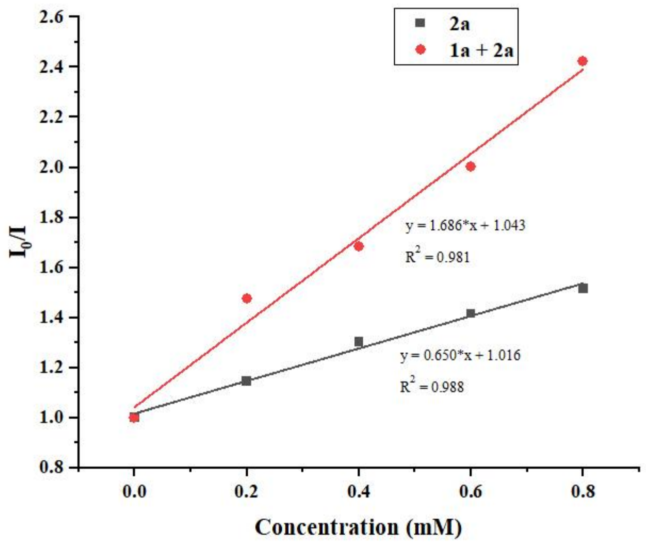

Figure S21. Stern-Volmer plot of 4CzIPN with **2a** and [**1a + 2a (1a : 2a = 2.5 : 1)].**

Stern–Volmer fluorescence quenching experiments illustrated that [**1a + 2a (1a : 2a = 2.5 : 1)]** exhibited a significant fluorescence quenching on the excited state of 4CzIPN.

6.4 UV-Vis absorption spectra

The UV-Vis absorption spectrum was performed on UV visible spectrophotometer (recorded in anhydrous DMSO in path quartz cuvettes using Lambda 365 UV-vis spectrophotometer).

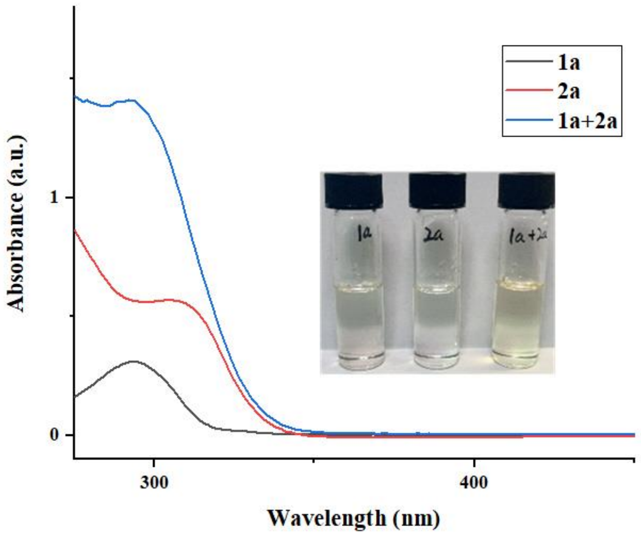

Figure S22. The UV−Vis absorption spectra **1a**, **2a**, and (**1a** + **2a**).

The UV−Vis absorption spectra of the individual reaction components, **1a** (1 × 10^{-1} mM), **2a** (1 × 10^{-1} mM), and [**1a** + **2a**] (1 × 10^{-1} mM, **1a**:**2a** = 1:1) were recorded in anhydrous DMSO. We observed the [**1a** + **2a**] mixture displayed a slight red-shift in absorbance and the absorption was significantly increased, which suggested the formation of an intermediary electron donor-acceptor (EDA) complex between **1a** and **2a** in DMSO. An obvious color change of the mixture solution also explained the formation of EDA complex to some extent.

6.5 Light on-off experiments

According to procedure G for the synthesis of conjugation of amino acid/peptide with aryl thianthrenium salt, six identical reactions for the synthesis of **4ea** are created in parallel and placed in the same environment.

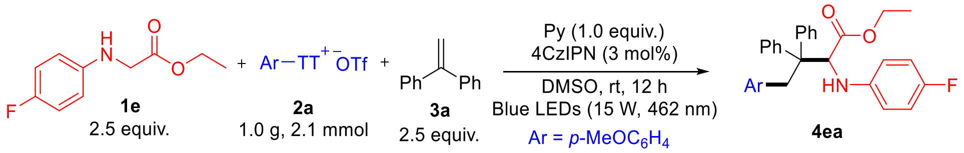

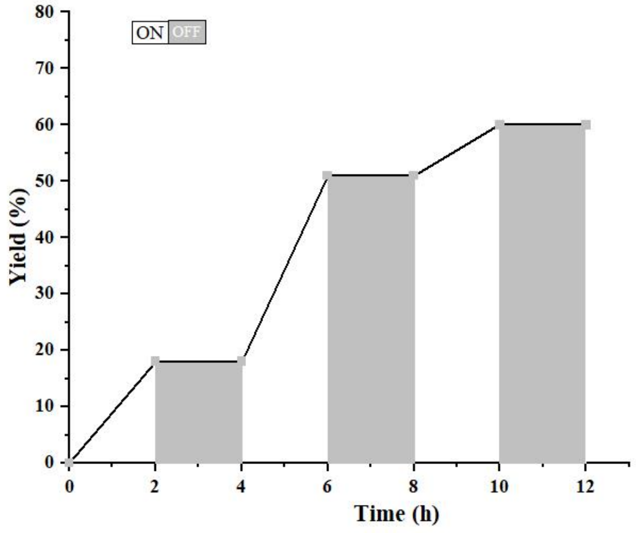

Figure S23. Light on-off experiments.

The light on-off experiments were carried out under the standard reaction conditions using a mixture of **1e** (0.375 mmol, 2.5 equiv.), **2a** (0.15 mmol, 1.0 equiv.), **3a** (0.375 mmol, 2.5 equiv.), Py (0.15 mmol, 1.0 equiv.), and dry DMSO (4 mL). Blue LED lamp (462 nm, E27, 15 W) light was switched on and off at the intervals of 120 minutes, and the reaction mixture was determined by ^{1}F NMR and isolated yield after each period. The graph, shows that the reaction proceeds normally when the light is turned on, and continuous irradiation was essential for the product formation.

6.6 Cyclic Voltammetry measurements

Cyclic voltammetric investigations were performed on the CHI-660E electrochemical workstation (Shanghai Chenhua Instrument Co., Ltd., China). with the conventional three electrode system. A glassy carbon electrode was used as the working electrode, a saturated calomel electrode (SCE) and a platinum wire were used as the reference electrode and counter electrode, respectively. Anisole sulfonium salt **2a** (3.75×10^{-2} M.), and Anisole sulfonium salt **2a** (3.75×10^{-2} M.) + *N*-phenyl-glycinethylester **1a** (9.38×10^{-2} M, **2a** : **1a** = 1:2.5.) were solved in anhydrous DMSO respectively and

experiments were conducted in the presence of Tetrabutylammonium hexafluorophosphate (0.1 M) as supporting electrolyte at a scan rate of 100 mV/s.

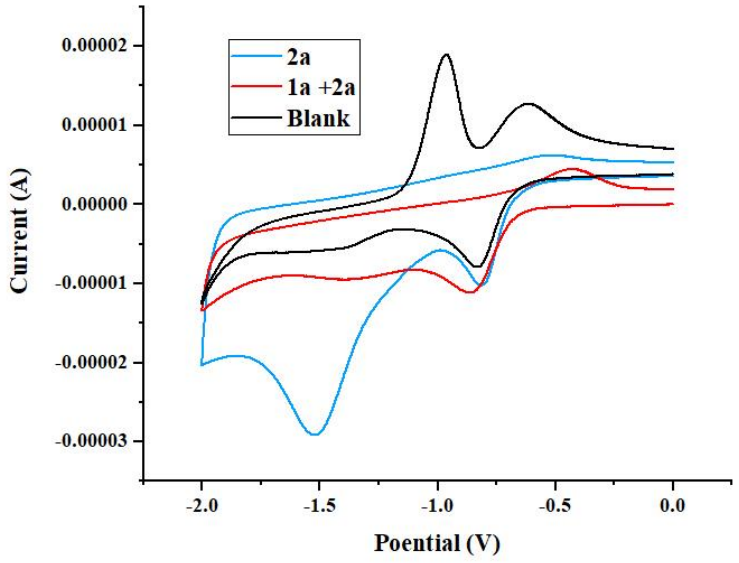

Figure S24. Cyclic Voltammetry measurement of **2a** and **[1a + 2a (1a : 2a = 2.5 : 1)]** in DMSO.

It was found that the reductive potential of thianthrenium salt **2a** was E^{red} = -1.5 V vs SCE in DMSO, indicating improbability of the reduction by reductive photocatalyst specie (E $[4CzIPN/4CzIPN^{\bullet -}]$ = -1.24 V versus SCE in MeCN). However, by mixing **1a** and **2a**, the value shifted to -1.18 V (vs SCE in DMSO), matching the photo catalytical reduction conditions, which was consistent with the observation of an electron donor-acceptor (EDA) complex from **1a** and **2a** by UV−Vis absorption experiments.

7 X-ray Crystallographic Data

7.1 Single-crystal X-ray diffraction measurement for 4ka

Single-crystal X-ray diffraction measurement for **4ka** was carried out at 150 K on

Bruker APEX II CCD diffractometer operating at 50 KV and 30 mA using Mo-*K*α radiation (λ = 0.71073Å). Single-crystal of **4ka** was determined at 213 K on Bruker D8 VENTURE diffractometer with a PHOTON 100 CMOS detector equipped with METALJET-X-ray Source (Ga, λ = 1.34138 Å). Crystals were mounted on a loop using Parabar 10312 oil for data collection. Data was collected with a series of φ and/or ω scans. Data was integrated using SAINT and scaled with either a numerical or multi-scan absorption correction using SADABS. Structures were solved using SHELXT and refined by full-matrix least squares on F^2 using the SHELXL and OLEX2 program. All non-hyd.r.ogen atoms were refined anisotropically, and all hyd.r.ogen atoms were added in idealized positions and refined using a riding model. The atomic coordinates and structure factors have been deposited in the Cambridge Structural Database (CSD) of the Cambridge Crystallographic Data Centre (CCDC), https://www.ccdc.cam.ac.uk/structures/ [CCDC number 2269817 for **4ka**].

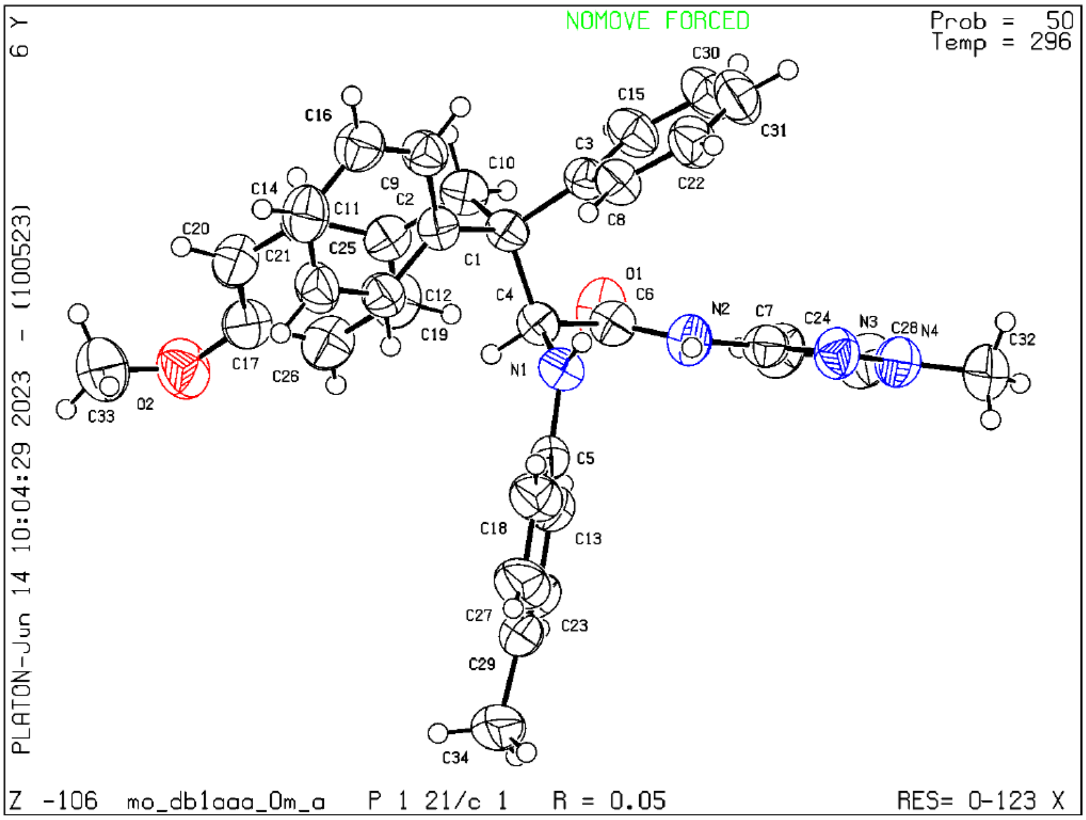

Figure S25. X-Ray crystallographic spectrum of **4ka** (50% thermal ellipsoids).

Supporting Information

Table S13. Crystal data and structure refinement for **4ka**

Identification code	2269817
Empirical formula	$C_{34}H_{34}N_4O_2$
Formula weight	530.65
Temperature/K	296.15
Crystal system	monoclinic
Space group	$P2_1/c$
a/Å	16.0510(19)
b/Å	9.7026(11)
c/Å	18.748(2)
α/°	90
β/°	91.177(3)
γ/°	90
Volume/Å^3	2919.1(6)
Z	4
ρ_{calc}g/cm^3	1.207
μ/mm^{-1}	0.076
F(000)	1128.0
Crystal size/mm^3	0.2 × 0.2 × 0.1
Radiation	MoKα (λ = 0.71073)
2Θ range for data collection/°	4.346 to 55.164
Index ranges	-20 ≤ h ≤ 20, -12 ≤ k ≤ 12, -24 ≤ l ≤ 24
Reflections collected	24979
Independent reflections	6738 [R_{int} = 0.02418, R_{sigma} = 0.0394]
Data/restraints/parameters	6738/0/364
Goodness-of-fit on F^2	1.021
Final R indexes [I>=2σ (I)]	R_1 = 0.0489, wR_2 = 0.1237

Final R indexes [all data]	R_1 = 0.0879, wR_2 = 0.1460
Largest diff. peak/hole / e Å^{-3}	0.40/-0.34

7.2 Single-crystal X-ray diffraction measurement for 6jg

Single-crystal X-ray diffraction measurement for **6jg** was carried out at 150 K on Bruker APEX II CCD diffractometer operating at 50 KV and 30 mA using Mo-*K*α radiation (λ = 0.71073Å). Single-crystal of **6jg** was determined at 213 K on Bruker D8 VENTURE diffractometer with a PHOTON 100 CMOS detector equipped with METALJET-X-ray Source (Ga, λ = 1.34138 Å). Crystals were mounted on a loop using Parabar 10312 oil for data collection. Data was collected with a series of φ and/or ω scans. Data was integrated using SAINT and scaled with either a numerical or multi-scan absorption correction using SADABS. Structures were solved using SHELXT and refined by full-matrix least squares on F^2 using the SHELXL and OLEX2 program. All non-hyd.r.ogen atoms were refined anisotropically, and all hyd.r.ogen atoms were added in idealized positions and refined using a riding model. The atomic coordinates and structure factors have been deposited in the Cambridge Structural Database (CSD) of the Cambridge Crystallographic Data Centre (CCDC), https://www.ccdc.cam.ac.uk/structures/ [CCDC number 2358315 for **6jg**].

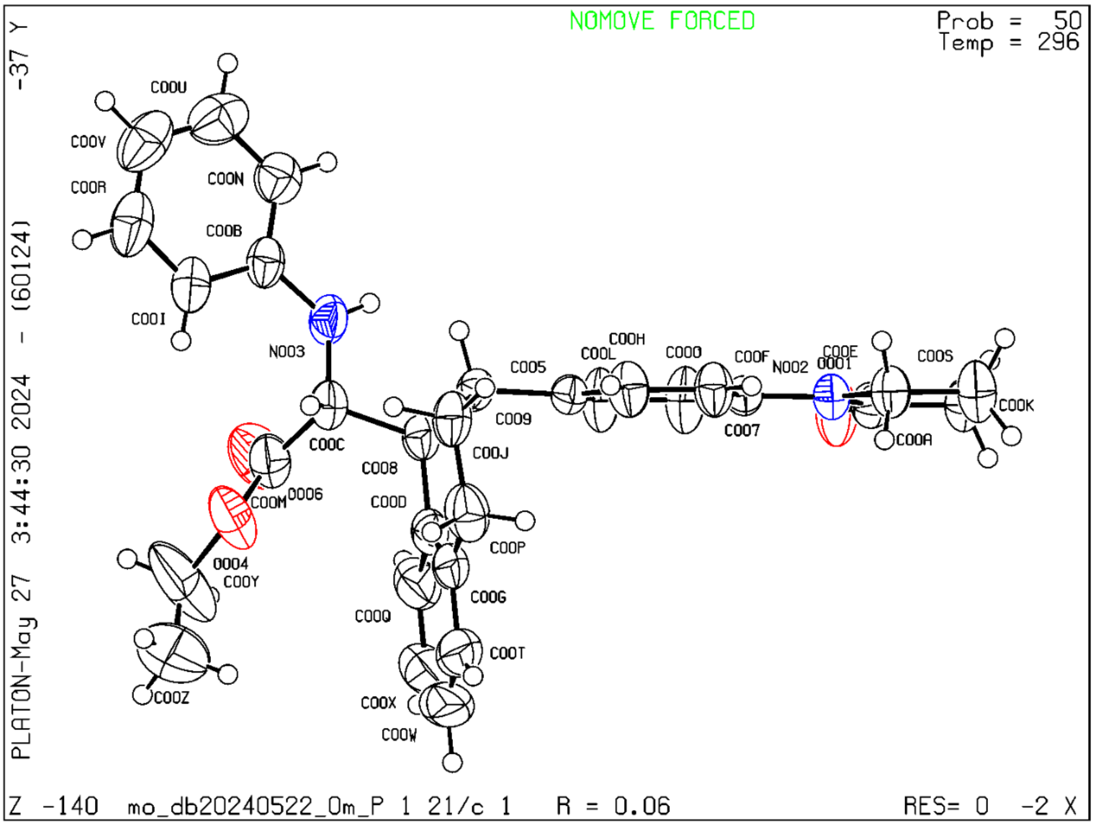

Figure S26. X-Ray crystallographic spectrum of **6jg** (50% thermal ellipsoids).

Table S14. Crystal data and structure refinement for **6jg**

Identification code	2358315
Empirical formula	$C_{30}H_{32}N_2O_3$
Formula weight	468.57
Temperature/K	296.15
Crystal system	monoclinic
Space group	$P2_1/c$
a/Å	9.435(3)
b/Å	27.713(8)
c/Å	10.134(3)
α/°	90
β/°	110.134(4)
γ/°	90

Volume/Å^3	2483.2(13)
Z	4
ρ_{calc}g/cm^3	1.253
μ/mm^{-1}	0.081
F(000)	1000.0
Crystal size/mm^3	0.04 × 0.02 × 0.02
Radiation	MoKα (λ = 0.71073)
2Θ range for data collection/°	4.346 to 55.164
Index ranges	-11 ≤ h ≤ 11, -31 ≤ k ≤ 34, -12 ≤ l ≤ 11
Reflections collected	13199
Independent reflections	2018 [R_{int} = 0.0284, R_{sigma} = 0.0389]
Data/restraints/parameters	5018/0/305
Goodness-of-fit on F^2	1.032
Final R indexes [I>=2σ (I)]	R_1 = 0.0588, wR_2 = 0.1375
Final R indexes [all data]	R_1 = 0.1058, wR_2 = 0.1612
Largest diff. peak/hole / e Å^{-3}	0.32/-0.26

8 References

(1) Z. Yang, J. Liu, L.-G. Xie, *Adv. Sci*. **2024**, 2402428.

(2) E. J. Moore, V. Bajaj, P. Steck, R. Fasan, *J. Org. Chem*. **2018**, *83*, 7480.

(3) Y. Gao, J. Liu, C. Wei, Y. Li, K. Zhang, L. Song, L. Cai, *Nat. Commun*. **2022**, *13*, 7450.

(4) S. Zhong, M. Nieger, A. Bihlmeier, M. Shi, S. Bräse, *Org. Biomol. Chem*. **2014**, *12*, 3265.

(5) a) W. Qi, S. Gu, L.-G. Xie, *Org. Let.* **2024**, *26*, 728; b) F. Ye, F. Berger, H. Jia, J. Ford, A. Wortman, J. Bçrgel, C. Genicot, T. Ritter, *Angew. Chem. Int. Ed.* **2019**, *58*, 14615; c) J. Wu, Z. Wang, X.-Y. Chen, Y. Wu, D. Wang, Q. Peng, P. Wang, *Sci. China Chem.* **2020**, *63*, 336; d) S. Tang, X. Zhao, L. Yang, B. Li, B. Wang, *Angew. Chem. Int. Ed.* **2022**, *61*, e202212975; e) R. A. Roberts, B. E. Metze, A. Nilova, D. R. Stuart, *J. Am. Chem. Soc.* **2023**, *145*, 3306; f) F. Juliá, Q. Shao, M. Duan, M. B. Plutschack, F. Berger, J. Mateos, C. Lu, X.-S. Xue, K. N. Houk, T. Ritter, *J. Am. Chem. So*c. **2021**, *143*, 16041; g) M. Wang, X. Zhang, M. Ma, B. Zha, *Org. Lett*, **2022**, *24*, 6031; h) K. Kafuta, A. Korzun, M. Böhm, C. Golz, M. Alcarazo, *Angew. Chem. Int. Ed.* **2020**, *59*, 1950; i) Z.-W. Cao, J.-X. Zhang, J.-T. Wang, L. Li, X.-Y. Chen, S.-N. Jin, Z.-Y. Cao, P. Wang, *Org. Lett.* **2024**, *26*, 6681.

(6) a) P. Xu, S. Wang, H. Xu, Y.-Q. Liu, R.-B. Li, W.-W. Liu, X.-Y. Wang, M.-L. Zou, Y. Zhou, D. Guo, X. Zhu, *ACS Catal.* **2023**, *13*, 2149; b) J. C. L. Walker, M. Oestreich, *Org. Lett*. **2018**, *20*, 6411.

(7) L. Song, W. Wang, J.-P. Yue, Y.-X. Jiang, M.-K. Wei, H.-P. Zhang, S.-S. Yan, L.-L. Liao, D.-G. Yu, *Nat. Catal*. **2022**, *5*, 832.

(8) J. Xu, J.-W. Liu, R. Wang, J. Yang, K.-K. Zhao, H.-J. Xu, *ACS Catal*. **2023**, *13*, 7339.

(9) E. Speckmeier, T. G. Fischer, K. Zeitler, *J. Am. Chem. Soc*. **2018**, *140*, 15353.

(10) K. Gadde, P. Mampuys, A. Guidetti, H. Y. Vincent Ching, W. A. Herrebout, S. Van Doorslaer, K. Abbaspour Tehrani, B. U. W. Maes, *ACS Catal*. **2020**, *10*, 8765.

(11) M. K. Bogdos, E. Pinard, J. A. Murphy, *Beilstein J. Org. Chem*. **2018**, *14*, 2035.

(12) M. Reckenthälera, A. G. Griesbeck, *Adv. Synth. Catal*. **2013**, *355*, 2727.

(13) C. Prier, D. Rankic, D. MacMillan, *Chem. Rev*. **2013**, *113*, 5322.

(14) W. Lee, Y. Koo, H. Jung, S. Chang, S. Hong, *Nat. Chem.* **2023**, *15*, 1091.

(15) Q.-Q. Zhou, Y.-Q. Zou, L.-Q. Lu, W.-G. Xiao, *Angew. Chem. Int. Ed*. **2019**, *58*, 1586.

(16) A. Joshi-Pangu, F. Lévesque, H. G. Roth, S. F. Oliver, L.-C. Campeau, D. Nicewicz, D. A. DiRocco, *J. Org. Chem*. **2016**, *81*, 7244.

(17) J. Luo, J. Zhang, *ACS Catal*. **2016**, *6*, 873.

(18) M. Zhu, X. Zhang**,** C. Zheng**,** S.-L. You, *Acc. Chem. Res*. **2022**, *55*, 2510.

9 NMR Spectra

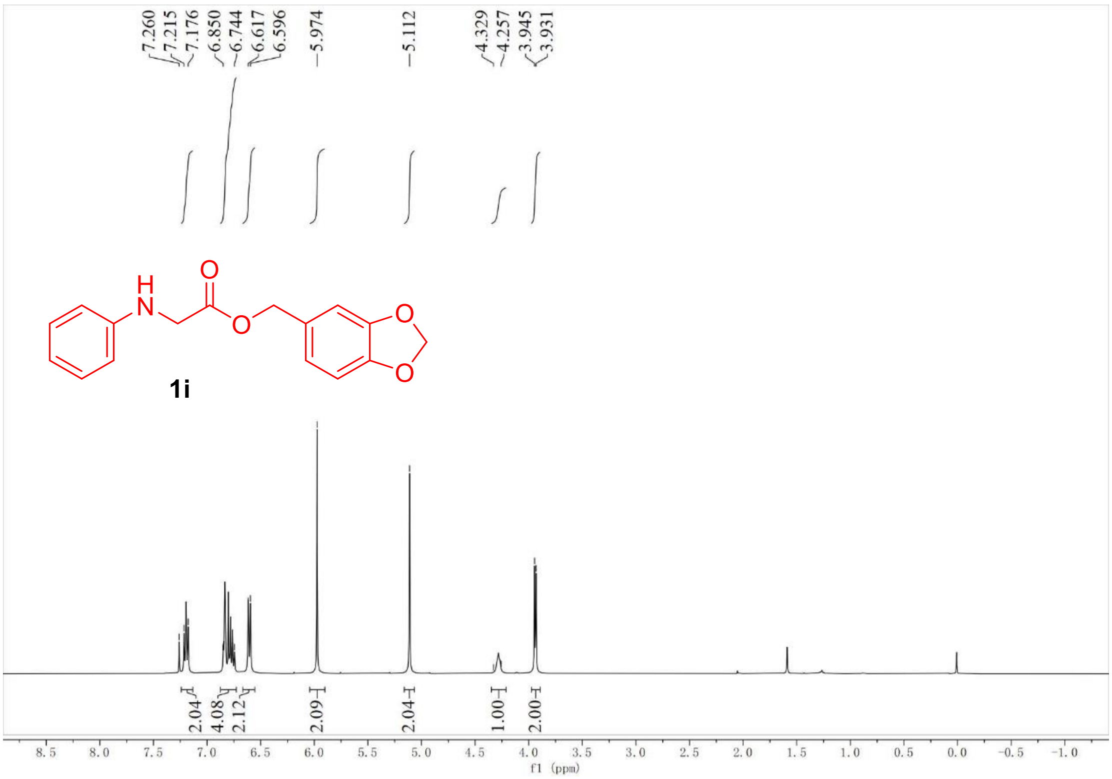

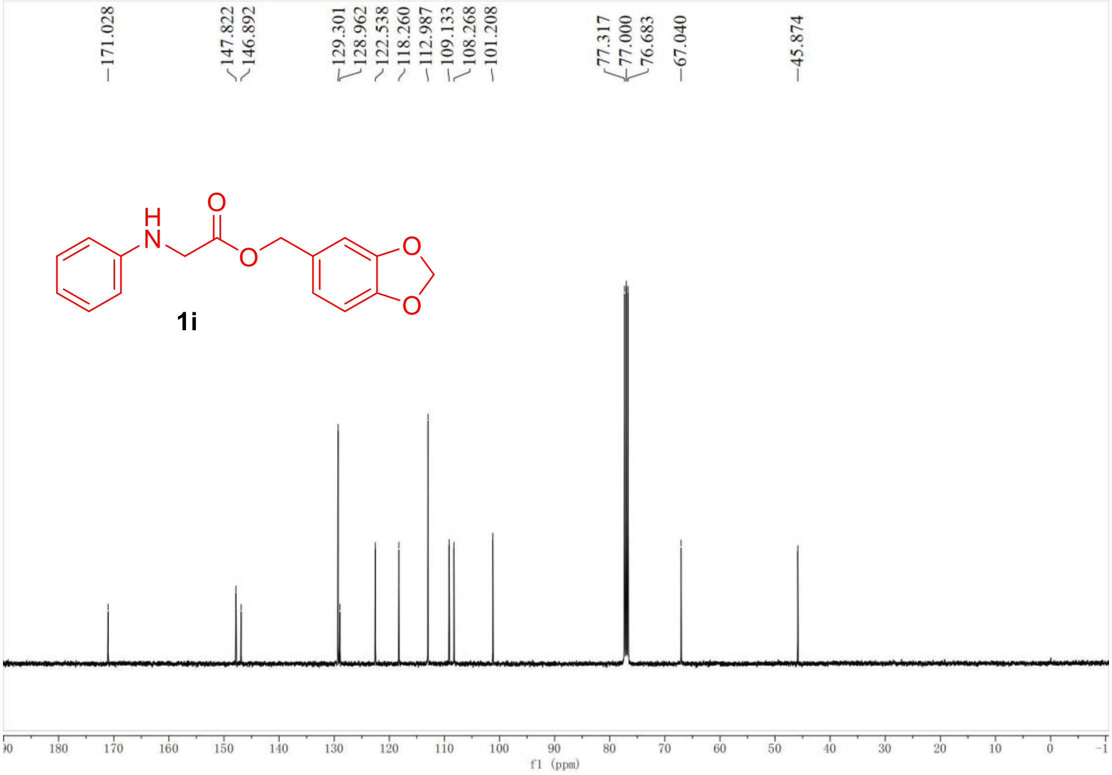

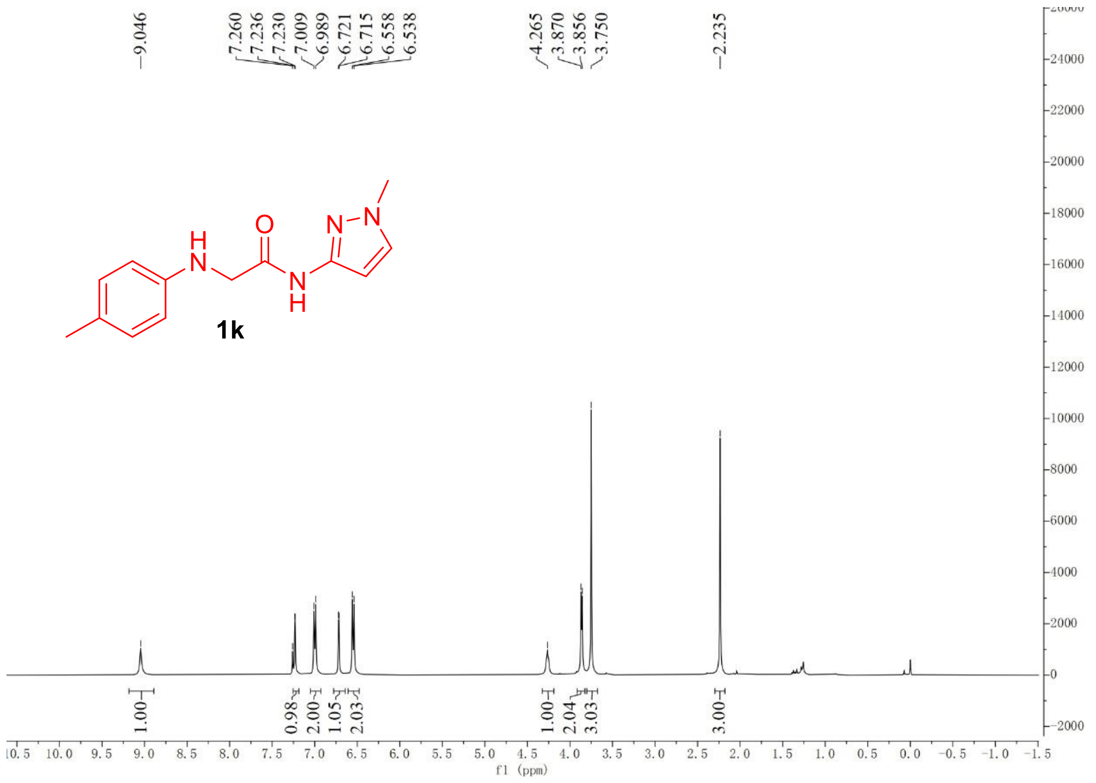
9.046
7.260
7.236
7.230
7.009
6.989
6.721
6.715
6.558
6.538
4.265
3.870
3.856
3.750
2.235
1k
1.00
0.98
2.00
1.05
2.03
1.00
2.04
3.03
3.00
f1 (ppm)

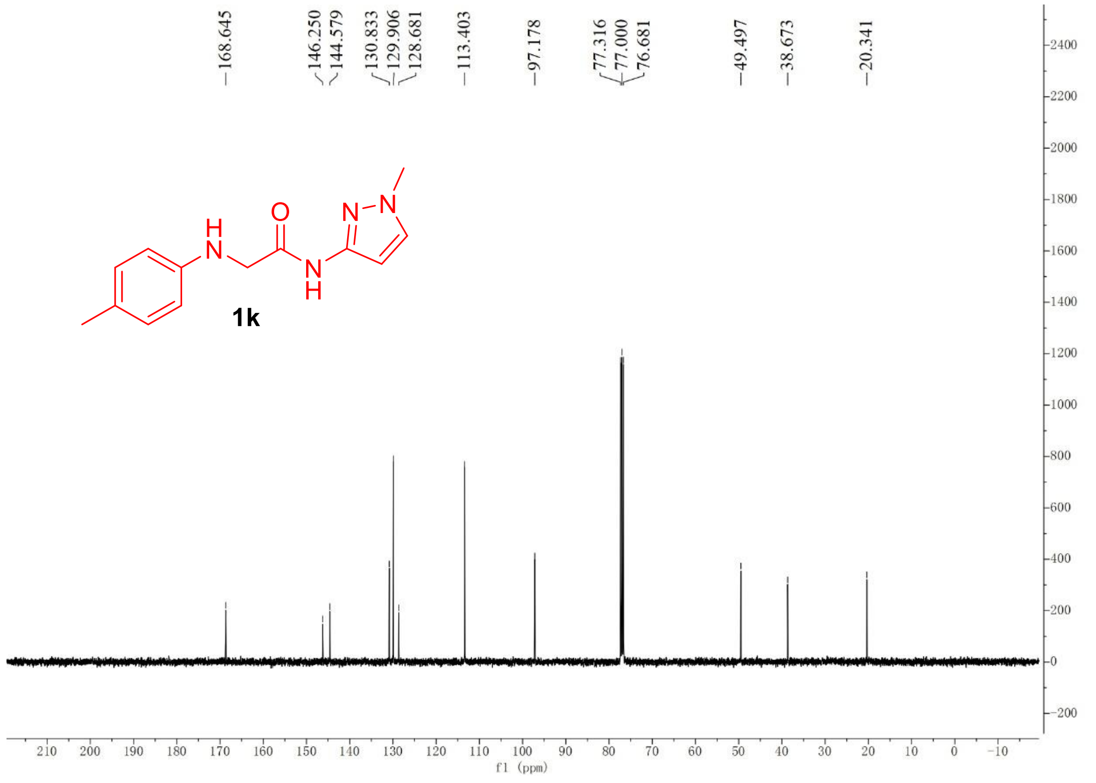
168.645
146.250
144.579
130.833
129.906
128.681
113.403
97.178
77.316
77.000
76.681
49.497
38.673
20.341
1k
f1 (ppm)

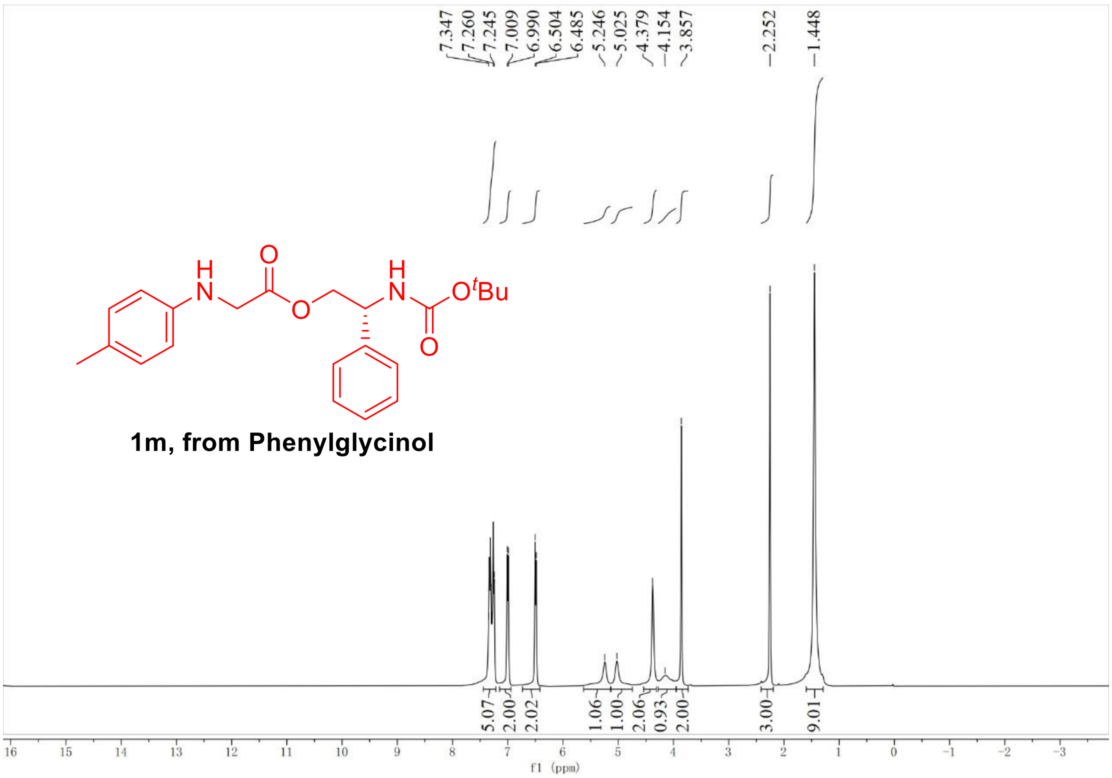
7.347
7.260
7.245
7.009
6.990
6.504
6.485
5.246
5.025
4.379
4.154
3.857
2.252
1.448
1m, from Phenylglycinol
5.07
2.00
2.02
1.06
1.00
2.06
0.93
2.00
3.00
9.01
f1 (ppm)

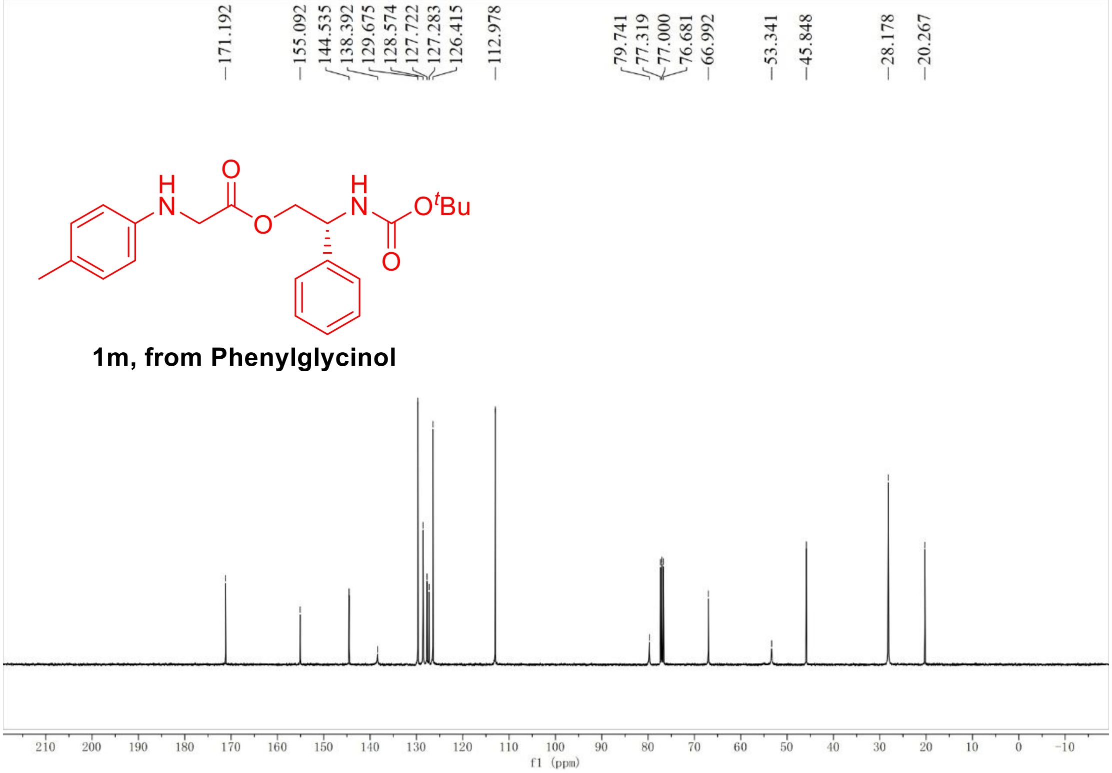
171.192
155.092
144.535
138.392
129.675
128.574
127.722
127.283
126.415
112.978
79.741
77.319
77.000
76.681
66.992
53.341
45.848
28.178
20.267
1m, from Phenylglycinol
f1 (ppm)

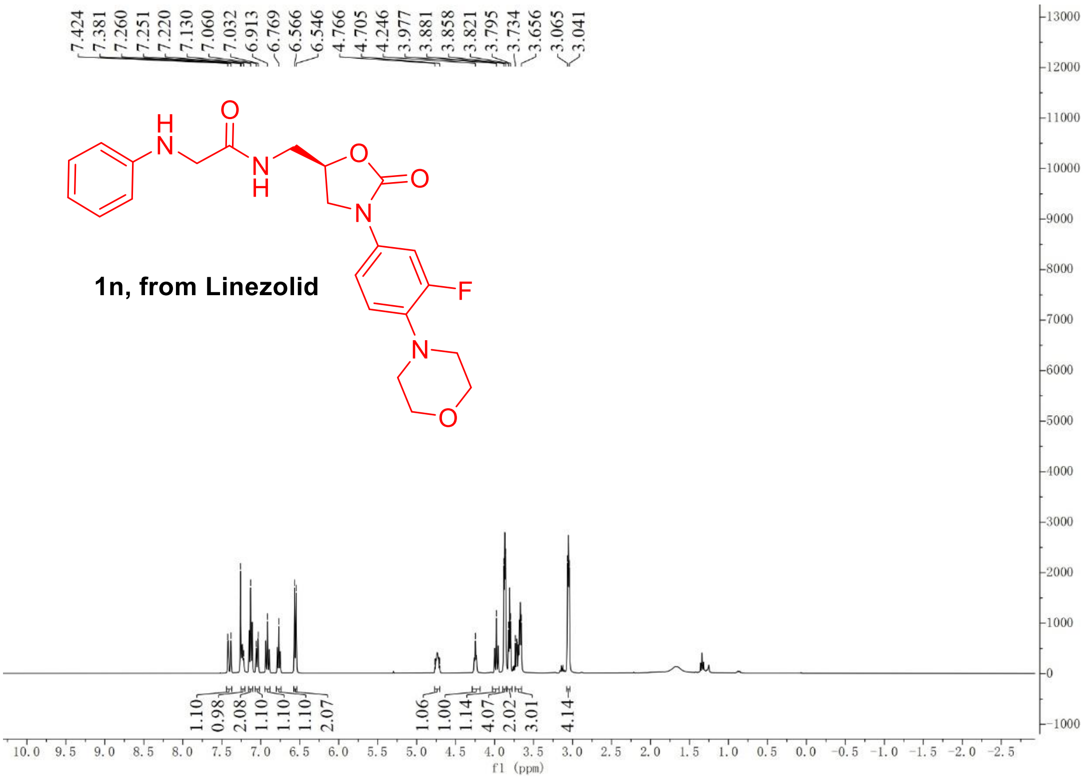

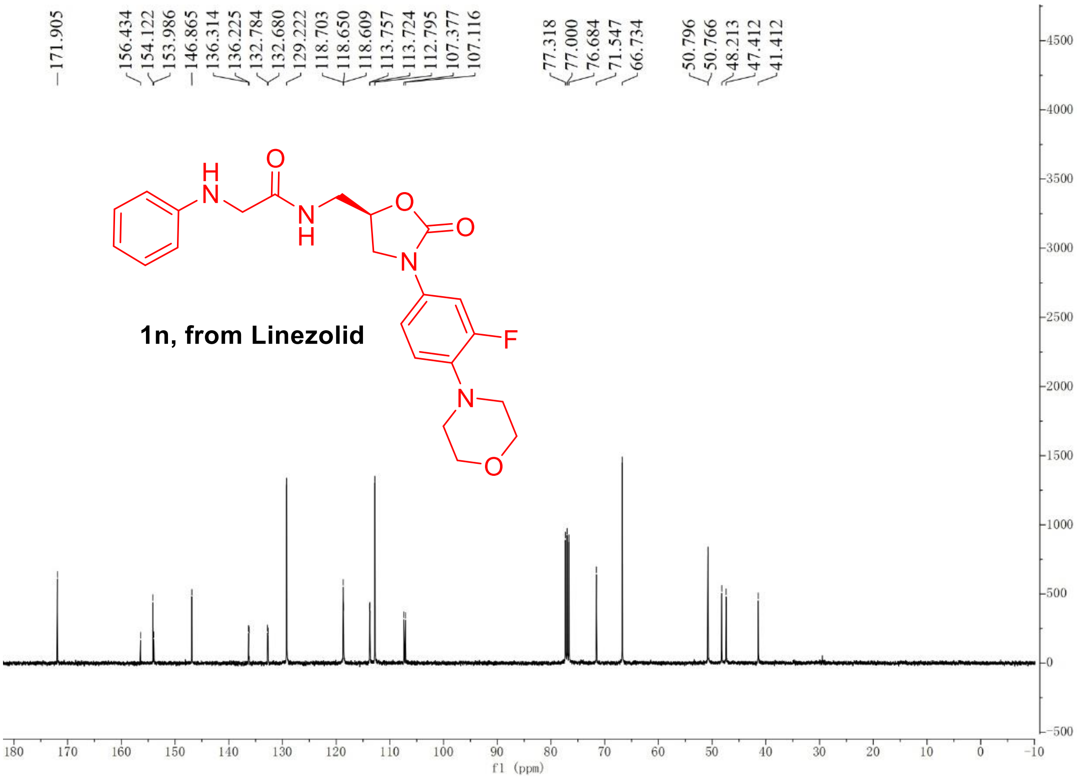

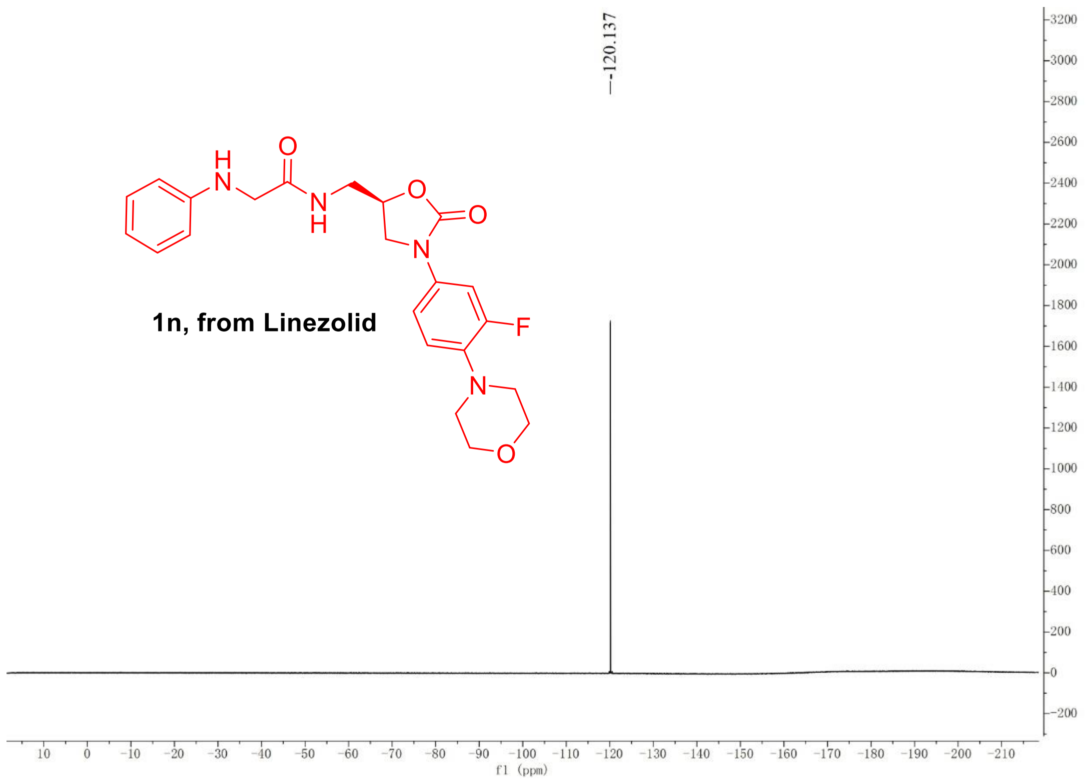
-120.137
1n, from Linezolid
f1 (ppm)

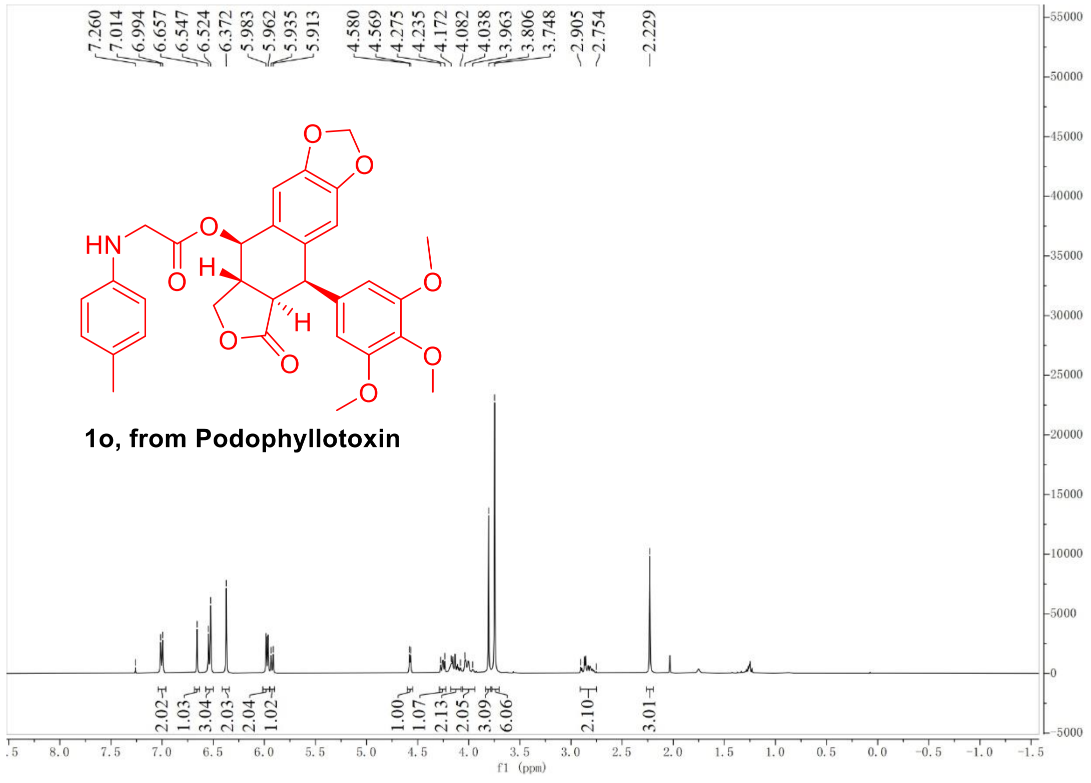
1o, from Podophyllotoxin
f1 (ppm)

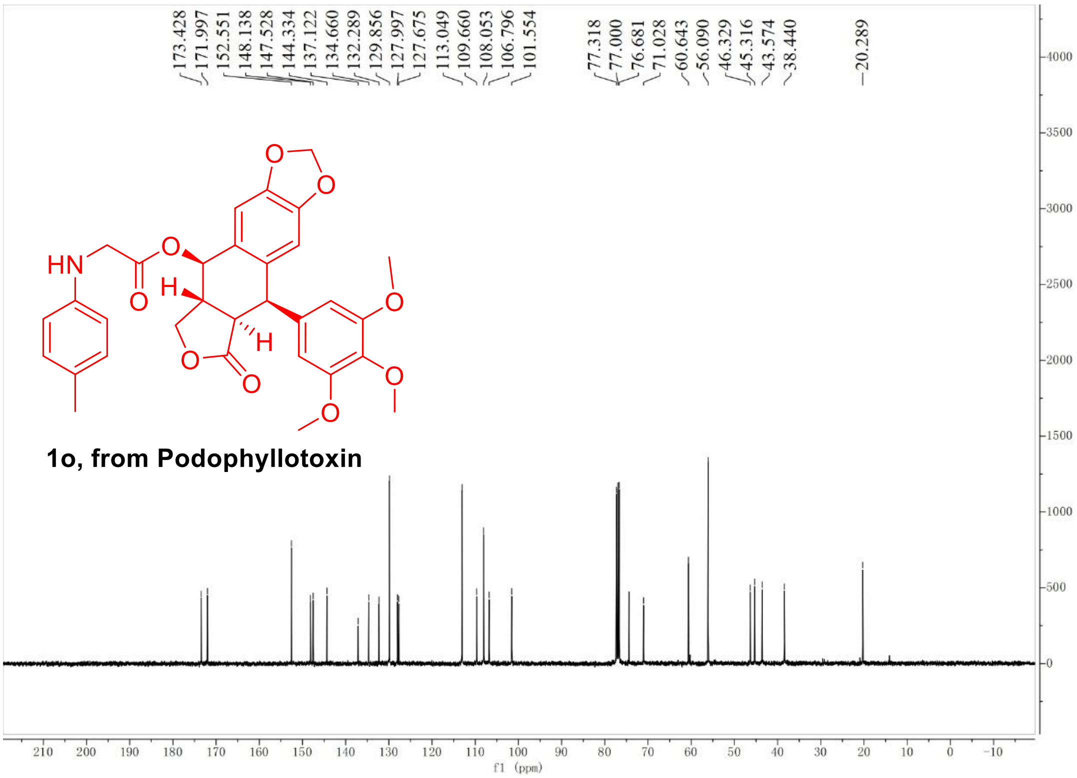
173.428
171.997
152.551
148.138
147.528
144.334
137.122
134.660
132.289
129.856
127.997
127.675
113.049
109.660
108.053
106.796
101.554
77.318
77.000
76.681
71.028
60.643
56.090
46.329
45.316
43.574
38.440
20.289
HN
O
O
H
O
O
H
O
O
O
O
1o, from Podophyllotoxin
f1 (ppm)

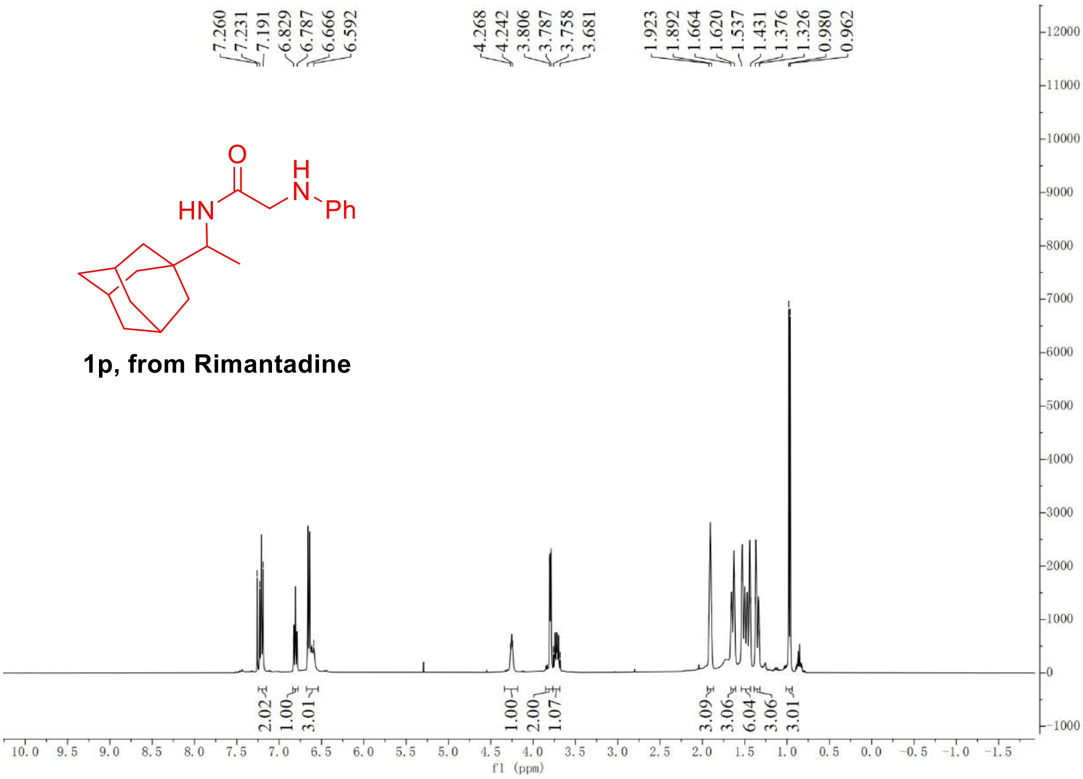
7.260
7.231
7.191
6.829
6.787
6.666
6.592
4.268
4.242
3.806
3.787
3.758
3.681
1.923
1.892
1.664
1.620
1.537
1.431
1.376
1.326
0.980
0.962
O
H
N
Ph
HN
1p, from Rimantadine
2.02
1.00
3.01
1.00
2.00
1.07
3.09
3.06
6.04
3.06
3.01
f1 (ppm)

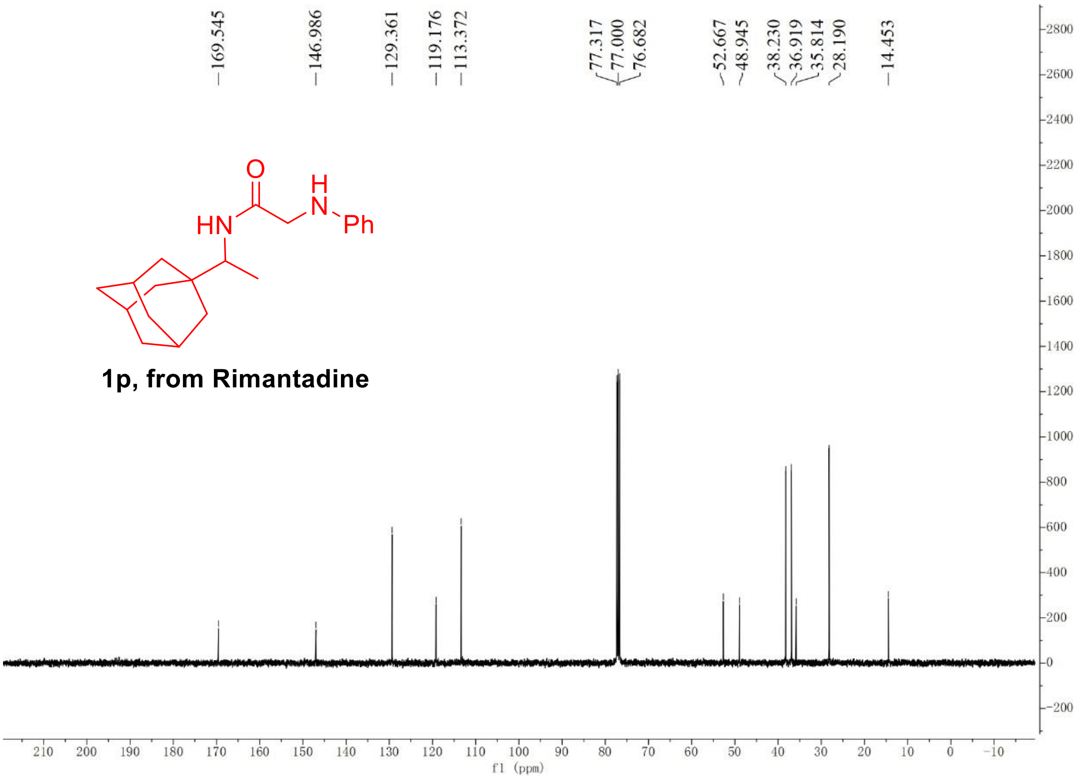
169.545
146.986
129.361
119.176
113.372
77.317
77.000
76.682
52.667
48.945
38.230
36.919
35.814
28.190
14.453
HN
O
H
N
Ph
1p, from Rimantadine
f1 (ppm)

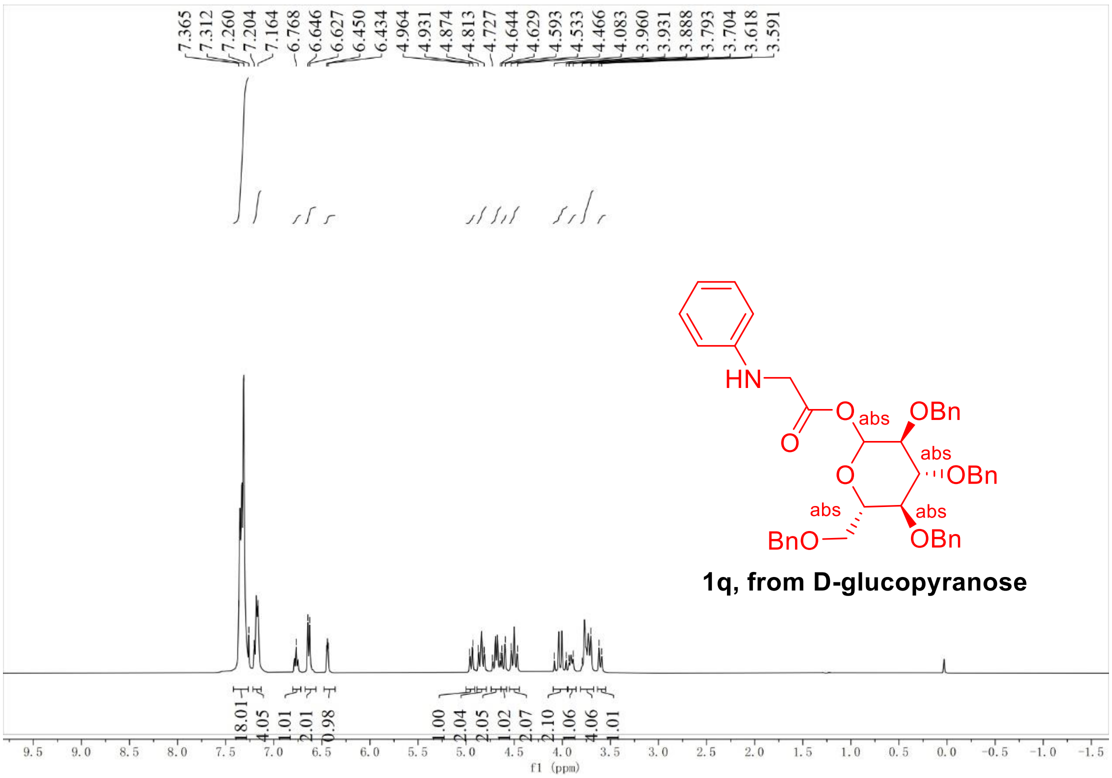
7.365
7.312
7.260
7.204
7.164
6.768
6.646
6.627
6.450
6.434
4.964
4.931
4.874
4.813
4.727
4.644
4.629
4.593
4.533
4.466
4.083
3.960
3.931
3.888
3.793
3.704
3.618
3.591
HN
O
O
abs
OBn
OBn
BnO
OBn
1q, from D-glucopyranose
18.01
4.05
1.01
2.01
0.98
1.00
2.04
2.05
1.02
2.07
2.10
1.06
4.06
1.01
f1 (ppm)

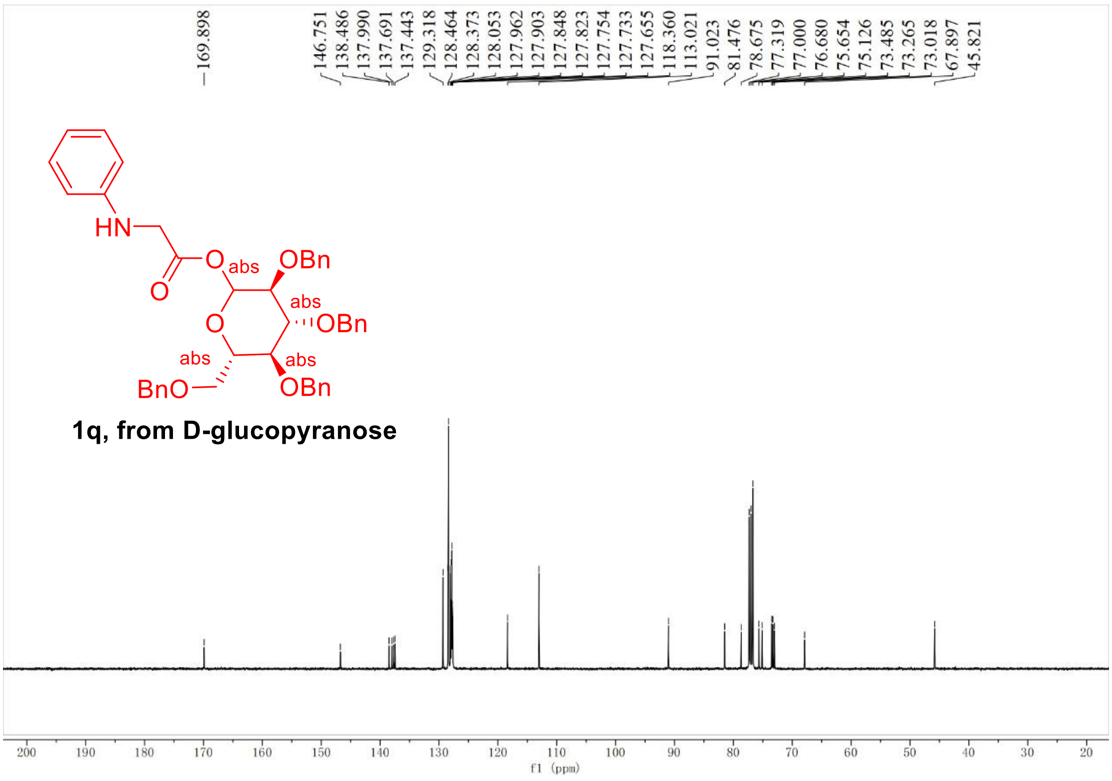
169.898
146.751
138.486
137.990
137.691
137.443
129.318
128.464
128.373
128.053
127.962
127.903
127.848
127.823
127.754
127.733
127.655
118.360
113.021
91.023
81.476
78.675
77.319
77.000
76.680
75.654
75.126
73.485
73.265
73.018
67.897
45.821
HN
O
abs
OBn
abs
OBn
abs
abs
BnO
OBn
1q, from D-glucopyranose
f1 (ppm)

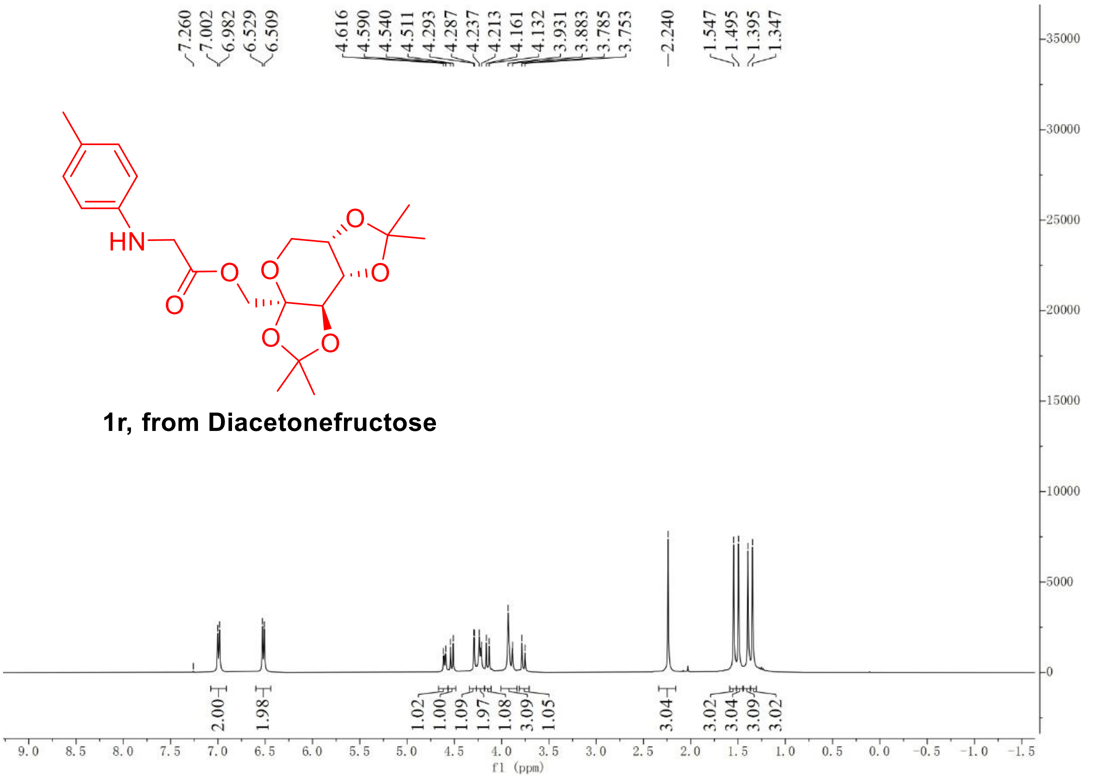
7.260
7.002
6.982
6.529
6.509
4.616
4.590
4.540
4.511
4.293
4.287
4.237
4.213
4.161
4.132
3.931
3.883
3.785
3.753
2.240
1.547
1.495
1.395
1.347
HN
O
1r, from Diacetonefructose
2.00
1.98
1.02
1.00
1.09
1.97
1.08
3.09
1.05
3.04
3.02
3.04
3.09
3.02
f1 (ppm)

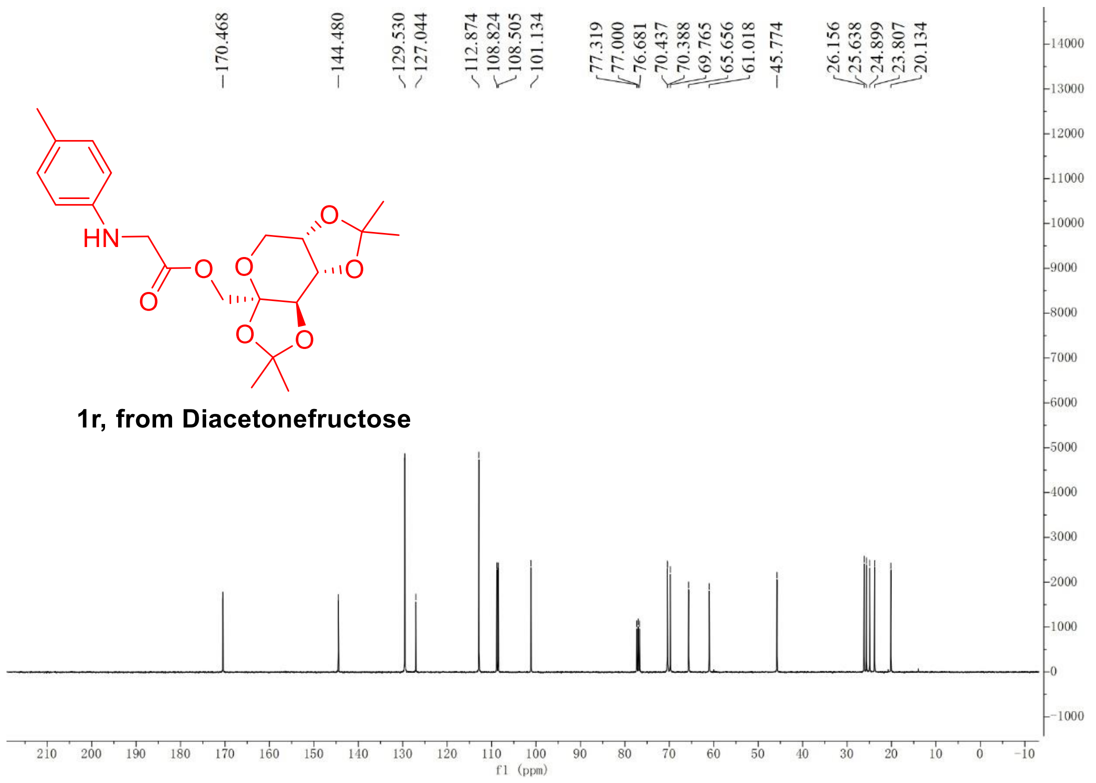
170.468
144.480
129.530
127.044
112.874
108.824
108.505
101.134
77.319
77.000
76.681
70.437
70.388
69.765
65.656
61.018
45.774
26.156
25.638
24.899
23.807
20.134
HN
O
1r, from Diacetonefructose
f1 (ppm)

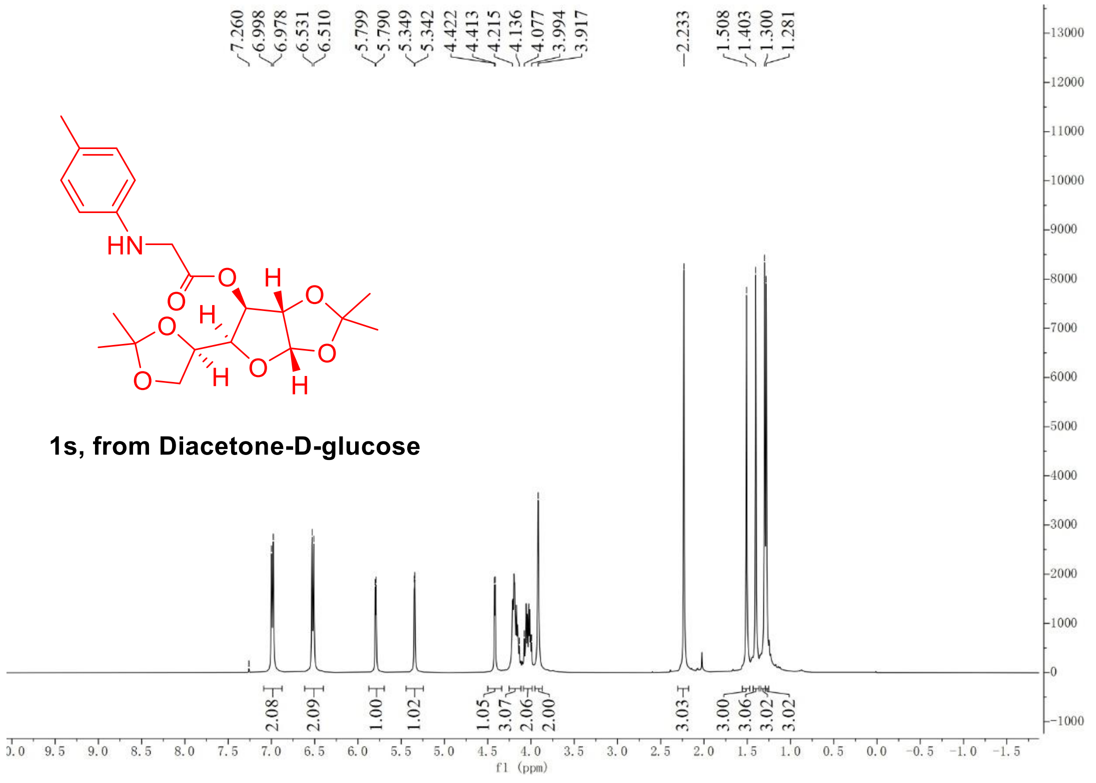
7.260
6.998
6.978
6.531
6.510
5.799
5.790
5.349
5.342
4.422
4.413
4.215
4.136
4.077
3.994
3.917
2.233
1.508
1.403
1.300
1.281
HN
O
H
1s, from Diacetone-D-glucose
2.08
2.09
1.00
1.02
1.05
3.07
2.06
2.00
3.03
3.00
3.06
3.02
3.02
f1 (ppm)

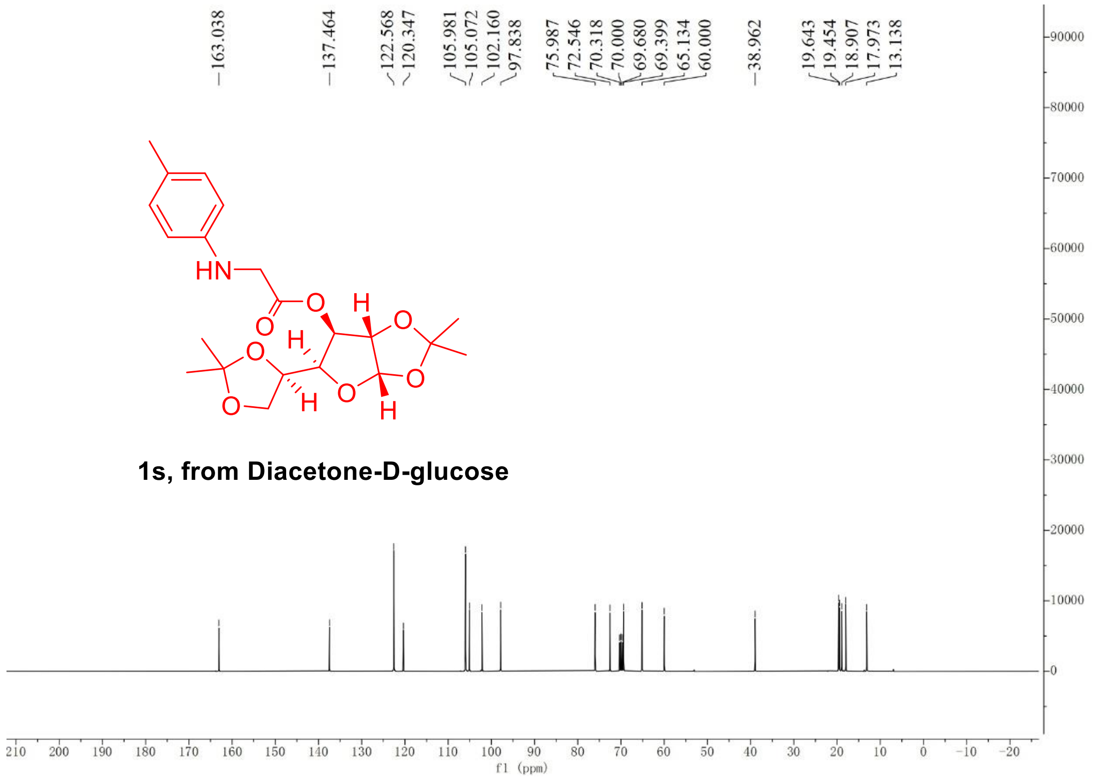
163.038
137.464
122.568
120.347
105.981
105.072
102.160
97.838
75.987
72.546
70.318
70.000
69.680
69.399
65.134
60.000
38.962
19.643
19.454
18.907
17.973
13.138
HN
O
O
H
O
O
H
O
O
H
O
H
1s, from Diacetone-D-glucose
f1 (ppm)

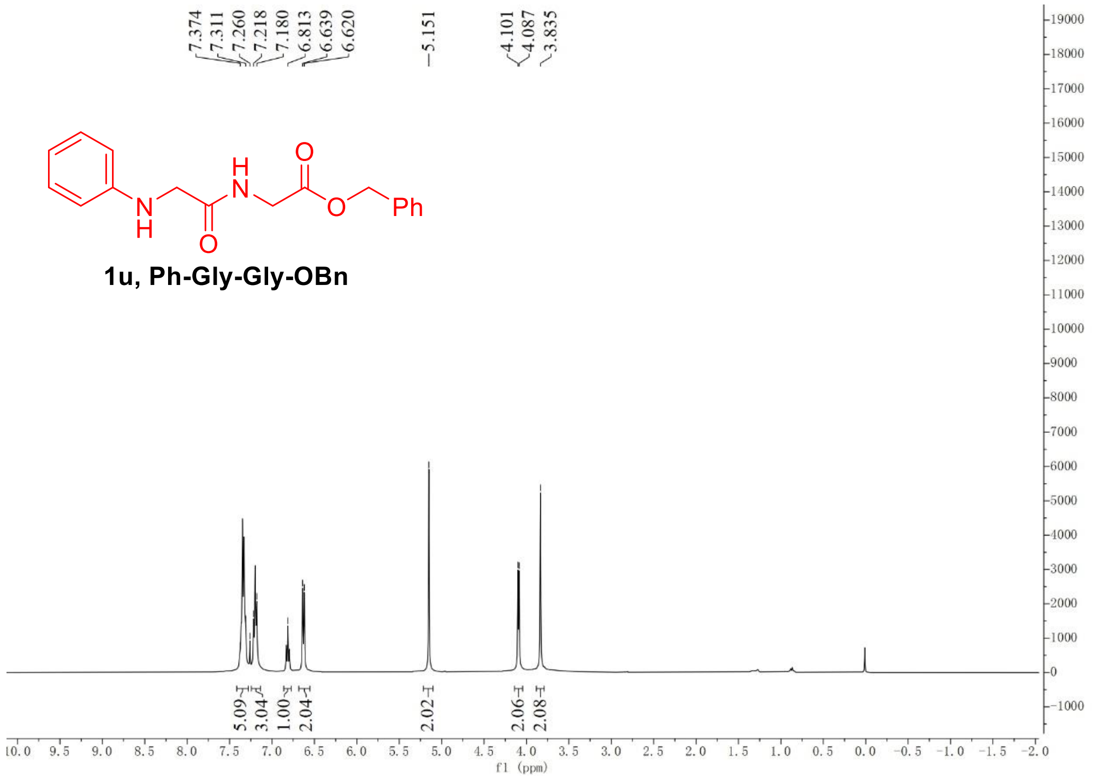
7.374
7.311
7.260
7.218
7.180
6.813
6.639
6.620
5.151
4.101
4.087
3.835
N
H
H
N
O
O
O
Ph
1u, Ph-Gly-Gly-OBn
5.09
3.04
1.00
2.04
2.02
2.06
2.08
f1 (ppm)

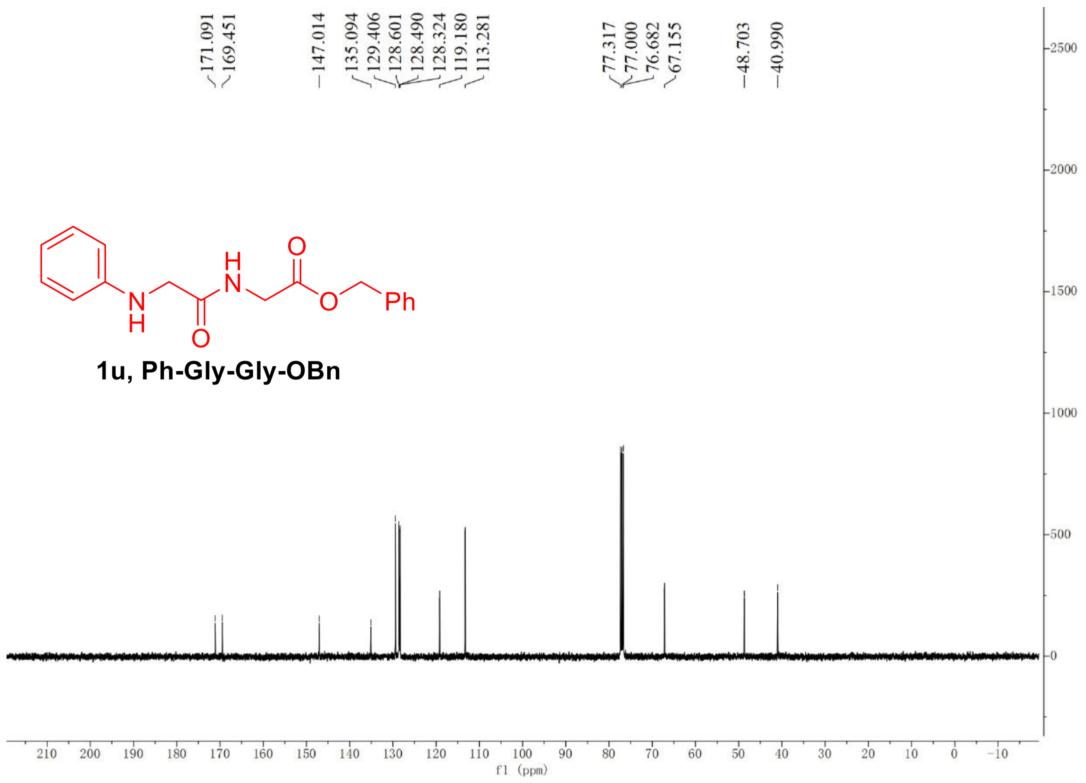
171.091
169.451
147.014
135.094
129.406
128.601
128.490
128.324
119.180
113.281
77.317
77.000
76.682
67.155
48.703
40.990
1u, Ph-Gly-Gly-OBn
f1 (ppm)

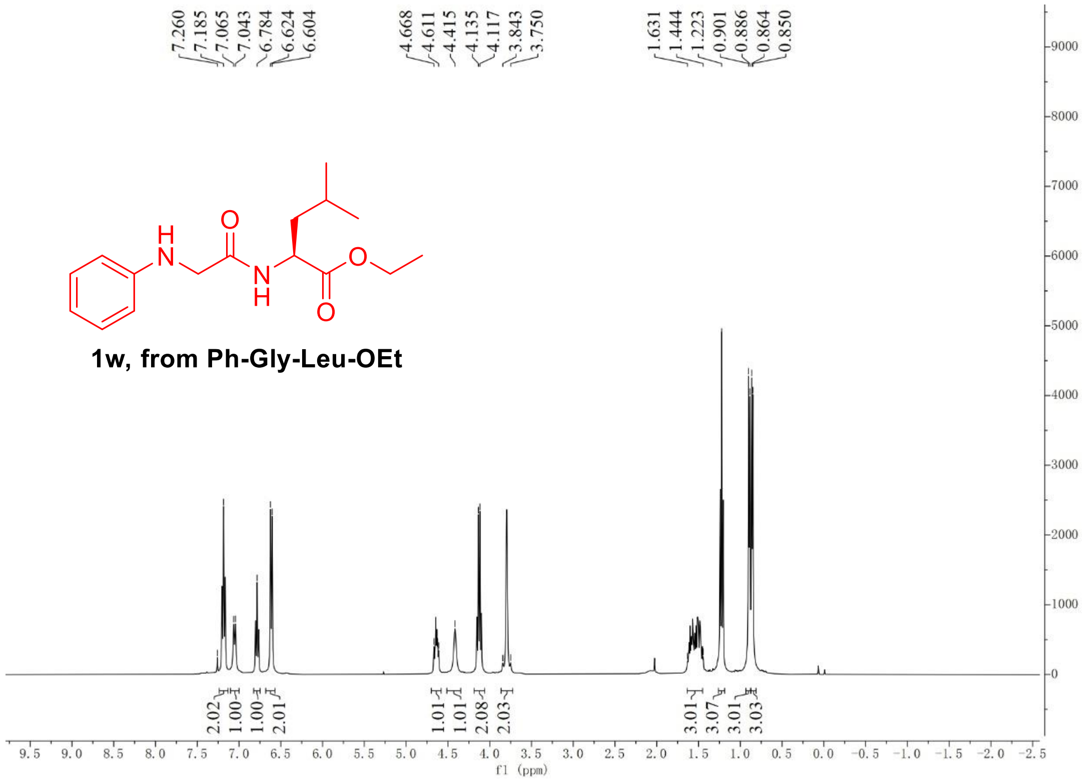
7.260
7.185
7.065
7.043
6.784
6.624
6.604
4.668
4.611
4.415
4.135
4.117
3.843
3.750
1.631
1.444
1.223
0.901
0.886
0.864
0.850
1w, from Ph-Gly-Leu-OEt
2.02
1.00
1.00
2.01
1.01
1.01
2.08
2.03
3.01
3.07
3.01
3.03
f1 (ppm)

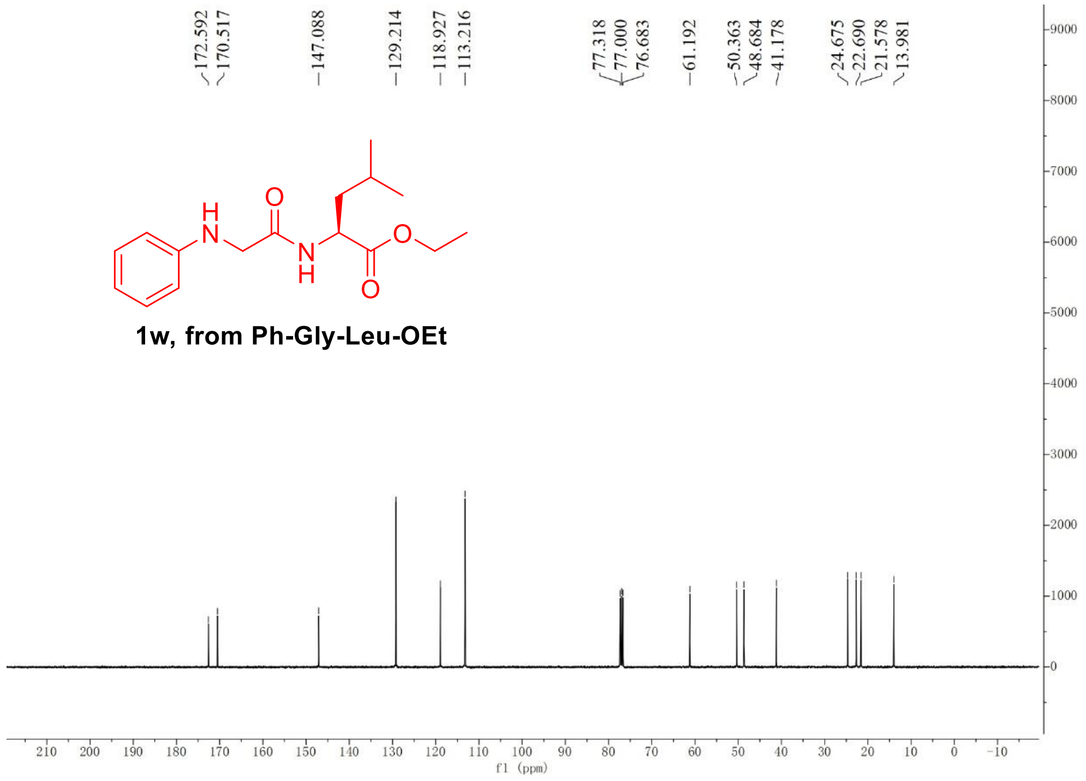
172.592
170.517
147.088
129.214
118.927
113.216
77.318
77.000
76.683
61.192
50.363
48.684
41.178
24.675
22.690
21.578
13.981
1w, from Ph-Gly-Leu-OEt
f1 (ppm)

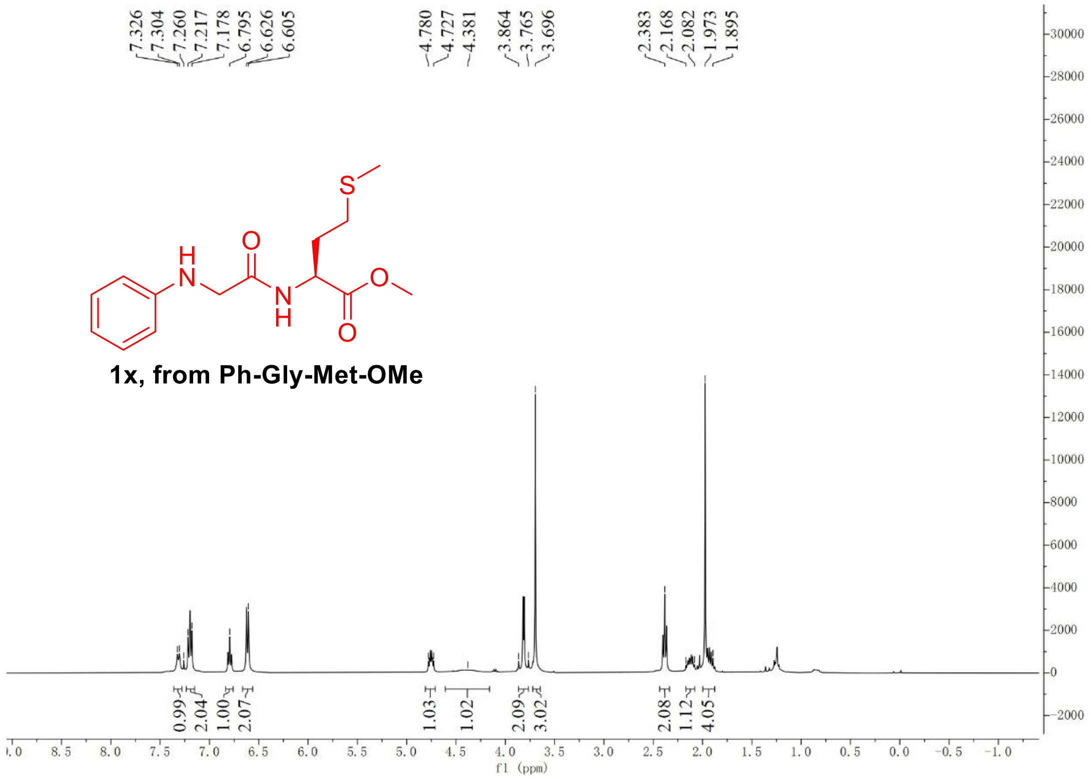
7.326
7.304
7.260
7.217
7.178
6.795
6.626
6.605
4.780
4.727
4.381
3.864
3.765
3.696
2.383
2.168
2.082
1.973
1.895
1x, from Ph-Gly-Met-OMe
0.99
2.04
1.00
2.07
1.03
1.02
2.09
3.02
2.08
1.12
4.05
f1 (ppm)

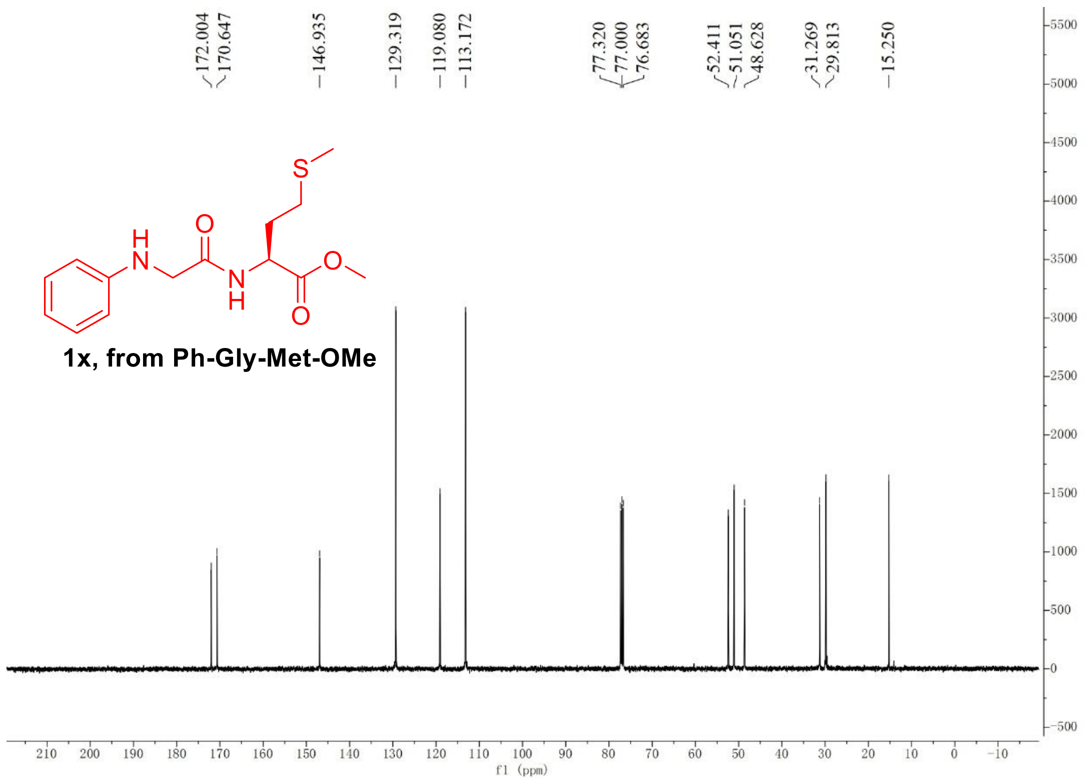
172.004
170.647
146.935
129.319
119.080
113.172
77.320
77.000
76.683
52.411
51.051
48.628
31.269
29.813
15.250
1x, from Ph-Gly-Met-OMe
f1 (ppm)

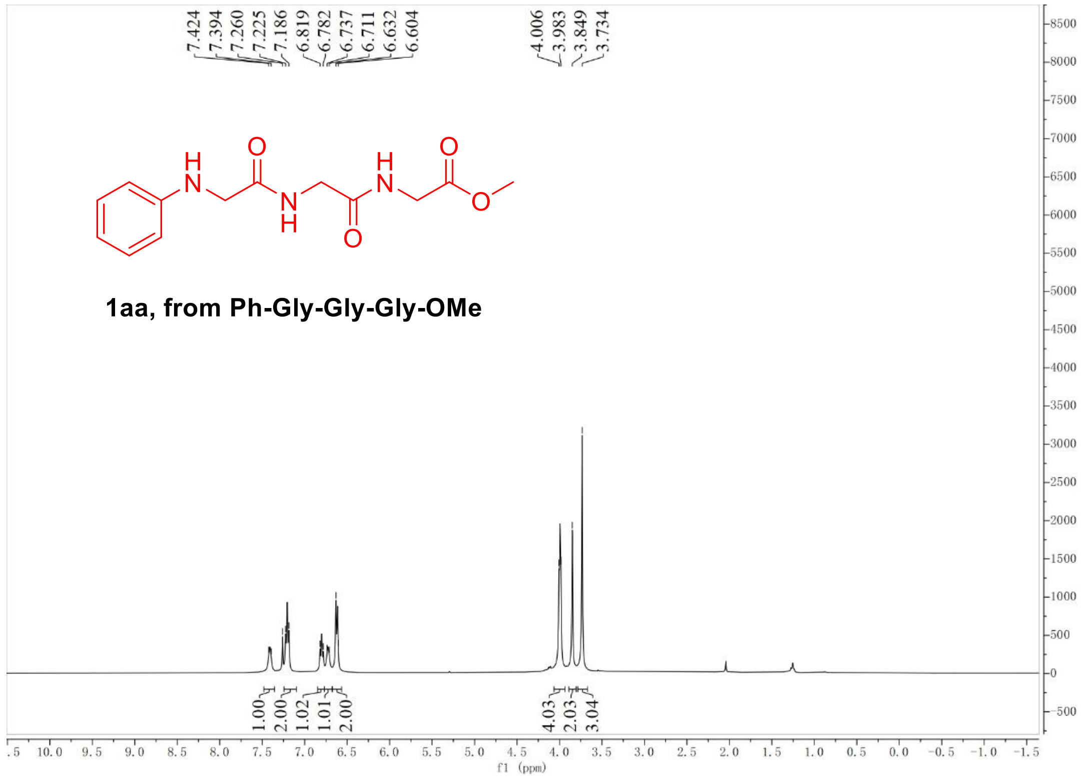
7.424
7.394
7.260
7.225
7.186
6.819
6.782
6.737
6.711
6.632
6.604
4.006
3.983
3.849
3.734
1aa, from Ph-Gly-Gly-Gly-OMe
1.00
2.00
1.02
1.01
2.00
4.03
2.03
3.04
f1 (ppm)

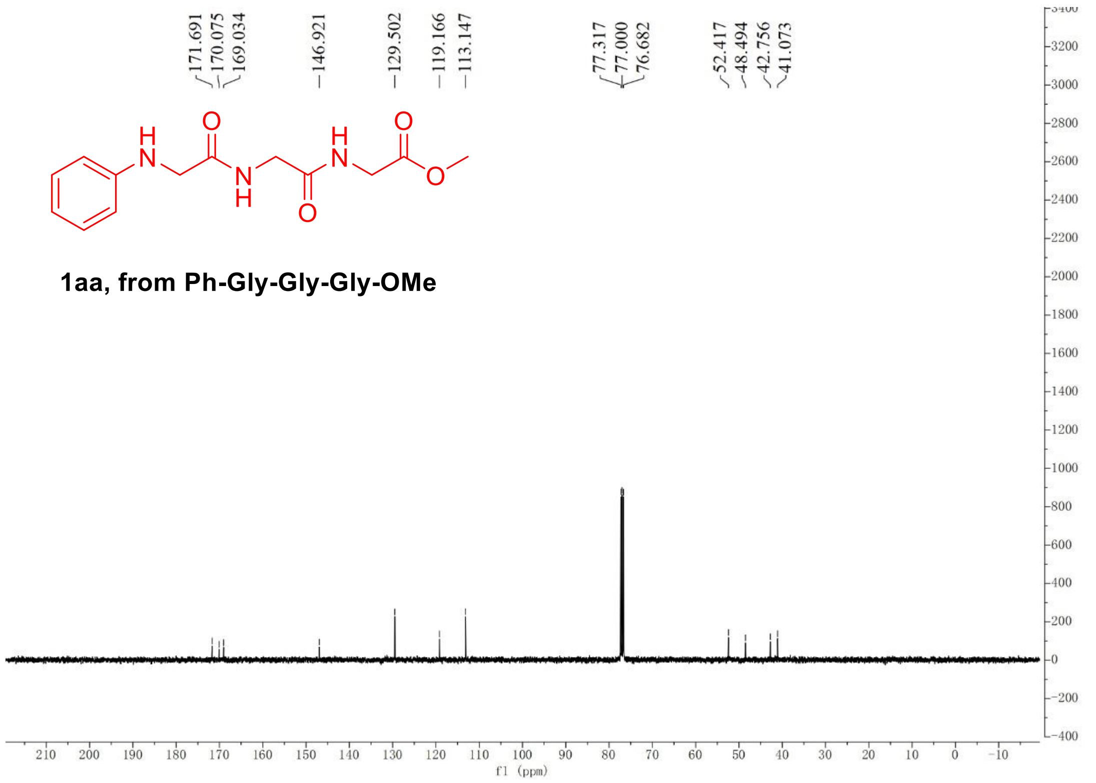

1aa, from Ph-Gly-Gly-Gly-OMe

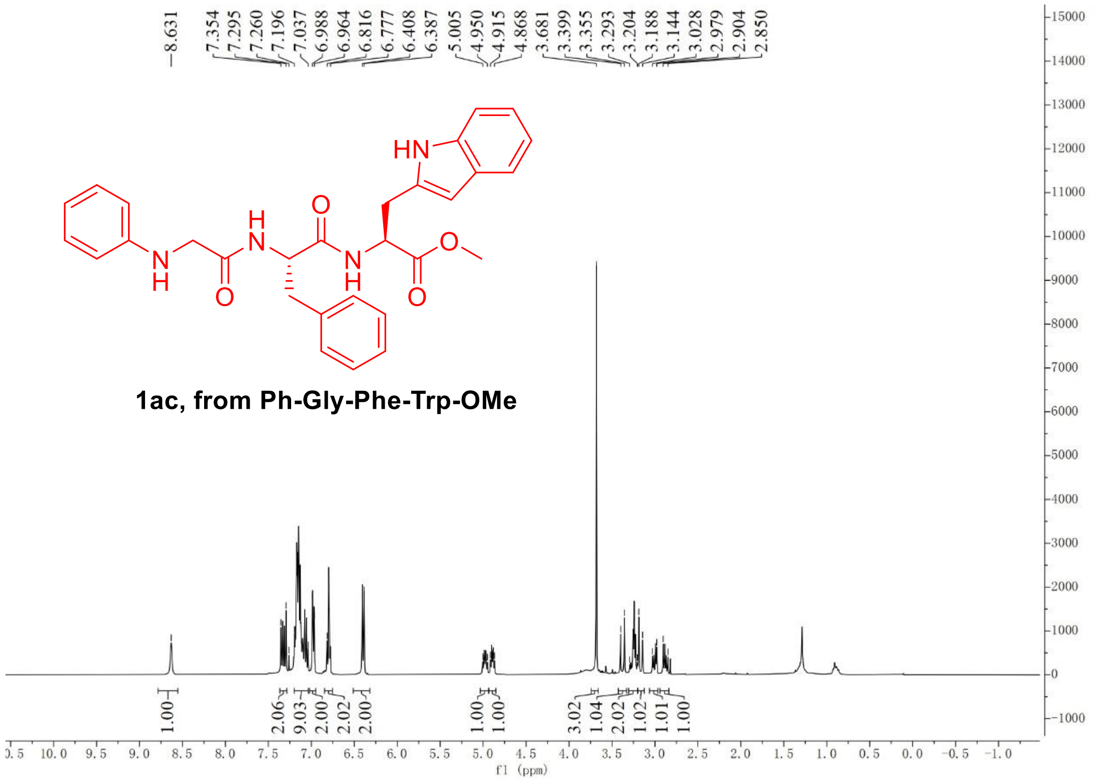

1ac, from Ph-Gly-Phe-Trp-OMe

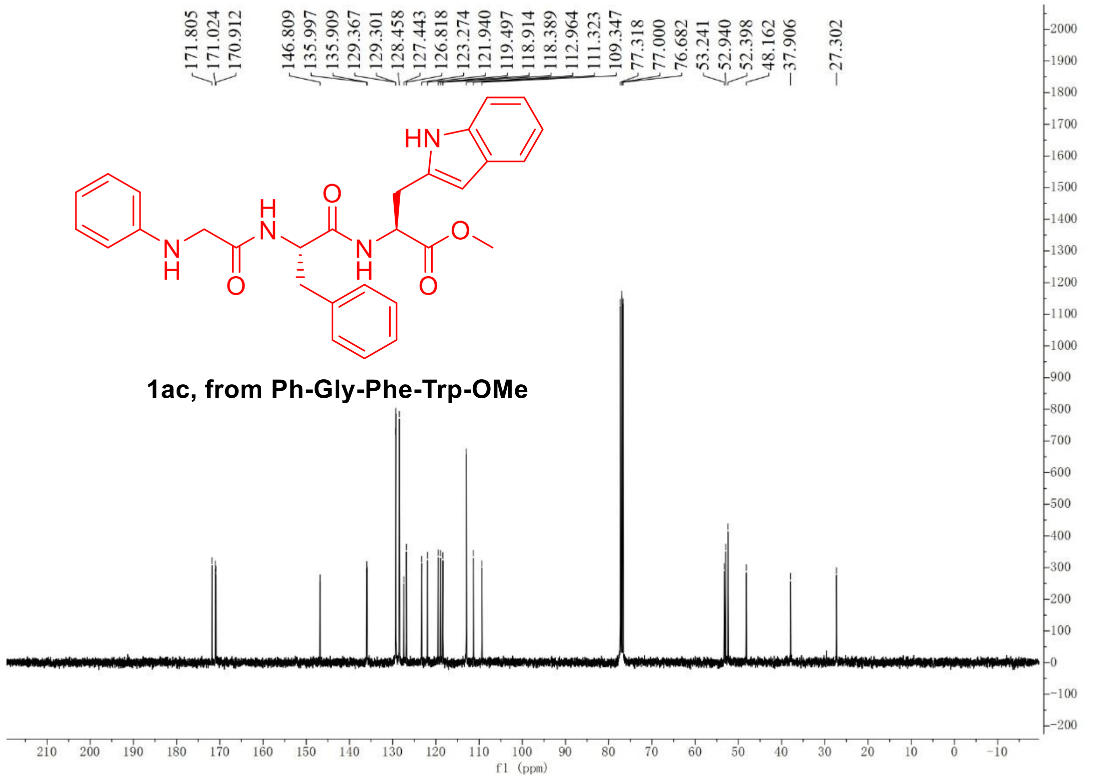
1ac, from Ph-Gly-Phe-Trp-OMe
f1 (ppm)

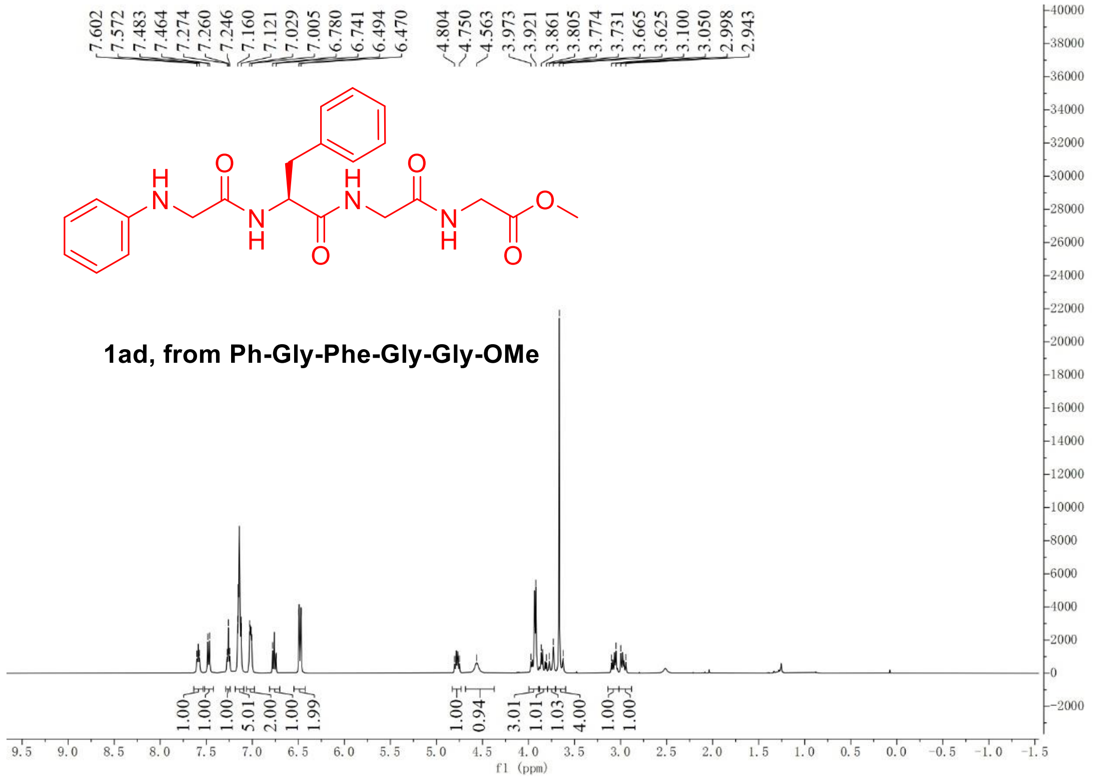
1ad, from Ph-Gly-Phe-Gly-Gly-OMe
f1 (ppm)

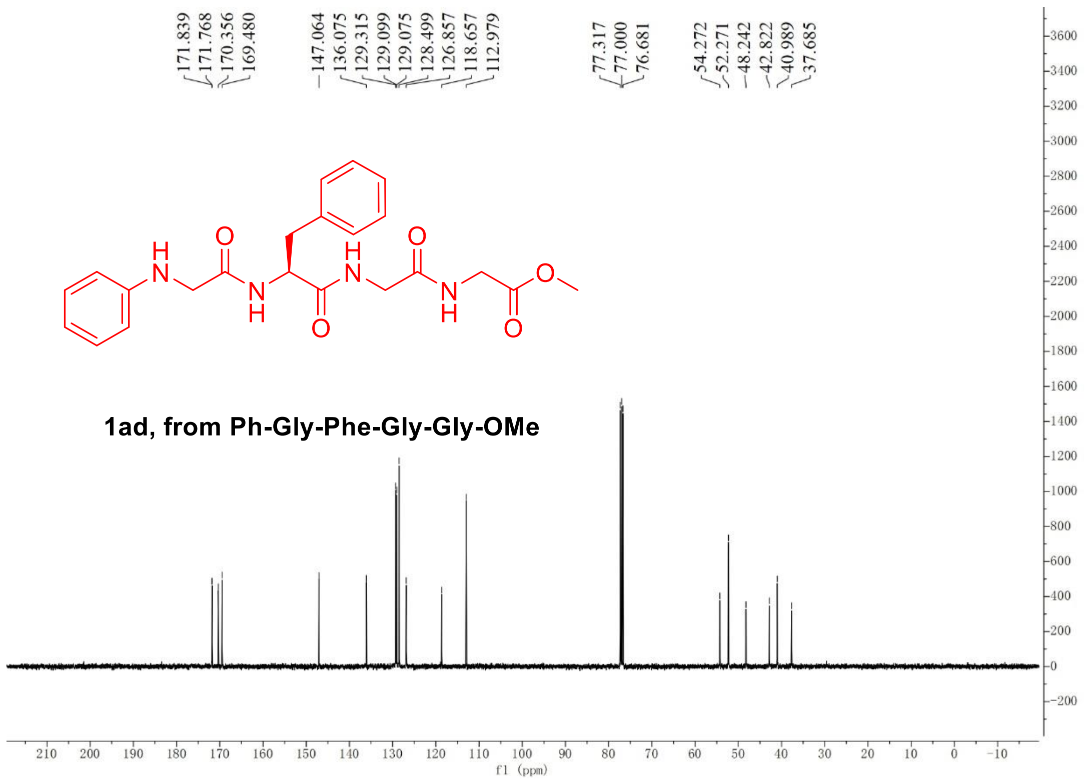

1ad, from Ph-Gly-Phe-Gly-Gly-OMe

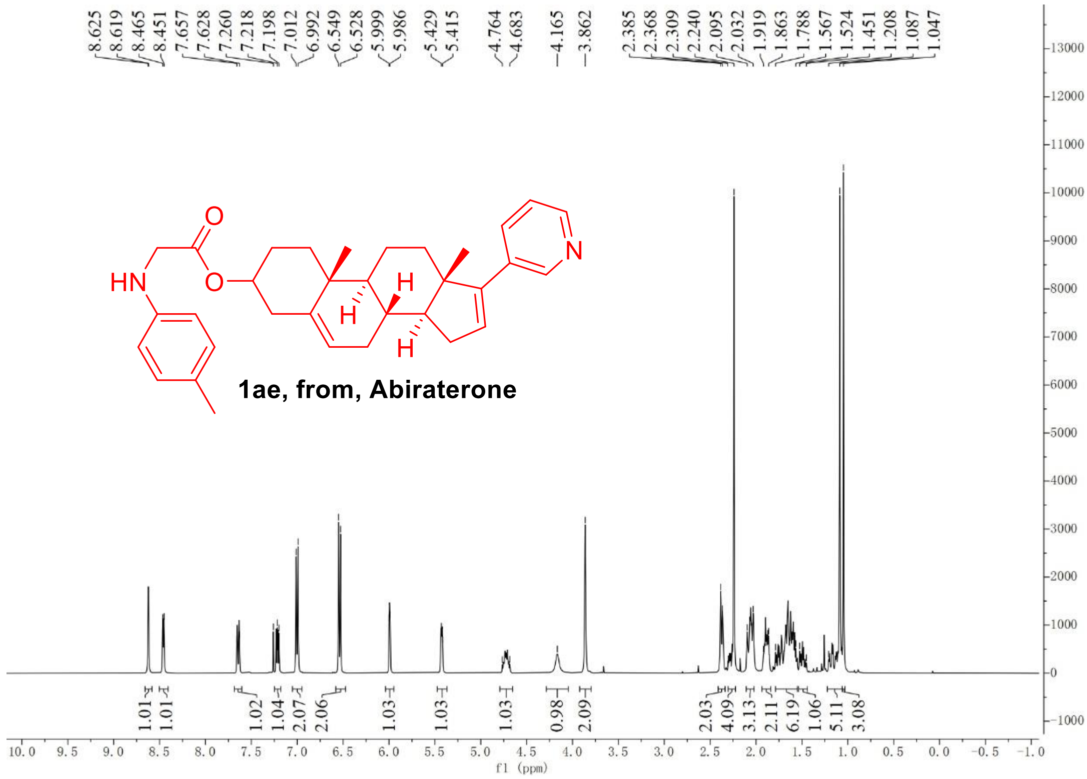

1ae, from, Abiraterone

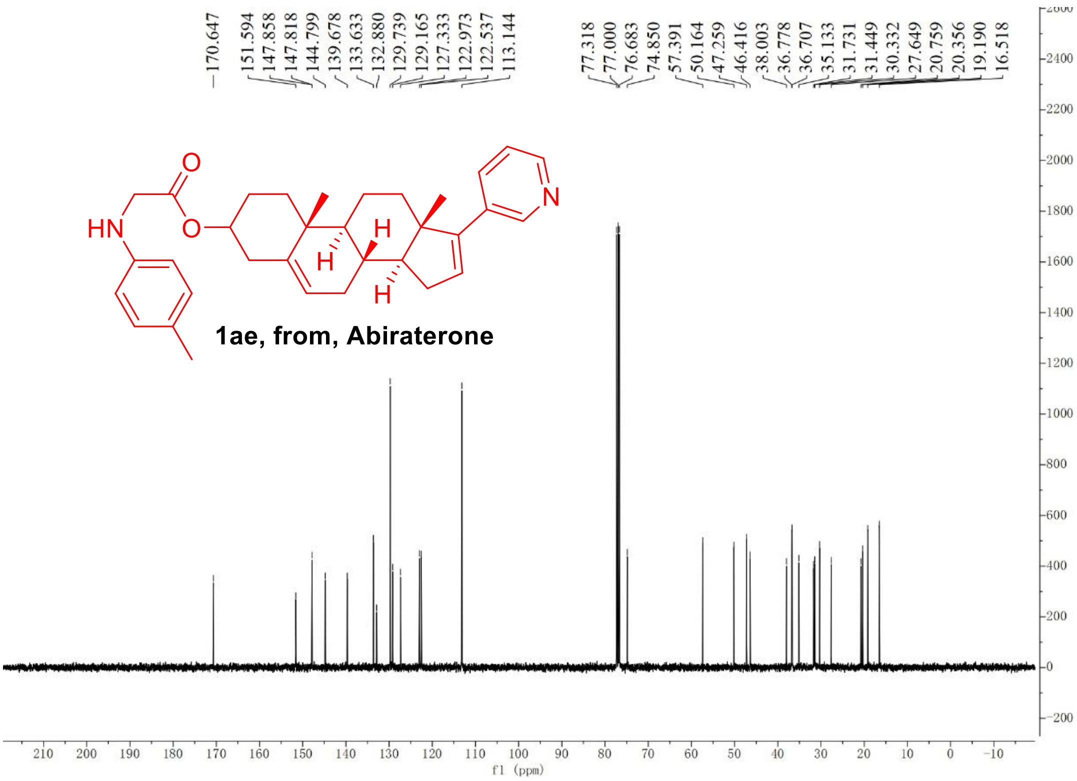
170.647
151.594
147.858
147.818
144.799
139.678
133.633
132.880
129.739
129.165
127.333
122.973
122.537
113.144
77.318
77.000
76.683
74.850
57.391
50.164
47.259
46.416
38.003
36.778
36.707
35.133
31.731
31.449
30.332
27.649
20.759
20.356
19.190
16.518
HN
O
O
H
H
H
N
1ae, from, Abiraterone
f1 (ppm)

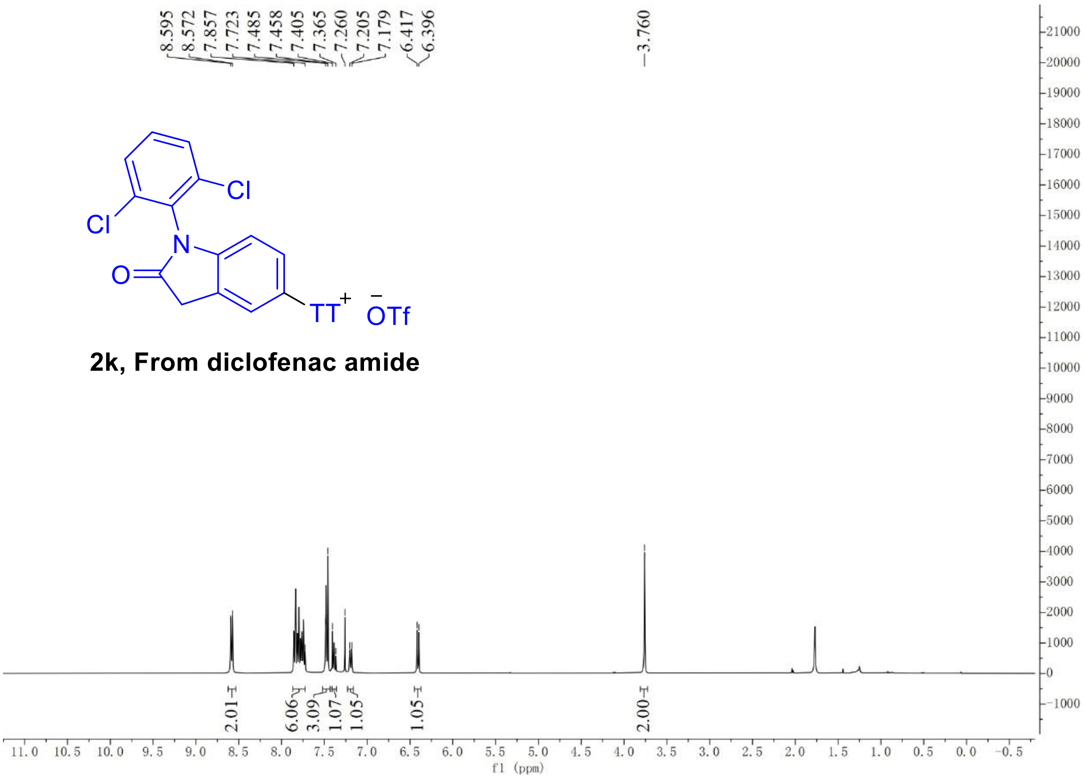
8.595
8.572
7.857
7.723
7.485
7.458
7.405
7.365
7.260
7.205
7.179
6.417
6.396
3.760
Cl
Cl
N
O
TT+
OTf
2k, From diclofenac amide
2.01
6.06
3.09
1.07
1.05
1.05
2.00
f1 (ppm)

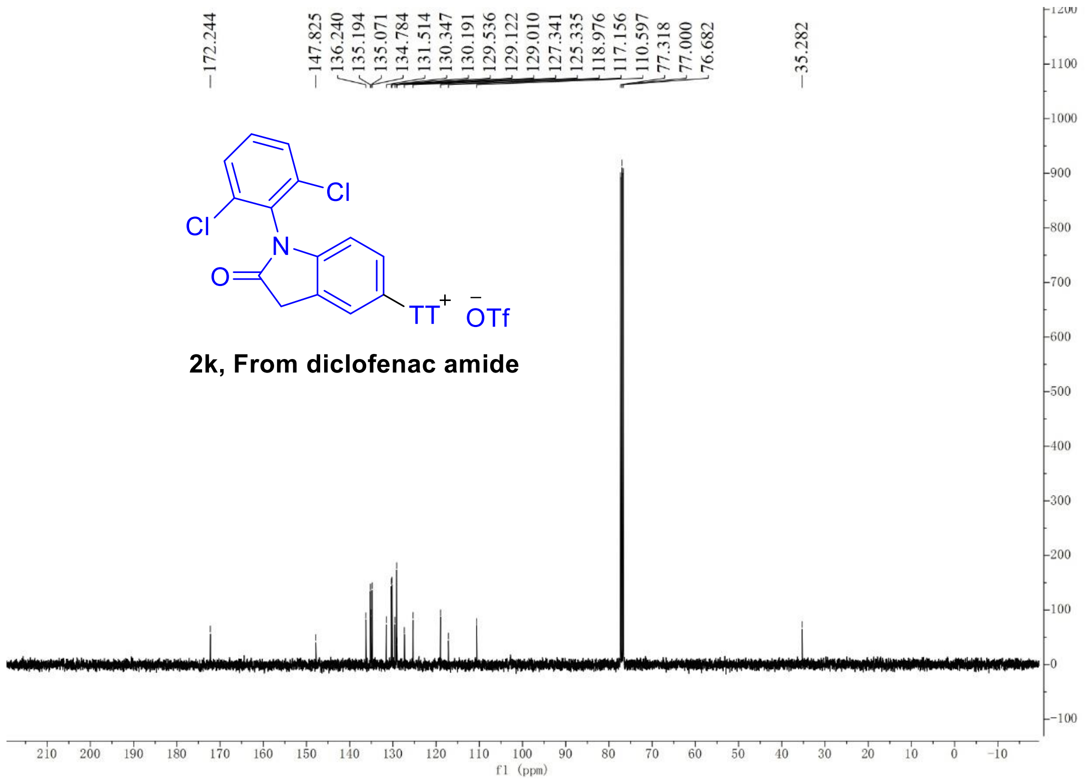
172.244
147.825
136.240
135.194
135.071
134.784
131.514
130.347
130.191
129.536
129.122
129.010
127.341
125.335
118.976
117.156
110.597
77.318
77.000
76.682
35.282
Cl
Cl
N
O
TT+
OTf
2k, From diclofenac amide
f1 (ppm)

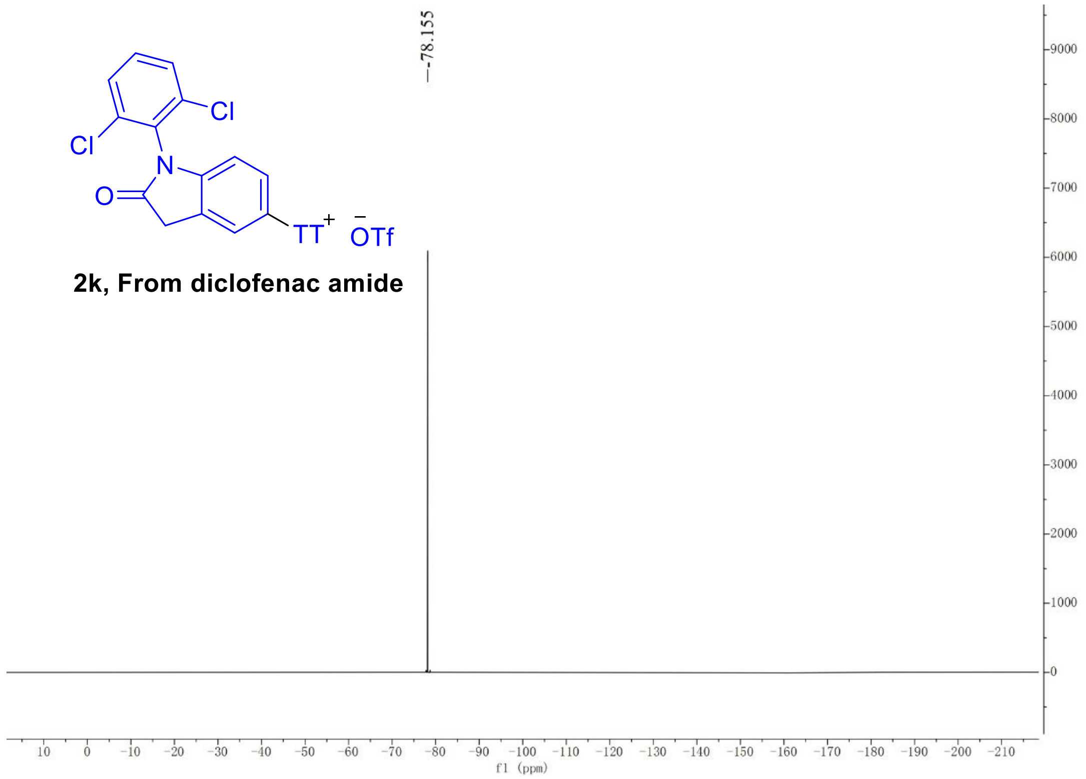
-78.155
Cl
Cl
N
O
TT+
OTf
2k, From diclofenac amide
f1 (ppm)

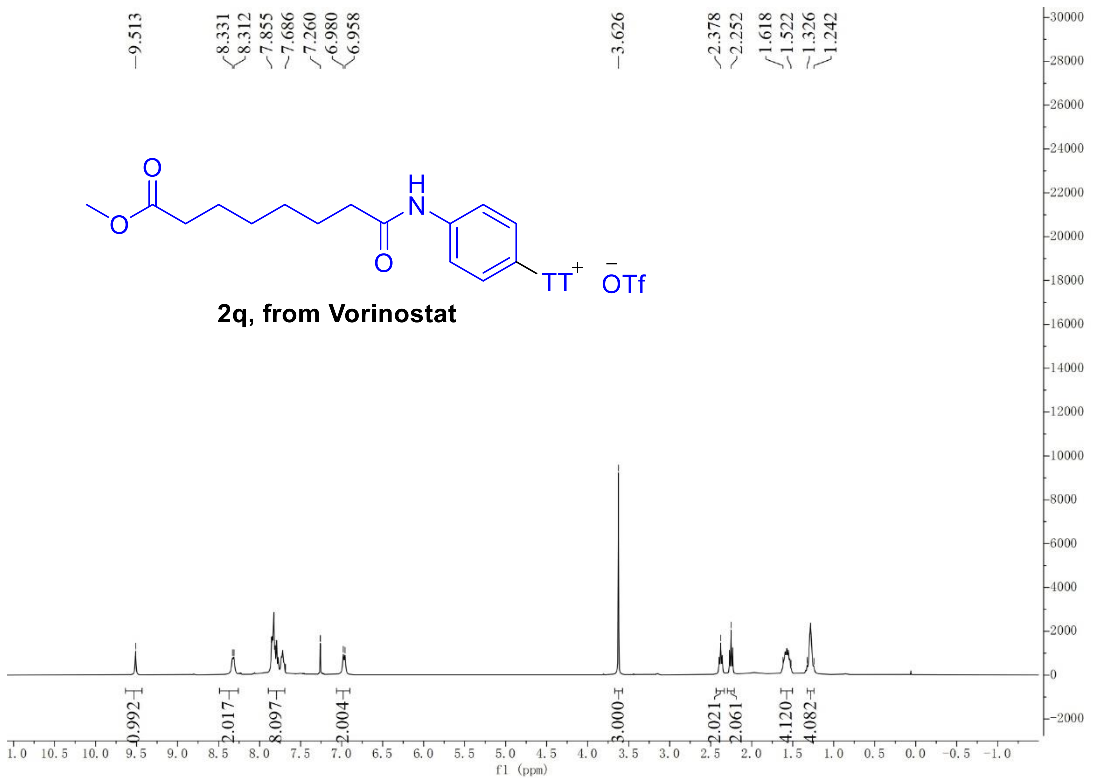

9.513
8.331
8.312
7.855
7.686
7.260
6.980
6.958
3.626
2.378
2.252
1.618
1.522
1.326
1.242
2q, from Vorinostat
TT+
OTf
0.992
2.017
8.097
2.004
3.000
2.021
2.061
4.120
4.082
f1 (ppm)

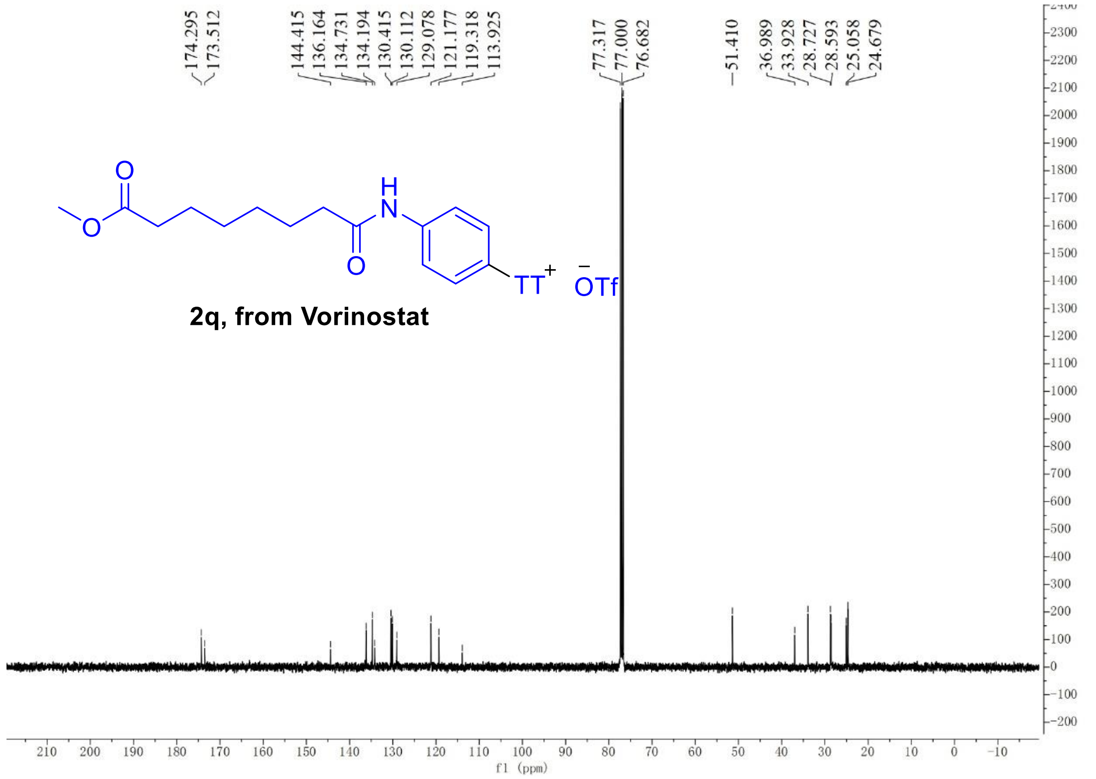

174.295
173.512
144.415
136.164
134.731
134.194
130.415
130.112
129.078
121.177
119.318
113.925
77.317
77.000
76.682
51.410
36.989
33.928
28.727
28.593
25.058
24.679
2q, from Vorinostat
TT+
OTf
f1 (ppm)

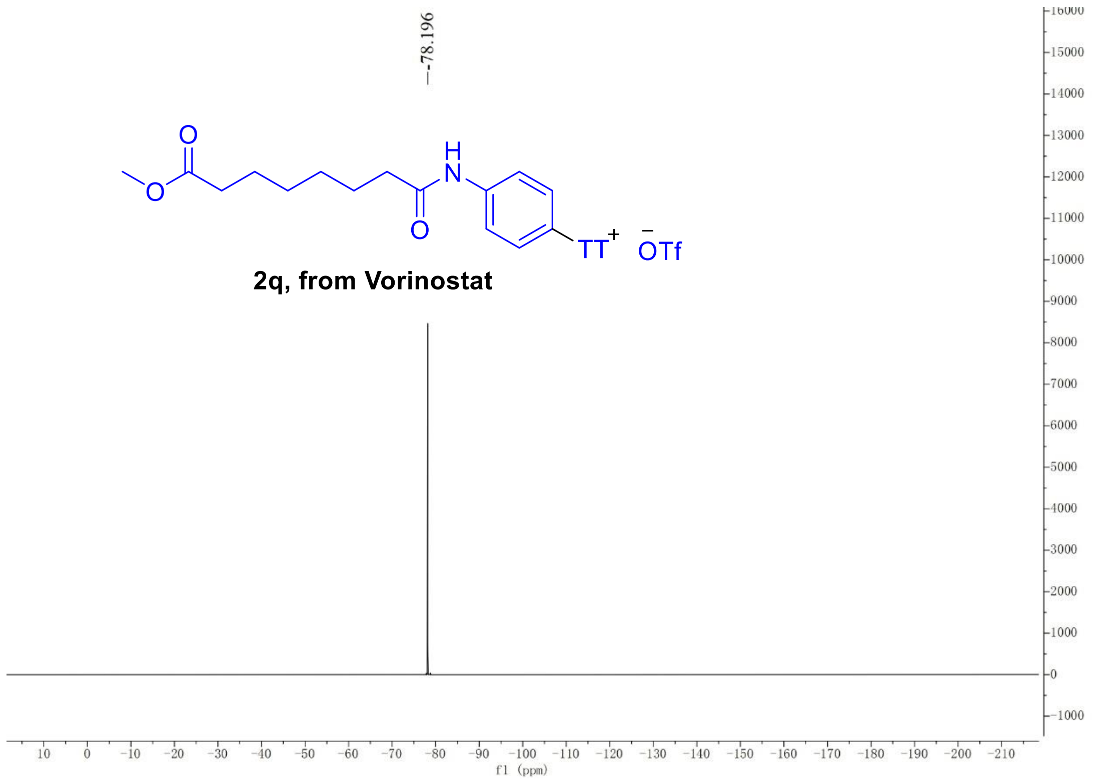

-78.196
2q, from Vorinostat
f1 (ppm)

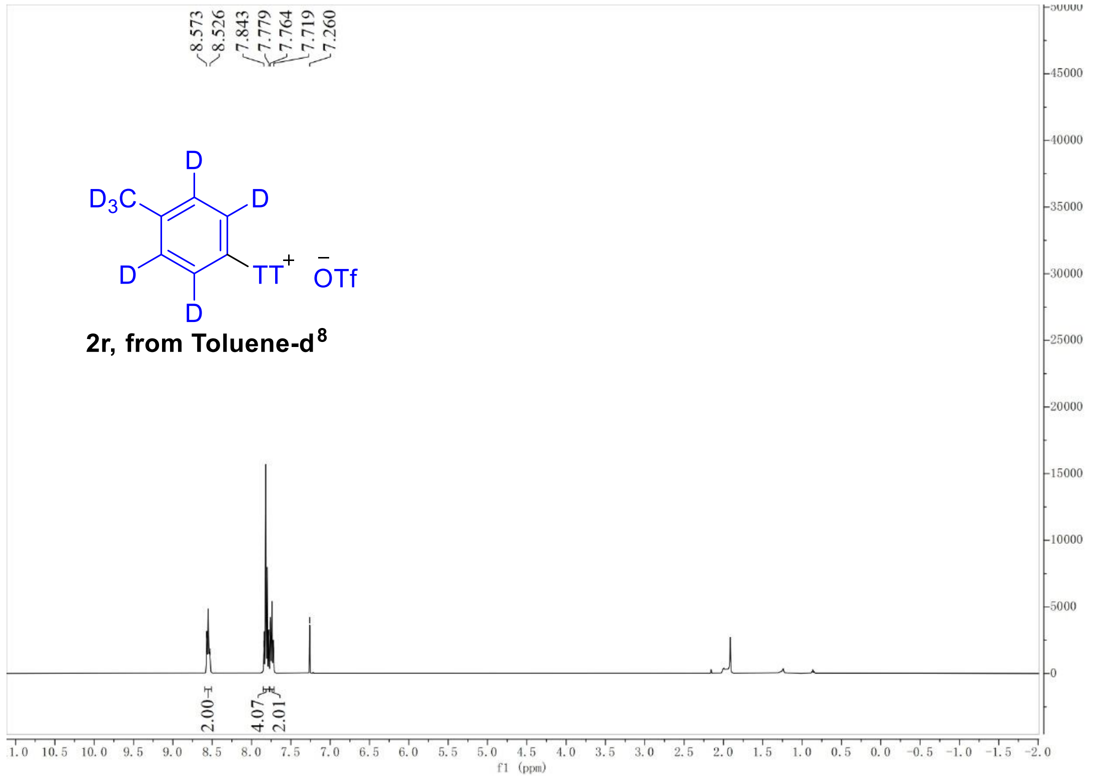

8.573
8.526
7.843
7.779
7.764
7.719
7.260
2r, from Toluene-d8
2.00
4.07
2.01
f1 (ppm)

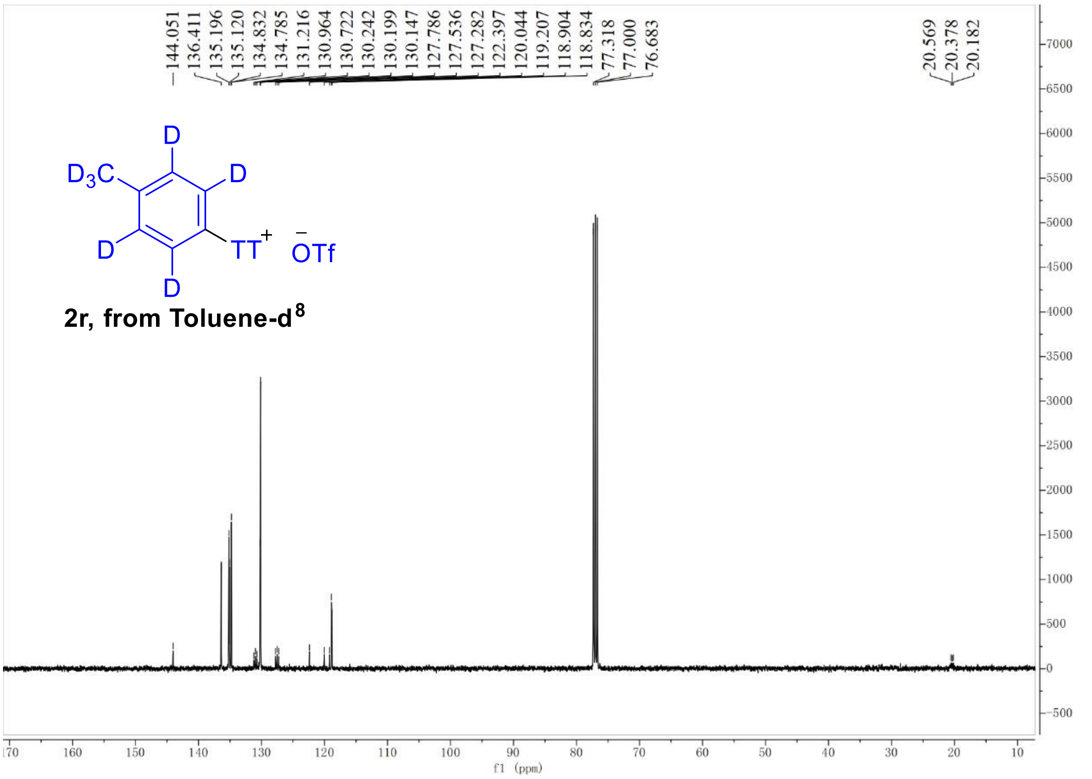
144.051
136.411
135.196
135.120
134.832
134.785
131.216
130.964
130.722
130.242
130.199
130.147
127.786
127.536
127.282
122.397
120.044
119.207
118.904
118.834
77.318
77.000
76.683
20.569
20.378
20.182
D3C
D
TT+
OTf
2r, from Toluene-d8
f1 (ppm)

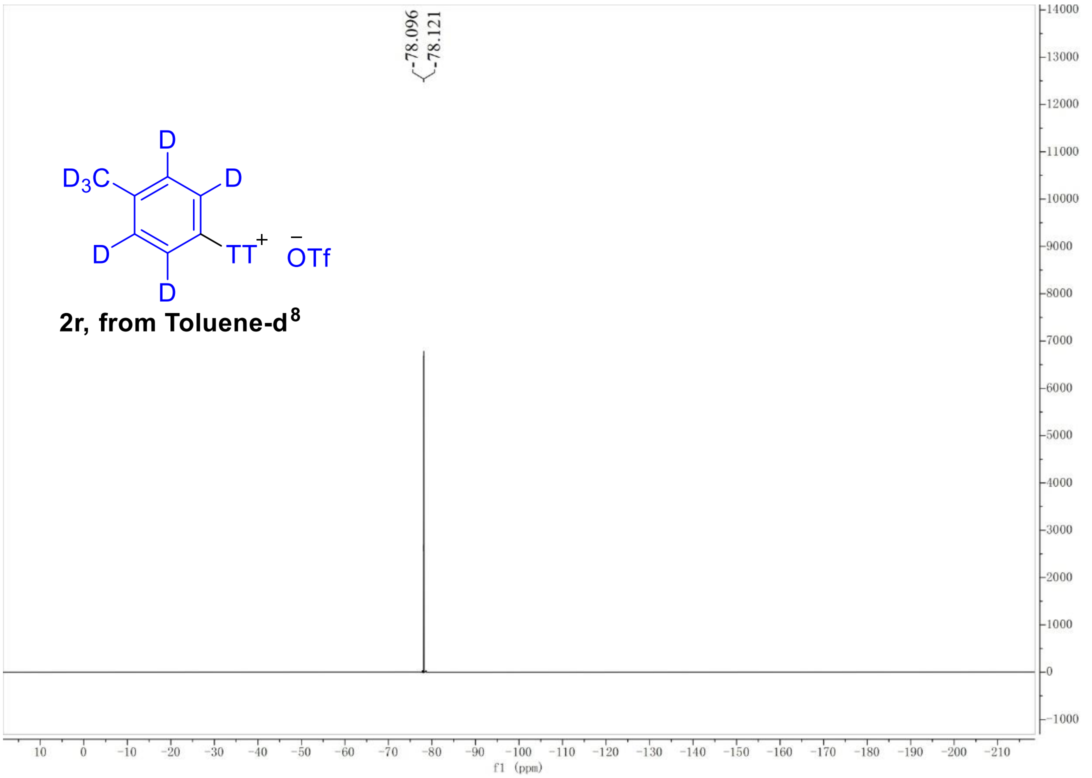
-78.096
-78.121
D3C
D
TT+
OTf
2r, from Toluene-d8
f1 (ppm)

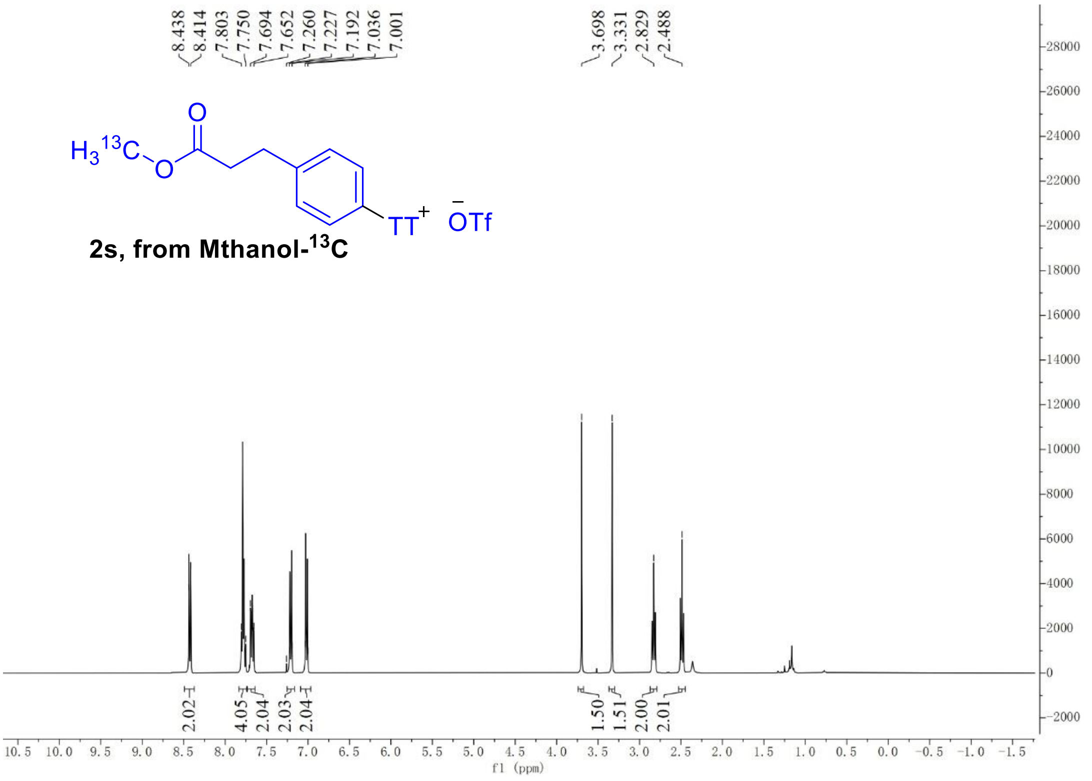
8.438
8.414
7.803
7.750
7.694
7.652
7.260
7.227
7.192
7.036
7.001
3.698
3.331
2.829
2.488
H3 13C O O TT+ OTf
2s, from Mthanol-13C
2.02
4.05
2.04
2.03
2.04
1.50
1.51
2.00
2.01
f1 (ppm)

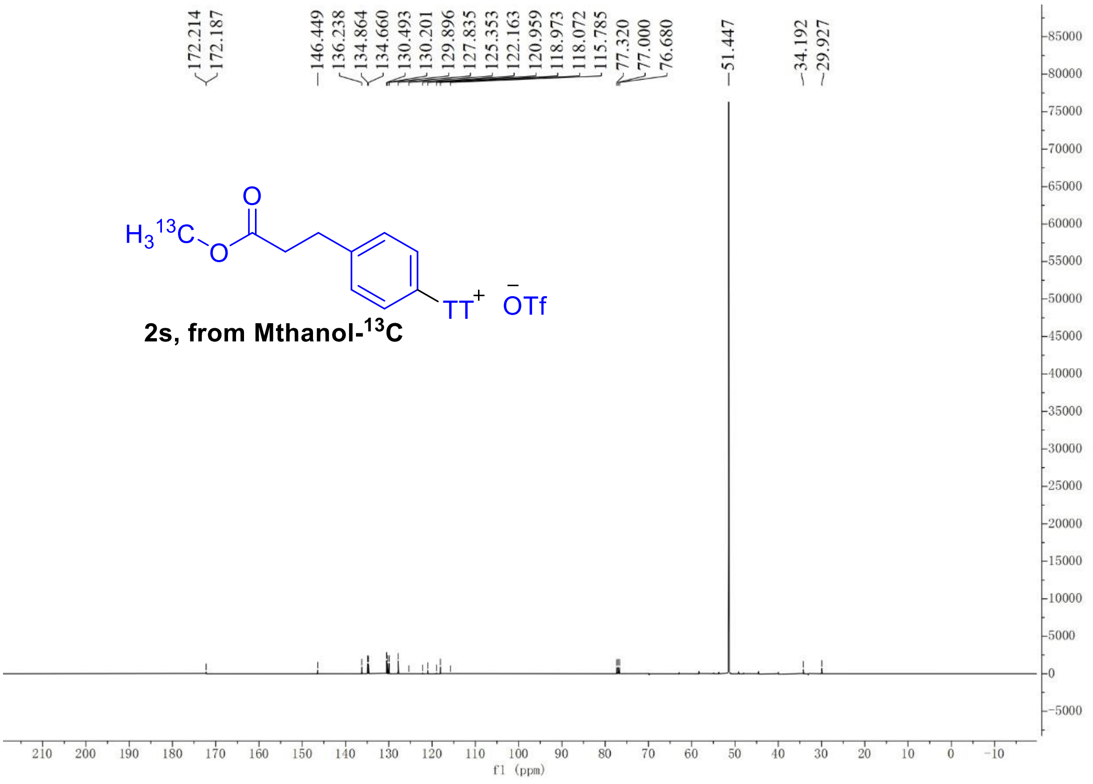
172.214
172.187
146.449
136.238
134.864
134.660
130.493
130.201
129.896
127.835
125.353
122.163
120.959
118.973
118.072
115.785
77.320
77.000
76.680
51.447
34.192
29.927
H3 13C O O TT+ OTf
2s, from Mthanol-13C
f1 (ppm)

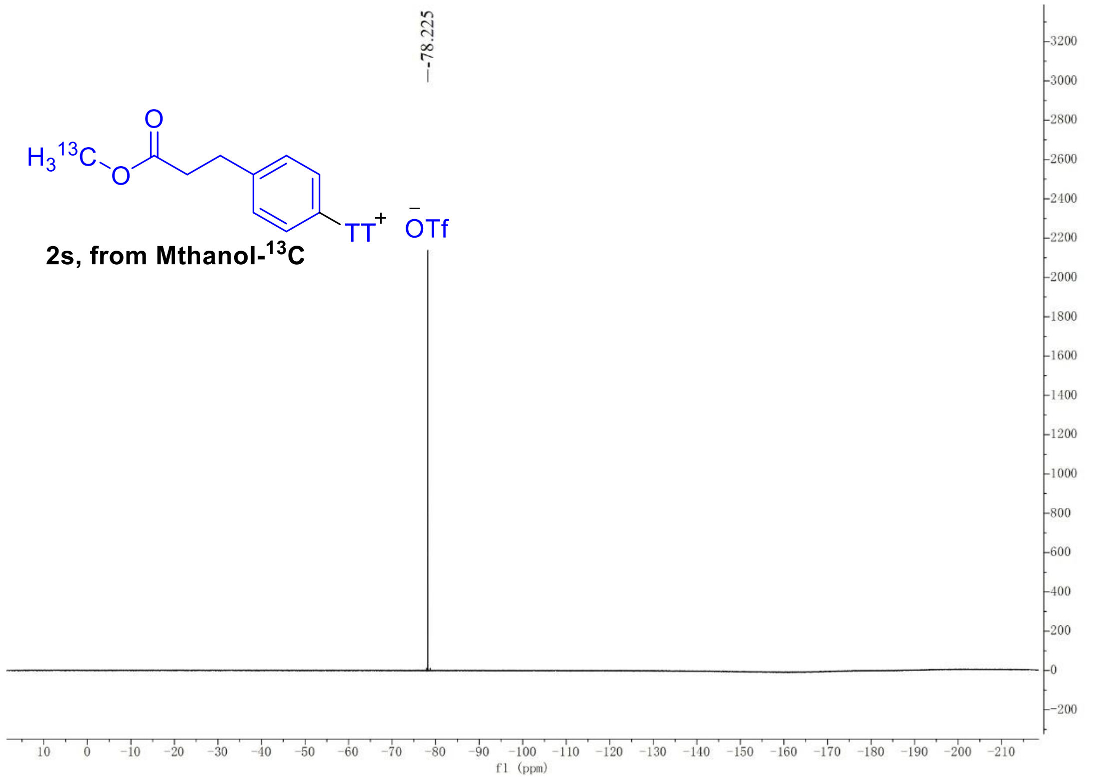

-78.225
2s, from Mthanol-13C
f1 (ppm)

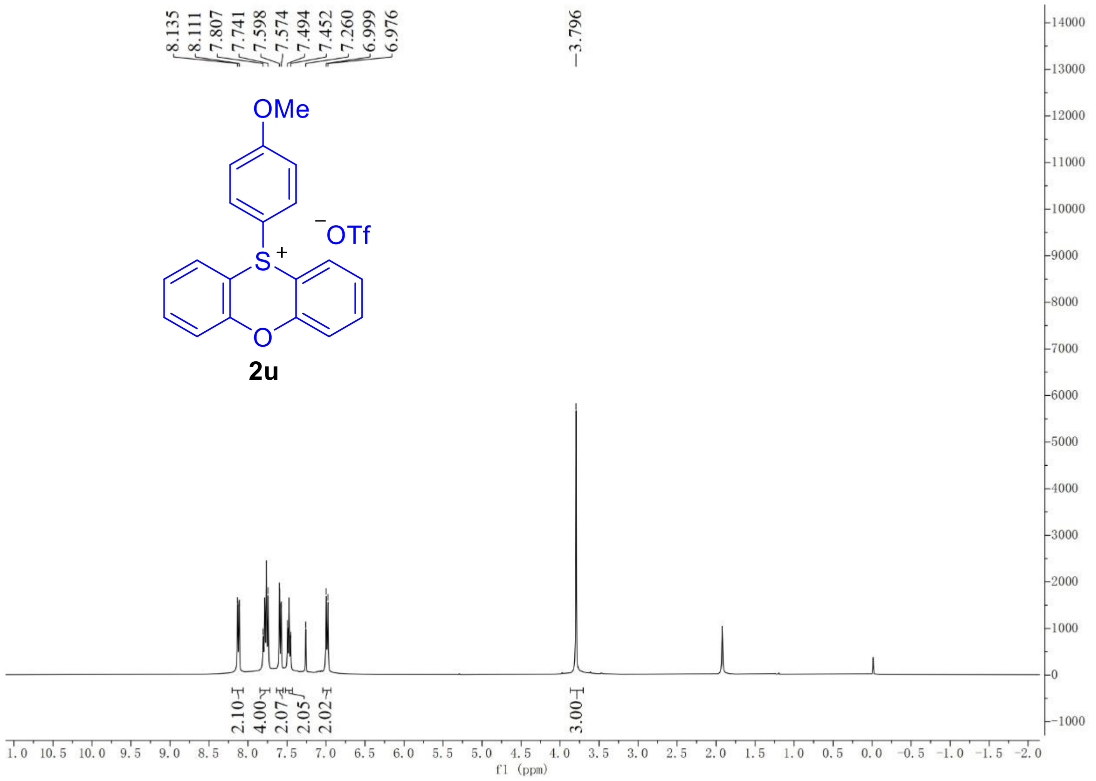

8.135
8.111
7.807
7.741
7.598
7.574
7.494
7.452
7.260
6.999
6.976
3.796
2u
2.10
4.00
2.07
2.05
2.02
3.00
f1 (ppm)

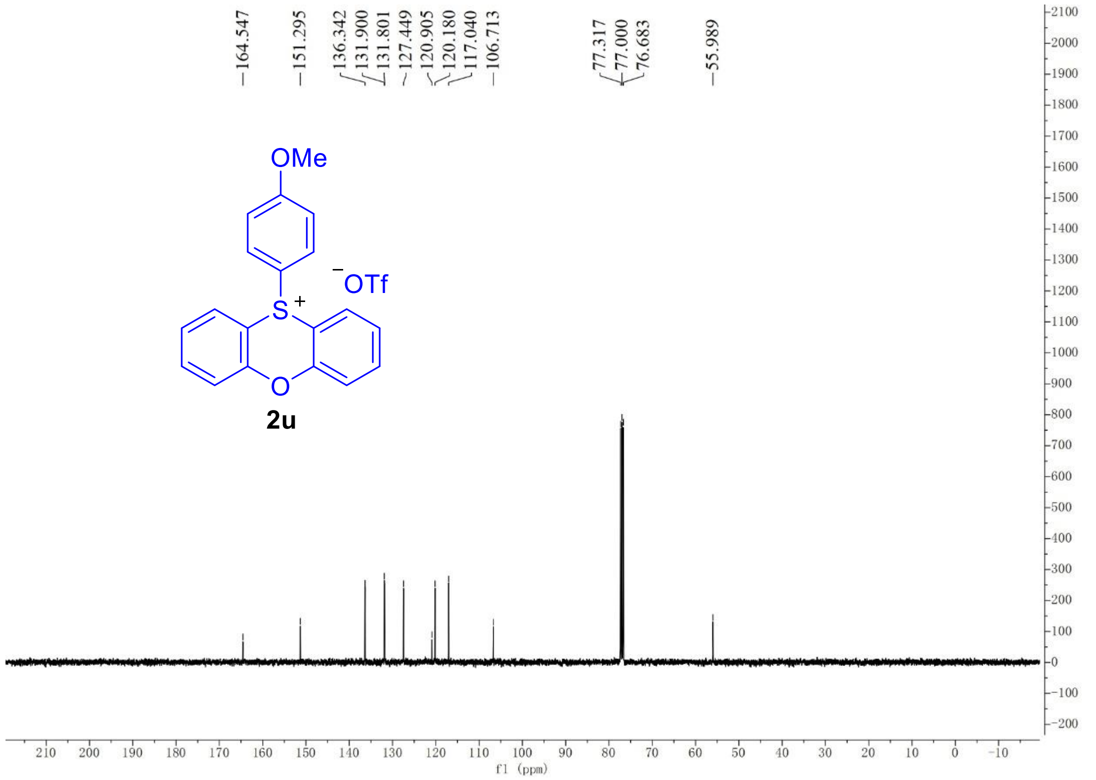
164.547
151.295
136.342
131.900
131.801
127.449
120.905
120.180
117.040
106.713
77.317
77.000
76.683
55.989
OMe
OTf
S
O
2u
f1 (ppm)

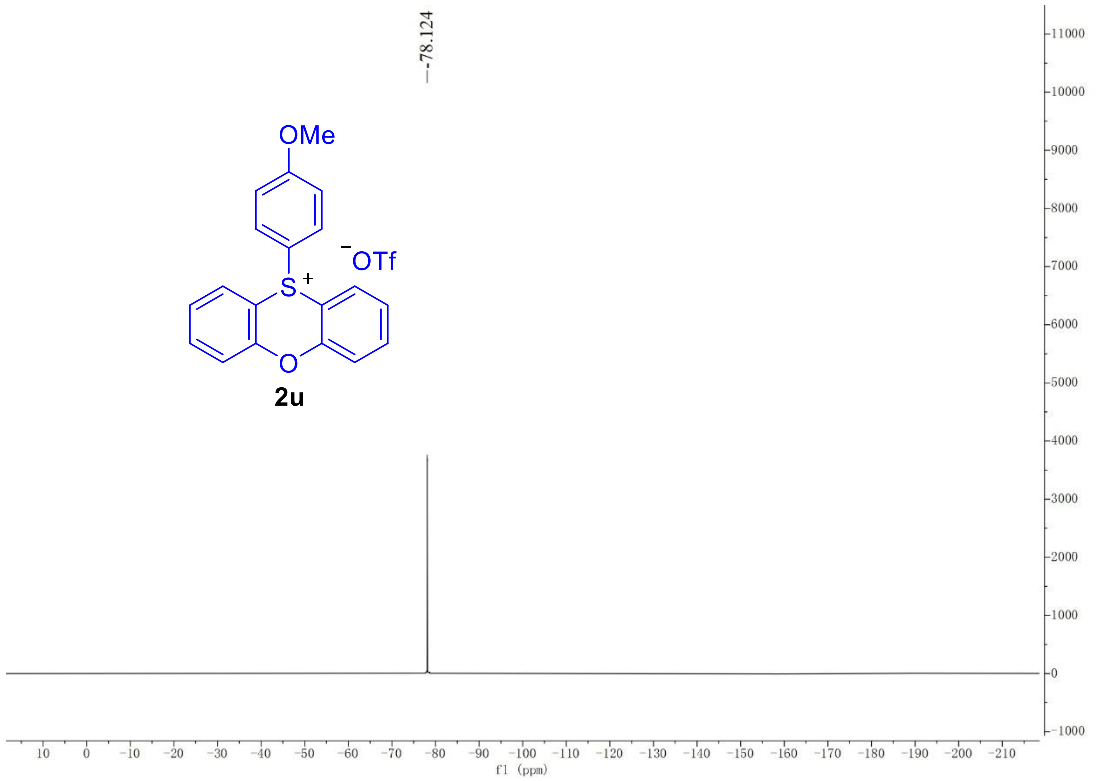
-78.124
OMe
OTf
S
O
2u
f1 (ppm)

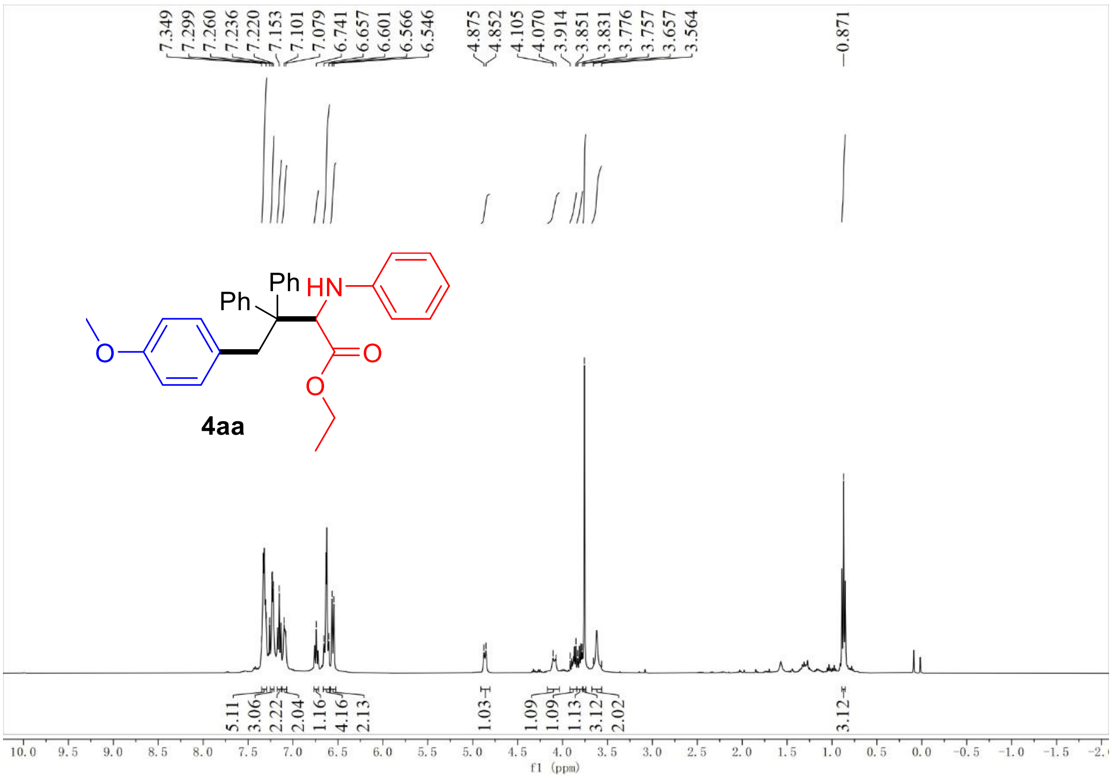
7.349
7.299
7.260
7.236
7.220
7.153
7.101
7.079
6.741
6.657
6.601
6.566
6.546
4.875
4.852
4.105
4.070
3.914
3.851
3.831
3.776
3.757
3.657
3.564
0.871
Ph
Ph
HN
O
O
O
4aa
5.11
3.06
2.22
2.04
1.16
4.16
2.13
1.03
1.09
1.09
1.13
3.12
2.02
3.12
f1 (ppm)

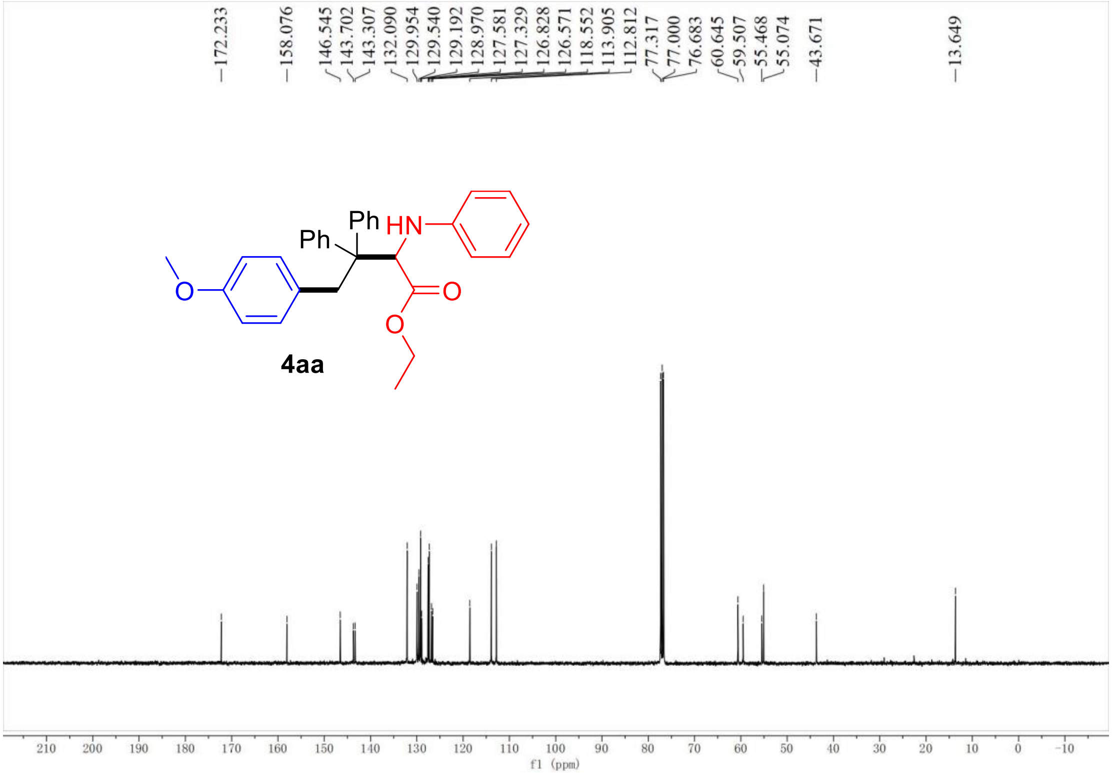
172.233
158.076
146.545
143.702
143.307
132.090
129.954
129.540
129.192
128.970
127.581
127.329
126.828
126.571
118.552
113.905
112.812
77.317
77.000
76.683
60.645
59.507
55.468
55.074
43.671
13.649
Ph
Ph
HN
O
O
O
4aa
f1 (ppm)

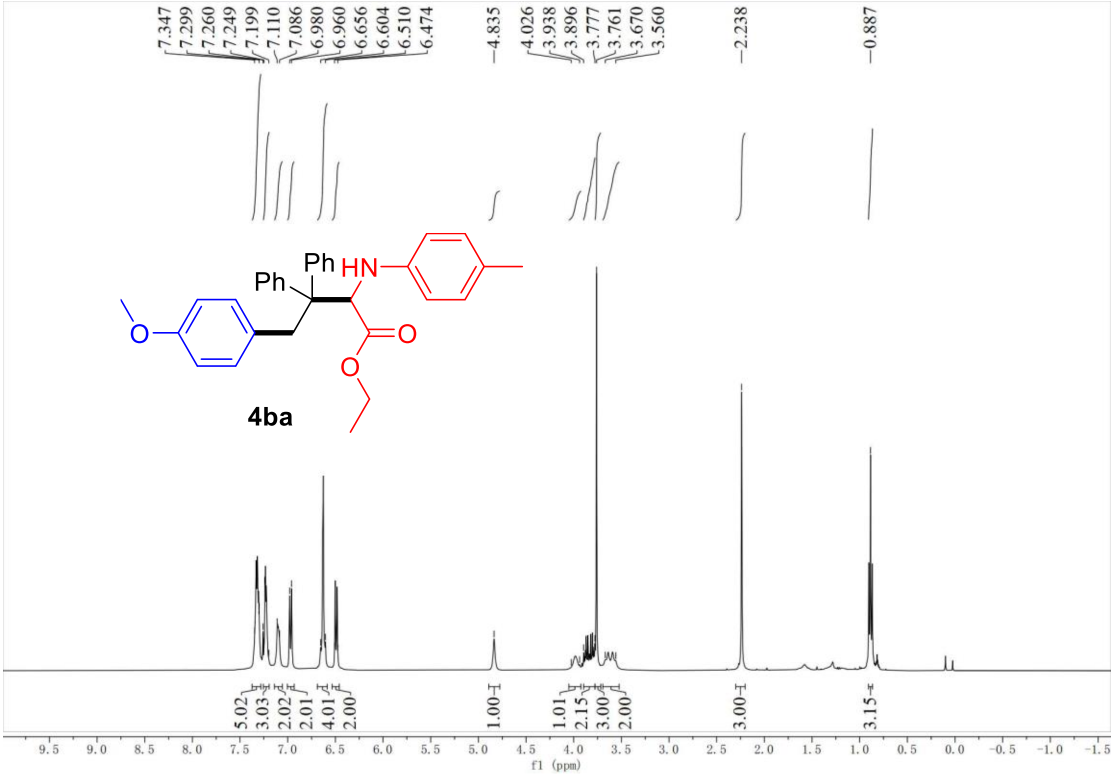

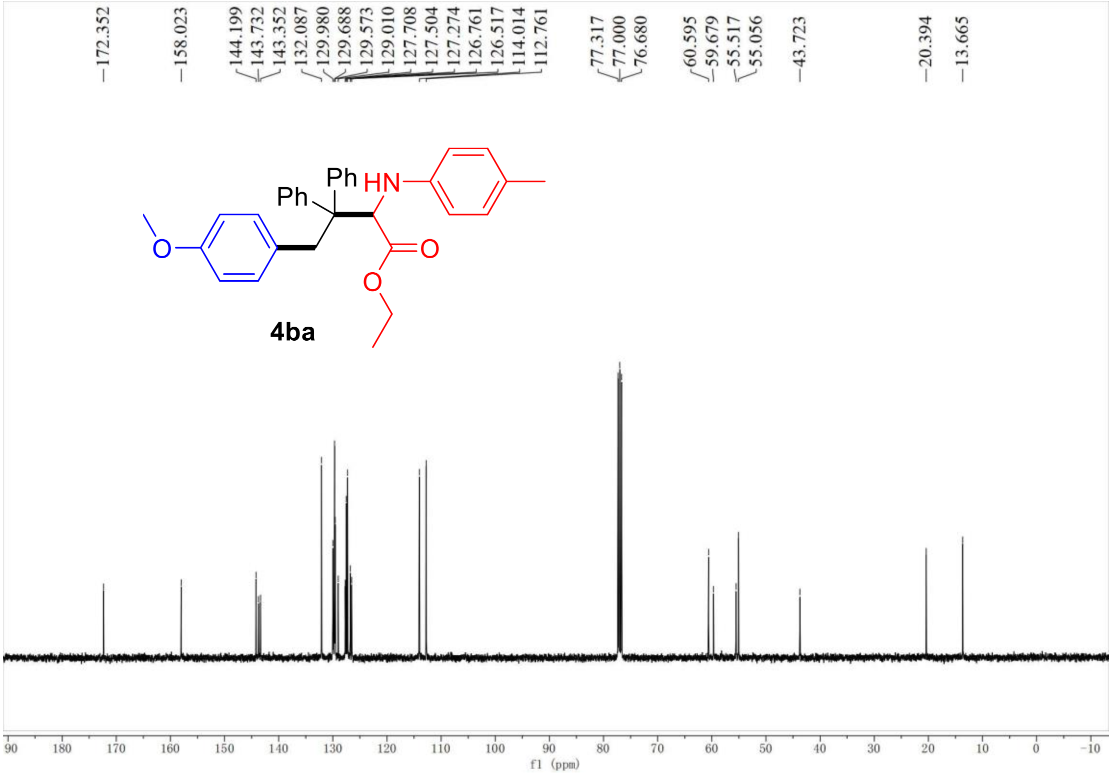

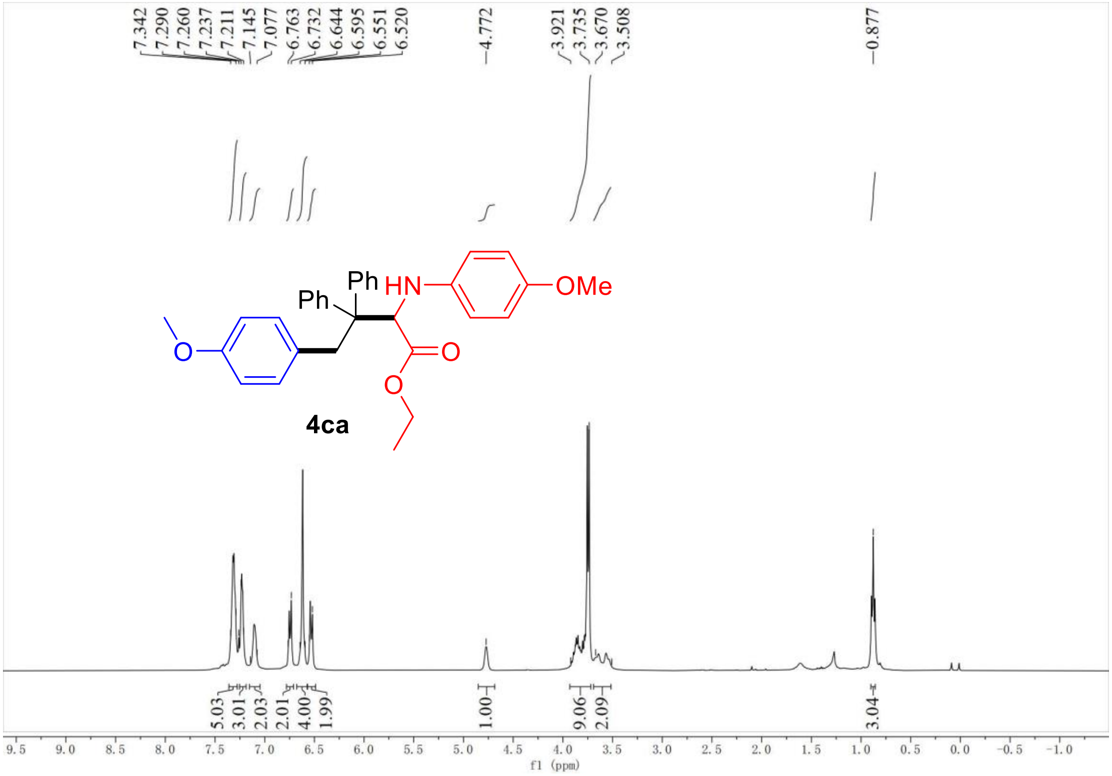
7.342
7.290
7.260
7.237
7.211
7.145
7.077
6.763
6.732
6.644
6.595
6.551
6.520
4.772
3.921
3.735
3.670
3.508
0.877
Ph
Ph
HN
OMe
O
O
O
4ca
5.03
3.01
2.03
2.01
4.00
1.99
1.00
9.06
2.09
3.04
f1 (ppm)

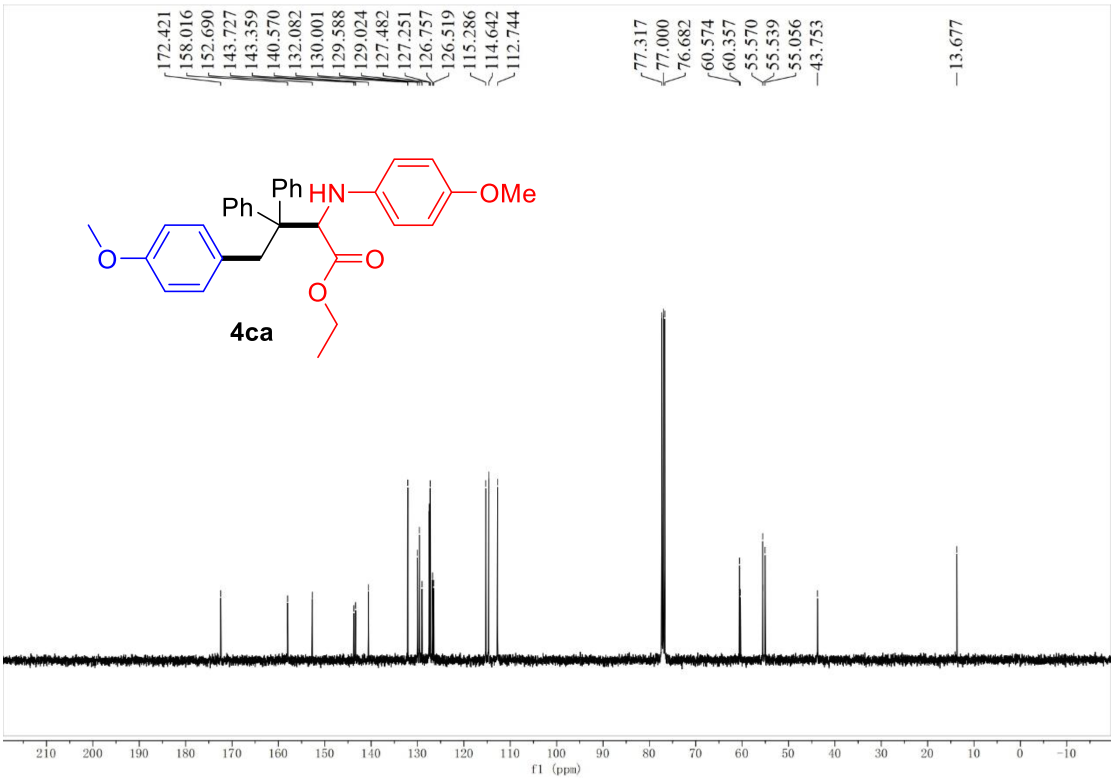
172.421
158.016
152.690
143.727
143.359
140.570
132.082
130.001
129.588
129.024
127.482
127.251
126.757
126.519
115.286
114.642
112.744
77.317
77.000
76.682
60.574
60.357
55.570
55.539
55.056
43.753
13.677
Ph
Ph
HN
OMe
O
O
O
4ca
f1 (ppm)

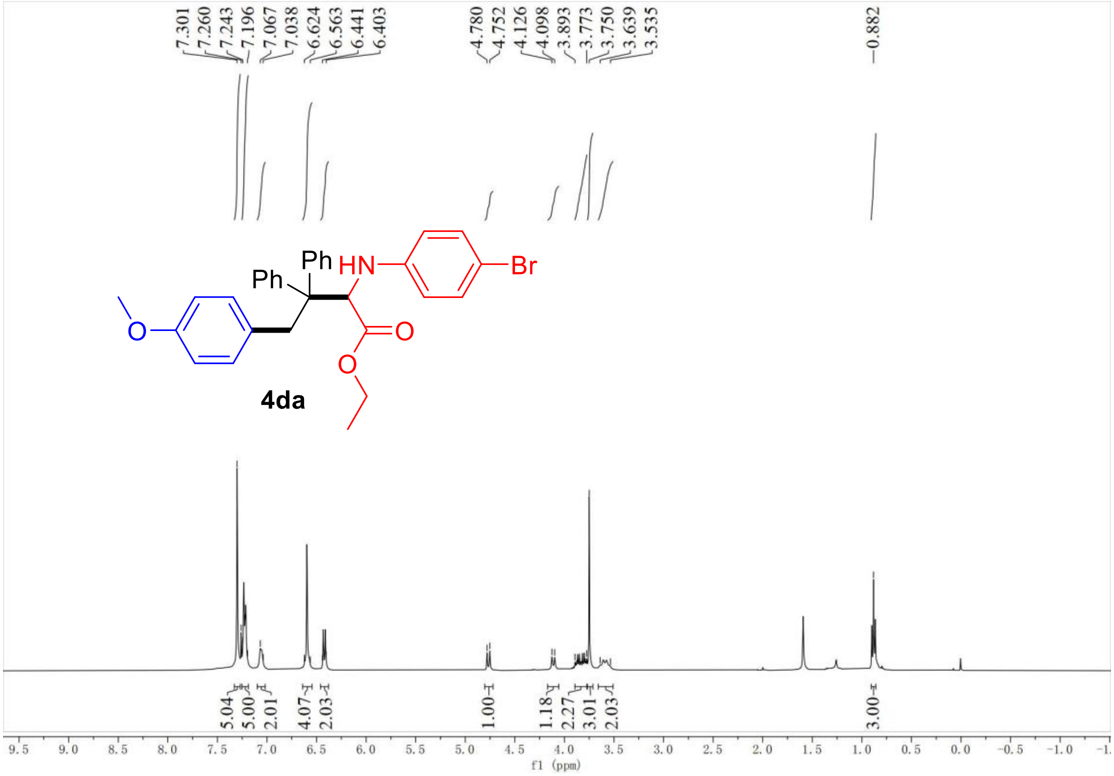

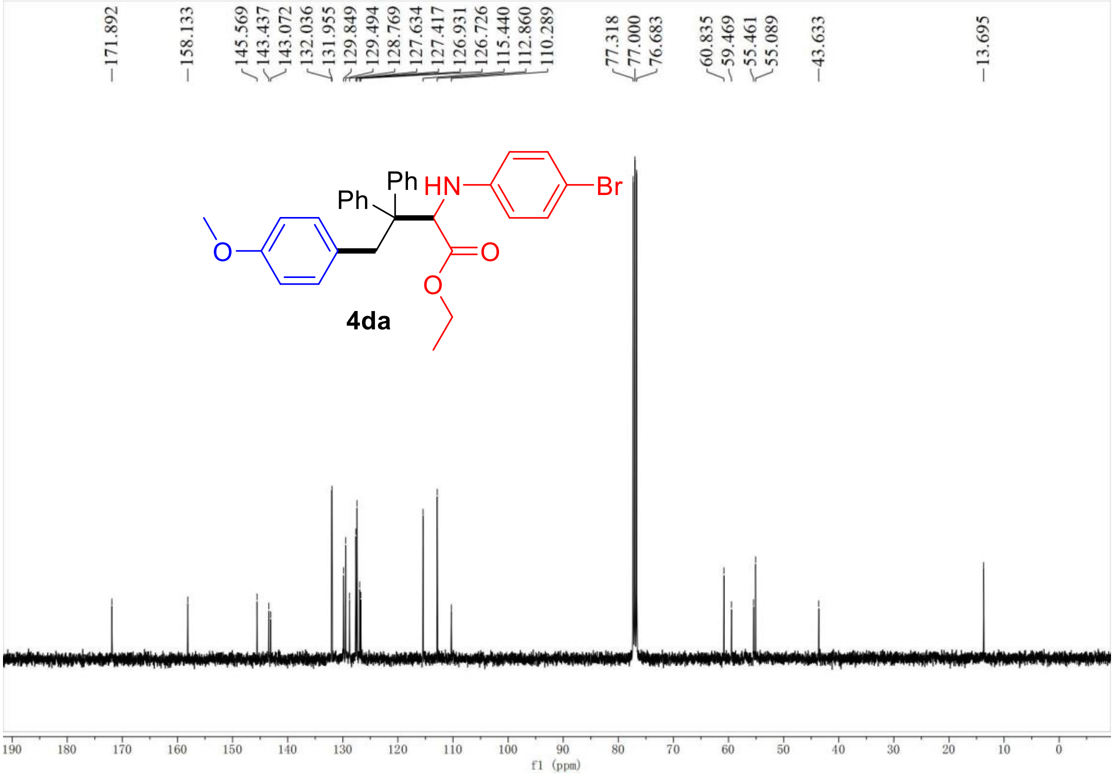

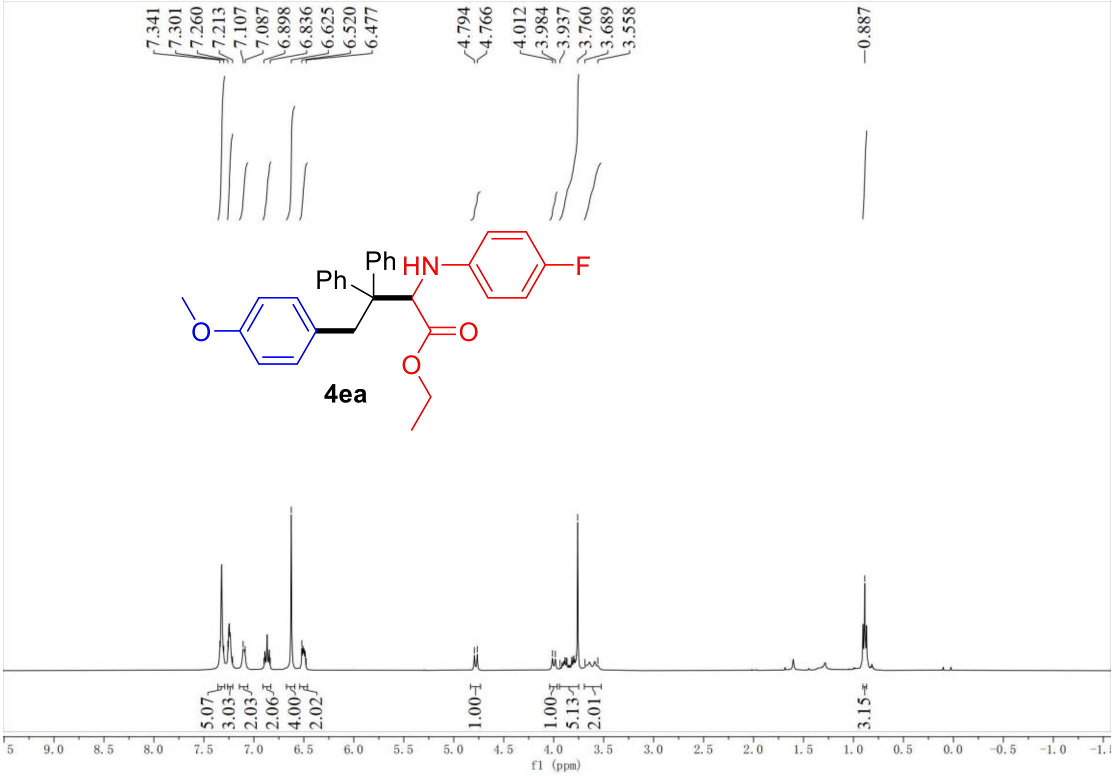

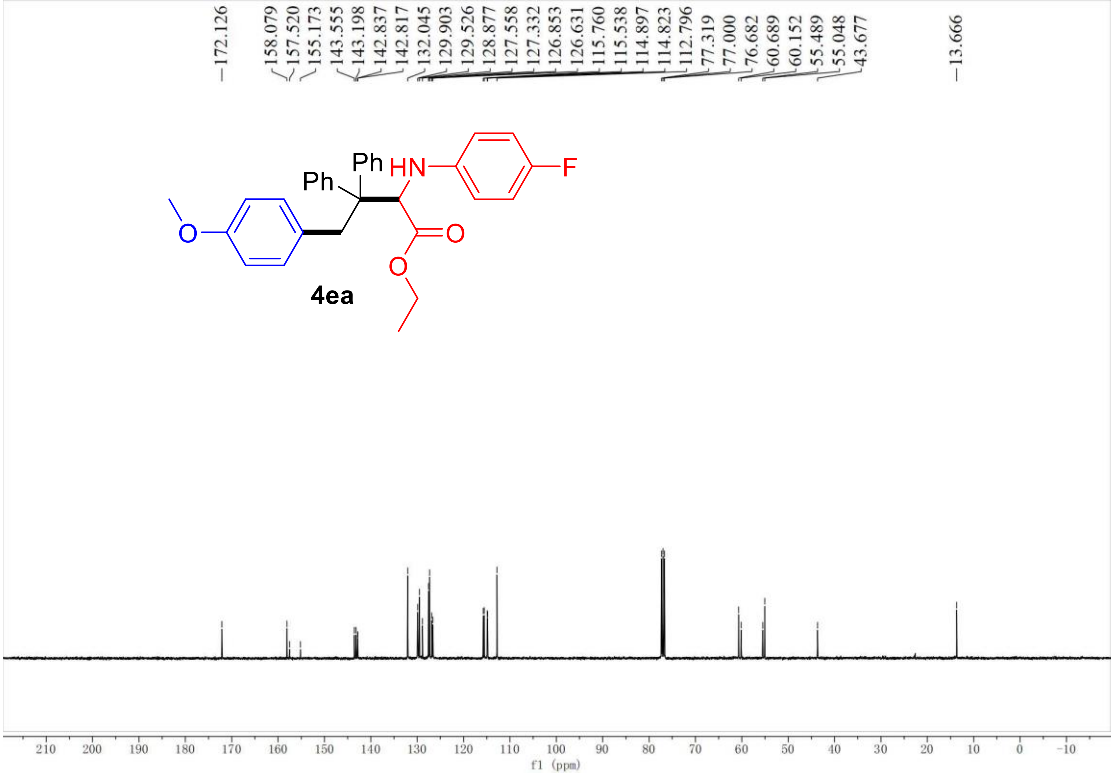

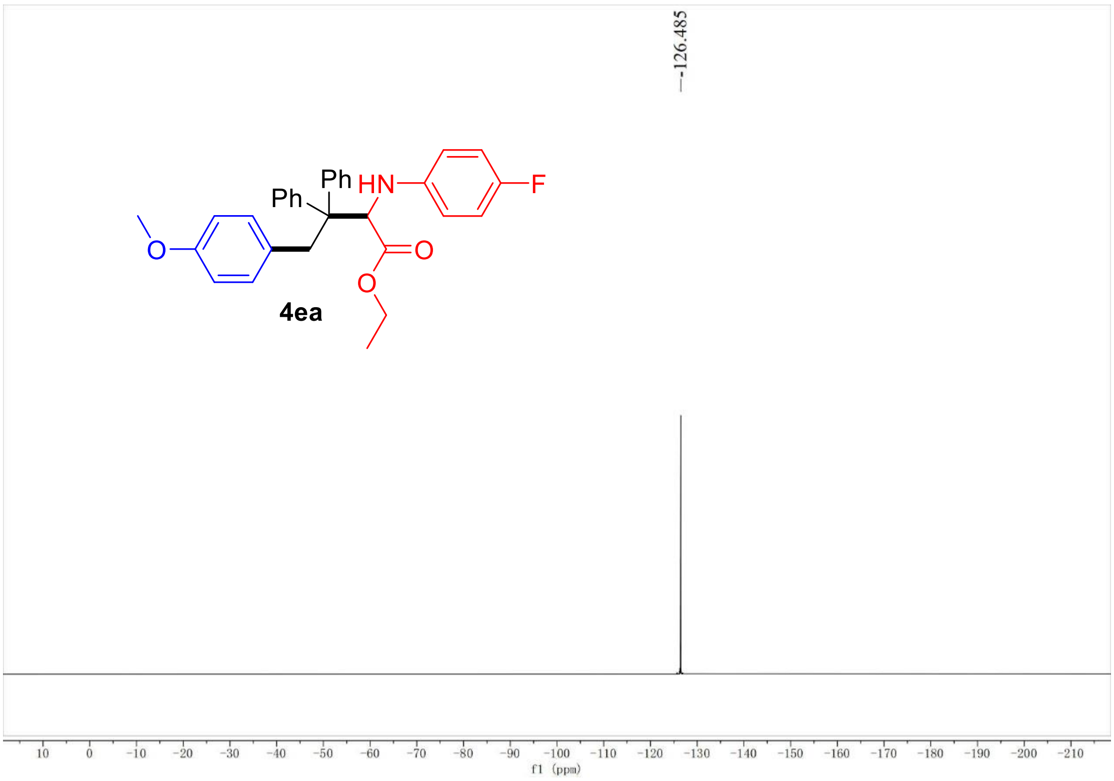
-126.485
Ph
Ph
HN
F
O
O
O
4ea
f1 (ppm)

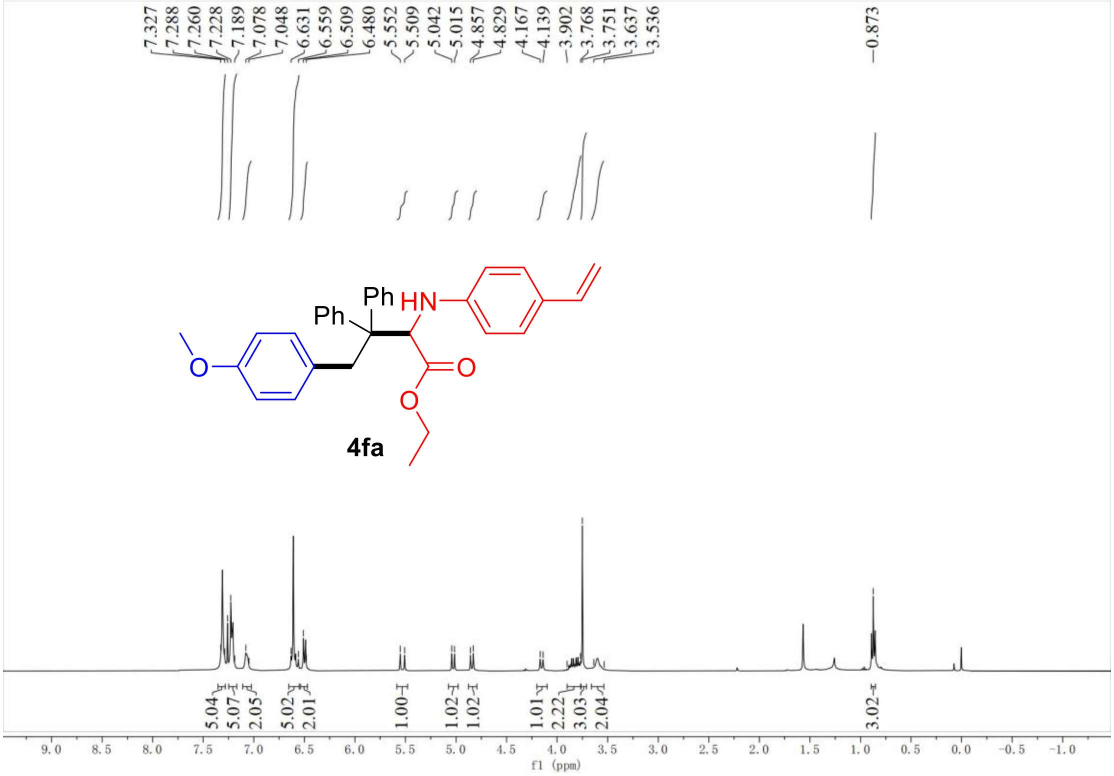
7.327
7.288
7.260
7.228
7.189
7.078
7.048
6.631
6.559
6.509
6.480
5.552
5.509
5.042
5.015
4.857
4.829
4.167
4.139
3.902
3.768
3.751
3.637
3.536
0.873
Ph
Ph
HN
O
O
O
4fa
5.04
5.07
2.05
5.02
2.01
1.00
1.02
1.02
1.01
2.22
3.03
2.04
3.02
f1 (ppm)

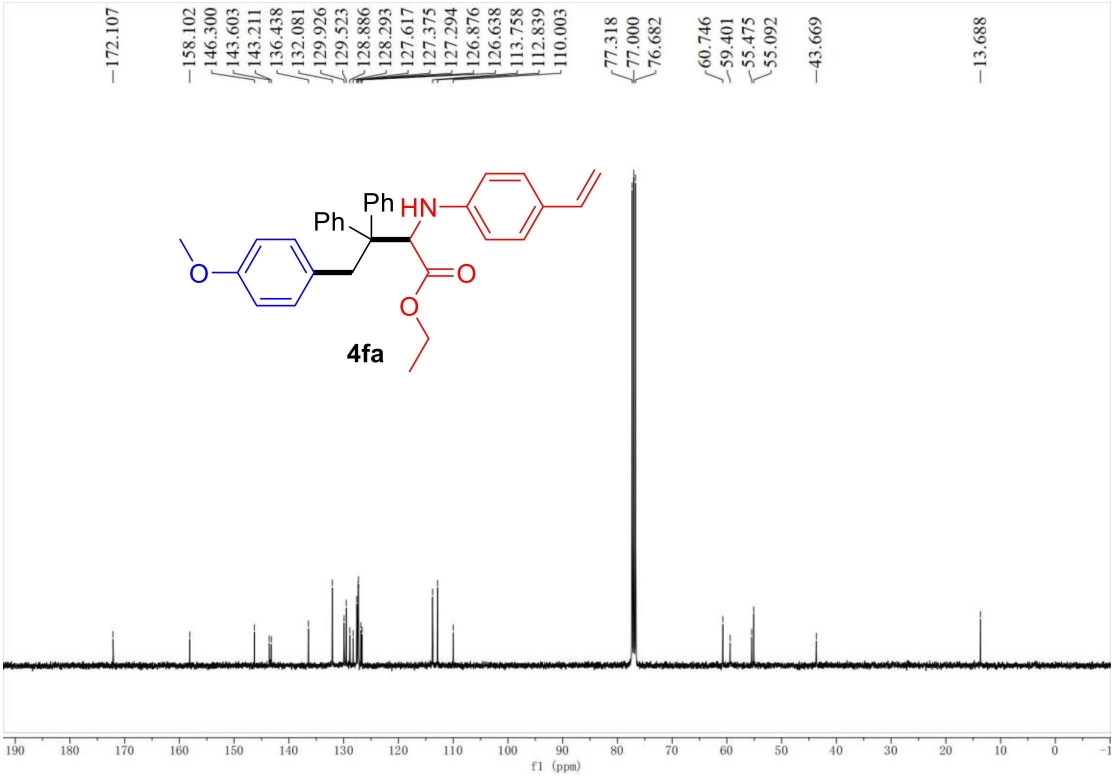
172.107
158.102
146.300
143.603
143.211
136.438
132.081
129.926
129.523
128.886
128.293
127.617
127.375
127.294
126.876
126.638
113.758
112.839
110.003
77.318
77.000
76.682
60.746
59.401
55.475
55.092
43.669
13.688
Ph
Ph
HN
O
O
O
4fa
f1 (ppm)

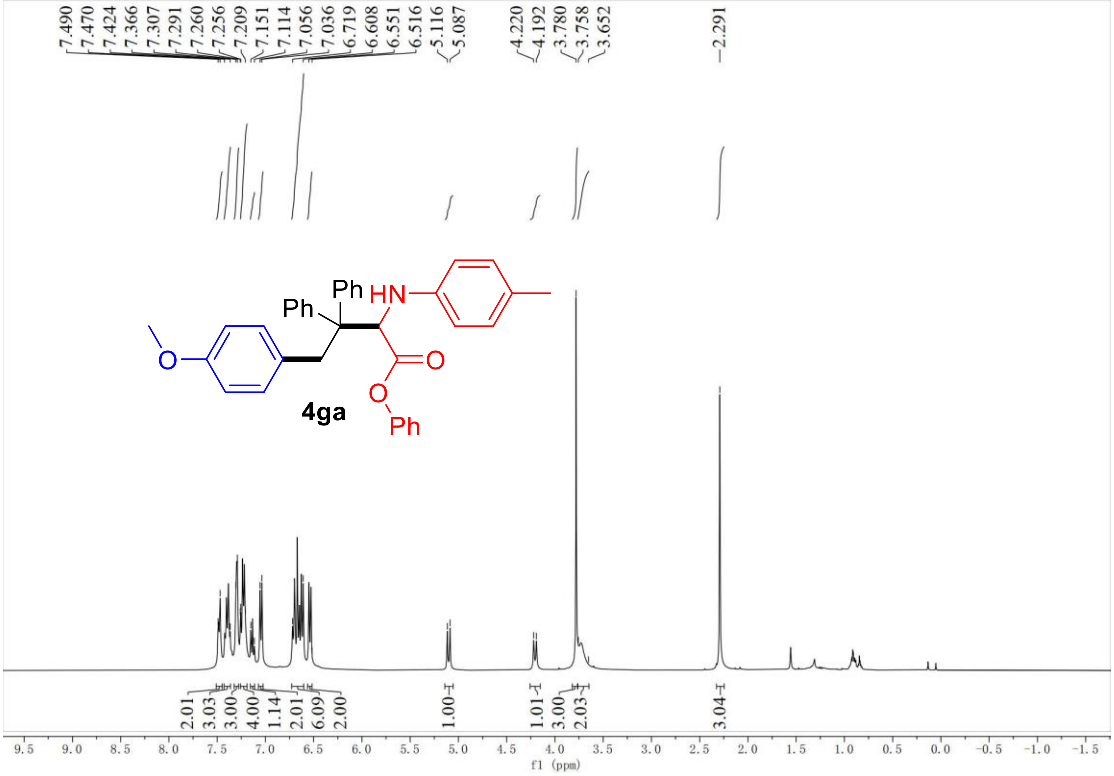
7.490
7.470
7.424
7.366
7.307
7.291
7.260
7.256
7.209
7.151
7.114
7.056
7.036
6.719
6.608
6.551
6.516
5.116
5.087
4.220
4.192
3.780
3.758
3.652
2.291
Ph
Ph
HN
O
O
O
Ph
4ga
2.01
3.03
3.00
4.00
1.14
2.01
6.09
2.00
1.00
1.01
3.00
2.03
3.04
f1 (ppm)

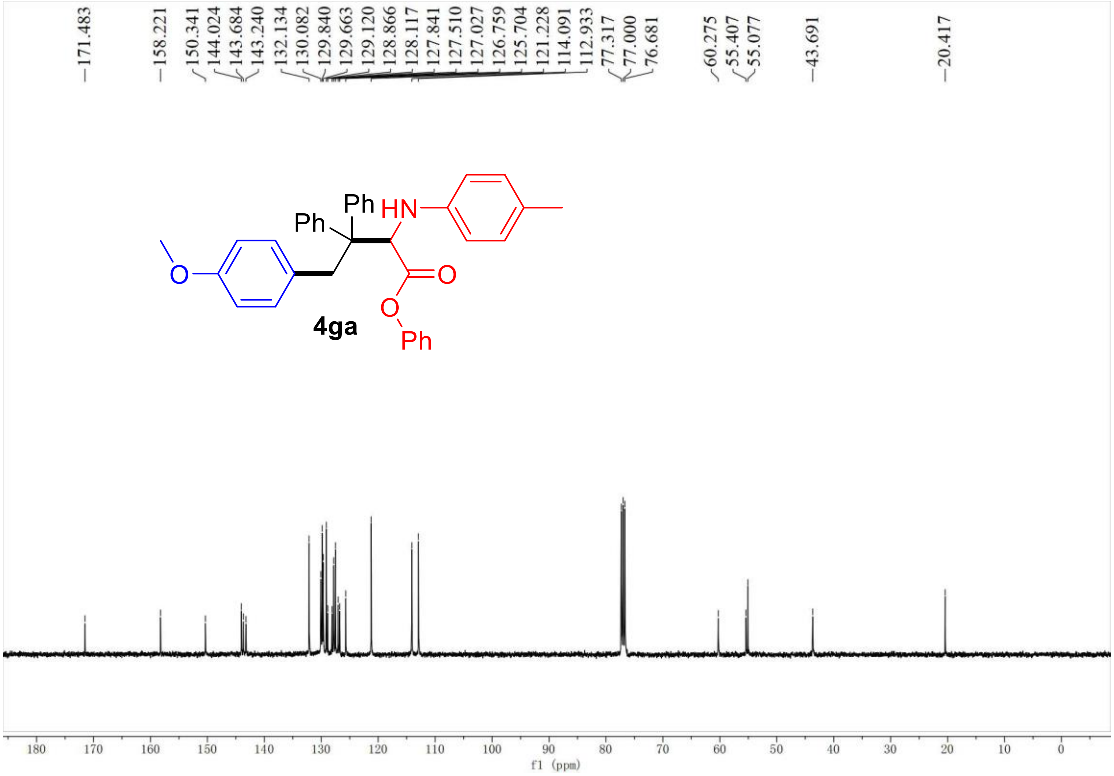

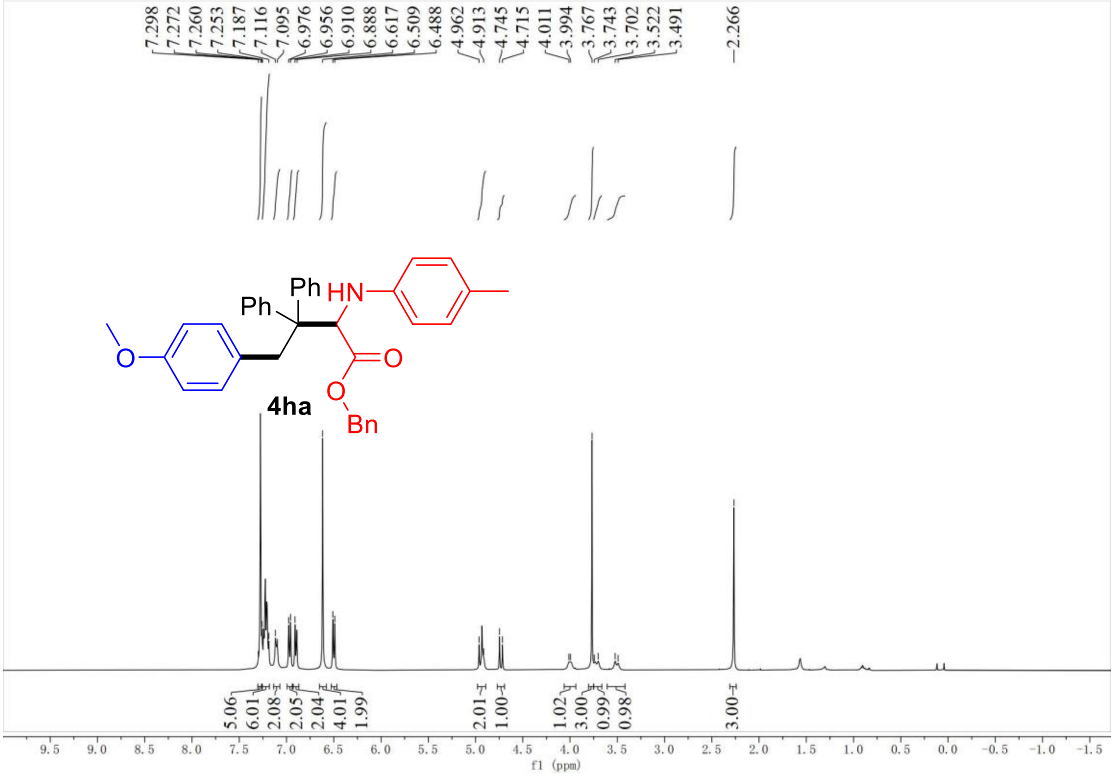

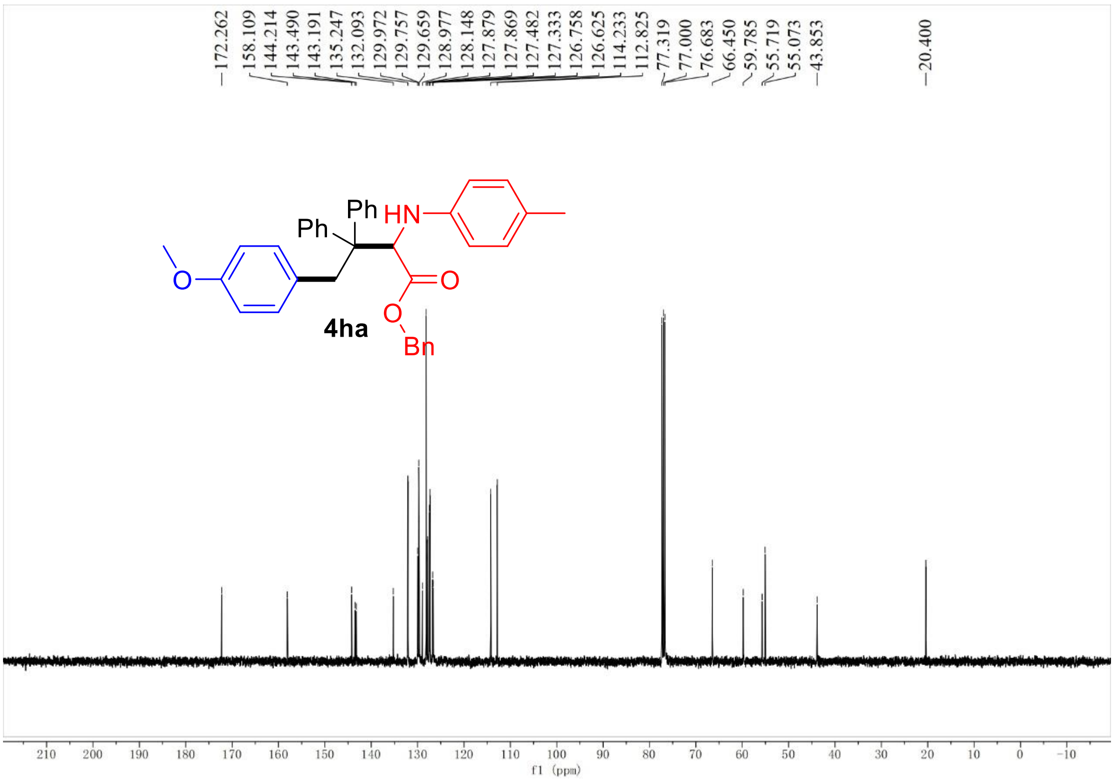

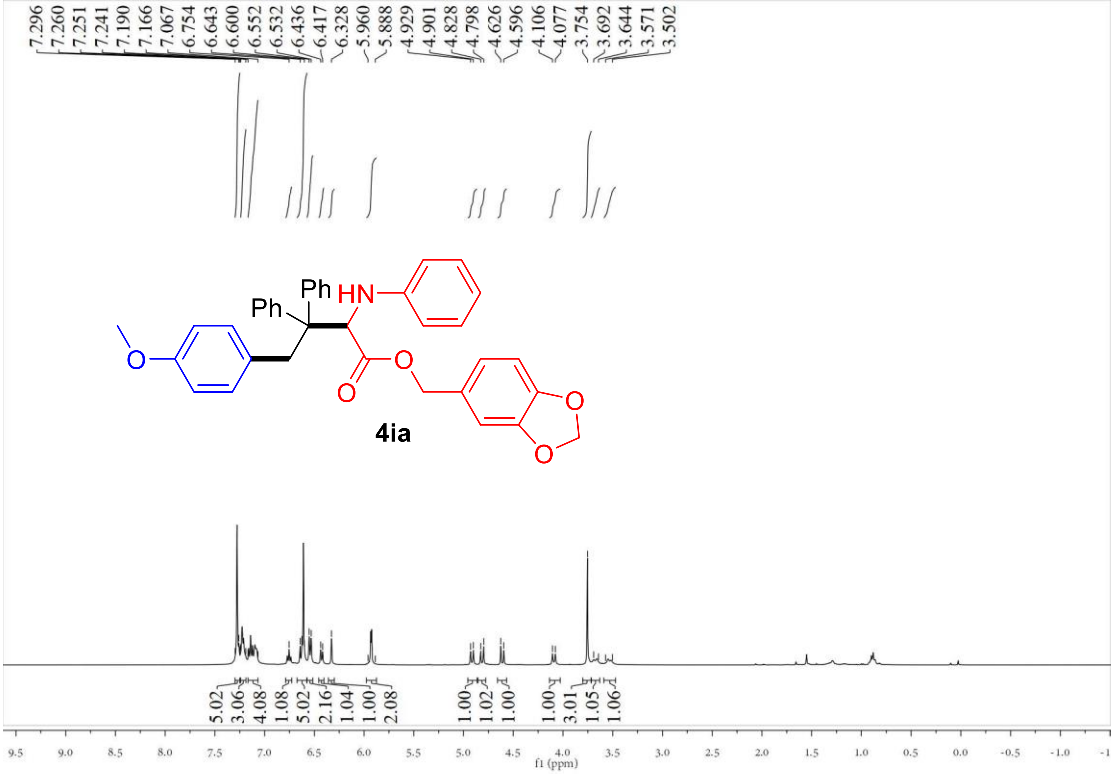

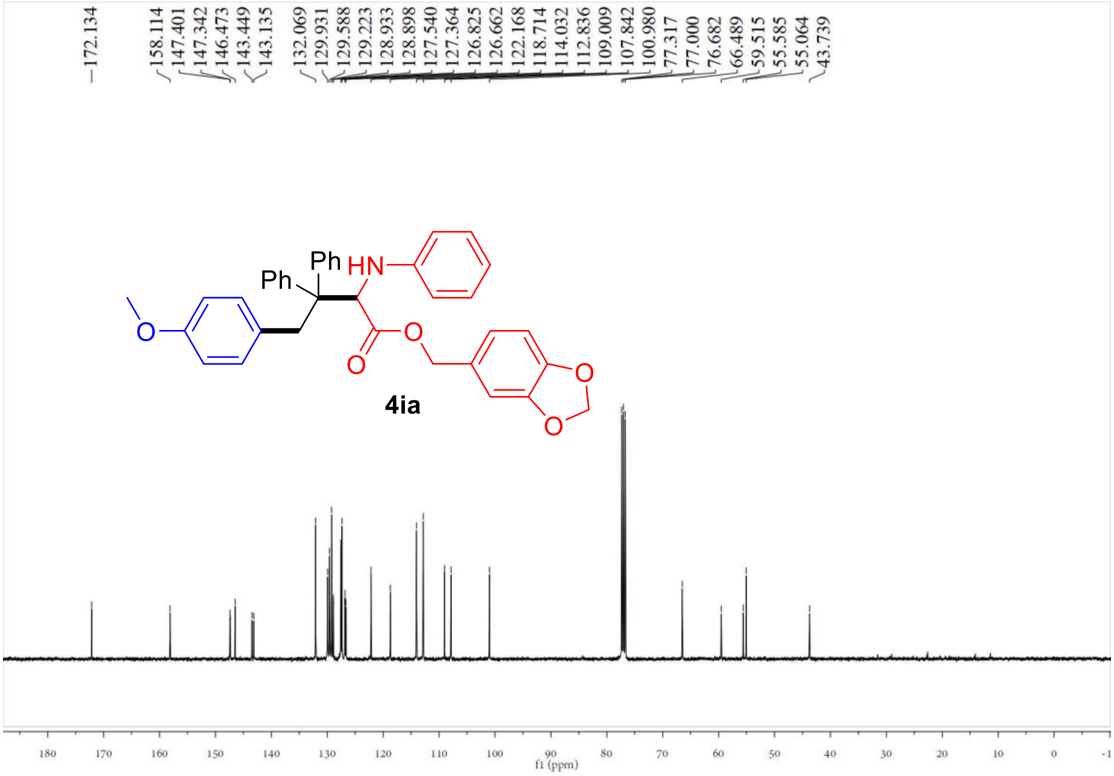

172.134
158.114
147.401
147.342
146.473
143.449
143.135
132.069
129.931
129.588
129.223
128.933
128.898
127.540
127.364
126.825
126.662
122.168
118.714
114.032
112.836
109.009
107.842
100.980
77.317
77.000
76.682
66.489
59.515
55.585
55.064
43.739
Ph
Ph
HN
O
O
O
O
O
4ia
f1 (ppm)

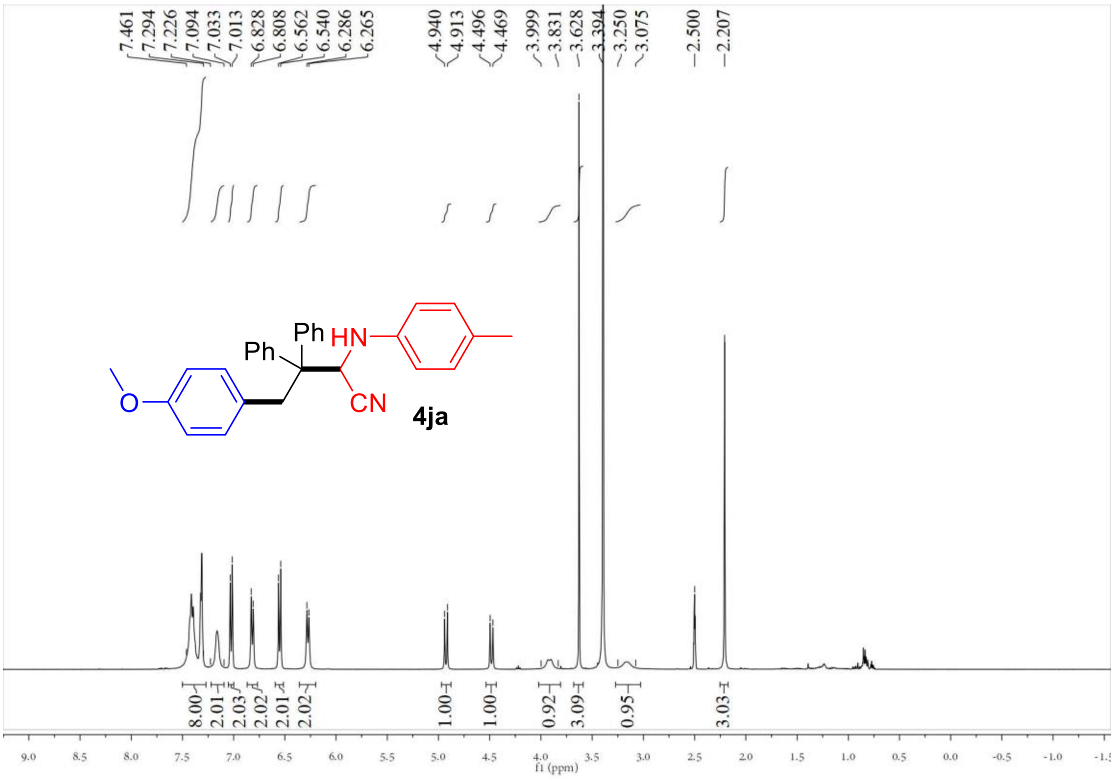

7.461
7.294
7.226
7.094
7.033
7.013
6.828
6.808
6.562
6.540
6.286
6.265
4.940
4.913
4.496
4.469
3.999
3.831
3.628
3.394
3.250
3.075
2.500
2.207
Ph
Ph
HN
O
CN
4ja
8.00
2.01
2.03
2.02
2.01
2.02
1.00
1.00
0.92
3.09
0.95
3.03
f1 (ppm)

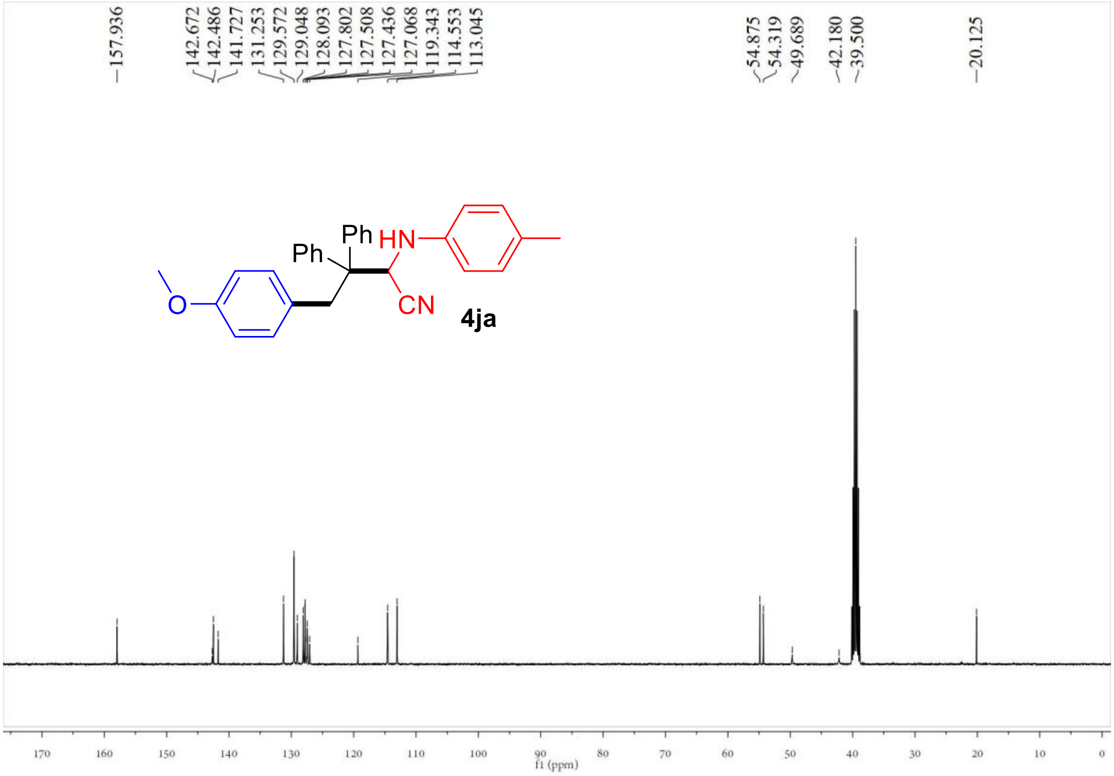

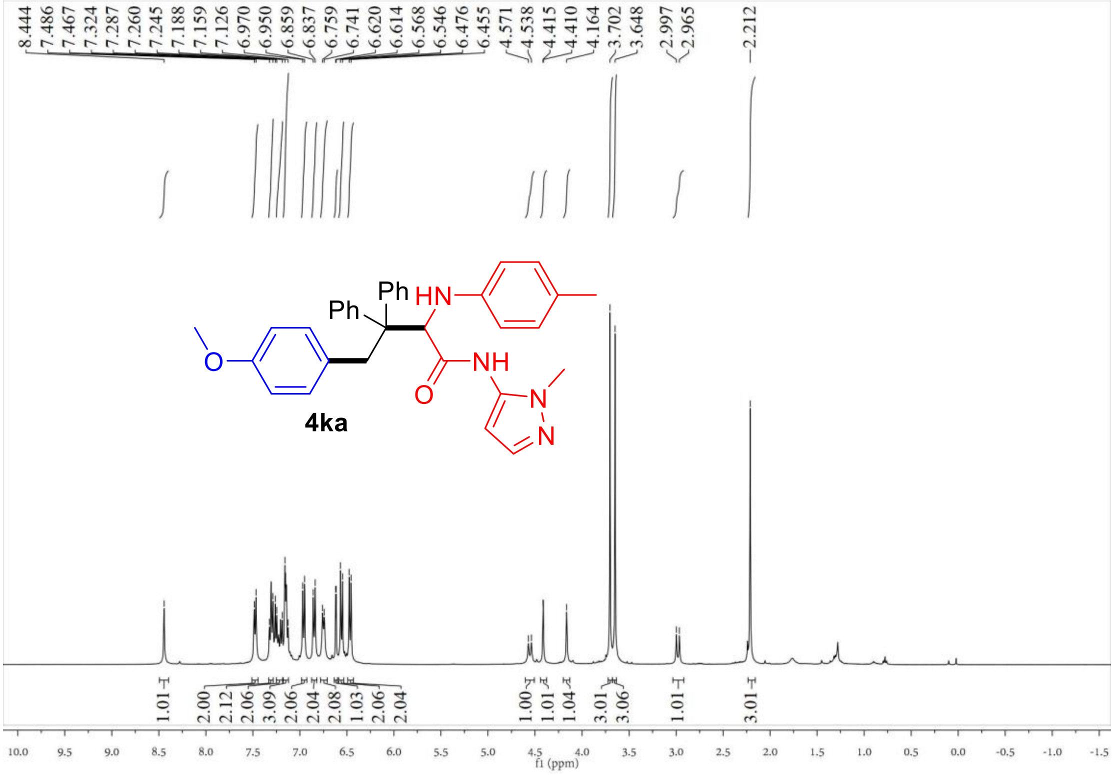

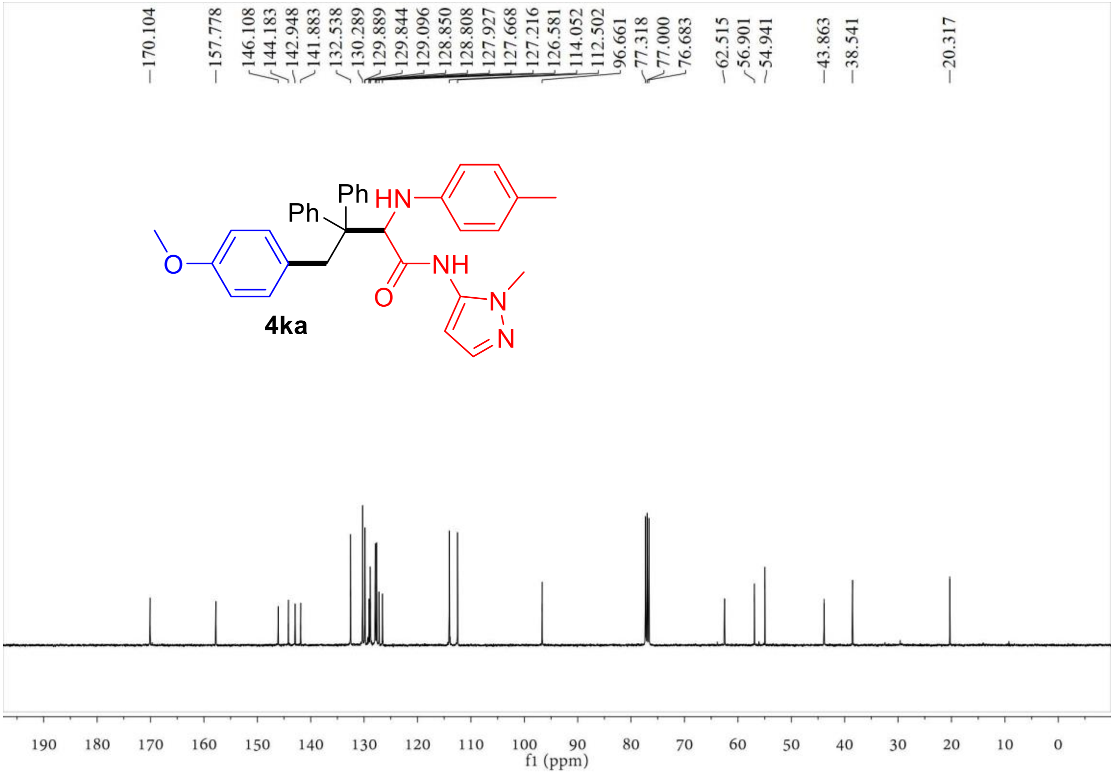
170.104
157.778
146.108
144.183
142.948
141.883
132.538
130.289
129.889
129.844
129.096
128.850
128.808
127.927
127.668
127.216
126.581
114.052
112.502
96.661
77.318
77.000
76.683
62.515
56.901
54.941
43.863
38.541
20.317
4ka
f1 (ppm)

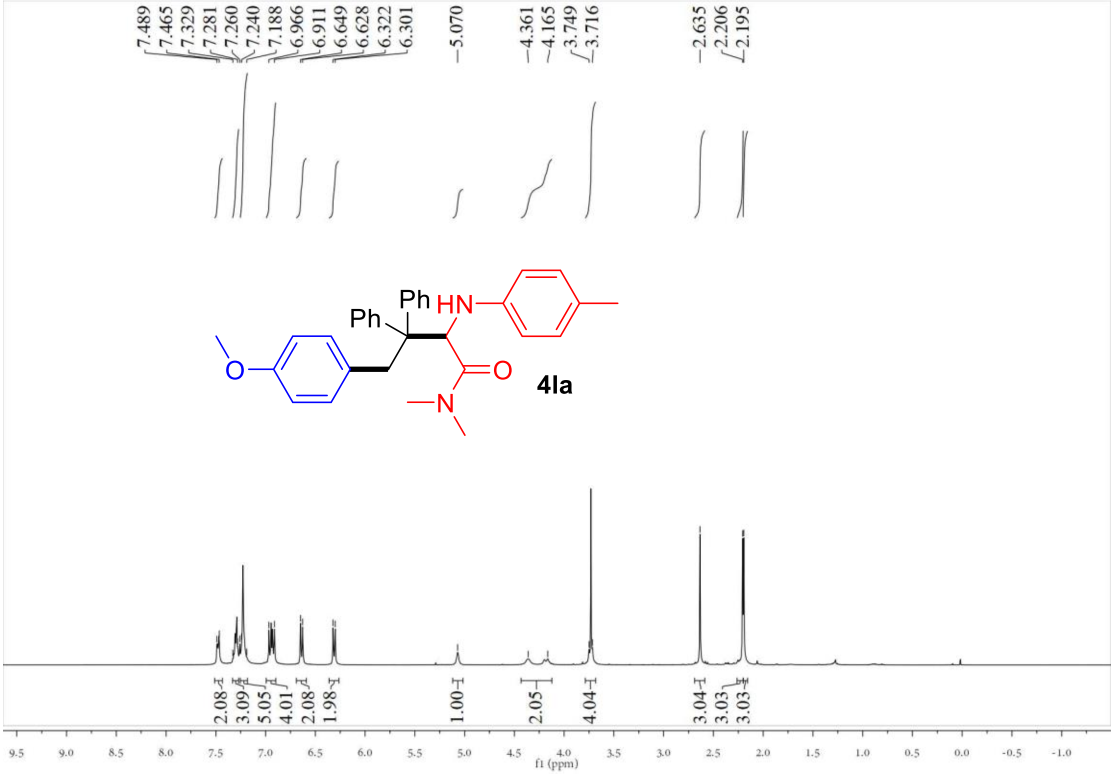
7.489
7.465
7.329
7.281
7.260
7.240
7.188
6.966
6.911
6.649
6.628
6.322
6.301
5.070
4.361
4.165
3.749
3.716
2.635
2.206
2.195
4la
2.08
3.09
5.05
4.01
2.08
1.98
1.00
2.05
4.04
3.04
3.03
3.03
f1 (ppm)

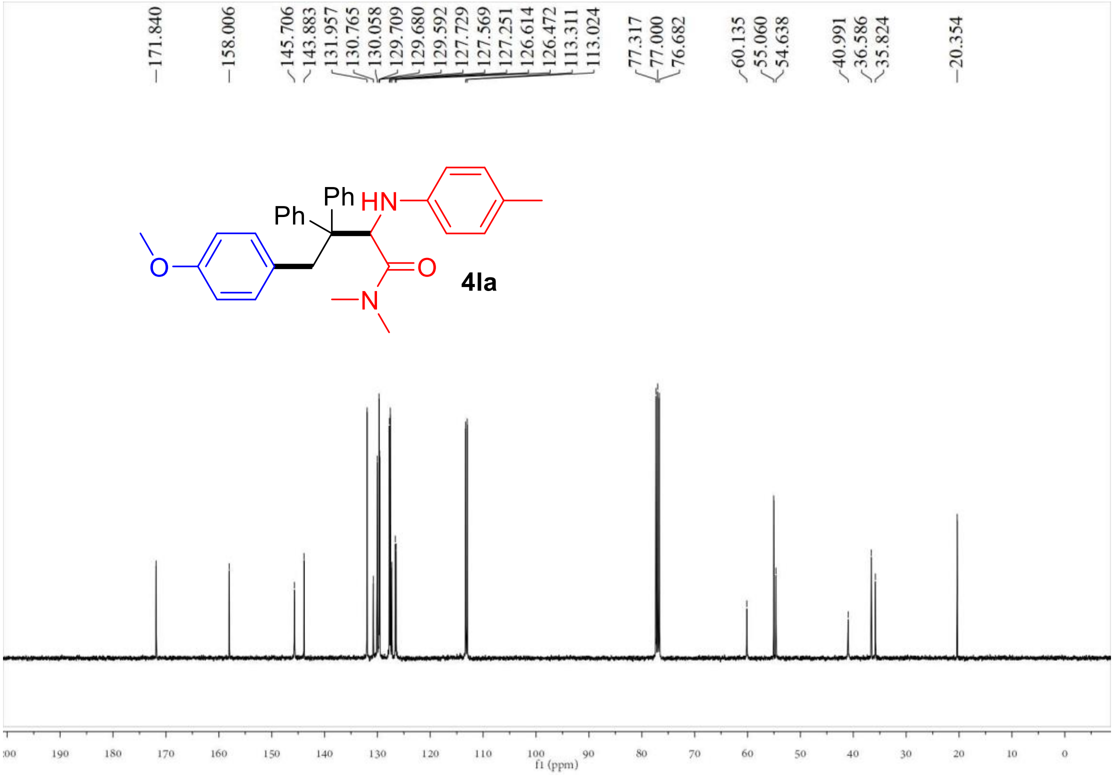

171.840
158.006
145.706
143.883
131.957
130.765
130.058
129.709
129.680
129.592
127.729
127.569
127.251
126.614
126.472
113.311
113.024
77.317
77.000
76.682
60.135
55.060
54.638
40.991
36.586
35.824
20.354
4la
f1 (ppm)

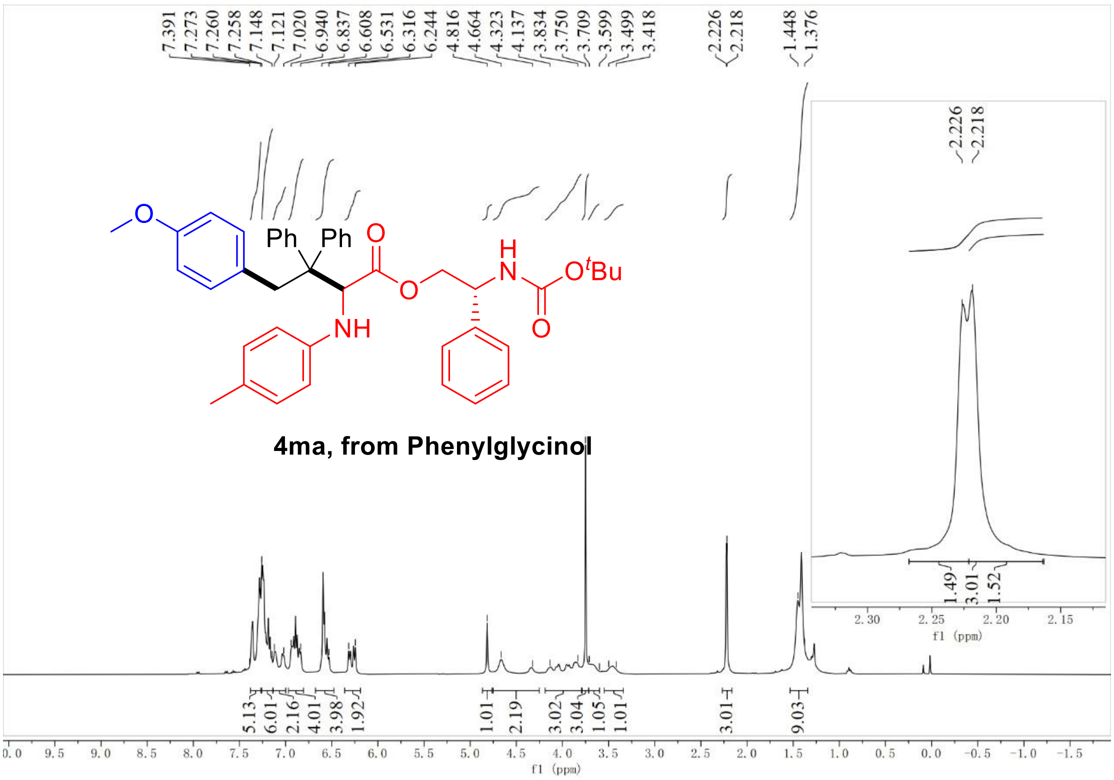

7.391
7.273
7.260
7.258
7.148
7.121
7.020
6.940
6.837
6.608
6.531
6.316
6.244
4.816
4.664
4.323
4.137
3.834
3.750
3.709
3.599
3.499
3.418
2.226
2.218
1.448
1.376
4ma, from Phenylglycinol
5.13
6.01
2.16
4.01
3.98
1.92
1.01
2.19
3.02
3.04
1.05
1.01
3.01
9.03
1.49
3.01
1.52
f1 (ppm)

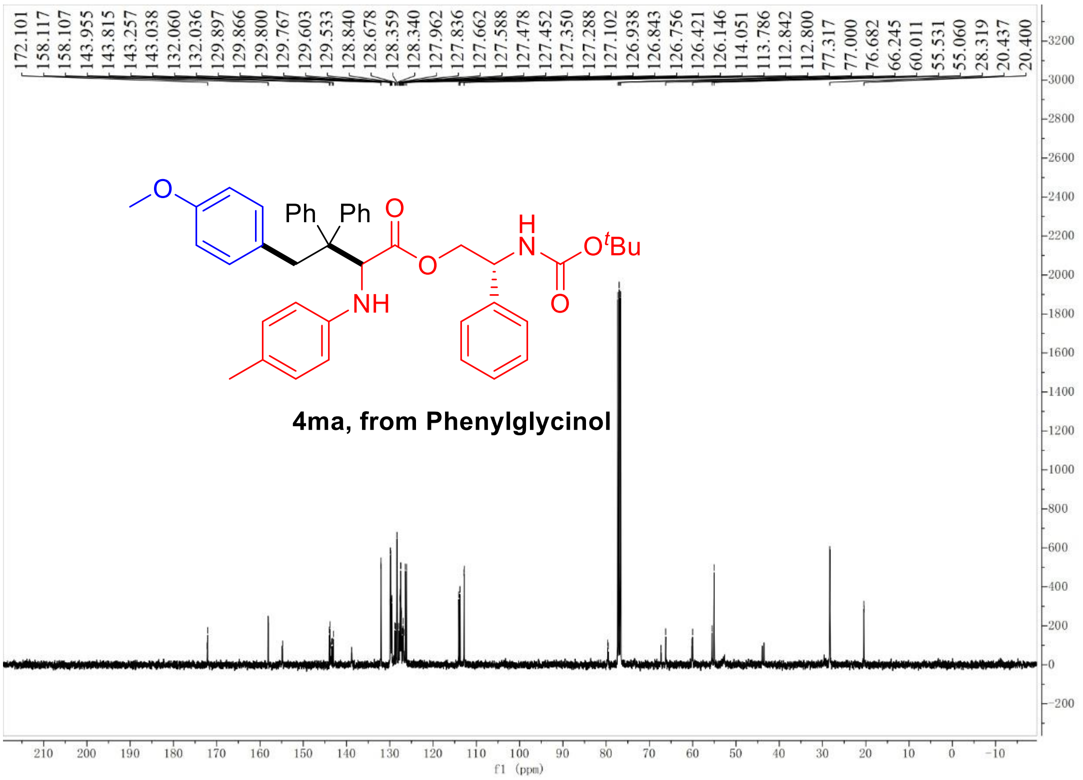
4ma, from Phenylglycinol
f1 (ppm)

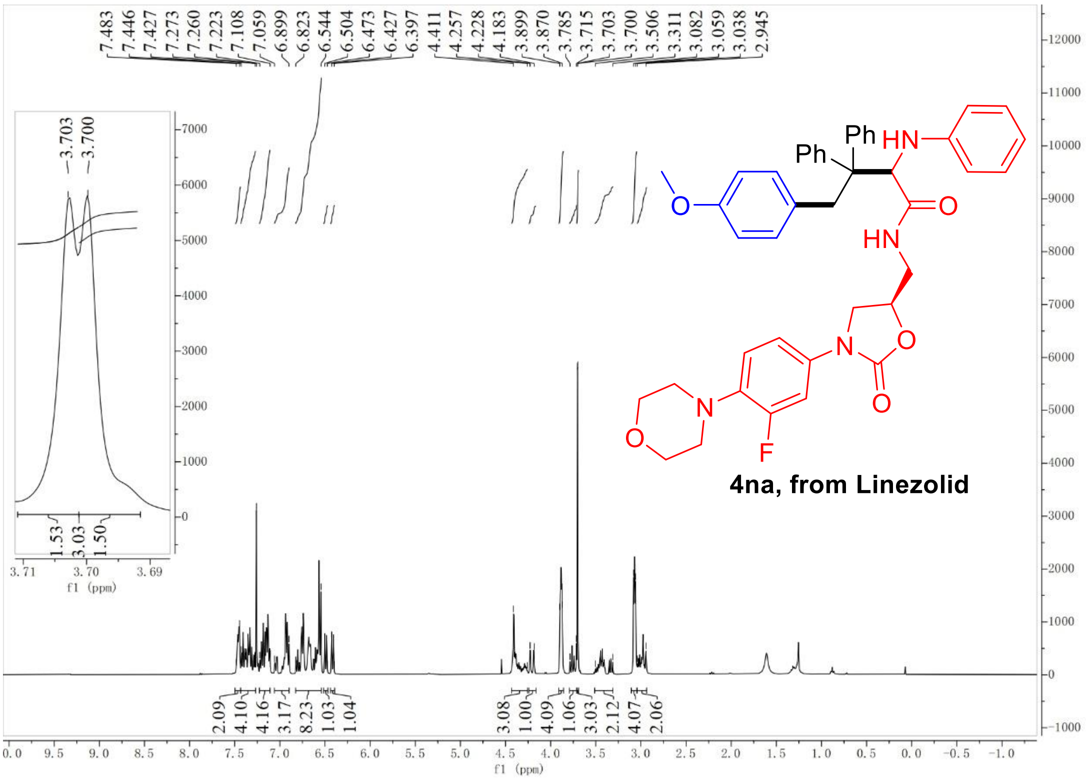
4na, from Linezolid
f1 (ppm)

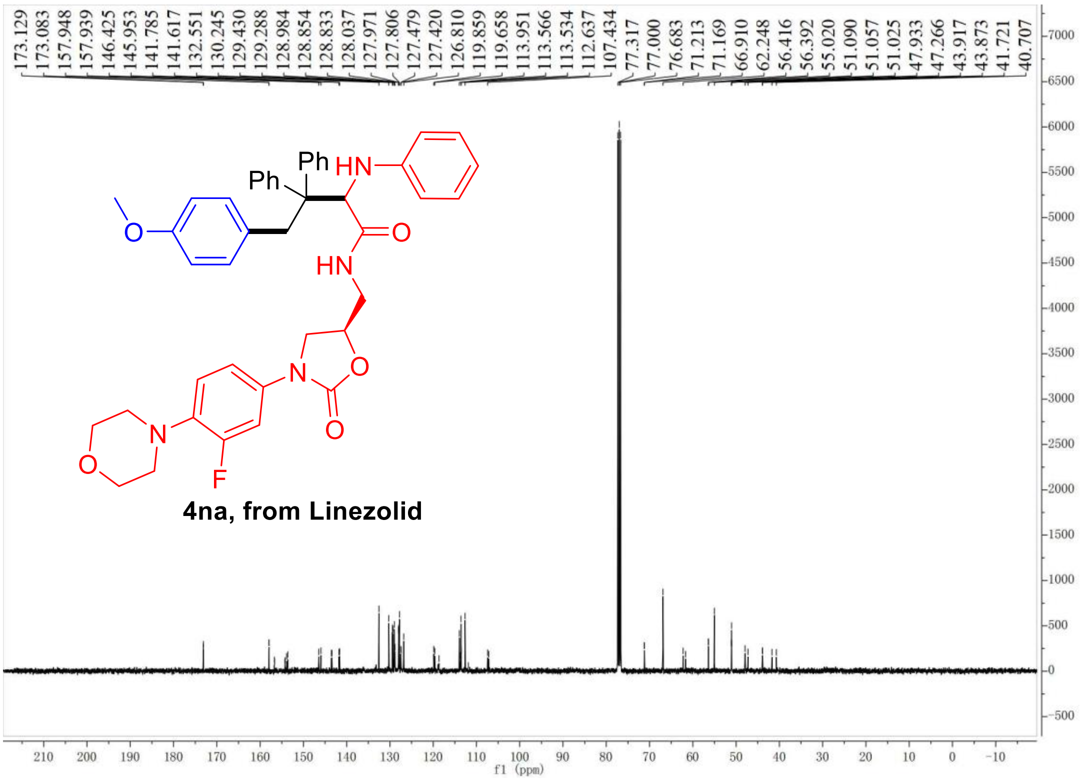
173.129
173.083
157.948
157.939
146.425
145.953
141.785
141.617
132.551
130.245
129.430
129.288
128.984
128.854
128.833
128.037
127.971
127.806
127.479
127.420
126.810
119.859
119.658
113.951
113.566
113.534
112.637
107.434
77.317
77.000
76.683
71.213
71.169
66.910
62.248
56.416
56.392
55.020
51.090
51.057
51.025
47.933
47.266
43.917
43.873
41.721
40.707
Ph
Ph
HN
O
O
HN
N
O
O
N
O
F
4na, from Linezolid
f1 (ppm)

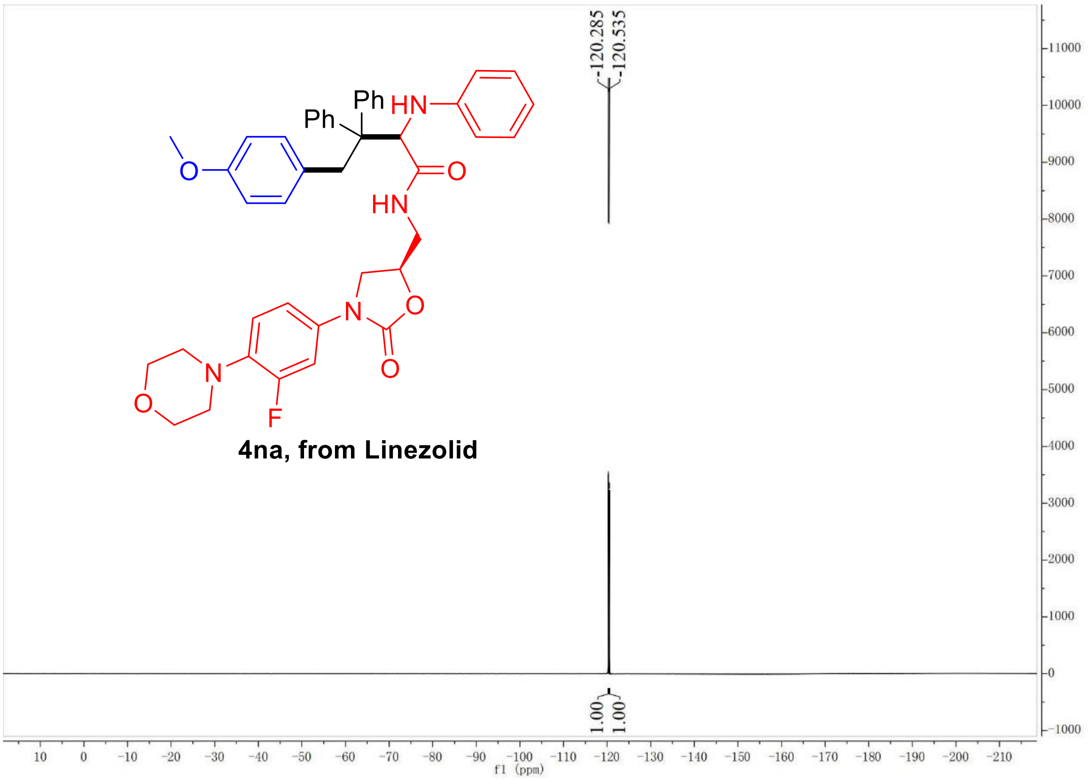
-120.285
-120.535
Ph
Ph
HN
O
O
HN
N
O
O
N
O
F
4na, from Linezolid
1.00
1.00
f1 (ppm)

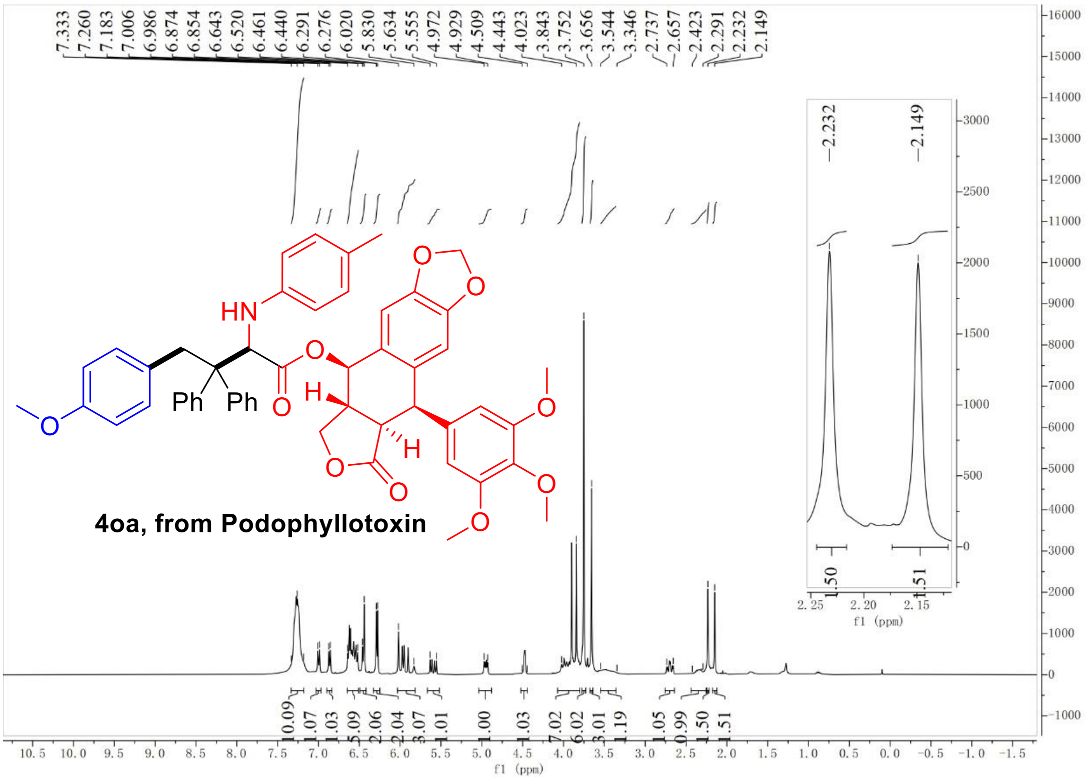
4oa, from Podophyllotoxin
f1 (ppm)

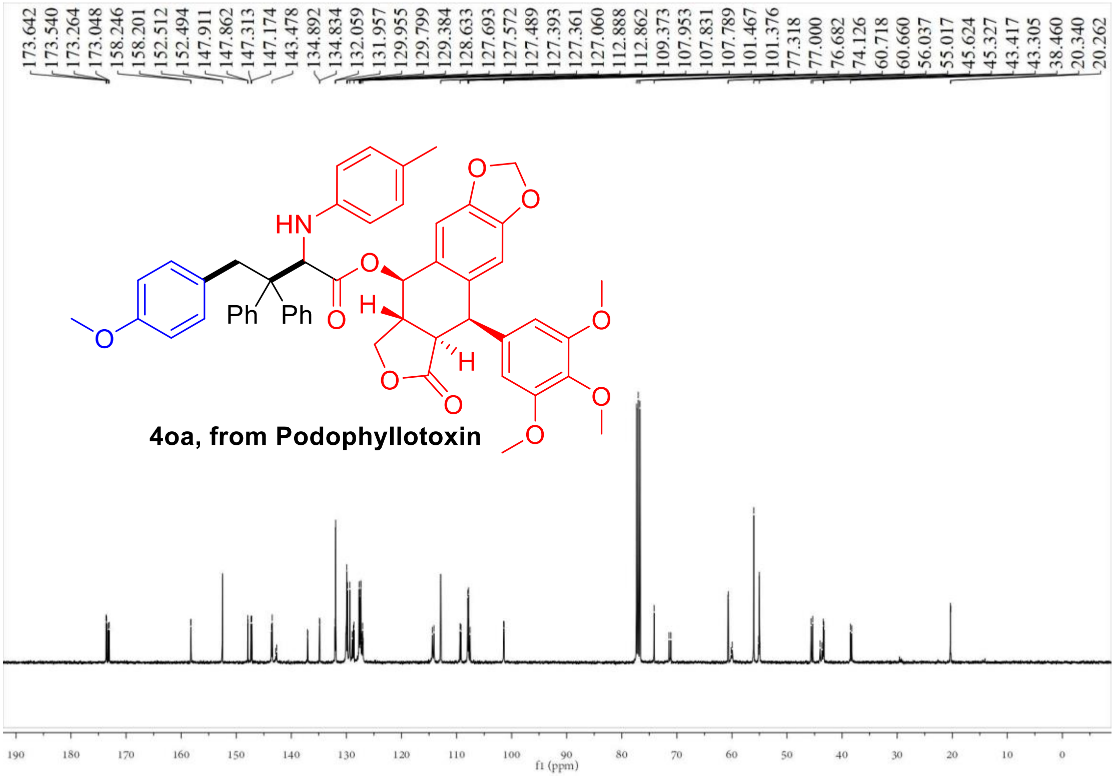
4oa, from Podophyllotoxin
f1 (ppm)

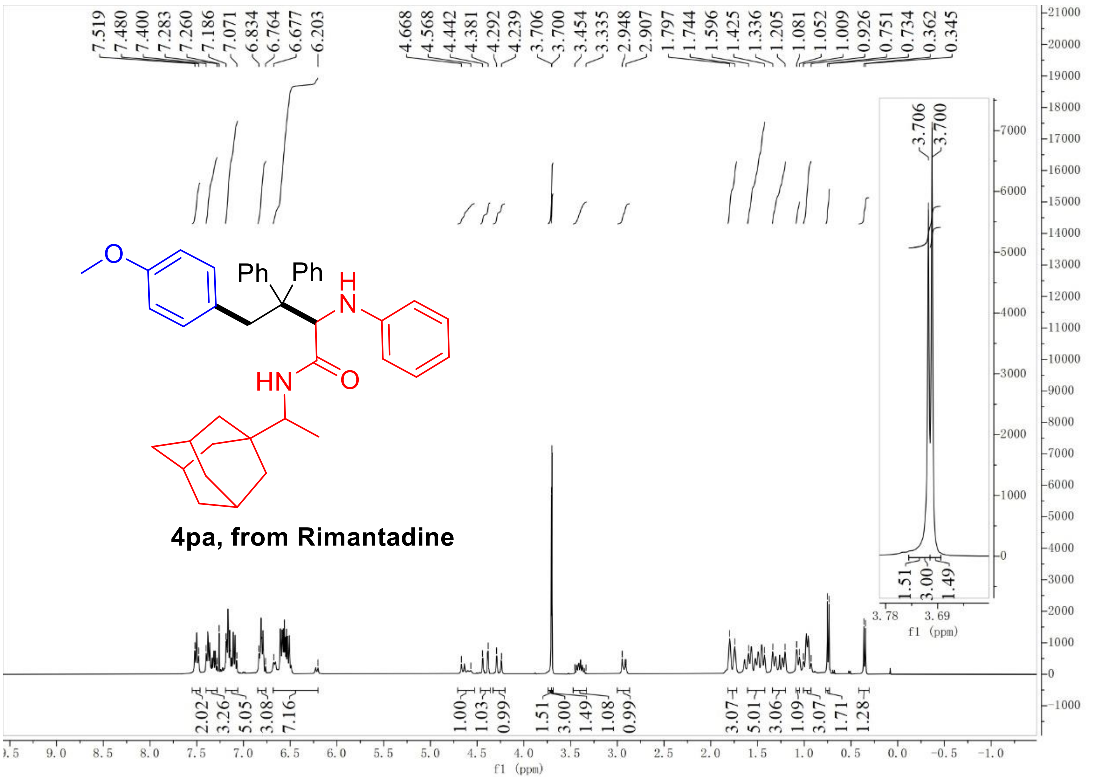
4pa, from Rimantadine

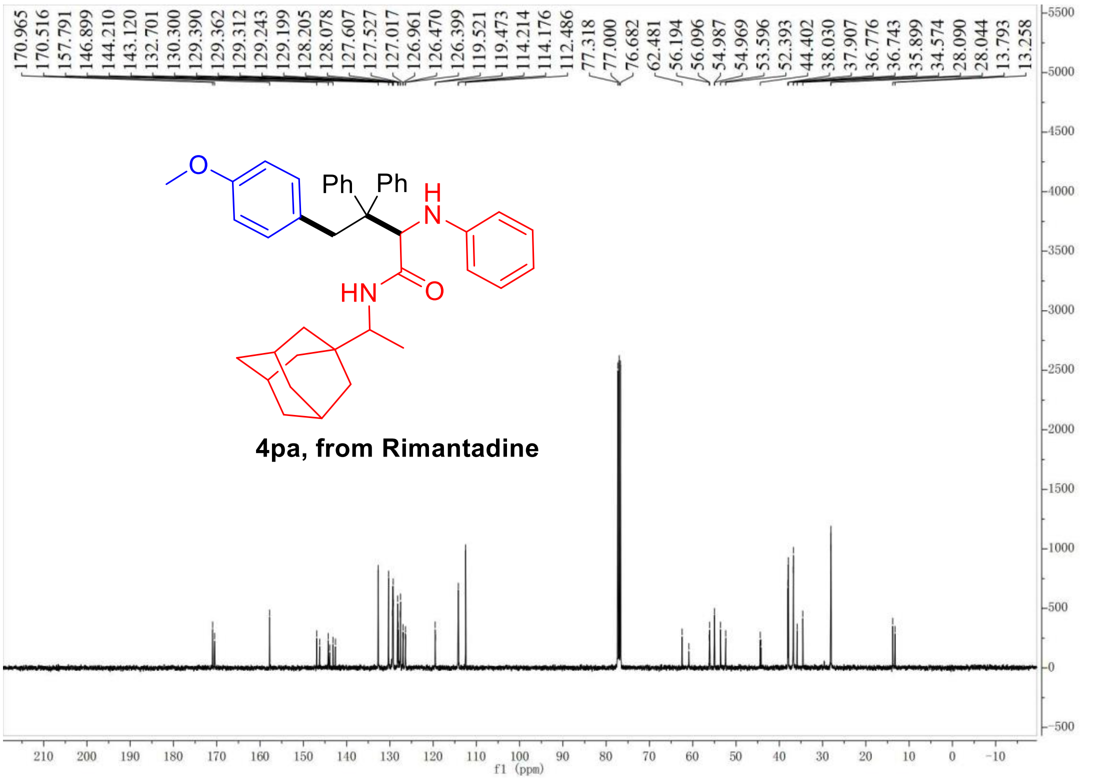
4pa, from Rimantadine

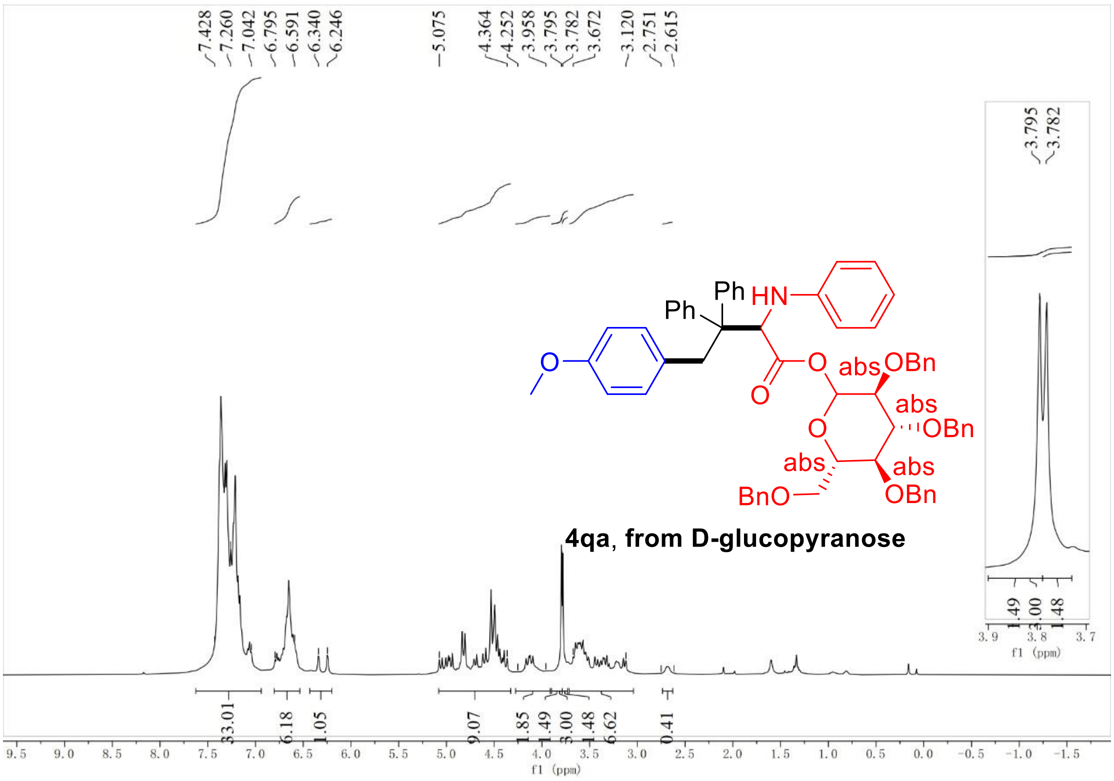
4qa, from D-glucopyranose
f1 (ppm)

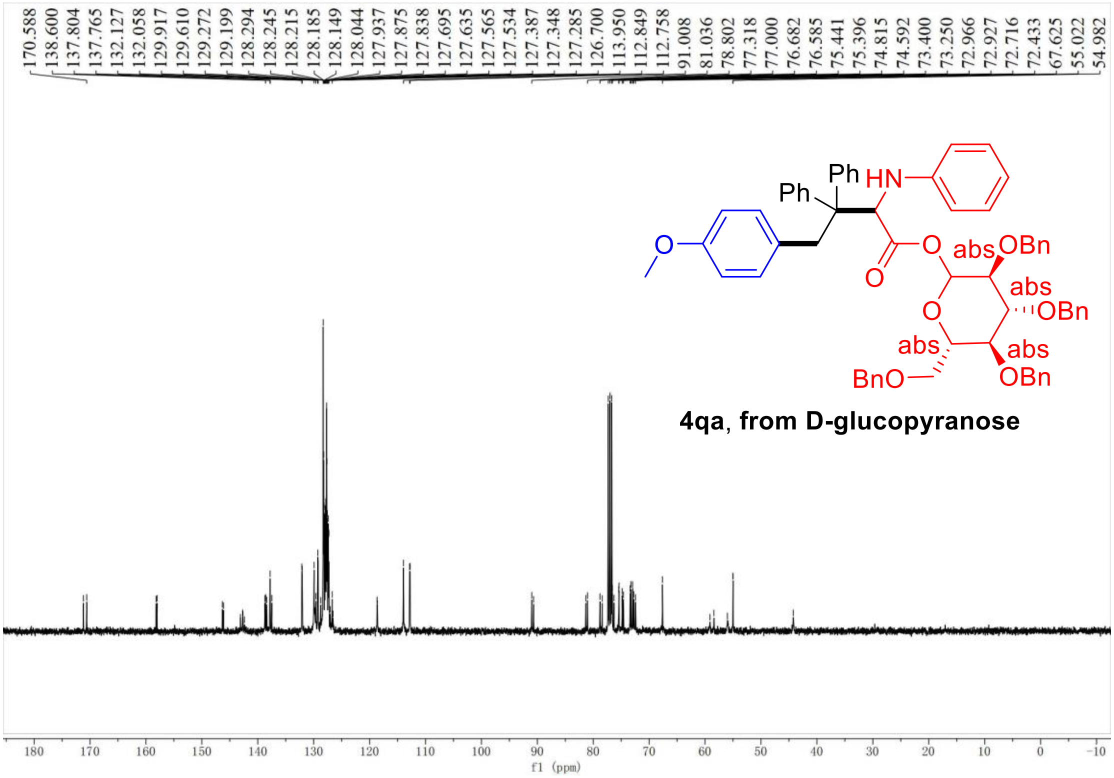
4qa, from D-glucopyranose
f1 (ppm)

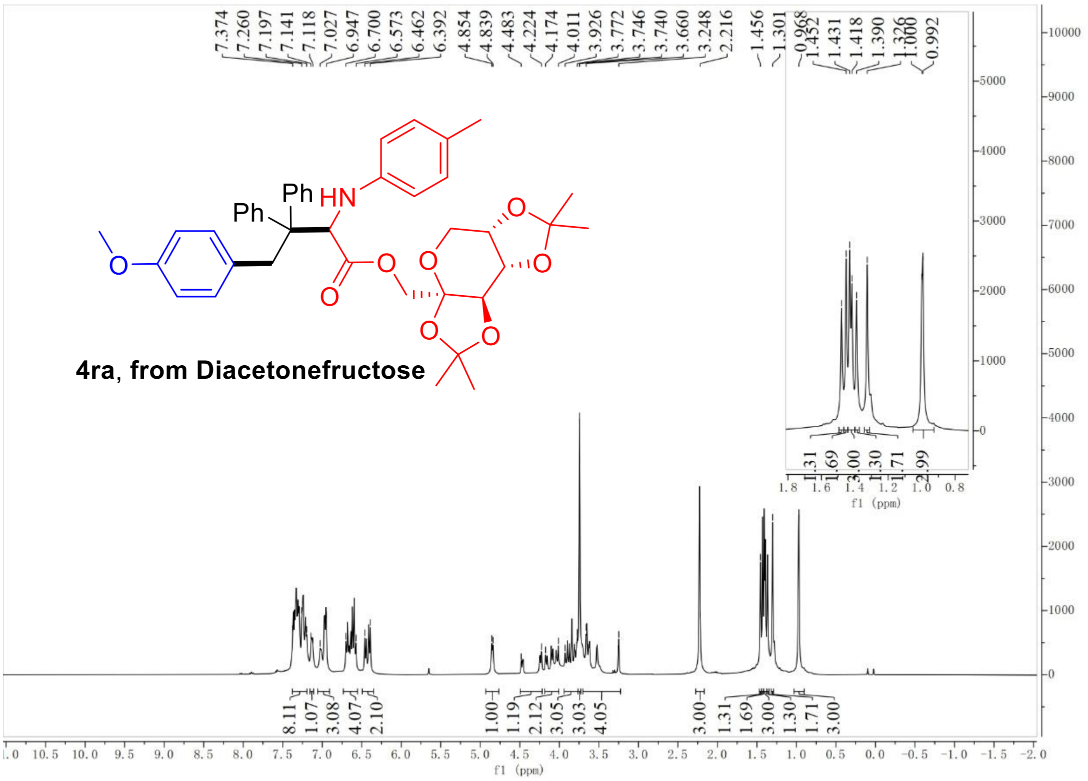
4ra, from Diacetonefructose
f1 (ppm)

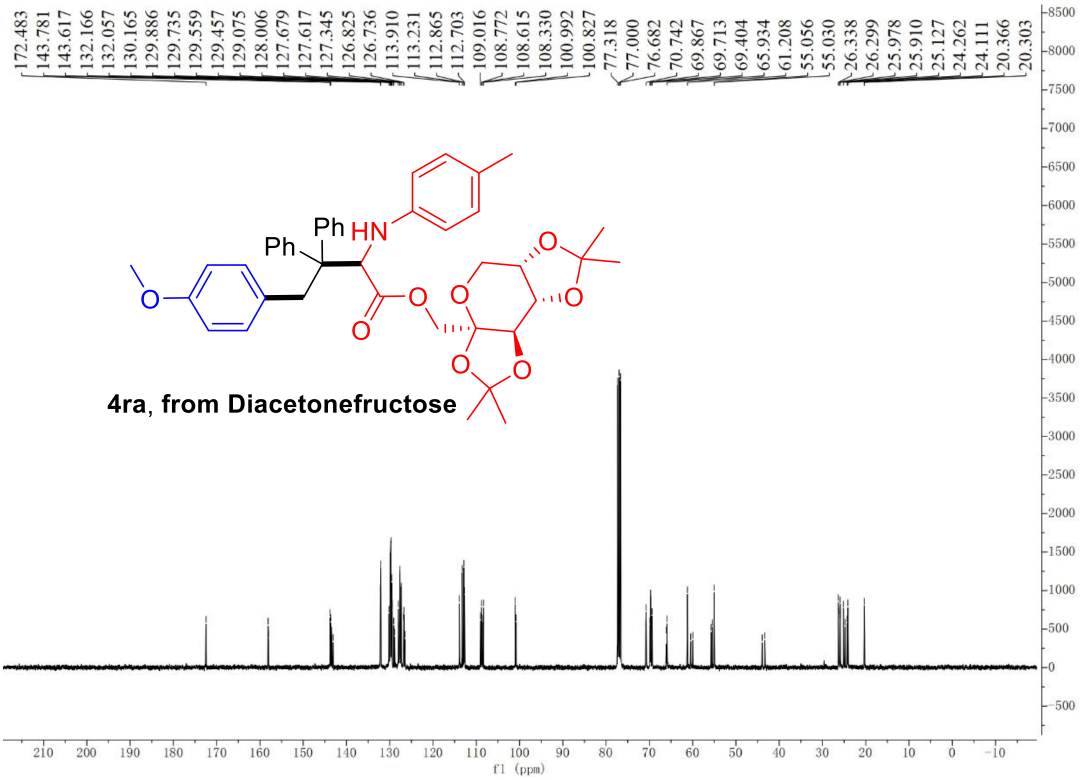
4ra, from Diacetonefructose
f1 (ppm)

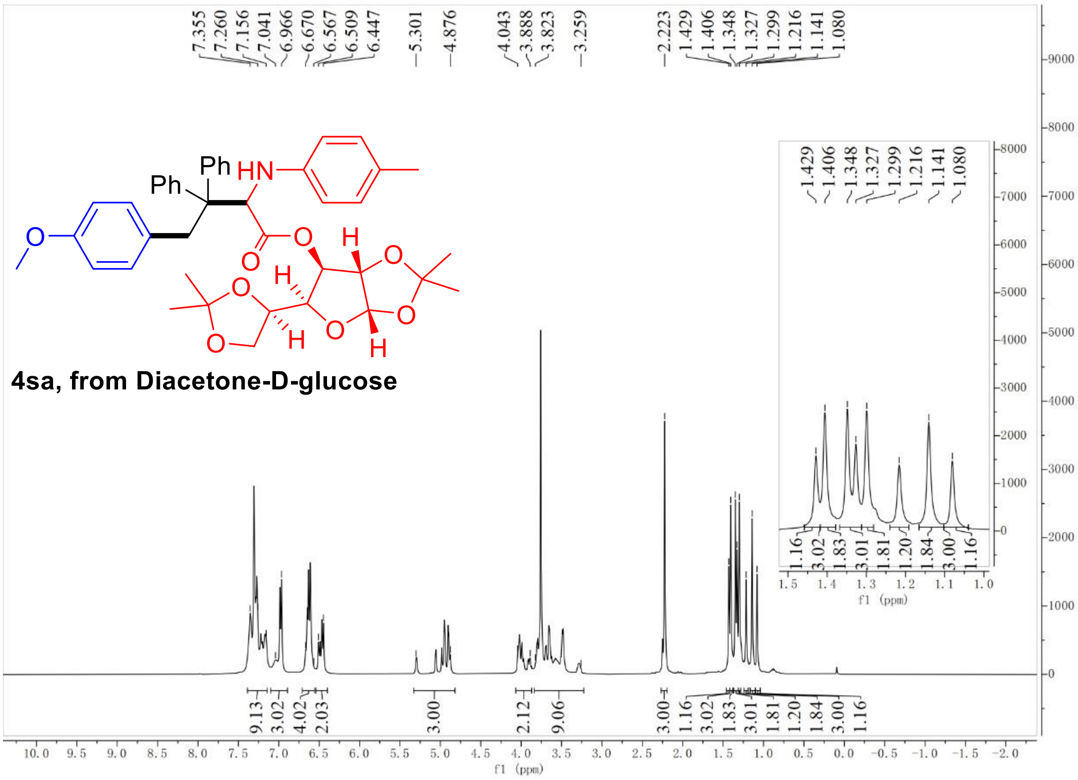
4sa, from Diacetone-D-glucose
f1 (ppm)

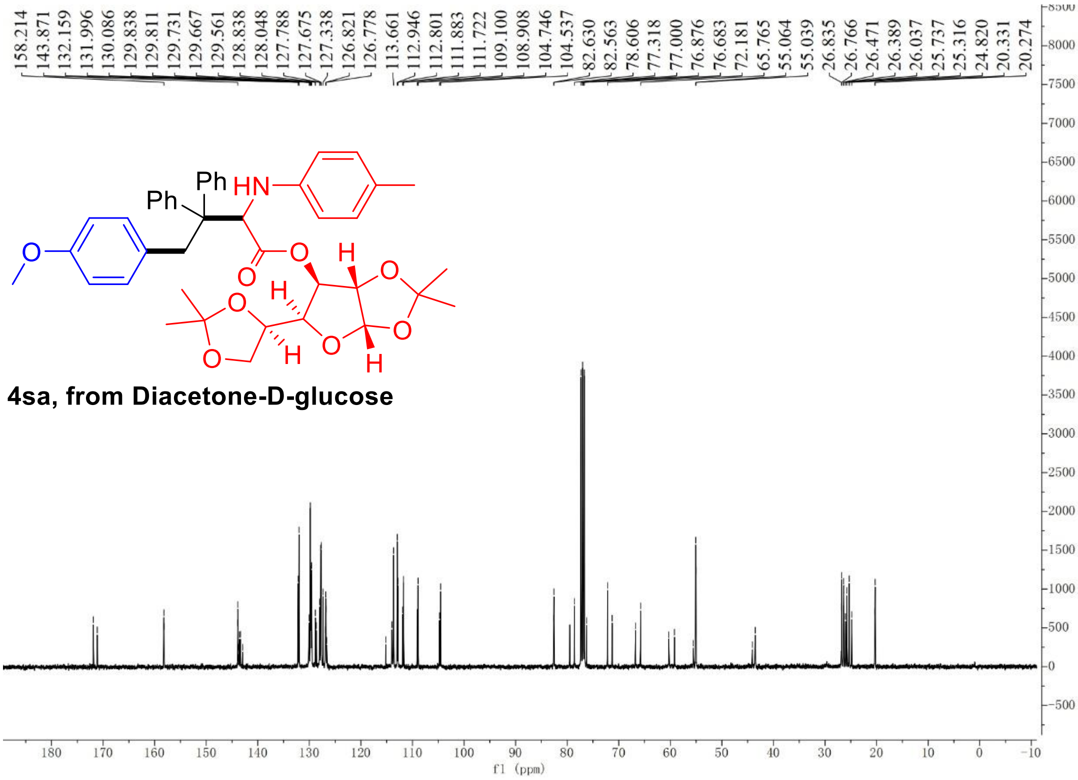
4sa, from Diacetone-D-glucose
f1 (ppm)

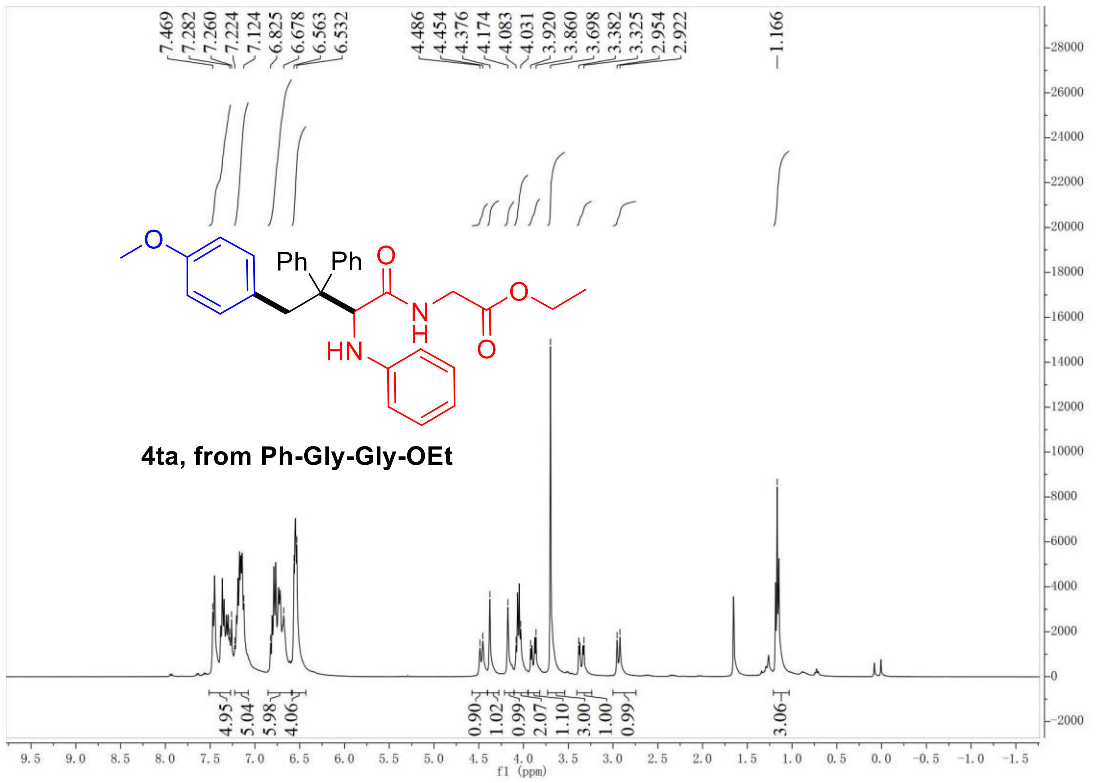
7.469
7.282
7.260
7.224
7.124
6.825
6.678
6.563
6.532
4.486
4.454
4.376
4.174
4.083
4.031
3.920
3.860
3.698
3.382
3.325
2.954
2.922
1.166
Ph
Ph
O
HN
N
H
4ta, from Ph-Gly-Gly-OEt
4.95
5.04
5.98
4.06
0.90
1.02
0.99
2.07
1.10
3.00
1.00
0.99
3.06
f1 (ppm)

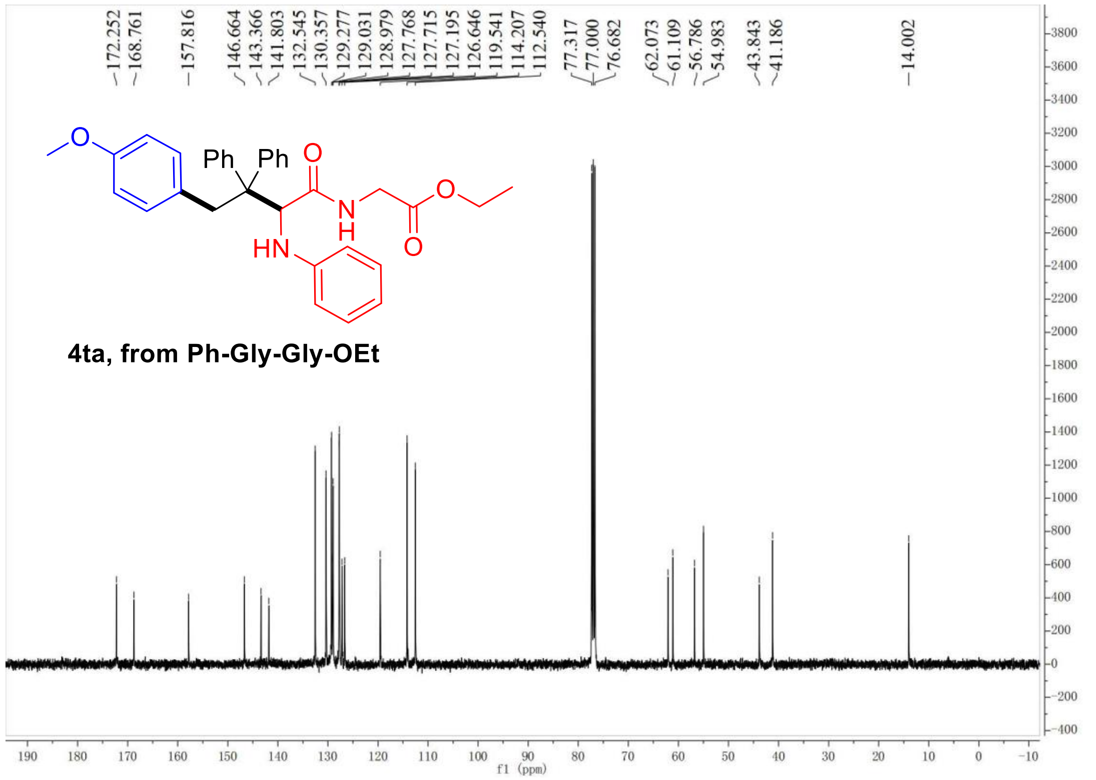
172.252
168.761
157.816
146.664
143.366
141.803
132.545
130.357
129.277
129.031
128.979
127.768
127.715
127.195
126.646
119.541
114.207
112.540
77.317
77.000
76.682
62.073
61.109
56.786
54.983
43.843
41.186
14.002
Ph
Ph
O
HN
N
H
4ta, from Ph-Gly-Gly-OEt
f1 (ppm)

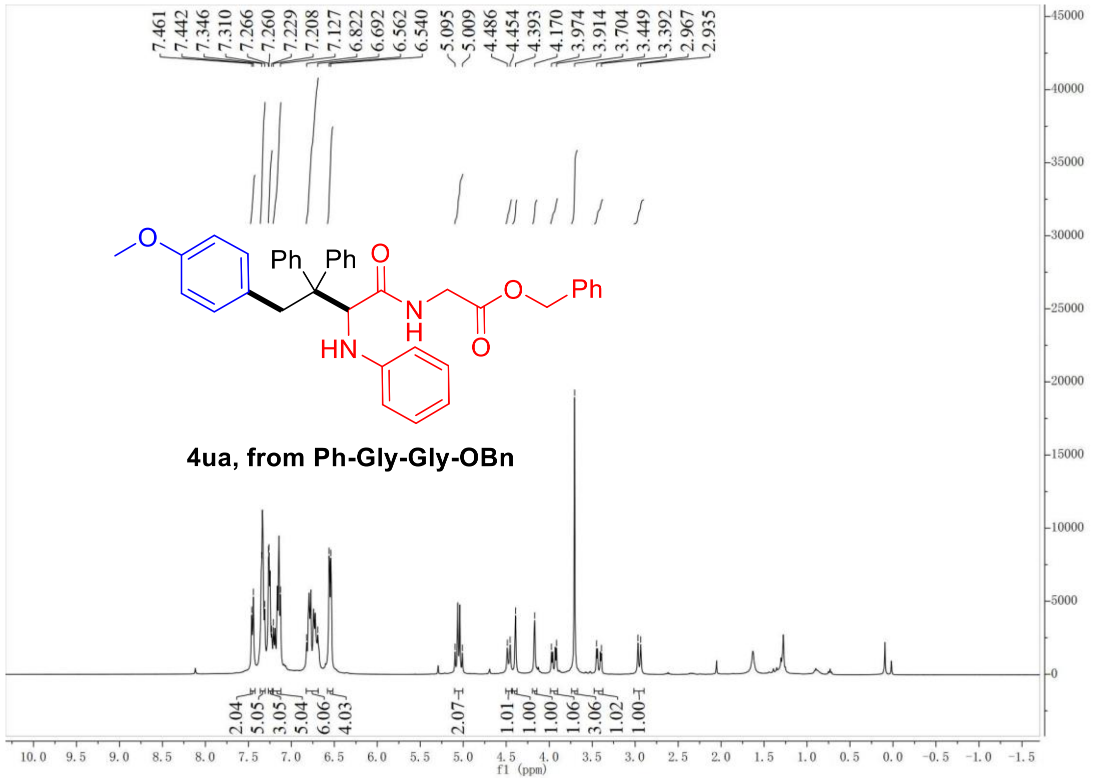
4ua, from Ph-Gly-Gly-OBn
f1 (ppm)

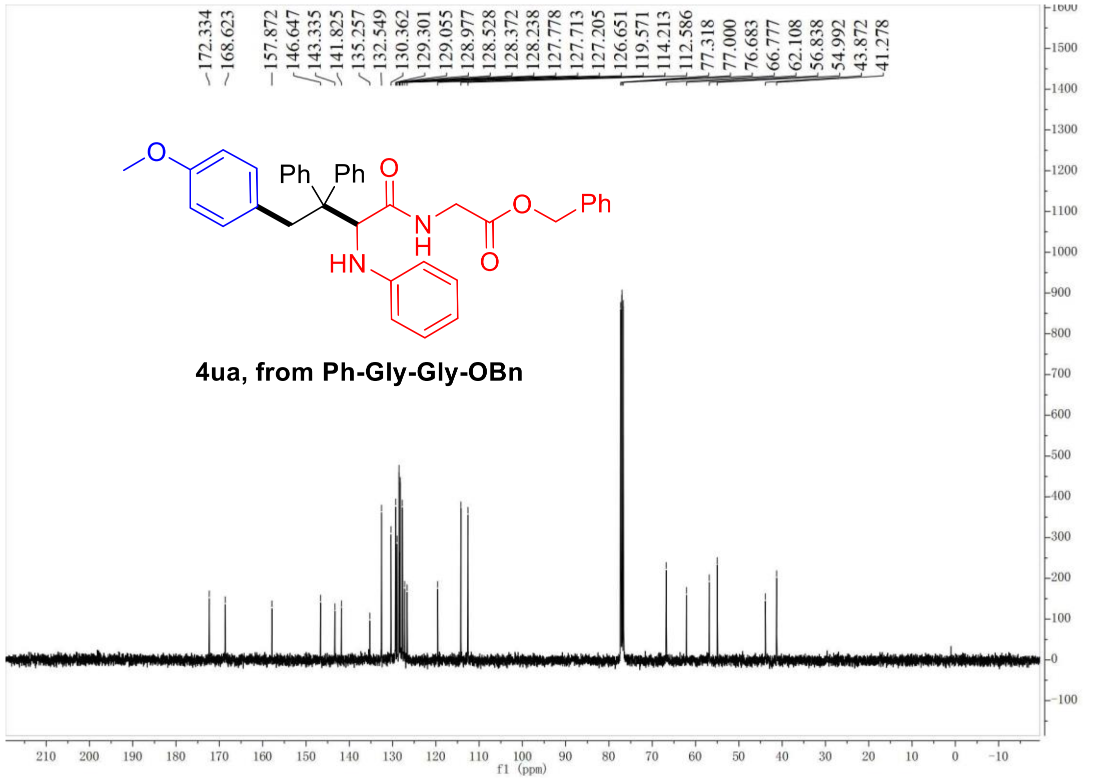
4ua, from Ph-Gly-Gly-OBn
f1 (ppm)

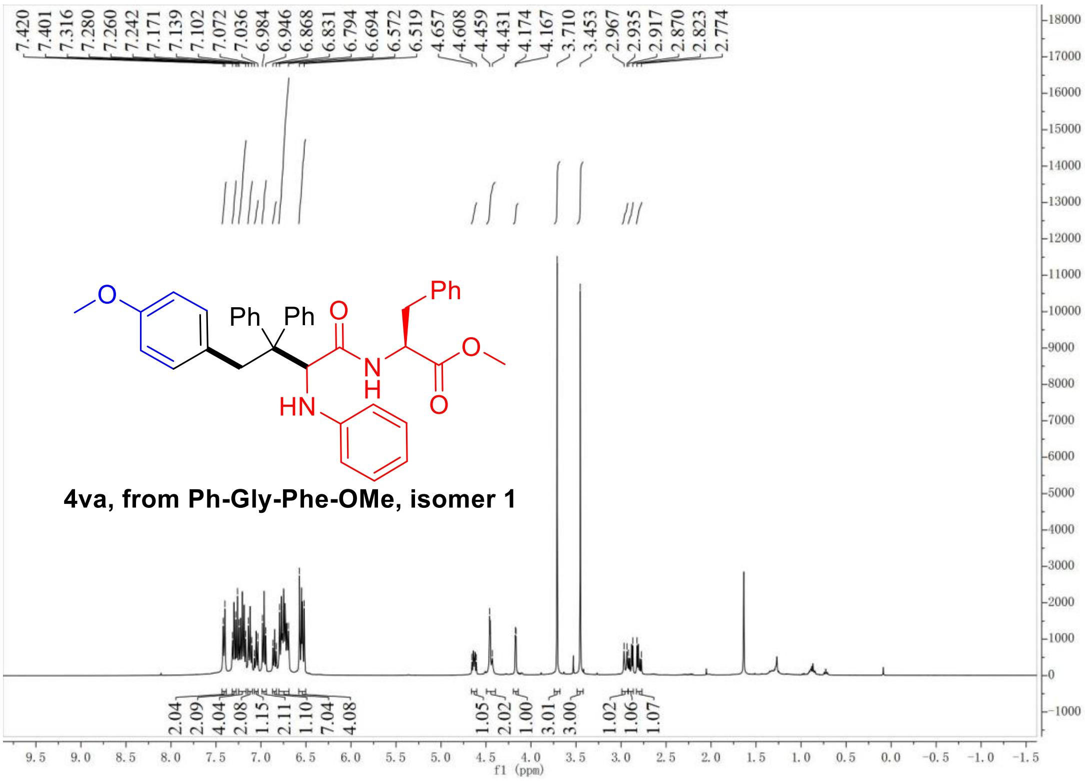
7.420
7.401
7.316
7.280
7.260
7.242
7.171
7.139
7.102
7.072
7.036
6.984
6.946
6.868
6.831
6.794
6.694
6.572
6.519
4.657
4.608
4.459
4.431
4.174
4.167
3.710
3.453
2.967
2.935
2.917
2.870
2.823
2.774
4va, from Ph-Gly-Phe-OMe, isomer 1
2.04
2.09
4.04
2.08
1.15
2.11
1.10
7.04
4.08
1.05
2.02
1.00
3.01
3.00
1.02
1.06
1.07
f1 (ppm)

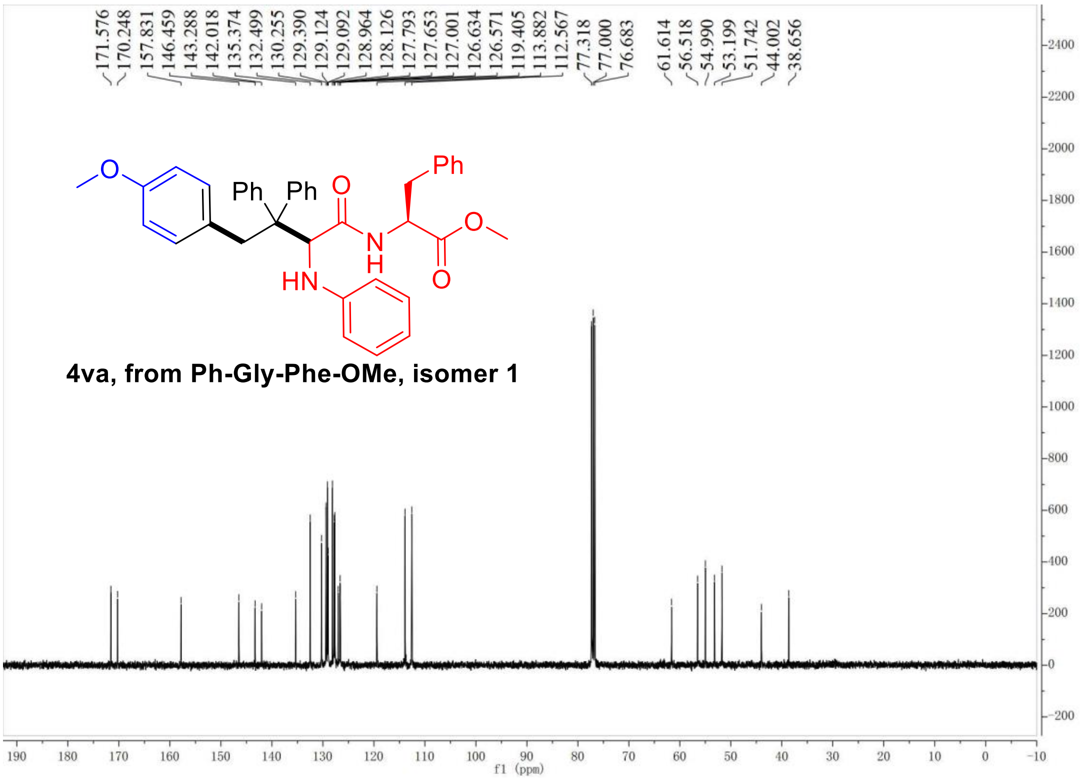
171.576
170.248
157.831
146.459
143.288
142.018
135.374
132.499
130.255
129.390
129.124
129.092
128.964
128.126
127.793
127.653
127.001
126.634
126.571
119.405
113.882
112.567
77.318
77.000
76.683
61.614
56.518
54.990
53.199
51.742
44.002
38.656
4va, from Ph-Gly-Phe-OMe, isomer 1
f1 (ppm)

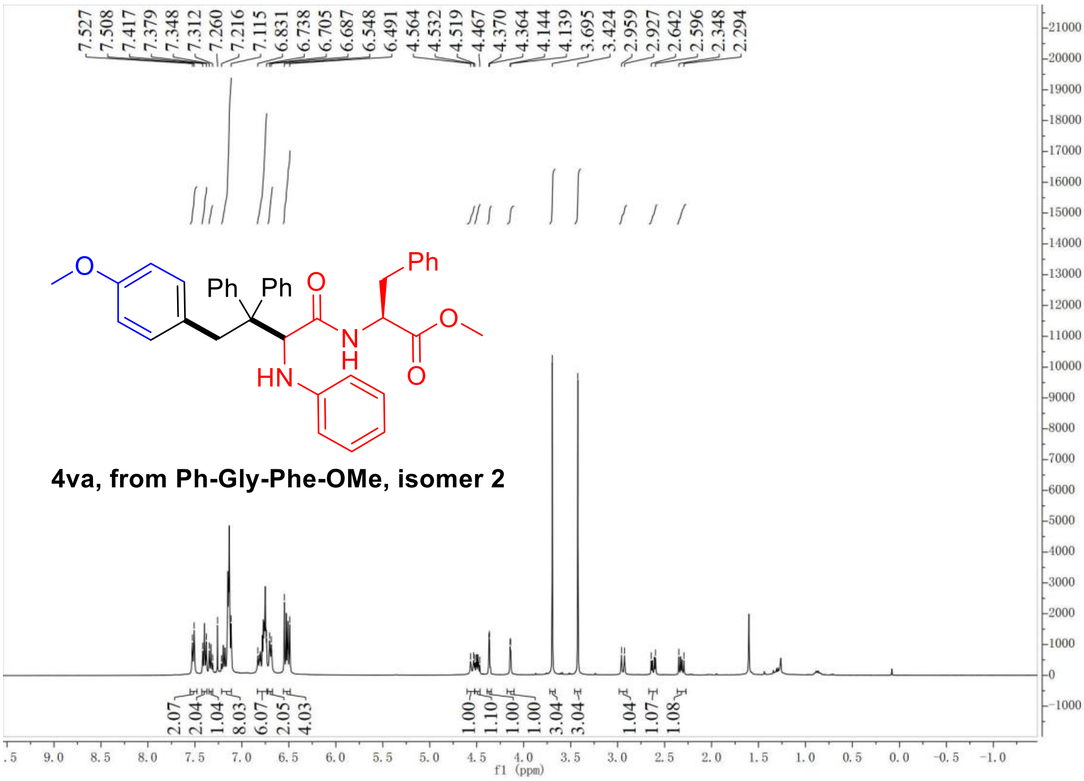

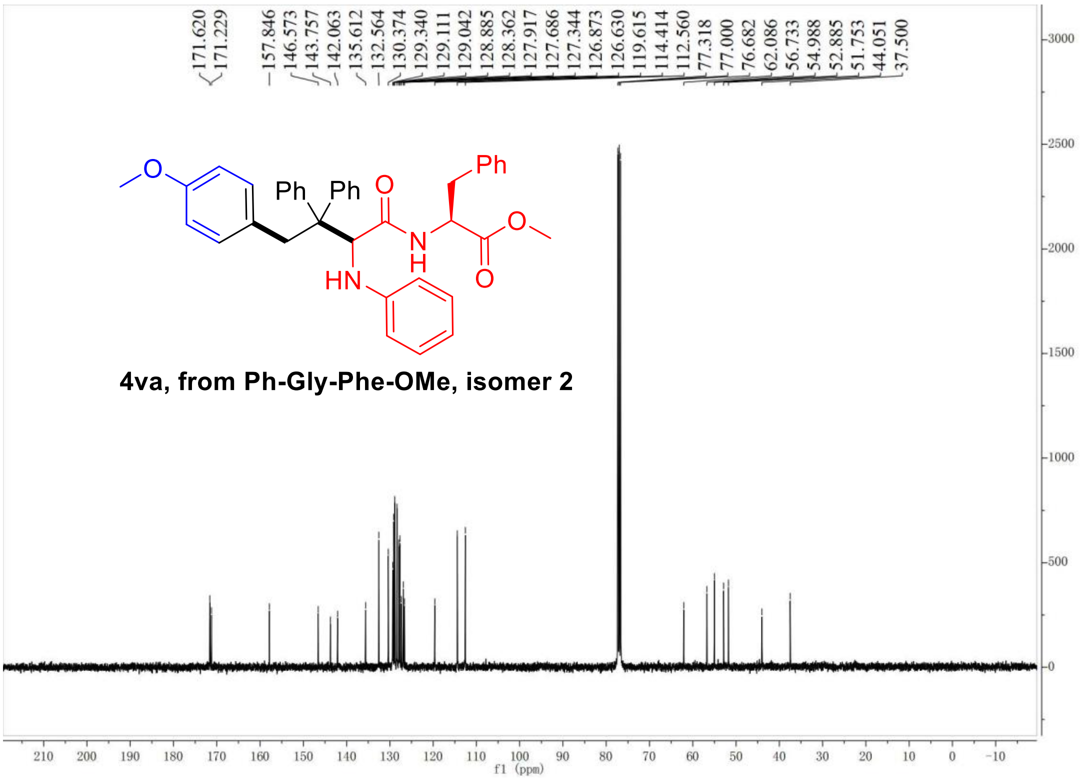

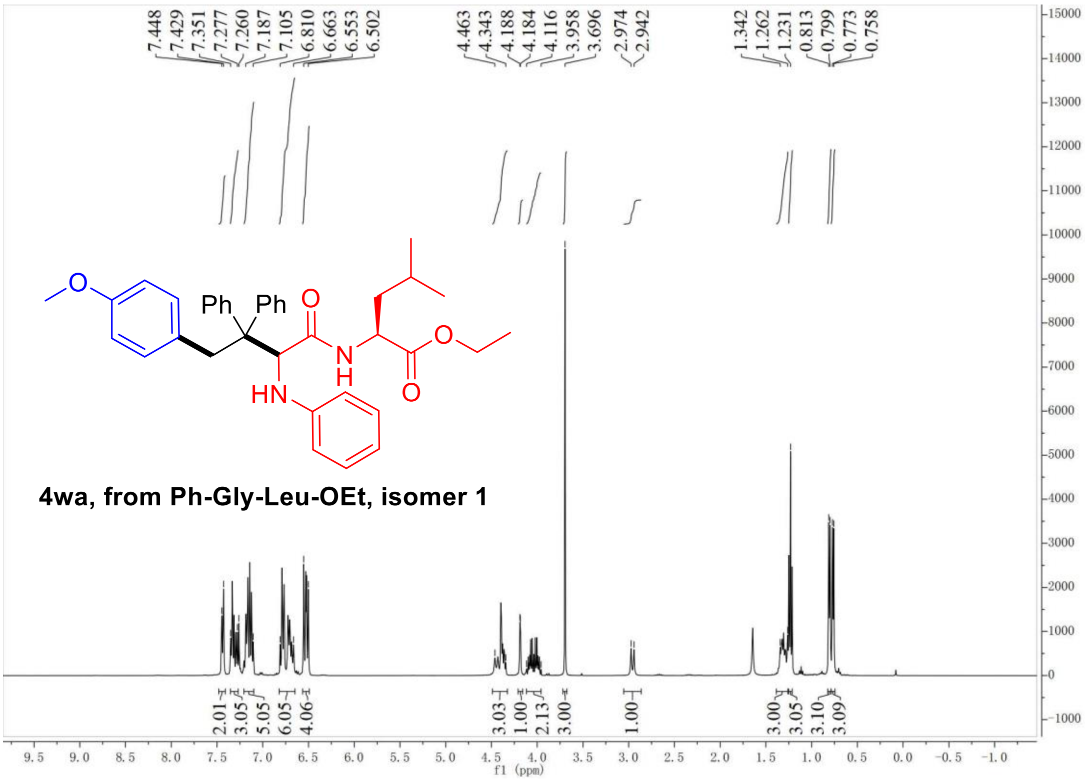
7.448
7.429
7.351
7.277
7.260
7.187
7.105
6.810
6.663
6.553
6.502
4.463
4.343
4.188
4.184
4.116
3.958
3.696
2.974
2.942
1.342
1.262
1.231
0.813
0.799
0.773
0.758
Ph
Ph
O
N
H
HN
4wa, from Ph-Gly-Leu-OEt, isomer 1
2.01
3.05
5.05
6.05
4.06
3.03
1.00
2.13
3.00
1.00
3.00
3.05
3.10
3.09
f1 (ppm)

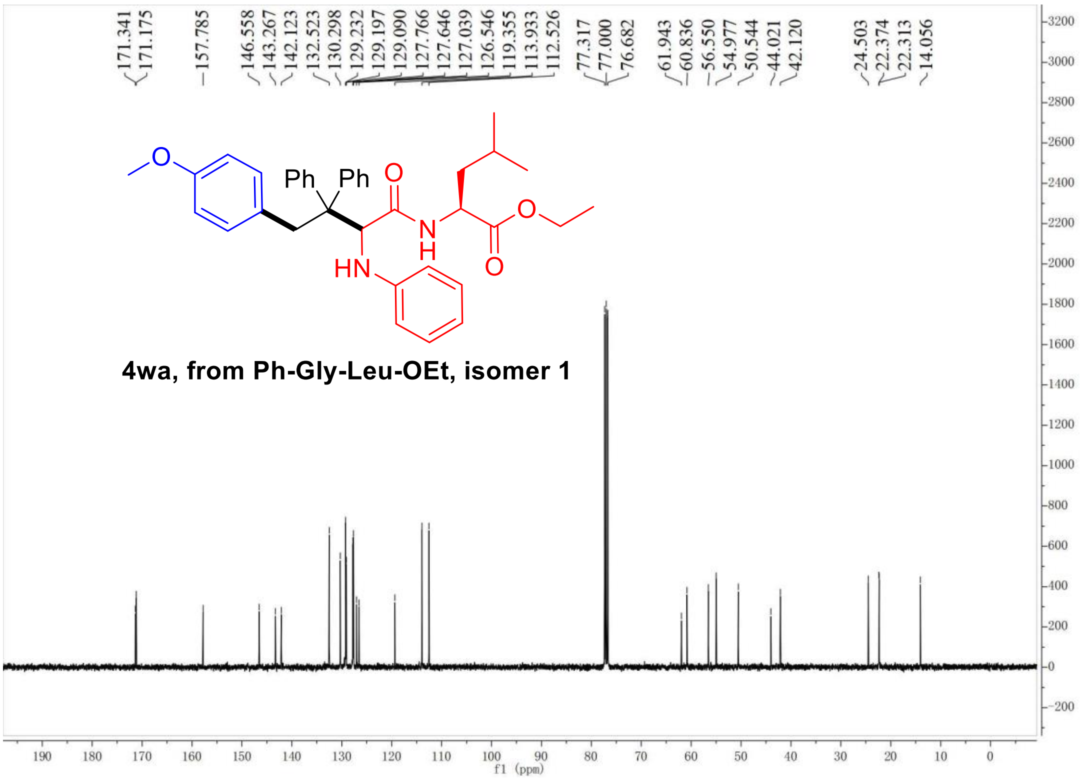
171.341
171.175
157.785
146.558
143.267
142.123
132.523
130.298
129.232
129.197
129.090
127.766
127.646
127.039
126.546
119.355
113.933
112.526
77.317
77.000
76.682
61.943
60.836
56.550
54.977
50.544
44.021
42.120
24.503
22.374
22.313
14.056
Ph
Ph
O
N
H
HN
4wa, from Ph-Gly-Leu-OEt, isomer 1
f1 (ppm)

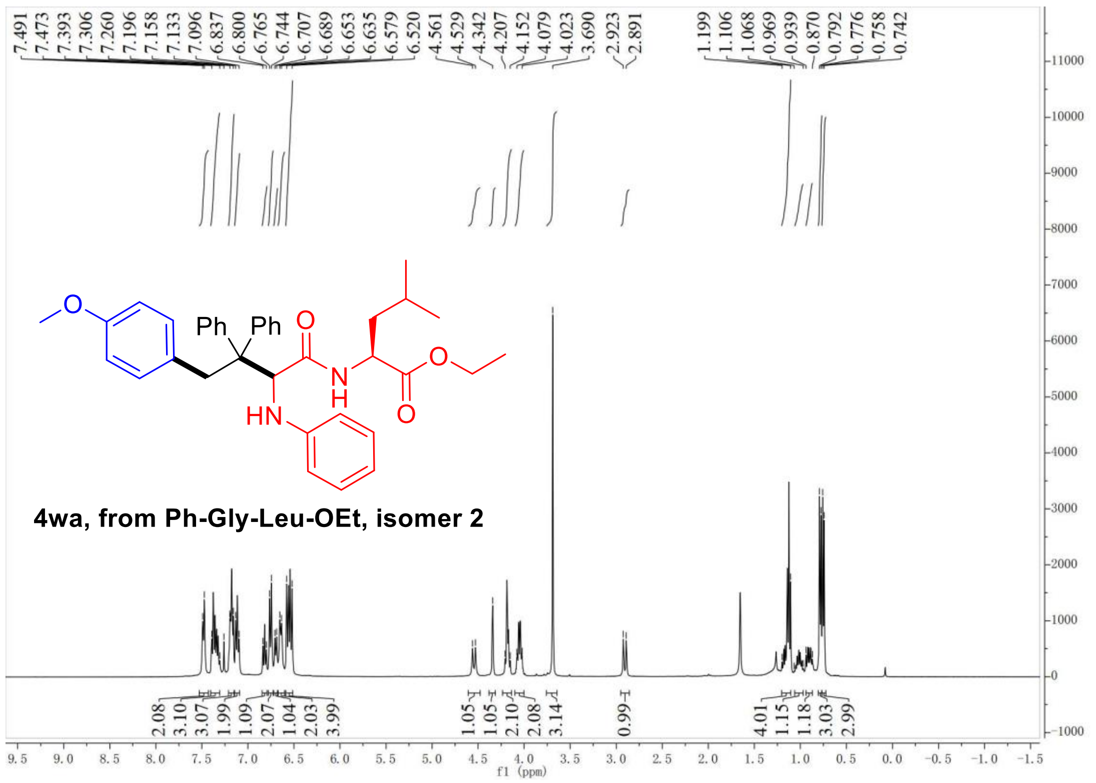

Ph
Ph
O
N
H
O
O
HN
4wa, from Ph-Gly-Leu-OEt, isomer 2
f1 (ppm)

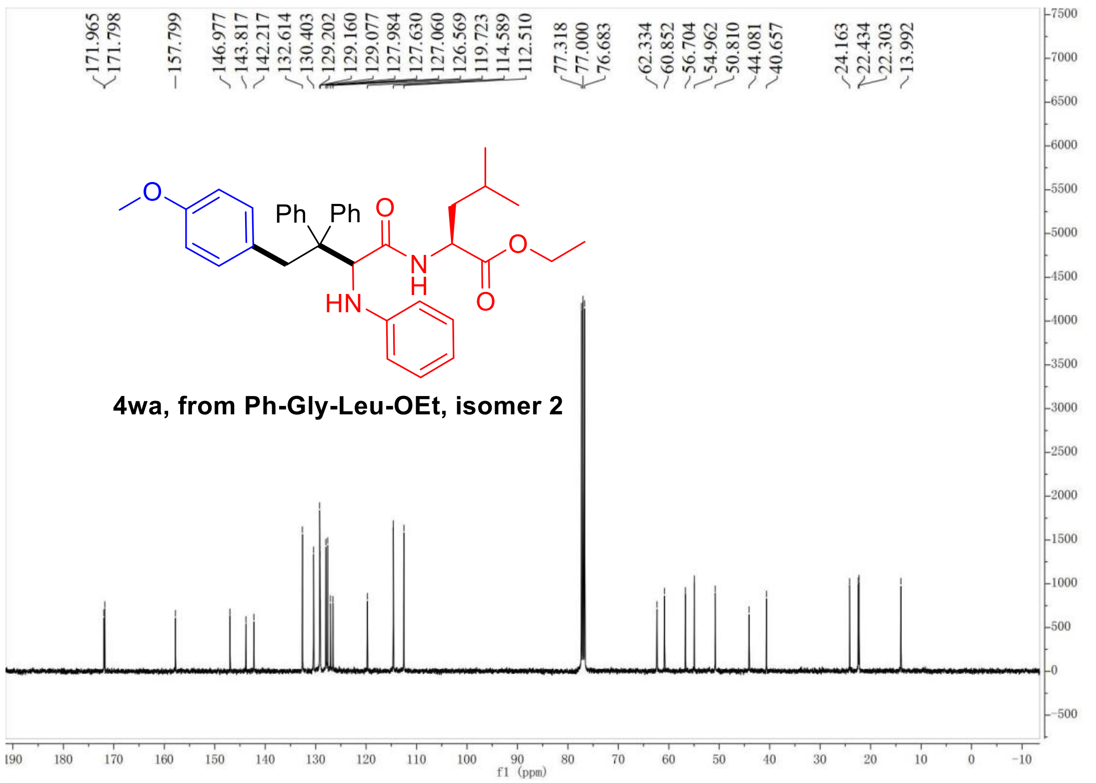

Ph
Ph
O
N
H
O
O
HN
4wa, from Ph-Gly-Leu-OEt, isomer 2
f1 (ppm)

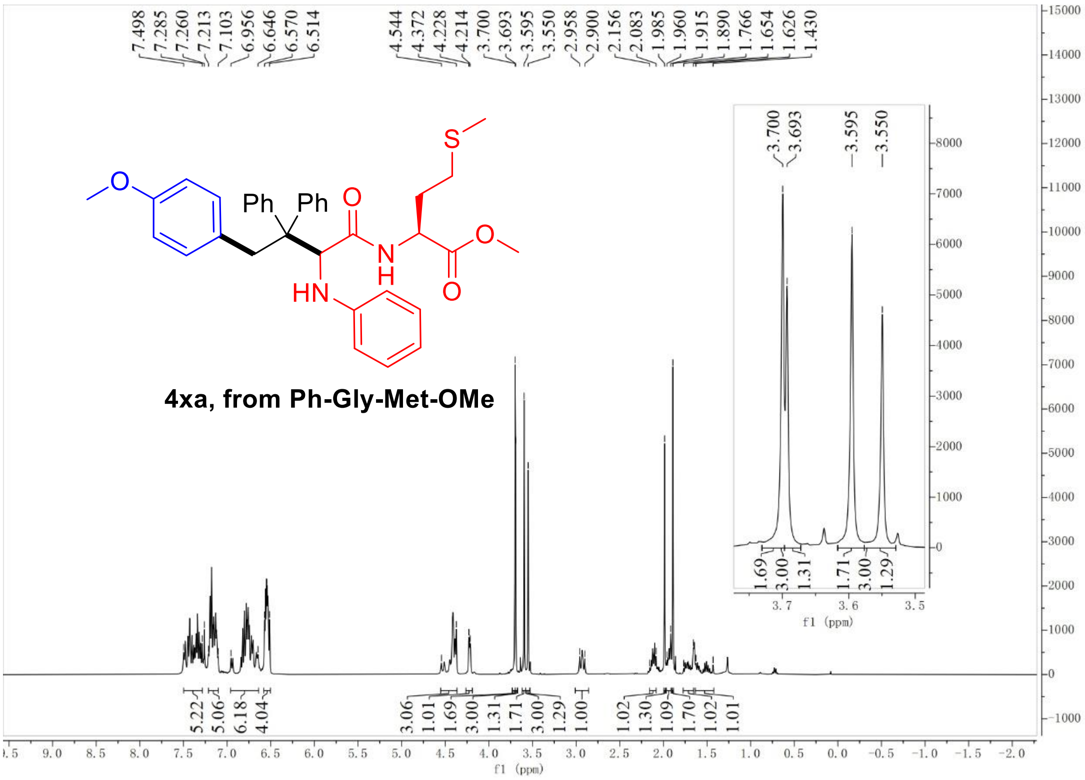

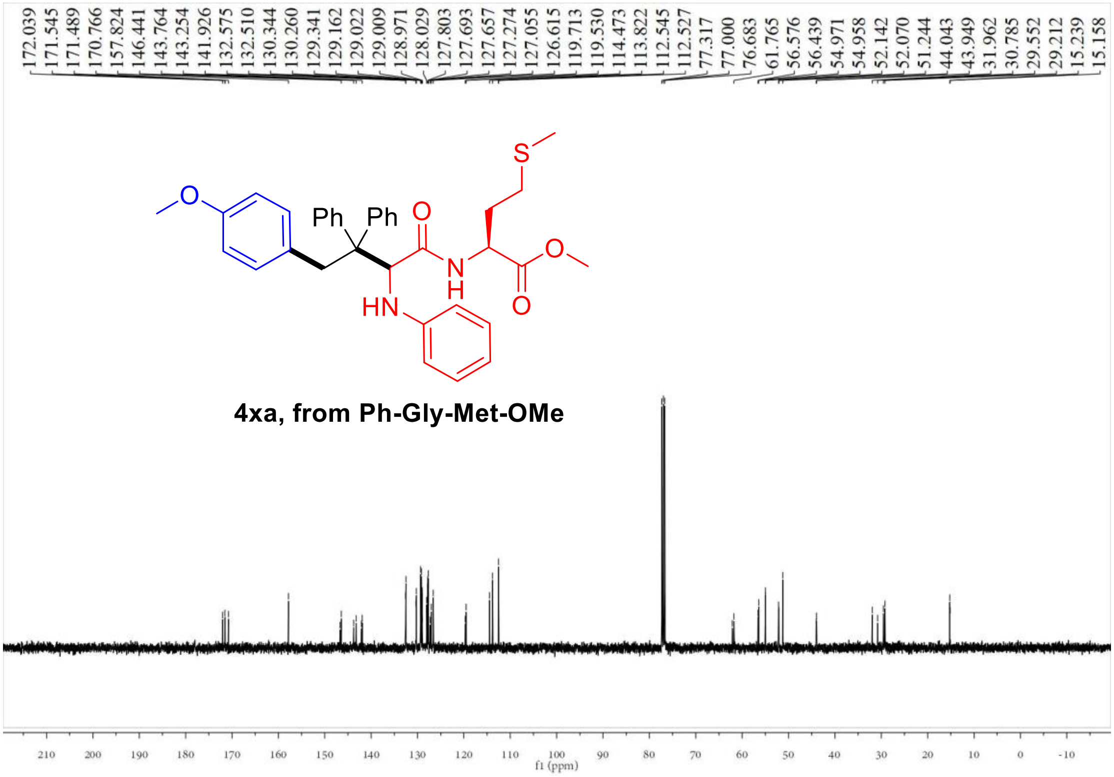

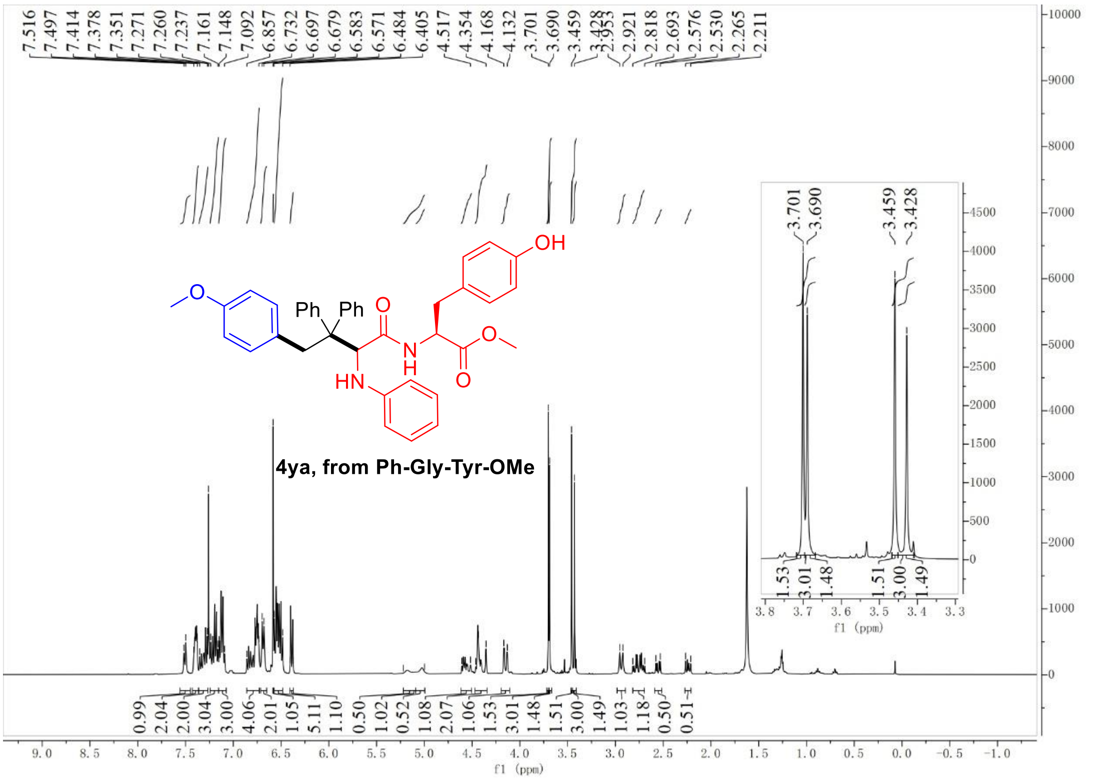
4ya, from Ph-Gly-Tyr-OMe

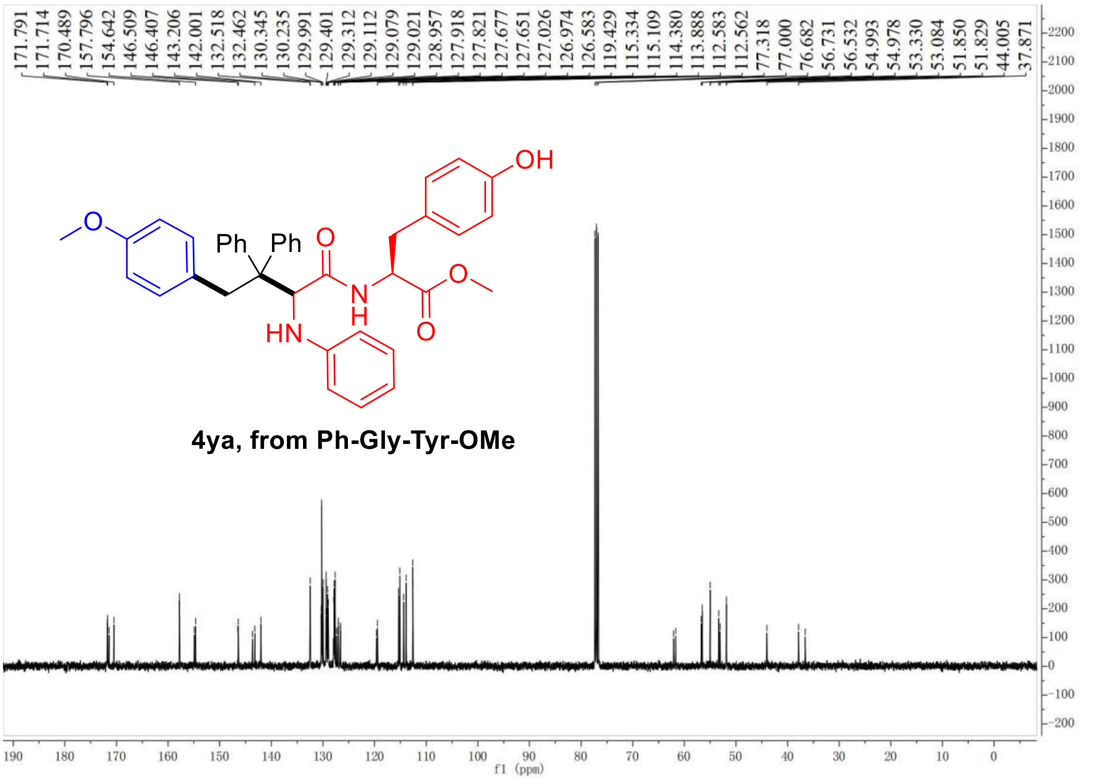
4ya, from Ph-Gly-Tyr-OMe

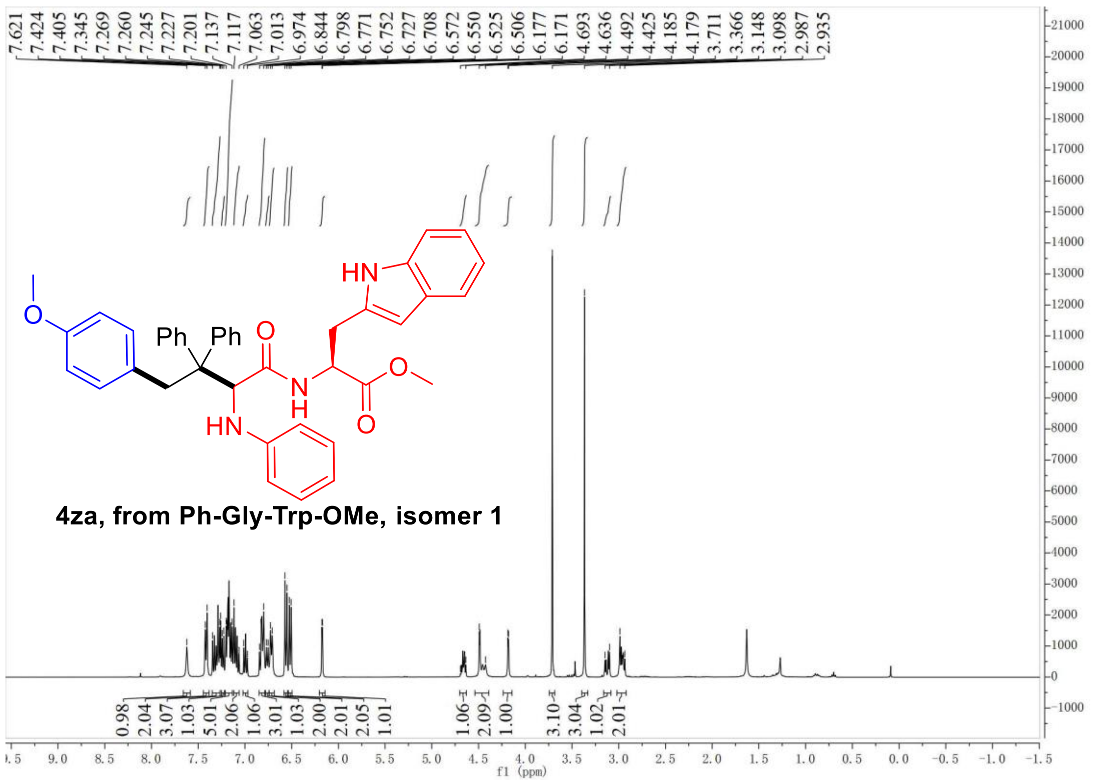
7.621
7.424
7.405
7.345
7.269
7.260
7.245
7.227
7.201
7.137
7.117
7.063
7.013
6.974
6.844
6.798
6.771
6.752
6.727
6.708
6.572
6.550
6.525
6.506
6.177
6.171
4.693
4.636
4.492
4.425
4.185
4.179
3.711
3.366
3.148
3.098
2.987
2.935
4za, from Ph-Gly-Trp-OMe, isomer 1
0.98
2.04
3.07
1.03
5.01
2.06
1.06
3.01
1.03
2.00
2.01
2.05
1.01
1.06
2.09
1.00
3.10
3.04
1.02
2.01
f1 (ppm)

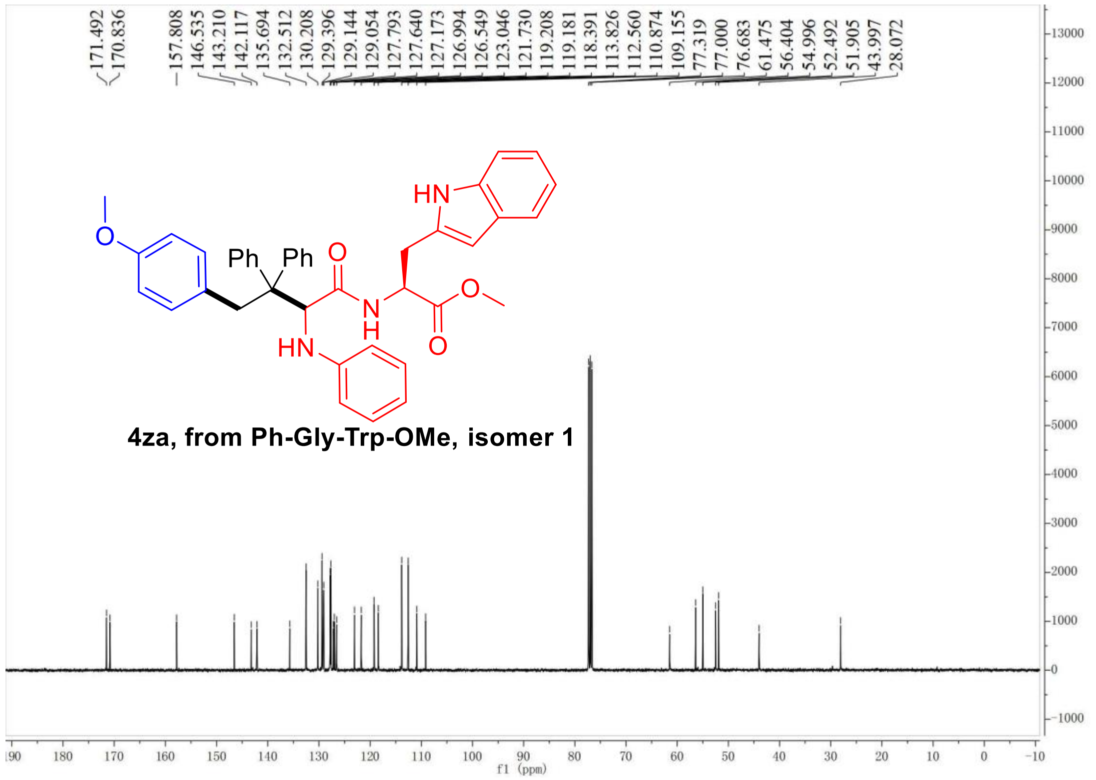
171.492
170.836
157.808
146.535
143.210
142.117
135.694
132.512
130.208
129.396
129.144
129.054
127.793
127.640
127.173
126.994
126.549
123.046
121.730
119.208
119.181
118.391
113.826
112.560
110.874
109.155
77.319
77.000
76.683
61.475
56.404
54.996
52.492
51.905
43.997
28.072
4za, from Ph-Gly-Trp-OMe, isomer 1
f1 (ppm)

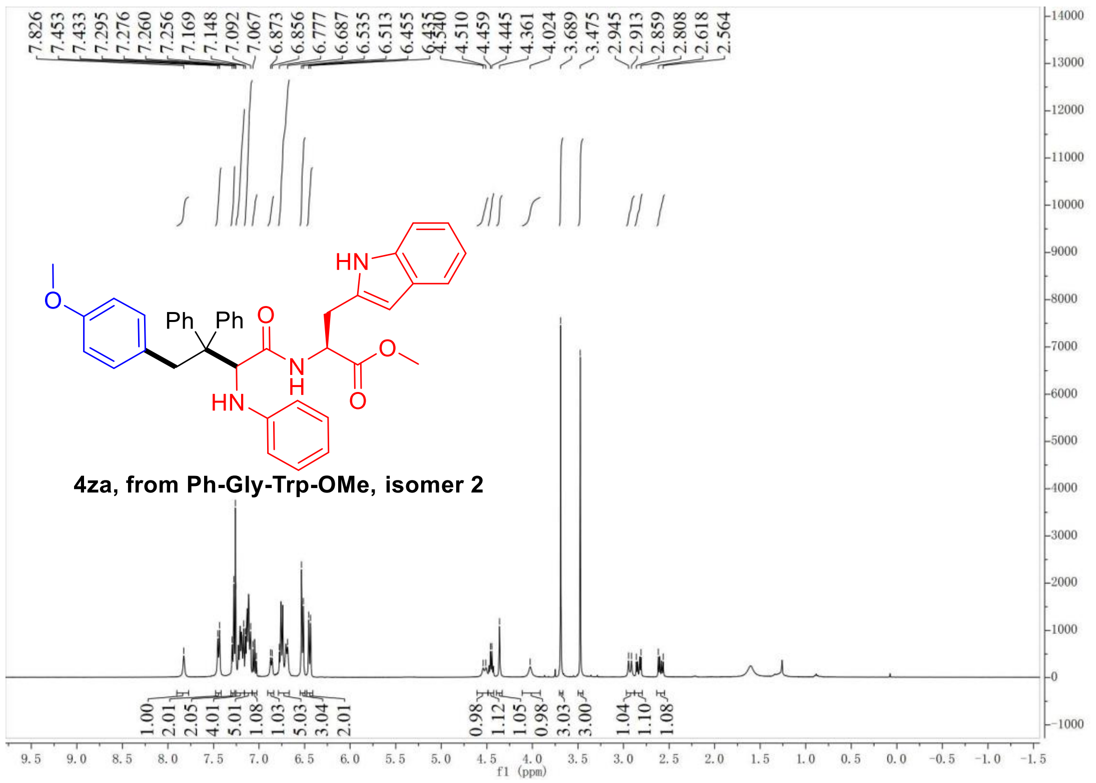

4za, from Ph-Gly-Trp-OMe, isomer 2

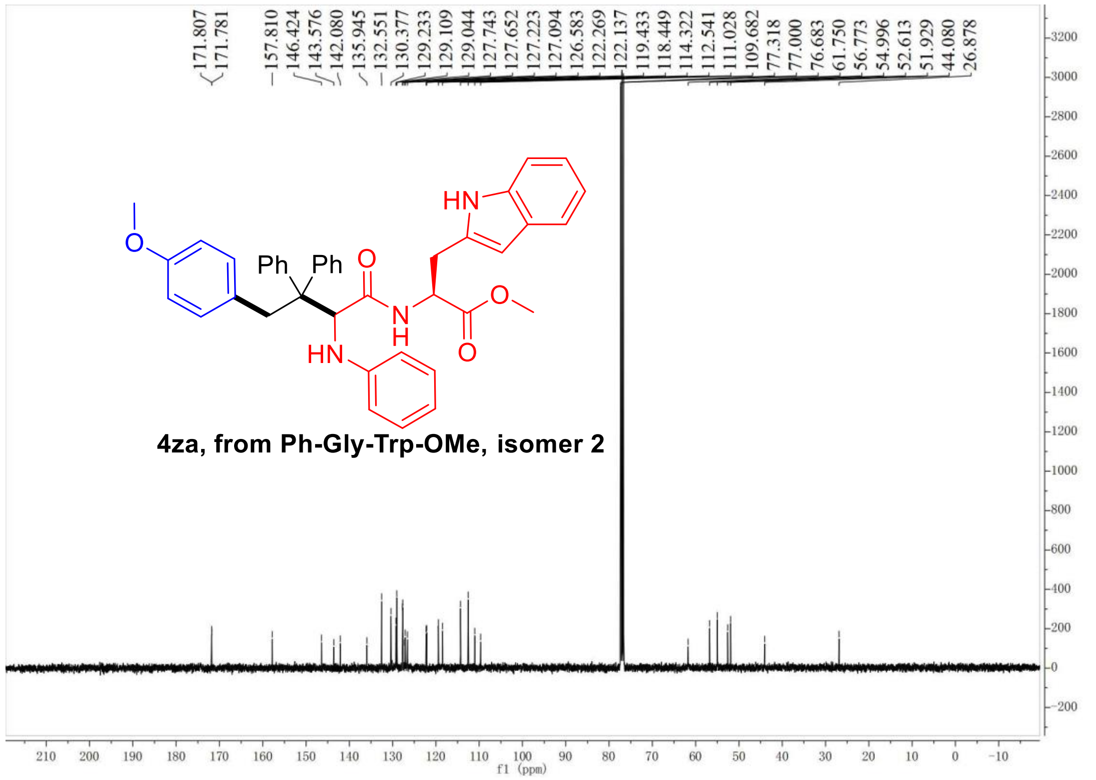

4za, from Ph-Gly-Trp-OMe, isomer 2

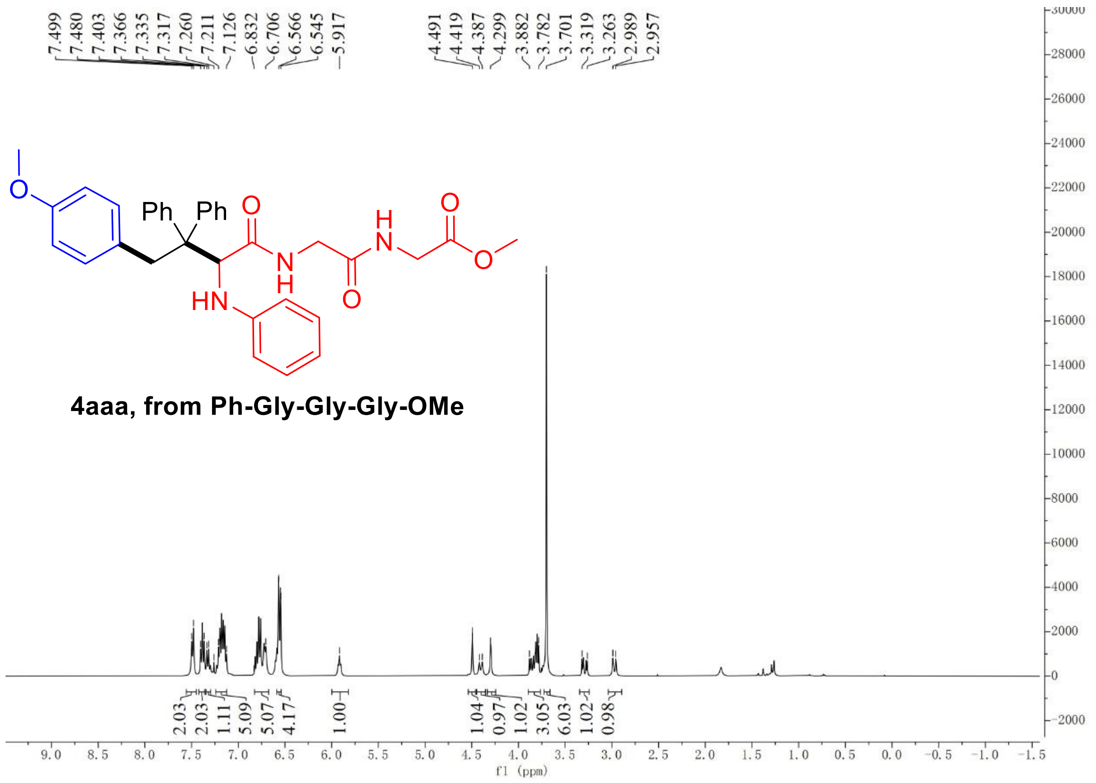

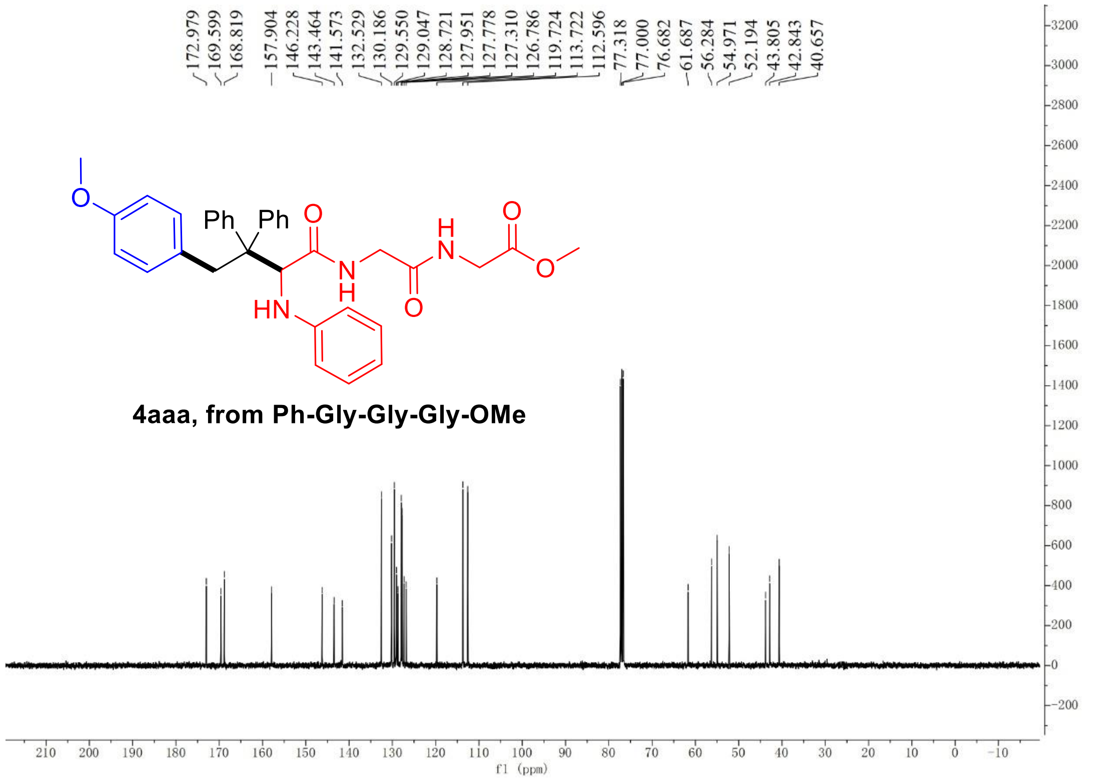

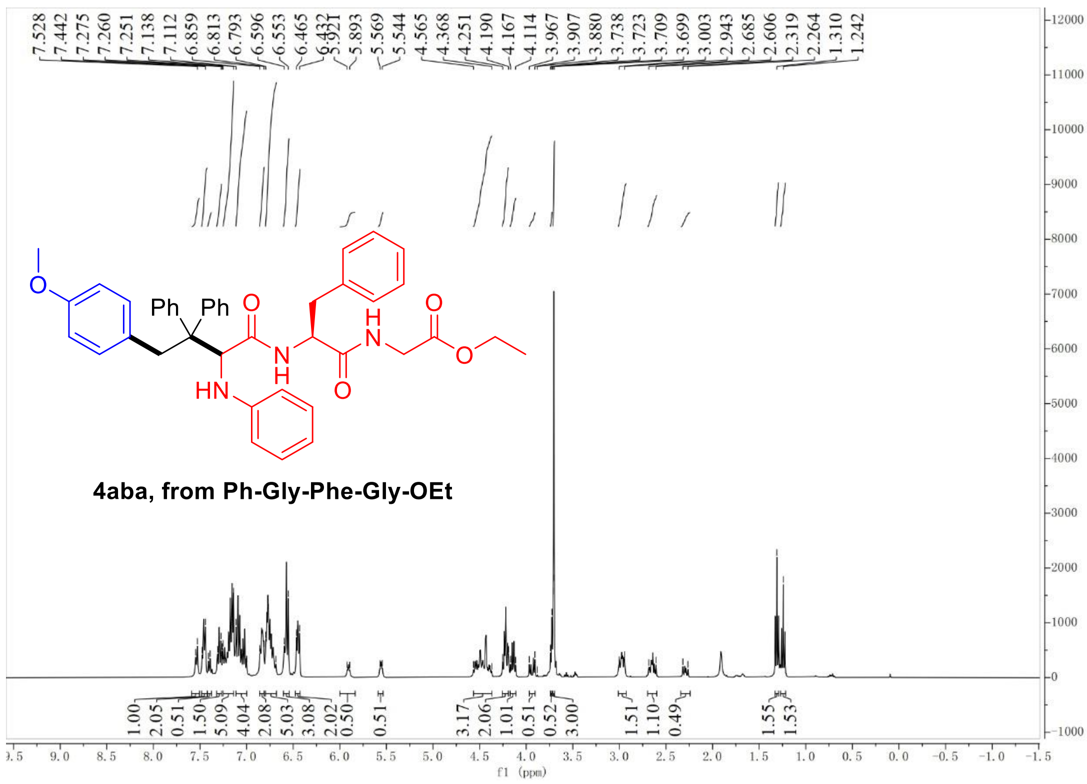
4aba, from Ph-Gly-Phe-Gly-OEt

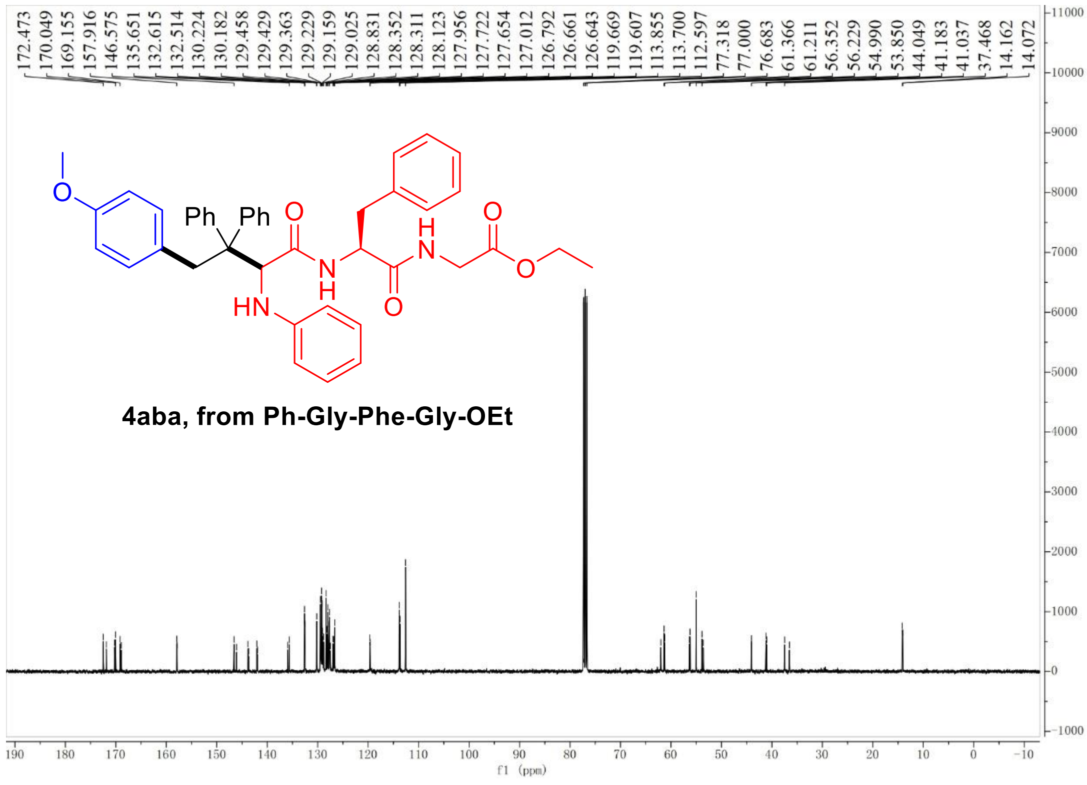
4aba, from Ph-Gly-Phe-Gly-OEt

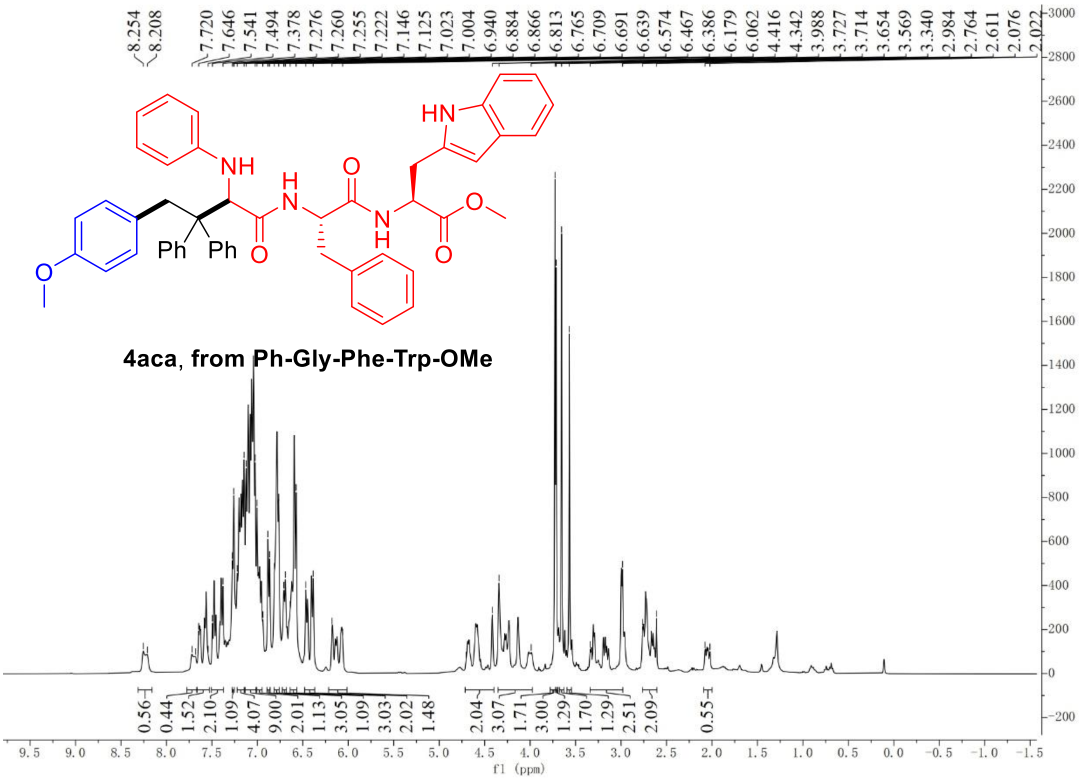
8.254
8.208
7.720
7.646
7.541
7.494
7.378
7.276
7.260
7.255
7.222
7.146
7.125
7.023
7.004
6.940
6.884
6.866
6.813
6.765
6.709
6.691
6.639
6.574
6.467
6.386
6.179
6.062
4.416
4.342
3.988
3.727
3.714
3.654
3.569
3.340
2.984
2.764
2.611
2.076
2.022
HN
NH
H
N
O
N
H
O
Ph
Ph
O
O
O
4aca, from Ph-Gly-Phe-Trp-OMe
f1 (ppm)

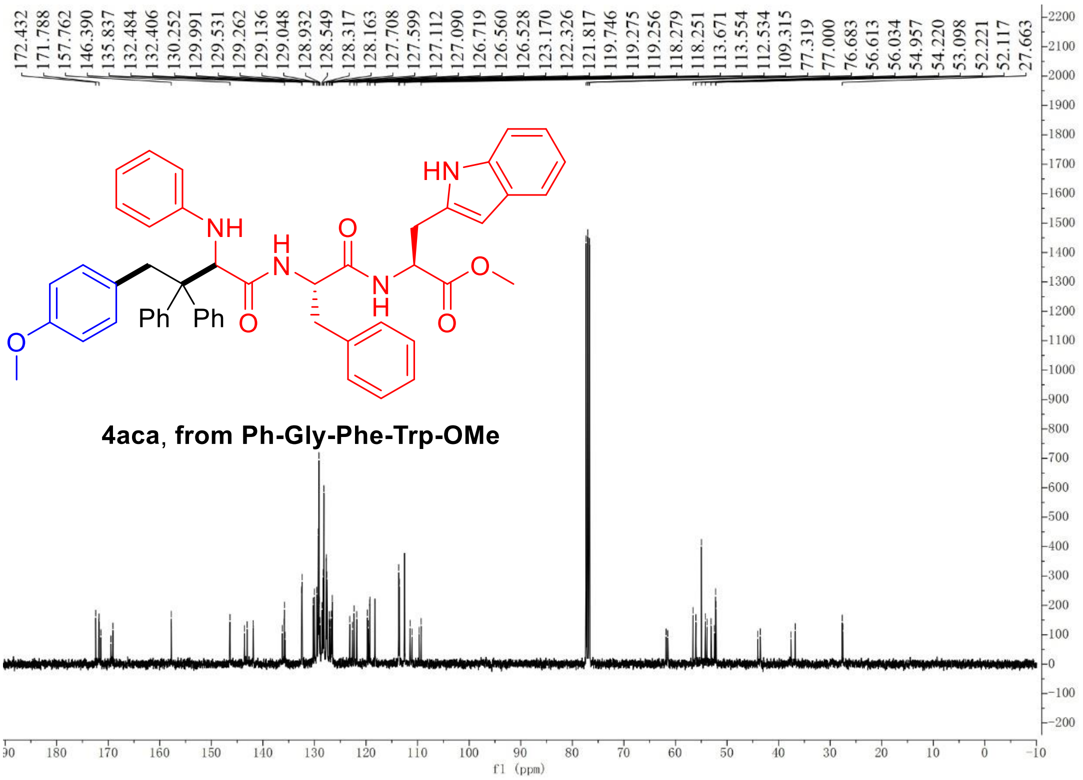
172.432
171.788
157.762
146.390
135.837
132.484
132.406
130.252
129.991
129.531
129.262
129.136
129.048
128.932
128.549
128.317
128.163
127.708
127.599
127.112
127.090
126.719
126.560
126.528
123.170
122.326
121.817
119.746
119.275
119.256
118.279
118.251
113.671
113.554
112.534
109.315
77.319
77.000
76.683
56.613
56.034
54.957
54.220
53.098
52.221
52.117
27.663
4aca, from Ph-Gly-Phe-Trp-OMe
f1 (ppm)

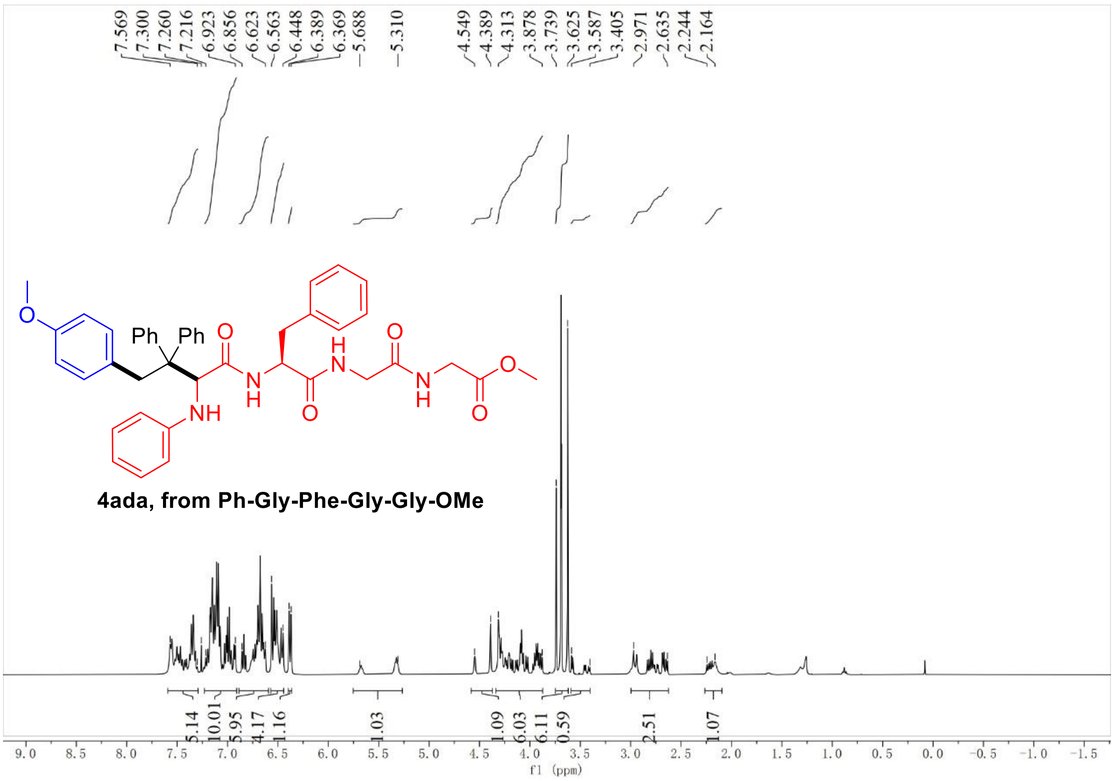
4ada, from Ph-Gly-Phe-Gly-Gly-OMe
Ph
Ph
NH
f1 (ppm)

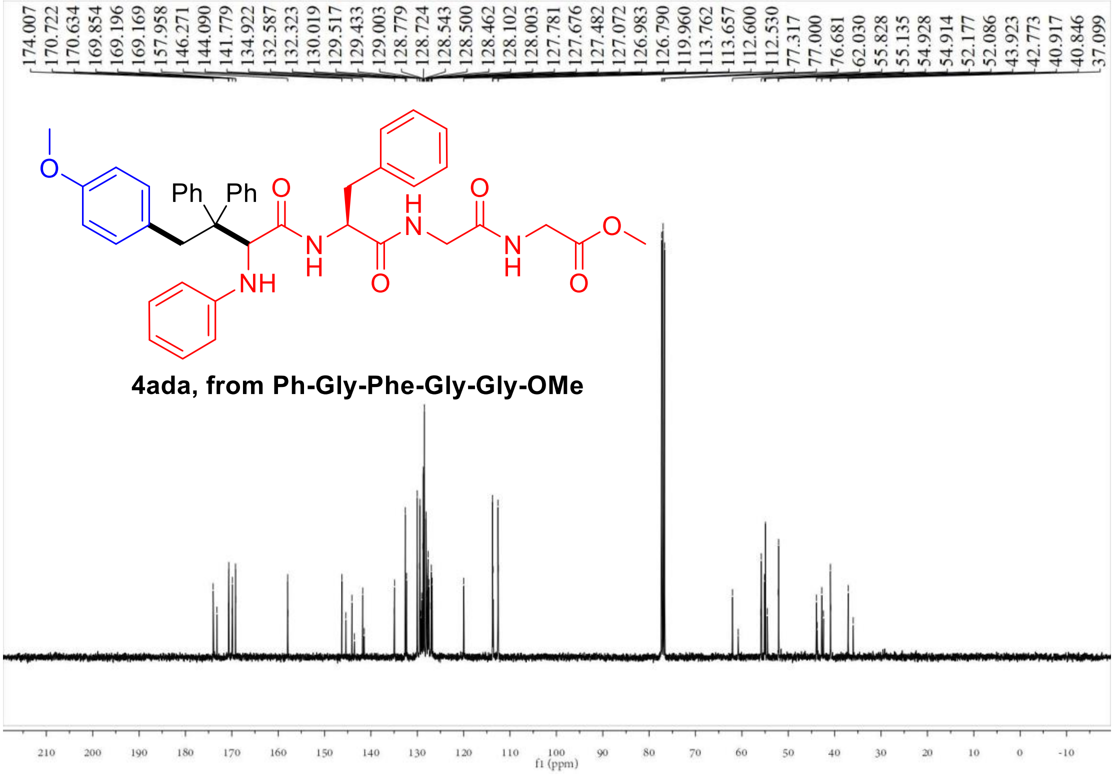
4ada, from Ph-Gly-Phe-Gly-Gly-OMe
Ph
Ph
NH
f1 (ppm)

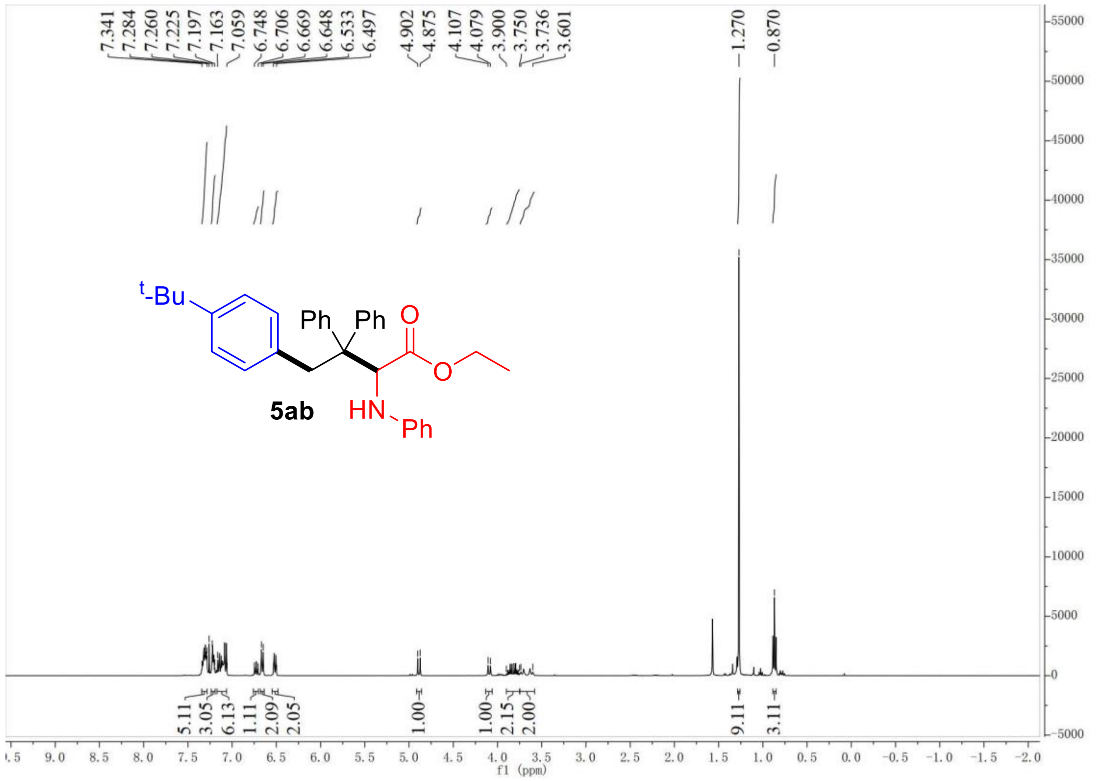

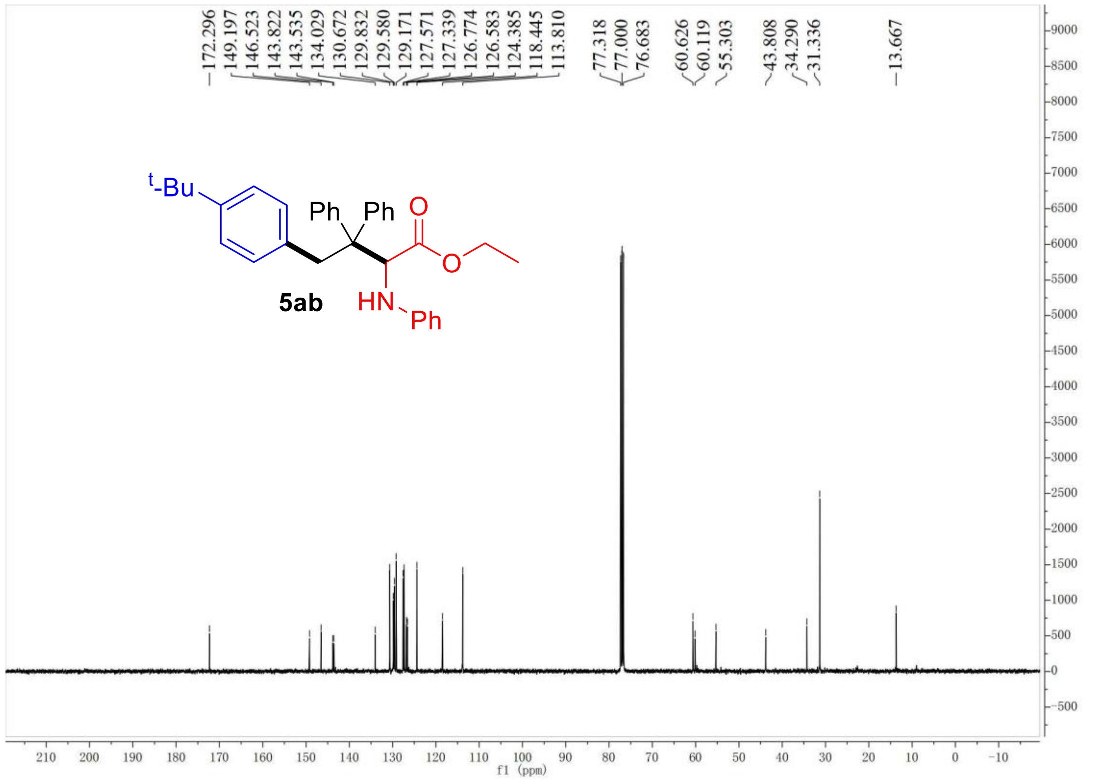

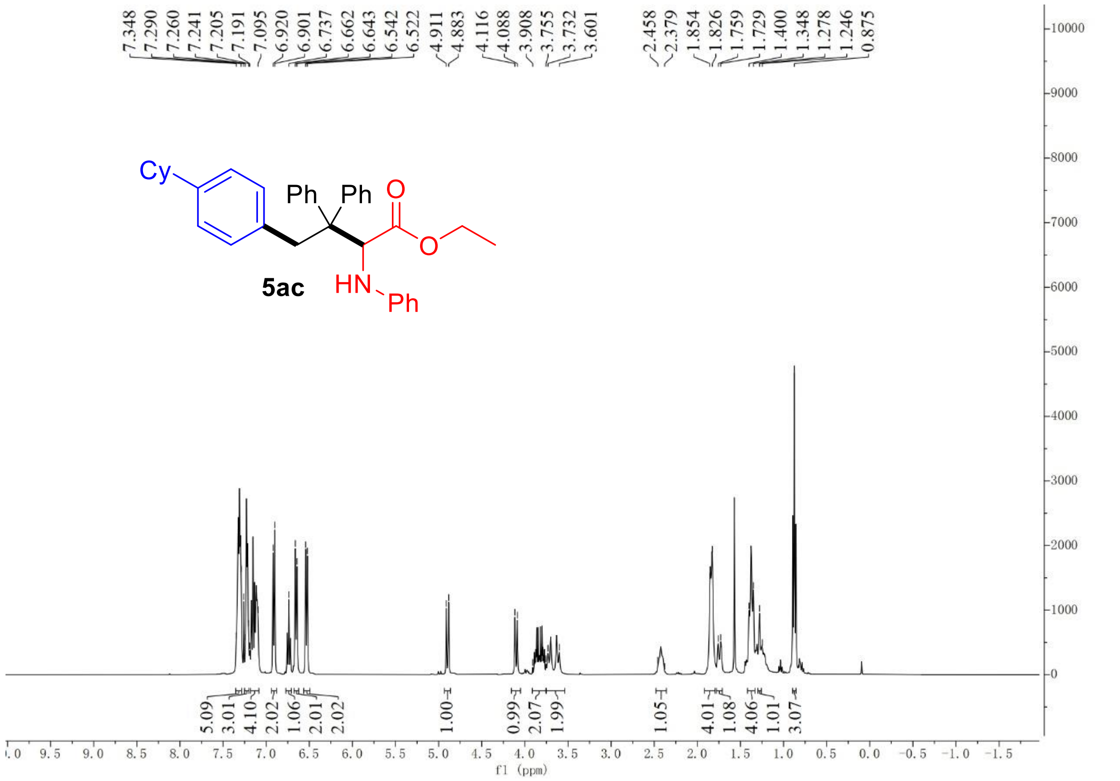

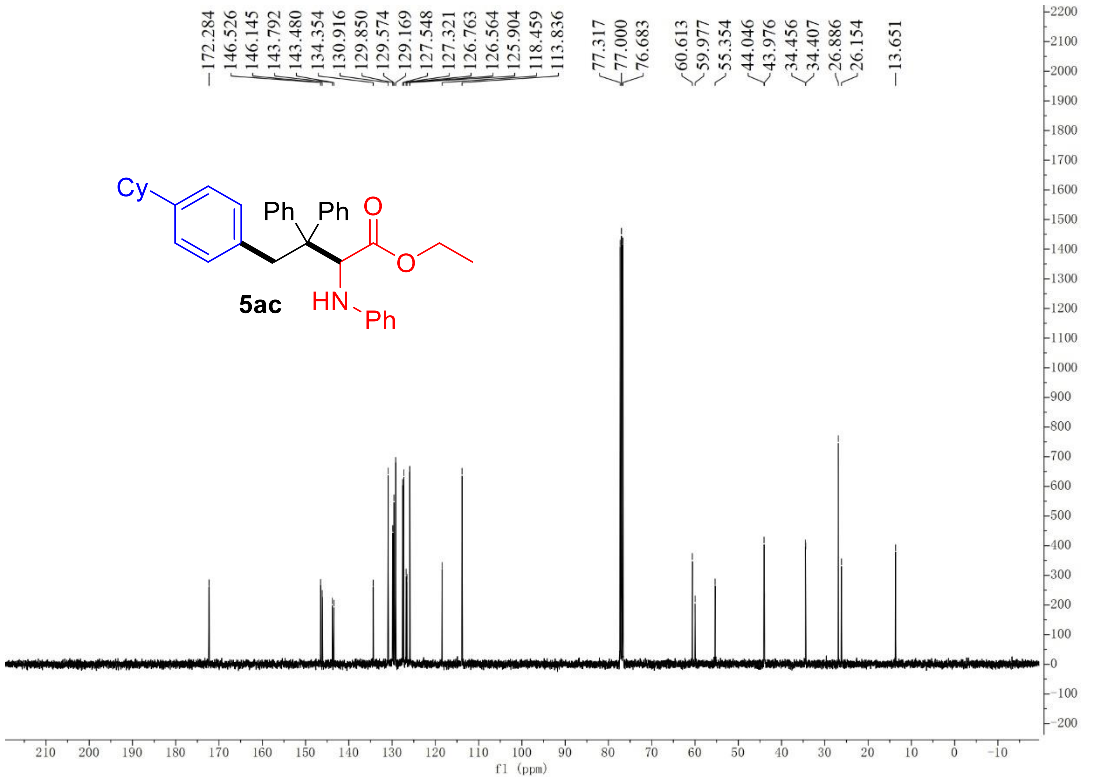

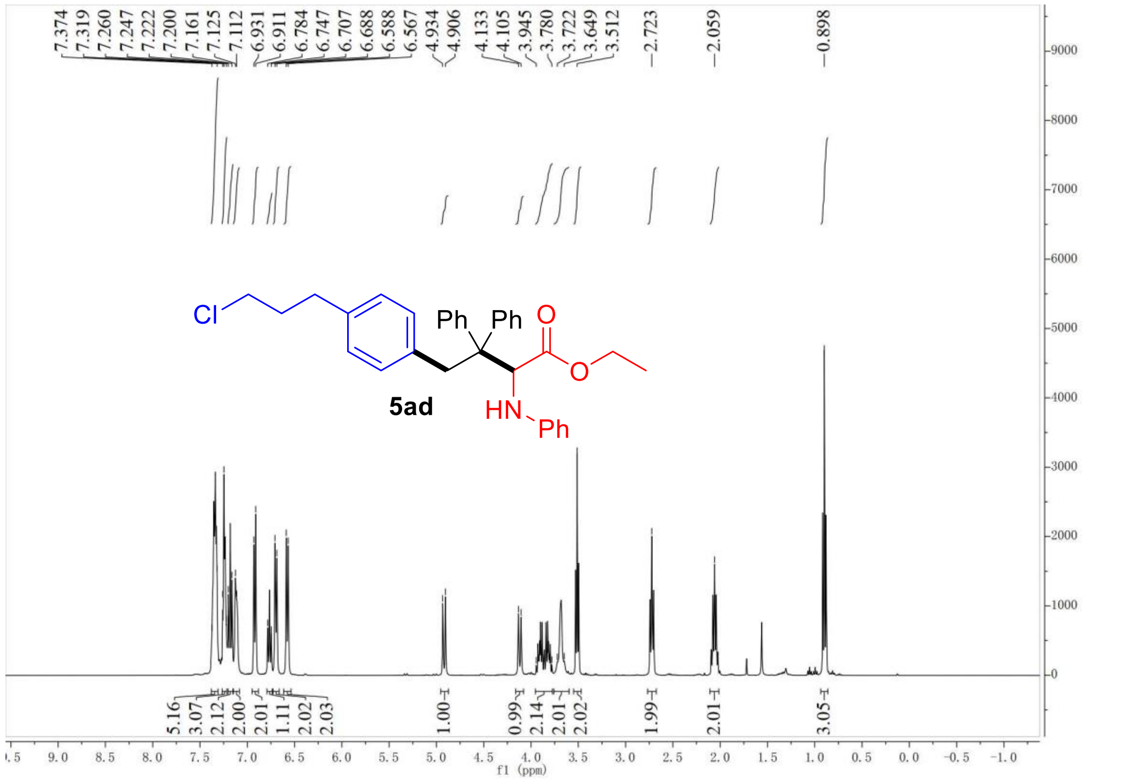

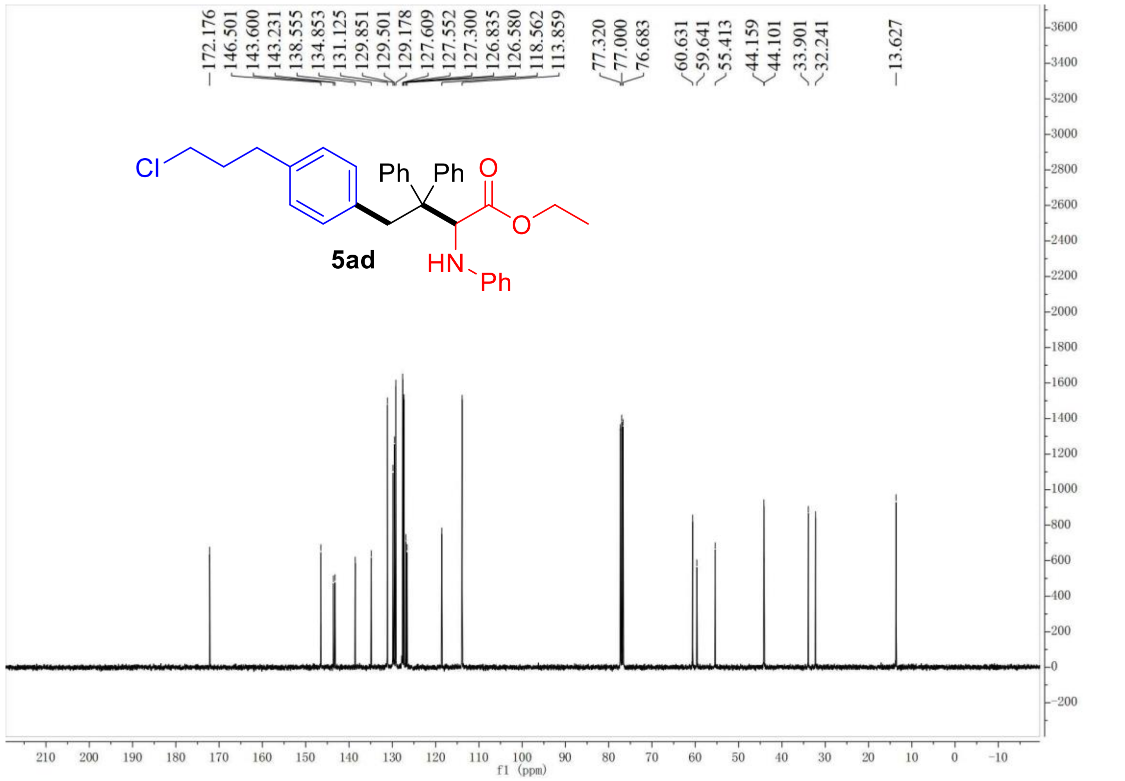

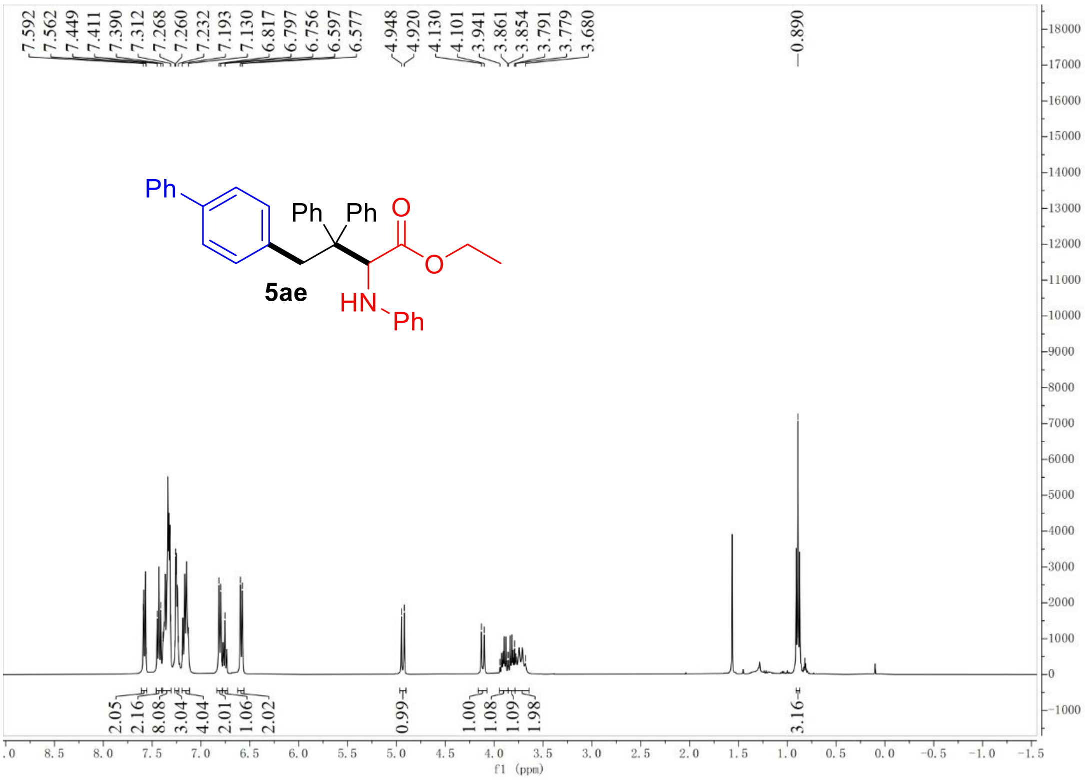

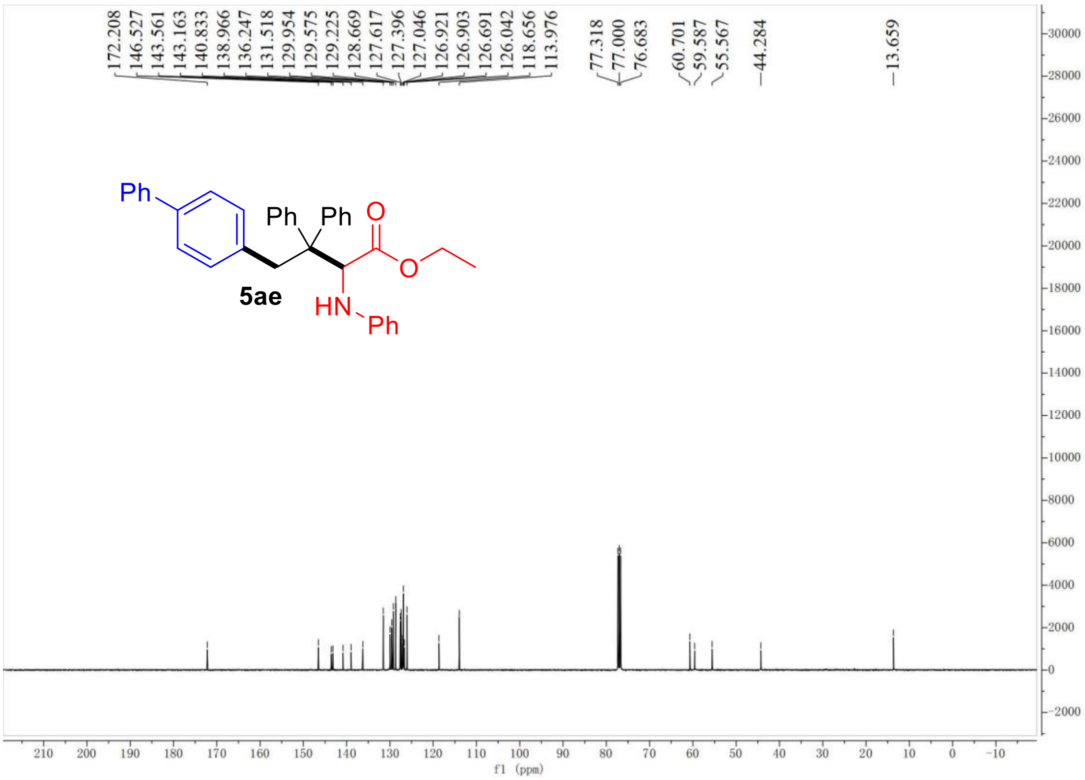

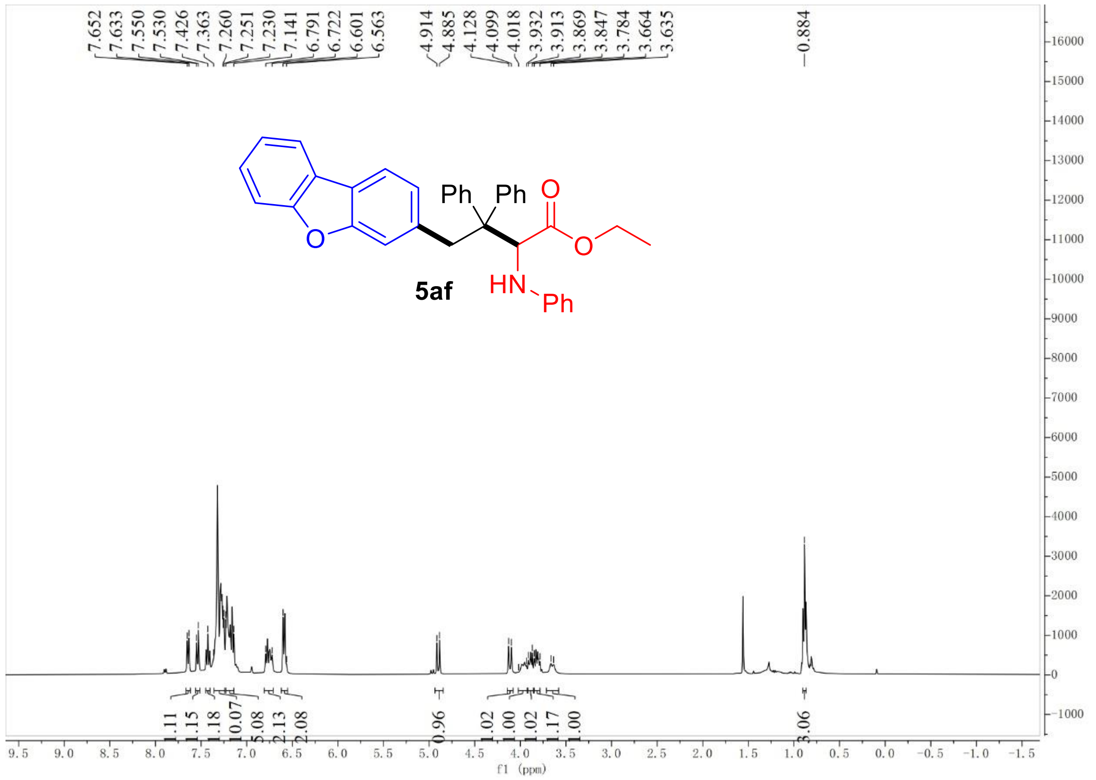
7.652
7.633
7.550
7.530
7.426
7.363
7.260
7.251
7.230
7.141
6.791
6.722
6.601
6.563
4.914
4.885
4.128
4.099
4.018
3.932
3.913
3.869
3.847
3.784
3.664
3.635
0.884
Ph
Ph
O
O
O
HN
Ph
5af
1.11
1.15
1.18
10.07
5.08
2.13
2.08
0.96
1.02
1.00
1.02
1.17
1.00
3.06
f1 (ppm)

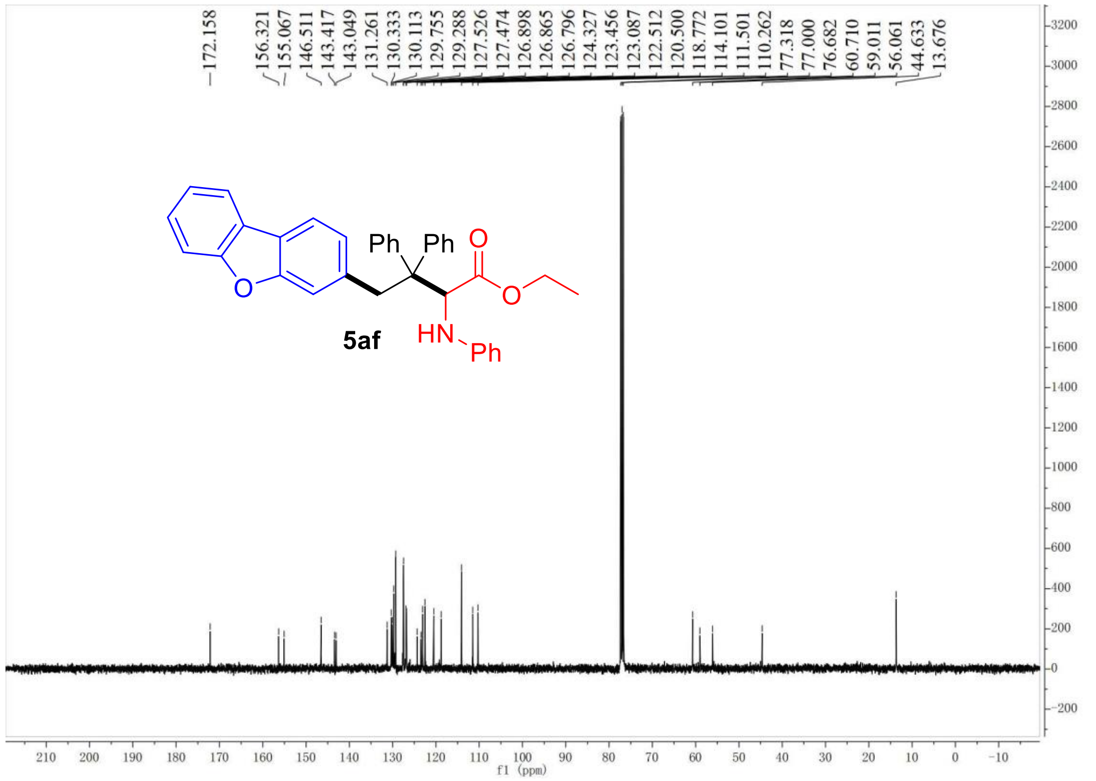
172.158
156.321
155.067
146.511
143.417
143.049
131.261
130.333
130.113
129.755
129.288
127.526
127.474
126.898
126.865
126.796
124.327
123.456
123.087
122.512
120.500
118.772
114.101
111.501
110.262
77.318
77.000
76.682
60.710
59.011
56.061
44.633
13.676
Ph
Ph
O
O
O
HN
Ph
5af
f1 (ppm)

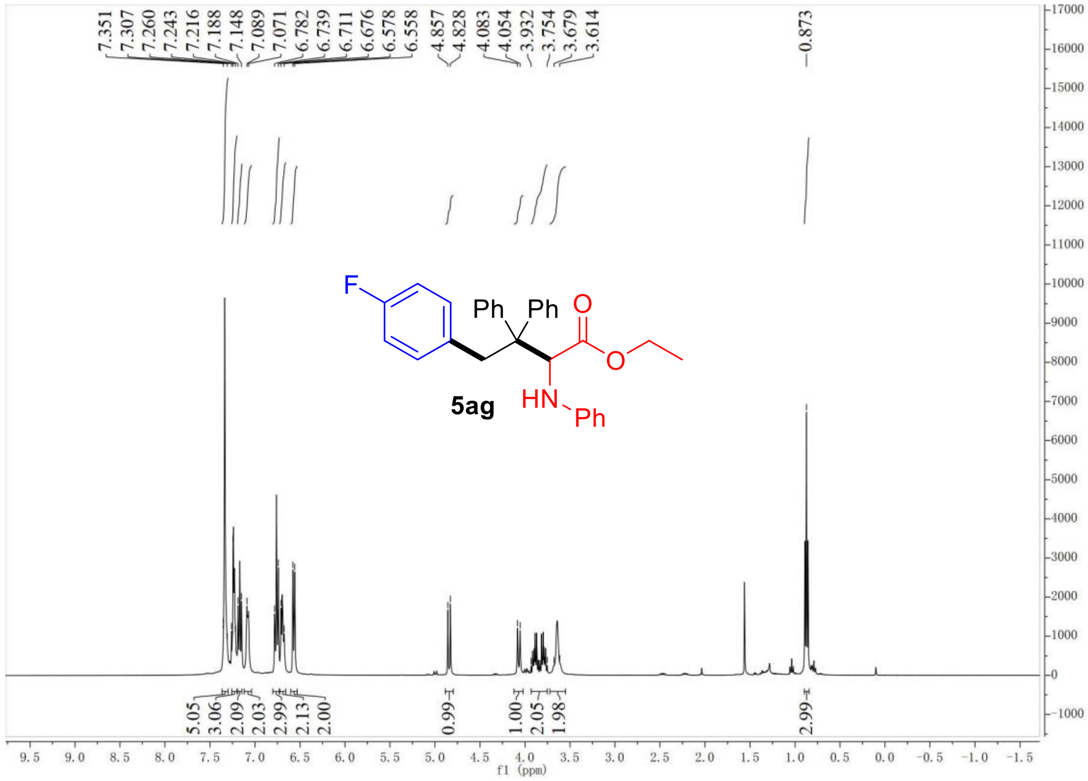

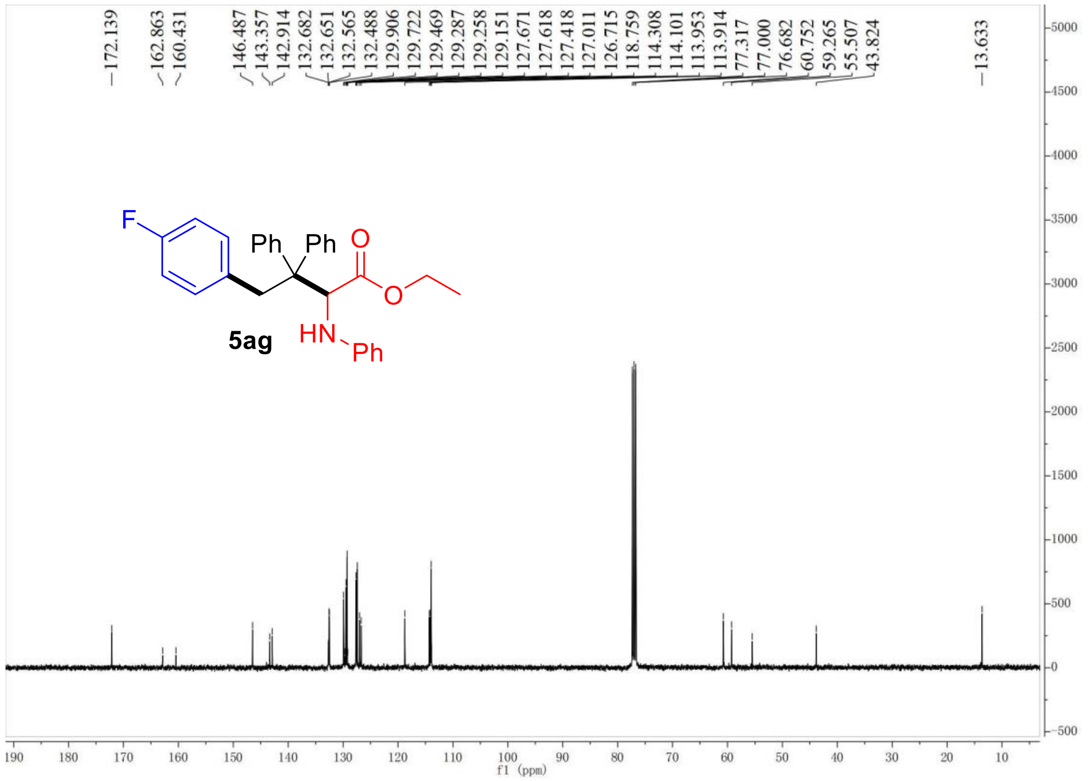

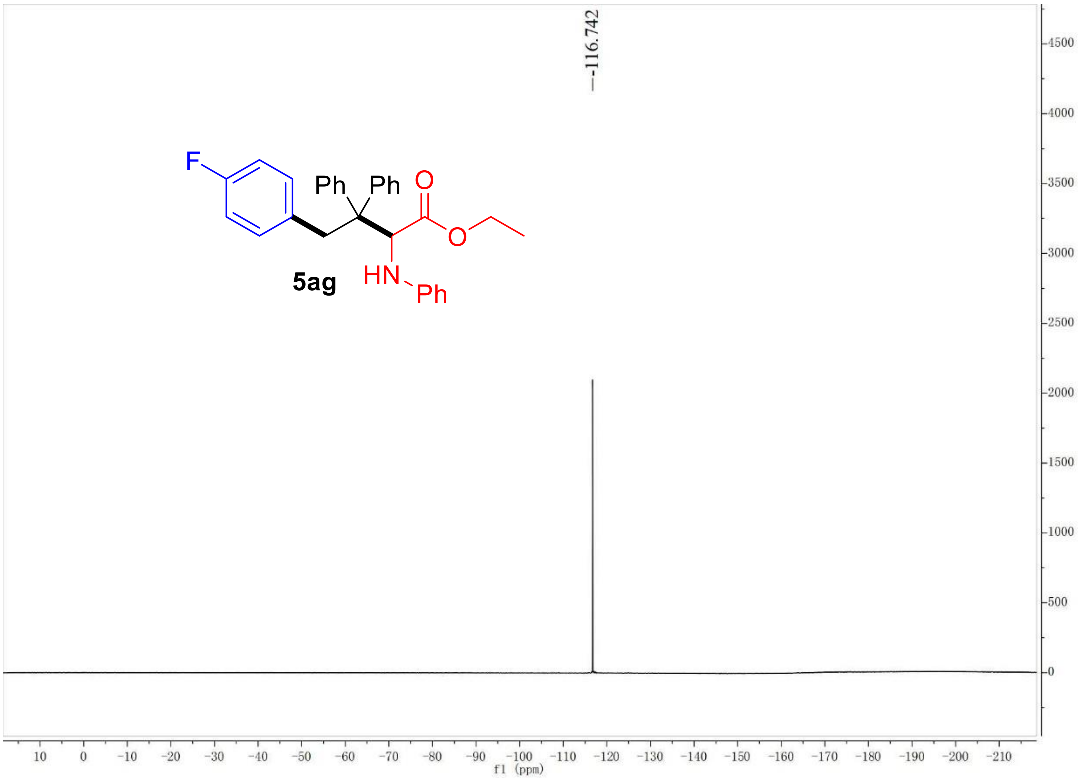
--116.742
F
Ph
Ph
O
O
5ag
HN
Ph
f1 (ppm)

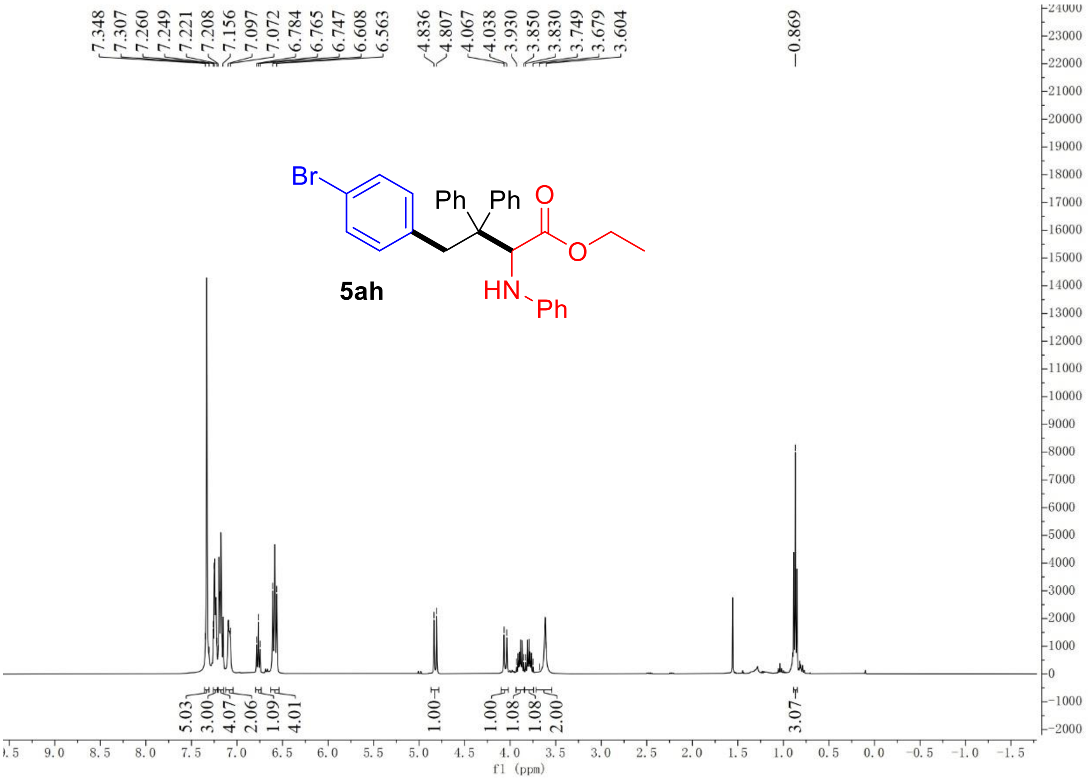
7.348
7.307
7.260
7.249
7.221
7.208
7.156
7.097
7.072
6.784
6.765
6.747
6.608
6.563
4.836
4.807
4.067
4.038
3.930
3.850
3.830
3.749
3.679
3.604
0.869
Br
Ph
Ph
O
O
5ah
HN
Ph
5.03
3.00
4.07
2.06
1.09
4.01
1.00
1.00
1.08
1.08
2.00
3.07
f1 (ppm)

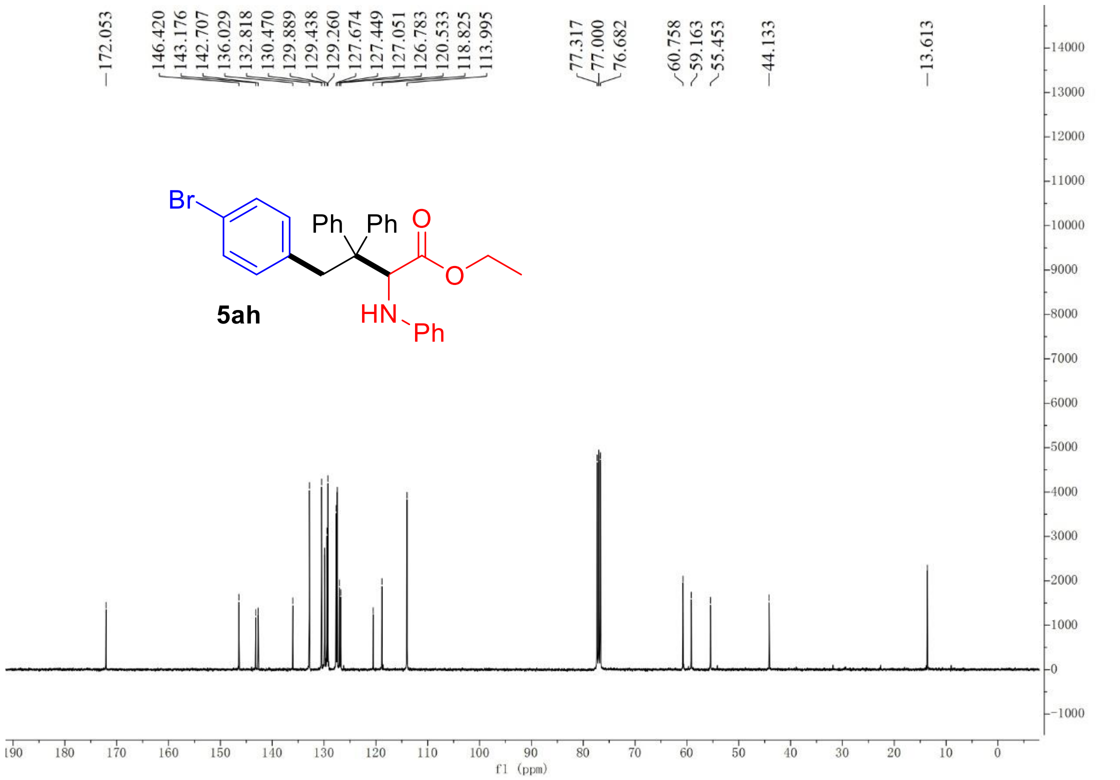
172.053
146.420
143.176
142.707
136.029
132.818
130.470
129.889
129.438
129.260
127.674
127.449
127.051
126.783
120.533
118.825
113.995
77.317
77.000
76.682
60.758
59.163
55.453
44.133
13.613
Br
Ph
Ph
O
O
HN
Ph
5ah
f1 (ppm)

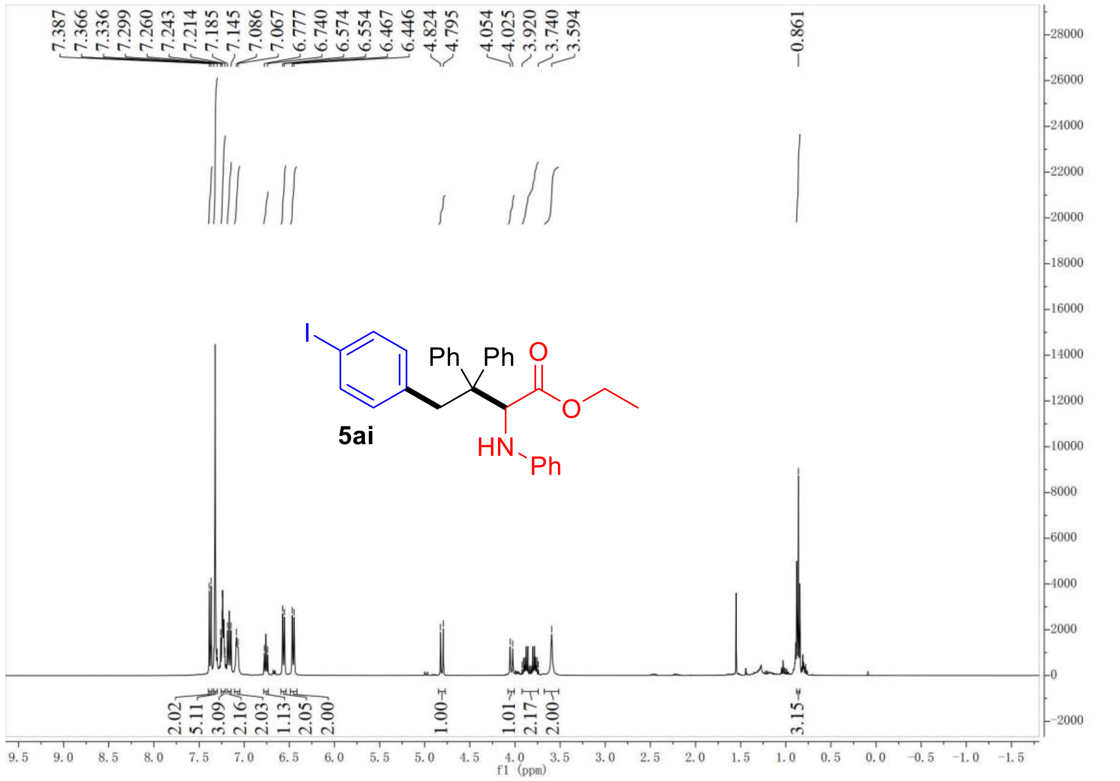
7.387
7.366
7.336
7.299
7.260
7.243
7.214
7.185
7.145
7.086
7.067
6.777
6.740
6.574
6.554
6.467
6.446
4.824
4.795
4.054
4.025
3.920
3.740
3.594
0.861
I
Ph
Ph
O
O
HN
Ph
5ai
2.02
5.11
3.09
2.16
2.03
1.13
2.05
2.00
1.00
1.01
2.17
2.00
3.15
f1 (ppm)

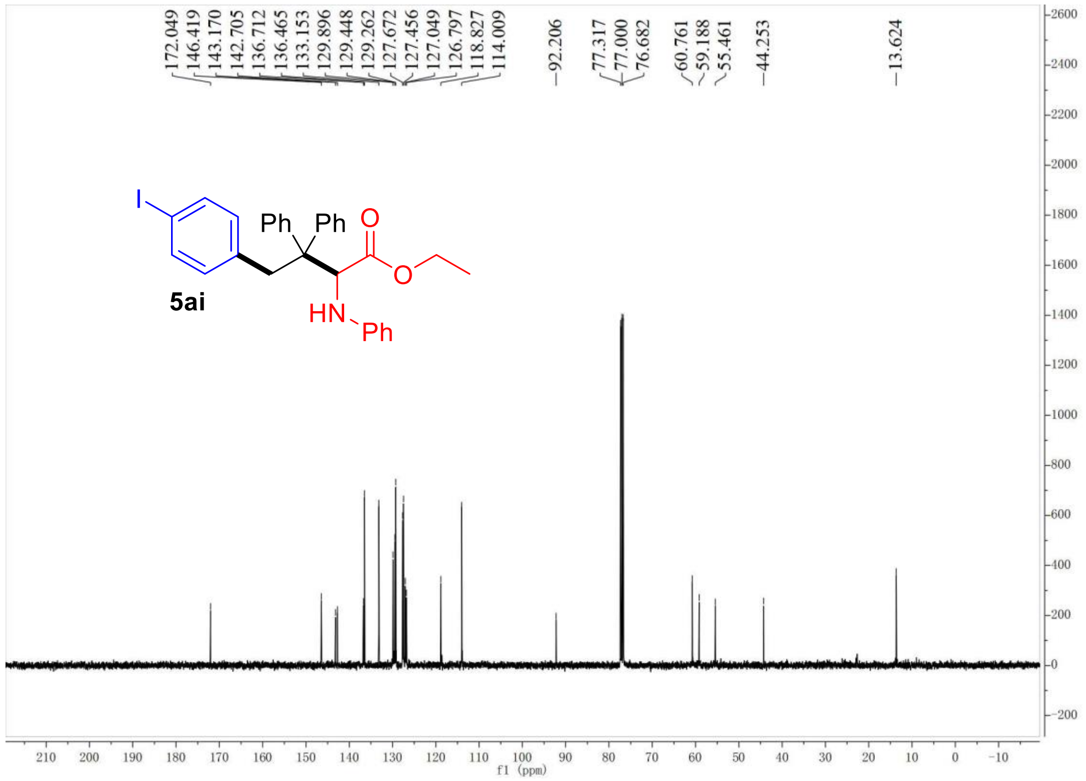
172.049
146.419
143.170
142.705
136.712
136.465
133.153
129.896
129.448
129.262
127.672
127.456
127.049
126.797
118.827
114.009
92.206
77.317
77.000
76.682
60.761
59.188
55.461
44.253
13.624
5ai
f1 (ppm)

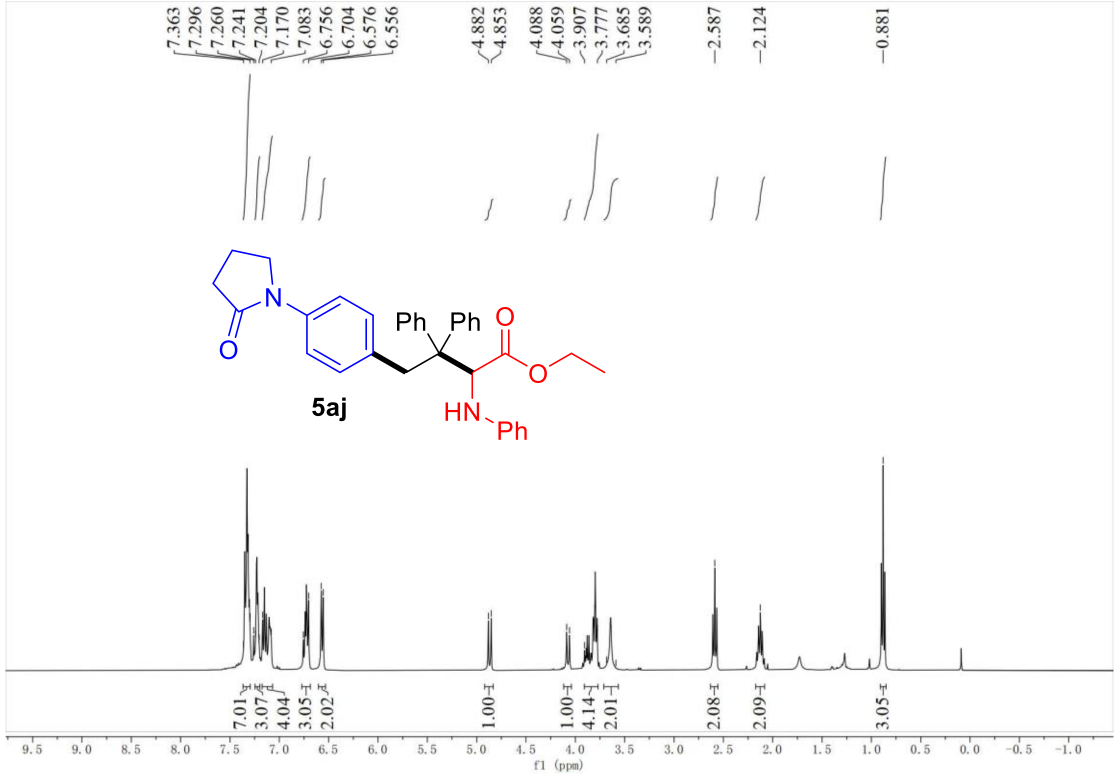
7.363
7.296
7.260
7.241
7.204
7.170
7.083
6.756
6.704
6.576
6.556
4.882
4.853
4.088
4.059
3.907
3.777
3.685
3.589
2.587
2.124
0.881
5aj
7.01
3.07
4.04
3.05
2.02
1.00
1.00
4.14
2.01
2.08
2.09
3.05
f1 (ppm)

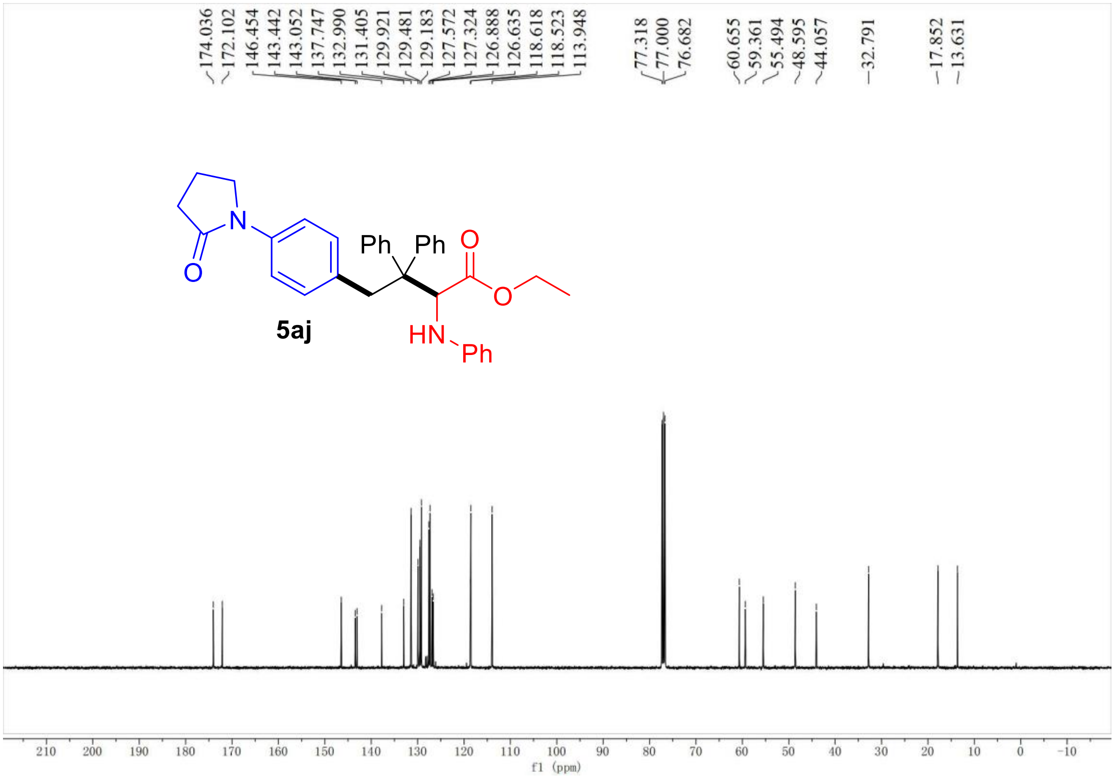
174.036
172.102
146.454
143.442
143.052
137.747
132.990
131.405
129.921
129.481
129.183
127.572
127.324
126.888
126.635
118.618
118.523
113.948
77.318
77.000
76.682
60.655
59.361
55.494
48.595
44.057
32.791
17.852
13.631
5aj
f1 (ppm)

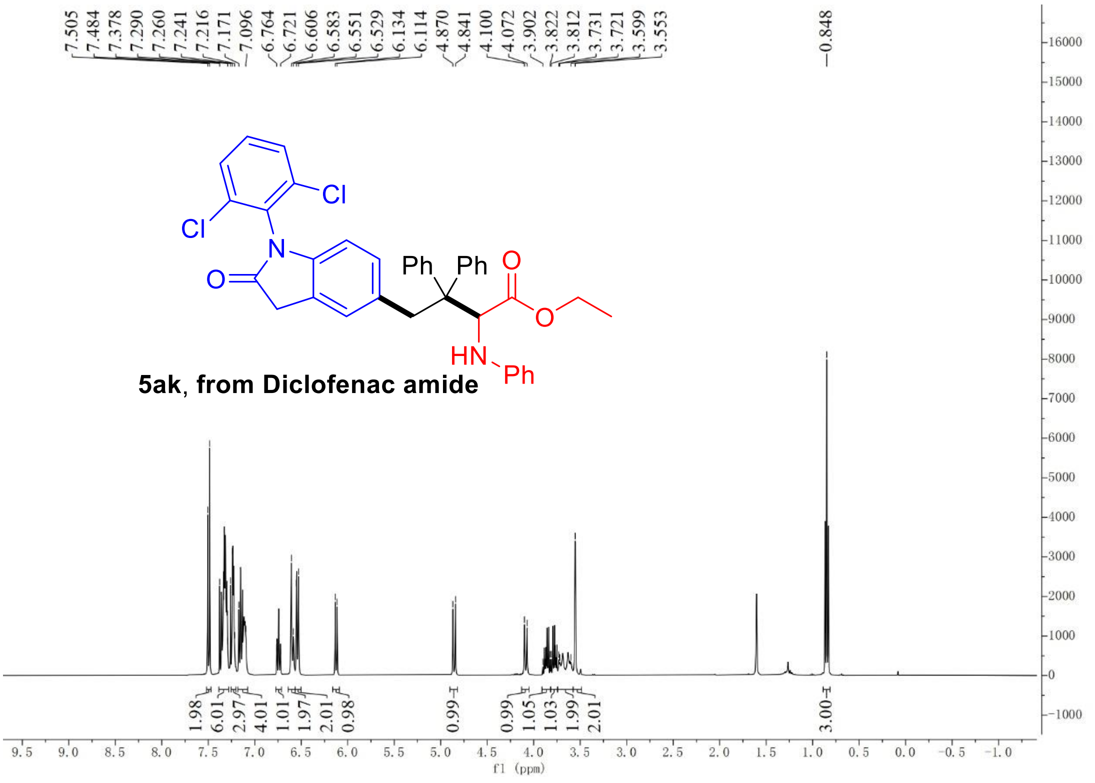
7.505
7.484
7.378
7.290
7.260
7.241
7.216
7.171
7.096
6.764
6.721
6.606
6.583
6.551
6.529
6.134
6.114
4.870
4.841
4.100
4.072
3.902
3.822
3.812
3.731
3.721
3.599
3.553
0.848
5ak, from Diclofenac amide
1.98
6.01
2.97
4.01
1.01
1.97
2.01
0.98
0.99
0.99
1.05
1.03
1.99
2.01
3.00
f1 (ppm)

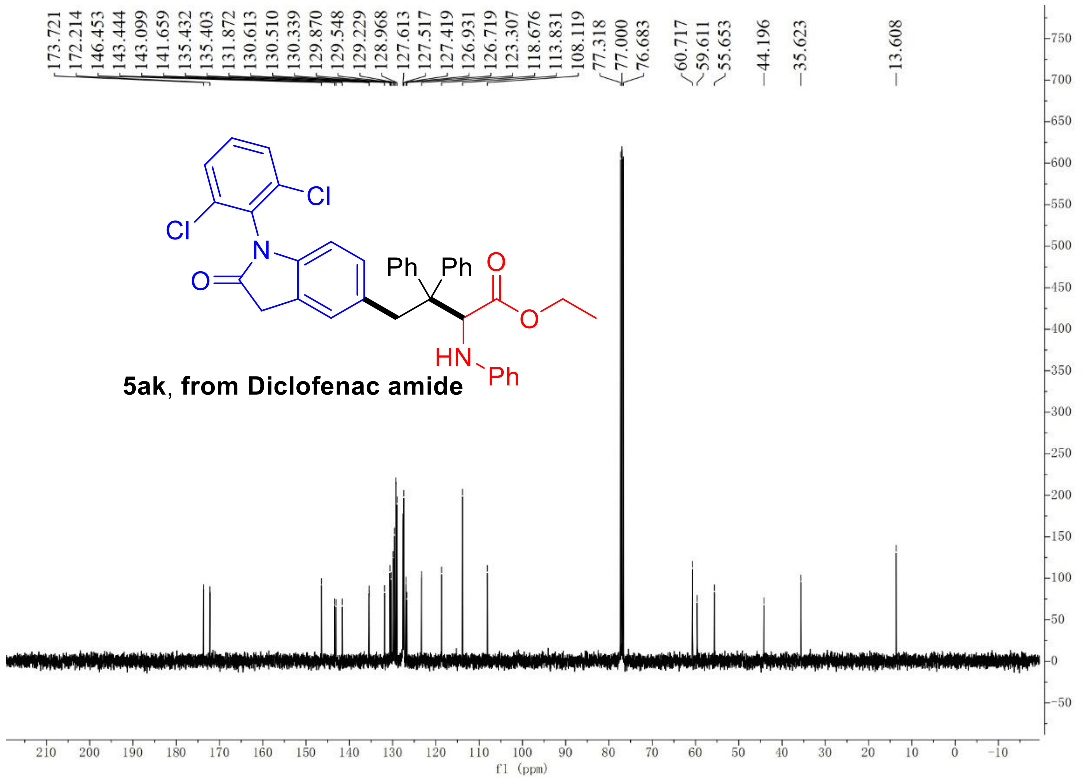
5ak, from Diclofenac amide

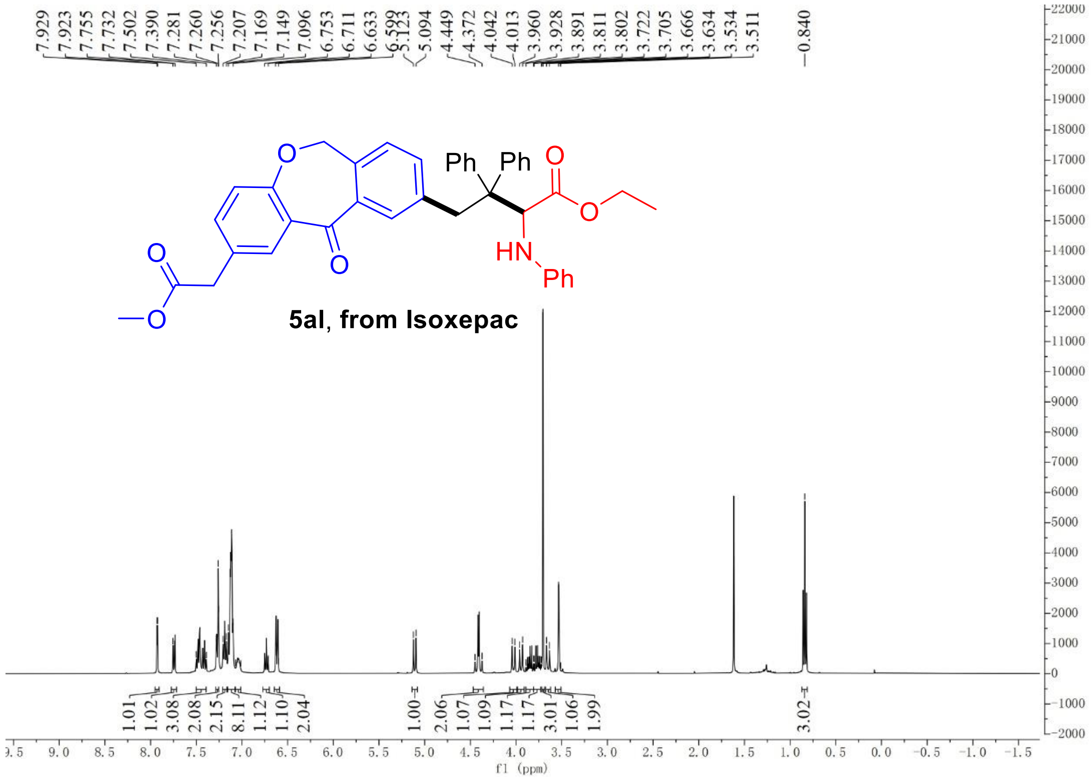
5al, from Isoxepac

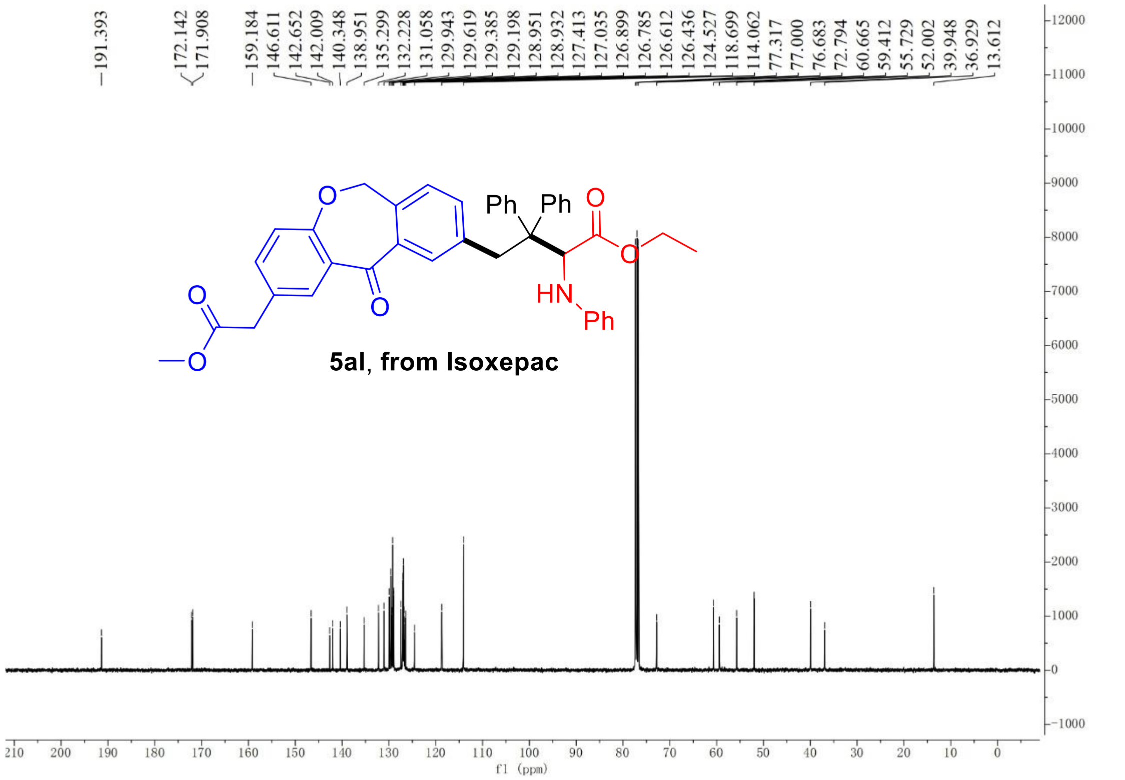
5al, from Isoxepac
Ph
Ph
HN
Ph
f1 (ppm)

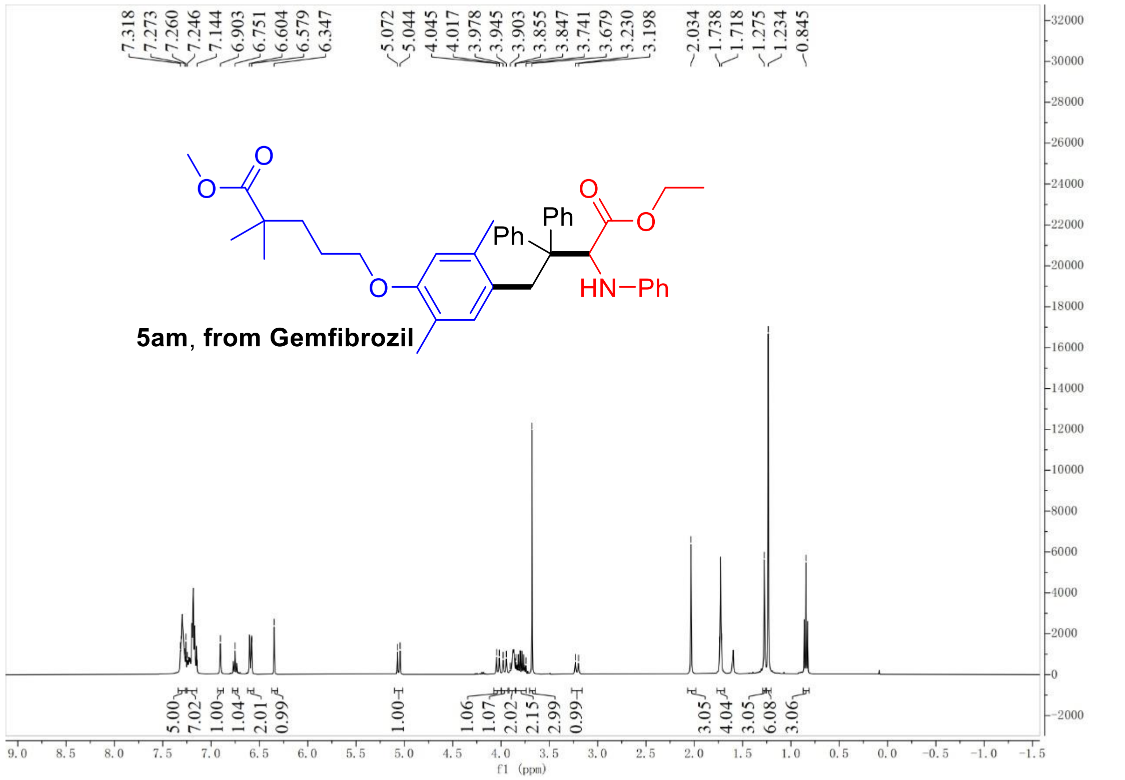
5am, from Gemfibrozil
Ph
Ph
HN−Ph
f1 (ppm)

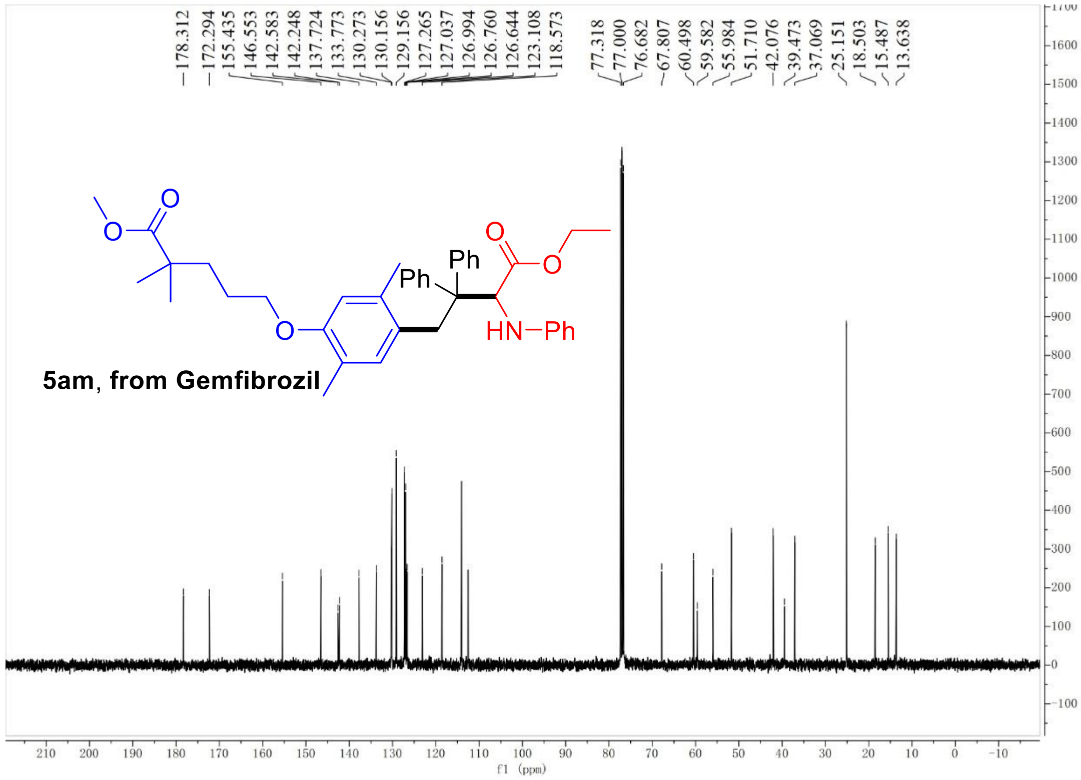
178.312
172.294
155.435
146.553
142.583
142.248
137.724
133.773
130.273
130.156
129.156
127.265
127.037
126.994
126.760
126.644
123.108
118.573
77.318
77.000
76.682
67.807
60.498
59.582
55.984
51.710
42.076
39.473
37.069
25.151
18.503
15.487
13.638
O
O
O
Ph
Ph
O
O
HN−Ph
5am, from Gemfibrozil
f1 (ppm)

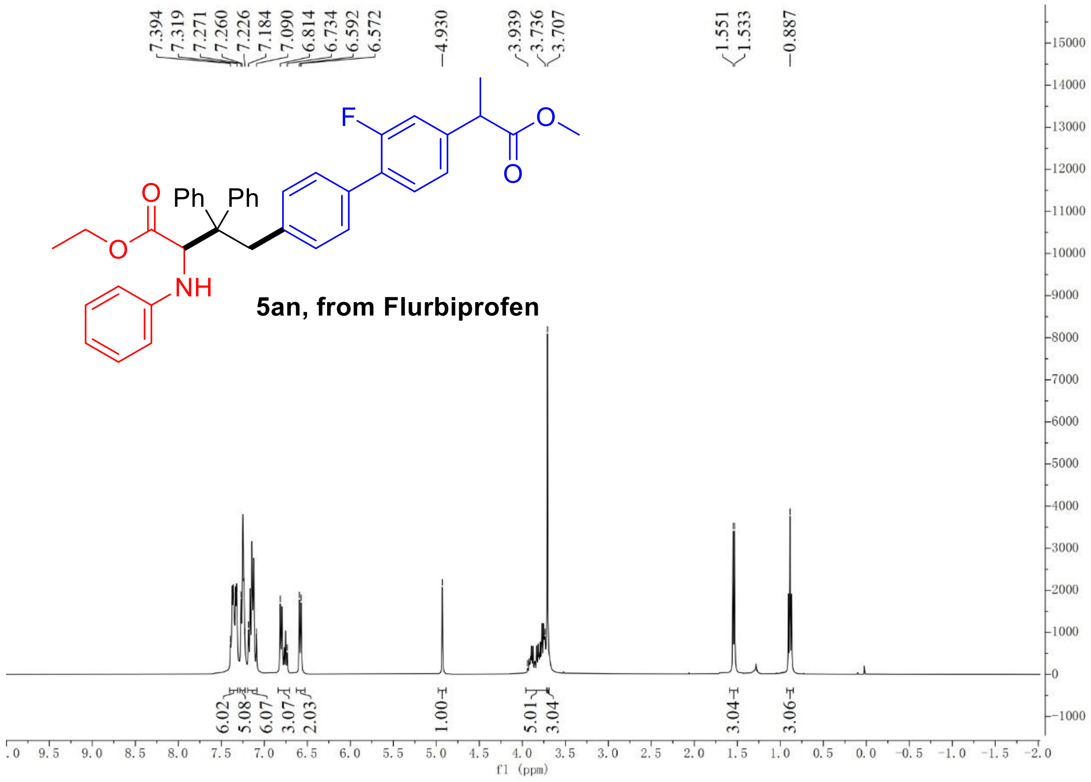
7.394
7.319
7.271
7.260
7.226
7.184
7.090
6.814
6.734
6.592
6.572
4.930
3.939
3.736
3.707
1.551
1.533
0.887
F
O
O
O
Ph
Ph
O
NH
5an, from Flurbiprofen
6.02
5.08
6.07
3.07
2.03
1.00
5.01
3.04
3.04
3.06
f1 (ppm)

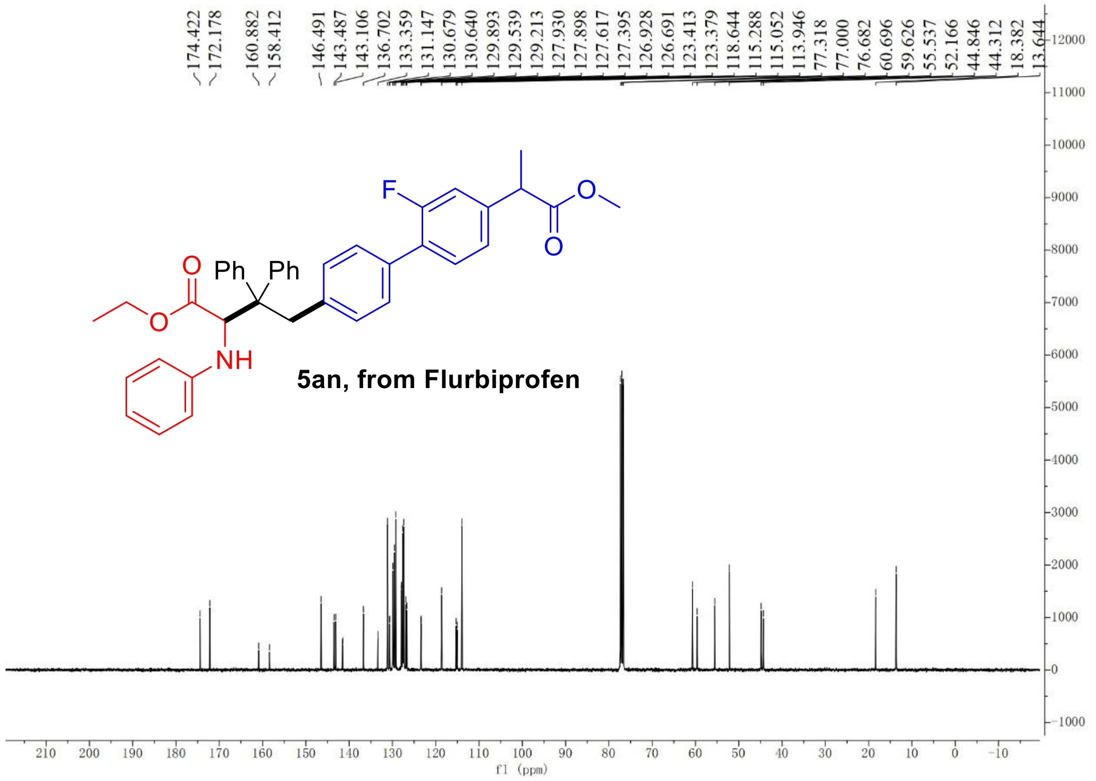
174.422
172.178
160.882
158.412
146.491
143.487
143.106
136.702
133.359
131.147
130.679
130.640
129.893
129.539
129.213
127.930
127.898
127.617
127.395
126.928
126.691
123.413
123.379
118.644
115.288
115.052
113.946
77.318
77.000
76.682
60.696
59.626
55.537
52.166
44.846
44.312
18.382
13.644
F
O
O
O
Ph
Ph
O
NH
5an, from Flurbiprofen
f1 (ppm)

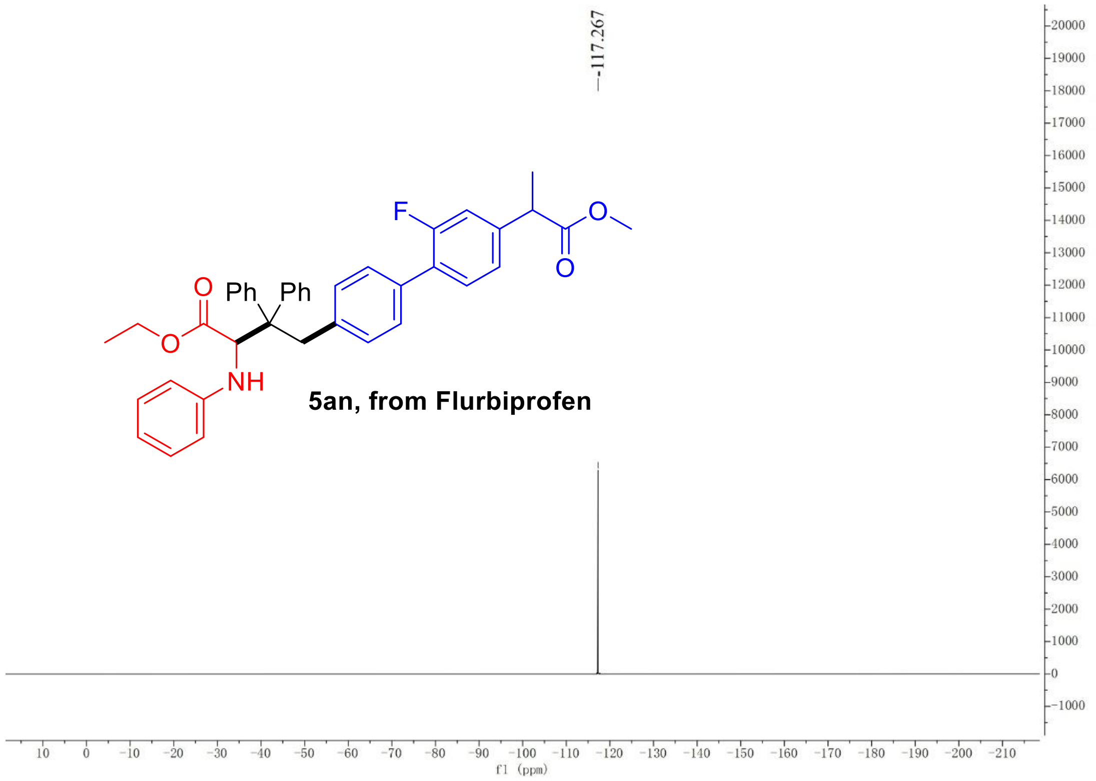
-117.267
F
O
O
O
Ph
Ph
O
NH
5an, from Flurbiprofen
f1 (ppm)

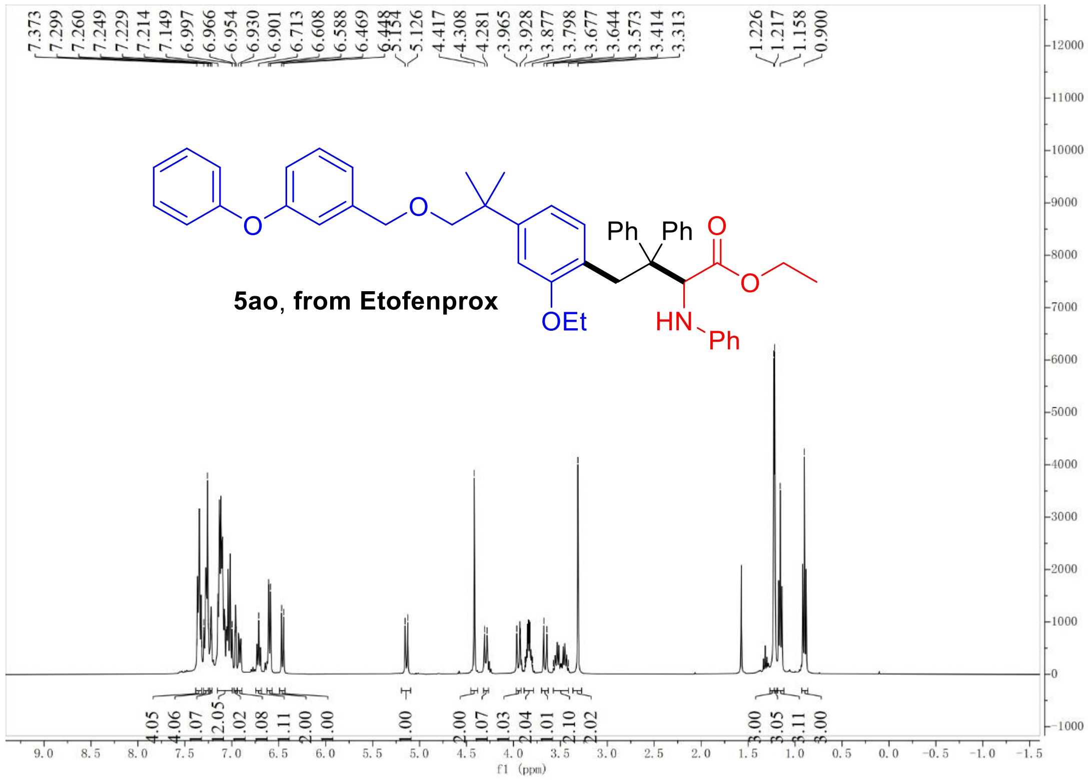
5ao, from Etofenprox
OEt
Ph
Ph
O
O
O
O
HN
Ph
f1 (ppm)

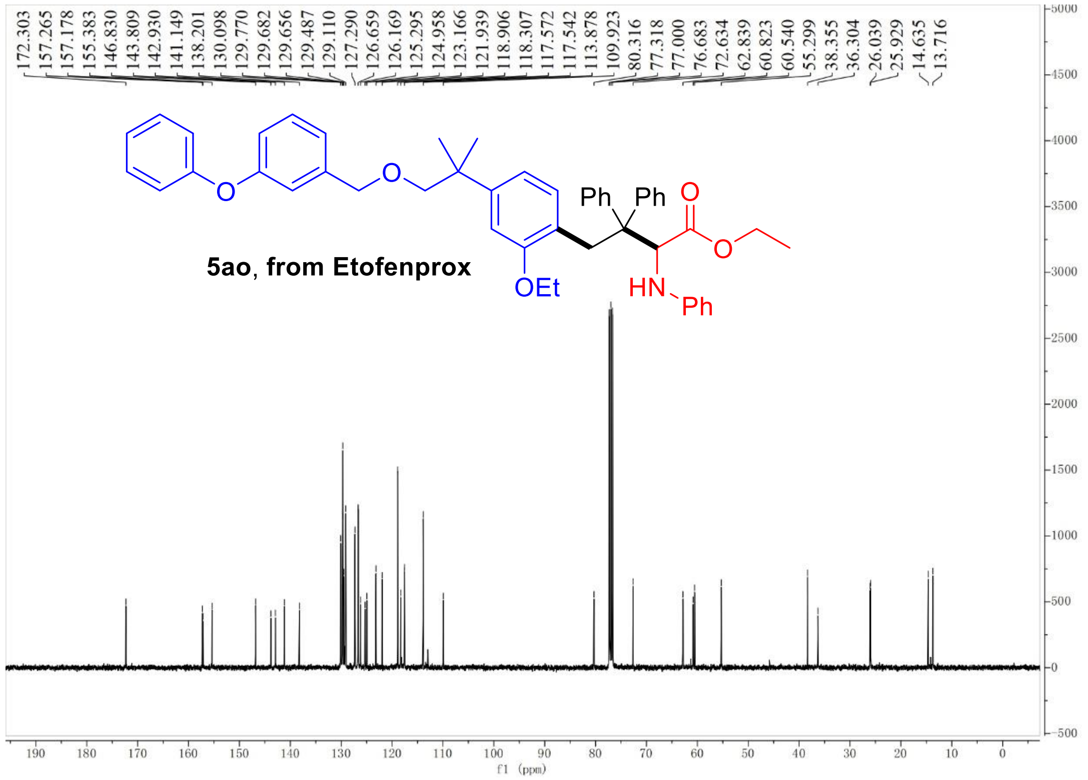
5ao, from Etofenprox
OEt
Ph
Ph
O
O
O
O
HN
Ph
f1 (ppm)

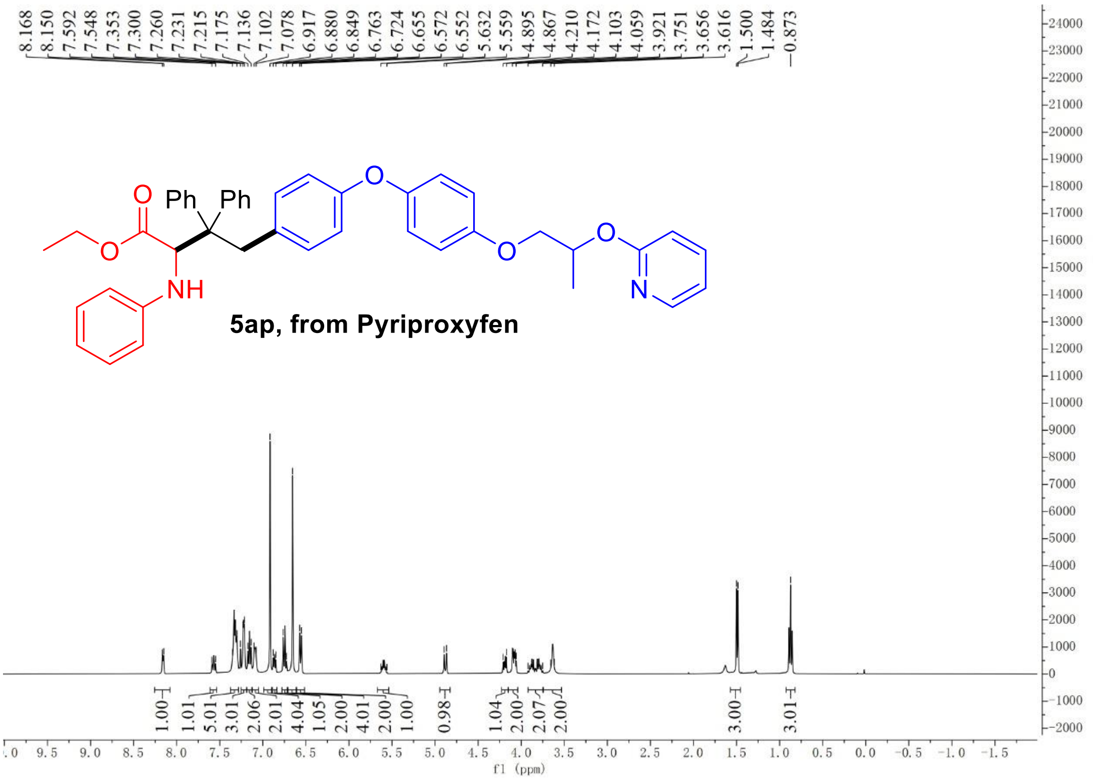
5ap, from Pyriproxyfen

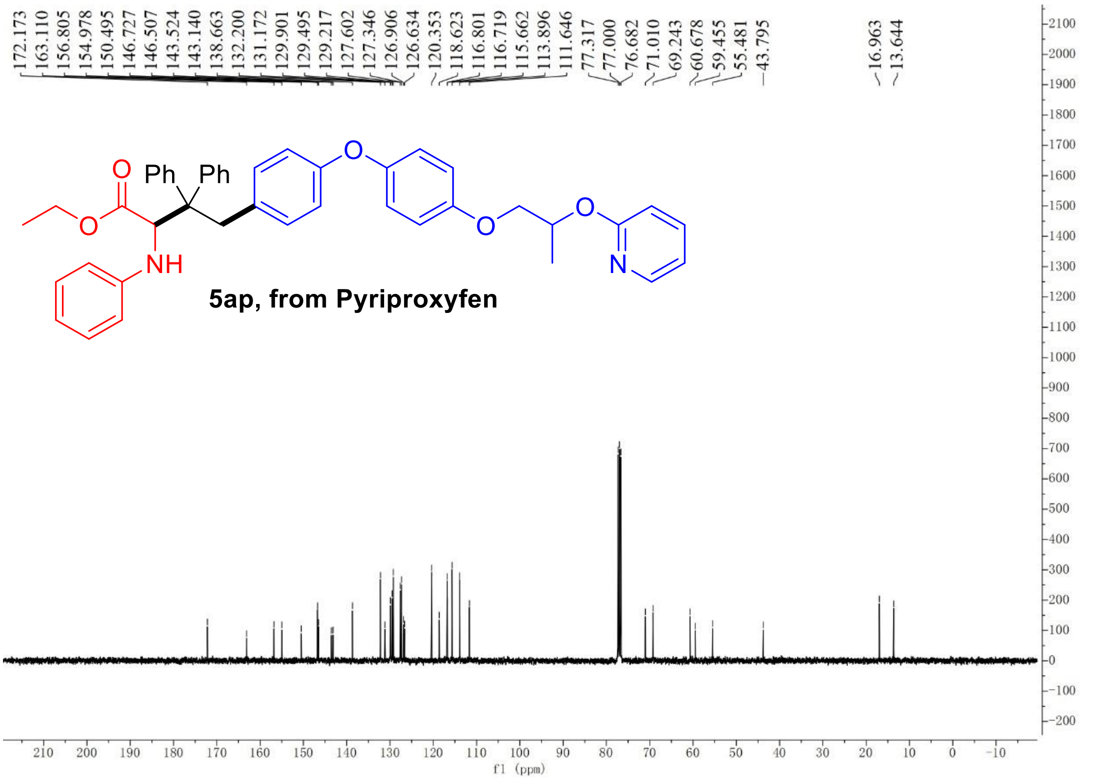
5ap, from Pyriproxyfen

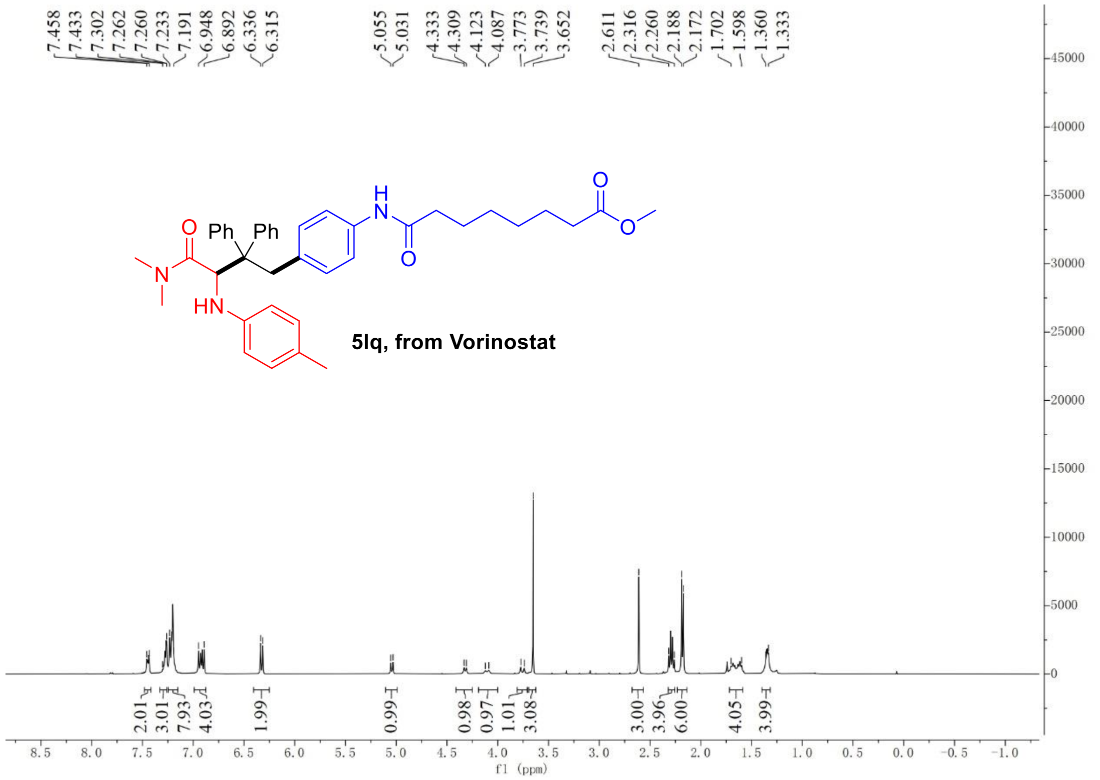
5lq, from Vorinostat
f1 (ppm)

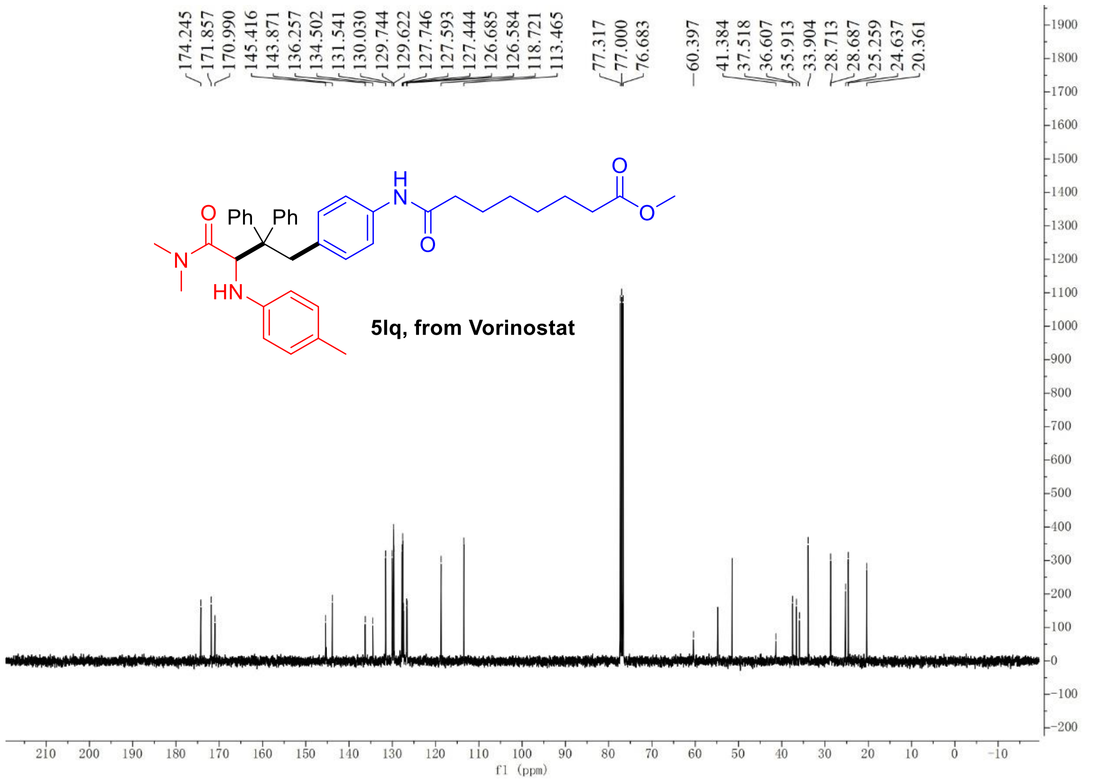
5lq, from Vorinostat
f1 (ppm)

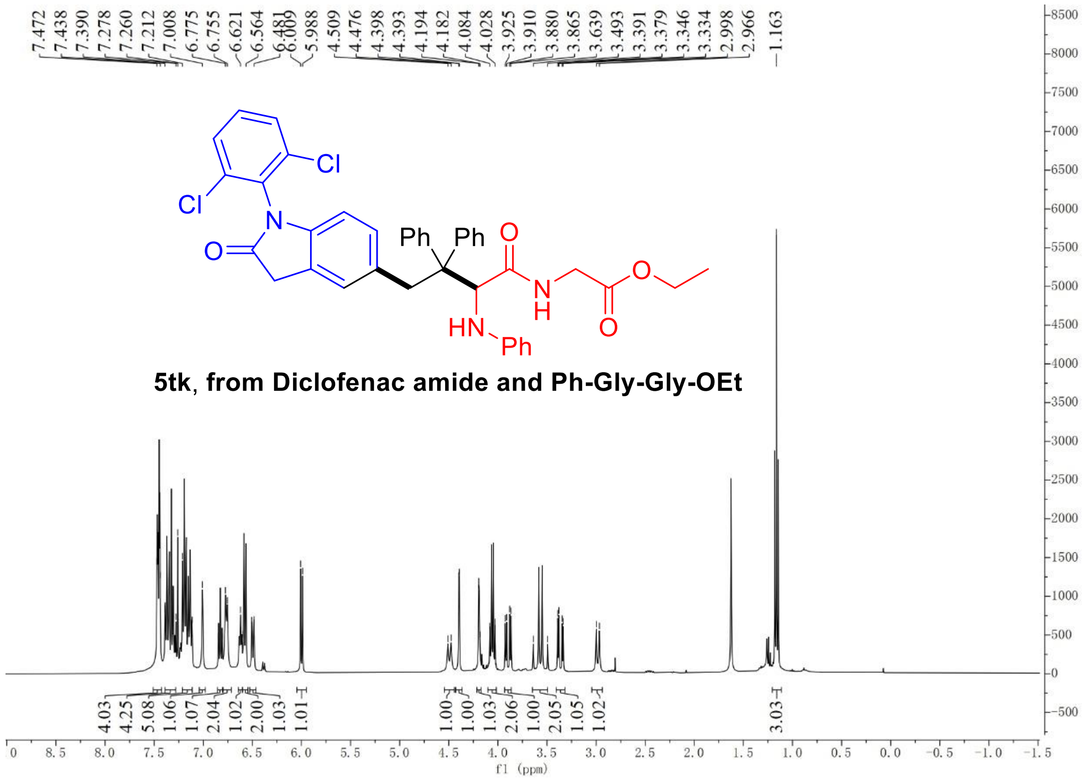
7.472
7.438
7.390
7.278
7.260
7.212
7.008
6.775
6.755
6.621
6.564
6.481
6.009
5.988
4.509
4.476
4.398
4.393
4.194
4.182
4.084
4.028
3.925
3.910
3.880
3.865
3.639
3.493
3.391
3.379
3.346
3.334
2.998
2.966
1.163
5tk, from Diclofenac amide and Ph-Gly-Gly-OEt
4.03
4.25
5.08
1.06
1.07
2.04
1.02
2.00
1.03
1.01
1.00
1.00
1.03
2.06
1.00
2.05
1.05
1.02
3.03
f1 (ppm)

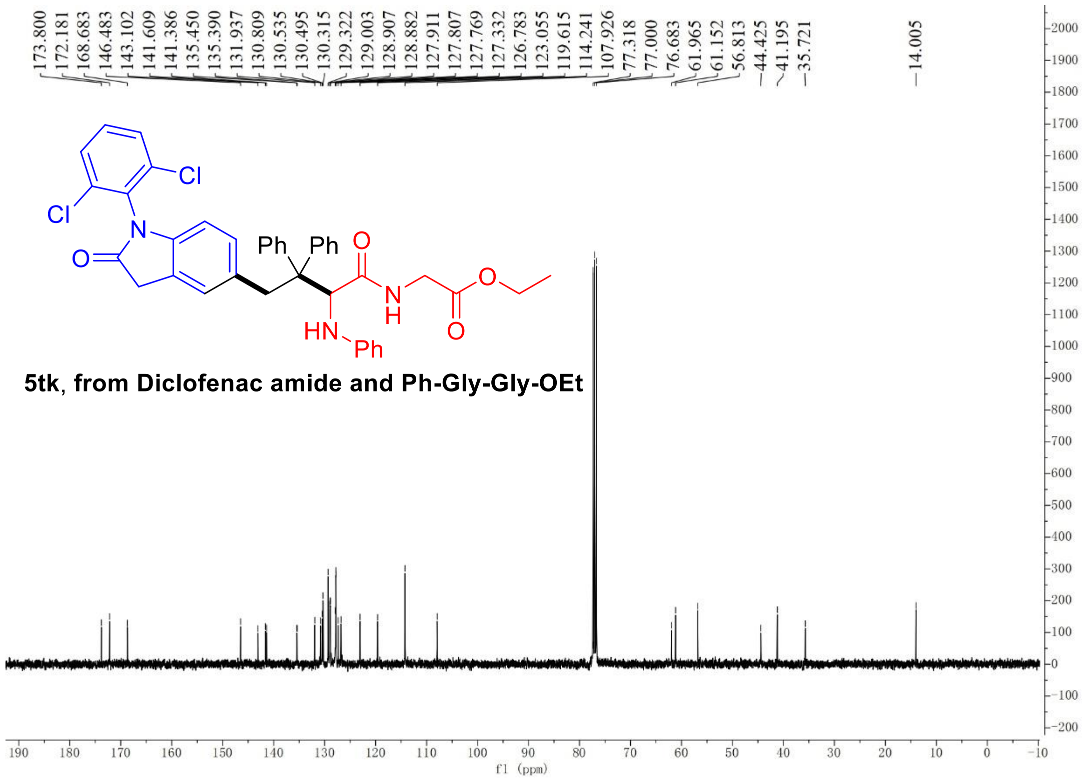
173.800
172.181
168.683
146.483
143.102
141.609
141.386
135.450
135.390
131.937
130.809
130.535
130.495
130.315
129.322
129.003
128.907
128.882
127.911
127.807
127.769
127.332
126.783
123.055
119.615
114.241
107.926
77.318
77.000
76.683
61.965
61.152
56.813
44.425
41.195
35.721
14.005
5tk, from Diclofenac amide and Ph-Gly-Gly-OEt
f1 (ppm)

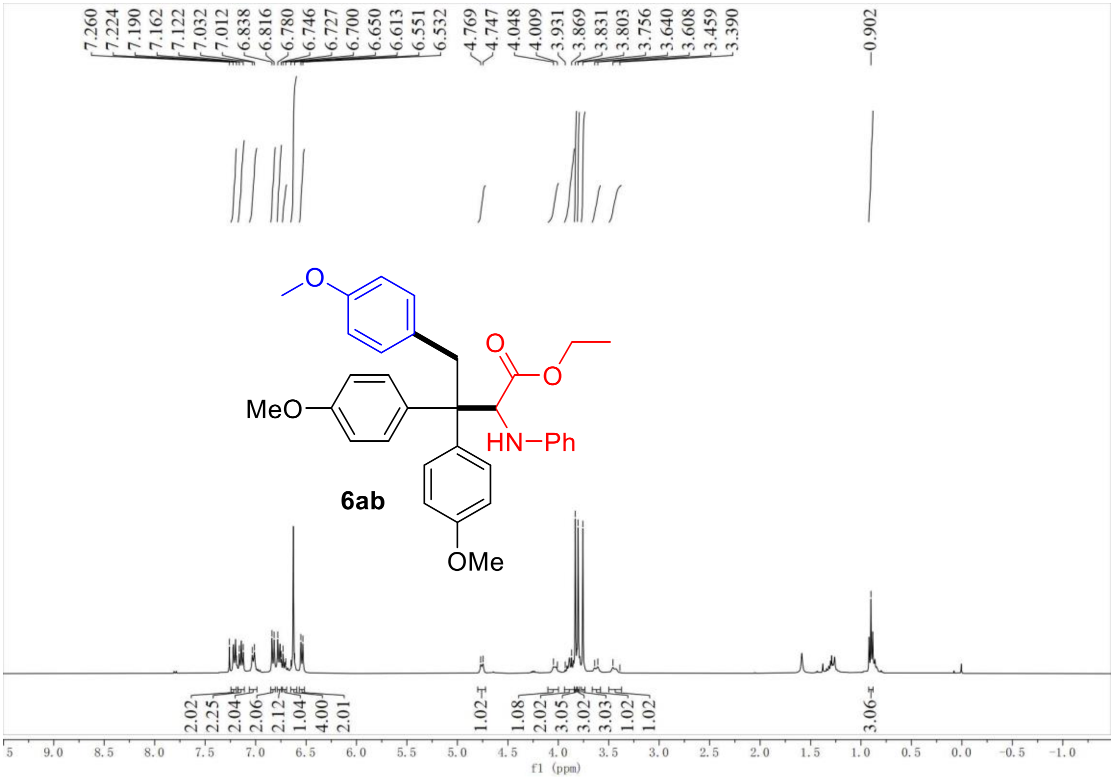

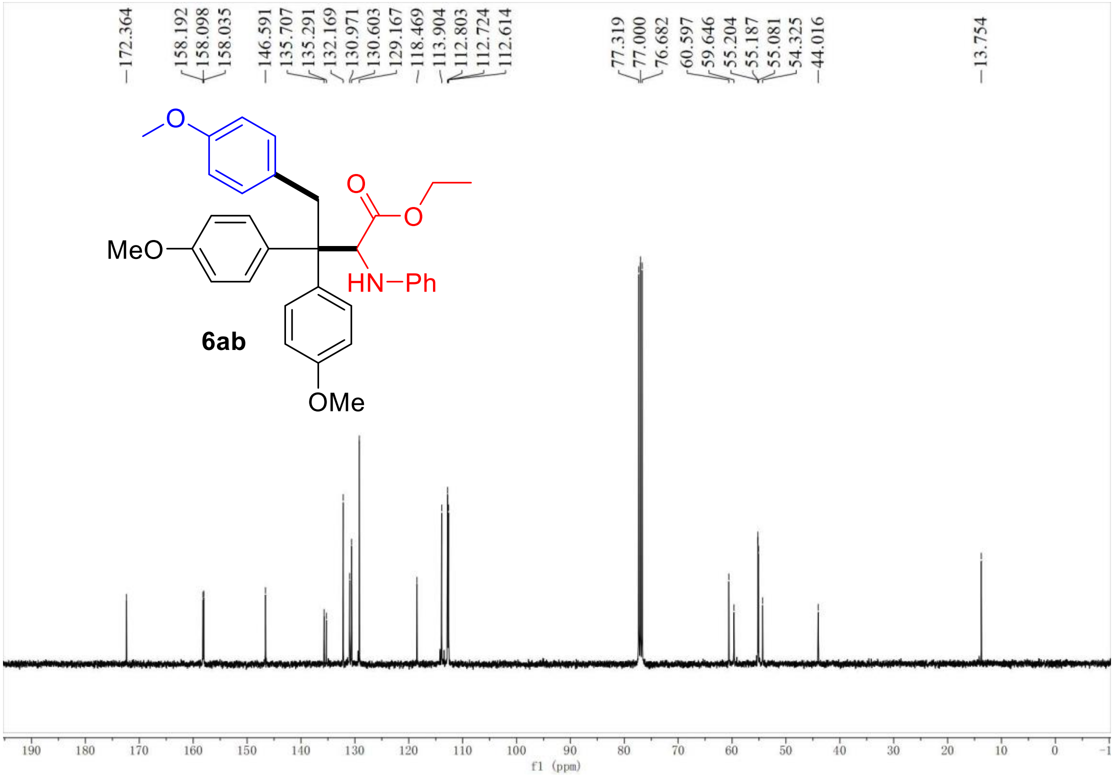

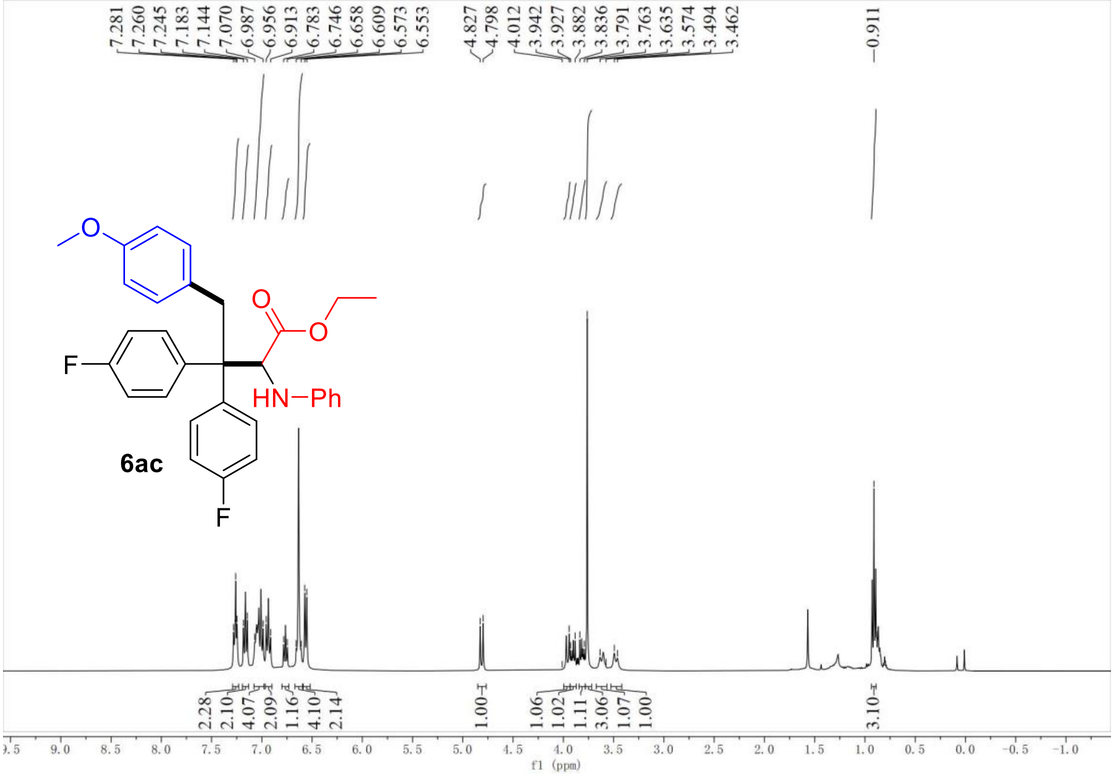
6ac
HN−Ph
f1 (ppm)

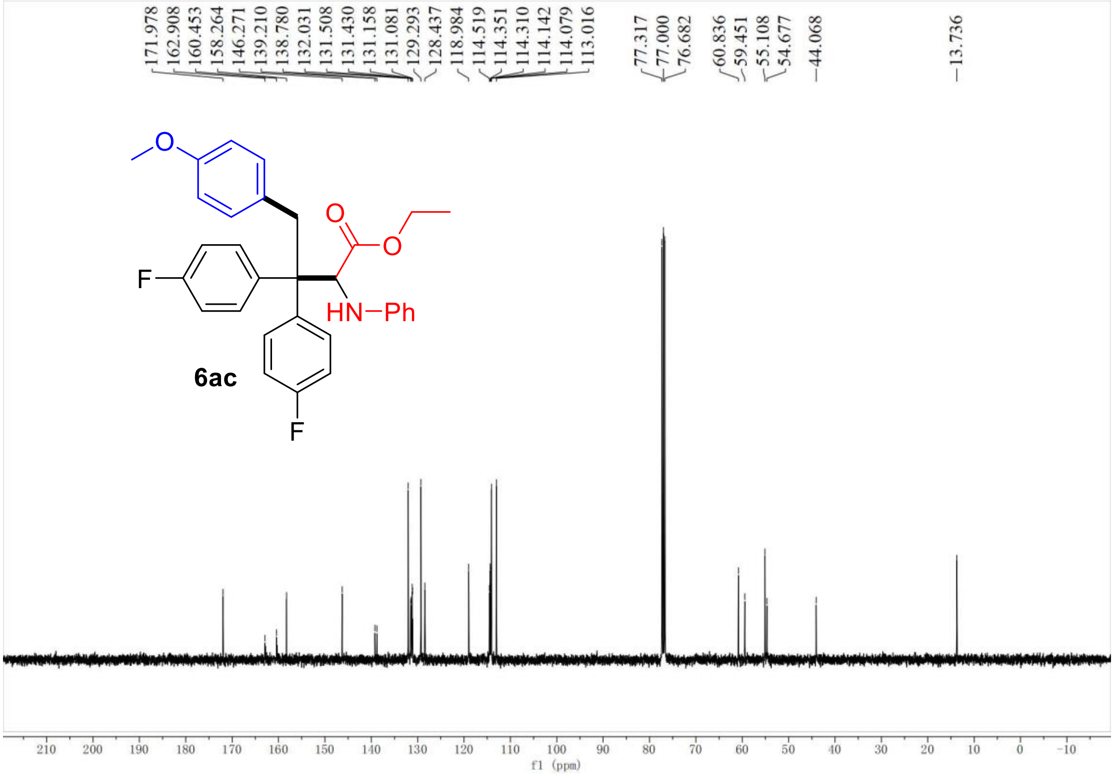
6ac
HN−Ph
f1 (ppm)

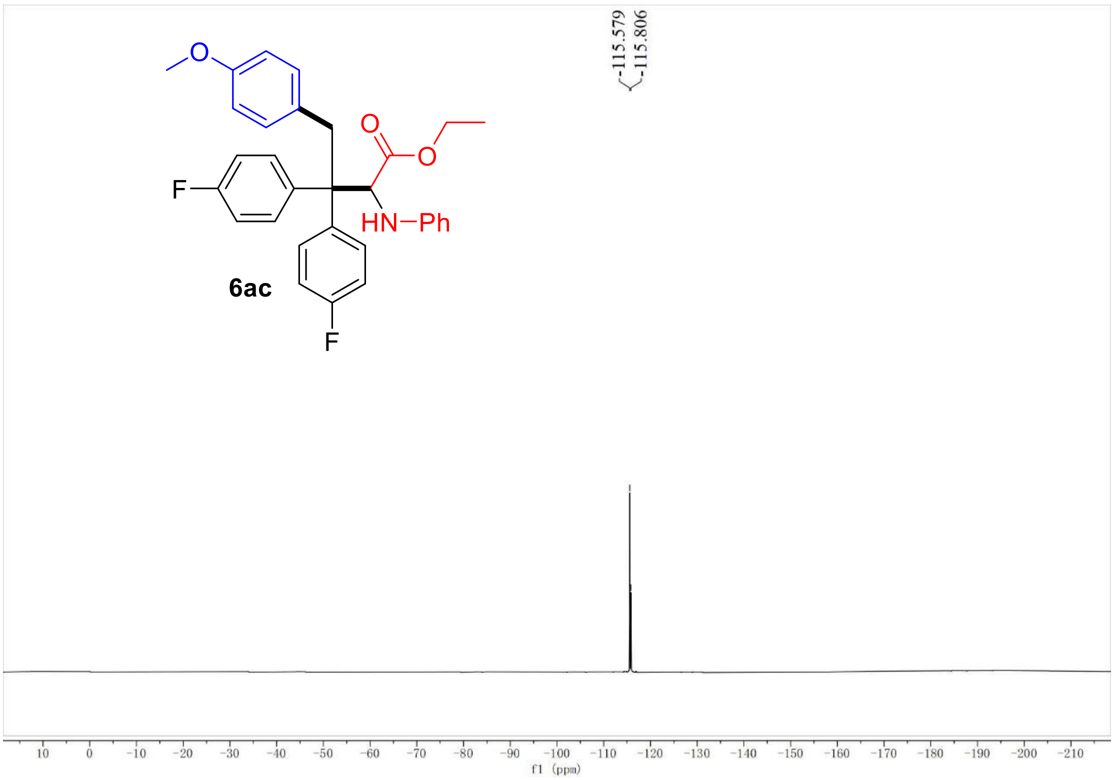

6ac
-115.579
-115.806
f1 (ppm)

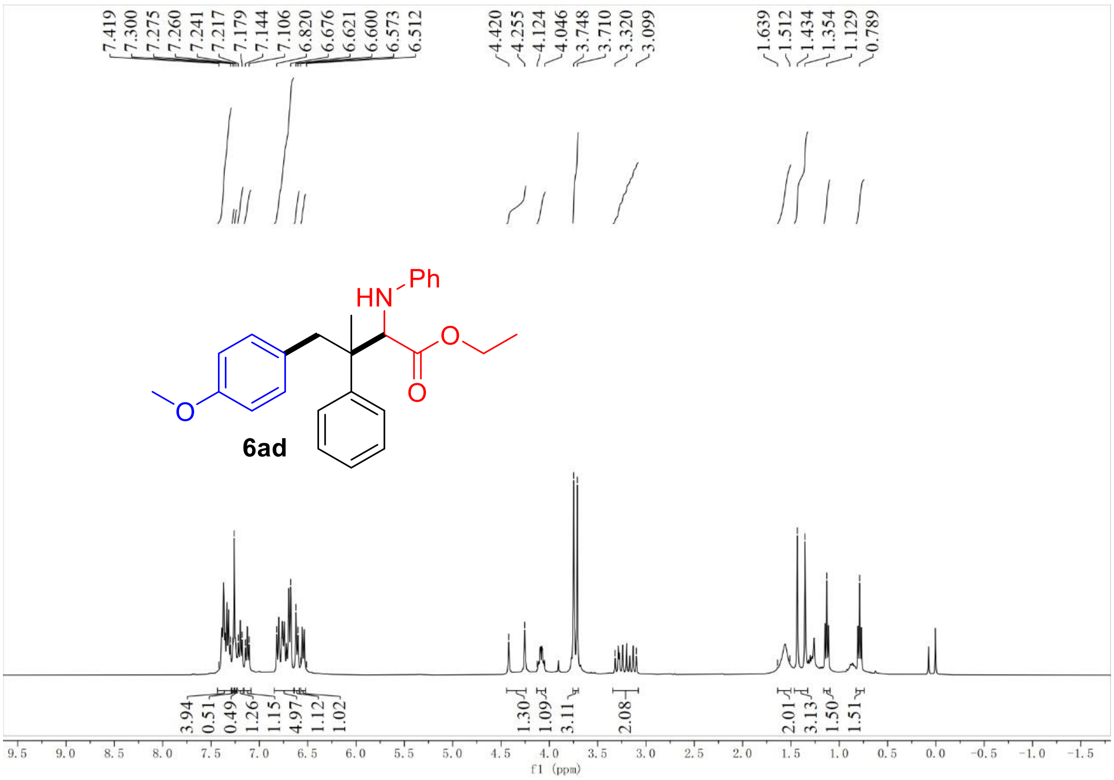

6ad
HN
Ph
f1 (ppm)

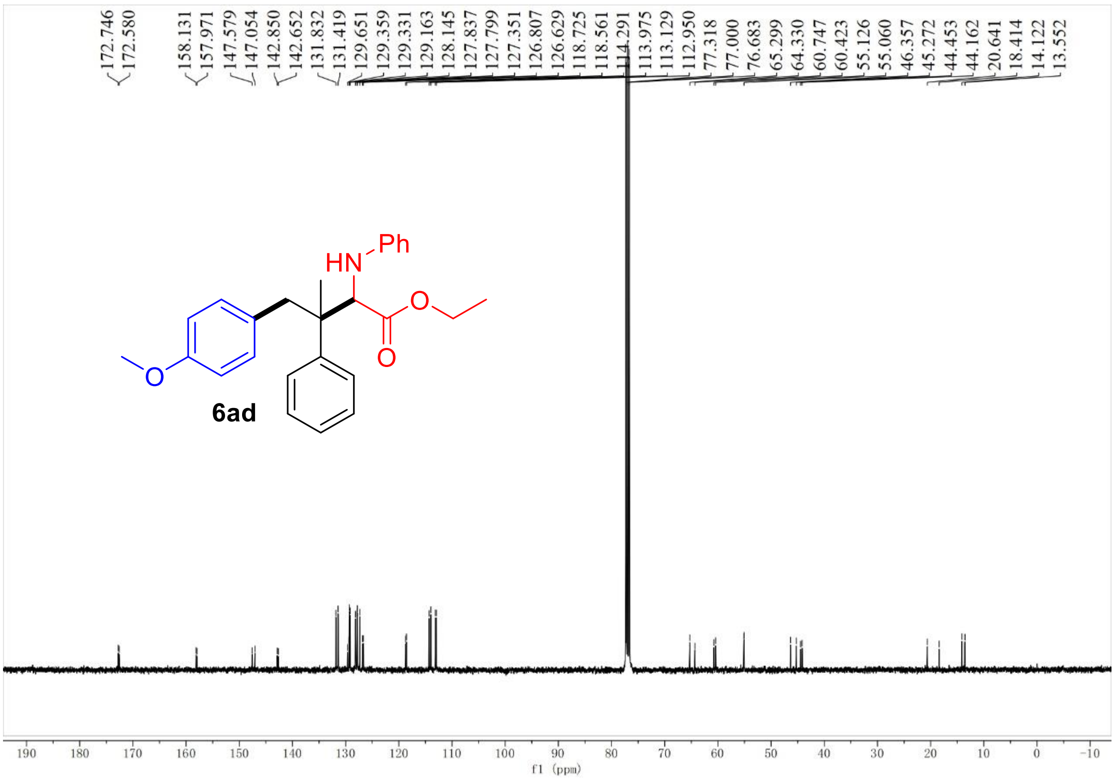
172.746
172.580
158.131
157.971
147.579
147.054
142.850
142.652
131.832
131.419
129.651
129.359
129.331
129.163
128.145
127.837
127.799
127.351
126.807
126.629
118.725
118.561
114.291
113.975
113.129
112.950
77.318
77.000
76.683
65.299
64.330
60.747
60.423
55.126
55.060
46.357
45.272
44.453
44.162
20.641
18.414
14.122
13.552
HN
Ph
O
O
O
6ad
190
180
170
160
150
140
130
120
110
100
90
80
70
60
50
40
30
20
10
0
-10
f1 (ppm)

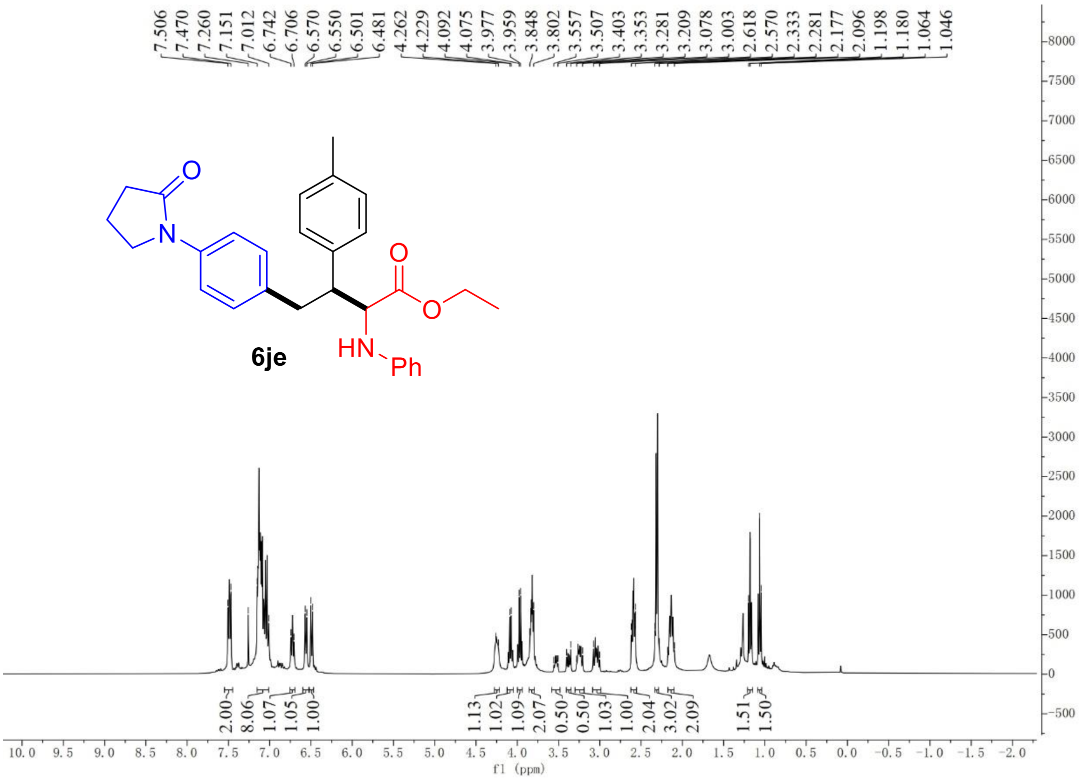
7.506
7.470
7.260
7.151
7.012
6.742
6.706
6.570
6.550
6.501
6.481
4.262
4.229
4.092
4.075
3.977
3.959
3.848
3.802
3.557
3.507
3.403
3.353
3.281
3.209
3.078
3.003
2.618
2.570
2.333
2.281
2.177
2.096
1.198
1.180
1.064
1.046
O
N
O
O
6je
HN
Ph
2.00
8.06
1.07
1.05
1.00
1.13
1.02
1.09
2.07
0.50
0.50
1.03
1.00
2.04
3.02
2.09
1.51
1.50
f1 (ppm)

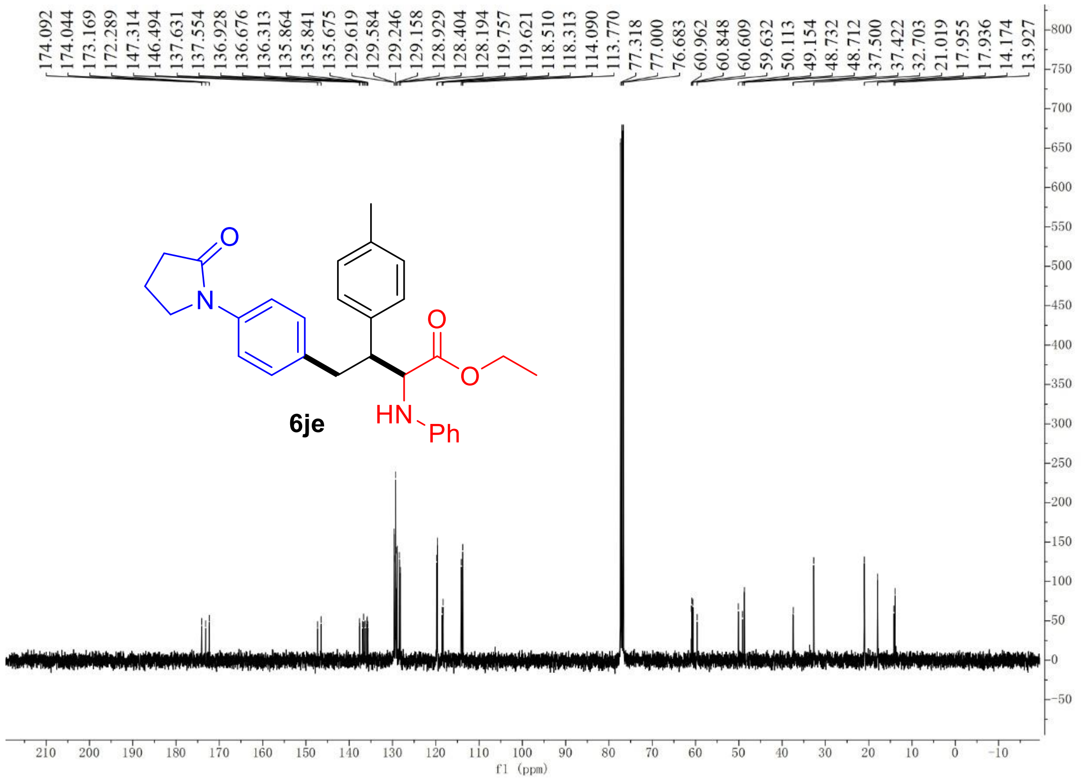
174.092
174.044
173.169
172.289
147.314
146.494
137.631
137.554
136.928
136.676
136.313
135.864
135.841
135.675
129.619
129.584
129.246
129.158
128.929
128.404
128.194
119.757
119.621
118.510
118.313
114.090
113.770
77.318
77.000
76.683
60.962
60.848
60.609
59.632
50.113
49.154
48.732
48.712
37.500
37.422
32.703
21.019
17.955
17.936
14.174
13.927
6je
HN
Ph
f1 (ppm)

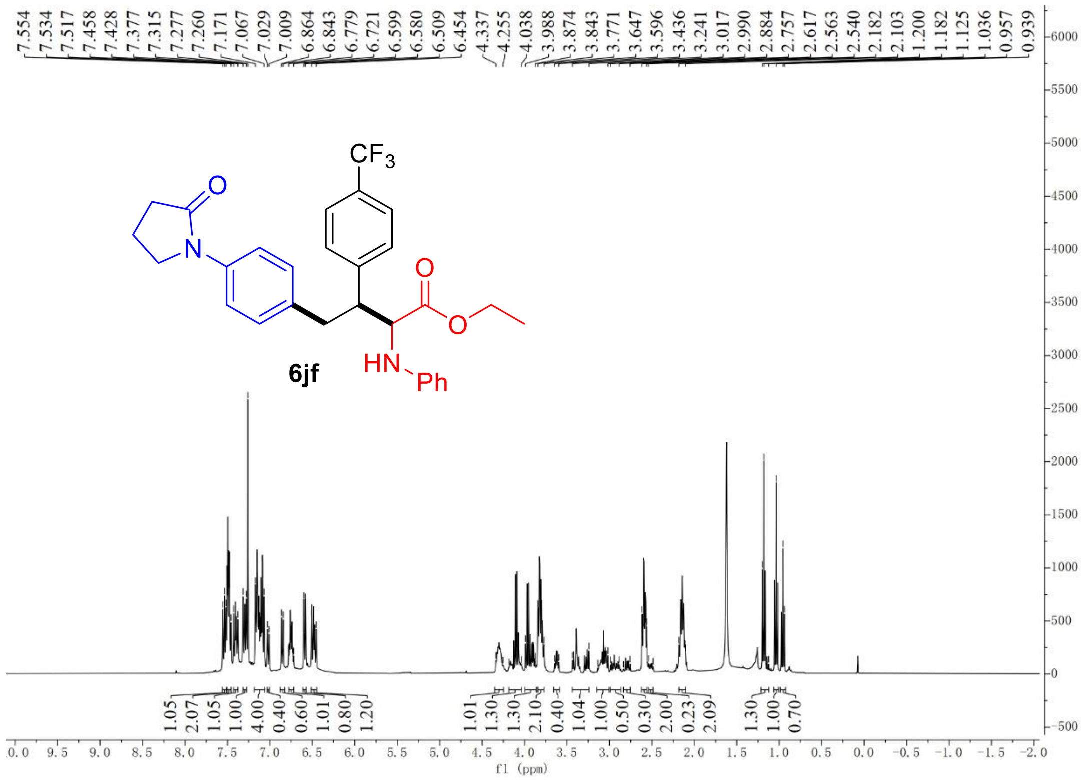
7.554
7.534
7.517
7.458
7.428
7.377
7.315
7.277
7.260
7.171
7.067
7.029
7.009
6.864
6.843
6.779
6.721
6.599
6.580
6.509
6.454
4.337
4.255
4.038
3.988
3.874
3.843
3.771
3.647
3.596
3.436
3.241
3.017
2.990
2.884
2.757
2.617
2.563
2.540
2.182
2.103
1.200
1.182
1.125
1.036
0.957
0.939
CF3
6jf
HN
Ph
f1 (ppm)

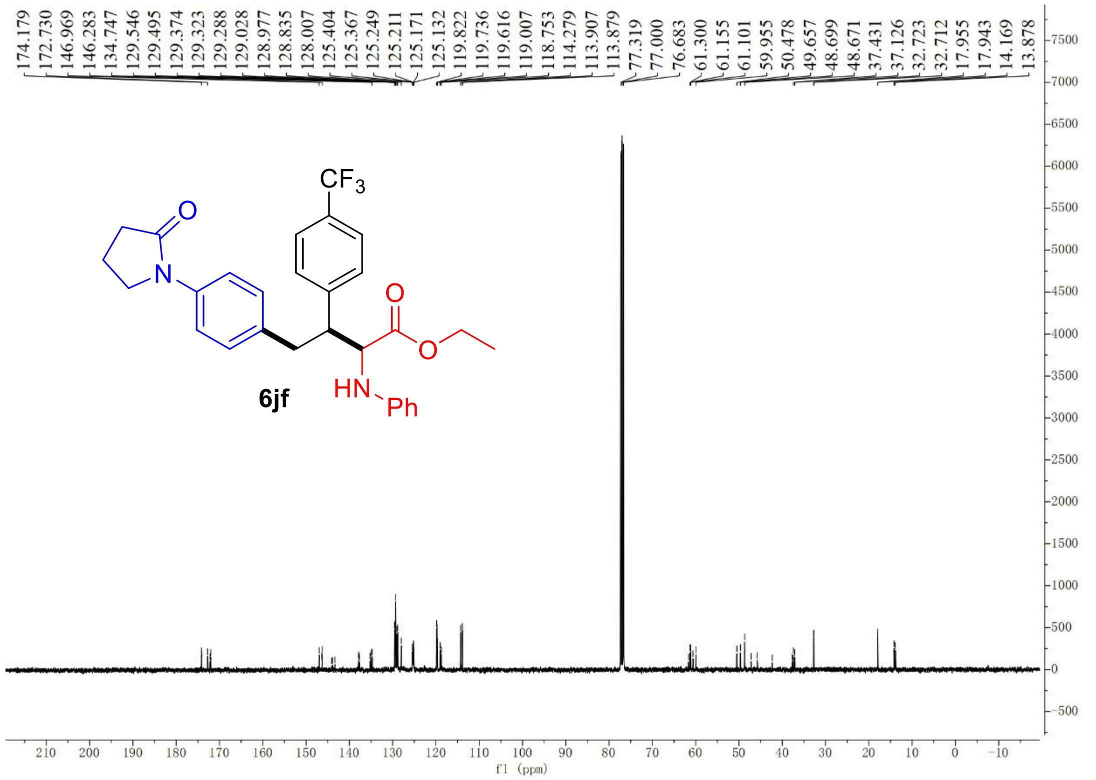
174.179
172.730
146.969
146.283
134.747
129.546
129.495
129.374
129.323
129.288
129.028
128.977
128.835
128.007
125.404
125.367
125.249
125.211
125.171
125.132
119.822
119.736
119.616
119.007
118.753
114.279
113.907
113.879
77.319
77.000
76.683
61.300
61.155
61.101
59.955
50.478
49.657
48.699
48.671
37.431
37.126
32.723
32.712
17.955
17.943
14.169
13.878
CF3
O
N
O
O
HN
Ph
6jf
f1 (ppm)

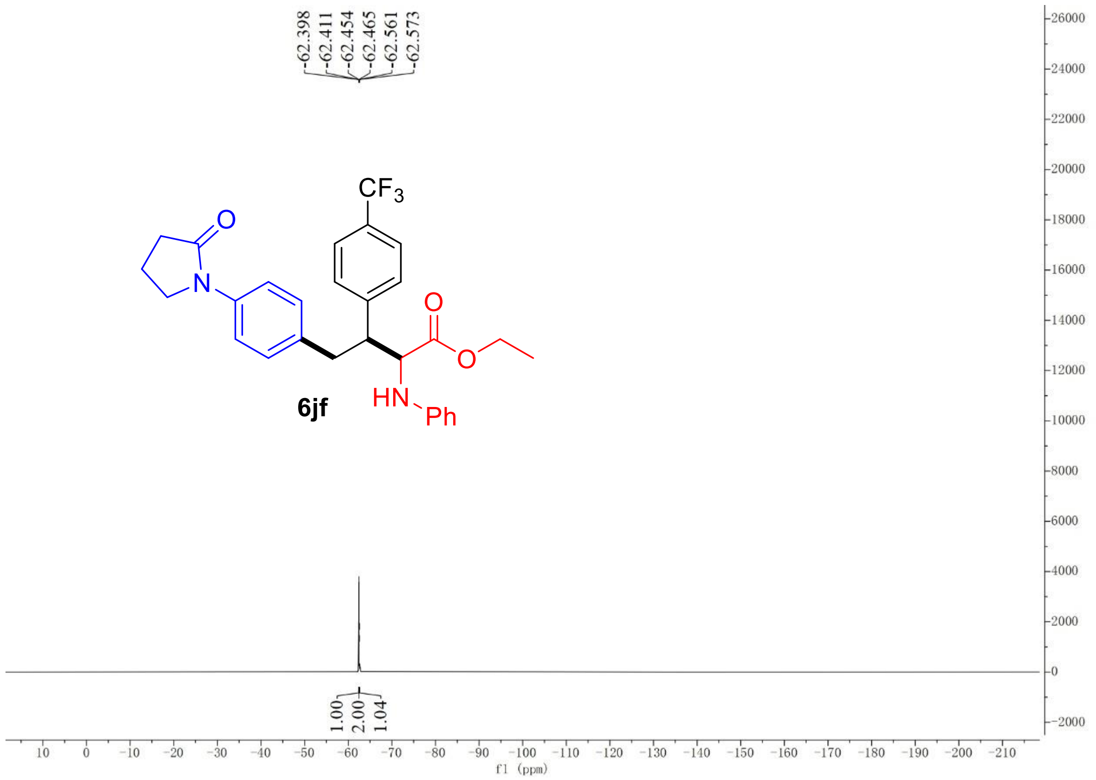
-62.398
-62.411
-62.454
-62.465
-62.561
-62.573
CF3
O
N
O
O
HN
Ph
6jf
1.00
2.00
1.04
f1 (ppm)

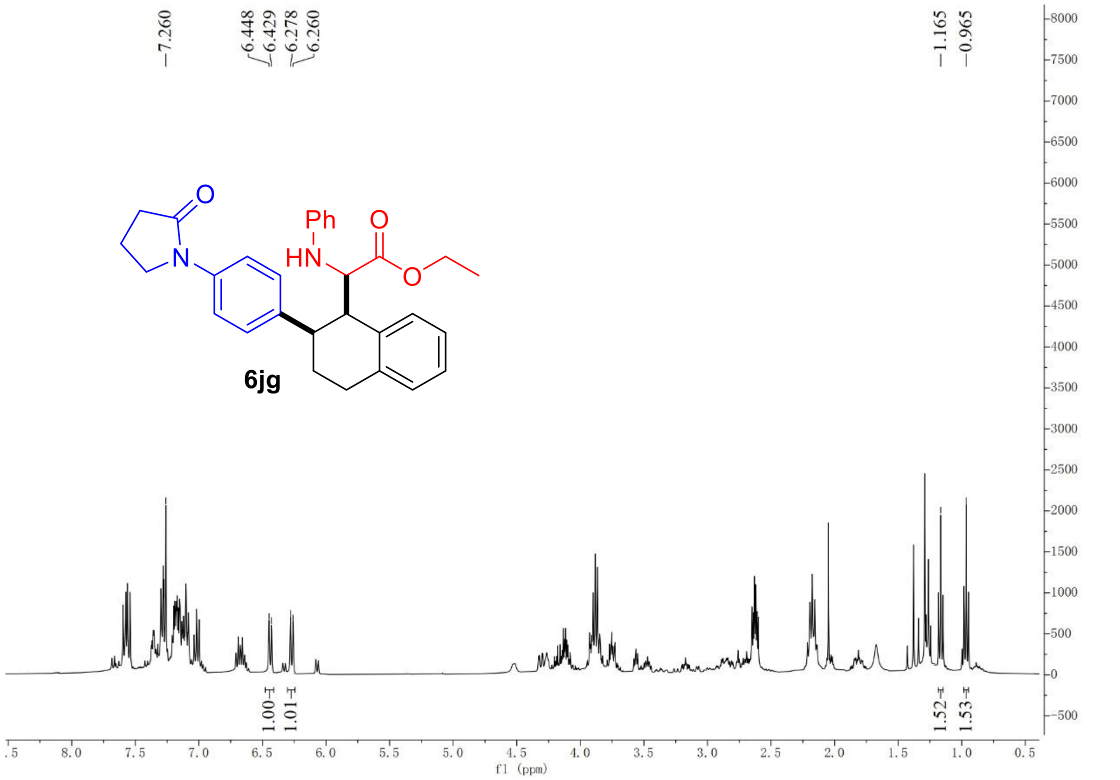
6jg
f1 (ppm)

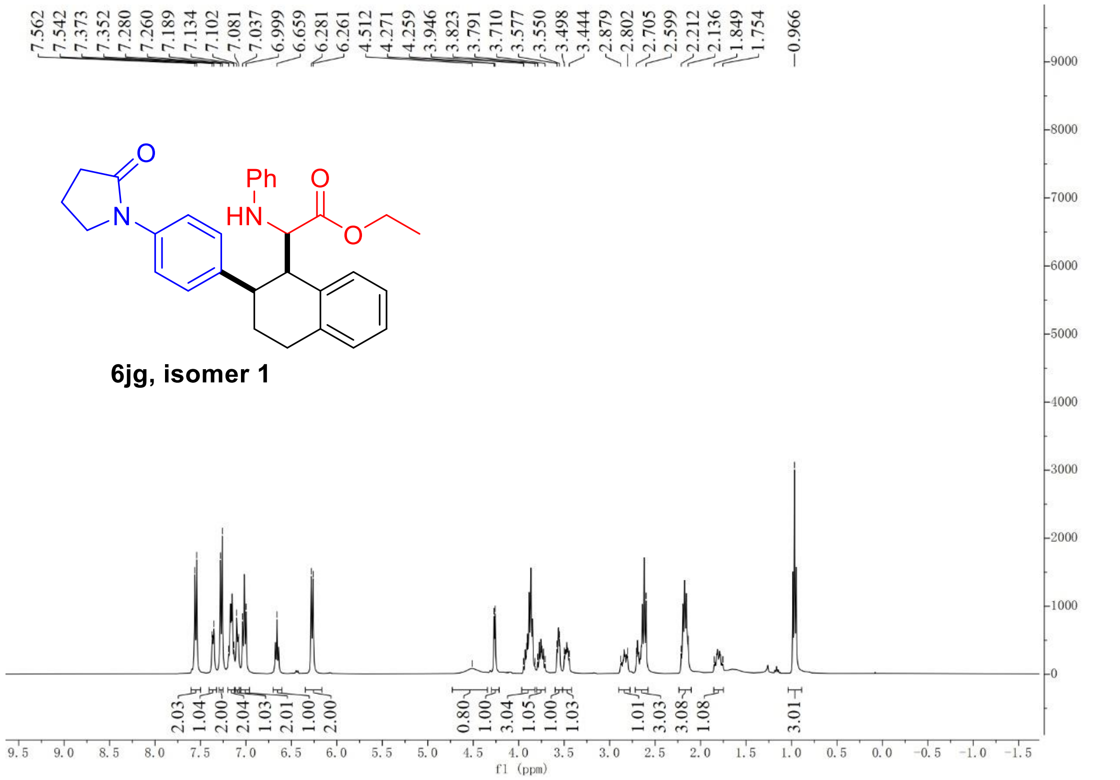
6jg, isomer 1
f1 (ppm)

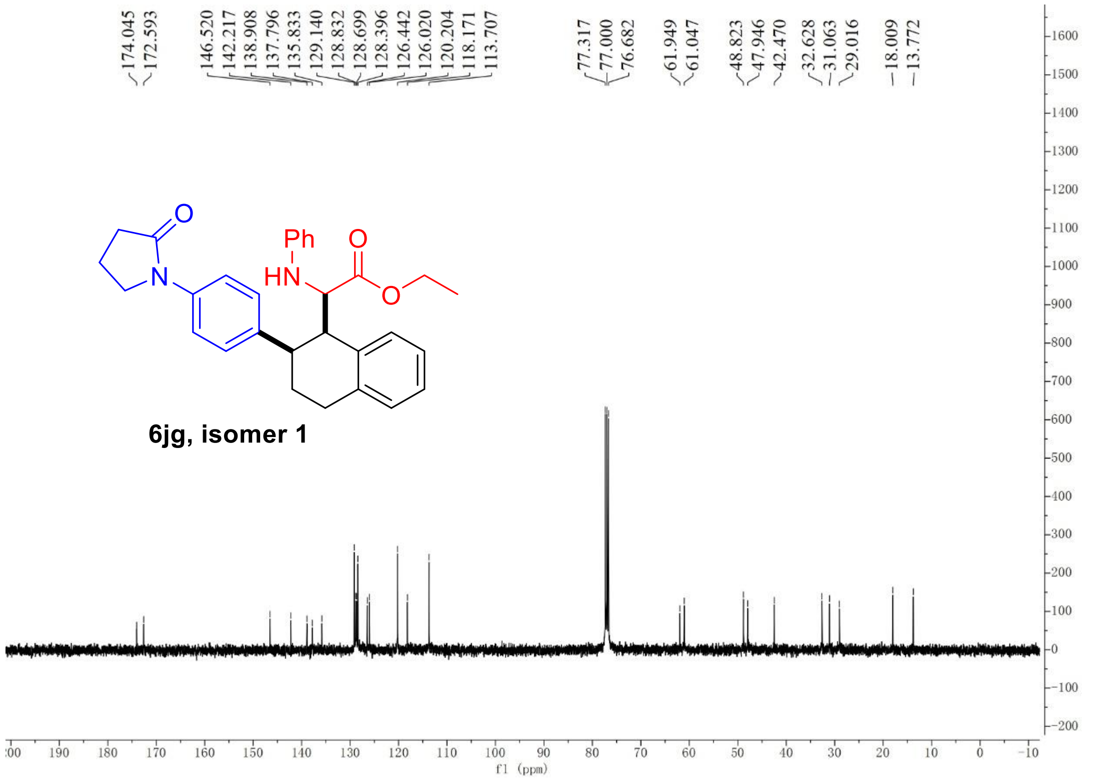
174.045
172.593
146.520
142.217
138.908
137.796
135.833
129.140
128.832
128.699
128.396
126.442
126.020
120.204
118.171
113.707
77.317
77.000
76.682
61.949
61.047
48.823
47.946
42.470
32.628
31.063
29.016
18.009
13.772
Ph
HN
O
N
O
6jg, isomer 1
f1 (ppm)

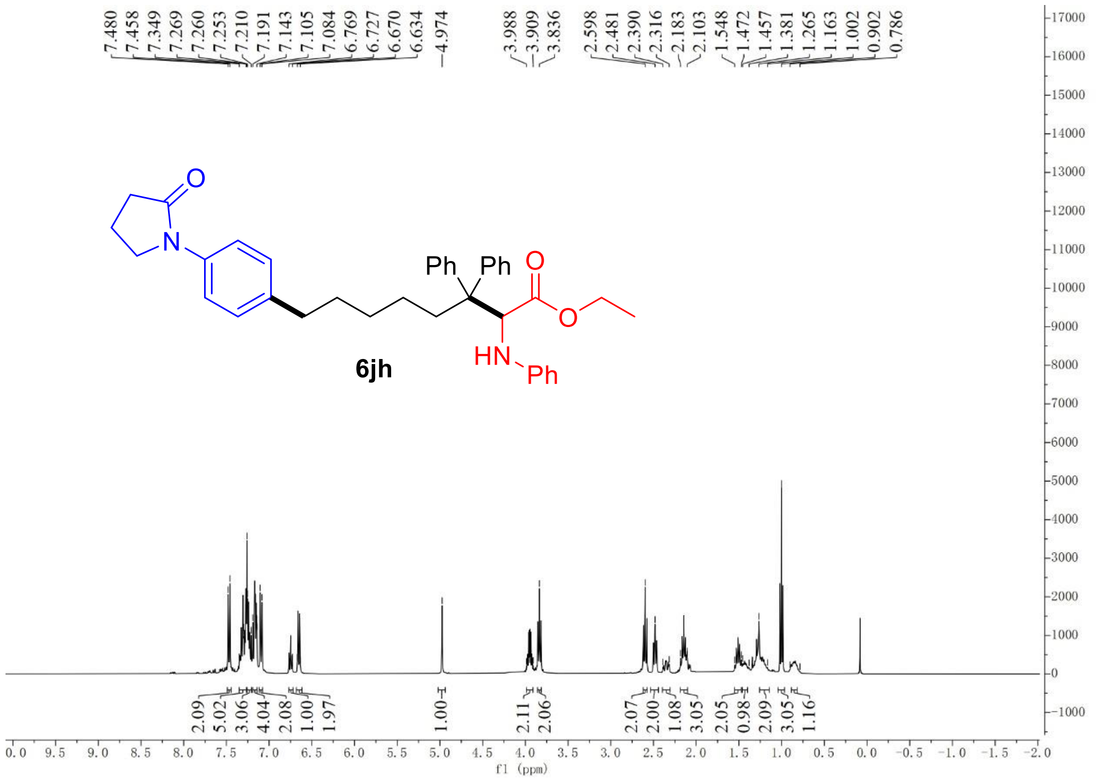
7.480
7.458
7.349
7.269
7.260
7.253
7.210
7.191
7.143
7.105
7.084
6.769
6.727
6.670
6.634
4.974
3.988
3.909
3.836
2.598
2.481
2.390
2.316
2.183
2.103
1.548
1.472
1.457
1.381
1.265
1.163
1.002
0.902
0.786
Ph
Ph
O
N
O
HN
Ph
6jh
2.09
5.02
3.06
4.04
2.08
1.00
1.97
1.00
2.11
2.06
2.07
2.00
1.08
3.05
2.05
0.98
2.09
3.05
1.16
f1 (ppm)

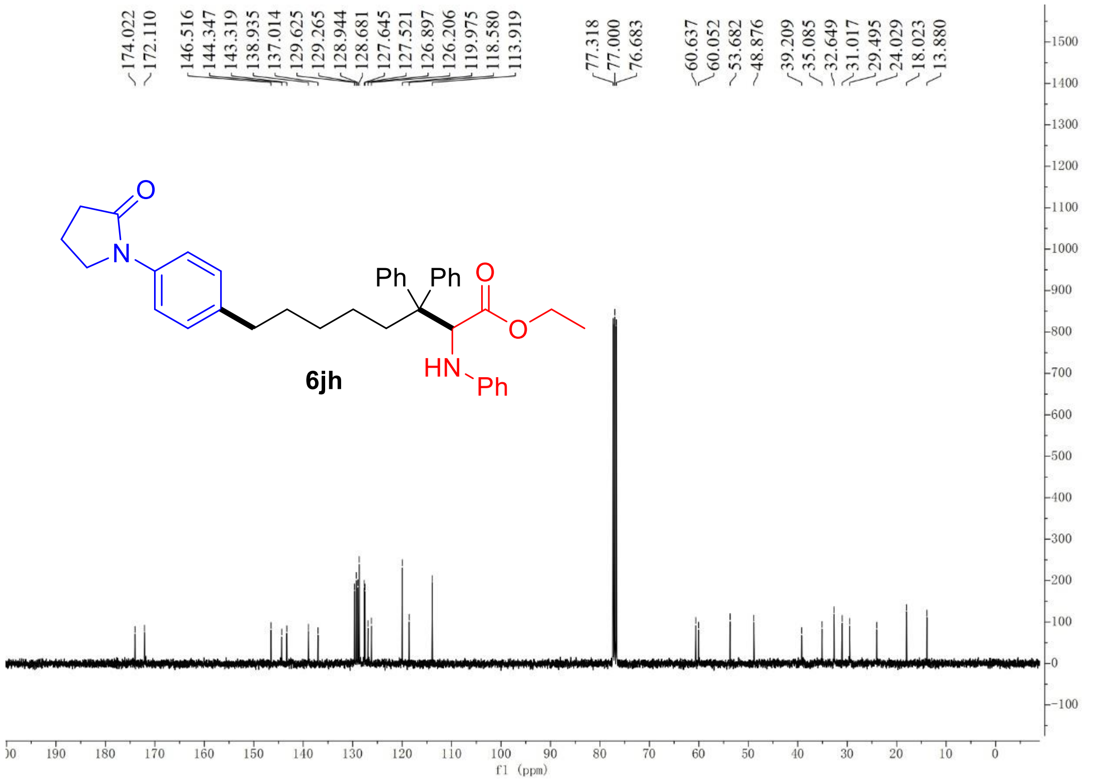

174.022
172.110
146.516
144.347
143.319
138.935
137.014
129.625
129.265
128.944
128.681
127.645
127.521
126.897
126.206
119.975
118.580
113.919
77.318
77.000
76.683
60.637
60.052
53.682
48.876
39.209
35.085
32.649
31.017
29.495
24.029
18.023
13.880
6jh
f1 (ppm)

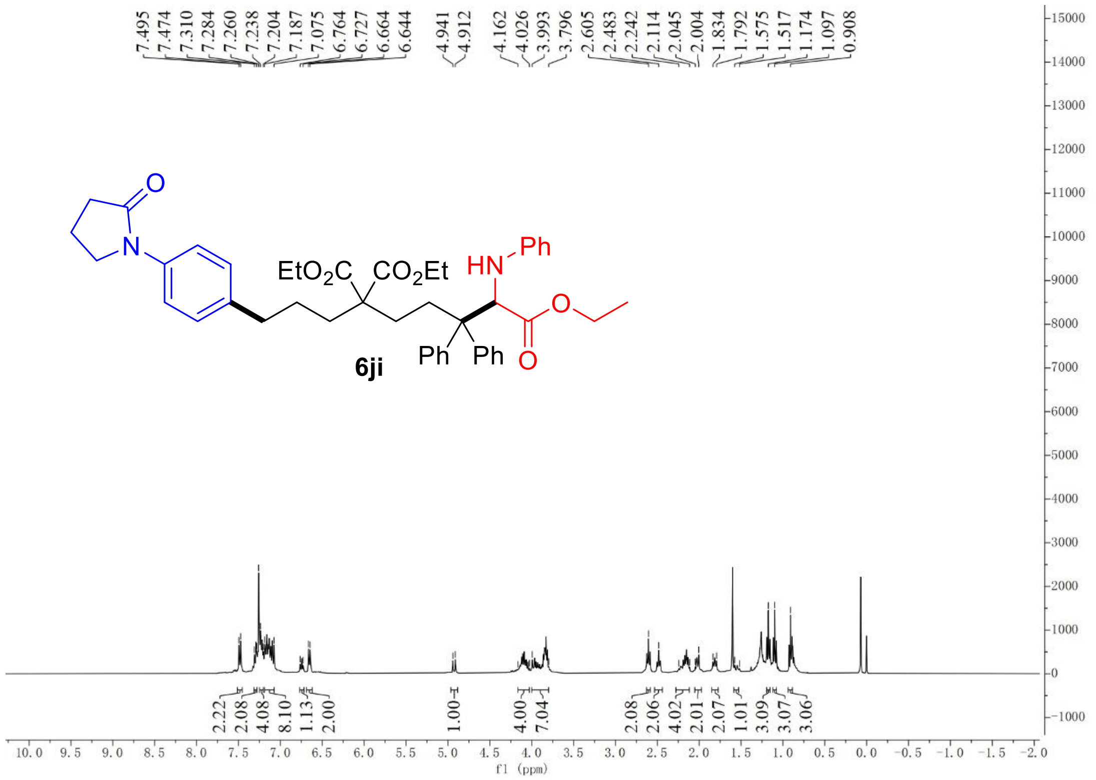

7.495
7.474
7.310
7.284
7.260
7.238
7.204
7.187
7.075
6.764
6.727
6.664
6.644
4.941
4.912
4.162
4.026
3.993
3.796
2.605
2.483
2.242
2.114
2.045
2.004
1.834
1.792
1.575
1.517
1.174
1.097
0.908
6ji
2.22
2.08
4.08
8.10
1.13
2.00
1.00
4.00
7.04
2.08
2.06
4.02
2.01
2.07
1.01
3.09
3.07
3.06
f1 (ppm)

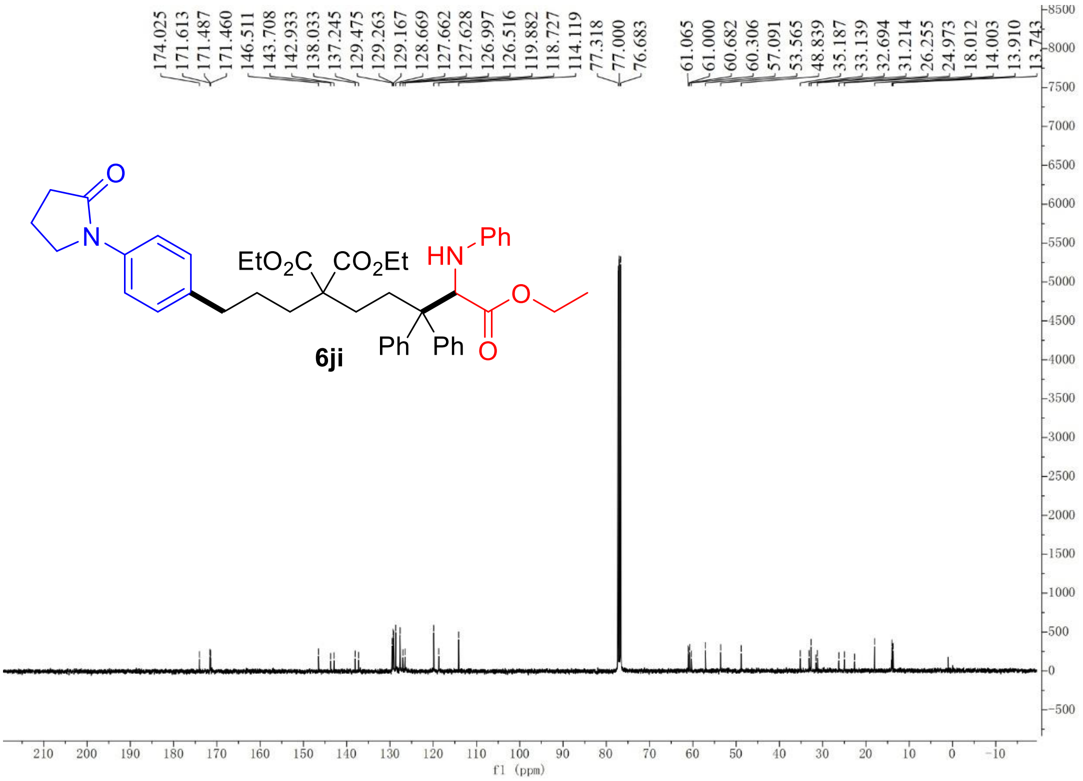

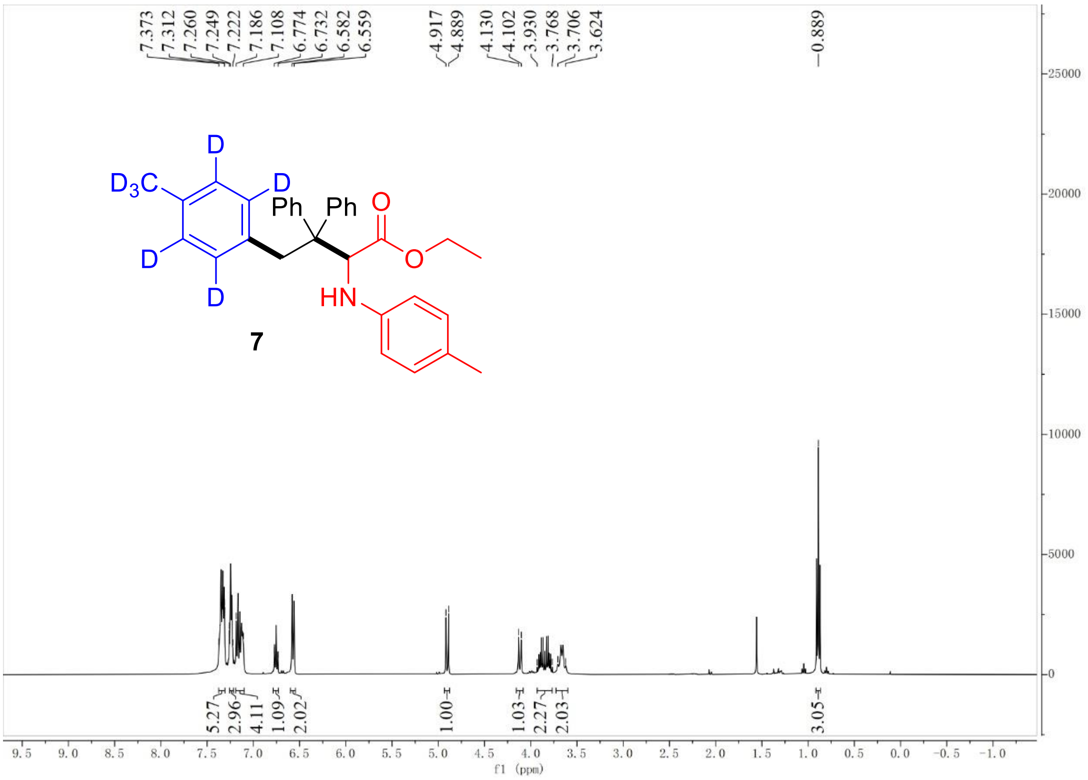

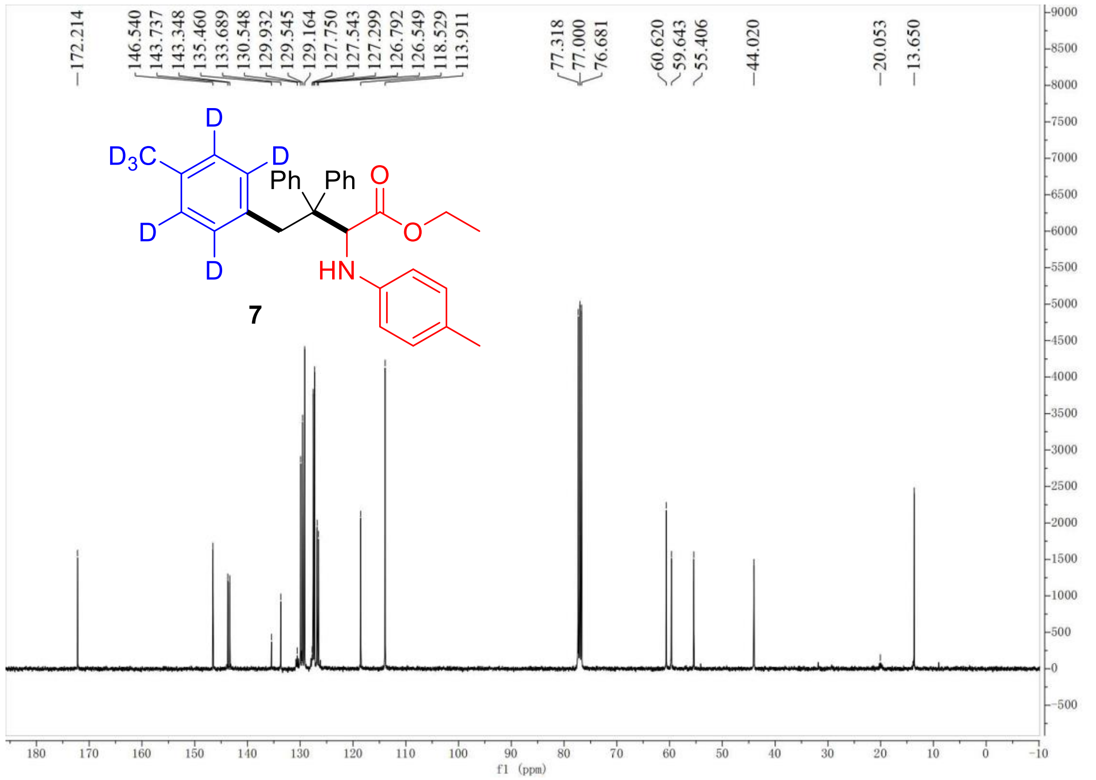
7
f1 (ppm)

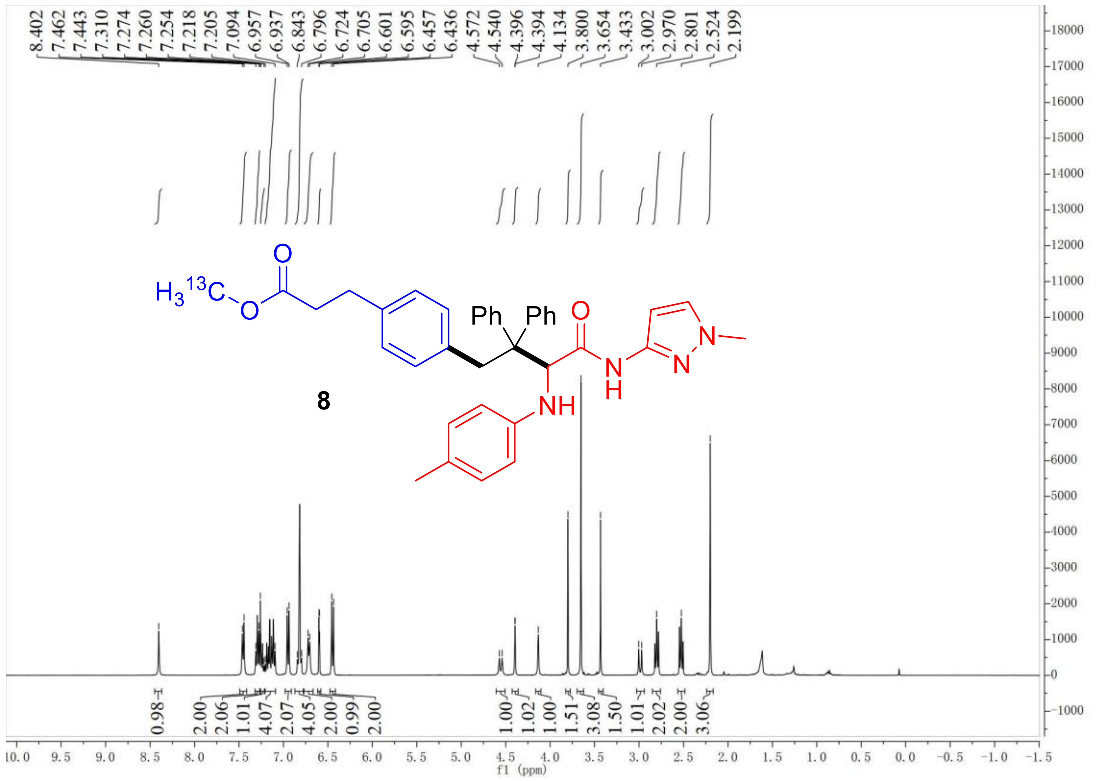
8
f1 (ppm)

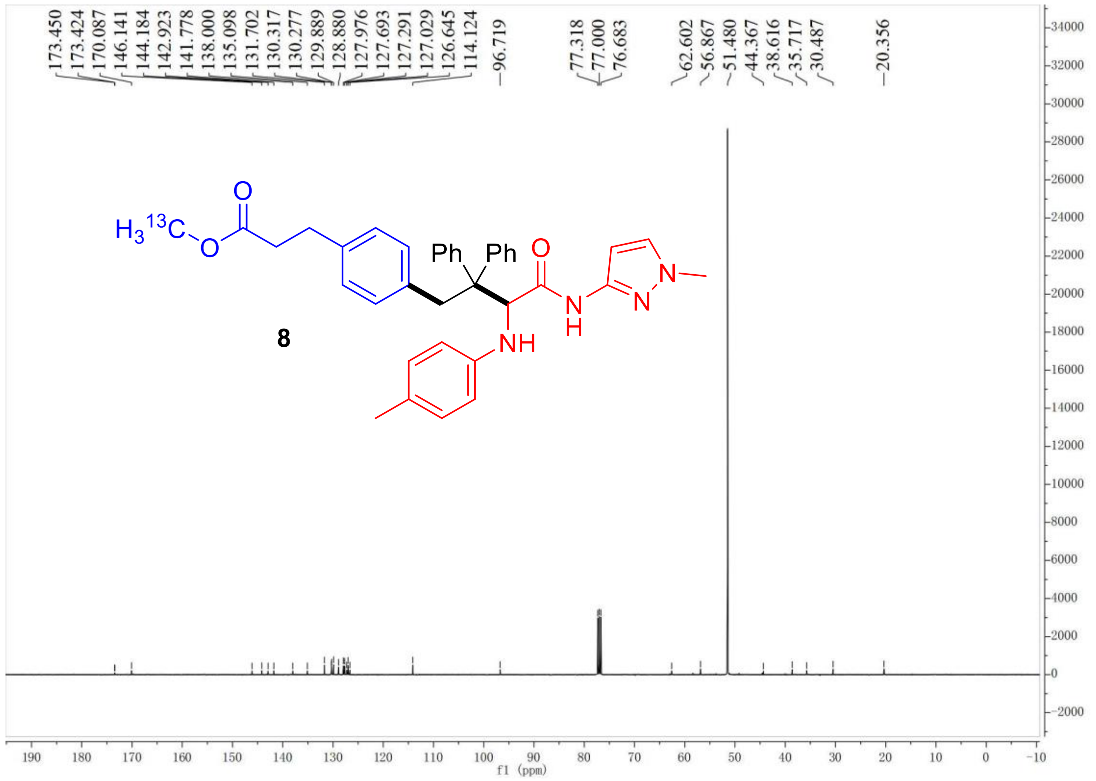
173.450
173.424
170.087
146.141
144.184
142.923
141.778
138.000
135.098
131.702
130.317
130.277
129.889
128.880
127.976
127.693
127.291
127.029
126.645
114.124
96.719
77.318
77.000
76.683
62.602
56.867
51.480
44.367
38.616
35.717
30.487
20.356
8
f1 (ppm)

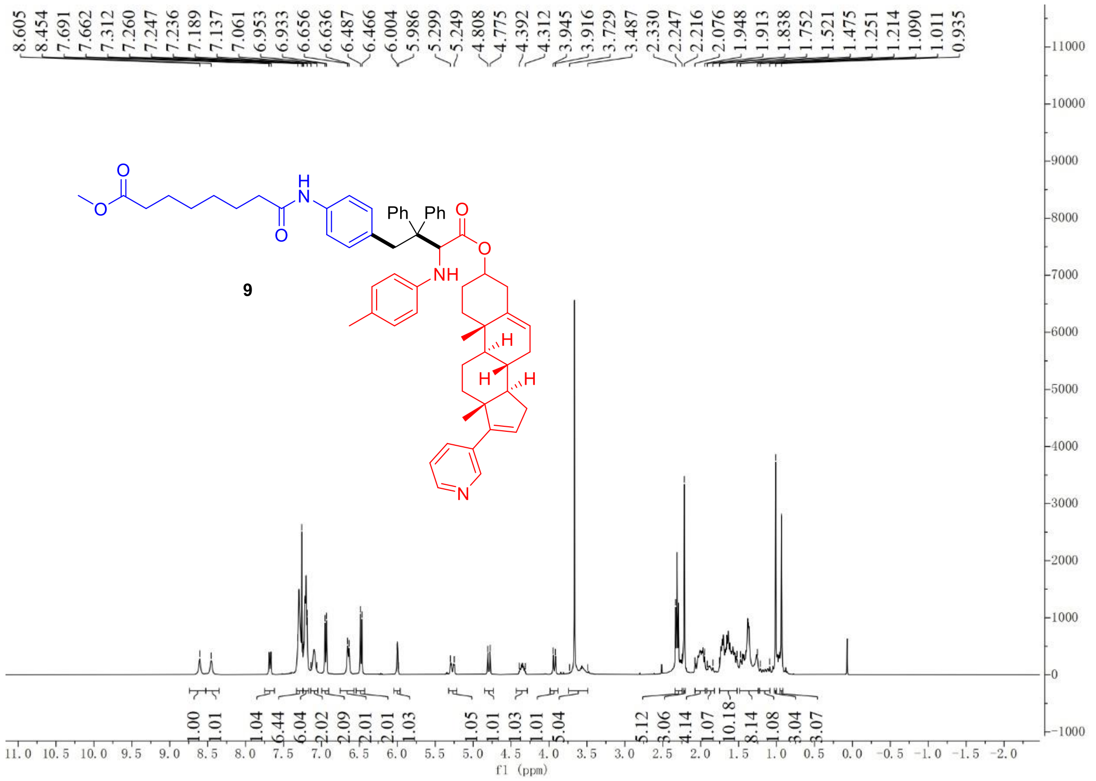
8.605
8.454
7.691
7.662
7.312
7.260
7.247
7.236
7.189
7.137
7.061
6.953
6.933
6.656
6.636
6.487
6.466
6.004
5.986
5.299
5.249
4.808
4.775
4.392
4.312
3.945
3.916
3.729
3.487
2.330
2.247
2.216
2.076
1.948
1.913
1.838
1.752
1.521
1.475
1.251
1.214
1.090
1.011
0.935
9
1.00
1.01
1.04
6.44
6.04
2.02
2.09
2.01
2.01
1.03
1.05
1.01
1.03
1.01
5.04
5.12
3.06
4.14
1.07
10.18
8.14
1.08
3.04
3.07
f1 (ppm)

Supporting Information

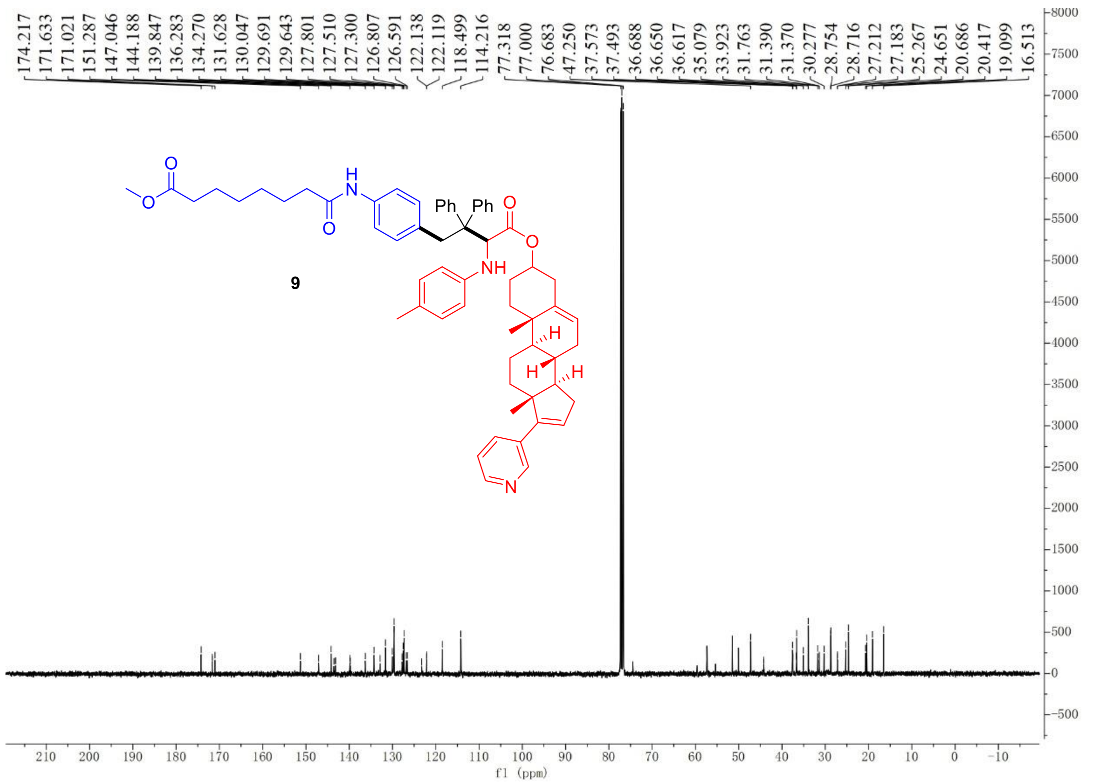

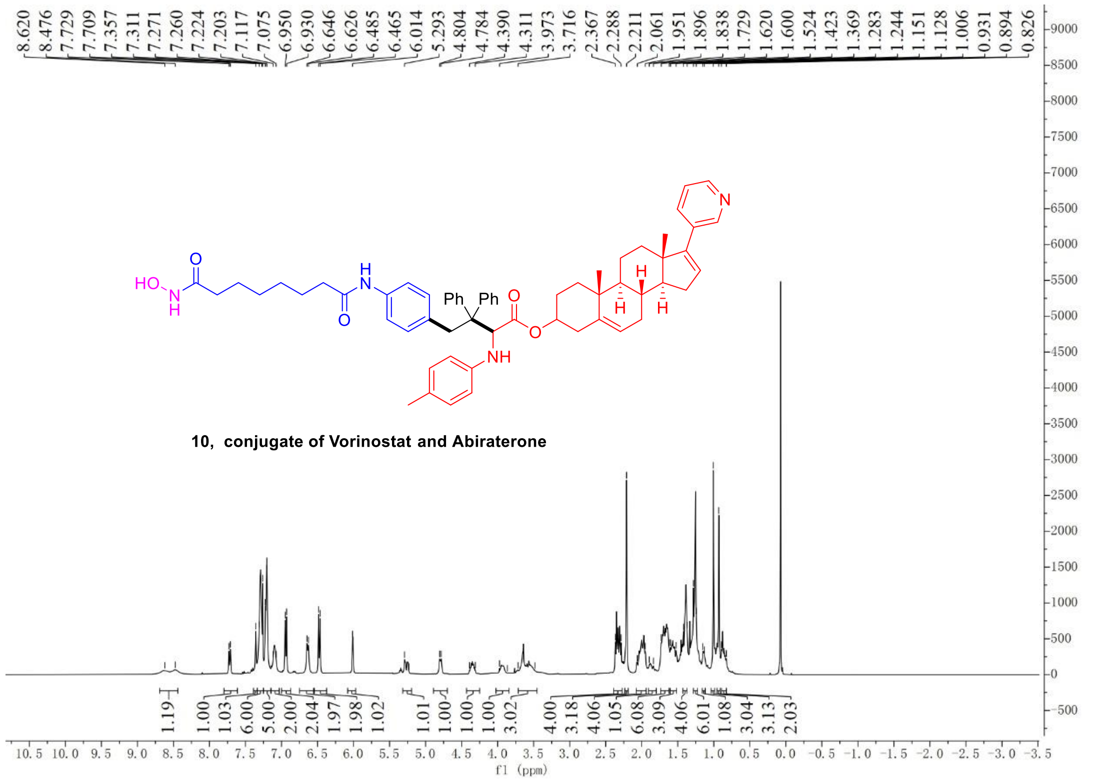

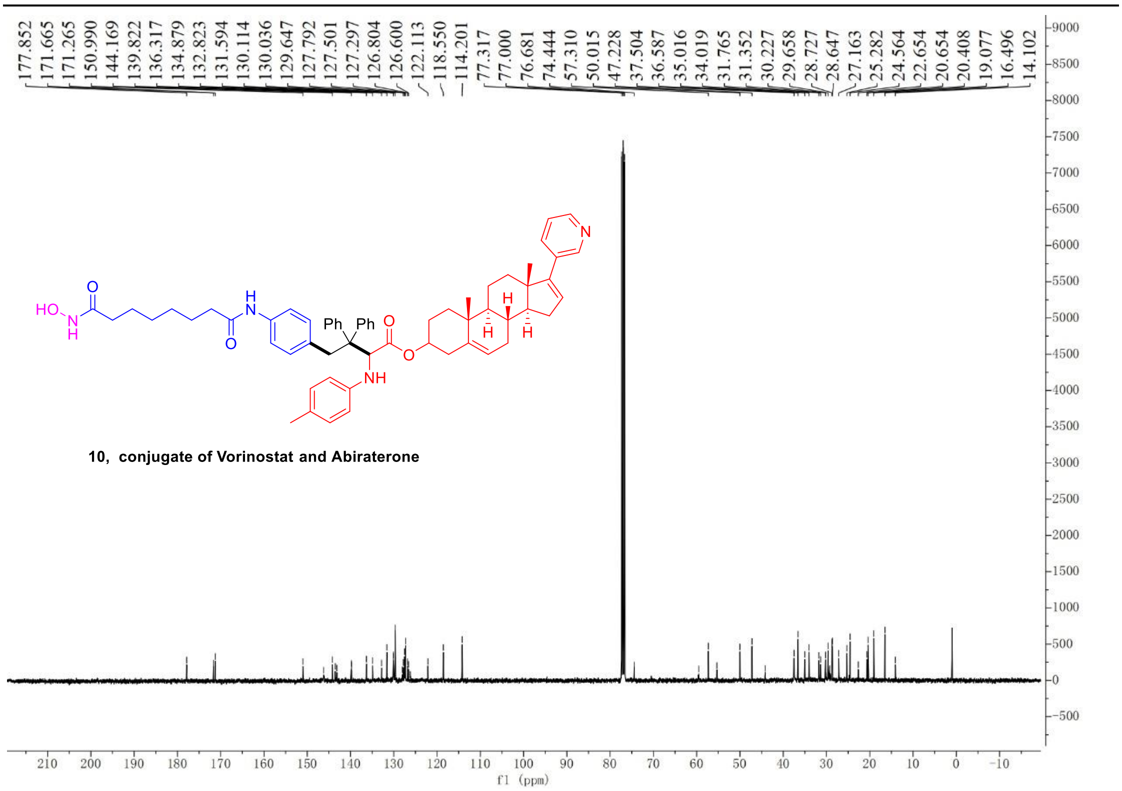

10, conjugate of Vorinostat and Abiraterone
